# Sequencing genomes from mixed DNA samples - evaluating the metagenome skimming approach in lichenized fungi

**DOI:** 10.1038/s41598-017-14576-6

**Published:** 2017-11-02

**Authors:** Anjuli Meiser, Jürgen Otte, Imke Schmitt, Francesco Dal Grande

**Affiliations:** 1 Institute of Ecology, Evolution and Diversity, Goethe University Frankfurt, Max-von-Laue Str. 13, D-60438 Frankfurt, Germany; 2 Senckenberg Biodiversity and Climate Research Centre (SBiK-F), Senckenberganlage 25, D-60486 Frankfurt, Germany

## Abstract

The metagenome skimming approach, i.e. low coverage shotgun sequencing of multi-species assemblages and subsequent reconstruction of individual genomes, is increasingly used for in-depth genomic characterization of ecological communities. This approach is a promising tool for reconstructing genomes of facultative symbionts, such as lichen-forming fungi, from metagenomic reads. However, no study has so far tested accuracy and completeness of assemblies based on metagenomic sequences compared to assemblies based on pure culture strains of lichenized fungi. Here we assembled the genomes of *Evernia prunastri* and *Pseudevernia furfuracea* based on metagenomic sequences derived from whole lichen thalli. We extracted fungal contigs using two different taxonomic binning methods, and performed gene prediction on the fungal contig subsets. We then assessed quality and completeness of the metagenome-based assemblies using genome assemblies as reference which are based on pure culture strains of the two fungal species. Our comparison showed that we were able to reconstruct fungal genomes from uncultured lichen thalli, and also cover most of the gene space (86–90%). Metagenome skimming will facilitate genome mining, comparative (phylo)genomics, and population genetics of lichen-forming fungi by circumventing the time-consuming, sometimes unfeasible, step of aposymbiotic cultivation.

## Introduction

In recent years, the decreasing costs and higher accessibility of high-throughput DNA sequencing technologies have revolutionized microbial ecology research. Direct sequencing of genomic material from the environment, commonly referred to as metagenomics, can provide a cultivation-independent assessment of the largely untapped genetic diversity and functional aspects of microbial communities. Whole-metagenome shotgun sequencing has been applied to study diverse microbiomes, spanning a range of natural environments, including the human body^1–3^. Metagenomics has not only been used to catalogue diversity, but it has also provided a fresh perspective on our understanding of the intricate, multi-species interactions driving symbiotic communities, and how these interactions influence ecosystems^4^. On the other hand, the conversion of these large volumes of sequencing data to biologically useful information remains a major challenge^5^.

With the improvement of bioinformatics tools, it is increasingly possible to assemble whole genomes from environmental communities of both prokaryotes and eukaryotes, and analyse their strain-level variation^6^. Although research on metagenomic assembly is still in its infancy, valuable insights have already been derived^7^. The annotation of metagenomic contigs from multi-species communities has proven useful to study evolutionary patterns, metabolic complementation, genetic exchange and/or modification between symbionts and their hosts in several symbiotic systems. The reconstruction of individual genomes from multi-species communities has also been used to isolate genes associated with the biosynthesis of novel biomolecules^8^. Assembly and annotation of sequencing data, however, pose several analytical challenges^9^. In particular, the co-occurrence of multiple strains or similar species – sometimes present at highly uneven ratios – may drastically reduce the quality of the reconstructed genomes^10^.

The lichen symbiosis is an example of a multi-species symbiotic assemblage, which we begin to understand much better since the advent of next-generation sequencing technologies^11^. In fact lichens are not simply an obligate association between a fungal (mycobiont) and a photosynthetic partner (photobiont), which can be either a cyanobacterium and/or a green alga^12^. The long-lived thalli of lichens constitute microhabitats harbouring a surprisingly high diversity of other eukaryotic and prokaryotic (both bacteria and archaea) microorganisms whose function has not yet been established^13,14^. Furthermore, next-generation sequencing data revealed the large extent to which multiple fungal species, and multiple photobiont lineages can be present within the same lichen individual^15–17^. Lichen-forming fungi are also relevant in natural product research as they produce a vast array of natural compounds many of which are bioactive^18–21^. To tackle evolutionary, ecological and biotechnological aspects of the lichen symbiosis, researchers have begun implementing metagenomic tools^22–26^. This is particularly relevant for studying lichen-forming fungi because these fungi are tedious to isolate^27^. Aposymbiotic cultivation of lichenized fungi is impeded by i) unknown culture conditions, ii) external fungal and bacterial contamination, iii) slow growth rates. Further, due to the obligate nature of the lichen symbiosis, for many lichen-forming fungi aposymbiotic culturing might not be possible at all^28^. For these reasons, metagenomic tools represent a promising, culture-independent approach to obtain genetic information on the lichen-forming fungi. However, we know little about the challenges and potential biases affecting the genomic assembly of metagenomic reads from whole lichen thalli.

The use of a single sequencing library layout (“metagenome skimming”) has been proven a viable approach to reconstruct genomes of the individual lichen symbiotic components, in particular the fungus^22,29^. Two approaches have been implemented, i) sequencing putative fungal DNA from portions of thalli from which algal and other possible contaminants had been manually removed^30^, ii) sequencing DNA isolated from whole thalli and extracting putative fungal contigs bioinformatically^29^. The first approach is not always feasible, because the morphology of many species precludes the physical separation of fungal and algal (and other potentially contaminating) cells. The second approach has two main disadvantages: i) assembly strategy depends on the individual experimental set-up, and particular attention should be paid to data with extreme coverage biases, ii) quality of the resulting fungal contig set depends on the assignment method, and on the database used for taxonomic assignment. As the number of studies utilising the metagenome skimming approach is destined to increase in the future, it is important to evaluate accuracy, completeness, and reliability of the method in reconstructing fungal genomes from whole lichen thalli.

Here we assessed the general applicability of the metagenome skimming approach for reconstructing the genome of lichen-forming fungi from whole thalli. For this purpose, we compared fungal assemblies extracted from metagenomic contig sets with the genomes obtained from pure cultures of the respective fungal species. As study systems we chose two lichens, *Evernia prunastri* (also known as oak moss) and *Pseudevernia furfuracea* (also known as tree moss), which are used in the fragrance industry^31,32^. Specifically, we addressed the following research questions: (i) Can metagenome assemblies be used to retrieve the fungal genome and gene space of a lichen-forming fungus? (ii) To what extent is contamination affecting our ability to reconstruct genomes of lichenized fungi from metagenomic samples?

## Material and Methods

### Fungal cultures and genome sequencing

The culture of the lichen-forming fungus *P. furfuracea* was obtained from the AKITA culture collection (collection number 0122 M). The culture of the lichen-forming fungus *E. prunastri* was obtained by picking single vegetative hyphal cells from a squash preparation of a lichen thallus using a micromanipulator following the protocol by Beck & Koop^33^. Details of the materials are given in Table 1. We grew the two fungal cultures on malt yeast extract medium. Cultures were kept in darkness in a climate chamber at 16 °C. We sub-cultured every two to three months onto fresh medium until sufficient biomass (~1 g) for genome sequencing was obtained.

**Table 1:** Specimen information and accession numbers of genomes and transcriptomes generated in this study (FR: Herbarium Senckenbergianum, Senckenberg Forschungsinstitut und Naturmuseum, Frankfurt/M, Germany; AKPM: Akita Prefectural Museum, Japan).

Species	Type of data generated	Source of DNA	Voucher information	Herbarium/culture code	NCBI accession number
*Evernia prunastri*	genome	fungal culture	Spain, 28048 Madrid, Fuencarral-El Pardo, 621 m; 40.48822, −3.75026; leg. F. Dal Grande & P. K. Divakar June, 2012	Imke Schmitt lab, SB iK-F, C 0001	NKYR00000000
*Evernia prunastri*	metagenome	thallus	Norway, Jeløya, 1519 Moss, 19 m; 59.42553, 10.60794; | eg. F. Dal Grande & G. Singh, August 2012	FR-0265082	SRS2339650
*Evernia prunastri*	metatranscriptome	thallus	Germany, Hesse, 60388 Frankfurt/Main, Bornweidstraße 42, 102 m; 50.148683, 8.758133; leg. I. Schmitt, January 2014	FR-0265083	SRS2339648
*Pseudevernia furfuracea*	genome	fungal culture	Slovenia, 17.10.1996, leg. Isao Yoshimura, originated from thallus; olivetoric acid chemotype	AKPM 0122M	NKYQ00000000
*Pseudevernia furfuracea*	metagenome	thallus	Germany, Hesse, Taunus, Großer Feldberg, 61440 Schmitten, 861m; 50.233780, 8.459419; leg. F. Dal Grande & I. Schmitt October 2012; physodic acid chemotype	N.A.	SRS2339646
*Pseudevernia furfuracea*	metatranscriptome	thallus	Germany, Hesse, Taunus, Großer Feldberg, 61440 Schmitten, 861 m; 50.233780, 8.459419; leg. F. Dal Grande & I. Schmitt, July 2013; physodic acid chemotype	FR-0265084	SRS2339645
*Pseudevernia furfuracea*	metatranscriptome	thallus	Spain, Guadalajara, 19223 Majaelra yo, 1359 m; 41.141758, -3.306956; leg. A. Crespo, F. Dal Grande & P. K. Divakar, June 2012; olivetoric acid chemotype	FR-0265085	SRS2339647

We isolated genomic DNA from each mycobiont culture following the CTAB Maxi-prep method^34^ after grinding the mycelium in liquid nitrogen with a mortar and pestle. The DNA was further purified with the PowerClean DNA Clean-Up Kit (MO BIO, Carlsbad, CA, USA) and sequenced using different platforms and library layouts. For the culture of *E. prunastri* we sequenced the following libraries: 300 bp paired-end library, 800 bp paired-end library and 3 kbp mate-pair library, on Illumina HiSeq (100 bp × 2). For the culture of *P. furfuracea* we sequenced the following libraries: 300 bp paired-end library on Illumina MiSeq (300 bp × 2) and two mate-pair libraries (3 kbp and 8 kbp) on Illumina HiSeq (150 bp × 2).

### Sequencing of metagenomes and metatranscriptomes

For the metagenomes, we sequenced genomic DNA isolated from whole lichen thalli of *E. prunastri* and *P. furfuracea* (one thallus each). Voucher information is given in Table 1. We washed the thalli thoroughly with sterile water, and checked under the stereomicroscope that thalli were free from visible parasitic infections. We isolated and purified genomic DNA as described above. For metagenome sequencing we chose a single library layout (250 bp × 2 Illumina MiSeq).

Additionally, we sequenced the metatranscriptome of *E. prunastri* and *P. furfuracea* to provide RNA-based evidence for improving gene model prediction. For *P. furfuracea* we isolated RNA from both chemical variants (i.e., chemotypes) of the species, the olivetoric acid and the physodic acid chemotypes. Whole lichen thalli were collected and stored directly in RNAlater (Sigma-Aldrich Chemie GmbH, Munich, Germany) (Table 1). Total RNA was isolated using the method described by Rubio-Piña & Zapata-Pérez^35^ after blotting the thalli dry and grinding them in liquid nitrogen with a mortar and pestle. The isolated poly-A^+^ RNA was further purified with the RNeasy MinElute Clean-up Kit (Qiagen, Hilden, Germany), and sequenced on Illumina MiSeq at StarSeq (Mainz, Germany) and a 250 bp paired-end library for both *P. furfuracea* chemotypes, and a 300 bp paired-end library for *E. prunastri*.

### Reference genome assemblies from culture

Reads from pure fungal cultures were adapter- and quality trimmed as follows: for the paired-end libraries we used Trimmomatic^36^ v0.33 with 2 seed mismatches, a palindrome clip threshold of 30 and a simple clip threshold of 10 for adaptor removal, a length cut-off of 60 for 100 bp reads and of 127 for 150 bp reads, removing leading and trailing low quality bases below a quality of 3 and a quality cut-off of an average 20 in a 5-base-wide sliding window. For the mate-pair libraries we used NxTrim^37^ v0.3.2 instead of Trimmomatic. Additionally, Sickle^38^ v1.33 was used with a 20-Phred quality threshold and length filter of 60 or 127 and *ecc.sh* in BBMap^39^ v35.14 was used with default settings for error correction. The trimmed and filtered short-insert reads of *P. furfuracea* were overlapped with PEAR^40^ v0.9.6. After some preliminary tests, we chose the best performing assembler for each species. For *E. prunastri* we used SPAdes^41^ v3.5.0 with the recommended settings *careful* and *k*-mer length *21,33,55,77*. For *P. furfuracea* we used omega^42^ v1.0.2 and a minimum overlap length of 60. We scaffolded contigs with SSPACE^43^ v3.0 and used GapFiller^44^ v1.10 to close remaining gaps. The resulting scaffolds were assigned taxonomically to Ascomycota with MetaWatt^45^ v.3.5.2 to filter out potential contaminants. Assembly statistics were accessed with Assemblathon^46^ and the genome completeness was estimated based on evolutionarily-informed expectations of gene content with BUSCO v.2.0 (Benchmarking Universal Single-Copy Orthologs)^47^ and a lineage-specific set of Ascomycota single-copy orthologs from OrthoDB^48^ v.9.

### Reference gene sets from culture

We performed *de novo* gene prediction and annotation on the assemblies based on pure fungal cultures using MAKER^49^ v2.31.8 in an iterative fashion following the recommendation and protocols of Campbell *et al*.^50^ and incorporating the metatranscriptome data quality filtered with Trimmomatic v.0.36 and aligned with bowtie^51^ v2.1.0. For the first round of MAKER we used Hidden Markov Models (HMMs) gained from GeneMark-ES^52^ v4.33 and SNAP^53^ with hints from CEGMA^54^ v2.4 (performed on iPlant^55^) and included RNA evidence through a TopHat^56^ v2.0.11 GFF file. Then we converted the first-round results to new SNAP and Augustus^57^ v3.0.2 HMMs and ran MAKER again. Additionally, we rescued rejected gene models (MAKER *standard* instead of *default build*) including all gene models that were supported by RNA evidence and all *ab initio* gene models encoding a protein family (Pfam) domain detected by InterProScan^58^ v.5.23–62.0 and that did not overlap with RNA evidence. For *P. furfuracea* we used the RNA evidence originating from the olivetoric acid chemotype to match the chemotype of the fungal culture. Gene set completeness was estimated as genome completeness with BUSCO (see above).

### Metagenomic assemblies

For metagenomic reads we tested different *de novo* assemblers for whole-genome shotgun sequence data to evaluate their performance and obtain the best possible assembly^29,46^. We used a range of different *de novo* assemblers relying on the detection of overlapping reads (overlap layout graph assemblers) as well as those utilizing de Bruijn graphs and included general-purpose assemblers as well as specialized metagenome assemblers. We used the following assemblers: MIRA^59^ v4.0.2, omega, SPAdes v3.8.1, metaSPAdes^9^ v3.8.1, metaVelvet^60^ v1.2.02 and IDBA-UD^10^ v1.2.0. MIRA was run with the default flags *genome*, *denovo*, *accurate* and an auto refining template size of minimum 151 to maximum 600 on reads overlapped with PEAR. Adaptor removal and quality trimming was not performed for MIRA following the developer’s recommendation. For the other assemblers, we adapter-trimmed and quality-filtered the raw reads as described above, but using Trimmomatic v.0.36 with a length cut-off of 150 and Sickle with a length filter of 127. Trimmed and filtered reads were overlapped with PEAR and used as input to the assemblers described hereafter. SPAdes was run with the recommended settings *careful* and *k*-mer length *21,33,55,77,99,127*. MetaSPAdes was run with the flag *meta* for metagenomic samples and the same *k*-mer length settings as in SPAdes. MetaVelvet was used with Velvet^61^ v1.2.10 assembling *k*-mer sizes from 51 to 231 with a step size of 20 using an estimated mean insert size of 273 for *E. prunastri* and 267 for *P. furfuracea* as calculated with bowtie v2.2.5 and a custom Python script. No consistent paired-end connection was chosen as recommended for metagenomic samples containing very dissimilar species and the expected coverage was set to *auto* initially and set manually in a re-run after inspection of *k*-mer coverage histograms plotted with the package plotrix^62^ v3.6–4 in R^63^ v3.3.2. The optimal metaVelvet *k*-mer sizes of 191 for *E. prunastri* and of 91 for *P. furfuracea* were chosen according to the VelvetOptimiser^64^ v2.2.5 manual and Greshake *et al*.^29^ by multiplying the N50 by the number of long contigs (>1 kbp). We tested omega with minimum overlap lengths between 100 and 200 and applied the same optimisation criteria as for the metaVelvet assemblies resulting in an overlap length of 140 for *E. prunastri* and 150 for *P. furfuracea*. IDBA-UD was chosen instead of meta-IDBA^65^ as it generally performs better according to the authors and was run with *k*-mer sizes from 51 to 231 with a step size of 20. All resulting assemblies of all assemblers were filtered for a minimum length of 400 bp using a custom Perl script.

### Taxonomic assignment

We compared two different approaches to extract fungal contigs from all metagenomic assemblies. For the first approach we ran DIAMOND^66^ v0.8.34.96 BLASTx with the *more-sensitive* mode for longer sequences and a default e-value cut-off of 0.001 against the NCBI Genbank *nr* protein database^67^ (downloaded in January 2017). We parsed the results with MEGAN^68^ v.6.7.7 with *max expected* set to 1E-10 and using the weighted lowest common ancestor (LCA) algorithm which improves the specificity of taxonomic assignment compared to the naive LCA algorithm^69^. For all assemblies, we exported all contigs assigned to Ascomycota to represent the expected mycobiont^15^. An assignment to lower taxonomic rank was not possible due to the lack of closely related genomes in the reference databases. All downstream analyses refer to the extracted Ascomycota subsets.

For the second approach, we used MetaWatt. While MEGAN classifies reads based on sequence similarity by finding the LCA in the NCBI taxonomy, MetaWatt makes use of ﻿multivariate statistics of tetranucleotide frequencies and differential coverage based binning of metagenomic contigs. MetaWatt also performs taxonomic profiling of bins with DIAMOND BLASTx against a database that we customized to include non-redundant genomes of 532 Eukaryota, 1936 Bacteria, and 132 Archaea at the genus rank and 619 viruses at family rank (generated in August 2017). We calculated read coverage by aligning the quality trimmed reads with bowtie v2.2.5, converting files with samtools v.1.1^70^ and running Qualimap v.2.2.1^71^. We disabled coverage based binning since we had only one read set as recommended in the manual. To identify tetranucleotide bins that belong to the respective mycobiont we used the following approach: we selected bins with an Ascomycota profile that had at least 50% of the fragments classified as Ascomycota and no other taxa represented in their taxonomic profile. We then merged all bins that met these criteria and manually unbinned contigs that were not classified as Ascomycota or ‘Unknown’ (see Supplementary Table S1).

### Assembler evaluation

To find the best fungal assembly from metagenomic reads, we assessed quality and completeness of the assemblies extracted with MEGAN and MetaWatt using as reference genomes and gene sets the assemblies based on pure fungal cultures in QUAST^72^ v.4.1. QUAST evaluates and compares assemblies based on alignment of contigs to references. We used a lower contig length threshold of 400 and the settings *scaffolds*, *eukaryote* and *fragmented*. We considered several assembly statistics and metrics from QUAST, e.g., number of contigs, total length and N50 (see full QUAST reports in the Supplementary File S6 or S7), number of misassemblies and the fraction of reference genome and genes covered compared to the reference genome. Additionally, we confirmed the QUAST evaluation with genome assembly and gene set completeness with BUSCO as described above. BUSCO results were visualized with the package ggplot2^73^ v.2.2.1 in R.

### Gene prediction and comparisons of genes sets

For comparing the gene sets of the reconstructed genomes from metagenomic reads with the gene sets of reference fungal cultures, we applied the following three steps to the best reconstructed genomes extracted with MEGAN and MetaWatt and for both species, respectively. First, we performed a *de novo* gene prediction and annotation on the fungal contig subsets using MAKER as described above, but using the RNA evidence originating from the physodic acid chemotype for P. furfuracea to match the chemotype of the mycobiont reconstructed from metagenome.

Second, we used Reciprocal Best Blast Hits (RBH) to find orthologous pairs between the gene sets, as a simple and fast method for comparing different gene sets resulting from different assemblies of the same species^74^. We ran BLASTp^75^ v2.2.30+ using final Smith-Waterman alignment and soft filtering (BLAST flags *use_sw_tback*, *soft_masking true*, *seq yes* and *evalue 1e-6*) for better detecting orthologs as RBH^74,76^. We filtered the BLAST results for a minimum identity of 70% over the alignment length (*pident*) and a minimum query coverage of 50% (*qcovhsp*)^77^, sorted for highest bit-score and lowest e-value and removed multiple identical top hits after manual inspection as not informative in the context of RBH. We used eulerAPE^78^ v.3.0.0 to draw area-proportional Venn diagrams to visualize the overlap among gene sets.

Third, we tested if metagenome-based assemblies are reliable to study fungal gene space when a fungal culture is not available. For this purpose, we chose to analyse the gene families of secondary metabolism as they represent typical targets for focused genome mining of lichen-forming fungi^19,79^. We thus identified and annotated secondary metabolite biosynthesis gene clusters with the fungal version of antiSMASH^80^ v.4.0.2, including polyketide synthases, non-ribosomal peptide synthetases, and terpene synthases. We used an annotated nucleotide file in EMBL format as input generated from the genome FASTA file and the GFF file from MAKER with scripts provided by M. H. Medema. We compared the outputs in terms of total genes and RBHs.

### Taxonomic composition of the metagenomic read sets

We investigated the taxonomic composition of the metagenomic read sets to estimate the abundance of the target mycobionts. For this, we taxonomically classified the trimmed and corrected metagenomic reads using Kraken^81^ v.0.10.5-beta. Kraken utilises exact alignments of overlapping read *k*-mers and is one of the best tools in terms of precision and accuracy^82^. Here we took advantage of the fact that we could incorporate the reference genomes of our fungal species into a custom-build database. It was therefore possible to estimate with precision the amount of reads of the lichen-forming fungus and compare it to that of other fungi in each metagenome. The Kraken database we built thus contained our reference genomes of *E. prunastri* and *P. furfuracea*, and the entire fungal RefSeq^83^ (release 79). We further included five lichen genomes (*Cladonia macilenta*, *Cladonia metacorallifera*, *Endocarpon pusillum*, *Gyalolechia flavorubescens*, *Umbilicaria muehlenbergii*) and the basidiomycete *Cystobasidium pallidum* ^15^ from NCBI GenBank. We hard-masked the database genomes for low-complexity regions with dustmasker v.1.0.0 (part of BLAST v.2.2.30) as recommended in the manual. We then built and classified the database for a read length of 250 bp.

To identify potential sources of contamination, we further taxonomically classified all metagenomic reads that were not classified as reference mycobiont by the above-mentioned Kraken approach. For this, we ran DIAMOND BLASTx with default settings against the NCBI Genbank *nr* protein database and parsed the results in MEGAN with *max expected* set to 0.001 and using the weighted LCA algorithm. Additionally, we extracted the reads that were assigned to non-target Ascomycota in MEGAN, assembled these as described above with SPAdes and searched for the presence of biosynthetic genes in the resulting scaffolds with antiSMASH.

## Results

### Sequencing results

For *E. prunastri* we obtained a total of 104,138,074 paired-end and mate-pair reads from culture, 21,622,755 paired-end reads from metagenome and 32,706,203 paired-end reads from metatranscriptome. For *P. furfuracea* we obtained a total of 70,695,549 paired-end and mate-pair reads from culture, 21,031,517 paired-end reads from metagenome, 17,357,422 paired-end reads from the olivetoric acid chemotype metatranscriptome and 18,321,601 paired-end reads from the physodic acid chemotype metatranscriptome.

### Reference genomes and gene sets

We first obtained references genomes for *E. prunastri* and *P. furfuracea* from fungal cultures. After quality filtering and trimming, we used 85.7% *E. prunastri* reads and *P. furfuracea* 79.3% reads for genome assembly. The genome of *E. prunastri* was assembled into 277 scaffolds with total length of 40 Mbp, N50 of 264,454 bp, an average coverage of ~410x, and an estimated genome completeness of 95.9% according to BUSCO. The genome of *P. furfuracea* was assembled into 46 scaffolds with a total length of 38 Mbp, N50 of 1,178,799 bp, average coverage of ~350x, and estimated 94.7% completeness. After quality trimming, we obtained 87.9% paired-end RNA reads of *E. prunastri* of which 55.4% aligned to the genome to give hints for gene prediction. For *P. furfuracea* 90.8% RNA reads survived quality filtering and 57.1% of these mapped against the genome. We predicted 10,992 genes for *E. prunastri* and 8,842 genes for *P. furfuracea* with an estimated gene set completeness of 92.1% and 91.8%, respectively. Both reference genomes and gene sets based on the pure culture strains of *E. prunastri* and *P. furfuracea* are summarized in Table 2.

**Table 2:** Reference genome assemblies and gene sets from pure culture strains.

	*Evernia prunastri*	*Pseudevernia furfuracea*
Number of scaffolds	277	46
Total size	40 Mb	38 Mb
N50	264,454 bp	1,178,799 bp
Average coverage	~410x	~350x
Number of genes	10,992	8,842
Genome completeness	C:95.9% [S:95.1%, D:0.8%], F:2.7%, M:1.4%	C:94.7% [S:94.6%, D:0.1%], F:3.5%, M:1.8%
Gene set completeness	C:92.1% [S:91.3%, D:0.8%], F:5.1%, M:2.8%	C:91.8% [S:91.8%, D:0.0%], F:5.0%, M:3.2%

Completeness is compared against 1,315 orthologous BUSCO marker genes for Ascomycota (C:complete [S:single-copy, D:duplicated], F:fragmented, M:missing).

### Evaluation of metagenomic assemblies

**Figure 1:**
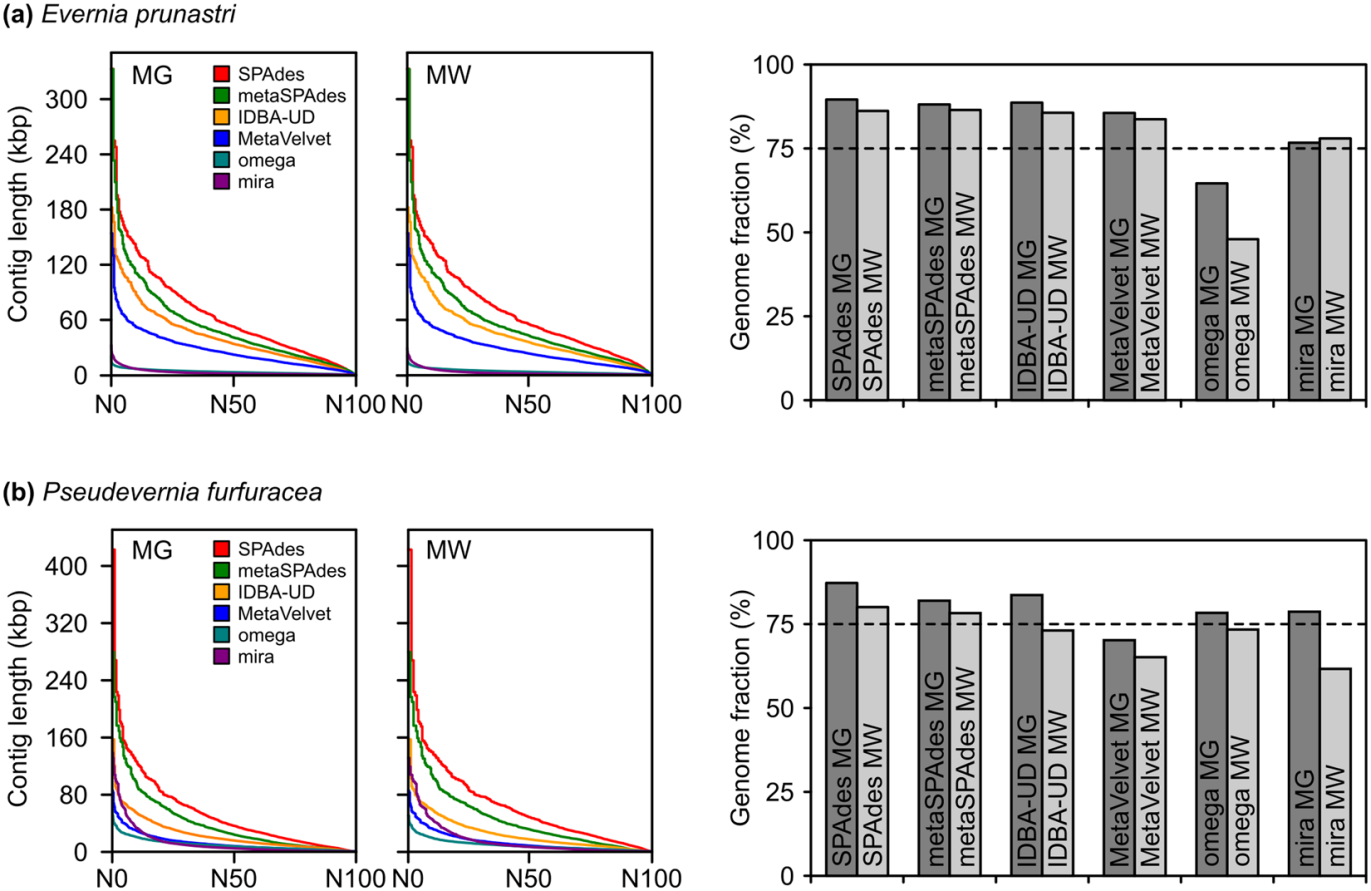
Comparison of six assembler s. Taxonomic assignment of metagenomic reads to Ascomycota was performed with MEGAN (MG) and MetaWatt (MW). N-Statistics are presented on the left side and the fractions of the fungal culture reference genome that is covered by the metagenome-based assemblies are presented on the right side. This analysis is based on QUAST. Full QUAST reports are provided in the Supplementary Files S6 and S7.

**Figure 2:**
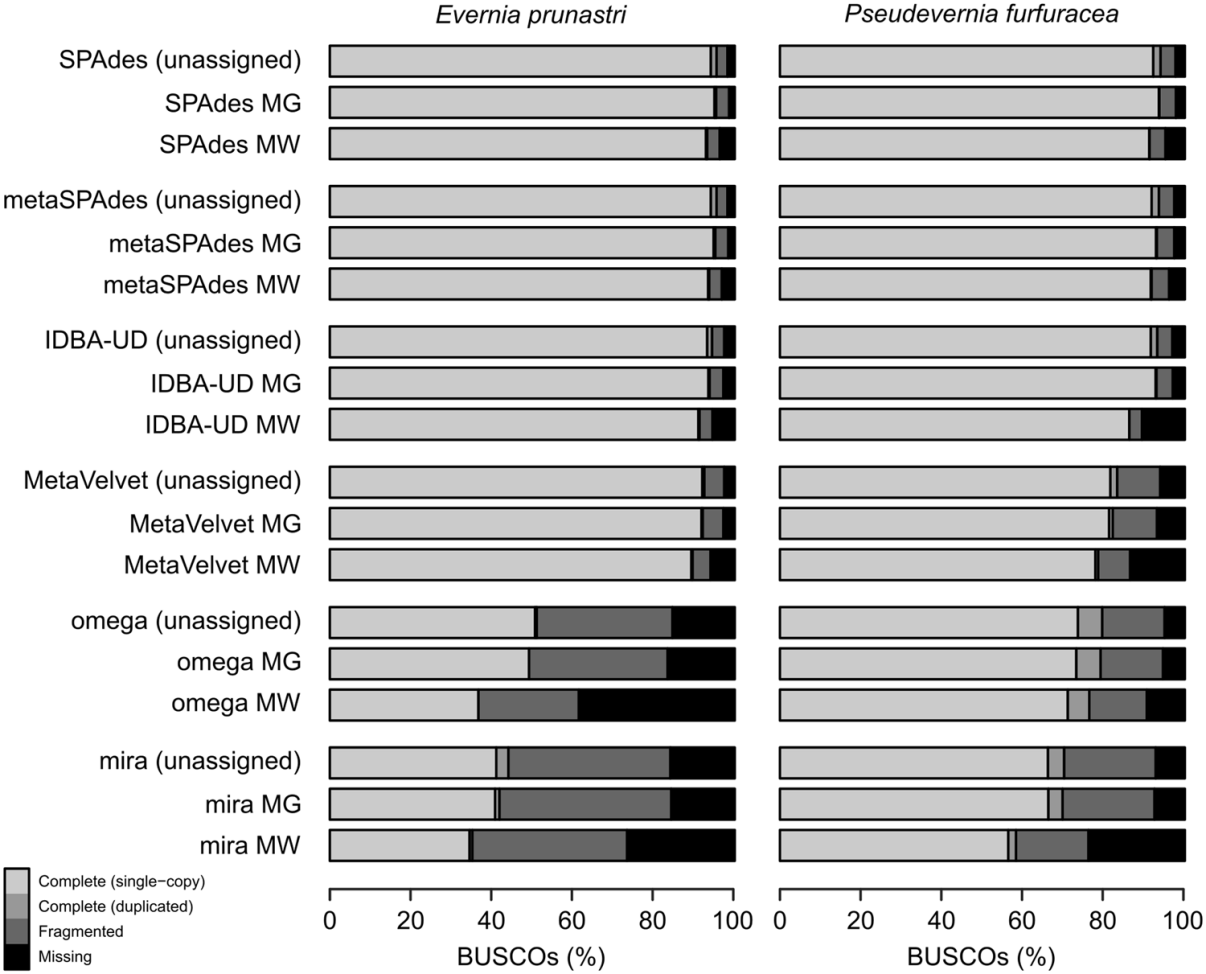
Genome completeness for genome assemblies from metagenomic lichen thalli using different assemblers. Taxonomic assignment of metagenomic reads to Ascomycota was performed with MEGAN (MG) and MetaWatt (MW). Percentage completeness is compared against 1,315 orthologous BUSCO marker genes for Ascomycota. Full BUSCO reports are provided in Supplementary Table S2.

After quality filtering and trimming, 29,573,575 (68.4%) *E. prunastri* metagenomic reads and 35,116,468 (83.4%) *P. furfuracea* metagenomic reads were used with six assemblers. We evaluated assembler performance based on the extracted fungal subset with MEGAN and MetaWatt based on overall genome statistics, overlap to reference, and fungal genome completeness. We observed highly different assemblies depending on the assembler used, while the choice of taxonomic assignment method did not yield significant differences (Fig. 1). Comparisons of N-statistics and the covered fraction of the reference genome are shown in Fig. 1. Full QUAST reports are provided in the Supplementary Files S6 and S7. SPAdes assemblies had the best assembly statistics and the highest overlap to the reference (86–90% for *E. prunastri* and 80–87% for *P. furfuracea*). MetaSPAdes was second best followed by IDBA-UD. We compared the fungal genome completeness based on BUSCO of all unassigned, MEGAN-assigned and MetaWatt-assigned assemblies in Fig. 2 (detailed values can be found in Supplementary Table S2). The assessment of completeness was based on a lineage-specific set for Ascomycota and therefore provides the possibility to access ‘potential’ completeness in the taxonomically unassigned assemblies. We observed that the fungal completeness in unassigned assemblies (95.5% for *E. prunastri*, 94.0% for *P. furfuracea*) was comparable to the MEGAN-assigned assemblies, while MetaWatt-assigned assemblies were slightly less complete (see Fig. 2 and Supplementary Table S2). The SPAdes assembly had the highest fungal completeness in MEGAN-assigned assemblies and thus corroborated the QUAST results. For MetaWatt-assigned assemblies, MetaSPAdes had a slightly higher fungal completeness compared to SPAdes, but, based on QUAST, SPAdes had overall better genome statistics and higher overlap to the reference genome. MEGAN generally assigned a higher number of contigs to Ascomycota compared to MetaWatt, although genome completeness did not deviate greatly between the two methods. We found a large core overlap of contigs assigned to Ascomycota by both taxonomic assignment methods (Supplementary Table S3). Overall, genome statistics, overlap to reference and fungal genome completeness showed the same trends in both examined species. For downstream analyses, we selected SPAdes assemblies assigned in MEGAN and MetaWatt for both species as best-possible reconstructed mycobiont genomes from metagenomic sets. These assemblies showed similar completeness as the reference genomes, but were, as expected, more fragmented as indicated by the higher number of scaffolds and the lower N50 (Table 3). SPAdes assemblies of *E. prunastri* had an average coverage of ~110x (MEGAN) and ~105x (MetaWatt), while SPAdes assemblies of *P. furfuracea* had an average coverage of ~135x (MEGAN) and ~160x (MetaWatt).

**Table 3:** Fungal genome assemblies and gene sets from metagenomic lichen thalli assembled with SPAdes.

	*Evernia prunastri*		*Pseudevernia furfuracea*	
	metagenome MG	metagenome MW	metagenome MG	metagenome MW
Number of scaffolds	1,775	1,624	3,558	1,829
Total size	39 Mb	37 Mb	43 Mb	33 Mb
N50	53,038	54,988	36,386	48,187
Average coverage	~110x	~105x	~135x	~160x
Number of genes	11,098	10,713	10,028	8,962
Genome completeness	C: 95.4%	C: 93.2%	C: 93.7%	C: 91.3%
Gene set completeness	C: 91.7%	C: 89.2%	C: 91.3%	C: 89.4%

Taxonomic assignment of metagenomic reads to Ascomycota was performed with MEGAN (MG) and MetaWatt (MW). Completeness is compared against 1,315 orthologous BUSCO marker genes for Ascomycota (C: complete BUSCO genes found).

### Comparison of gene sets

After quality trimming, we obtained 28,755,102 (87.9%) paired-end RNA reads of *E. prunastri* of which 53.9% aligned to the MEGAN- and 51.3% aligned to the MetaWatt-assigned metagenome to give hints for gene prediction. For *P. furfuracea* 16,649,331 (90.9%) paired-end RNA reads remained after quality filtering and 58.6% of these mapped against the MEGAN-assigned metagenome assembly, and 53.2% mapped against the MetaWatt-assigned assembly. We predicted 11,098 genes from the MEGAN-assigned assembly and 10,713 genes from the MetaWatt-assigned assembly for *E. prunastri*. For *P. furfuracea* we predicted 10,028 genes from the MEGAN-assigned assembly and 8,962 genes from the MetaWatt-assigned assembly. These four gene sets had completeness between 89.2% and 91.7% (Table 3) which is comparable to the reference gene sets.

**Figure 3:**
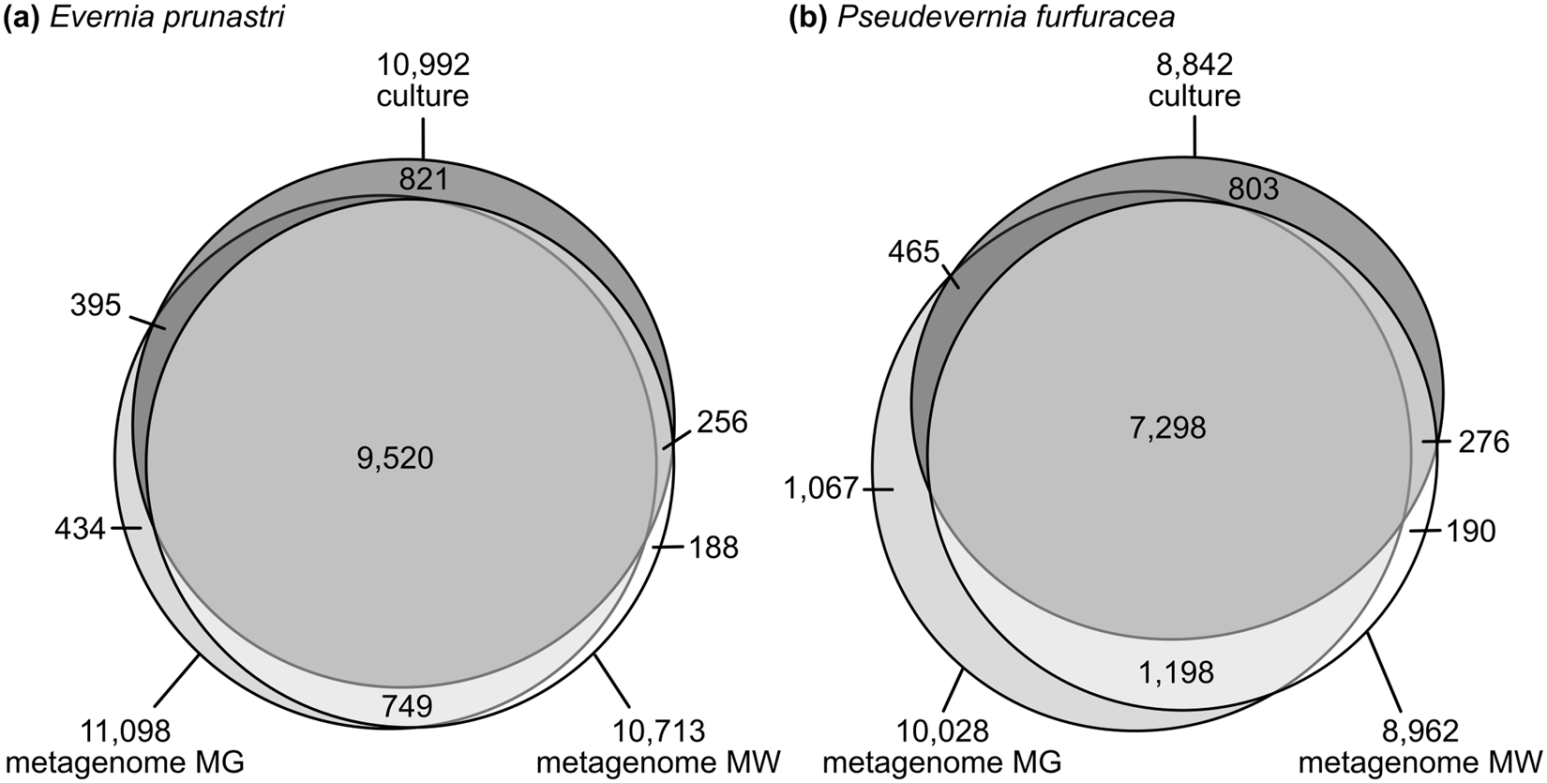
Orthologous gene sets in assemblies from fungal culture and from natural lichen sample. Analyses are based on Reciprocal Best Hits. Taxonomic assignment of metagenomic reads to Ascomycota was performed with MEGAN (MG) and MetaWatt (MW).

We identified orthologous pairs (RBH) among the different gene sets of each species. The reference genes overlapped to a large extent with the genes from the MEGAN- and the MetaWatt-assigned metagenome assemblies with a core overlap between the three of 87% for *E. prunastri* and 83% for *P. furfuracea* (Fig. 3). The MEGAN set covered 90.2% and MetaWatt covered 88.9% of the pure fungal culture genes in *E. prunastri*. In *P. furfuracea* the MEGAN set covered 87.8% and MetaWatt covered 85.6% of the pure fungal culture genes. The metagenomic-based gene sets overlapped with 10,269 genes in *E. prunastri* and 8,496 genes in *P. furfuracea*.

We identified a high number of reducing and non-reducing polyketide synthases, non-ribosomal peptide synthetases and terpene synthases with antiSMASH in both species. In total, we found 50 biosynthetic genes in the *E. prunastri* culture-based reference genome and 51 and 49 biosynthetic genes in the MEGAN- and MetaWatt-assigned metagenome assemblies. In *P. furfuracea* we found 31 biosynthetic genes in the reference and 31 and 27 biosynthetic genes in the MEGAN- and MetaWatt-assigned assemblies, respectively. We observed a high overlap of genes between biosynthetic genes found in culture and in metagenomic samples. In *E. prunastri* 82% of the biosynthetic genes in the culture (41 of 50) were also present in the metagenome, while we found 71% (22 of 31) of the biosynthetic genes in the metagenome of *P. furfuracea* (Table 4).

**Table 4:** Biosynthetic genes identified in cultures, the MEGAN-assigned metagenomes (MG) and the MetaWatt-assigned metagenomes (MW).

	*Evernia prunastri*				*Pseudevernia furfuracea*			
	culture	metagenome MG	metagenome MW	common	culture	metagenome MG	metagenome MW	common
Reducing type I PKS	20	20	20	19	12	9	7	6
Non-reducing type I PKS	8	8	7	6	5	6	6	5
Type III PKS	2	2	2	2	2	2	2	2
Hybrid PKS-NRPS	4	3	3	3	3	3	3	2
NRPS	4	4	5	3	4	5	4	3
Terpene synthases	12	14	12	8	5	6	5	4
Total	50	51	49	41	31	31	27	22

The metagenome derived assemblies were taxonomically assigned to Ascomycota. PKS (Polyketide synthases), NRPS (Non-ribosomal peptide synthetases). Type III PKSs refer to chalcone synthases.

### Taxonomic composition of metagenomic reads

**Figure 4:**
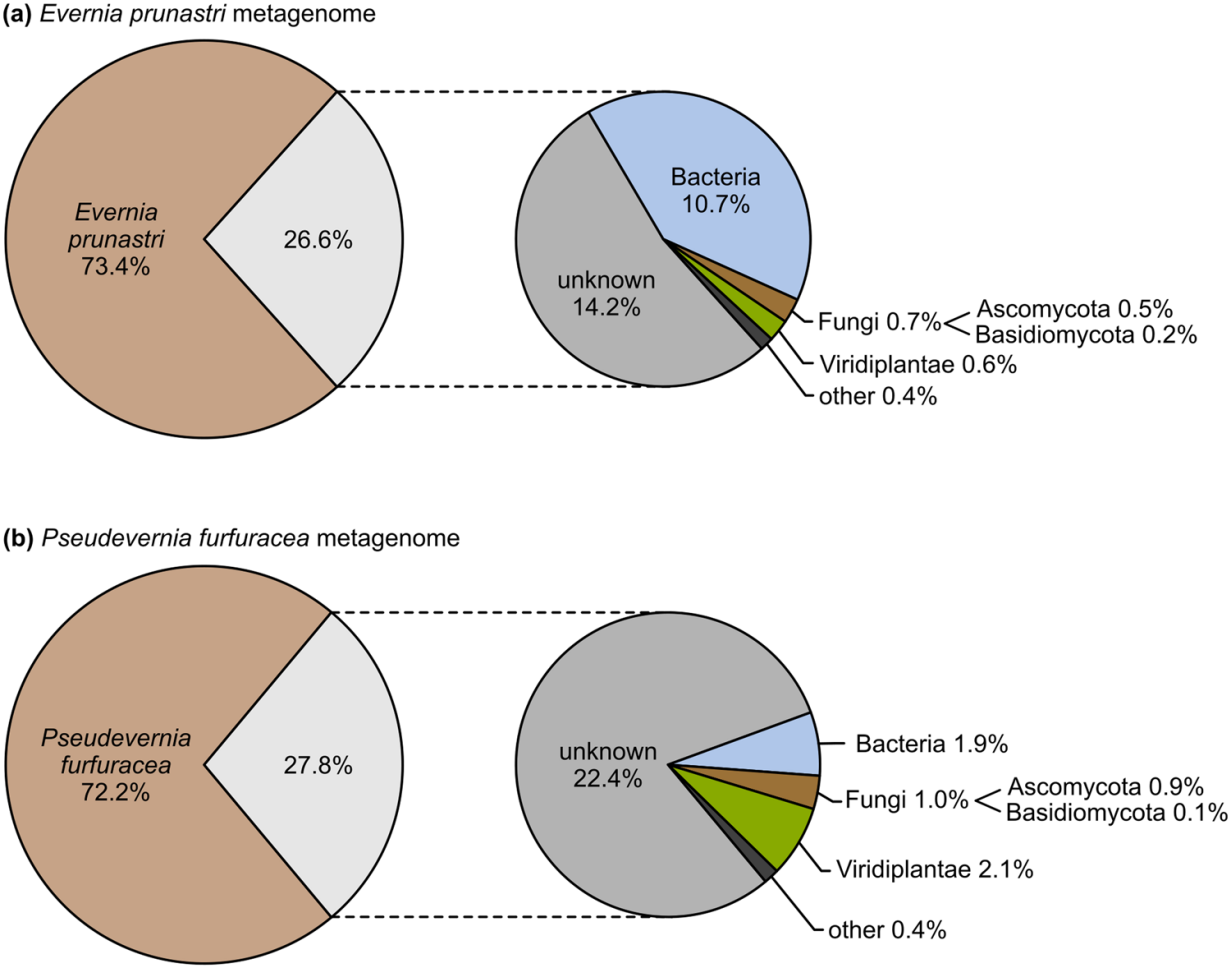
Taxonomic classification and abundance estimation of the quality filtered metagenomic reads from lichen thalli. For the pie chart on the left the reads were assigned using Kraken against a custom database of fungi to assess how many reads belong to the reference lichen-forming fungus. For the pie chart on the right, we classified all reads that did not belong to the lichen-forming fungus using BLAST/MEGAN with the NCBI *nr* protein database. The ‘unknown’ proportion refers to reads without a BLAST hit or reads that could not be assigned in MEGAN. Detailed read numbers can be found in Supplementary Table S4.

The taxonomic classification and abundance estimation of quality-filtered metagenomic reads is shown in Fig. 4. Kraken estimated 13,850,095 (73.4%) metagenomic reads to belong to *E. prunastri* and 14,178,239 (72.2%) to *P. furfuracea*. A further classification of the 26–27% of reads that were not classified as reference mycobionts with BLAST/MEGAN against the entire GenBank *nr* protein database is also shown in Fig. 4. In the metagenome of *E. prunastri* we found 10.7% Bacteria, 0.7% Fungi (0.5% Ascomycota, 0.2% Basidiomycota) and 0.6% Viridiplantae. In *P. furfuracea* we found 1.9% Bacteria, 1% Fungi (0.9% Ascomycota, 0.1% Basidiomycota) and 2.1% Viridiplantae. A large proportion of the reads (14.2% for *E. prunastri* and 22.4% for *P. furfuracea*) were of unknown origin, i.e. without a BLAST hit or not assignable in MEGAN. Detailed read numbers of the classification with MEGAN are given in the Supplementary Table S4.

The search for biosynthetic genes in the assembled proportion of non-target Ascomycota resulted in one non-ribosomal peptide synthetase in the *E. prunastri* metagenome and one hybrid polyketide non-ribosomal peptide synthetase in the metagenome of *P. furfuracea*.

### Data availability

This Whole Genome Shotgun project has been deposited at DDBJ/ENA/GenBank under the accessions NKYR00000000 (*Evernia prunastri* mycobiont culture) and NKYQ00000000 (*Pseudevernia furfuracea* mycobiont culture). The metagenomic sequence data and metatranscriptomes are available under the accession number SRP111200. The metagenomic assemblies are available at figshare (https://doi.org/10.6084/m9.figshare.5531692).

## Discussion

We evaluated the applicability of the metagenome skimming approach to assemble genomes of lichen-forming fungi by comparing genomes assembled from mixed DNA samples (natural thalli) to genomes assembled from DNA from pure fungal cultures. The metagenome-derived fungal assemblies are comparable to the reference genomes from pure culture in terms of total genome size and genome completeness. While the metagenome-derived genomes from a single shotgun sequencing library are naturally more fragmented than the respective references that were sequenced at a deeper coverage and with different sequencing libraries, they still cover most of the reference gene space (estimated 86–90%).

### Comparison of metagenomic assembly strategies

We observed extensive differences in assembly quality among assemblers. To some extent this might be due to the presence of highly uneven read coverages. Different species in a metagenomic sample have different abundances resulting in a highly non-uniform read coverage across different genomes. Furthermore, the coverage of most species from a metagenomic sample is much lower than in a typical sequencing project of a cultivated sample^7,9^. Lichen thalli represent multi-species communities of fungal, algal and bacterial species^14^ that were shown to have uneven coverages and therefore pose challenges for assemblers^29^. The best performing assemblers, SPAdes, MetaSPAdes and IDBA-UD, were to some extent especially designed for sequencing data with highly uneven coverage^9,10,41^, while in contrast omega and MetaVelvet are surprisingly negatively affected by such data^29^. The latter two employ coverage information to distinguish between species and therefore might have difficulties in assembling the fungal genome from uneven coverage short-read sequencing data^84^.

Our study was designed to compare metagenomic assemblies against pure fungal reference genomes. Our results suggest that a reference is not necessarily needed to choose the best assembler, as assembly statistics and fungal genome completeness alone would have led to the selection of the same assembler. SPAdes, or its metagenomic variant MetaSPAdes, seem to be among the most reliable (and fastest) tools for assembling metagenomic reads in the presence of highly uneven read coverage.

### Comparison of metagenomic binning approaches

The choice of taxonomic binning method (MEGAN and MetaWatt) made no large difference, but MEGAN performed slightly better in terms of mycobiont completeness. On the one hand, the BLAST/MEGAN approach is commonly used^29,85,86^, and constant development and added functionality (e.g., functional profiling and direct comparison of several samples) have improved MEGAN over time^68,69,87^. MEGAN relies on an initial BLAST run against the NCBI *nr* database which requires considerable disk space for the database and computational power for the DIAMOND search. On the other hand, MetaWatt requires considerably less time for a similarly good taxonomic assignment as it can already be efficiently used with a smaller database of genomes by deploying ﻿multivariate statistics of tetranucleotide frequencies and differential coverage based binning rather than sequence similarity.

### Comparison of gene space

We were able to cover up to 88–90% of the respective reference gene spaces. Furthermore, we showed that metagenome-derived assemblies reliably recovered almost all members of the diverse gene families involved in secondary metabolism. We showed a high diversity of secondary metabolite genes in *E. prunastri* and *P. furfuracea* as expected from the high number of substances that have been reported from extracts of these species^31,32^. The high overlap of these biosynthetic genes in the metagenomes compared to their reference suggests a great potential of the metagenome skimming approach for natural product discovery in lichens^88^. Beside mining the genomes for secondary metabolites that produce interesting lichen compounds, the potential of whole genome mining can be extended to other target gene families, e.g. mating-type genes, symbiosis-related proteins or secreted effector proteins^89^.

A few secondary metabolism-related genes were exclusively found in the metagenomes. These genes may result either from the annotation of more fragmented assemblies or, as shown in two cases, from contamination, e.g. from other non-lichen-forming ascomycetes (i.e., lichenicolous fungi). By using the fungal genomes obtained from aposymbiotic cultures, we were able to access the taxonomic composition of reads used for the metagenomic assemblies. Interestingly, nearly three-quarters of our metagenomic reads represent the lichen-forming fungus while other ascomycetes constituted less than 0.9% of the total reads. This suggests that only a minor fraction of the additional fungal gene models found in the metagenomes may represent contamination from other fungi that could not be filtered out during taxonomic assignment due to incompleteness of the reference database.

One of the essential steps in reconstructing genomes from metagenomic samples is the taxonomic assignment of sequences^90^. Sensitivity and precision of assignment methods depend on the availability and quality of reference databases and taxonomies^91,92^. Taxonomic binning of metagenomic sequences will improve in the future when more genomes become available in public databases and the development of sequence composition-based methods in comparison to similarity-based methods will progress further. Until then, without good reference databases it is not possible to entirely exclude contamination in metagenomic assemblies or target specific genomes by filtering out unwanted sequences.

### Additional applications and conclusions

Our results show that metagenome skimming constitutes a comprehensive genome-mining tool for lichens, and potentially for other microbial symbioses. Fungal genomes reconstructed from metagenomic read sets can be used in comparative phylogenomics, an approach to link genomic features to traits in a phylogenetic context^93^. A recent study showed that phylogenomic datasets can be useful to resolve evolutionary relationships among cryptic lichen-forming fungal lineages^94^. In this context, metagenome skimming can drastically extend the traditional set of DNA barcodes on which to infer phylogenetic relationships, while circumventing the necessity for a reference genome from culture^95,96^. This is especially important for filling gaps in the tree of life as some species rich-clades in the Ascomycota consist entirely of lichenized fungi (Lecanoromycetes, Lichinomycetes, and a large subset of Eurotiomycetes). Additionally, it will help to better understand the impact of lichenization on the evolution of fungal genomes.

Another significant potential of metagenome skimming lies in the development of high-resolution population genetic markers. A sensitive fingerprinting technique is especially important in highly clonal organism such as lichens^97^. Here, microsatellites can be used to investigate microevolutionary processes at the level of populations to study association patterns or reconstruct gene flow and symbiont transmission^86,98,99^. In recent years, an increasing number of studies have used genome skimming short read libraries for marker isolation, in particular fungus-specific microsatellites (SSRs). Metagenome skimming is in fact cheaper and more informative than the library microsatellite enrichment approach, as it retrieves all kinds of repeats (microsatellites, minisatellites, and potentially also transposable elements). Furthermore, here we have shown that the metagenome skimming approach covers most of the gene space of the fungal symbiont.

In conclusion, our findings suggest that metagenome skimming is a viable tool to reconstruct nearly complete fungal genomes from uncultured lichen thalli. Additionally, algal and bacterial contigs can be accessed via taxonomic binning and functional annotation to explore biodiversity and natural compound diversity^23,88^. Metagenome skimming circumvents the time-consuming step of cultivation and can be applied to unculturable organisms, symbiotic consortia, and other complex communities. It therefore bears a great potential to be applied in several research fields that are rapidly changing our view on the ecology and evolution of symbiotic associations.

## Electronic supplementary material

Supplementary Information

Supplementary_S6-S7
