# Supplementary material for: Sequencing genomes from mixed DNA samples - evaluating the metagenome skimming approach in lichenized fungi
Source: Sci Rep. 2017 Nov 2;7:14881. doi: 10.1038/s41598-017-14576-6 (PMC5668418; doi:10.1038/s41598-017-14576-6)
Supplement: Supplementary file 1 — Supplementary Information [file 41598_2017_14576_MOESM1_ESM.pdf]

Supplementary Information to

Sequencing genomes from mixed DNA samples - evaluating the metagenome skimming approach in lichenized fungi

Anjuli Meiser[1,2], Jürgen Otte[2], Imke Schmitt[1,2*], Francesco Dal Grande[2*]

[1] Institute of Ecology, Evolution and Diversity, Goethe University Frankfurt, Max-von-Laue Str. 13, D-60438 Frankfurt, Germany
[2] Senckenberg Biodiversity and Climate Research Centre (SBiK-F), Senckenberganlage 25, D-60486 Frankfurt, Germany

[*] F.D.G. (francesco.dalgrande@senckenberg.de), I.S. (imke.schmitt@senckenberg.de)

Table S1. Identification of tetranucleotide bins that correspond to the lichen-forming fungus with MetaWatt.

Table S2. BUSCO genome completeness of all fungal genomes assembled from metagenomic source and assigned to Ascomycota.

Table S3. Number of contigs of assemblies based on metagenomic reads and taxonomic assignment of metagenomic reads to Ascomycota with MEGAN and MetaWatt.

Table S4. Taxonomic assignment of quality filtered metagenomic reads from lichen thalli that were not assigned as the reference lichen-forming fungus.

Table S5. Taxonomic assignment of scaffolds assembled with SPAdes from metagenomic reads from lichen thalli.

Table S1. Identification of tetranucleotide bins that correspond to the lichen-forming fungus with MetaWatt. Bins that had an Ascomycota profile with at least 50% of the fragments classified as Ascomycota and no other taxa represented in their taxonomic profile were selected. All bins that met these criteria were merged and contigs that were not classified as Ascomycota or 'Unknown' were unbinned manually. Statistics presented here refer to the combined bins for each assembly and species.

Species		SPAdes	metaSPAdes	IDBA-UD	MetaVelvet	omega	mira
Evernia prunastri	Combined bins	3	4	3	4	5	16
	Removed contigs	68	29	58	34	72	814
	All contigs	1,624	1,602	2,757	2,432	7,577	39,338
	Ascomycota contigs	995 (61%)	1,262 (79%)	1,417 (51%)	2,017 (83%)	4,789 (63%)	15,744 (40%)
	'Unknown' contigs	629	340	1340	415	2,788	23,594
	Size	36.8 Mb	36.4 Mb	36.8 Mb	35.3 Mb	20.2 Mb	58.7 Mb
	N50	54,988	42,862	35,676	23,489	3,602	1,679
	GC	49.3-50.6%	49.4-50.7%	49.1-50.6%	49.2-50.6%	49.8-51.8%	48.8-51.5%
	tRNA's	24	26	29	22	7	78
	Coding density	54.3%	54.6%	53.9%	54.5%	60.4%	55.2%
	Coverage	106.9x	106.8x	105.4x	105.8x	105.6x	49.2x
	Profile completeness	52.4%	53.7%	51.8%	54.1%	34.5%	52.5%
	Profile copies	1.6x	1.6x	1.6x	1.6x	1.4x	2.1x
	Abundance	67.8%	67.8%	67.0%	75.3%	47.1%	50.6%
	Tetranucleotide	99.8%	99.8%	99.9%	99.8%	100%	100%
Pseudevernia furfuracea	Combined bins	2	2	2	4	4	4
	Removed contigs	33	69	58	117	85	125
	All Contigs	1,829	3,702	4,036	6,340	8,029	10,238
	Ascomycota contigs	1,216 (66%)	1,808 (49%)	2,195 (54%)	3,457 (55%)	4,995 (62%)	4,174 (41%)
	'Unknown' contigs	613	1,894	1,841	2,883	3,034	6,064
	Size	32.3 Mb	32.3 Mb	30.3 Mb	27.2 Mb	35 MB	28.6 Mb
	N50	48,031	30,899	17,504	8,549	7,180	6,760
	GC	48.9-50.2%	49.2-50.3%	48.8-50.2%	48.9-50.4%	48.8-50.5%	49.3-50.8%
	tRNA's	22	15	20	15	18	13
	Coding density	55.0%	56.6%	56.0%	58.0%	56.6%	58.3%
	Coverage	152.4x	153x	153.7x	152.4x	129.4x	110.8x
	Profile completeness	54.8%	54.5%	48.3%	51.5%	52.9%	50.0%
	Profile copies	1.6x	1.6x	1.6x	1.7x	1.8x	1.7x
	Abundance	65.0%	64.0%	59.2%	58.2%	70.5%	43.7%
	Tetranucleotide	99.9%	99.9%	100%	100%	100%	100%

Table S2. Genome completeness for genome assemblies from metagenomic lichen thalli using different assemblers. Taxonomic assignment of metagenomic reads to Ascomycota was performed with MEGAN (MG) and MetaWatt (MW). Percentage completeness is compared against 1,315 orthologous BUSCO marker genes for Ascomycota (C: complete [S: single-copy, D: duplicated], F: fragmented, M: missing).

(a) *Evernia prunastri*

	unassigned assembly	metagenome MG	metagenome MW
SPAdes	C:95.5% [S:94.1%, D:1.4%], F:2.7%, M:1.8%	C:95.4% [S:95.0%, D:0.4%], F:3.2%, M:1.4%	C:93.2% [S:92.9%, D:0.3%], F:3.1%, M:3.7%
metaSPAdes	C:95.5% [S:94.1%, D:1.4%], F:2.7%, M:1.8%	C:95.2% [S:94.8%, D:0.4%], F:3.2%, M:1.6%	C:93.7% [S:93.4%, D:0.3%], F:3.1%, M:3.2%
IDBA-UD	C:94.4% [S:93.2%, D:1.2%], F:3.0%, M:2.6%	C:93.8% [S:93.5%, D:0.3%], F:3.4%, M:2.8%	C:91.3% [S:91.0%, D:0.3%], F:3.2%, M:5.5%
metaVelvet	C:92.5% [S:92.0%, D:0.5%], F:4.9%, M:2.6%	C:92.1% [S:91.8%, D:0.3%], F:5.1%, M:2.8%	C:89.6% [S:89.3%, D:0.3%], F:4.4%, M:6.0%
omega	C:51.1% [S:50.7%, D:0.4%], F:33.5%, M:15.4%	C:49.2% [S:49.2%, D:0.0%], F:34.2%, M:16.6%	C:36.7% [S:36.7%, D:0.0%], F:24.8%, M:38.5%
mira	C:44.1% [S:41.1%, D:3.0%], F:40.0%, M:15.9%	C:41.9% [S:40.8%, D:1.1%], F:42.4%, M:15.7%	C:35.2% [S:34.5%, D:0.7%], F:38.2%, M:26.6%

(b) *Pseudevernia furfuracea*

	unassigned assembly	metagenome MG	metagenome MW
SPAdes	C:94.0% [S:92.2%, D:1.8%], F:3.7%, M:2.3%	C:93.7% [S:93.6%, D:0.1%], F:4.1%, M:2.2%	C:91.3% [S:91.2%, D:0.1%], F:3.9%, M:4.8%
metaSPAdes	C:93.6% [S:91.8%, D:1.8%], F:3.8%, M:2.6%	C:93.1% [S:92.9%, D:0.2%], F:4.3%, M:2.6%	C:91.8% [S:91.6%, D:0.2%], F:4.3%, M:3.9%
IDBA-UD	C:93.2% [S:91.6%, D:1.6%], F:3.7%, M:3.1%	C:93.0% [S:92.8%, D:0.2%], F:4.0%, M:3.0%	C:86.3% [S:86.3%, D:0.0%], F:3.1%, M:10.6%
metaVelvet	C:83.3% [S:81.6%, D:1.7%], F:10.6%, M:6.1%	C:82.2% [S:81.3%, D:0.9%], F:10.9%, M:6.9%	C:78.6% [S:77.9%, D:0.7%], F:7.9%, M:13.5%
omega	C:79.6% [S:73.6%, D:6.0%], F:15.4%, M:5.0%	C:79.2% [S:73.2%, D:6.0%], F:15.4%, M:5.4%	C:76.4% [S:71.1%, D:5.3%], F:14.2%, M:9.4%
mira	C:70.2% [S:66.2%, D:4.0%], F:22.6%, M:7.2%	C:69.8% [S:66.3%, D:3.5%], F:22.7%, M:7.5%	C:58.3% [S:56.4%, D:1.9%], F:17.9%, M:23.8%

Table S3. Number of contigs of assemblies based on metagenomic reads. Taxonomic assignment of metagenomic reads to Ascomycota was performed with MEGAN (MG) and MetaWatt (MW).

Species	metagenome assembly	SPAdes	metaSPAdes	IDBA-UD	MetaVelvet	omega	mira
Evernia prunastri	unfiltered and unassigned	51,428	67,447	98,046	16,779	34,042	148,894
	min 400 bp	49,746	61,771	95,085	14,558	32,679	130,534
	min 400 bp + Ascomycota MG	1,775	1,838	3,127	2,911	11,221	36,083
	min 400 bp + Ascomycota MW	1,624	1,602	2,757	2,432	7,577	39,338
	overlap (identical contigs in MG and MW)	1,026	1,275	1,527	2,076	5,647	21,409
Pseudevernia furfuracea	unfiltered and unassigned	62,248	101,711	145,738	226,304	26,661	143,739
	min 400 bp	60,165	90,678	144,848	126,609	26,130	113,825
	min 400 bp + Ascomycota MG	3,558	4,794	6,006	8,384	10,229	21,159
	min 400 bp + Ascomycota MW	1,829	3,702	4,036	6,340	8,029	10,238
	overlap (identical contigs in MG and MW)	1,300	2,190	2,516	3,997	5,838	6,831

Table S4. Taxonomic assignment of quality filtered metagenomic reads from lichen thalli that were not assigned as reference lichen-forming fungus (see Fig. 4 in the main text). These reads were classified using DIAMOND/MEGAN against the NCBI GenBank *nr* protein database. Only phyla above 0.1% are presented.

		Evernia prunastri		*Pseudevernia furfuracea*	
		7,880,997	26.6%	9,776,595	27.8%
No blast hits		4,176,471	14.1%	7,863,588	22.4%
Not assigned		16,915	0.1%	5,618	0.0%
Archaea		442	0.0%	482	0.0%
Bacteria		3,168,381	10.7%	659,705	1.9%
Eukaryota		457,096	1.5%	1,197,044	3.4%
kingdom	Fungi	213,931	0.7%	346,484	1.0%
	Metazoa	17,126	0.1%	27,789	0.1%
	Viridiplantae	189,160	0.6%	743,704	2.1%
phylum (> 0.1%)	Acidobacteria	415,805	1.4%	197,181	0.6%
	Actinobacteria	74,317	0.3%	30,936	0.1%
	Ascomycota	154,470	0.5%	302,340	0.9%
	Basidiomycota	49,305	0.2%	31,477	0.1%
	Chlorophyta	131,863	0.4%	573,746	1.6%
	Proteobacteria	2,445,225	8.3%	307,007	0.9%
	Streptophyta	38,053	0.1%	97,982	0.3%

Table S5. Taxonomic assignment of scaffolds assembled with SPAdes from metagenomic reads from lichen thalli. These scaffolds were classified using DIAMOND/MEGAN with the NCBI GenBank *nr* protein database. Only phyla above 0.1% are presented.

	Evernia prunastri				*Pseudevernia furfuracea*			
	number of scaffolds		length		number of scaffolds		length	
Total	49,746		126,470,248		60,165		186,849,783	
No blast hits	15,751	31.7%	19,909,906	15.7%	23,231	38.6%	31,266,115	16.7%
Not assigned	6,157	12.4%	9,350,115	7.4%	9,401	15.6%	20,532,506	11.0%
Eukaryota	8,420	16.9%	50,879,619	40.2%	20,748	34.5%	117,884,326	63.1%
Fungi	2,296	4.6%	39,589,067	31.3%	3,880	6.4%	43,657,327	23.4%
Ascomycota	1,775	3.6%	38,671,927	30.6%	3,558	5.9%	42,531,237	22.8%
Basidiomycota	407	0.8%	490,161	0.4%	193	0.3%	394,925	0.2%
Viridiplantae	5,101	10.3%	9,608,042	7.6%	15,683	26.1%	67,410,521	36.1%
Chlorophyta	3,333	6.7%	6,443,731	5.1%	13,013	21.6%	49,318,344	26.4%
Streptophyta	902	1.8%	1,623,011	1.3%	1,275	2.1%	8,306,445	4.4%
Metazoa	201	0.4%	365,771	0.3%	241	0.4%	1,439,461	0.8%
Arthropoda	25	0.1%	51,029	0.0%	32	0.1%	164,902	0.1%
Chordata	75	0.2%	147,033	0.1%	83	0.1%	547,521	0.3%
Bacteria	19,058	38.3%	45,660,763	36.1%	6,335	10.5%	14,666,264	7.8%
Acidobacteria	3,042	6.1%	10,455,677	8.3%	3,415	5.7%	6,662,926	3.6%
Actinobacteria	479	1.0%	494,543	0.4%	57	0.1%	114,791	0.1%
Bacteroidetes	35	0.1%	56,628	0.0%	21	0.0%	139,474	0.1%
Cyanobacteria	57	0.1%	105,054	0.1%	47	0.1%	222,491	0.1%
Firmicutes	50	0.1%	93,783	0.1%	45	0.1%	150,082	0.1%
Proteobacteria	14,109	28.4%	31,900,387	25.2%	2,072	3.4%	5,241,637	2.8%
Viruses	16	0.0%	22,036	0.0%	53	0.1%	299,184	0.2%
