# Supplementary material for: Sequencing genomes from mixed DNA samples - evaluating the metagenome skimming approach in lichenized fungi
Source: Sci Rep. 2017 Nov 2;7:14881. doi: 10.1038/s41598-017-14576-6 (PMC5668418; doi:10.1038/s41598-017-14576-6)
Supplement: Supplementary file 2 — Supplementary_S6-S7 [file 41598_2017_14576_MOESM2_ESM.zip › Supplementary_S6.html]

|  |
| --- |
| QUAST **Quality Assessment Tool for Genome Assemblies** by Center for Algorithmic Biotechnology |

Loading...

Aligned to
""
|
 bp
|
 % G+C
|
 chromosomes
  
 reads
|
 mapped
|
 properly paired
  
 genes
|
 operons

Unfortunately, JavaScript in your
browser is disabled or is not supported.
We need JavaScript to build report and plots.

Worst
Median
Best

Show heatmap

Combined reference size:
 bp

|  |  |
| --- | --- |
|  |  |
|  | |  |
| Contigs are ordered from largest (contig #1) to smallest.  Contigs are broken into nonoverlapping 100 bp windows. Plot shows numbers of windows for each GC percentage. |

{"minContig":400,"report":[["Statistics without reference",[{"values":[1775,1624,1838,1602,3127,2757,2911,2432,11221,7577,36083,39338],"quality":"Less is better","isMain":true,"metricName":"# contigs"},{"values":[1775,1624,1838,1602,3127,2757,2911,2432,11221,7577,36083,39338],"quality":"Less is better","isMain":false,"metricName":"# contigs (>= 0 bp)"},{"values":[1380,1087,1541,1373,1795,1517,2476,2231,8826,6182,23218,22802],"quality":"Less is better","isMain":false,"metricName":"# contigs (>= 1000 bp)"},{"values":[1048,918,1258,1179,1418,1311,1848,1793,1091,887,1188,1100],"quality":"Less is better","isMain":false,"metricName":"# contigs (>= 5000 bp)"},{"values":[864,797,982,956,1099,1064,1252,1257,50,46,246,229],"quality":"Less is better","isMain":false,"metricName":"# contigs (>= 10000 bp)"},{"values":[519,513,519,519,535,532,425,430,0,0,5,4],"quality":"Less is better","isMain":false,"metricName":"# contigs (>= 25000 bp)"},{"values":[236,237,188,187,161,160,69,69,0,0,0,0],"quality":"Less is better","isMain":false,"metricName":"# contigs (>= 50000 bp)"},{"values":[332411,332411,332073,332073,182161,182161,154093,154093,20600,20600,32673,29245],"quality":"More is better","isMain":true,"metricName":"Largest contig"},{"values":[38671927,36768376,37514446,36436585,38442682,36813103,36134292,35276741,27785829,20188275,58701573,58791663],"quality":"More is better","isMain":true,"metricName":"Total length"},{"values":[38671927,36768376,37514446,36436585,38442682,36813103,36134292,35276741,27785829,20188275,58701573,58791663],"quality":"More is better","isMain":false,"metricName":"Total length (>= 0 bp)"},{"values":[38392225,36374213,37301533,36270082,37704803,36137819,35866335,35147845,26061730,19168442,49151127,46848352],"quality":"More is better","isMain":true,"metricName":"Total length (>= 1000 bp)"},{"values":[37564797,35954752,36514797,35731730,36706730,35530956,34009050,33855867,7321437,6040380,9716605,8995054],"quality":"More is better","isMain":false,"metricName":"Total length (>= 5000 bp)"},{"values":[36227121,35084171,34505417,34099321,34342596,33642087,29651210,29844369,581810,543762,3416908,3176952],"quality":"More is better","isMain":true,"metricName":"Total length (>= 10000 bp)"},{"values":[30503098,30288520,26777359,26779769,24980918,24822160,16563581,16708961,0,0,140563,107890],"quality":"More is better","isMain":false,"metricName":"Total length (>= 25000 bp)"},{"values":[20379131,20398909,15097177,15036336,11891589,11830198,4488127,4488127,0,0,0,0],"quality":"More is better","isMain":true,"metricName":"Total length (>= 50000 bp)"},{"values":[53038,54988,41143,42862,34185,35676,22590,23489,3348,3602,1816,1680],"quality":"More is better","isMain":false,"metricName":"N50"},{"values":[28660,31230,22103,23744,19068,20253,12338,13254,2064,2236,1207,1103],"quality":"More is better","isMain":false,"metricName":"N75"},{"values":[216,199,269,256,339,317,489,469,2721,1854,8641,9403],"quality":"Less is better","isMain":false,"metricName":"L50"},{"values":[464,417,577,542,713,658,1024,964,5347,3629,18563,20178],"quality":"Less is better","isMain":false,"metricName":"L75"},{"values":["49.25","49.76","49.40","49.93","49.18","49.68","49.64","49.82","50.12","51.00","49.70","50.22"],"quality":"Equal","isMain":false,"metricName":"GC (%)"}]],["Misassemblies",[{"values":[486,388,245,193,603,432,381,332,609,180,91735,100293],"quality":"Less is better","isMain":true,"metricName":"# misassemblies"},{"values":[61,48,53,32,72,54,64,52,29,13,1088,1018],"quality":"Less is better","isMain":false,"metricName":" # relocations"},{"values":[422,338,190,160,526,374,316,279,575,166,88450,96747],"quality":"Less is better","isMain":false,"metricName":" # translocations"},{"values":[3,2,2,1,5,4,1,1,5,1,2197,2528],"quality":"Less is better","isMain":false,"metricName":" # inversions"},{"values":[368,302,201,174,471,355,306,277,571,175,26002,28399],"quality":"Less is better","isMain":false,"metricName":"# misassembled contigs"},{"values":[14182023,13546538,7265340,6903122,9741124,9057798,5410592,5333114,1170604,525849,44550234,44518244],"quality":"Less is better","isMain":true,"metricName":"Misassembled contigs length"},{"values":[470,402,391,323,465,410,422,368,229,118,770,524],"quality":"Less is better","isMain":false,"metricName":"# local misassemblies"}]],["Unaligned",[{"values":[326,529,269,203,434,880,119,126,295,186,396,418],"quality":"Less is better","isMain":false,"metricName":"# fully unaligned contigs"},{"values":[308096,422722,280476,207022,268966,503034,428984,431751,631598,400031,490565,436672],"quality":"Less is better","isMain":false,"metricName":"Fully unaligned length"},{"values":[362,228,375,311,866,656,290,262,307,152,4053,4020],"quality":"Less is better","isMain":false,"metricName":"# partially unaligned contigs"},{"values":[65,34,58,38,82,51,33,28,22,4,886,571],"quality":"Less is better","isMain":false,"metricName":" # with misassembly"},{"values":[92,62,93,74,114,90,80,69,72,39,558,333],"quality":"Less is better","isMain":false,"metricName":" # both parts are significant"},{"values":[1533548,998113,1283612,972096,1550562,1160015,675805,620937,300834,134229,1853089,1291672],"quality":"Less is better","isMain":false,"metricName":"Partially unaligned length"}]],["Mismatches",[{"values":[49684,46642,48019,46130,49226,46237,45458,43797,29617,21778,49417,50008],"quality":"Less is better","isMain":false,"metricName":"# mismatches"},{"values":[6099,5523,5788,5425,5713,5104,4851,4518,2864,2194,6922,6868],"quality":"Less is better","isMain":false,"metricName":"# indels"},{"values":[30577,27134,28597,26137,28644,24404,23541,21911,13568,10450,24428,22735],"quality":"Less is better","isMain":false,"metricName":"Indels length"},{"values":["137.47","134.13","135.08","132.24","137.62","133.80","131.64","129.67","113.62","112.50","159.66","158.95"],"quality":"Less is better","isMain":true,"metricName":"# mismatches per 100 kbp"},{"values":["16.87","15.88","16.28","15.55","15.97","14.77","14.05","13.38","10.99","11.33","22.36","21.83"],"quality":"Less is better","isMain":true,"metricName":"# indels per 100 kbp"},{"values":[4659,4245,4439,4194,4381,3969,3725,3472,2208,1698,6012,6018],"quality":"Less is better","isMain":false,"metricName":" # short indels"},{"values":[1440,1278,1349,1231,1332,1135,1126,1046,656,496,910,850],"quality":"Less is better","isMain":false,"metricName":" # long indels"},{"values":[2748,1001,3492,2249,0,0,520,500,2,1,67273,68318],"quality":"Less is better","isMain":false,"metricName":"# N's"},{"values":["7.11","2.72","9.31","6.17","0.00","0.00","1.44","1.42","0.01","0.00","114.60","116.20"],"quality":"Less is better","isMain":true,"metricName":"# N's per 100 kbp"}]],["Genome statistics",[{"values":["89.581","86.188","88.108","86.461","88.658","85.649","85.587","83.714","64.607","47.979","76.712","77.979"],"quality":"More is better","isMain":true,"metricName":"Genome fraction (%)"},{"values":["1.021","1.018","1.013","1.011","1.026","1.019","1.016","1.014","1.031","1.016","1.833","1.825"],"quality":"Less is better","isMain":true,"metricName":"Duplication ratio"},{"values":["9080 + 761 part","8912 + 719 part","8829 + 838 part","8944 + 802 part","8690 + 1075 part","8624 + 1013 part","8144 + 1366 part","8214 + 1305 part","3380 + 5175 part","2600 + 4296 part","2255 + 7555 part","2224 + 7655 part"],"quality":"More is better","isMain":true,"metricName":"# genes"},{"values":[309161,309161,308964,308964,181892,181892,154093,154093,20600,20600,17481,17481],"quality":"More is better","isMain":false,"metricName":"Largest alignment"},{"values":[50329,50329,38255,38377,32719,32301,19814,20143,2187,458,2544,2312],"quality":"More is better","isMain":false,"metricName":"NG50"},{"values":[25837,25355,18591,18446,17054,16283,9385,9545,null,null,1770,1639],"quality":"More is better","isMain":false,"metricName":"NG75"},{"values":[42671,44503,36156,38050,29363,31002,20756,21534,3207,3470,664,647],"quality":"More is better","isMain":false,"metricName":"NA50"},{"values":[21023,24519,18609,20211,14618,16517,10797,11632,1883,2088,168,167],"quality":"More is better","isMain":false,"metricName":"NA75"},{"values":[41024,41439,33333,33575,27851,28064,18274,18406,2008,null,1167,1120],"quality":"More is better","isMain":true,"metricName":"NGA50"},{"values":[18542,18392,14989,15293,12238,11985,7545,8005,null,null,622,608],"quality":"More is better","isMain":false,"metricName":"NGA75"},{"values":[232,233,304,305,367,369,588,585,5033,7543,4288,4640],"quality":"Less is better","isMain":false,"metricName":"LG50"},{"values":[510,512,682,683,792,803,1315,1300,null,null,9148,9923],"quality":"Less is better","isMain":false,"metricName":"LG75"},{"values":[267,244,304,289,389,362,531,510,2806,1893,19269,19947],"quality":"Less is better","isMain":false,"metricName":"LA50"},{"values":[582,516,662,617,846,766,1131,1063,5606,3752,68051,69858],"quality":"Less is better","isMain":false,"metricName":"LA75"},{"values":[287,286,345,344,423,422,640,637,5263,null,8660,8925],"quality":"Less is better","isMain":false,"metricName":"LGA50"},{"values":[645,643,788,783,953,954,1476,1456,null,null,20682,21325],"quality":"Less is better","isMain":false,"metricName":"LGA75"}]],["Predicted genes",[]],["Reference statistics",[{"values":[40346456,40346456,40346456,40346456,40346456,40346456,40346456,40346456,40346456,40346456,40346456,40346456],"quality":"Equal","isMain":false,"metricName":"Reference length"},{"values":["48.97","48.97","48.97","48.97","48.97","48.97","48.97","48.97","48.97","48.97","48.97","48.97"],"quality":"Equal","isMain":false,"metricName":"Reference GC (%)"},{"values":[10992,10992,10992,10992,10992,10992,10992,10992,10992,10992,10992,10992],"quality":"Equal","isMain":false,"metricName":"Reference genes"}]]],"referenceName":"Evernia\_prunastri\_reference","date":"13 September 2017, Wednesday, 15:49:25","order":[0,1,2,3,4,5,6,7,8,9,10,11],"assembliesNames":["SPAdes MEGAN","SPAdes MetaWatt","metaSPAdes MEGAN","metaSPAdes MetaWatt","IDBA-UD MEGAN","IDBA-UD MetaWatt","MetaVelvet MEGAN","MetaVelvet MetaWatt","omega MEGAN","omega MetaWatt","mira MEGAN","mira MetaWatt"]}

{{ qualities }}

{{ mainMetrics }}

{"lists\_of\_lengths":[[4411623,3051267,2536527,2200443,1938762,1764251,1576855,1446294,1352683,1246093,1171400,1116965,1046703,979925,907128,848924,795313,754668,710162,672095,623278,576695,543396,512445,486231,459337,432881,400319,368113,341506,321751,298445,274271,252984,232804,210972,195246,175539,151747,138137,127455,114672,101006,90700,75366,64891,58014,51362,44672,40443,37316,32574,27562,24914,22884,21748,20938,20023,19150,18569,18044,17634,17313,16890,16618,16389,15232,12501,2869],[4411623,3051267,2532467,2188932,1929592,1759383,1569232,1449351,1356329,1249917,1172262,1117901,1047850,976993,904244,842880,788906,748904,701104,662336,604558,560156,525004,490456,461033,432515,393960,354842,328147,295380,263775,231456,201747,167814,141565,126636,105873,79838,55462,39298,31066,27124,24980,23554,22688,21808,20956,20443,19943,19568,19190,18804,18433,18031,17744,17441,17187,16992,16810,16578,16386,16058,5604],[3874790,2578227,2110254,1787630,1605668,1470757,1368027,1278730,1210942,1136619,1059444,1004661,939511,884269,847070,804718,762490,723269,690812,664331,630795,596710,565908,539503,518743,497249,479499,460216,440423,423253,401241,370074,350308,333900,314929,297793,278922,263487,244422,229116,218139,205790,195063,186061,172650,160520,149828,137461,128123,116003,103099,91825,81907,73371,64918,55478,46321,37807,31191,25637,22796,21207,20268,19270,18467,17724,17223,16792,16458,15781,8558],[3874790,2578227,2110254,1787630,1603643,1466287,1364239,1276056,1208158,1135121,1062185,1009517,944534,888018,848727,806279,762552,723269,690812,664331,630487,595002,563527,535731,514640,492371,472936,451968,433660,414049,384191,357060,339818,320155,301927,282582,264192,242867,227099,213528,197676,184492,168287,153586,139449,126163,107791,92692,79350,66166,51205,37581,28153,23631,21443,20114,19057,18235,17430,16861,16348,8456],[3131642,2203053,1826493,1647000,1461470,1369145,1271842,1184903,1134933,1073166,1017491,968374,917340,870985,837441,796513,764349,736679,705915,681137,653340,621398,595898,569849,545969,529229,513750,495260,476223,459746,442875,427015,407997,387398,371504,352512,337320,322263,311274,298779,283002,269739,257828,246848,235679,221922,211265,200943,191462,179762,168706,158766,148976,140709,131206,123287,113701,105828,95890,84880,74466,64538,56842,48680,42244,37135,32838,29830,27352,25203,23456,22035,20968,20258,19354,18619,17996,17582,17139,16774,16473,16151,15849,15573,15255,14984,14766,14544,14396,14254,14024,13781,13557,13296,13048,12869,12641,12488,12330,12215,12057,11986,11986,11986,11986,11986,11986,11935,11882,11815,11734,11642,11589,11540,11490,11456,11378,11304,11244,11160,2868],[3131642,2203053,1826493,1645031,1455948,1366314,1267876,1183559,1131502,1069146,1013427,964042,909352,863399,829123,790002,758739,731005,700699,677689,648480,613717,586173,559300,537400,521855,501895,483431,463965,447642,431214,412135,392206,374778,355828,339182,322686,309693,296621,280287,265550,253480,243058,230553,218635,205731,187804,171072,157501,144010,131270,119214,105102,87559,71116,61836,45532,32825,25715,22710,21196,20027,19160,18213,17611,17160,16658,16221,15853,15599,15358,15105,14861,14662,14486,14280,14097,13933,13787,13599,13425,13300,13183,13075,12926,12767,12643,12513,12396,12251,12110,12004,11974,11914,11845,11730,11643,11578,11520,11469,11424,11376,11312,11268,11220,11160,409],[2117489,1495296,1318934,1211610,1120467,1045629,982910,918031,881036,846559,812771,775667,741455,711153,688722,669000,645400,617347,594223,573751,555027,536512,517225,502712,489463,480106,465823,453444,444237,433106,420995,406906,390295,379952,366842,356972,347488,336845,327832,320300,313080,307739,300280,293091,285213,277505,270496,264147,257206,250059,244504,238173,232130,224441,218287,213155,204713,197703,190572,186431,180932,176835,171751,166579,161683,156566,152791,148389,142879,138847,133322,127994,123510,118993,114460,110809,106948,103937,99198,94353,90741,84326,79719,75009,70686,65036,60890,56337,52037,47720,43357,39327,35242,32242,28404,25323,22744,20720,19456,18757,17788,16667,15991,15312,14803,14257,13617,12999,12321,11775,11164,10292],[2117489,1495296,1318934,1211610,1120467,1045629,982910,918031,881193,847757,815251,778822,744656,714238,691382,671665,650574,622704,599713,579223,560866,543179,523937,508216,493564,482925,470759,457650,448183,438325,427330,413024,398061,383388,371818,359593,351402,340099,330457,322251,315265,309366,301567,294398,286021,278009,271571,265126,258688,251312,246214,240378,234249,228250,221123,216087,209858,201577,193180,188262,182136,175333,169804,163298,156638,151767,145551,139726,133541,127038,121343,114696,108755,102540,95421,90018,82868,75854,68049,61015,55321,48780,42872,37529,32311,27505,23773,20543,18285,16489,14808,12772,11507,5783],[329388,272396,250811,235204,225613,220046,213572,207241,201598,196676,193178,189045,185519,182308,178622,176116,173483,171214,168519,166050,164034,161000,158770,156641,154460,152194,150629,149265,147696,145953,144370,142976,141808,140697,139651,138151,136767,135600,134053,132945,131761,130414,129399,128365,127320,126307,125178,124039,123030,121836,120613,119696,118869,117888,116828,115841,114979,114045,113319,112643,111873,111241,110370,109736,109082,108418,107743,107136,106406,105752,105013,104379,103782,103089,102556,102084,101556,101066,100361,99869,99241,98485,97899,97433,96802,96214,95626,95115,94727,94075,93575,93047,92391,91872,91300,90809,90363,89963,89503,89048,88557,88180,87805,87392,87075,86710,86348,85917,85341,84946,84546,84145,83689,83261,82822,82419,82081,81643,81253,80889,80558,80095,79734,79383,78903,78486,78125,77775,77377,77059,76651,76280,75967,75621,75180,74837,74549,74178,73752,73403,73053,72724,72385,71975,71625,71368,71017,70682,70178,69740,69341,69034,68681,68317,68005,67692,67319,66968,66703,66391,66142,65886,65565,65215,64912,64571,64276,63893,63580,63369,63100,62794,62485,62186,61869,61593,61321,61080,60868,60597,60369,60047,59764,59478,59188,58946,58732,58500,58289,58002,57724,57455,57205,56897,56598,56366,56118,55787,55494,55253,54983,54701,54471,54258,54051,53709,53377,53127,52871,52562,52328,52115,51813,51639,51398,51170,50898,50647,50412,50169,49959,49741,49484,49223,49008,48781,48522,48290,48067,47816,47591,47392,47164,46999,46823,46567,46319,46041,45874,45658,45434,45221,45008,44786,44561,44376,44117,43896,43710,43474,43239,43083,42865,42669,42443,42227,42050,41853,41655,41472,41263,41076,40874,40669,40445,40228,40044,39794,39596,39440,39226,38972,38733,38537,38383,38197,37958,37743,37536,37267,37046,36845,36637,36432,36188,36016,35867,35678,35488,35230,35016,34841,34635,34444,34264,34078,33892,33689,33503,33314,33134,32943,32765,32563,32350,32091,31863,31675,31526,31382,31226,31031,30853,30663,30460,30268,30078,29858,29726,29525,29338,29146,28992,28787,28603,28419,28254,28117,27923,27729,27592,27458,27276,27096,26871,26662,26479,26319,26158,25988,25829,25689,25516,25320,25120,24984,24840,24694,24503,24368,24260,24137,23957,23759,23579,23386,23229,23052,22875,22748,22607,22439,22251,22103,21922,21751,21557,21374,21259,21129,20973,20819,20617,20481,20352,20182,20028,19878,19716,19508,19327,19139,18964,18778,18585,18354,18212,18088,17958,17813,17666,17501,17335,17136,17019,16876,16743,16608,16467,16358,16214,16075,15909,15717,15567,15461,15361,15253,15127,15021,14890,14772,14682,14606,14510,14414,14307,14205,14072,13995,13896,13770,13645,13478,13347,13157,12867,12621,12286,11833,11202,6150],[333730,269733,247587,230649,222227,215560,208290,201810,196401,192573,188243,184430,180315,176351,173185,169885,166330,163499,160130,157769,154255,151625,150082,148163,145883,143853,142146,140707,139243,137384,135922,134102,132571,130741,129466,128059,126778,125384,123922,122520,120963,119554,118176,116844,115598,114512,113392,112201,111236,110297,109553,108732,107953,107264,106387,105491,104620,103787,102891,102215,101507,100614,99881,99155,98370,97762,96988,96202,95451,94849,94194,93497,92709,91774,90984,90300,89725,89122,88474,87818,87312,86886,86316,85628,84974,84295,83615,82915,82385,81914,81307,80802,80195,79701,79145,78465,77877,77302,76751,76288,75852,75157,74707,74206,73663,73201,72666,72092,71608,71162,70623,69899,69393,68912,68429,68089,67663,67059,66572,66108,65743,65290,64918,64477,63870,63413,63092,62633,62134,61675,61226,60908,60507,60107,59740,59447,59110,58782,58476,58165,57764,57294,56776,56395,56018,55595,55176,54768,54392,54026,53650,53259,52874,52524,52275,51921,51594,51181,50917,50572,50157,49855,49465,49033,48635,48225,47861,47483,47089,46817,46510,46110,45790,45473,45214,44952,44615,44212,43899,43630,43266,43003,42665,42294,41979,41660,41335,41047,40715,40380,40070,39657,39419,39087,38651,38363,38058,37692,37382,37085,36780,36459,36123,35870,35635,35278,34920,34552,34213,33870,33599,33292,33026,32769,32545,32261,31921,31658,31343,31009,30770,30488,30205,29868,29607,29370,29120,28884,28607,28362,28098,27847,27567,27252,26985,26667,26345,26072,25837,25600,25250,24961,24700,24472,24270,24014,23692,23447,23199,22958,22722,22510,22237,21923,21695,21458,21189,20909,20666,20426,20225,20028,19724,19341,18969,18701,18381,18114,17854,17593,17259,17000,16783,16615,16430,16205,15961,15630,15399,15154,14986,14715,14509,14299,14065,13804,13478,13098,12771,12352,11641,4623],[596934,447665,391004,360346,335046,314425,296094,282445,271087,260212,249960,240282,231629,224398,217504,211109,206381,202194,198486,194502,189375,185496,182006,177435,174155,170789,168051,164955,162448,160307,158117,155952,153848,151619,149554,147383,145575,143747,141551,139431,137549,136192,134679,132859,131598,130093,128460,127329,125971,124427,123134,121567,120357,119240,118042,117143,115799,114531,113311,112447,111299,110486,109685,108646,107838,107057,106469,105833,105034,104263,103449,102627,101839,101070,100278,99529,98655,97855,97246,96606,96138,95499,94803,94355,93803,93115,92453,91987,91374,90866,90322,89896,89383,88840,88318,87772,87318,86860,86395,86104,85757,85265,84802,84324,83916,83390,82957,82554,82133,81758,81304,80970,80635,80243,79980,79549,79182,78770,78492,78209,77830,77497,77192,76753,76348,76025,75650,75400,75114,74838,74582,74324,74065,73790,73494,73114,72787,72480,72221,71907,71671,71366,71126,70843,70586,70374,70130,69845,69629,69391,69162,68975,68757,68557,68303,68042,67775,67546,67328,67106,66911,66727,66545,66354,66202,66018,65809,65571,65372,65197,65022,64810,64603,64362,64211,64048,63893,63691,63563,63389,63162,62997,62849,62705,62541,62406,62257,62077,61893,61736,61585,61386,61202,61023,60859,60722,60591,60459,60320,60158,59994,59864,59724,59566,59416,59276,59112,58972,58851,58718,58571,58406,58271,58145,57990,57861,57738,57622,57414,57259,57139,56989,56859,56757,56638,56494,56350,56256,56143,56033,55929,55813,55711,55582,55461,55353,55251,55124,55020,54892,54810,54707,54616,54509,54411,54315,54211,54123,53975,53866,53770,53676,53579,53488,53392,53289,53183,53084,52985,52885,52762,52681,52588,52486,52379,52260,52146,52043,51951,51858,51757,51674,51569,51491,51406,51317,51225,51150,51082,50999,50922,50865,50784,50734,50677,50592,50499,50432,50375,50289,50207,50123,50044,49968,49890,49794,49720,49616,49527,49459,49368,49294,49202,49125,49040,48947,48862,48789,48728,48650,48586,48524,48460,48378,48314,48255,48209,48146,48083,48026,47940,47873,47801,47719,47657,47608,47545,47495,47448,47374,47315,47258,47203,47154,47084,47009,46950,46873,46816,46754,46698,46627,46580,46523,46467,46409,46342,46279,46208,46134,46074,46026,45973,45927,45868,45822,45770,45721,45651,45585,45510,45445,45389,45341,45286,45239,45164,45103,45052,44973,44917,44863,44800,44747,44694,44650,44591,44536,44488,44437,44395,44347,44290,44232,44178,44117,44057,43994,43945,43884,43837,43790,43744,43682,43636,43586,43534,43473,43421,43377,43323,43277,43219,43173,43131,43081,43021,42982,42941,42895,42842,42794,42746,42695,42656,42598,42539,42495,42463,42423,42382,42343,42306,42261,42205,42159,42114,42047,42001,41960,41915,41873,41829,41791,41745,41711,41676,41632,41588,41542,41502,41453,41399,41349,41313,41269,41230,41194,41152,41116,41075,41024,40969,40912,40870,40829,40790,40745,40686,40649,40601,40559,40507,40454,40404,40363,40312,40277,40224,40182,40150,40108,40059,40029,40000,39953,39921,39891,39848,39810,39758,39706,39665,39623,39577,39524,39482,39449,39411,39374,39338,39304,39268,39222,39176,39138,39088,39057,39016,38980,38946,38912,38873,38835,38795,38759,38725,38677,38633,38595,38565,38523,38487,38445,38415,38377,38336,38300,38254,38212,38189,38161,38127,38090,38056,38015,37961,37919,37886,37850,37821,37786,37759,37718,37688,37649,37596,37562,37523,37476,37435,37395,37354,37313,37270,37227,37195,37170,37132,37085,37039,37002,36954,36915,36887,36854,36822,36780,36735,36706,36665,36626,36595,36566,36540,36497,36463,36434,36410,36383,36339,36305,36274,36233,36200,36151,36107,36075,36028,35991,35954,35925,35890,35860,35820,35785,35747,35706,35679,35635,35598,35577,35550,35518,35485,35453,35421,35385,35349,35308,35276,35224,35189,35149,35111,35070,35034,35007,34972,34935,34894,34851,34827,34793,34756,34721,34684,34655,34618,34583,34544,34505,34466,34427,34393,34361,34318,34281,34255,34226,34193,34161,34134,34104,34075,34042,34005,33974,33942,33906,33864,33819,33786,33749,33714,33694,33668,33637,33605,33577,33542,33512,33483,33456,33413,33372,33341,33301,33268,33233,33200,33165,33131,33096,33062,33025,32994,32969,32942,32913,32881,32849,32817,32786,32750,32719,32681,32644,32606,32574,32543,32514,32471,32430,32393,32360,32334,32305,32277,32248,32213,32170,32135,32095,32067,32030,31999,31966,31927,31888,31851,31812,31780,31735,31704,31677,31637,31595,31566,31534,31503,31454,31421,31385,31355,31310,31278,31261,31225,31184,31148,31118,31081,31054,31029,31002,30965,30936,30904,30870,30845,30819,30774,30736,30693,30656,30633,30601,30569,30544,30513,30485,30453,30419,30378,30343,30323,30295,30274,30252,30220,30196,30160,30123,30084,30057,30025,30001,29977,29947,29908,29874,29834,29804,29771,29739,29708,29680,29638,29608,29576,29547,29516,29482,29454,29418,29389,29363,29335,29299,29262,29226,29200,29171,29146,29119,29093,29068,29045,29020,28989,28955,28925,28902,28869,28841,28811,28782,28759,28732,28698,28665,28648,28617,28587,28556,28519,28485,28464,28435,28402,28374,28344,28317,28283,28252,28226,28195,28168,28128,28093,28058,28026,27998,27971,27950,27922,27891,27862,27837,27800,27773,27745,27729,27702,27670,27631,27595,27570,27550,27516,27482,27459,27433,27405,27367,27335,27313,27281,27248,27226,27190,27166,27140,27110,27078,27049,27007,26968,26950,26922,26884,26850,26821,26802,26773,26742,26717,26690,26666,26633,26608,26584,26553,26519,26490,26467,26442,26408,26378,26347,26316,26285,26254,26224,26195,26164,26138,26117,26090,26063,26036,26000,25966,25926,25905,25875,25847,25815,25792,25761,25740,25716,25695,25669,25637,25601,25576,25551,25515,25491,25467,25435,25415,25393,25360,25328,25300,25281,25250,25223,25188,25164,25137,25106,25088,25064,25037,25010,24981,24960,24938,24910,24882,24856,24825,24795,24771,24752,24730,24694,24674,24654,24631,24603,24585,24564,24541,24518,24500,24472,24453,24421,24404,24374,24350,24335,24310,24289,24263,24245,24218,24188,24171,24152,24127,24102,24077,24050,24034,24013,23997,23972,23952,23925,23904,23883,23865,23842,23824,23808,23785,23757,23738,23712,23688,23666,23637,23610,23586,23560,23544,23515,23504,23484,23457,23437,23412,23387,23367,23334,23311,23286,23260,23240,23211,23189,23166,23141,23110,23087,23054,23023,23003,22984,22963,22936,22915,22884,22861,22830,22802,22783,22760,22729,22708,22686,22656,22628,22603,22578,22566,22535,22508,22487,22464,22439,22412,22390,22371,22346,22319,22296,22270,22255,22227,22203,22178,22161,22136,22106,22075,22049,22026,22003,21981,21960,21938,21913,21892,21866,21835,21809,21787,21757,21736,21706,21672,21649,21625,21590,21568,21542,21522,21496,21476,21452,21429,21399,21368,21338,21312,21287,21265,21236,21213,21190,21168,21146,21113,21083,21057,21027,21008,20983,20960,20932,20905,20872,20841,20820,20794,20769,20748,20727,20711,20688,20663,20644,20619,20596,20564,20536,20510,20480,20449,20425,20401,20377,20358,20329,20306,20285,20255,20248,20228,20203,20185,20154,20120,20081,20057,20030,20005,19979,19953,19927,19901,19869,19840,19811,19784,19752,19727,19696,19670,19644,19614,19594,19555,19524,19499,19467,19435,19410,19383,19355,19321,19283,19257,19224,19189,19152,19121,19098,19069,19042,19006,18978,18946,18924,18891,18857,18829,18813,18791,18767,18738,18702,18668,18649,18624,18604,18574,18543,18500,18473,18447,18419,18378,18354,18327,18300,18265,18228,18195,18158,18127,18088,18057,18027,17998,17961,17924,17890,17855,17821,17802,17780,17747,17713,17682,17652,17609,17580,17552,17517,17489,17458,17436,17402,17382,17356,17324,17293,17259,17233,17205,17172,17142,17108,17075,17035,16995,16963,16937,16906,16874,16848,16813,16789,16767,16735,16703,16666,16632,16593,16560,16524,16481,16432,16391,16362,16317,16285,16250,16220,16194,16164,16126,16086,16059,16027,15994,15971,15921,15878,15840,15813,15781,15737,15689,15651,15609,15580,15537,15495,15451,15410,15372,15337,15298,15258,15220,15185,15149,15092,15043,15001,14972,14940,14898,14862,14831,14812,14784,14740,14702,14656,14624,14590,14555,14513,14483,14437,14393,14357,14314,14265,14234,14200,14157,14122,14089,14052,14006,13963,13920,13869,13826,13799,13764,13726,13696,13652,13602,13559,13523,13485,13441,13399,13360,13309,13256,13214,13164,13111,13066,13034,12995,12950,12908,12876,12847,12803,12769,12737,12698,12649,12601,12565,12515,12460,12401,12343,12295,12231,12163,12117,12070,12028,11973,11914,11871,11816,11753,11696,11631,11575,11512,11464,11411,11351,11292,11234,11186,11119,11041,10989,10940,10871,10793,10733,10665,10607,10551,10472,8418],[584079,440567,382725,352165,328522,307384,289896,276219,265142,252973,241722,231852,223892,215962,209091,204916,200196,195987,191238,186687,182961,179494,175096,171997,168919,165595,162574,160136,157775,155343,152694,150509,148069,146069,144020,141319,139048,137421,136192,134619,132713,131236,129406,127833,126488,124816,123315,121598,120125,118799,117652,116557,115104,113576,112483,111379,110518,109595,108576,107709,106854,106227,105451,104400,103416,102421,101627,100812,99917,99131,98109,97387,96741,96179,95461,94755,94222,93458,92660,92118,91471,90843,90276,89691,89019,88472,87885,87321,86781,86288,85852,85231,84628,84047,83465,82927,82380,81944,81434,81038,80684,80202,79902,79460,79051,78652,78404,78035,77579,77247,76856,76348,75914,75508,75205,74927,74662,74390,74101,73733,73419,73011,72652,72347,71999,71733,71471,71216,70949,70663,70401,70164,69840,69575,69274,69053,68818,68548,68301,68053,67766,67494,67219,66920,66699,66448,66256,66068,65845,65589,65371,65185,64995,64795,64562,64303,64088,63894,63689,63521,63321,63110,62923,62782,62583,62396,62211,62023,61826,61674,61466,61258,61092,60923,60763,60618,60445,60284,60108,59942,59790,59653,59499,59356,59171,59024,58870,58701,58522,58352,58241,58083,57942,57802,57691,57539,57341,57236,57104,56945,56817,56684,56533,56378,56261,56145,56037,55916,55805,55693,55532,55405,55297,55175,55060,54918,54838,54715,54625,54511,54407,54318,54207,54082,53933,53832,53738,53637,53526,53394,53278,53176,53072,52946,52831,52708,52621,52495,52403,52292,52184,52087,51988,51889,51788,51693,51592,51506,51414,51311,51234,51154,51084,50993,50911,50843,50759,50700,50618,50527,50442,50384,50300,50221,50136,50045,49960,49873,49775,49685,49563,49463,49364,49272,49180,49107,49042,48960,48869,48793,48725,48633,48558,48483,48422,48333,48279,48218,48174,48116,48037,47963,47897,47825,47740,47669,47634,47566,47510,47457,47386,47315,47241,47176,47102,47022,46969,46901,46838,46765,46714,46644,46587,46537,46481,46419,46335,46281,46208,46135,46060,45997,45942,45881,45820,45768,45695,45629,45562,45488,45439,45400,45351,45293,45244,45178,45114,45058,44990,44933,44866,44799,44742,44693,44636,44575,44512,44458,44402,44343,44290,44236,44179,44115,44045,43988,43939,43885,43837,43794,43750,43703,43653,43598,43550,43486,43436,43380,43323,43273,43221,43172,43128,43068,43013,42953,42886,42827,42773,42721,42675,42628,42555,42501,42466,42420,42382,42341,42276,42214,42169,42125,42067,42011,41966,41922,41874,41831,41782,41727,41673,41623,41582,41532,41475,41411,41366,41336,41302,41260,41223,41185,41139,41092,41036,40984,40934,40882,40838,40804,40761,40712,40656,40615,40576,40538,40493,40437,40388,40331,40289,40248,40205,40161,40127,40077,40037,39990,39945,39916,39883,39837,39788,39741,39705,39665,39619,39575,39517,39479,39431,39381,39336,39303,39261,39214,39173,39140,39092,39067,39027,38988,38943,38903,38857,38814,38770,38738,38699,38663,38628,38598,38565,38537,38503,38468,38431,38393,38346,38320,38281,38235,38201,38171,38134,38098,38054,38006,37974,37937,37904,37865,37837,37799,37771,37741,37707,37679,37639,37603,37566,37532,37496,37459,37430,37395,37364,37328,37292,37252,37218,37184,37158,37113,37073,37036,36991,36956,36923,36897,36870,36841,36813,36779,36737,36699,36657,36620,36587,36549,36509,36467,36440,36406,36364,36330,36290,36255,36218,36173,36141,36098,36069,36024,35987,35956,35929,35899,35867,35834,35796,35752,35717,35687,35652,35616,35594,35569,35537,35511,35476,35450,35407,35373,35330,35299,35272,35226,35201,35173,35147,35110,35067,35038,35013,34982,34943,34913,34879,34849,34822,34786,34761,34730,34696,34658,34628,34593,34560,34530,34493,34458,34425,34393,34351,34312,34278,34248,34203,34167,34131,34097,34069,34038,34015,33973,33942,33913,33875,33837,33801,33774,33744,33716,33693,33669,33641,33612,33592,33569,33534,33508,33482,33453,33414,33377,33343,33312,33261,33221,33187,33158,33128,33100,33066,33033,32999,32971,32941,32910,32888,32853,32820,32791,32765,32733,32699,32666,32638,32596,32569,32544,32509,32471,32435,32405,32367,32343,32310,32283,32252,32225,32186,32154,32122,32080,32045,32012,31974,31943,31910,31861,31822,31792,31766,31731,31692,31662,31620,31588,31556,31532,31500,31469,31437,31402,31373,31340,31308,31279,31250,31215,31183,31153,31119,31086,31058,31029,31007,30962,30934,30903,30869,30841,30813,30775,30746,30716,30669,30642,30612,30589,30553,30529,30495,30460,30430,30412,30377,30344,30319,30290,30270,30243,30216,30192,30160,30130,30095,30066,30031,29999,29978,29955,29911,29879,29855,29821,29785,29755,29719,29688,29654,29620,29597,29560,29530,29496,29463,29438,29400,29367,29331,29303,29273,29242,29214,29184,29155,29133,29100,29078,29057,29033,29001,28976,28939,28912,28887,28860,28835,28799,28776,28751,28719,28691,28662,28631,28598,28567,28540,28509,28478,28458,28431,28405,28371,28347,28322,28282,28244,28222,28196,28167,28128,28095,28061,28036,28004,27979,27954,27937,27903,27877,27848,27818,27794,27773,27751,27734,27713,27680,27651,27625,27592,27567,27540,27514,27488,27456,27433,27412,27391,27357,27326,27306,27280,27248,27228,27201,27177,27152,27135,27106,27078,27047,27016,26982,26957,26930,26905,26884,26859,26832,26811,26801,26774,26749,26719,26689,26665,26642,26619,26590,26562,26526,26500,26472,26453,26429,26396,26370,26351,26328,26307,26280,26250,26227,26207,26182,26159,26135,26112,26086,26056,26028,26000,25970,25942,25922,25906,25876,25849,25824,25797,25780,25752,25734,25710,25680,25660,25636,25612,25586,25563,25540,25508,25492,25465,25433,25414,25396,25364,25344,25319,25298,25272,25251,25224,25196,25168,25146,25133,25116,25093,25075,25042,25020,25003,24986,24960,24944,24920,24888,24863,24843,24813,24785,24761,24746,24723,24700,24680,24669,24643,24615,24593,24570,24548,24518,24499,24482,24456,24436,24414,24391,24363,24346,24327,24310,24285,24272,24258,24232,24213,24187,24170,24154,24132,24114,24099,24068,24045,24024,23998,23984,23960,23937,23920,23899,23879,23860,23832,23816,23797,23773,23748,23736,23712,23688,23674,23650,23627,23608,23591,23567,23546,23523,23504,23486,23468,23452,23426,23408,23389,23374,23350,23322,23296,23279,23258,23244,23218,23196,23178,23166,23140,23120,23096,23077,23054,23030,23010,22994,22984,22958,22937,22918,22892,22870,22854,22830,22805,22786,22770,22746,22723,22698,22688,22666,22646,22623,22607,22571,22554,22531,22506,22485,22464,22435,22412,22399,22383,22360,22345,22319,22292,22268,22246,22226,22199,22178,22161,22145,22113,22089,22066,22046,22022,22005,21989,21970,21952,21933,21913,21892,21867,21851,21830,21803,21781,21759,21736,21710,21686,21660,21638,21611,21581,21560,21538,21523,21499,21476,21457,21444,21423,21398,21373,21344,21320,21295,21274,21260,21240,21216,21200,21180,21164,21140,21124,21101,21085,21060,21034,21010,21000,20981,20956,20939,20909,20887,20864,20844,20826,20802,20784,20771,20748,20730,20710,20681,20668,20644,20625,20599,20575,20561,20536,20511,20488,20467,20445,20422,20394,20377,20358,20335,20313,20300,20277,20254,20245,20228,20207,20183,20164,20127,20099,20079,20065,20046,20022,20005,19978,19950,19922,19897,19876,19859,19832,19810,19785,19760,19737,19717,19698,19677,19656,19631,19614,19604,19582,19554,19527,19507,19492,19466,19438,19422,19399,19381,19354,19338,19308,19286,19264,19240,19217,19191,19169,19138,19123,19101,19080,19057,19030,19006,18980,18959,18930,18916,18889,18867,18833,18816,18798,18772,18748,18721,18696,18671,18668,18643,18618,18603,18579,18553,18519,18494,18474,18460,18435,18417,18400,18369,18344,18320,18291,18264,18238,18222,18193,18161,18141,18112,18096,18070,18048,18019,18003,17987,17966,17941,17914,17890,17870,17852,17819,17804,17784,17760,17738,17720,17703,17680,17656,17633,17608,17582,17570,17543,17519,17498,17469,17445,17418,17393,17368,17354,17330,17306,17289,17264,17242,17220,17201,17177,17157,17130,17108,17083,17060,17028,16992,16960,16941,16918,16894,16869,16848,16826,16803,16796,16775,16752,16724,16698,16680,16652,16619,16587,16556,16525,16499,16470,16443,16423,16392,16376,16354,16337,16309,16285,16252,16231,16205,16182,16159,16126,16108,16080,16049,16012,15990,15969,15929,15896,15873,15846,15819,15794,15770,15728,15696,15666,15634,15607,15577,15559,15528,15506,15477,15443,15418,15395,15375,15348,15332,15297,15264,15243,15215,15190,15161,15146,15103,15069,15032,15005,14984,14956,14934,14906,14886,14862,14838,14820,14797,14784,14763,14738,14712,14687,14664,14636,14600,14579,14555,14524,14497,14473,14456,14430,14405,14382,14355,14330,14299,14256,14230,14200,14169,14137,14110,14077,14045,14017,13988,13951,13930,13901,13866,13845,13821,13797,13776,13754,13722,13702,13682,13641,13609,13587,13561,13531,13505,13477,13444,13408,13380,13344,13314,13278,13258,13234,13202,13166,13139,13106,13081,13056,13019,12990,12963,12929,12905,12875,12843,12805,12775,12743,12709,12673,12646,12612,12586,12562,12537,12506,12476,12444,12413,12379,12342,12313,12287,12255,12216,12177,12145,12119,12097,12068,12052,12019,11988,11956,11921,11891,11865,11829,11795,11760,11723,11693,11663,11617,11582,11536,11501,11473,11431,11403,11370,11331,11301,11262,11223,11192,11164,11123,11090,11061,11015,10981,10948,10907,10861,10826,10797,10768,10730,10699,10673,10638,10608,10588,10557,10520,10478,10442,10405]],"filenames":["SPAdes MEGAN","SPAdes MetaWatt","metaSPAdes MEGAN","metaSPAdes MetaWatt","IDBA-UD MEGAN","IDBA-UD MetaWatt","MetaVelvet MEGAN","MetaVelvet MetaWatt","omega MEGAN","omega MetaWatt","mira MEGAN","mira MetaWatt"]}

{"assemblies\_lengths":[38671927,36768376,37514446,36436585,38442682,36813103,36134292,35276741,27785829,20188275,58701573,58791663],"filenames":["SPAdes MEGAN","SPAdes MetaWatt","metaSPAdes MEGAN","metaSPAdes MetaWatt","IDBA-UD MEGAN","IDBA-UD MetaWatt","MetaVelvet MEGAN","MetaVelvet MetaWatt","omega MEGAN","omega MetaWatt","mira MEGAN","mira MetaWatt"]}

{"reflen":[732541,716303,630155,573690,570865,567381,546657,532226,516248,504301,488836,466440,462416,461818,447826,442055,413805,411754,404769,384467,373358,365257,363261,361866,354730,354419,353928,348940,343826,341765,339915,339382,338815,335967,334716,332479,323691,317967,311858,304408,304155,301963,299886,296022,290336,289622,285051,284413,282225,271036,264454,246552,239730,232062,230988,228812,226694,226551,224248,222846,222335,221796,221681,218075,217148,216643,215090,211422,208103,203932,202592,202135,201920,197825,196752,197057,191261,190284,188134,187044,185518,182912,181662,181022,179040,178853,176674,175850,172819,171668,171511,168396,165620,164421,164249,164041,161197,161192,160547,156394,156416,155621,154311,151354,145101,141948,141740,140510,139666,136509,132781,131944,129426,127888,125453,124439,121619,116377,115007,112235,112014,111107,109193,111539,107837,107400,105358,103913,103081,103209,101080,100297,100303,100299,100063,98451,98201,95931,95666,92451,92286,89942,86611,86474,85269,83128,82503,82408,81745,81223,78729,78230,79059,77139,75297,74468,72820,72394,72239,71754,71545,71060,69435,69179,68454,68659,67720,66751,66125,64508,64279,64042,63737,63362,62588,60471,60212,60149,58998,58892,58304,58270,57735,56932,56727,56280,56120,55478,55097,54377,54288,54417,53880,53455,53308,51704,50424,50122,49523,48878,48799,47614,45669,45680,42081,41211,41053,40947,40686,40103,40307,39313,37694,37424,37356,37341,35284,34742,33387,32888,32404,32292,33788,30195,29375,28741,29328,27322,26599,26385,26213,26176,25783,25654,25446,25406,25305,22825,21571,20856,20819,19572,19580,17916,17224,16884,16288,14434,12886,12836,12398,12431,11353,11182,11114,10788,10514,9908,9697,9316,11193,9114,8560,8513,7051,6654,4956,4893,4497,3420,3253,3142,2359,1886,1615,1533,931]}

{"tickX":26}

{"coord\_y":[[332411,332411,332411,254571,254571,247717,247717,195466,195466,191156,191156,177765,177765,173612,173612,170239,170239,167106,167106,162871,162871,159923,159923,157524,157524,156399,156399,151375,151375,150363,150363,150230,150230,148339,148339,147170,147170,146100,146100,144023,144023,143007,143007,142465,142465,138341,138341,135668,135668,135080,135080,132702,132702,130898,130898,129441,129441,128572,128572,128070,128070,127938,127938,127273,127273,126977,126977,126334,126334,124655,124655,124528,124528,124112,124112,118504,118504,114846,114846,112261,112261,111956,111956,111544,111544,110988,110988,110374,110374,109303,109303,108924,108924,108909,108909,108095,108095,107066,107066,106883,106883,106435,106435,106381,106381,105942,105942,105561,105561,105314,105314,104707,104707,103299,103299,102203,102203,101640,101640,100927,100927,98942,98942,98585,98585,97453,97453,96365,96365,96300,96300,96121,96121,95867,95867,95777,95777,95285,95285,95087,95087,94790,94790,94131,94131,93585,93585,92562,92562,91919,91919,91600,91600,91574,91574,90991,90991,90730,90730,90283,90283,89642,89642,89107,89107,88857,88857,87693,87693,87392,87392,86692,86692,86347,86347,86295,86295,85924,85924,85704,85704,85066,85066,83926,83926,83817,83817,83546,83546,83117,83117,82365,82365,82258,82258,81871,81871,80401,80401,80366,80366,80274,80274,79744,79744,79739,79739,79287,79287,79219,79219,79146,79146,78966,78966,78351,78351,77263,77263,76932,76932,76501,76501,76344,76344,76128,76128,75532,75532,75422,75422,74880,74880,74834,74834,74339,74339,74269,74269,73728,73728,73462,73462,72750,72750,72204,72204,71895,71895,71573,71573,71226,71226,71223,71223,71156,71156,70768,70768,70651,70651,70049,70049,69795,69795,69775,69775,69718,69718,69696,69696,69540,69540,69083,69083,69060,69060,69050,69050,68924,68924,68611,68611,68435,68435,67470,67470,67449,67449,67390,67390,67136,67136,66989,66989,66730,66730,66721,66721,66639,66639,66473,66473,66338,66338,66015,66015,65892,65892,65785,65785,65488,65488,65181,65181,65108,65108,64909,64909,64218,64218,64185,64185,63935,63935,63304,63304,62514,62514,61362,61362,60953,60953,60524,60524,59767,59767,59529,59529,59450,59450,59375,59375,59366,59366,59205,59205,59082,59082,58983,58983,58593,58593,58454,58454,58023,58023,57884,57884,57768,57768,57415,57415,57410,57410,57403,57403,57149,57149,57058,57058,57022,57022,56967,56967,56963,56963,56865,56865,56583,56583,56477,56477,56452,56452,55889,55889,55472,55472,55402,55402,55353,55353,54988,54988,54970,54970,54880,54880,54346,54346,54292,54292,54270,54270,54046,54046,54038,54038,54024,54024,54022,54022,53953,53953,53683,53683,53350,53350,53337,53337,53294,53294,53245,53245,53156,53156,53042,53042,53038,53038,52948,52948,52925,52925,52516,52516,52209,52209,52192,52192,52110,52110,51856,51856,51412,51412,51365,51365,51312,51312,51113,51113,51086,51086,51045,51045,50814,50814,50685,50685,50329,50329,50314,50314,50307,50307,50277,50277,50149,50149,49613,49613,49135,49135,48820,48820,48594,48594,48587,48587,48516,48516,48408,48408,48332,48332,48273,48273,47951,47951,47700,47700,47659,47659,47610,47610,47456,47456,47298,47298,47228,47228,47125,47125,46987,46987,46922,46922,46897,46897,46896,46896,46621,46621,46556,46556,46483,46483,46359,46359,46146,46146,45900,45900,45808,45808,45699,45699,45652,45652,45621,45621,45512,45512,45465,45465,45281,45281,45075,45075,45033,45033,45024,45024,44836,44836,44811,44811,44617,44617,44593,44593,44533,44533,44487,44487,44405,44405,44333,44333,44224,44224,44220,44220,44079,44079,44066,44066,44064,44064,44058,44058,44040,44040,44020,44020,43806,43806,43778,43778,43734,43734,43516,43516,43361,43361,43304,43304,43142,43142,43034,43034,42955,42955,42946,42946,42935,42935,42756,42756,42682,42682,42671,42671,42427,42427,42308,42308,42279,42279,42160,42160,42156,42156,41750,41750,41644,41644,41439,41439,41398,41398,41393,41393,41295,41295,41022,41022,40917,40917,40906,40906,40849,40849,40835,40835,40754,40754,40734,40734,40649,40649,40544,40544,40373,40373,40222,40222,40091,40091,40069,40069,39873,39873,39736,39736,39679,39679,39661,39661,39582,39582,39357,39357,39348,39348,39210,39210,39146,39146,39060,39060,39009,39009,39000,39000,38842,38842,38823,38823,38767,38767,38756,38756,38530,38530,38285,38285,38167,38167,38162,38162,37999,37999,37800,37800,37758,37758,37608,37608,37538,37538,37415,37415,37273,37273,37117,37117,36925,36925,36725,36725,36677,36677,36542,36542,36415,36415,36242,36242,36135,36135,36077,36077,36027,36027,35985,35985,35965,35965,35905,35905,35644,35644,35608,35608,35458,35458,35394,35394,35207,35207,35019,35019,34916,34916,34848,34848,34843,34843,34771,34771,34686,34686,34596,34596,34585,34585,34582,34582,34365,34365,34028,34028,33864,33864,33784,33784,33707,33707,33690,33690,33574,33574,33538,33538,33518,33518,33498,33498,33287,33287,33218,33218,33133,33133,33098,33098,32979,32979,32938,32938,32810,32810,32745,32745,32740,32740,32739,32739,32681,32681,32669,32669,32455,32455,32403,32403,32399,32399,32220,32220,32212,32212,32190,32190,32088,32088,32055,32055,31856,31856,31754,31754,31701,31701,31657,31657,31475,31475,31378,31378,31230,31230,31205,31205,31196,31196,31167,31167,31029,31029,30936,30936,30868,30868,30852,30852,30653,30653,30594,30594,30516,30516,30402,30402,30308,30308,30300,30300,30272,30272,30059,30059,30040,30040,29962,29962,29908,29908,29888,29888,29835,29835,29803,29803,29780,29780,29730,29730,29697,29697,29646,29646,29624,29624,29617,29617,29563,29563,29435,29435,29266,29266,29184,29184,29122,29122,29078,29078,28991,28991,28861,28861,28814,28814,28787,28787,28778,28778,28763,28763,28745,28745,28740,28740,28666,28666,28664,28664,28660,28660,28651,28651,28629,28629,28490,28490,28467,28467,28461,28461,28345,28345,28202,28202,27925,27925,27838,27838,27783,27783,27581,27581,27551,27551,27514,27514,27439,27439,27360,27360,27344,27344,27325,27325,27308,27308,27306,27306,27250,27250,27240,27240,27079,27079,27057,27057,26884,26884,26637,26637,26623,26623,26614,26614,26538,26538,26518,26518,26440,26440,26327,26327,26310,26310,26303,26303,26289,26289,26284,26284,26216,26216,26188,26188,26144,26144,26036,26036,26034,26034,26008,26008,25906,25906,25879,25879,25857,25857,25850,25850,25837,25837,25801,25801,25701,25701,25600,25600,25565,25565,25557,25557,25462,25462,25438,25438,25355,25355,25165,25165,24983,24983,24951,24951,24805,24805,24641,24641,24592,24592,24581,24581,24563,24563,24551,24551,24349,24349,24348,24348,24236,24236,24200,24200,24192,24192,24118,24118,24024,24024,23979,23979,23957,23957,23848,23848,23488,23488,23484,23484,23462,23462,23390,23390,23190,23190,23187,23187,23153,23153,23035,23035,22954,22954,22801,22801,22783,22783,22722,22722,22668,22668,22665,22665,22560,22560,22497,22497,22467,22467,22408,22408,22279,22279,22206,22206,22201,22201,22191,22191,22180,22180,22120,22120,22075,22075,22026,22026,22002,22002,21999,21999,21895,21895,21859,21859,21801,21801,21760,21760,21727,21727,21407,21407,21396,21396,21367,21367,21328,21328,21275,21275,21219,21219,21161,21161,21133,21133,21112,21112,21071,21071,21025,21025,21020,21020,21004,21004,20870,20870,20853,20853,20849,20849,20824,20824,20804,20804,20754,20754,20693,20693,20676,20676,20670,20670,20618,20618,20490,20490,20456,20456,20409,20409,20319,20319,20292,20292,20279,20279,20274,20274,20201,20201,20160,20160,20116,20116,20075,20075,19930,19930,19891,19891,19807,19807,19791,19791,19785,19785,19747,19747,19727,19727,19671,19671,19596,19596,19526,19526,19451,19451,19429,19429,19417,19417,19327,19327,19257,19257,19253,19253,19210,19210,19133,19133,19100,19100,19097,19097,19062,19062,19048,19048,19046,19046,19029,19029,19008,19008,18995,18995,18930,18930,18915,18915,18890,18890,18864,18864,18836,18836,18744,18744,18733,18733,18717,18717,18594,18594,18589,18589,18540,18540,18494,18494,18492,18492,18302,18302,18249,18249,18224,18224,18123,18123,18116,18116,18113,18113,18062,18062,18061,18061,18051,18051,18000,18000,17995,17995,17897,17897,17869,17869,17836,17836,17741,17741,17677,17677,17676,17676,17619,17619,17596,17596,17587,17587,17549,17549,17514,17514,17513,17513,17504,17504,17499,17499,17442,17442,17431,17431,17342,17342,17292,17292,17272,17272,17199,17199,17145,17145,17116,17116,17064,17064,17025,17025,17018,17018,17000,17000,16942,16942,16844,16844,16811,16811,16720,16720,16717,16717,16708,16708,16700,16700,16672,16672,16669,16669,16619,16619,16532,16532,16451,16451,16448,16448,16377,16377,16357,16357,16300,16300,16216,16216,16213,16213,16191,16191,16026,16026,16017,16017,15976,15976,15916,15916,15892,15892,15867,15867,15802,15802,15755,15755,15738,15738,15693,15693,15645,15645,15533,15533,15430,15430,15426,15426,15260,15260,15202,15202,15136,15136,15115,15115,15059,15059,14980,14980,14937,14937,14914,14914,14908,14908,14829,14829,14769,14769,14765,14765,14654,14654,14580,14580,14557,14557,14543,14543,14496,14496,14479,14479,14373,14373,14309,14309,14289,14289,14280,14280,14278,14278,14252,14252,14182,14182,14127,14127,14125,14125,14101,14101,14043,14043,14026,14026,13969,13969,13934,13934,13909,13909,13858,13858,13851,13851,13698,13698,13615,13615,13585,13585,13563,13563,13540,13540,13396,13396,13318,13318,13315,13315,13302,13302,13291,13291,13273,13273,13271,13271,13177,13177,13134,13134,13112,13112,13110,13110,13076,13076,13073,13073,13064,13064,13045,13045,13042,13042,13014,13014,12973,12973,12960,12960,12958,12958,12920,12920,12888,12888,12862,12862,12829,12829,12805,12805,12804,12804,12756,12756,12651,12651,12647,12647,12585,12585,12578,12578,12564,12564,12463,12463,12450,12450,12393,12393,12348,12348,12344,12344,12334,12334,12315,12315,12285,12285,12236,12236,12201,12201,12189,12189,12167,12167,12146,12146,12135,12135,12117,12117,12116,12116,12072,12072,12050,12050,12036,12036,12011,12011,11908,11908,11901,11901,11885,11885,11800,11800,11716,11716,11679,11679,11625,11625,11597,11597,11592,11592,11550,11550,11498,11498,11474,11474,11435,11435,11432,11432,11361,11361,11285,11285,11176,11176,11133,11133,11088,11088,11067,11067,11033,11033,11024,11024,10963,10963,10961,10961,10922,10922,10853,10853,10832,10832,10760,10760,10744,10744,10742,10742,10720,10720,10712,10712,10707,10707,10706,10706,10577,10577,10568,10568,10547,10547,10442,10442,10413,10413,10399,10399,10360,10360,10357,10357,10288,10288,10264,10264,10259,10259,10256,10256,10159,10159,10146,10146,10105,10105,10088,10088,10082,10082,10047,10047,10041,10041,10009,10009,9954,9954,9910,9910,9899,9899,9873,9873,9859,9859,9849,9849,9820,9820,9770,9770,9726,9726,9639,9639,9630,9630,9546,9546,9536,9536,9522,9522,9390,9390,9357,9357,9336,9336,9320,9320,9319,9319,9305,9305,9247,9247,9203,9203,9186,9186,9161,9161,9147,9147,9109,9109,9058,9058,9054,9054,9012,9012,9010,9010,9004,9004,8972,8972,8956,8956,8949,8949,8925,8925,8869,8869,8833,8833,8807,8807,8793,8793,8750,8750,8699,8699,8697,8697,8682,8682,8569,8569,8560,8560,8524,8524,8490,8490,8434,8434,8389,8389,8340,8340,8335,8335,8281,8281,8260,8260,8254,8254,8216,8216,8194,8194,8097,8097,8047,8047,8004,8004,7982,7982,7962,7962,7928,7928,7922,7922,7912,7912,7890,7890,7863,7863,7840,7840,7813,7813,7727,7727,7708,7708,7682,7682,7678,7678,7649,7649,7644,7644,7632,7632,7628,7628,7626,7626,7607,7607,7572,7572,7556,7556,7540,7540,7520,7520,7519,7519,7507,7507,7485,7485,7450,7450,7449,7449,7433,7433,7416,7416,7414,7414,7404,7404,7397,7397,7370,7370,7358,7358,7355,7355,7297,7297,7213,7213,7137,7137,7088,7088,7006,7006,6987,6987,6984,6984,6979,6979,6959,6959,6958,6958,6848,6848,6792,6792,6742,6742,6642,6642,6618,6618,6605,6605,6574,6574,6536,6536,6518,6518,6508,6508,6488,6488,6486,6486,6441,6441,6400,6400,6372,6372,6361,6361,6344,6344,6274,6274,6138,6138,6136,6136,6052,6052,6028,6028,6004,6004,5973,5973,5960,5960,5888,5888,5878,5878,5866,5866,5852,5852,5818,5818,5817,5817,5766,5766,5693,5693,5649,5649,5623,5623,5618,5618,5607,5607,5601,5601,5562,5562,5557,5557,5528,5528,5515,5515,5512,5512,5490,5490,5476,5476,5435,5435,5434,5434,5428,5428,5411,5411,5372,5372,5345,5345,5341,5341,5319,5319,5298,5298,5282,5282,5270,5270,5268,5268,5261,5261,5247,5247,5233,5233,5217,5217,5216,5216,5215,5215,5209,5209,5203,5203,5184,5184,5173,5173,5142,5142,5134,5134,5096,5096,5081,5081,5055,5055,5044,5044,5040,5040,5027,5027,4994,4994,4990,4990,4936,4936,4928,4928,4923,4923,4891,4891,4878,4878,4866,4866,4808,4808,4797,4797,4788,4788,4775,4775,4761,4761,4760,4760,4708,4708,4702,4702,4668,4668,4663,4663,4654,4654,4653,4653,4606,4606,4605,4605,4588,4588,4566,4566,4530,4530,4506,4506,4481,4481,4478,4478,4472,4472,4465,4465,4406,4406,4401,4401,4399,4399,4386,4386,4375,4375,4340,4340,4318,4318,4244,4244,4213,4213,4210,4210,4209,4209,4208,4208,4193,4193,4166,4166,4113,4113,4112,4112,4089,4089,4087,4087,4061,4061,4021,4021,4002,4002,3970,3970,3937,3937,3860,3860,3837,3837,3835,3835,3830,3830,3819,3819,3800,3800,3798,3798,3788,3788,3786,3786,3782,3782,3778,3778,3762,3762,3747,3747,3746,3746,3734,3734,3710,3710,3709,3709,3706,3706,3696,3696,3669,3669,3628,3628,3611,3611,3603,3603,3593,3593,3566,3566,3560,3560,3549,3549,3500,3500,3492,3492,3490,3490,3471,3471,3433,3433,3423,3423,3420,3420,3417,3417,3408,3408,3337,3337,3309,3309,3290,3290,3288,3288,3277,3277,3255,3255,3253,3253,3145,3145,3120,3120,3086,3086,3072,3072,3067,3067,3054,3054,2982,2982,2958,2958,2940,2940,2902,2902,2891,2891,2835,2835,2806,2806,2804,2804,2796,2796,2789,2789,2788,2788,2784,2784,2778,2778,2753,2753,2728,2728,2727,2727,2716,2716,2690,2690,2668,2668,2664,2664,2661,2661,2648,2648,2644,2644,2601,2601,2583,2583,2567,2567,2519,2519,2495,2495,2494,2494,2490,2490,2474,2474,2411,2411,2399,2399,2378,2378,2371,2371,2364,2364,2363,2363,2340,2340,2338,2338,2335,2335,2334,2334,2331,2331,2326,2326,2317,2317,2316,2316,2315,2315,2308,2308,2307,2307,2300,2300,2297,2297,2286,2286,2246,2246,2243,2243,2238,2238,2213,2213,2211,2211,2191,2191,2185,2185,2184,2184,2180,2180,2174,2174,2161,2161,2149,2149,2146,2146,2141,2141,2135,2135,2114,2114,2107,2107,2105,2105,2097,2097,2096,2096,2080,2080,2060,2060,2037,2037,2034,2034,2023,2023,1987,1987,1984,1984,1982,1982,1980,1980,1966,1966,1963,1963,1962,1962,1961,1961,1953,1953,1938,1938,1922,1922,1883,1883,1881,1881,1876,1876,1846,1846,1833,1833,1806,1806,1804,1804,1791,1791,1786,1786,1785,1785,1773,1773,1770,1770,1767,1767,1760,1760,1755,1755,1740,1740,1724,1724,1715,1715,1703,1703,1685,1685,1684,1684,1680,1680,1664,1664,1655,1655,1648,1648,1640,1640,1638,1638,1635,1635,1630,1630,1609,1609,1605,1605,1604,1604,1602,1602,1595,1595,1591,1591,1580,1580,1564,1564,1563,1563,1562,1562,1547,1547,1541,1541,1539,1539,1534,1534,1533,1533,1532,1532,1523,1523,1518,1518,1516,1516,1512,1512,1506,1506,1499,1499,1493,1493,1479,1479,1475,1475,1467,1467,1463,1463,1459,1459,1454,1454,1452,1452,1443,1443,1436,1436,1434,1434,1433,1433,1431,1431,1429,1429,1422,1422,1419,1419,1412,1412,1405,1405,1392,1392,1387,1387,1385,1385,1384,1384,1378,1378,1365,1365,1363,1363,1361,1361,1360,1360,1350,1350,1349,1349,1342,1342,1328,1328,1291,1291,1283,1283,1272,1272,1265,1265,1256,1256,1250,1250,1249,1249,1243,1243,1237,1237,1220,1220,1201,1201,1157,1157,1150,1150,1144,1144,1140,1140,1130,1130,1128,1128,1127,1127,1124,1124,1118,1118,1108,1108,1083,1083,1078,1078,1077,1077,1072,1072,1070,1070,1066,1066,1055,1055,1054,1054,1053,1053,1047,1047,1046,1046,1043,1043,1042,1042,1037,1037,1030,1030,1020,1020,1018,1018,1012,1012,1008,1008,1006,1006,996,996,995,995,992,992,981,981,975,975,970,970,968,968,961,961,960,960,959,959,948,948,945,945,944,944,943,943,931,931,930,930,925,925,923,923,919,919,918,918,914,914,904,904,900,900,898,898,892,892,889,889,887,887,884,884,883,883,882,882,881,881,878,878,874,874,872,872,871,871,868,868,865,865,863,863,862,862,859,859,858,858,856,856,853,853,851,851,848,848,845,845,843,843,841,841,837,837,834,834,833,833,832,832,830,830,829,829,826,826,823,823,821,821,820,820,819,819,818,818,816,816,814,814,812,812,811,811,810,810,809,809,805,805,804,804,803,803,802,802,801,801,799,799,798,798,797,797,796,796,795,795,794,794,793,793,792,792,791,791,788,788,785,785,778,778,777,777,774,774,768,768,764,764,761,761,760,760,759,759,757,757,756,756,754,754,753,753,752,752,751,751,749,749,748,748,747,747,745,745,744,744,742,742,741,741,740,740,739,739,737,737,733,733,730,730,729,729,727,727,726,726,724,724,723,723,722,722,721,721,720,720,718,718,717,717,715,715,714,714,712,712,710,710,708,708,707,707,706,706,704,704,703,703,702,702,701,701,700,700,699,699,696,696,694,694,693,693,692,692,691,691,690,690,689,689,688,688,687,687,684,684,683,683,682,682,680,680,679,679,678,678,677,677,676,676,675,675,674,674,673,673,672,672,671,671,670,670,669,669,668,668,667,667,666,666,663,663,662,662,661,661,659,659,658,658,656,656,654,654,653,653,652,652,651,651,649,649,647,647,645,645,644,644,643,643,642,642,641,641,640,640,639,639,638,638,637,637,636,636,635,635,634,634,633,633,632,632,631,631,630,630,629,629,628,628,627,627,626,626,625,625,624,624,623,623,622,622,621,621,620,620,610,610,608,608,597,597,596,596,589,589,587,587,583,583,573,573,556,556,554,554,546,546,543,543,541,541,540,540,538,538,533,533,526,526,524,524,523,523,520,520,516,516,512,512,511,511,507,507,502,502,497,497,495,495,476,476,475,475,470,470,468,468,460,460,451,451,444,444,442,442,441,441,436,436,433,433,430,430,428,428,424,424,414,414,412,412,403,403,401,401,0.0],[332411,332411,332411,254571,254571,247717,247717,195466,195466,191156,191156,177765,177765,173612,173612,170239,170239,167106,167106,162871,162871,159923,159923,157524,157524,156399,156399,151375,151375,150363,150363,150230,150230,148339,148339,147170,147170,146100,146100,144023,144023,143007,143007,142465,142465,138341,138341,135668,135668,135080,135080,132702,132702,130898,130898,129441,129441,128572,128572,128070,128070,127938,127938,127273,127273,126977,126977,126334,126334,124655,124655,124528,124528,124112,124112,118504,118504,114846,114846,112261,112261,111956,111956,111544,111544,110988,110988,110374,110374,109303,109303,108924,108924,108909,108909,108095,108095,107066,107066,106883,106883,106435,106435,106381,106381,105942,105942,105561,105561,105314,105314,104707,104707,103299,103299,102203,102203,101640,101640,100927,100927,98942,98942,98585,98585,97453,97453,96365,96365,96300,96300,96121,96121,95867,95867,95777,95777,95285,95285,95087,95087,94131,94131,93585,93585,92562,92562,91919,91919,91600,91600,91574,91574,90991,90991,90730,90730,90283,90283,89642,89642,89107,89107,88857,88857,87693,87693,87392,87392,86692,86692,86347,86347,86295,86295,85924,85924,85704,85704,85066,85066,83926,83926,83817,83817,83546,83546,83117,83117,82365,82365,82258,82258,81871,81871,80401,80401,80366,80366,80274,80274,79744,79744,79739,79739,79287,79287,79219,79219,79146,79146,78966,78966,78351,78351,77263,77263,76932,76932,76501,76501,76344,76344,76128,76128,75532,75532,75422,75422,74880,74880,74834,74834,74339,74339,74269,74269,73728,73728,73462,73462,72750,72750,72204,72204,71895,71895,71573,71573,71226,71226,71223,71223,71156,71156,70768,70768,70651,70651,70049,70049,69795,69795,69775,69775,69718,69718,69696,69696,69540,69540,69083,69083,69060,69060,69050,69050,68924,68924,68611,68611,68435,68435,67470,67470,67449,67449,67390,67390,67136,67136,66989,66989,66730,66730,66721,66721,66639,66639,66473,66473,66338,66338,66015,66015,65892,65892,65785,65785,65488,65488,65181,65181,65108,65108,64909,64909,64218,64218,64185,64185,63935,63935,63304,63304,62514,62514,61362,61362,60953,60953,60524,60524,59767,59767,59529,59529,59450,59450,59375,59375,59366,59366,59205,59205,59082,59082,58983,58983,58593,58593,58454,58454,58023,58023,57884,57884,57768,57768,57558,57558,57415,57415,57410,57410,57403,57403,57149,57149,57058,57058,57022,57022,57010,57010,56967,56967,56963,56963,56865,56865,56583,56583,56477,56477,56452,56452,55889,55889,55472,55472,55402,55402,55353,55353,54988,54988,54970,54970,54880,54880,54346,54346,54292,54292,54270,54270,54046,54046,54038,54038,54024,54024,54022,54022,53953,53953,53683,53683,53350,53350,53337,53337,53294,53294,53245,53245,53156,53156,53042,53042,53038,53038,52948,52948,52925,52925,52516,52516,52209,52209,52192,52192,52110,52110,51856,51856,51412,51412,51365,51365,51312,51312,51113,51113,51086,51086,51045,51045,50814,50814,50685,50685,50329,50329,50314,50314,50307,50307,50277,50277,50149,50149,49613,49613,49135,49135,48820,48820,48594,48594,48587,48587,48516,48516,48408,48408,48332,48332,48273,48273,47951,47951,47700,47700,47659,47659,47610,47610,47456,47456,47298,47298,47228,47228,47125,47125,46987,46987,46922,46922,46897,46897,46896,46896,46621,46621,46556,46556,46483,46483,46359,46359,46146,46146,45900,45900,45808,45808,45699,45699,45652,45652,45621,45621,45512,45512,45465,45465,45281,45281,45075,45075,45033,45033,45024,45024,44836,44836,44811,44811,44617,44617,44593,44593,44533,44533,44487,44487,44405,44405,44333,44333,44224,44224,44220,44220,44079,44079,44066,44066,44064,44064,44058,44058,44040,44040,44020,44020,43806,43806,43778,43778,43734,43734,43516,43516,43361,43361,43304,43304,43142,43142,43034,43034,42955,42955,42946,42946,42935,42935,42756,42756,42682,42682,42671,42671,42427,42427,42375,42375,42308,42308,42279,42279,42160,42160,42156,42156,41750,41750,41644,41644,41439,41439,41398,41398,41393,41393,41295,41295,41022,41022,40917,40917,40906,40906,40849,40849,40835,40835,40754,40754,40734,40734,40649,40649,40544,40544,40373,40373,40222,40222,40069,40069,39873,39873,39736,39736,39679,39679,39661,39661,39582,39582,39357,39357,39348,39348,39146,39146,39060,39060,39009,39009,39000,39000,38842,38842,38823,38823,38767,38767,38756,38756,38530,38530,38285,38285,38167,38167,38162,38162,37999,37999,37800,37800,37758,37758,37608,37608,37538,37538,37415,37415,37273,37273,37117,37117,36925,36925,36725,36725,36677,36677,36542,36542,36415,36415,36242,36242,36135,36135,36077,36077,36027,36027,35985,35985,35965,35965,35905,35905,35644,35644,35608,35608,35458,35458,35394,35394,35207,35207,35019,35019,34916,34916,34848,34848,34843,34843,34771,34771,34686,34686,34596,34596,34582,34582,34449,34449,34365,34365,34028,34028,33864,33864,33784,33784,33690,33690,33574,33574,33538,33538,33498,33498,33287,33287,33218,33218,33133,33133,33098,33098,32979,32979,32938,32938,32810,32810,32745,32745,32740,32740,32739,32739,32681,32681,32669,32669,32455,32455,32403,32403,32399,32399,32220,32220,32212,32212,32190,32190,32088,32088,32055,32055,31856,31856,31754,31754,31701,31701,31657,31657,31475,31475,31378,31378,31230,31230,31205,31205,31196,31196,31167,31167,31029,31029,30936,30936,30868,30868,30852,30852,30653,30653,30516,30516,30402,30402,30308,30308,30300,30300,30272,30272,30059,30059,30040,30040,29962,29962,29908,29908,29888,29888,29835,29835,29803,29803,29780,29780,29730,29730,29697,29697,29646,29646,29624,29624,29617,29617,29563,29563,29435,29435,29266,29266,29184,29184,29122,29122,29078,29078,28991,28991,28861,28861,28814,28814,28787,28787,28778,28778,28763,28763,28745,28745,28740,28740,28666,28666,28664,28664,28660,28660,28651,28651,28629,28629,28490,28490,28467,28467,28461,28461,28345,28345,28202,28202,27925,27925,27838,27838,27783,27783,27581,27581,27514,27514,27439,27439,27360,27360,27344,27344,27325,27325,27308,27308,27306,27306,27250,27250,27240,27240,27079,27079,27057,27057,26884,26884,26637,26637,26623,26623,26614,26614,26538,26538,26518,26518,26440,26440,26327,26327,26310,26310,26289,26289,26284,26284,26216,26216,26188,26188,26144,26144,26036,26036,26034,26034,26008,26008,25906,25906,25879,25879,25857,25857,25850,25850,25837,25837,25801,25801,25701,25701,25600,25600,25565,25565,25557,25557,25462,25462,25438,25438,25355,25355,25165,25165,24983,24983,24951,24951,24805,24805,24641,24641,24563,24563,24551,24551,24459,24459,24348,24348,24236,24236,24200,24200,24192,24192,24024,24024,23979,23979,23957,23957,23848,23848,23488,23488,23484,23484,23462,23462,23390,23390,23190,23190,23187,23187,23035,23035,22954,22954,22801,22801,22783,22783,22722,22722,22668,22668,22665,22665,22560,22560,22497,22497,22408,22408,22279,22279,22201,22201,22191,22191,22180,22180,22120,22120,22075,22075,22026,22026,22002,22002,21999,21999,21895,21895,21859,21859,21801,21801,21760,21760,21727,21727,21407,21407,21396,21396,21367,21367,21328,21328,21275,21275,21219,21219,21161,21161,21133,21133,21071,21071,21025,21025,21020,21020,20870,20870,20853,20853,20849,20849,20824,20824,20804,20804,20693,20693,20676,20676,20670,20670,20618,20618,20490,20490,20456,20456,20409,20409,20319,20319,20292,20292,20274,20274,20201,20201,20160,20160,20116,20116,19930,19930,19891,19891,19807,19807,19791,19791,19785,19785,19727,19727,19671,19671,19596,19596,19526,19526,19429,19429,19417,19417,19327,19327,19257,19257,19253,19253,19210,19210,19133,19133,19100,19100,19097,19097,19062,19062,19048,19048,19046,19046,19008,19008,18995,18995,18930,18930,18890,18890,18744,18744,18733,18733,18717,18717,18594,18594,18540,18540,18494,18494,18492,18492,18302,18302,18249,18249,18224,18224,18123,18123,18116,18116,18113,18113,18062,18062,18061,18061,18051,18051,18000,18000,17995,17995,17869,17869,17836,17836,17741,17741,17677,17677,17676,17676,17619,17619,17596,17596,17591,17591,17587,17587,17549,17549,17526,17526,17514,17514,17513,17513,17504,17504,17499,17499,17442,17442,17431,17431,17342,17342,17272,17272,17199,17199,17145,17145,17116,17116,17064,17064,17025,17025,17018,17018,16942,16942,16821,16821,16720,16720,16717,16717,16708,16708,16700,16700,16672,16672,16669,16669,16532,16532,16451,16451,16448,16448,16357,16357,16300,16300,16216,16216,16213,16213,16191,16191,16026,16026,16017,16017,15976,15976,15916,15916,15892,15892,15867,15867,15802,15802,15755,15755,15738,15738,15693,15693,15645,15645,15426,15426,15260,15260,15136,15136,15059,15059,14980,14980,14914,14914,14908,14908,14829,14829,14769,14769,14765,14765,14654,14654,14648,14648,14580,14580,14557,14557,14543,14543,14496,14496,14373,14373,14309,14309,14278,14278,14182,14182,14125,14125,14101,14101,14043,14043,14026,14026,13934,13934,13858,13858,13851,13851,13706,13706,13698,13698,13615,13615,13585,13585,13563,13563,13540,13540,13396,13396,13318,13318,13315,13315,13302,13302,13291,13291,13273,13273,13177,13177,13134,13134,13112,13112,13110,13110,13076,13076,13064,13064,13045,13045,13042,13042,13014,13014,12973,12973,12958,12958,12920,12920,12888,12888,12862,12862,12829,12829,12804,12804,12756,12756,12578,12578,12564,12564,12450,12450,12393,12393,12348,12348,12315,12315,12285,12285,12236,12236,12201,12201,12167,12167,12146,12146,12117,12117,12116,12116,12072,12072,11901,11901,11885,11885,11879,11879,11716,11716,11679,11679,11625,11625,11597,11597,11592,11592,11550,11550,11474,11474,11435,11435,11432,11432,11361,11361,11285,11285,11176,11176,11133,11133,11088,11088,11033,11033,10963,10963,10961,10961,10922,10922,10853,10853,10832,10832,10760,10760,10742,10742,10720,10720,10707,10707,10706,10706,10577,10577,10568,10568,10547,10547,10399,10399,10360,10360,10357,10357,10259,10259,10256,10256,10159,10159,10146,10146,10088,10088,10047,10047,10009,10009,9873,9873,9859,9859,9849,9849,9820,9820,9639,9639,9630,9630,9546,9546,9522,9522,9390,9390,9357,9357,9336,9336,9320,9320,9319,9319,9247,9247,9203,9203,9147,9147,9109,9109,9054,9054,9012,9012,8972,8972,8949,8949,8925,8925,8869,8869,8807,8807,8793,8793,8785,8785,8699,8699,8697,8697,8682,8682,8606,8606,8560,8560,8524,8524,8490,8490,8434,8434,8389,8389,8340,8340,8281,8281,8194,8194,8097,8097,8047,8047,8004,8004,7982,7982,7962,7962,7928,7928,7890,7890,7863,7863,7813,7813,7727,7727,7708,7708,7678,7678,7649,7649,7556,7556,7540,7540,7485,7485,7449,7449,7414,7414,7397,7397,7358,7358,7006,7006,6990,6990,6987,6987,6984,6984,6958,6958,6848,6848,6792,6792,6742,6742,6618,6618,6605,6605,6591,6591,6574,6574,6571,6571,6536,6536,6486,6486,6441,6441,6400,6400,6372,6372,6361,6361,6358,6358,6274,6274,6183,6183,6138,6138,6136,6136,6028,6028,5983,5983,5960,5960,5888,5888,5852,5852,5818,5818,5693,5693,5649,5649,5618,5618,5607,5607,5601,5601,5557,5557,5515,5515,5512,5512,5490,5490,5476,5476,5440,5440,5435,5435,5411,5411,5341,5341,5298,5298,5282,5282,5270,5270,5268,5268,5261,5261,5258,5258,5247,5247,5233,5233,5217,5217,5216,5216,5215,5215,5173,5173,5142,5142,5134,5134,5081,5081,5055,5055,5044,5044,5027,5027,4994,4994,4936,4936,4923,4923,4903,4903,4891,4891,4878,4878,4797,4797,4788,4788,4782,4782,4768,4768,4760,4760,4663,4663,4654,4654,4639,4639,4605,4605,4530,4530,4472,4472,4455,4455,4444,4444,4364,4364,4350,4350,4332,4332,4319,4319,4318,4318,4210,4210,4208,4208,4160,4160,4113,4113,4089,4089,4087,4087,4061,4061,4006,4006,4002,4002,3991,3991,3970,3970,3937,3937,3837,3837,3835,3835,3830,3830,3782,3782,3757,3757,3710,3710,3706,3706,3696,3696,3693,3693,3669,3669,3566,3566,3549,3549,3500,3500,3492,3492,3445,3445,3408,3408,3295,3295,3288,3288,3255,3255,3123,3123,3067,3067,2851,2851,2835,2835,2796,2796,2727,2727,2639,2639,2625,2625,2622,2622,2601,2601,2563,2563,2547,2547,2494,2494,2492,2492,2490,2490,2471,2471,2418,2418,2411,2411,2390,2390,2361,2361,2349,2349,2318,2318,2316,2316,2297,2297,2246,2246,2146,2146,2141,2141,2097,2097,2067,2067,2046,2046,1984,1984,1933,1933,1914,1914,1903,1903,1883,1883,1881,1881,1857,1857,1849,1849,1848,1848,1846,1846,1833,1833,1771,1771,1755,1755,1746,1746,1744,1744,1715,1715,1709,1709,1608,1608,1602,1602,1597,1597,1562,1562,1547,1547,1499,1499,1441,1441,1436,1436,1431,1431,1392,1392,1383,1383,1378,1378,1350,1350,1315,1315,1311,1311,1304,1304,1291,1291,1287,1287,1281,1281,1278,1278,1269,1269,1261,1261,1235,1235,1226,1226,1223,1223,1217,1217,1216,1216,1207,1207,1202,1202,1197,1197,1192,1192,1187,1187,1184,1184,1180,1180,1175,1175,1174,1174,1168,1168,1154,1154,1147,1147,1137,1137,1133,1133,1124,1124,1112,1112,1110,1110,1096,1096,1093,1093,1089,1089,1083,1083,1082,1082,1077,1077,1061,1061,1052,1052,1050,1050,1048,1048,1046,1046,1021,1021,1015,1015,1013,1013,1010,1010,1007,1007,1005,1005,1002,1002,997,997,994,994,993,993,991,991,990,990,987,987,984,984,981,981,978,978,973,973,970,970,969,969,968,968,967,967,966,966,964,964,962,962,961,961,960,960,954,954,952,952,950,950,944,944,943,943,939,939,936,936,935,935,931,931,928,928,926,926,924,924,923,923,921,921,918,918,916,916,910,910,905,905,904,904,902,902,900,900,897,897,892,892,891,891,889,889,888,888,887,887,885,885,883,883,882,882,881,881,879,879,877,877,876,876,874,874,873,873,871,871,870,870,869,869,868,868,867,867,866,866,864,864,863,863,858,858,857,857,856,856,854,854,853,853,851,851,850,850,849,849,844,844,842,842,839,839,835,835,834,834,833,833,832,832,828,828,827,827,824,824,823,823,818,818,816,816,814,814,812,812,811,811,809,809,808,808,806,806,805,805,804,804,803,803,802,802,801,801,800,800,798,798,797,797,796,796,795,795,793,793,791,791,789,789,788,788,787,787,786,786,785,785,784,784,783,783,780,780,778,778,777,777,776,776,774,774,773,773,772,772,771,771,770,770,769,769,768,768,767,767,765,765,764,764,763,763,762,762,761,761,760,760,759,759,758,758,757,757,756,756,755,755,753,753,752,752,751,751,750,750,749,749,747,747,746,746,745,745,743,743,742,742,741,741,740,740,739,739,738,738,737,737,736,736,735,735,734,734,733,733,732,732,731,731,730,730,729,729,728,728,724,724,723,723,721,721,720,720,719,719,718,718,717,717,716,716,715,715,714,714,713,713,711,711,710,710,708,708,707,707,706,706,705,705,704,704,703,703,702,702,701,701,700,700,697,697,696,696,695,695,694,694,692,692,691,691,690,690,689,689,688,688,687,687,686,686,685,685,684,684,683,683,682,682,681,681,680,680,678,678,677,677,676,676,675,675,674,674,673,673,672,672,671,671,670,670,669,669,668,668,667,667,666,666,665,665,663,663,662,662,661,661,660,660,659,659,658,658,657,657,656,656,654,654,653,653,652,652,651,651,650,650,649,649,648,648,647,647,645,645,644,644,643,643,642,642,641,641,640,640,639,639,638,638,637,637,636,636,635,635,634,634,633,633,632,632,631,631,630,630,629,629,628,628,627,627,626,626,625,625,624,624,623,623,622,622,621,621,620,620,608,608,604,604,565,565,554,554,538,538,520,520,514,514,512,512,468,468,451,451,447,447,441,441,416,416,413,413,0.0],[332073,332073,332073,233031,233031,209587,209587,190801,190801,176308,176308,158793,158793,157299,157299,155777,155777,151900,151900,142495,142495,137015,137015,134959,134959,132268,132268,130436,130436,127449,127449,124586,124586,124011,124011,123469,123469,120167,120167,120111,120111,119202,119202,117690,117690,117189,117189,115898,115898,111288,111288,110988,110988,110896,110896,109608,109608,108861,108861,107781,107781,106613,106613,106243,106243,106064,106064,105513,105513,105109,105109,104346,104346,103203,103203,101981,101981,100755,100755,100055,100055,97302,97302,95640,95640,93477,93477,92934,92934,91932,91932,91840,91840,90984,90984,90792,90792,89368,89368,89270,89270,88981,88981,88679,88679,87826,87826,87771,87771,87266,87266,86691,86691,86223,86223,85662,85662,85070,85070,84399,84399,84042,84042,83771,83771,83766,83766,82071,82071,81849,81849,81596,81596,80454,80454,79808,79808,79661,79661,78999,78999,77236,77236,76862,76862,75841,75841,75239,75239,75235,75235,75001,75001,74914,74914,73001,73001,72815,72815,72748,72748,71801,71801,71584,71584,70824,70824,70735,70735,69974,69974,69955,69955,69786,69786,69617,69617,69412,69412,69393,69393,69243,69243,68988,68988,68922,68922,67395,67395,67312,67312,67249,67249,67045,67045,66897,66897,66589,66589,66469,66469,65902,65902,65780,65780,65621,65621,65574,65574,65259,65259,65108,65108,65044,65044,64185,64185,64100,64100,63989,63989,63946,63946,63714,63714,62593,62593,62556,62556,62361,62361,61983,61983,61485,61485,61218,61218,61209,61209,60841,60841,60522,60522,60511,60511,59637,59637,59590,59590,59504,59504,59448,59448,59300,59300,59271,59271,59216,59216,59078,59078,58816,58816,58598,58598,58443,58443,58263,58263,58119,58119,58060,58060,57590,57590,57497,57497,57361,57361,57306,57306,57203,57203,56838,56838,56397,56397,56384,56384,56237,56237,56124,56124,55999,55999,55638,55638,55370,55370,55233,55233,55070,55070,54944,54944,54924,54924,54866,54866,54792,54792,54685,54685,54346,54346,54168,54168,53867,53867,53831,53831,53258,53258,53102,53102,53037,53037,52888,52888,52844,52844,52825,52825,52792,52792,52781,52781,52735,52735,52613,52613,52595,52595,52391,52391,52387,52387,52357,52357,52215,52215,52109,52109,52082,52082,51884,51884,51622,51622,51293,51293,51156,51156,50849,50849,50558,50558,50451,50451,50369,50369,50250,50250,50133,50133,50063,50063,49954,49954,49946,49946,49898,49898,49535,49535,49491,49491,49486,49486,49254,49254,49073,49073,48968,48968,48960,48960,48487,48487,48373,48373,48350,48350,48285,48285,48269,48269,48226,48226,48203,48203,48160,48160,48087,48087,47901,47901,47884,47884,47712,47712,47623,47623,47421,47421,47407,47407,47377,47377,47316,47316,47211,47211,47163,47163,47152,47152,47139,47139,47103,47103,46821,46821,46793,46793,46491,46491,46418,46418,46292,46292,46236,46236,45747,45747,45722,45722,45608,45608,45351,45351,45344,45344,45221,45221,45209,45209,45181,45181,45100,45100,45045,45045,44964,44964,44732,44732,44587,44587,44479,44479,44374,44374,44280,44280,44207,44207,44147,44147,44034,44034,44024,44024,43942,43942,43888,43888,43806,43806,43749,43749,43490,43490,43357,43357,43132,43132,43104,43104,42894,42894,42862,42862,42363,42363,42154,42154,41982,41982,41923,41923,41737,41737,41680,41680,41573,41573,41473,41473,41390,41390,41328,41328,41296,41296,41159,41159,41143,41143,40780,40780,40754,40754,40735,40735,40714,40714,40672,40672,40540,40540,40331,40331,40298,40298,40284,40284,40268,40268,40245,40245,40201,40201,40065,40065,40045,40045,39954,39954,39931,39931,39737,39737,39527,39527,39415,39415,39222,39222,39190,39190,39118,39118,39068,39068,39028,39028,38937,38937,38798,38798,38653,38653,38614,38614,38464,38464,38432,38432,38377,38377,38255,38255,38252,38252,38240,38240,37965,37965,37711,37711,37703,37703,37691,37691,37596,37596,37547,37547,37452,37452,37202,37202,37134,37134,37090,37090,37058,37058,36891,36891,36683,36683,36607,36607,36481,36481,36401,36401,36399,36399,36320,36320,36202,36202,36100,36100,35935,35935,35903,35903,35694,35694,35528,35528,35497,35497,35459,35459,35450,35450,35318,35318,35298,35298,35289,35289,34831,34831,34814,34814,34699,34699,34692,34692,34630,34630,34528,34528,34523,34523,34427,34427,34336,34336,34305,34305,34228,34228,34200,34200,34185,34185,33769,33769,33765,33765,33756,33756,33725,33725,33709,33709,33703,33703,33653,33653,33603,33603,33580,33580,33561,33561,33536,33536,33521,33521,33423,33423,33398,33398,33328,33328,33286,33286,33145,33145,33105,33105,33102,33102,33081,33081,32937,32937,32927,32927,32810,32810,32796,32796,32700,32700,32677,32677,32625,32625,32585,32585,32551,32551,32294,32294,32270,32270,32263,32263,32218,32218,32211,32211,32209,32209,32127,32127,32093,32093,31985,31985,31979,31979,31766,31766,31746,31746,31576,31576,31531,31531,31497,31497,31475,31475,31458,31458,31405,31405,31235,31235,31129,31129,31068,31068,31067,31067,31054,31054,30902,30902,30896,30896,30879,30879,30724,30724,30711,30711,30706,30706,30684,30684,30604,30604,30521,30521,30513,30513,30465,30465,30439,30439,30228,30228,30205,30205,30154,30154,30143,30143,30030,30030,29978,29978,29916,29916,29904,29904,29900,29900,29798,29798,29769,29769,29532,29532,29506,29506,29413,29413,29198,29198,29124,29124,29101,29101,29005,29005,28954,28954,28893,28893,28812,28812,28804,28804,28793,28793,28788,28788,28761,28761,28747,28747,28734,28734,28733,28733,28715,28715,28668,28668,28625,28625,28403,28403,28327,28327,28318,28318,28267,28267,28229,28229,27976,27976,27869,27869,27856,27856,27739,27739,27735,27735,27730,27730,27677,27677,27571,27571,27554,27554,27544,27544,27515,27515,27443,27443,27320,27320,27301,27301,27272,27272,27261,27261,27240,27240,27114,27114,27071,27071,26994,26994,26963,26963,26927,26927,26888,26888,26881,26881,26874,26874,26851,26851,26770,26770,26767,26767,26580,26580,26556,26556,26522,26522,26460,26460,26444,26444,26430,26430,26390,26390,26354,26354,26346,26346,26344,26344,26306,26306,26278,26278,26261,26261,26227,26227,26170,26170,26158,26158,26116,26116,26067,26067,26043,26043,25916,25916,25912,25912,25850,25850,25811,25811,25777,25777,25769,25769,25728,25728,25683,25683,25648,25648,25577,25577,25513,25513,25477,25477,25469,25469,25427,25427,25412,25412,25345,25345,25300,25300,25253,25253,25158,25158,25134,25134,25086,25086,25000,25000,24860,24860,24803,24803,24789,24789,24770,24770,24744,24744,24641,24641,24576,24576,24548,24548,24485,24485,24427,24427,24426,24426,24314,24314,24310,24310,24297,24297,24290,24290,24263,24263,24200,24200,24194,24194,24111,24111,24040,24040,23981,23981,23848,23848,23836,23836,23744,23744,23735,23735,23713,23713,23710,23710,23673,23673,23491,23491,23442,23442,23383,23383,23293,23293,23209,23209,23079,23079,23052,23052,23040,23040,23036,23036,23034,23034,23020,23020,22999,22999,22926,22926,22813,22813,22788,22788,22772,22772,22712,22712,22640,22640,22615,22615,22575,22575,22569,22569,22554,22554,22491,22491,22464,22464,22405,22405,22384,22384,22206,22206,22187,22187,22103,22103,22012,22012,22007,22007,21974,21974,21891,21891,21821,21821,21809,21809,21778,21778,21746,21746,21733,21733,21659,21659,21656,21656,21588,21588,21549,21549,21535,21535,21511,21511,21501,21501,21419,21419,21388,21388,21379,21379,21337,21337,21330,21330,21310,21310,21187,21187,21128,21128,21107,21107,20996,20996,20973,20973,20916,20916,20893,20893,20884,20884,20865,20865,20822,20822,20816,20816,20767,20767,20723,20723,20678,20678,20659,20659,20611,20611,20563,20563,20553,20553,20541,20541,20524,20524,20523,20523,20433,20433,20411,20411,20317,20317,20303,20303,20297,20297,20258,20258,20236,20236,20228,20228,20226,20226,20218,20218,20162,20162,20063,20063,20054,20054,20051,20051,20037,20037,19996,19996,19989,19989,19904,19904,19889,19889,19884,19884,19853,19853,19824,19824,19766,19766,19763,19763,19718,19718,19691,19691,19679,19679,19664,19664,19658,19658,19635,19635,19584,19584,19497,19497,19465,19465,19457,19457,19433,19433,19416,19416,19359,19359,19330,19330,19258,19258,19242,19242,19233,19233,19221,19221,19132,19132,19056,19056,19020,19020,19012,19012,18990,18990,18968,18968,18923,18923,18918,18918,18843,18843,18831,18831,18790,18790,18784,18784,18777,18777,18710,18710,18702,18702,18698,18698,18669,18669,18645,18645,18612,18612,18591,18591,18585,18585,18583,18583,18551,18551,18531,18531,18526,18526,18499,18499,18490,18490,18476,18476,18458,18458,18446,18446,18420,18420,18413,18413,18295,18295,18274,18274,18203,18203,18189,18189,18171,18171,18159,18159,18157,18157,18156,18156,18145,18145,18100,18100,18079,18079,17978,17978,17951,17951,17842,17842,17835,17835,17799,17799,17794,17794,17773,17773,17767,17767,17752,17752,17723,17723,17693,17693,17627,17627,17599,17599,17514,17514,17492,17492,17466,17466,17448,17448,17434,17434,17365,17365,17305,17305,17270,17270,17251,17251,17226,17226,17196,17196,17166,17166,17156,17156,17091,17091,17083,17083,17030,17030,17022,17022,17018,17018,16965,16965,16963,16963,16928,16928,16917,16917,16890,16890,16882,16882,16853,16853,16817,16817,16806,16806,16789,16789,16752,16752,16738,16738,16712,16712,16711,16711,16709,16709,16699,16699,16639,16639,16630,16630,16574,16574,16556,16556,16539,16539,16482,16482,16393,16393,16355,16355,16343,16343,16294,16294,16270,16270,16260,16260,16218,16218,16184,16184,16158,16158,16153,16153,16148,16148,16062,16062,16059,16059,16036,16036,16022,16022,15999,15999,15950,15950,15875,15875,15872,15872,15826,15826,15801,15801,15797,15797,15769,15769,15756,15756,15732,15732,15720,15720,15667,15667,15613,15613,15611,15611,15557,15557,15547,15547,15539,15539,15518,15518,15357,15357,15356,15356,15134,15134,15080,15080,15057,15057,15037,15037,14991,14991,14989,14989,14977,14977,14975,14975,14963,14963,14890,14890,14832,14832,14765,14765,14687,14687,14572,14572,14571,14571,14553,14553,14448,14448,14360,14360,14343,14343,14269,14269,14263,14263,14251,14251,14181,14181,14047,14047,14034,14034,13922,13922,13902,13902,13901,13901,13891,13891,13867,13867,13857,13857,13814,13814,13800,13800,13791,13791,13780,13780,13747,13747,13746,13746,13733,13733,13709,13709,13657,13657,13639,13639,13570,13570,13538,13538,13523,13523,13501,13501,13477,13477,13474,13474,13431,13431,13412,13412,13385,13385,13338,13338,13303,13303,13301,13301,13263,13263,13245,13245,13242,13242,13233,13233,13177,13177,13175,13175,13167,13167,13107,13107,13072,13072,13060,13060,13054,13054,13052,13052,12998,12998,12990,12990,12982,12982,12955,12955,12928,12928,12850,12850,12829,12829,12825,12825,12824,12824,12800,12800,12775,12775,12770,12770,12754,12754,12676,12676,12629,12629,12608,12608,12566,12566,12562,12562,12545,12545,12522,12522,12496,12496,12416,12416,12345,12345,12302,12302,12292,12292,12282,12282,12279,12279,12262,12262,12253,12253,12214,12214,12211,12211,12175,12175,12151,12151,12135,12135,12095,12095,12087,12087,12009,12009,12006,12006,11910,11910,11909,11909,11900,11900,11895,11895,11884,11884,11870,11870,11788,11788,11763,11763,11757,11757,11725,11725,11714,11714,11706,11706,11668,11668,11658,11658,11621,11621,11602,11602,11592,11592,11515,11515,11510,11510,11500,11500,11494,11494,11467,11467,11413,11413,11393,11393,11370,11370,11356,11356,11294,11294,11221,11221,11220,11220,11168,11168,11160,11160,11110,11110,11059,11059,11047,11047,11023,11023,11020,11020,11019,11019,10987,10987,10958,10958,10863,10863,10849,10849,10831,10831,10814,10814,10807,10807,10794,10794,10709,10709,10699,10699,10681,10681,10645,10645,10642,10642,10629,10629,10605,10605,10565,10565,10521,10521,10508,10508,10489,10489,10430,10430,10414,10414,10373,10373,10368,10368,10367,10367,10366,10366,10330,10330,10270,10270,10263,10263,10254,10254,10235,10235,10189,10189,10185,10185,10145,10145,10138,10138,10127,10127,10088,10088,10078,10078,10063,10063,10058,10058,10047,10047,10034,10034,9980,9980,9970,9970,9968,9968,9954,9954,9944,9944,9939,9939,9844,9844,9824,9824,9793,9793,9751,9751,9735,9735,9730,9730,9724,9724,9606,9606,9514,9514,9468,9468,9425,9425,9361,9361,9343,9343,9308,9308,9254,9254,9238,9238,9235,9235,9233,9233,9232,9232,9172,9172,9136,9136,9123,9123,9100,9100,9097,9097,9090,9090,9086,9086,9084,9084,9063,9063,9035,9035,8941,8941,8936,8936,8907,8907,8898,8898,8890,8890,8869,8869,8865,8865,8805,8805,8798,8798,8788,8788,8782,8782,8775,8775,8753,8753,8730,8730,8720,8720,8702,8702,8695,8695,8674,8674,8665,8665,8654,8654,8653,8653,8629,8629,8612,8612,8607,8607,8578,8578,8569,8569,8555,8555,8539,8539,8529,8529,8525,8525,8495,8495,8467,8467,8460,8460,8457,8457,8402,8402,8396,8396,8380,8380,8358,8358,8308,8308,8277,8277,8265,8265,8236,8236,8222,8222,8193,8193,8188,8188,8182,8182,8175,8175,8164,8164,8155,8155,8136,8136,8108,8108,8105,8105,8086,8086,8052,8052,8019,8019,8012,8012,7999,7999,7984,7984,7944,7944,7914,7914,7900,7900,7882,7882,7855,7855,7849,7849,7840,7840,7827,7827,7805,7805,7804,7804,7801,7801,7773,7773,7761,7761,7738,7738,7733,7733,7708,7708,7684,7684,7649,7649,7636,7636,7619,7619,7605,7605,7592,7592,7585,7585,7577,7577,7567,7567,7546,7546,7540,7540,7518,7518,7498,7498,7487,7487,7485,7485,7477,7477,7469,7469,7449,7449,7435,7435,7413,7413,7392,7392,7391,7391,7373,7373,7363,7363,7362,7362,7351,7351,7316,7316,7314,7314,7312,7312,7310,7310,7306,7306,7300,7300,7299,7299,7292,7292,7250,7250,7191,7191,7187,7187,7177,7177,7166,7166,7162,7162,7132,7132,7124,7124,7118,7118,7109,7109,7049,7049,7037,7037,7031,7031,7025,7025,6983,6983,6965,6965,6954,6954,6952,6952,6946,6946,6945,6945,6938,6938,6869,6869,6830,6830,6813,6813,6809,6809,6800,6800,6708,6708,6697,6697,6670,6670,6659,6659,6643,6643,6615,6615,6542,6542,6533,6533,6518,6518,6508,6508,6487,6487,6485,6485,6463,6463,6460,6460,6451,6451,6388,6388,6386,6386,6371,6371,6369,6369,6361,6361,6267,6267,6262,6262,6259,6259,6225,6225,6221,6221,6217,6217,6216,6216,6211,6211,6184,6184,6129,6129,6115,6115,6113,6113,6111,6111,6088,6088,6083,6083,6078,6078,6056,6056,6050,6050,6049,6049,6037,6037,6034,6034,6028,6028,6002,6002,5965,5965,5960,5960,5946,5946,5942,5942,5928,5928,5903,5903,5881,5881,5840,5840,5837,5837,5814,5814,5811,5811,5794,5794,5779,5779,5775,5775,5750,5750,5729,5729,5685,5685,5676,5676,5674,5674,5572,5572,5560,5560,5539,5539,5494,5494,5487,5487,5485,5485,5469,5469,5442,5442,5427,5427,5423,5423,5403,5403,5372,5372,5364,5364,5362,5362,5354,5354,5321,5321,5303,5303,5302,5302,5290,5290,5284,5284,5265,5265,5255,5255,5246,5246,5225,5225,5220,5220,5209,5209,5194,5194,5185,5185,5159,5159,5154,5154,5126,5126,5107,5107,5102,5102,5092,5092,5072,5072,5068,5068,5062,5062,5054,5054,5050,5050,5012,5012,5001,5001,4973,4973,4960,4960,4957,4957,4885,4885,4878,4878,4876,4876,4873,4873,4850,4850,4845,4845,4842,4842,4803,4803,4786,4786,4785,4785,4775,4775,4745,4745,4715,4715,4714,4714,4711,4711,4625,4625,4590,4590,4551,4551,4547,4547,4544,4544,4542,4542,4539,4539,4536,4536,4524,4524,4497,4497,4489,4489,4475,4475,4420,4420,4396,4396,4386,4386,4384,4384,4356,4356,4343,4343,4337,4337,4335,4335,4275,4275,4268,4268,4233,4233,4171,4171,4164,4164,4138,4138,4132,4132,4123,4123,4085,4085,4084,4084,4071,4071,4049,4049,4039,4039,4035,4035,4022,4022,3999,3999,3998,3998,3957,3957,3939,3939,3916,3916,3901,3901,3851,3851,3841,3841,3839,3839,3737,3737,3735,3735,3718,3718,3704,3704,3696,3696,3691,3691,3671,3671,3654,3654,3640,3640,3612,3612,3604,3604,3602,3602,3599,3599,3596,3596,3589,3589,3583,3583,3578,3578,3575,3575,3509,3509,3487,3487,3445,3445,3438,3438,3415,3415,3391,3391,3370,3370,3359,3359,3347,3347,3333,3333,3324,3324,3289,3289,3275,3275,3263,3263,3252,3252,3229,3229,3227,3227,3215,3215,3206,3206,3198,3198,3197,3197,3196,3196,3194,3194,3181,3181,3151,3151,3141,3141,3089,3089,3080,3080,3071,3071,3053,3053,3038,3038,3025,3025,3017,3017,3005,3005,3003,3003,2988,2988,2951,2951,2942,2942,2909,2909,2905,2905,2903,2903,2887,2887,2879,2879,2878,2878,2875,2875,2867,2867,2847,2847,2836,2836,2833,2833,2816,2816,2808,2808,2806,2806,2796,2796,2794,2794,2789,2789,2766,2766,2752,2752,2730,2730,2729,2729,2699,2699,2691,2691,2683,2683,2642,2642,2640,2640,2603,2603,2601,2601,2598,2598,2588,2588,2576,2576,2575,2575,2564,2564,2559,2559,2557,2557,2555,2555,2537,2537,2501,2501,2491,2491,2469,2469,2455,2455,2410,2410,2382,2382,2371,2371,2369,2369,2365,2365,2360,2360,2356,2356,2325,2325,2289,2289,2282,2282,2254,2254,2253,2253,2248,2248,2247,2247,2246,2246,2241,2241,2233,2233,2192,2192,2184,2184,2148,2148,2146,2146,2138,2138,2085,2085,2079,2079,2071,2071,2070,2070,2058,2058,2056,2056,2047,2047,2046,2046,1973,1973,1967,1967,1963,1963,1962,1962,1944,1944,1942,1942,1914,1914,1908,1908,1890,1890,1881,1881,1876,1876,1860,1860,1853,1853,1846,1846,1833,1833,1799,1799,1788,1788,1773,1773,1765,1765,1742,1742,1723,1723,1717,1717,1709,1709,1698,1698,1696,1696,1671,1671,1669,1669,1611,1611,1609,1609,1604,1604,1597,1597,1593,1593,1571,1571,1565,1565,1564,1564,1556,1556,1536,1536,1533,1533,1494,1494,1476,1476,1468,1468,1459,1459,1450,1450,1445,1445,1444,1444,1442,1442,1411,1411,1408,1408,1399,1399,1378,1378,1370,1370,1347,1347,1337,1337,1328,1328,1327,1327,1309,1309,1307,1307,1297,1297,1292,1292,1283,1283,1278,1278,1269,1269,1254,1254,1245,1245,1237,1237,1223,1223,1215,1215,1186,1186,1178,1178,1165,1165,1150,1150,1147,1147,1121,1121,1120,1120,1116,1116,1103,1103,1086,1086,1083,1083,1078,1078,1059,1059,1050,1050,1037,1037,1029,1029,1008,1008,1007,1007,993,993,988,988,987,987,986,986,984,984,983,983,982,982,972,972,968,968,958,958,956,956,953,953,946,946,944,944,943,943,929,929,928,928,924,924,919,919,915,915,908,908,905,905,899,899,898,898,889,889,874,874,873,873,872,872,871,871,869,869,868,868,865,865,860,860,855,855,853,853,851,851,847,847,841,841,838,838,837,837,835,835,833,833,830,830,828,828,823,823,820,820,819,819,814,814,810,810,809,809,808,808,804,804,802,802,801,801,799,799,797,797,796,796,794,794,793,793,791,791,785,785,784,784,782,782,781,781,777,777,775,775,774,774,771,771,770,770,768,768,765,765,762,762,761,761,759,759,757,757,756,756,754,754,752,752,751,751,750,750,748,748,745,745,744,744,742,742,739,739,738,738,733,733,730,730,729,729,727,727,726,726,724,724,723,723,722,722,721,721,720,720,718,718,717,717,715,715,711,711,709,709,702,702,700,700,698,698,696,696,693,693,692,692,691,691,690,690,689,689,688,688,687,687,684,684,682,682,680,680,679,679,678,678,676,676,675,675,674,674,673,673,672,672,671,671,669,669,663,663,662,662,661,661,659,659,658,658,656,656,655,655,654,654,653,653,652,652,651,651,649,649,647,647,646,646,644,644,643,643,642,642,641,641,640,640,639,639,637,637,636,636,635,635,634,634,633,633,632,632,631,631,630,630,629,629,628,628,627,627,626,626,624,624,623,623,622,622,621,621,620,620,595,595,587,587,575,575,569,569,565,565,564,564,555,555,551,551,550,550,549,549,542,542,538,538,533,533,516,516,510,510,476,476,475,475,462,462,451,451,448,448,442,442,436,436,414,414,409,409,404,404,403,403,0.0],[332073,332073,332073,233031,233031,209587,209587,190801,190801,176308,176308,158793,158793,157299,157299,155777,155777,151900,151900,142495,142495,137015,137015,134959,134959,132268,132268,130436,130436,127449,127449,124586,124586,124011,124011,123469,123469,120167,120167,120111,120111,119202,119202,117690,117690,117189,117189,115898,115898,111288,111288,110988,110988,110896,110896,109608,109608,108861,108861,107781,107781,106613,106613,106243,106243,106064,106064,105513,105513,105109,105109,104346,104346,103203,103203,101981,101981,100755,100755,100055,100055,97302,97302,95640,95640,93477,93477,92934,92934,91932,91932,91840,91840,90984,90984,90792,90792,89368,89368,89270,89270,88981,88981,88679,88679,87826,87826,87771,87771,87266,87266,86691,86691,86223,86223,85662,85662,85070,85070,84399,84399,84042,84042,83771,83771,83766,83766,82071,82071,81849,81849,81596,81596,80454,80454,79808,79808,79661,79661,78999,78999,77236,77236,76862,76862,75841,75841,75239,75239,75235,75235,75001,75001,74914,74914,73001,73001,72815,72815,72748,72748,71801,71801,71584,71584,70824,70824,70735,70735,69974,69974,69955,69955,69786,69786,69617,69617,69412,69412,69393,69393,69243,69243,68988,68988,68922,68922,67395,67395,67312,67312,67249,67249,67045,67045,66897,66897,66589,66589,66469,66469,65902,65902,65780,65780,65621,65621,65574,65574,65259,65259,65108,65108,65044,65044,64185,64185,64100,64100,63989,63989,63946,63946,63714,63714,62593,62593,62556,62556,62361,62361,61983,61983,61485,61485,61218,61218,61209,61209,60522,60522,60511,60511,59637,59637,59590,59590,59504,59504,59448,59448,59300,59300,59271,59271,59216,59216,59078,59078,58816,58816,58598,58598,58443,58443,58263,58263,58119,58119,58060,58060,57590,57590,57497,57497,57361,57361,57306,57306,57203,57203,56838,56838,56397,56397,56384,56384,56237,56237,56124,56124,55999,55999,55638,55638,55370,55370,55233,55233,55070,55070,54944,54944,54924,54924,54866,54866,54792,54792,54685,54685,54346,54346,54168,54168,53867,53867,53831,53831,53258,53258,53102,53102,53037,53037,52888,52888,52844,52844,52825,52825,52792,52792,52781,52781,52735,52735,52613,52613,52595,52595,52391,52391,52387,52387,52357,52357,52215,52215,52109,52109,52082,52082,51884,51884,51622,51622,51293,51293,51156,51156,50849,50849,50558,50558,50451,50451,50369,50369,50250,50250,50133,50133,50063,50063,49954,49954,49946,49946,49898,49898,49535,49535,49491,49491,49486,49486,49254,49254,49073,49073,48968,48968,48960,48960,48487,48487,48373,48373,48350,48350,48285,48285,48269,48269,48226,48226,48203,48203,48160,48160,48087,48087,47901,47901,47884,47884,47712,47712,47623,47623,47421,47421,47407,47407,47377,47377,47316,47316,47211,47211,47163,47163,47152,47152,47139,47139,47103,47103,46821,46821,46793,46793,46491,46491,46418,46418,46292,46292,46236,46236,45747,45747,45722,45722,45608,45608,45351,45351,45344,45344,45221,45221,45209,45209,45181,45181,45100,45100,45045,45045,44964,44964,44732,44732,44587,44587,44479,44479,44374,44374,44280,44280,44207,44207,44147,44147,44034,44034,44024,44024,43942,43942,43888,43888,43806,43806,43749,43749,43490,43490,43357,43357,43319,43319,43132,43132,43104,43104,42894,42894,42862,42862,42363,42363,42206,42206,42154,42154,41982,41982,41923,41923,41737,41737,41680,41680,41573,41573,41473,41473,41390,41390,41328,41328,41296,41296,41159,41159,41143,41143,40817,40817,40780,40780,40754,40754,40735,40735,40714,40714,40672,40672,40540,40540,40331,40331,40298,40298,40284,40284,40268,40268,40245,40245,40201,40201,40065,40065,40045,40045,39954,39954,39931,39931,39737,39737,39527,39527,39415,39415,39222,39222,39190,39190,39118,39118,39068,39068,39028,39028,38937,38937,38798,38798,38653,38653,38614,38614,38464,38464,38432,38432,38377,38377,38255,38255,38252,38252,38240,38240,37965,37965,37711,37711,37703,37703,37691,37691,37596,37596,37547,37547,37452,37452,37202,37202,37134,37134,37090,37090,37058,37058,36891,36891,36683,36683,36607,36607,36481,36481,36401,36401,36399,36399,36320,36320,36202,36202,36100,36100,35935,35935,35903,35903,35694,35694,35528,35528,35497,35497,35459,35459,35450,35450,35318,35318,35298,35298,35289,35289,34831,34831,34814,34814,34699,34699,34692,34692,34630,34630,34528,34528,34523,34523,34427,34427,34336,34336,34305,34305,34228,34228,34200,34200,34185,34185,34103,34103,33769,33769,33765,33765,33756,33756,33725,33725,33709,33709,33703,33703,33603,33603,33580,33580,33561,33561,33536,33536,33521,33521,33423,33423,33328,33328,33286,33286,33145,33145,33105,33105,33102,33102,33081,33081,32937,32937,32927,32927,32810,32810,32796,32796,32700,32700,32677,32677,32625,32625,32585,32585,32551,32551,32294,32294,32270,32270,32263,32263,32218,32218,32211,32211,32209,32209,32127,32127,32093,32093,31985,31985,31979,31979,31766,31766,31746,31746,31576,31576,31531,31531,31497,31497,31475,31475,31458,31458,31405,31405,31235,31235,31129,31129,31068,31068,31067,31067,31054,31054,30902,30902,30896,30896,30879,30879,30724,30724,30711,30711,30706,30706,30684,30684,30604,30604,30521,30521,30513,30513,30465,30465,30439,30439,30228,30228,30205,30205,30154,30154,30030,30030,29978,29978,29916,29916,29904,29904,29900,29900,29798,29798,29769,29769,29532,29532,29506,29506,29413,29413,29198,29198,29124,29124,29101,29101,29005,29005,28954,28954,28893,28893,28812,28812,28804,28804,28793,28793,28788,28788,28761,28761,28747,28747,28734,28734,28733,28733,28715,28715,28668,28668,28625,28625,28403,28403,28327,28327,28318,28318,28267,28267,28229,28229,27976,27976,27869,27869,27856,27856,27739,27739,27735,27735,27730,27730,27677,27677,27571,27571,27554,27554,27544,27544,27515,27515,27443,27443,27320,27320,27301,27301,27272,27272,27261,27261,27240,27240,27114,27114,27071,27071,26994,26994,26963,26963,26927,26927,26888,26888,26881,26881,26874,26874,26851,26851,26770,26770,26767,26767,26580,26580,26556,26556,26522,26522,26460,26460,26444,26444,26430,26430,26390,26390,26354,26354,26346,26346,26344,26344,26306,26306,26278,26278,26261,26261,26227,26227,26170,26170,26158,26158,26116,26116,26067,26067,26043,26043,25916,25916,25912,25912,25850,25850,25811,25811,25777,25777,25769,25769,25728,25728,25683,25683,25648,25648,25577,25577,25513,25513,25477,25477,25469,25469,25427,25427,25412,25412,25345,25345,25300,25300,25253,25253,25158,25158,25134,25134,25086,25086,25000,25000,24860,24860,24803,24803,24789,24789,24770,24770,24744,24744,24641,24641,24576,24576,24548,24548,24485,24485,24427,24427,24426,24426,24314,24314,24310,24310,24297,24297,24290,24290,24263,24263,24200,24200,24194,24194,24111,24111,24040,24040,23848,23848,23836,23836,23744,23744,23735,23735,23713,23713,23710,23710,23673,23673,23491,23491,23442,23442,23383,23383,23293,23293,23215,23215,23209,23209,23079,23079,23052,23052,23040,23040,23036,23036,23034,23034,23020,23020,22926,22926,22813,22813,22788,22788,22772,22772,22712,22712,22640,22640,22615,22615,22575,22575,22569,22569,22554,22554,22491,22491,22464,22464,22405,22405,22384,22384,22236,22236,22206,22206,22187,22187,22103,22103,22012,22012,22007,22007,21974,21974,21891,21891,21821,21821,21778,21778,21746,21746,21733,21733,21659,21659,21656,21656,21588,21588,21549,21549,21535,21535,21511,21511,21501,21501,21419,21419,21379,21379,21337,21337,21277,21277,21187,21187,21128,21128,21107,21107,20996,20996,20916,20916,20914,20914,20893,20893,20884,20884,20865,20865,20822,20822,20816,20816,20767,20767,20723,20723,20678,20678,20659,20659,20611,20611,20563,20563,20553,20553,20541,20541,20524,20524,20523,20523,20433,20433,20411,20411,20317,20317,20303,20303,20297,20297,20258,20258,20236,20236,20228,20228,20226,20226,20162,20162,20063,20063,20054,20054,20051,20051,20037,20037,19996,19996,19989,19989,19904,19904,19889,19889,19884,19884,19853,19853,19824,19824,19766,19766,19763,19763,19691,19691,19679,19679,19664,19664,19658,19658,19635,19635,19584,19584,19497,19497,19465,19465,19457,19457,19433,19433,19416,19416,19359,19359,19330,19330,19258,19258,19242,19242,19233,19233,19221,19221,19132,19132,19056,19056,19020,19020,19012,19012,18990,18990,18968,18968,18923,18923,18918,18918,18843,18843,18831,18831,18790,18790,18784,18784,18777,18777,18726,18726,18710,18710,18702,18702,18698,18698,18645,18645,18612,18612,18591,18591,18585,18585,18551,18551,18531,18531,18526,18526,18490,18490,18476,18476,18446,18446,18420,18420,18413,18413,18295,18295,18203,18203,18189,18189,18171,18171,18159,18159,18157,18157,18156,18156,18145,18145,18100,18100,17978,17978,17951,17951,17842,17842,17835,17835,17799,17799,17794,17794,17773,17773,17767,17767,17752,17752,17723,17723,17693,17693,17627,17627,17599,17599,17514,17514,17492,17492,17482,17482,17466,17466,17448,17448,17401,17401,17365,17365,17329,17329,17305,17305,17270,17270,17251,17251,17226,17226,17196,17196,17166,17166,17156,17156,17091,17091,17083,17083,17030,17030,17022,17022,16998,16998,16963,16963,16928,16928,16917,16917,16890,16890,16882,16882,16853,16853,16817,16817,16752,16752,16738,16738,16735,16735,16712,16712,16711,16711,16709,16709,16699,16699,16639,16639,16630,16630,16574,16574,16539,16539,16482,16482,16393,16393,16355,16355,16343,16343,16294,16294,16270,16270,16260,16260,16218,16218,16184,16184,16158,16158,16153,16153,16148,16148,16062,16062,16059,16059,16036,16036,16022,16022,15999,15999,15950,15950,15875,15875,15872,15872,15826,15826,15801,15801,15797,15797,15769,15769,15756,15756,15732,15732,15720,15720,15667,15667,15611,15611,15557,15557,15547,15547,15539,15539,15441,15441,15357,15357,15134,15134,15080,15080,15057,15057,15037,15037,14991,14991,14989,14989,14977,14977,14975,14975,14890,14890,14832,14832,14765,14765,14687,14687,14629,14629,14572,14572,14571,14571,14553,14553,14448,14448,14360,14360,14343,14343,14269,14269,14263,14263,14251,14251,14181,14181,14047,14047,14034,14034,13922,13922,13902,13902,13901,13901,13891,13891,13867,13867,13857,13857,13814,13814,13800,13800,13791,13791,13780,13780,13747,13747,13746,13746,13733,13733,13709,13709,13657,13657,13639,13639,13570,13570,13538,13538,13523,13523,13501,13501,13477,13477,13474,13474,13431,13431,13412,13412,13385,13385,13338,13338,13303,13303,13301,13301,13263,13263,13245,13245,13242,13242,13233,13233,13177,13177,13175,13175,13167,13167,13107,13107,13054,13054,13052,13052,12990,12990,12982,12982,12928,12928,12850,12850,12829,12829,12825,12825,12824,12824,12800,12800,12791,12791,12775,12775,12770,12770,12754,12754,12676,12676,12629,12629,12608,12608,12566,12566,12562,12562,12545,12545,12522,12522,12416,12416,12345,12345,12302,12302,12292,12292,12282,12282,12279,12279,12262,12262,12253,12253,12214,12214,12211,12211,12151,12151,12135,12135,12095,12095,12087,12087,12044,12044,12009,12009,12006,12006,11910,11910,11909,11909,11900,11900,11895,11895,11884,11884,11870,11870,11788,11788,11763,11763,11757,11757,11725,11725,11714,11714,11668,11668,11658,11658,11621,11621,11602,11602,11592,11592,11515,11515,11500,11500,11494,11494,11467,11467,11413,11413,11393,11393,11370,11370,11356,11356,11294,11294,11279,11279,11221,11221,11215,11215,11168,11168,11160,11160,11110,11110,11047,11047,11023,11023,11020,11020,11019,11019,10987,10987,10958,10958,10863,10863,10849,10849,10831,10831,10814,10814,10807,10807,10794,10794,10709,10709,10699,10699,10681,10681,10645,10645,10642,10642,10629,10629,10605,10605,10565,10565,10521,10521,10489,10489,10414,10414,10373,10373,10368,10368,10367,10367,10366,10366,10330,10330,10270,10270,10263,10263,10254,10254,10189,10189,10185,10185,10145,10145,10138,10138,10127,10127,10088,10088,10078,10078,10063,10063,10047,10047,10034,10034,9980,9980,9970,9970,9968,9968,9954,9954,9939,9939,9793,9793,9751,9751,9735,9735,9730,9730,9724,9724,9626,9626,9606,9606,9514,9514,9468,9468,9425,9425,9361,9361,9343,9343,9308,9308,9254,9254,9238,9238,9235,9235,9233,9233,9232,9232,9219,9219,9172,9172,9136,9136,9123,9123,9100,9100,9097,9097,9090,9090,9084,9084,9063,9063,9035,9035,8941,8941,8936,8936,8898,8898,8890,8890,8869,8869,8865,8865,8805,8805,8798,8798,8788,8788,8753,8753,8730,8730,8720,8720,8702,8702,8695,8695,8687,8687,8674,8674,8654,8654,8653,8653,8629,8629,8607,8607,8578,8578,8569,8569,8555,8555,8539,8539,8529,8529,8525,8525,8495,8495,8467,8467,8402,8402,8396,8396,8380,8380,8358,8358,8339,8339,8327,8327,8277,8277,8265,8265,8236,8236,8193,8193,8188,8188,8182,8182,8175,8175,8155,8155,8136,8136,8108,8108,8052,8052,8019,8019,8012,8012,7999,7999,7984,7984,7944,7944,7914,7914,7900,7900,7882,7882,7840,7840,7804,7804,7773,7773,7761,7761,7708,7708,7684,7684,7649,7649,7636,7636,7619,7619,7605,7605,7585,7585,7577,7577,7567,7567,7546,7546,7540,7540,7518,7518,7487,7487,7485,7485,7477,7477,7449,7449,7435,7435,7413,7413,7373,7373,7363,7363,7362,7362,7316,7316,7312,7312,7310,7310,7306,7306,7300,7300,7299,7299,7292,7292,7190,7190,7187,7187,7166,7166,7162,7162,7124,7124,7118,7118,7037,7037,7031,7031,7028,7028,6965,6965,6954,6954,6946,6946,6945,6945,6938,6938,6835,6835,6813,6813,6809,6809,6747,6747,6708,6708,6697,6697,6676,6676,6670,6670,6659,6659,6643,6643,6615,6615,6591,6591,6542,6542,6533,6533,6519,6519,6518,6518,6502,6502,6485,6485,6471,6471,6460,6460,6388,6388,6369,6369,6361,6361,6267,6267,6259,6259,6225,6225,6217,6217,6216,6216,6211,6211,6146,6146,6129,6129,6115,6115,6113,6113,6088,6088,6071,6071,6056,6056,6050,6050,6037,6037,6035,6035,6007,6007,6002,6002,5946,5946,5928,5928,5903,5903,5840,5840,5838,5838,5811,5811,5794,5794,5750,5750,5729,5729,5685,5685,5676,5676,5674,5674,5603,5603,5560,5560,5539,5539,5526,5526,5495,5495,5494,5494,5487,5487,5469,5469,5449,5449,5442,5442,5427,5427,5423,5423,5403,5403,5362,5362,5354,5354,5350,5350,5303,5303,5302,5302,5290,5290,5284,5284,5265,5265,5256,5256,5246,5246,5232,5232,5225,5225,5209,5209,5194,5194,5185,5185,5159,5159,5107,5107,5092,5092,5075,5075,5072,5072,5068,5068,5062,5062,5001,5001,4964,4964,4957,4957,4885,4885,4878,4878,4876,4876,4845,4845,4801,4801,4764,4764,4711,4711,4689,4689,4677,4677,4625,4625,4590,4590,4547,4547,4542,4542,4497,4497,4494,4494,4489,4489,4420,4420,4356,4356,4343,4343,4337,4337,4335,4335,4317,4317,4265,4265,4233,4233,4220,4220,4171,4171,4164,4164,4132,4132,4123,4123,4106,4106,4071,4071,4056,4056,4049,4049,4035,4035,3957,3957,3939,3939,3933,3933,3916,3916,3851,3851,3841,3841,3822,3822,3716,3716,3704,3704,3696,3696,3691,3691,3671,3671,3640,3640,3636,3636,3619,3619,3612,3612,3604,3604,3602,3602,3596,3596,3583,3583,3578,3578,3575,3575,3573,3573,3509,3509,3487,3487,3445,3445,3438,3438,3423,3423,3403,3403,3382,3382,3354,3354,3333,3333,3289,3289,3275,3275,3229,3229,3206,3206,3198,3198,3197,3197,3194,3194,3159,3159,3091,3091,3089,3089,3066,3066,3038,3038,3025,3025,3020,3020,3017,3017,3005,3005,2982,2982,2942,2942,2925,2925,2905,2905,2887,2887,2879,2879,2867,2867,2796,2796,2794,2794,2782,2782,2770,2770,2766,2766,2730,2730,2691,2691,2614,2614,2603,2603,2602,2602,2601,2601,2598,2598,2564,2564,2555,2555,2537,2537,2521,2521,2509,2509,2500,2500,2497,2497,2492,2492,2472,2472,2455,2455,2444,2444,2365,2365,2356,2356,2336,2336,2314,2314,2272,2272,2262,2262,2250,2250,2233,2233,2219,2219,2148,2148,2096,2096,2085,2085,2071,2071,1967,1967,1942,1942,1940,1940,1936,1936,1911,1911,1890,1890,1881,1881,1879,1879,1860,1860,1853,1853,1835,1835,1820,1820,1800,1800,1799,1799,1788,1788,1745,1745,1723,1723,1669,1669,1645,1645,1622,1622,1605,1605,1564,1564,1551,1551,1546,1546,1525,1525,1508,1508,1504,1504,1501,1501,1465,1465,1450,1450,1442,1442,1423,1423,1416,1416,1399,1399,1378,1378,1363,1363,1346,1346,1343,1343,1309,1309,1278,1278,1266,1266,1237,1237,1226,1226,1191,1191,1187,1187,1178,1178,1159,1159,1154,1154,1137,1137,1129,1129,1121,1121,1120,1120,1112,1112,1093,1093,1083,1083,1078,1078,1065,1065,1064,1064,1020,1020,1012,1012,1007,1007,994,994,987,987,984,984,982,982,981,981,971,971,956,956,948,948,940,940,939,939,928,928,925,925,922,922,919,919,916,916,914,914,913,913,908,908,905,905,902,902,901,901,899,899,889,889,888,888,887,887,872,872,870,870,865,865,864,864,862,862,861,861,860,860,859,859,855,855,851,851,845,845,842,842,841,841,831,831,828,828,822,822,821,821,818,818,815,815,814,814,810,810,809,809,808,808,804,804,802,802,798,798,796,796,794,794,793,793,792,792,791,791,788,788,786,786,785,785,782,782,777,777,775,775,774,774,770,770,769,769,768,768,767,767,766,766,762,762,761,761,760,760,759,759,758,758,755,755,754,754,752,752,750,750,745,745,742,742,741,741,740,740,739,739,735,735,734,734,731,731,730,730,729,729,728,728,727,727,726,726,721,721,720,720,719,719,718,718,716,716,715,715,711,711,709,709,707,707,706,706,705,705,704,704,702,702,701,701,700,700,697,697,695,695,693,693,692,692,691,691,687,687,685,685,682,682,681,681,680,680,679,679,678,678,675,675,674,674,673,673,672,672,671,671,670,670,669,669,668,668,667,667,665,665,664,664,663,663,662,662,660,660,659,659,658,658,656,656,654,654,652,652,651,651,650,650,649,649,648,648,647,647,646,646,643,643,642,642,640,640,639,639,637,637,636,636,635,635,634,634,633,633,632,632,630,630,629,629,628,628,627,627,626,626,624,624,623,623,622,622,621,621,620,620,614,614,607,607,595,595,591,591,558,558,550,550,531,531,514,514,505,505,457,457,451,451,425,425,415,415,403,403,0.0],[182161,182161,182161,173781,173781,166288,166288,137195,137195,129502,129502,128942,128942,126397,126397,124595,124595,122639,122639,120826,120826,117290,117290,115783,115783,115118,115118,111468,111468,110595,110595,109213,109213,107780,107780,106715,106715,106302,106302,106041,106041,105405,105405,102551,102551,101902,101902,101315,101315,101024,101024,100814,100814,97711,97711,95789,95789,94288,94288,93156,93156,91626,91626,91420,91420,90833,90833,90568,90568,87929,87929,86649,86649,86431,86431,85574,85574,84148,84148,83861,83861,83503,83503,81850,81850,81363,81363,80602,80602,79788,79788,77912,77912,77226,77226,77126,77126,76931,76931,76050,76050,75659,75659,75060,75060,74437,74437,73799,73799,73438,73438,72956,72956,72145,72145,71849,71849,71375,71375,71222,71222,71169,71169,71099,71099,70847,70847,70774,70774,69988,69988,69815,69815,69813,69813,69714,69714,69653,69653,69594,69594,69347,69347,68963,68963,68803,68803,68156,68156,67292,67292,66892,66892,66679,66679,66674,66674,65905,65905,65810,65810,65526,65526,65480,65480,65297,65297,65228,65228,64846,64846,64813,64813,64562,64562,64363,64363,64007,64007,63828,63828,63780,63780,63472,63472,63314,63314,62737,62737,62636,62636,62490,62490,62419,62419,62129,62129,61428,61428,61391,61391,61290,61290,61105,61105,59624,59624,59520,59520,59422,59422,59409,59409,58751,58751,58719,58719,58073,58073,57746,57746,57370,57370,57205,57205,57091,57091,56988,56988,56496,56496,55669,55669,55610,55610,55606,55606,55577,55577,55446,55446,55271,55271,55237,55237,54966,54966,54759,54759,54720,54720,54386,54386,54272,54272,54186,54186,54109,54109,53900,53900,53889,53889,53886,53886,53872,53872,53843,53843,53429,53429,53397,53397,53248,53248,53198,53198,53025,53025,53019,53019,52931,52931,52884,52884,52862,52862,52566,52566,52487,52487,52333,52333,52310,52310,52037,52037,51854,51854,51629,51629,51473,51473,51466,51466,51383,51383,51140,51140,51084,51084,51069,51069,51054,51054,50453,50453,50180,50180,50030,50030,49704,49704,49640,49640,49590,49590,49460,49460,49234,49234,49183,49183,49043,49043,48911,48911,48836,48836,48835,48835,48613,48613,48580,48580,48477,48477,48261,48261,47775,47775,47760,47760,47656,47656,47579,47579,47410,47410,47390,47390,47119,47119,47103,47103,47011,47011,46693,46693,46615,46615,46331,46331,46183,46183,45948,45948,45928,45928,45832,45832,45814,45814,45755,45755,45720,45720,45321,45321,45294,45294,45217,45217,45213,45213,45160,45160,45117,45117,45018,45018,44912,44912,44900,44900,44846,44846,44805,44805,44743,44743,44730,44730,44694,44694,44689,44689,44606,44606,44564,44564,44403,44403,44376,44376,44347,44347,44294,44294,44241,44241,44180,44180,44013,44013,43984,43984,43972,43972,43928,43928,43837,43837,43527,43527,43272,43272,43211,43211,43207,43207,43163,43163,43071,43071,42967,42967,42911,42911,42602,42602,42594,42594,42495,42495,42479,42479,42257,42257,42156,42156,42037,42037,42023,42023,41965,41965,41959,41959,41823,41823,41698,41698,41642,41642,41565,41565,41519,41519,41484,41484,41331,41331,41246,41246,41167,41167,41014,41014,40928,40928,40846,40846,40838,40838,40794,40794,40650,40650,40634,40634,40479,40479,40445,40445,40394,40394,40272,40272,40231,40231,40162,40162,39893,39893,39670,39670,39669,39669,39619,39619,39590,39590,39532,39532,39515,39515,39415,39415,39309,39309,39254,39254,38980,38980,38972,38972,38951,38951,38877,38877,38832,38832,38819,38819,38754,38754,38681,38681,38673,38673,38644,38644,38465,38465,38412,38412,38357,38357,38215,38215,38203,38203,38126,38126,38019,38019,37990,37990,37813,37813,37773,37773,37594,37594,37575,37575,37518,37518,37514,37514,37496,37496,37324,37324,37188,37188,37157,37157,37152,37152,37115,37115,36959,36959,36950,36950,36875,36875,36699,36699,36616,36616,36600,36600,36558,36558,36547,36547,36511,36511,36502,36502,36431,36431,36270,36270,36255,36255,36151,36151,36109,36109,35977,35977,35736,35736,35676,35676,35535,35535,35483,35483,35482,35482,35457,35457,35427,35427,35289,35289,35254,35254,35174,35174,34991,34991,34880,34880,34842,34842,34634,34634,34621,34621,34436,34436,34398,34398,34343,34343,34293,34293,34196,34196,34185,34185,34035,34035,34025,34025,34012,34012,34005,34005,33860,33860,33819,33819,33690,33690,33593,33593,33580,33580,33526,33526,33499,33499,33464,33464,33451,33451,33413,33413,33359,33359,33287,33287,33283,33283,33266,33266,33246,33246,33219,33219,33213,33213,33146,33146,33128,33128,32855,32855,32826,32826,32787,32787,32740,32740,32719,32719,32705,32705,32597,32597,32576,32576,32408,32408,32363,32363,32342,32342,32330,32330,32301,32301,32233,32233,32211,32211,32153,32153,32134,32134,32094,32094,32093,32093,32091,32091,32015,32015,31976,31976,31957,31957,31951,31951,31832,31832,31771,31771,31687,31687,31375,31375,31366,31366,31274,31274,31208,31208,31203,31203,31137,31137,31112,31112,31032,31032,31007,31007,30930,30930,30868,30868,30720,30720,30632,30632,30492,30492,30450,30450,30418,30418,30412,30412,30379,30379,30362,30362,30322,30322,30311,30311,30231,30231,30173,30173,30163,30163,30126,30126,30123,30123,30062,30062,30012,30012,29989,29989,29981,29981,29850,29850,29845,29845,29736,29736,29730,29730,29673,29673,29575,29575,29442,29442,29303,29303,29290,29290,29275,29275,29265,29265,29243,29243,29236,29236,29186,29186,29184,29184,29162,29162,29154,29154,29103,29103,29062,29062,29057,29057,29050,29050,29024,29024,28922,28922,28846,28846,28825,28825,28807,28807,28781,28781,28762,28762,28716,28716,28629,28629,28622,28622,28620,28620,28610,28610,28547,28547,28519,28519,28435,28435,28334,28334,28218,28218,28150,28150,28081,28081,28005,28005,27989,27989,27985,27985,27940,27940,27893,27893,27892,27892,27850,27850,27843,27843,27780,27780,27778,27778,27730,27730,27709,27709,27492,27492,27418,27418,27372,27372,27354,27354,27347,27347,27346,27346,27338,27338,27264,27264,27227,27227,27222,27222,27087,27087,27081,27081,26973,26973,26938,26938,26925,26925,26902,26902,26857,26857,26850,26850,26792,26792,26779,26779,26721,26721,26712,26712,26701,26701,26656,26656,26555,26555,26538,26538,26534,26534,26511,26511,26510,26510,26492,26492,26387,26387,26383,26383,26337,26337,26329,26329,26259,26259,26247,26247,26198,26198,26126,26126,26095,26095,26047,26047,25993,25993,25988,25988,25984,25984,25976,25976,25951,25951,25901,25901,25736,25736,25708,25708,25696,25696,25678,25678,25660,25660,25590,25590,25552,25552,25498,25498,25487,25487,25472,25472,25469,25469,25401,25401,25367,25367,25328,25328,25220,25220,25181,25181,25134,25134,25010,25010,24988,24988,24873,24873,24863,24863,24800,24800,24783,24783,24782,24782,24755,24755,24750,24750,24655,24655,24548,24548,24496,24496,24453,24453,24438,24438,24402,24402,24287,24287,24286,24286,24258,24258,24239,24239,24218,24218,24209,24209,24087,24087,24053,24053,23961,23961,23918,23918,23827,23827,23825,23825,23799,23799,23692,23692,23601,23601,23563,23563,23527,23527,23509,23509,23503,23503,23459,23459,23457,23457,23423,23423,23404,23404,23402,23402,23391,23391,23298,23298,23292,23292,23262,23262,23215,23215,23205,23205,23188,23188,23185,23185,23178,23178,23157,23157,23097,23097,22936,22936,22857,22857,22821,22821,22769,22769,22748,22748,22731,22731,22714,22714,22658,22658,22516,22516,22440,22440,22427,22427,22347,22347,22333,22333,22315,22315,22304,22304,22276,22276,22201,22201,22198,22198,22145,22145,22132,22132,22048,22048,22037,22037,22036,22036,22035,22035,22027,22027,22015,22015,21995,21995,21959,21959,21952,21952,21935,21935,21804,21804,21652,21652,21627,21627,21609,21609,21584,21584,21522,21522,21505,21505,21490,21490,21446,21446,21440,21440,21414,21414,21355,21355,21258,21258,21219,21219,21216,21216,21210,21210,21115,21115,21099,21099,21048,21048,21003,21003,20980,20980,20947,20947,20930,20930,20928,20928,20893,20893,20869,20869,20833,20833,20824,20824,20744,20744,20727,20727,20713,20713,20693,20693,20653,20653,20648,20648,20625,20625,20605,20605,20586,20586,20545,20545,20522,20522,20434,20434,20431,20431,20421,20421,20414,20414,20394,20394,20389,20389,20386,20386,20377,20377,20350,20350,20348,20348,20303,20303,20302,20302,20280,20280,20273,20273,20266,20266,20253,20253,20200,20200,20176,20176,20141,20141,20139,20139,20069,20069,20054,20054,20025,20025,20023,20023,20015,20015,19984,19984,19977,19977,19940,19940,19927,19927,19866,19866,19849,19849,19805,19805,19779,19779,19772,19772,19753,19753,19737,19737,19705,19705,19654,19654,19645,19645,19626,19626,19546,19546,19530,19530,19495,19495,19340,19340,19337,19337,19312,19312,19259,19259,19215,19215,19196,19196,19195,19195,19187,19187,19178,19178,19167,19167,19166,19166,19151,19151,19114,19114,19068,19068,19064,19064,19041,19041,19020,19020,19012,19012,19003,19003,18999,18999,18997,18997,18992,18992,18989,18989,18935,18935,18925,18925,18877,18877,18865,18865,18851,18851,18794,18794,18714,18714,18704,18704,18653,18653,18609,18609,18543,18543,18524,18524,18517,18517,18514,18514,18511,18511,18474,18474,18456,18456,18443,18443,18342,18342,18291,18291,18182,18182,18177,18177,18166,18166,18107,18107,18089,18089,18052,18052,18017,18017,17996,17996,17995,17995,17918,17918,17872,17872,17859,17859,17845,17845,17834,17834,17816,17816,17803,17803,17798,17798,17776,17776,17752,17752,17746,17746,17723,17723,17720,17720,17717,17717,17701,17701,17640,17640,17639,17639,17630,17630,17614,17614,17610,17610,17577,17577,17573,17573,17571,17571,17532,17532,17525,17525,17486,17486,17387,17387,17379,17379,17364,17364,17286,17286,17224,17224,17214,17214,17188,17188,17171,17171,17168,17168,17167,17167,17160,17160,17110,17110,17054,17054,17043,17043,17032,17032,16937,16937,16918,16918,16912,16912,16869,16869,16857,16857,16853,16853,16852,16852,16844,16844,16841,16841,16839,16839,16818,16818,16775,16775,16761,16761,16751,16751,16726,16726,16720,16720,16691,16691,16661,16661,16623,16623,16587,16587,16577,16577,16576,16576,16499,16499,16479,16479,16455,16455,16408,16408,16345,16345,16317,16317,16283,16283,16254,16254,16249,16249,16232,16232,16227,16227,16212,16212,16205,16205,16095,16095,16041,16041,15999,15999,15967,15967,15965,15965,15927,15927,15857,15857,15846,15846,15844,15844,15809,15809,15779,15779,15768,15768,15742,15742,15738,15738,15734,15734,15693,15693,15679,15679,15659,15659,15636,15636,15559,15559,15557,15557,15556,15556,15540,15540,15534,15534,15463,15463,15456,15456,15354,15354,15336,15336,15303,15303,15301,15301,15288,15288,15199,15199,15127,15127,15064,15064,15052,15052,15038,15038,14986,14986,14927,14927,14886,14886,14881,14881,14866,14866,14861,14861,14832,14832,14809,14809,14803,14803,14799,14799,14759,14759,14707,14707,14704,14704,14698,14698,14678,14678,14664,14664,14595,14595,14571,14571,14548,14548,14530,14530,14525,14525,14455,14455,14451,14451,14429,14429,14405,14405,14392,14392,14384,14384,14337,14337,14316,14316,14306,14306,14302,14302,14281,14281,14263,14263,14256,14256,14241,14241,14240,14240,14217,14217,14172,14172,14118,14118,14107,14107,14085,14085,13928,13928,13910,13910,13886,13886,13864,13864,13853,13853,13789,13789,13754,13754,13732,13732,13703,13703,13694,13694,13685,13685,13674,13674,13655,13655,13650,13650,13568,13568,13507,13507,13489,13489,13487,13487,13469,13469,13464,13464,13399,13399,13384,13384,13382,13382,13317,13317,13313,13313,13279,13279,13263,13263,13252,13252,13233,13233,13174,13174,13162,13162,13158,13158,13142,13142,13117,13117,13109,13109,13091,13091,13079,13079,13061,13061,13035,13035,13021,13021,13020,13020,13008,13008,12961,12961,12938,12938,12928,12928,12883,12883,12873,12873,12858,12858,12795,12795,12779,12779,12769,12769,12754,12754,12702,12702,12670,12670,12650,12650,12648,12648,12647,12647,12604,12604,12587,12587,12580,12580,12540,12540,12532,12532,12516,12516,12417,12417,12404,12404,12385,12385,12370,12370,12356,12356,12307,12307,12300,12300,12246,12246,12243,12243,12241,12241,12215,12215,12212,12212,12195,12195,12189,12189,12168,12168,12158,12158,12141,12141,12120,12120,12109,12109,12089,12089,12084,12084,12075,12075,12041,12041,12029,12029,12015,12015,11990,11990,11948,11948,11935,11935,11918,11918,11910,11910,11891,11891,11882,11882,11857,11857,11841,11841,11831,11831,11816,11816,11791,11791,11781,11781,11685,11685,11676,11676,11660,11660,11647,11647,11595,11595,11592,11592,11585,11585,11562,11562,11558,11558,11547,11547,11545,11545,11517,11517,11472,11472,11452,11452,11438,11438,11434,11434,11423,11423,11396,11396,11332,11332,11315,11315,11289,11289,11265,11265,11264,11264,11257,11257,11206,11206,11158,11158,11097,11097,11080,11080,11074,11074,11002,11002,10993,10993,10986,10986,10949,10949,10941,10941,10940,10940,10921,10921,10874,10874,10841,10841,10779,10779,10765,10765,10740,10740,10718,10718,10708,10708,10688,10688,10672,10672,10669,10669,10664,10664,10658,10658,10622,10622,10621,10621,10596,10596,10591,10591,10577,10577,10565,10565,10522,10522,10509,10509,10469,10469,10468,10468,10429,10429,10422,10422,10419,10419,10389,10389,10384,10384,10380,10380,10353,10353,10334,10334,10285,10285,10264,10264,10252,10252,10213,10213,10174,10174,10148,10148,10131,10131,10127,10127,10117,10117,10101,10101,10085,10085,10082,10082,10064,10064,10029,10029,10013,10013,10011,10011,9996,9996,9977,9977,9955,9955,9930,9930,9912,9912,9911,9911,9895,9895,9882,9882,9876,9876,9870,9870,9838,9838,9835,9835,9834,9834,9769,9769,9760,9760,9757,9757,9725,9725,9690,9690,9688,9688,9667,9667,9660,9660,9625,9625,9573,9573,9565,9565,9553,9553,9544,9544,9538,9538,9537,9537,9509,9509,9503,9503,9489,9489,9480,9480,9444,9444,9432,9432,9426,9426,9402,9402,9392,9392,9382,9382,9381,9381,9380,9380,9353,9353,9327,9327,9308,9308,9306,9306,9292,9292,9279,9279,9276,9276,9247,9247,9237,9237,9220,9220,9188,9188,9142,9142,9117,9117,9113,9113,9110,9110,9084,9084,9073,9073,9061,9061,8986,8986,8981,8981,8976,8976,8970,8970,8942,8942,8894,8894,8867,8867,8859,8859,8853,8853,8822,8822,8784,8784,8735,8735,8723,8723,8712,8712,8701,8701,8680,8680,8654,8654,8594,8594,8587,8587,8582,8582,8575,8575,8572,8572,8524,8524,8513,8513,8496,8496,8483,8483,8476,8476,8461,8461,8457,8457,8454,8454,8446,8446,8441,8441,8437,8437,8427,8427,8412,8412,8391,8391,8389,8389,8381,8381,8370,8370,8350,8350,8348,8348,8347,8347,8280,8280,8263,8263,8249,8249,8212,8212,8207,8207,8199,8199,8083,8083,8070,8070,8064,8064,8063,8063,8048,8048,8019,8019,8014,8014,8004,8004,7992,7992,7979,7979,7977,7977,7944,7944,7940,7940,7933,7933,7929,7929,7927,7927,7922,7922,7893,7893,7883,7883,7844,7844,7834,7834,7830,7830,7821,7821,7811,7811,7773,7773,7754,7754,7753,7753,7734,7734,7728,7728,7719,7719,7705,7705,7691,7691,7666,7666,7664,7664,7631,7631,7577,7577,7566,7566,7565,7565,7564,7564,7563,7563,7525,7525,7516,7516,7515,7515,7506,7506,7501,7501,7490,7490,7479,7479,7448,7448,7446,7446,7442,7442,7414,7414,7394,7394,7381,7381,7376,7376,7373,7373,7338,7338,7322,7322,7304,7304,7286,7286,7270,7270,7263,7263,7213,7213,7210,7210,7206,7206,7195,7195,7193,7193,7179,7179,7128,7128,7119,7119,7082,7082,7072,7072,7040,7040,7021,7021,6988,6988,6977,6977,6941,6941,6932,6932,6915,6915,6910,6910,6888,6888,6881,6881,6876,6876,6873,6873,6833,6833,6832,6832,6806,6806,6802,6802,6764,6764,6735,6735,6726,6726,6725,6725,6717,6717,6709,6709,6666,6666,6660,6660,6654,6654,6652,6652,6634,6634,6567,6567,6544,6544,6540,6540,6526,6526,6519,6519,6480,6480,6450,6450,6438,6438,6424,6424,6411,6411,6384,6384,6382,6382,6350,6350,6341,6341,6333,6333,6321,6321,6295,6295,6294,6294,6270,6270,6252,6252,6242,6242,6236,6236,6223,6223,6201,6201,6196,6196,6184,6184,6175,6175,6166,6166,6163,6163,6134,6134,6123,6123,6106,6106,6089,6089,6070,6070,6065,6065,6041,6041,6032,6032,6030,6030,6001,6001,5995,5995,5985,5985,5952,5952,5938,5938,5897,5897,5895,5895,5881,5881,5879,5879,5872,5872,5866,5866,5857,5857,5840,5840,5832,5832,5804,5804,5789,5789,5772,5772,5771,5771,5743,5743,5735,5735,5709,5709,5685,5685,5676,5676,5654,5654,5648,5648,5629,5629,5616,5616,5579,5579,5573,5573,5564,5564,5557,5557,5550,5550,5547,5547,5528,5528,5510,5510,5508,5508,5503,5503,5490,5490,5476,5476,5453,5453,5435,5435,5428,5428,5419,5419,5405,5405,5396,5396,5384,5384,5365,5365,5363,5363,5347,5347,5346,5346,5343,5343,5341,5341,5322,5322,5314,5314,5291,5291,5216,5216,5213,5213,5181,5181,5170,5170,5168,5168,5158,5158,5156,5156,5146,5146,5130,5130,5121,5121,5116,5116,5071,5071,5011,5011,5009,5009,5003,5003,4999,4999,4997,4997,4985,4985,4983,4983,4973,4973,4971,4971,4954,4954,4953,4953,4946,4946,4945,4945,4925,4925,4922,4922,4917,4917,4903,4903,4886,4886,4884,4884,4877,4877,4874,4874,4834,4834,4825,4825,4773,4773,4760,4760,4757,4757,4743,4743,4722,4722,4716,4716,4692,4692,4682,4682,4666,4666,4661,4661,4647,4647,4636,4636,4597,4597,4558,4558,4557,4557,4545,4545,4539,4539,4533,4533,4516,4516,4489,4489,4482,4482,4479,4479,4444,4444,4394,4394,4392,4392,4374,4374,4372,4372,4370,4370,4350,4350,4346,4346,4344,4344,4335,4335,4264,4264,4258,4258,4255,4255,4251,4251,4233,4233,4224,4224,4220,4220,4203,4203,4193,4193,4190,4190,4183,4183,4149,4149,4147,4147,4138,4138,4130,4130,4129,4129,4118,4118,4115,4115,4106,4106,4103,4103,4092,4092,4085,4085,4079,4079,4070,4070,4060,4060,4046,4046,4016,4016,3993,3993,3986,3986,3974,3974,3971,3971,3944,3944,3910,3910,3901,3901,3900,3900,3899,3899,3895,3895,3851,3851,3840,3840,3833,3833,3809,3809,3727,3727,3726,3726,3722,3722,3701,3701,3692,3692,3687,3687,3675,3675,3670,3670,3651,3651,3635,3635,3621,3621,3600,3600,3589,3589,3579,3579,3562,3562,3523,3523,3514,3514,3509,3509,3480,3480,3455,3455,3430,3430,3418,3418,3416,3416,3385,3385,3363,3363,3344,3344,3338,3338,3337,3337,3334,3334,3319,3319,3316,3316,3306,3306,3278,3278,3274,3274,3240,3240,3188,3188,3187,3187,3146,3146,3142,3142,3134,3134,3129,3129,3109,3109,3102,3102,3095,3095,3071,3071,3061,3061,3033,3033,3025,3025,3020,3020,2947,2947,2946,2946,2942,2942,2940,2940,2922,2922,2913,2913,2892,2892,2837,2837,2836,2836,2835,2835,2770,2770,2753,2753,2752,2752,2749,2749,2724,2724,2706,2706,2700,2700,2695,2695,2683,2683,2665,2665,2638,2638,2587,2587,2580,2580,2558,2558,2556,2556,2555,2555,2527,2527,2511,2511,2489,2489,2486,2486,2484,2484,2479,2479,2474,2474,2455,2455,2416,2416,2399,2399,2393,2393,2388,2388,2383,2383,2372,2372,2371,2371,2363,2363,2355,2355,2349,2349,2343,2343,2334,2334,2314,2314,2305,2305,2285,2285,2244,2244,2243,2243,2238,2238,2231,2231,2203,2203,2197,2197,2185,2185,2180,2180,2169,2169,2129,2129,2120,2120,2098,2098,2083,2083,2077,2077,2073,2073,2071,2071,2048,2048,2014,2014,2006,2006,2001,2001,1994,1994,1987,1987,1984,1984,1983,1983,1972,1972,1955,1955,1916,1916,1914,1914,1904,1904,1884,1884,1876,1876,1858,1858,1815,1815,1802,1802,1797,1797,1789,1789,1781,1781,1774,1774,1763,1763,1747,1747,1744,1744,1735,1735,1731,1731,1708,1708,1702,1702,1690,1690,1680,1680,1679,1679,1664,1664,1657,1657,1639,1639,1636,1636,1622,1622,1616,1616,1608,1608,1606,1606,1603,1603,1584,1584,1569,1569,1567,1567,1562,1562,1548,1548,1544,1544,1539,1539,1516,1516,1511,1511,1510,1510,1508,1508,1503,1503,1498,1498,1497,1497,1491,1491,1478,1478,1460,1460,1458,1458,1447,1447,1446,1446,1436,1436,1425,1425,1403,1403,1398,1398,1396,1396,1386,1386,1374,1374,1370,1370,1368,1368,1351,1351,1349,1349,1338,1338,1336,1336,1335,1335,1327,1327,1318,1318,1309,1309,1306,1306,1298,1298,1295,1295,1293,1293,1271,1271,1270,1270,1267,1267,1266,1266,1254,1254,1248,1248,1246,1246,1245,1245,1242,1242,1241,1241,1236,1236,1231,1231,1227,1227,1226,1226,1222,1222,1209,1209,1208,1208,1205,1205,1197,1197,1196,1196,1187,1187,1186,1186,1182,1182,1176,1176,1158,1158,1153,1153,1146,1146,1137,1137,1136,1136,1133,1133,1131,1131,1129,1129,1127,1127,1123,1123,1118,1118,1110,1110,1109,1109,1106,1106,1100,1100,1092,1092,1083,1083,1081,1081,1080,1080,1070,1070,1068,1068,1067,1067,1066,1066,1065,1065,1062,1062,1058,1058,1051,1051,1050,1050,1046,1046,1044,1044,1038,1038,1035,1035,1033,1033,1030,1030,1029,1029,1028,1028,1027,1027,1021,1021,1016,1016,1012,1012,1009,1009,998,998,995,995,988,988,983,983,980,980,979,979,976,976,975,975,974,974,973,973,968,968,964,964,961,961,959,959,957,957,954,954,949,949,948,948,946,946,944,944,934,934,927,927,926,926,922,922,921,921,920,920,919,919,917,917,914,914,913,913,905,905,904,904,895,895,892,892,890,890,886,886,879,879,878,878,873,873,870,870,869,869,868,868,866,866,863,863,860,860,858,858,857,857,855,855,854,854,851,851,850,850,848,848,845,845,840,840,837,837,835,835,831,831,828,828,827,827,824,824,821,821,819,819,817,817,816,816,815,815,813,813,812,812,811,811,807,807,806,806,805,805,803,803,800,800,798,798,797,797,795,795,793,793,791,791,790,790,789,789,788,788,787,787,785,785,783,783,782,782,781,781,780,780,779,779,778,778,777,777,775,775,774,774,773,773,771,771,770,770,769,769,767,767,766,766,761,761,757,757,754,754,752,752,751,751,750,750,749,749,748,748,747,747,746,746,744,744,742,742,741,741,739,739,733,733,730,730,729,729,728,728,727,727,726,726,725,725,724,724,722,722,721,721,720,720,718,718,714,714,713,713,711,711,709,709,708,708,707,707,706,706,704,704,703,703,702,702,700,700,699,699,698,698,697,697,696,696,695,695,693,693,692,692,691,691,690,690,689,689,687,687,686,686,685,685,684,684,683,683,682,682,681,681,680,680,679,679,678,678,677,677,675,675,674,674,672,672,671,671,670,670,669,669,668,668,667,667,665,665,663,663,662,662,659,659,658,658,655,655,653,653,652,652,651,651,650,650,649,649,647,647,646,646,645,645,644,644,643,643,642,642,641,641,640,640,639,639,638,638,637,637,636,636,635,635,632,632,631,631,629,629,628,628,627,627,626,626,625,625,624,624,622,622,621,621,619,619,618,618,617,617,616,616,615,615,613,613,612,612,610,610,609,609,608,608,607,607,606,606,605,605,604,604,603,603,602,602,600,600,599,599,598,598,596,596,595,595,594,594,592,592,591,591,590,590,589,589,588,588,586,586,585,585,584,584,583,583,582,582,581,581,580,580,579,579,578,578,577,577,576,576,575,575,574,574,573,573,572,572,571,571,570,570,569,569,568,568,567,567,566,566,565,565,564,564,563,563,562,562,561,561,560,560,559,559,558,558,557,557,556,556,555,555,554,554,553,553,552,552,551,551,550,550,549,549,548,548,546,546,545,545,544,544,543,543,542,542,541,541,539,539,538,538,536,536,535,535,534,534,533,533,532,532,531,531,530,530,529,529,528,528,527,527,526,526,525,525,524,524,523,523,522,522,521,521,520,520,519,519,518,518,517,517,516,516,515,515,514,514,513,513,512,512,511,511,510,510,509,509,508,508,506,506,505,505,504,504,502,502,501,501,500,500,499,499,498,498,497,497,496,496,495,495,494,494,492,492,491,491,490,490,489,489,488,488,487,487,486,486,485,485,484,484,483,483,482,482,481,481,480,480,479,479,478,478,477,477,476,476,475,475,474,474,473,473,472,472,471,471,470,470,469,469,468,468,467,467,466,466,465,465,464,464,463,463,462,462,461,461,460,460,459,459,458,458,457,457,456,456,455,455,454,454,453,453,452,452,451,451,450,450,449,449,448,448,447,447,446,446,445,445,444,444,443,443,442,442,441,441,440,440,439,439,438,438,437,437,436,436,435,435,434,434,433,433,432,432,431,431,430,430,425,425,422,422,420,420,414,414,413,413,412,412,408,408,406,406,401,401,0.0],[182161,182161,182161,173781,173781,166288,166288,137195,137195,129502,129502,128942,128942,126397,126397,124595,124595,122639,122639,120826,120826,117290,117290,115783,115783,115118,115118,111468,111468,110595,110595,109213,109213,107780,107780,106715,106715,106302,106302,106041,106041,105405,105405,102551,102551,101902,101902,101315,101315,101024,101024,100814,100814,97711,97711,95789,95789,94288,94288,93156,93156,91626,91626,91420,91420,90833,90833,90568,90568,87929,87929,86649,86649,86431,86431,85574,85574,84148,84148,83861,83861,83503,83503,81850,81850,81363,81363,80602,80602,79788,79788,77912,77912,77226,77226,77126,77126,76931,76931,76050,76050,75659,75659,75060,75060,74437,74437,73799,73799,73438,73438,72956,72956,72145,72145,71849,71849,71375,71375,71222,71222,71169,71169,71099,71099,70847,70847,70774,70774,69988,69988,69815,69815,69813,69813,69714,69714,69653,69653,69594,69594,69347,69347,68963,68963,68803,68803,68156,68156,67292,67292,66892,66892,66679,66679,66674,66674,65905,65905,65810,65810,65526,65526,65480,65480,65297,65297,65228,65228,64846,64846,64813,64813,64562,64562,64363,64363,64007,64007,63828,63828,63780,63780,63472,63472,63314,63314,62737,62737,62636,62636,62490,62490,62419,62419,62129,62129,61428,61428,61290,61290,61105,61105,59624,59624,59520,59520,59422,59422,59409,59409,58751,58751,58719,58719,58073,58073,57746,57746,57370,57370,57205,57205,57091,57091,56988,56988,56496,56496,55669,55669,55610,55610,55606,55606,55577,55577,55446,55446,55271,55271,55237,55237,54966,54966,54759,54759,54720,54720,54386,54386,54272,54272,54186,54186,54109,54109,53900,53900,53889,53889,53886,53886,53872,53872,53843,53843,53429,53429,53397,53397,53248,53248,53198,53198,53025,53025,53019,53019,52931,52931,52884,52884,52862,52862,52566,52566,52487,52487,52333,52333,52310,52310,52037,52037,51854,51854,51629,51629,51473,51473,51466,51466,51383,51383,51140,51140,51084,51084,51069,51069,51054,51054,50453,50453,50180,50180,50030,50030,49704,49704,49640,49640,49590,49590,49460,49460,49234,49234,49183,49183,49043,49043,48911,48911,48836,48836,48835,48835,48613,48613,48580,48580,48477,48477,48261,48261,47775,47775,47760,47760,47656,47656,47579,47579,47410,47410,47390,47390,47119,47119,47103,47103,47011,47011,46693,46693,46615,46615,46331,46331,46183,46183,46088,46088,45948,45948,45928,45928,45832,45832,45814,45814,45755,45755,45720,45720,45321,45321,45294,45294,45217,45217,45213,45213,45160,45160,45117,45117,44912,44912,44900,44900,44846,44846,44805,44805,44743,44743,44730,44730,44694,44694,44689,44689,44606,44606,44564,44564,44403,44403,44376,44376,44347,44347,44294,44294,44180,44180,44157,44157,44013,44013,43984,43984,43972,43972,43928,43928,43837,43837,43527,43527,43272,43272,43211,43211,43207,43207,43163,43163,42967,42967,42911,42911,42602,42602,42594,42594,42495,42495,42479,42479,42257,42257,42156,42156,42037,42037,42023,42023,41965,41965,41959,41959,41823,41823,41698,41698,41642,41642,41565,41565,41519,41519,41484,41484,41331,41331,41246,41246,41167,41167,41014,41014,40928,40928,40846,40846,40838,40838,40794,40794,40650,40650,40634,40634,40479,40479,40445,40445,40394,40394,40272,40272,40231,40231,40162,40162,39893,39893,39670,39670,39669,39669,39619,39619,39590,39590,39532,39532,39515,39515,39415,39415,39309,39309,39254,39254,38980,38980,38972,38972,38951,38951,38877,38877,38832,38832,38819,38819,38754,38754,38681,38681,38673,38673,38644,38644,38465,38465,38412,38412,38357,38357,38215,38215,38203,38203,38126,38126,38019,38019,37990,37990,37813,37813,37773,37773,37594,37594,37575,37575,37518,37518,37514,37514,37496,37496,37324,37324,37188,37188,37157,37157,37152,37152,37115,37115,36950,36950,36875,36875,36699,36699,36616,36616,36600,36600,36558,36558,36547,36547,36511,36511,36502,36502,36431,36431,36270,36270,36255,36255,36151,36151,36109,36109,35977,35977,35736,35736,35676,35676,35535,35535,35483,35483,35482,35482,35457,35457,35427,35427,35254,35254,35174,35174,34991,34991,34880,34880,34842,34842,34634,34634,34621,34621,34436,34436,34398,34398,34343,34343,34293,34293,34196,34196,34185,34185,34035,34035,34025,34025,34012,34012,34005,34005,33819,33819,33690,33690,33580,33580,33526,33526,33499,33499,33464,33464,33451,33451,33413,33413,33359,33359,33287,33287,33283,33283,33266,33266,33246,33246,33219,33219,33213,33213,33146,33146,33128,33128,32855,32855,32826,32826,32787,32787,32740,32740,32719,32719,32705,32705,32597,32597,32576,32576,32408,32408,32363,32363,32342,32342,32330,32330,32301,32301,32233,32233,32211,32211,32153,32153,32134,32134,32094,32094,32093,32093,32091,32091,32015,32015,31976,31976,31957,31957,31951,31951,31832,31832,31771,31771,31687,31687,31618,31618,31375,31375,31366,31366,31274,31274,31208,31208,31203,31203,31137,31137,31112,31112,31032,31032,31007,31007,30930,30930,30868,30868,30720,30720,30632,30632,30492,30492,30450,30450,30418,30418,30412,30412,30379,30379,30362,30362,30322,30322,30311,30311,30231,30231,30173,30173,30163,30163,30126,30126,30123,30123,30062,30062,30012,30012,29989,29989,29981,29981,29850,29850,29845,29845,29736,29736,29730,29730,29673,29673,29575,29575,29442,29442,29303,29303,29290,29290,29275,29275,29265,29265,29243,29243,29236,29236,29230,29230,29186,29186,29162,29162,29154,29154,29103,29103,29062,29062,29057,29057,29050,29050,29024,29024,28922,28922,28846,28846,28825,28825,28807,28807,28781,28781,28762,28762,28716,28716,28629,28629,28622,28622,28620,28620,28610,28610,28547,28547,28519,28519,28435,28435,28334,28334,28218,28218,28150,28150,28081,28081,28005,28005,27989,27989,27985,27985,27940,27940,27929,27929,27893,27893,27892,27892,27850,27850,27843,27843,27780,27780,27778,27778,27730,27730,27492,27492,27418,27418,27372,27372,27354,27354,27347,27347,27346,27346,27338,27338,27264,27264,27227,27227,27222,27222,27087,27087,27081,27081,26973,26973,26938,26938,26925,26925,26902,26902,26857,26857,26850,26850,26792,26792,26779,26779,26721,26721,26712,26712,26701,26701,26656,26656,26628,26628,26555,26555,26538,26538,26534,26534,26511,26511,26510,26510,26419,26419,26387,26387,26383,26383,26337,26337,26329,26329,26259,26259,26247,26247,26198,26198,26126,26126,26095,26095,26047,26047,25993,25993,25988,25988,25984,25984,25980,25980,25976,25976,25951,25951,25901,25901,25736,25736,25708,25708,25696,25696,25678,25678,25660,25660,25590,25590,25552,25552,25498,25498,25487,25487,25472,25472,25469,25469,25401,25401,25367,25367,25328,25328,25220,25220,25181,25181,25134,25134,25010,25010,24988,24988,24873,24873,24800,24800,24783,24783,24782,24782,24750,24750,24655,24655,24548,24548,24496,24496,24453,24453,24402,24402,24287,24287,24286,24286,24258,24258,24239,24239,24218,24218,24087,24087,24053,24053,23961,23961,23918,23918,23827,23827,23825,23825,23799,23799,23692,23692,23563,23563,23527,23527,23509,23509,23503,23503,23459,23459,23457,23457,23423,23423,23404,23404,23402,23402,23391,23391,23298,23298,23292,23292,23262,23262,23215,23215,23205,23205,23188,23188,23178,23178,23157,23157,23097,23097,22936,22936,22857,22857,22821,22821,22769,22769,22748,22748,22731,22731,22714,22714,22658,22658,22516,22516,22513,22513,22440,22440,22427,22427,22333,22333,22315,22315,22304,22304,22276,22276,22201,22201,22198,22198,22132,22132,22048,22048,22037,22037,22036,22036,22035,22035,22027,22027,21995,21995,21959,21959,21952,21952,21935,21935,21804,21804,21652,21652,21627,21627,21609,21609,21584,21584,21522,21522,21505,21505,21490,21490,21446,21446,21414,21414,21355,21355,21258,21258,21219,21219,21216,21216,21210,21210,21115,21115,21099,21099,21048,21048,21014,21014,21003,21003,20980,20980,20947,20947,20930,20930,20928,20928,20893,20893,20869,20869,20833,20833,20824,20824,20744,20744,20727,20727,20713,20713,20653,20653,20648,20648,20625,20625,20605,20605,20586,20586,20545,20545,20522,20522,20434,20434,20431,20431,20414,20414,20394,20394,20389,20389,20386,20386,20377,20377,20350,20350,20348,20348,20303,20303,20302,20302,20280,20280,20273,20273,20266,20266,20253,20253,20200,20200,20176,20176,20139,20139,20069,20069,20054,20054,20025,20025,20023,20023,20015,20015,19984,19984,19977,19977,19940,19940,19866,19866,19849,19849,19805,19805,19779,19779,19772,19772,19753,19753,19737,19737,19705,19705,19654,19654,19645,19645,19626,19626,19546,19546,19530,19530,19495,19495,19340,19340,19337,19337,19312,19312,19259,19259,19215,19215,19196,19196,19195,19195,19187,19187,19178,19178,19167,19167,19166,19166,19151,19151,19114,19114,19064,19064,19041,19041,19020,19020,19012,19012,19003,19003,18999,18999,18997,18997,18992,18992,18989,18989,18935,18935,18877,18877,18851,18851,18794,18794,18714,18714,18704,18704,18653,18653,18609,18609,18543,18543,18524,18524,18517,18517,18514,18514,18511,18511,18474,18474,18456,18456,18443,18443,18342,18342,18291,18291,18182,18182,18177,18177,18166,18166,18156,18156,18107,18107,18089,18089,18052,18052,18017,18017,17996,17996,17995,17995,17918,17918,17859,17859,17845,17845,17834,17834,17816,17816,17803,17803,17798,17798,17776,17776,17752,17752,17746,17746,17723,17723,17720,17720,17717,17717,17701,17701,17639,17639,17630,17630,17614,17614,17610,17610,17577,17577,17573,17573,17571,17571,17535,17535,17532,17532,17486,17486,17470,17470,17387,17387,17379,17379,17364,17364,17286,17286,17214,17214,17188,17188,17168,17168,17167,17167,17160,17160,17110,17110,17054,17054,17043,17043,17032,17032,16937,16937,16918,16918,16912,16912,16869,16869,16857,16857,16853,16853,16852,16852,16844,16844,16841,16841,16839,16839,16818,16818,16812,16812,16775,16775,16751,16751,16726,16726,16720,16720,16691,16691,16661,16661,16623,16623,16587,16587,16577,16577,16576,16576,16499,16499,16479,16479,16455,16455,16408,16408,16345,16345,16317,16317,16283,16283,16254,16254,16249,16249,16232,16232,16227,16227,16212,16212,16205,16205,16095,16095,16041,16041,15999,15999,15967,15967,15965,15965,15927,15927,15857,15857,15846,15846,15844,15844,15809,15809,15779,15779,15768,15768,15742,15742,15738,15738,15734,15734,15693,15693,15679,15679,15659,15659,15636,15636,15559,15559,15557,15557,15556,15556,15553,15553,15540,15540,15534,15534,15463,15463,15456,15456,15354,15354,15303,15303,15301,15301,15288,15288,15199,15199,15127,15127,15064,15064,15052,15052,15038,15038,14986,14986,14927,14927,14881,14881,14866,14866,14861,14861,14832,14832,14809,14809,14803,14803,14799,14799,14759,14759,14707,14707,14704,14704,14698,14698,14678,14678,14664,14664,14652,14652,14628,14628,14595,14595,14571,14571,14548,14548,14530,14530,14525,14525,14455,14455,14405,14405,14392,14392,14384,14384,14337,14337,14316,14316,14306,14306,14281,14281,14263,14263,14256,14256,14241,14241,14240,14240,14217,14217,14172,14172,14118,14118,14107,14107,14085,14085,13928,13928,13910,13910,13886,13886,13864,13864,13853,13853,13789,13789,13754,13754,13732,13732,13710,13710,13703,13703,13694,13694,13685,13685,13674,13674,13655,13655,13650,13650,13568,13568,13507,13507,13489,13489,13487,13487,13469,13469,13464,13464,13399,13399,13384,13384,13382,13382,13317,13317,13313,13313,13263,13263,13252,13252,13233,13233,13174,13174,13158,13158,13142,13142,13117,13117,13109,13109,13091,13091,13079,13079,13061,13061,13035,13035,13021,13021,13020,13020,13008,13008,12961,12961,12938,12938,12928,12928,12883,12883,12878,12878,12858,12858,12795,12795,12779,12779,12769,12769,12754,12754,12670,12670,12650,12650,12648,12648,12647,12647,12604,12604,12587,12587,12580,12580,12532,12532,12516,12516,12417,12417,12404,12404,12385,12385,12370,12370,12356,12356,12307,12307,12300,12300,12246,12246,12243,12243,12241,12241,12212,12212,12195,12195,12189,12189,12168,12168,12158,12158,12120,12120,12109,12109,12089,12089,12084,12084,12075,12075,12041,12041,12029,12029,12015,12015,11990,11990,11948,11948,11935,11935,11918,11918,11910,11910,11891,11891,11882,11882,11857,11857,11831,11831,11816,11816,11781,11781,11766,11766,11685,11685,11676,11676,11660,11660,11647,11647,11595,11595,11592,11592,11585,11585,11562,11562,11558,11558,11547,11547,11545,11545,11517,11517,11472,11472,11452,11452,11438,11438,11434,11434,11423,11423,11373,11373,11332,11332,11315,11315,11289,11289,11264,11264,11257,11257,11206,11206,11158,11158,11097,11097,11080,11080,11074,11074,11002,11002,10993,10993,10986,10986,10949,10949,10941,10941,10940,10940,10924,10924,10921,10921,10874,10874,10841,10841,10779,10779,10765,10765,10740,10740,10718,10718,10688,10688,10669,10669,10664,10664,10658,10658,10622,10622,10621,10621,10596,10596,10591,10591,10589,10589,10577,10577,10565,10565,10522,10522,10469,10469,10468,10468,10429,10429,10422,10422,10419,10419,10380,10380,10353,10353,10285,10285,10264,10264,10252,10252,10213,10213,10174,10174,10148,10148,10131,10131,10127,10127,10117,10117,10101,10101,10085,10085,10082,10082,10064,10064,10029,10029,10013,10013,10011,10011,9996,9996,9977,9977,9955,9955,9912,9912,9911,9911,9895,9895,9882,9882,9876,9876,9870,9870,9838,9838,9835,9835,9834,9834,9769,9769,9760,9760,9757,9757,9725,9725,9690,9690,9688,9688,9639,9639,9625,9625,9573,9573,9565,9565,9553,9553,9544,9544,9538,9538,9537,9537,9509,9509,9503,9503,9489,9489,9480,9480,9444,9444,9432,9432,9426,9426,9402,9402,9392,9392,9382,9382,9381,9381,9380,9380,9353,9353,9327,9327,9308,9308,9306,9306,9292,9292,9279,9279,9276,9276,9247,9247,9237,9237,9220,9220,9188,9188,9142,9142,9126,9126,9113,9113,9110,9110,9084,9084,9073,9073,9061,9061,9027,9027,8986,8986,8981,8981,8976,8976,8942,8942,8894,8894,8876,8876,8867,8867,8859,8859,8853,8853,8822,8822,8784,8784,8735,8735,8723,8723,8712,8712,8701,8701,8680,8680,8654,8654,8594,8594,8587,8587,8582,8582,8575,8575,8572,8572,8524,8524,8513,8513,8496,8496,8483,8483,8476,8476,8461,8461,8454,8454,8446,8446,8441,8441,8437,8437,8427,8427,8412,8412,8391,8391,8389,8389,8381,8381,8350,8350,8348,8348,8347,8347,8263,8263,8249,8249,8212,8212,8207,8207,8199,8199,8193,8193,8083,8083,8070,8070,8064,8064,8063,8063,8048,8048,8019,8019,8014,8014,8004,8004,7992,7992,7944,7944,7940,7940,7929,7929,7928,7928,7927,7927,7893,7893,7883,7883,7830,7830,7821,7821,7811,7811,7773,7773,7753,7753,7728,7728,7691,7691,7666,7666,7664,7664,7631,7631,7577,7577,7566,7566,7506,7506,7479,7479,7448,7448,7442,7442,7394,7394,7381,7381,7322,7322,7286,7286,7255,7255,7210,7210,7195,7195,7193,7193,7179,7179,7128,7128,7082,7082,7021,7021,6988,6988,6977,6977,6941,6941,6932,6932,6910,6910,6888,6888,6873,6873,6833,6833,6741,6741,6735,6735,6726,6726,6725,6725,6717,6717,6709,6709,6708,6708,6666,6666,6660,6660,6654,6654,6646,6646,6611,6611,6602,6602,6580,6580,6544,6544,6540,6540,6519,6519,6480,6480,6450,6450,6438,6438,6411,6411,6382,6382,6341,6341,6333,6333,6321,6321,6295,6295,6294,6294,6278,6278,6242,6242,6201,6201,6196,6196,6184,6184,6175,6175,6163,6163,6147,6147,6123,6123,6106,6106,6089,6089,6049,6049,6030,6030,6001,6001,5995,5995,5952,5952,5938,5938,5895,5895,5881,5881,5872,5872,5866,5866,5857,5857,5840,5840,5832,5832,5809,5809,5771,5771,5743,5743,5735,5735,5709,5709,5676,5676,5648,5648,5575,5575,5573,5573,5564,5564,5557,5557,5550,5550,5547,5547,5528,5528,5510,5510,5503,5503,5476,5476,5419,5419,5396,5396,5365,5365,5347,5347,5346,5346,5343,5343,5341,5341,5336,5336,5314,5314,5280,5280,5216,5216,5213,5213,5194,5194,5181,5181,5121,5121,5116,5116,5059,5059,5003,5003,4999,4999,4997,4997,4985,4985,4983,4983,4973,4973,4971,4971,4954,4954,4953,4953,4945,4945,4925,4925,4917,4917,4903,4903,4884,4884,4877,4877,4874,4874,4868,4868,4825,4825,4760,4760,4743,4743,4722,4722,4716,4716,4701,4701,4666,4666,4661,4661,4658,4658,4647,4647,4636,4636,4597,4597,4558,4558,4539,4539,4533,4533,4516,4516,4489,4489,4479,4479,4427,4427,4372,4372,4346,4346,4335,4335,4276,4276,4270,4270,4224,4224,4220,4220,4203,4203,4193,4193,4190,4190,4183,4183,4149,4149,4144,4144,4138,4138,4115,4115,4106,4106,4085,4085,4079,4079,4064,4064,4016,4016,3993,3993,3986,3986,3944,3944,3938,3938,3934,3934,3910,3910,3900,3900,3898,3898,3851,3851,3833,3833,3806,3806,3722,3722,3707,3707,3676,3676,3651,3651,3635,3635,3611,3611,3600,3600,3588,3588,3523,3523,3514,3514,3455,3455,3418,3418,3416,3416,3344,3344,3334,3334,3319,3319,3306,3306,3261,3261,3240,3240,3146,3146,3111,3111,3095,3095,3071,3071,2948,2948,2946,2946,2922,2922,2913,2913,2908,2908,2892,2892,2837,2837,2835,2835,2767,2767,2753,2753,2752,2752,2749,2749,2747,2747,2724,2724,2716,2716,2706,2706,2700,2700,2697,2697,2679,2679,2674,2674,2664,2664,2656,2656,2645,2645,2638,2638,2621,2621,2612,2612,2587,2587,2580,2580,2558,2558,2556,2556,2555,2555,2500,2500,2489,2489,2484,2484,2479,2479,2474,2474,2456,2456,2455,2455,2446,2446,2393,2393,2343,2343,2337,2337,2334,2334,2332,2332,2306,2306,2302,2302,2258,2258,2217,2217,2169,2169,2129,2129,2071,2071,2051,2051,2006,2006,2002,2002,2001,2001,1955,1955,1936,1936,1916,1916,1911,1911,1876,1876,1753,1753,1735,1735,1734,1734,1693,1693,1690,1690,1679,1679,1657,1657,1637,1637,1636,1636,1633,1633,1616,1616,1609,1609,1603,1603,1516,1516,1513,1513,1495,1495,1484,1484,1464,1464,1460,1460,1425,1425,1402,1402,1377,1377,1370,1370,1312,1312,1295,1295,1267,1267,1256,1256,1244,1244,1235,1235,1226,1226,1213,1213,1206,1206,1184,1184,1181,1181,1171,1171,1166,1166,1158,1158,1152,1152,1127,1127,1114,1114,1110,1110,1094,1094,1066,1066,1061,1061,1056,1056,1050,1050,1040,1040,1030,1030,1029,1029,1016,1016,995,995,994,994,992,992,990,990,982,982,977,977,968,968,960,960,956,956,955,955,945,945,939,939,935,935,930,930,926,926,915,915,914,914,913,913,907,907,905,905,901,901,894,894,890,890,887,887,882,882,877,877,874,874,873,873,866,866,864,864,863,863,862,862,861,861,857,857,854,854,851,851,850,850,849,849,848,848,846,846,844,844,838,838,837,837,833,833,832,832,831,831,828,828,823,823,822,822,819,819,815,815,812,812,811,811,807,807,805,805,802,802,801,801,797,797,793,793,788,788,787,787,783,783,781,781,780,780,778,778,776,776,775,775,773,773,772,772,771,771,769,769,768,768,766,766,764,764,763,763,758,758,757,757,755,755,754,754,750,750,749,749,748,748,747,747,746,746,745,745,743,743,741,741,736,736,734,734,732,732,730,730,729,729,727,727,725,725,724,724,717,717,716,716,712,712,711,711,710,710,709,709,705,705,704,704,702,702,701,701,699,699,698,698,697,697,695,695,694,694,692,692,691,691,688,688,686,686,685,685,684,684,683,683,682,682,681,681,680,680,677,677,675,675,674,674,672,672,670,670,669,669,668,668,667,667,666,666,665,665,664,664,663,663,662,662,659,659,657,657,656,656,655,655,651,651,650,650,649,649,648,648,647,647,646,646,645,645,644,644,643,643,640,640,639,639,637,637,635,635,634,634,632,632,631,631,630,630,629,629,628,628,626,626,625,625,624,624,623,623,622,622,621,621,619,619,618,618,617,617,616,616,615,615,614,614,613,613,612,612,610,610,609,609,608,608,607,607,606,606,605,605,604,604,603,603,602,602,601,601,600,600,598,598,597,597,596,596,595,595,594,594,593,593,592,592,591,591,590,590,589,589,588,588,587,587,586,586,585,585,584,584,583,583,580,580,579,579,578,578,576,576,575,575,574,574,573,573,572,572,571,571,570,570,569,569,568,568,567,567,566,566,565,565,564,564,563,563,562,562,561,561,560,560,559,559,558,558,557,557,556,556,555,555,554,554,553,553,552,552,551,551,550,550,549,549,548,548,547,547,546,546,545,545,544,544,543,543,542,542,541,541,540,540,539,539,538,538,537,537,536,536,535,535,534,534,533,533,532,532,531,531,530,530,529,529,528,528,527,527,526,526,525,525,524,524,523,523,522,522,521,521,520,520,519,519,518,518,517,517,516,516,515,515,514,514,513,513,512,512,511,511,510,510,509,509,508,508,507,507,506,506,505,505,504,504,503,503,502,502,501,501,500,500,499,499,498,498,497,497,496,496,495,495,494,494,493,493,492,492,491,491,490,490,489,489,488,488,487,487,486,486,485,485,484,484,483,483,482,482,481,481,480,480,479,479,478,478,477,477,476,476,475,475,474,474,473,473,472,472,471,471,470,470,469,469,468,468,467,467,466,466,465,465,464,464,463,463,462,462,461,461,460,460,459,459,458,458,457,457,456,456,455,455,454,454,453,453,452,452,451,451,450,450,449,449,448,448,447,447,446,446,445,445,444,444,443,443,442,442,441,441,440,440,439,439,438,438,437,437,436,436,435,435,434,434,433,433,432,432,431,431,430,430,429,429,420,420,415,415,409,409,0.0],[154093,154093,154093,137572,137572,130024,130024,95133,95133,90516,90516,88301,88301,82683,82683,81794,81794,80050,80050,79622,79622,75970,75970,73724,73724,73444,73444,72462,72462,71246,71246,70434,70434,69796,69796,67253,67253,66685,66685,66635,66635,66629,66629,65542,65542,65203,65203,65181,65181,63814,63814,63683,63683,63450,63450,62705,62705,61160,61160,60513,60513,59680,59680,59458,59458,58835,58835,58702,58702,58396,58396,58333,58333,57935,57935,57754,57754,57527,57527,57401,57401,57318,57318,57099,57099,57071,57071,57029,57029,55693,55693,54808,54808,54725,54725,54594,54594,54272,54272,54076,54076,53845,53845,52917,52917,52487,52487,52473,52473,52390,52390,52022,52022,51969,51969,51928,51928,51920,51920,51533,51533,51454,51454,51322,51322,51116,51116,51103,51103,51091,51091,51015,51015,50809,50809,50429,50429,50281,50281,49951,49951,49907,49907,49880,49880,49413,49413,49284,49284,48889,48889,48825,48825,48781,48781,48662,48662,48646,48646,48604,48604,48334,48334,48244,48244,48177,48177,47827,47827,47776,47776,47418,47418,47199,47199,47194,47194,46883,46883,46760,46760,46737,46737,46105,46105,46073,46073,46021,46021,45984,45984,45670,45670,45659,45659,45622,45622,45463,45463,45324,45324,45052,45052,44981,44981,44939,44939,44918,44918,44832,44832,44827,44827,44694,44694,44646,44646,44628,44628,44264,44264,44132,44132,44033,44033,44021,44021,43973,43973,43923,43923,43851,43851,43352,43352,43283,43283,43249,43249,42061,42061,42056,42056,41952,41952,41947,41947,41864,41864,41824,41824,41710,41710,41576,41576,41379,41379,41216,41216,41174,41174,41163,41163,41126,41126,41042,41042,40899,40899,40850,40850,40843,40843,40824,40824,40796,40796,40700,40700,40544,40544,40504,40504,40496,40496,40440,40440,40248,40248,40177,40177,40154,40154,39927,39927,39859,39859,39744,39744,39661,39661,39650,39650,39559,39559,39280,39280,39093,39093,39082,39082,38968,38968,38923,38923,38896,38896,38803,38803,38747,38747,38624,38624,38607,38607,38489,38489,38338,38338,38239,38239,38090,38090,38084,38084,38064,38064,37863,37863,37742,37742,37699,37699,37698,37698,37648,37648,37637,37637,37335,37335,37247,37247,36800,36800,36799,36799,36743,36743,36718,36718,36635,36635,36442,36442,36353,36353,36084,36084,35869,35869,35845,35845,35837,35837,35803,35803,35761,35761,35459,35459,35445,35445,35375,35375,35354,35354,35337,35337,35336,35336,35303,35303,35205,35205,35192,35192,35171,35171,35161,35161,35045,35045,34976,34976,34841,34841,34801,34801,34654,34654,34614,34614,34611,34611,34599,34599,34527,34527,34519,34519,34488,34488,34465,34465,34388,34388,34379,34379,34290,34290,34157,34157,33975,33975,33962,33962,33877,33877,33872,33872,33864,33864,33803,33803,33776,33776,33756,33756,33695,33695,33677,33677,33633,33633,33620,33620,33603,33603,33486,33486,33449,33449,33349,33349,33218,33218,33208,33208,33132,33132,33056,33056,32977,32977,32934,32934,32920,32920,32827,32827,32825,32825,32796,32796,32789,32789,32763,32763,32660,32660,32646,32646,32645,32645,32637,32637,32626,32626,32428,32428,32394,32394,32377,32377,32367,32367,32220,32220,32177,32177,32132,32132,32119,32119,32077,32077,32025,32025,32010,32010,32001,32001,31981,31981,31868,31868,31725,31725,31660,31660,31618,31618,31566,31566,31528,31528,31503,31503,31414,31414,31399,31399,31391,31391,31322,31322,31304,31304,31285,31285,31271,31271,31160,31160,31095,31095,30926,30926,30817,30817,30772,30772,30737,30737,30635,30635,30621,30621,30589,30589,30583,30583,30576,30576,30512,30512,30482,30482,30417,30417,30380,30380,30364,30364,30347,30347,30288,30288,30174,30174,30169,30169,30046,30046,29983,29983,29957,29957,29938,29938,29720,29720,29536,29536,29518,29518,29516,29516,29412,29412,29344,29344,29243,29243,29227,29227,29220,29220,29117,29117,29098,29098,29083,29083,28971,28971,28954,28954,28899,28899,28885,28885,28869,28869,28861,28861,28842,28842,28695,28695,28690,28690,28678,28678,28579,28579,28555,28555,28529,28529,28422,28422,28394,28394,28388,28388,28360,28360,28355,28355,28347,28347,28338,28338,28294,28294,28255,28255,28198,28198,28075,28075,28029,28029,27993,27993,27894,27894,27842,27842,27722,27722,27645,27645,27624,27624,27610,27610,27585,27585,27558,27558,27524,27524,27447,27447,27441,27441,27369,27369,27324,27324,27239,27239,27206,27206,27205,27205,27197,27197,27161,27161,27152,27152,27117,27117,27070,27070,27048,27048,27007,27007,27004,27004,27001,27001,26995,26995,26891,26891,26836,26836,26833,26833,26770,26770,26710,26710,26681,26681,26675,26675,26556,26556,26509,26509,26475,26475,26468,26468,26440,26440,26438,26438,26428,26428,26376,26376,26354,26354,26345,26345,26331,26331,26309,26309,26301,26301,26265,26265,26198,26198,26197,26197,26178,26178,26163,26163,26097,26097,26062,26062,26035,26035,25981,25981,25972,25972,25964,25964,25955,25955,25947,25947,25903,25903,25893,25893,25857,25857,25790,25790,25756,25756,25735,25735,25695,25695,25655,25655,25605,25605,25568,25568,25519,25519,25516,25516,25487,25487,25443,25443,25438,25438,25437,25437,25357,25357,25333,25333,25308,25308,25300,25300,25262,25262,25261,25261,25229,25229,25224,25224,25098,25098,25089,25089,25081,25081,24993,24993,24925,24925,24922,24922,24875,24875,24862,24862,24829,24829,24782,24782,24753,24753,24683,24683,24467,24467,24433,24433,24419,24419,24405,24405,24359,24359,24315,24315,24278,24278,24248,24248,24244,24244,24222,24222,24195,24195,24114,24114,24096,24096,24060,24060,23984,23984,23931,23931,23898,23898,23833,23833,23801,23801,23767,23767,23764,23764,23684,23684,23681,23681,23627,23627,23565,23565,23548,23548,23523,23523,23501,23501,23489,23489,23456,23456,23419,23419,23323,23323,23311,23311,23308,23308,23252,23252,23195,23195,23189,23189,23148,23148,23143,23143,23090,23090,23040,23040,23024,23024,23021,23021,22999,22999,22910,22910,22884,22884,22747,22747,22727,22727,22708,22708,22663,22663,22653,22653,22619,22619,22599,22599,22590,22590,22584,22584,22575,22575,22560,22560,22512,22512,22483,22483,22480,22480,22475,22475,22471,22471,22388,22388,22359,22359,22348,22348,22318,22318,22229,22229,22220,22220,22211,22211,22165,22165,22125,22125,22101,22101,22039,22039,22015,22015,21912,21912,21911,21911,21839,21839,21828,21828,21810,21810,21792,21792,21786,21786,21753,21753,21729,21729,21724,21724,21723,21723,21708,21708,21663,21663,21656,21656,21650,21650,21628,21628,21613,21613,21593,21593,21549,21549,21458,21458,21436,21436,21428,21428,21367,21367,21349,21349,21332,21332,21328,21328,21313,21313,21268,21268,21228,21228,21168,21168,21105,21105,21101,21101,21065,21065,21060,21060,20998,20998,20965,20965,20961,20961,20943,20943,20926,20926,20915,20915,20913,20913,20905,20905,20867,20867,20835,20835,20731,20731,20696,20696,20662,20662,20637,20637,20586,20586,20565,20565,20563,20563,20531,20531,20499,20499,20442,20442,20419,20419,20414,20414,20375,20375,20333,20333,20325,20325,20290,20290,20266,20266,20206,20206,20143,20143,20117,20117,20077,20077,20013,20013,19981,19981,19971,19971,19961,19961,19938,19938,19933,19933,19930,19930,19910,19910,19901,19901,19882,19882,19814,19814,19811,19811,19786,19786,19759,19759,19740,19740,19716,19716,19700,19700,19692,19692,19689,19689,19645,19645,19644,19644,19605,19605,19596,19596,19595,19595,19527,19527,19513,19513,19486,19486,19447,19447,19446,19446,19425,19425,19417,19417,19407,19407,19316,19316,19309,19309,19286,19286,19268,19268,19252,19252,19240,19240,19219,19219,19202,19202,19201,19201,19182,19182,19123,19123,19099,19099,19066,19066,19060,19060,19046,19046,19022,19022,18998,18998,18991,18991,18978,18978,18943,18943,18935,18935,18906,18906,18888,18888,18873,18873,18870,18870,18853,18853,18843,18843,18775,18775,18748,18748,18747,18747,18729,18729,18722,18722,18721,18721,18693,18693,18684,18684,18675,18675,18654,18654,18651,18651,18648,18648,18644,18644,18643,18643,18621,18621,18587,18587,18568,18568,18567,18567,18566,18566,18559,18559,18531,18531,18530,18530,18529,18529,18476,18476,18470,18470,18466,18466,18448,18448,18429,18429,18427,18427,18416,18416,18405,18405,18392,18392,18391,18391,18363,18363,18319,18319,18271,18271,18258,18258,18230,18230,18228,18228,18123,18123,18088,18088,18068,18068,18065,18065,18031,18031,18006,18006,17989,17989,17987,17987,17950,17950,17943,17943,17924,17924,17917,17917,17915,17915,17907,17907,17904,17904,17809,17809,17801,17801,17777,17777,17721,17721,17717,17717,17711,17711,17705,17705,17672,17672,17667,17667,17666,17666,17638,17638,17637,17637,17540,17540,17534,17534,17524,17524,17494,17494,17466,17466,17454,17454,17437,17437,17426,17426,17424,17424,17412,17412,17393,17393,17376,17376,17374,17374,17366,17366,17352,17352,17345,17345,17336,17336,17325,17325,17306,17306,17296,17296,17282,17282,17265,17265,17235,17235,17222,17222,17205,17205,17203,17203,17187,17187,17184,17184,17174,17174,17161,17161,17160,17160,17140,17140,17127,17127,17081,17081,17057,17057,17050,17050,17039,17039,17038,17038,17034,17034,17033,17033,17014,17014,16977,16977,16907,16907,16899,16899,16888,16888,16828,16828,16819,16819,16815,16815,16788,16788,16787,16787,16780,16780,16774,16774,16758,16758,16754,16754,16730,16730,16709,16709,16666,16666,16652,16652,16645,16645,16639,16639,16619,16619,16586,16586,16569,16569,16558,16558,16537,16537,16532,16532,16530,16530,16519,16519,16518,16518,16504,16504,16490,16490,16487,16487,16480,16480,16479,16479,16476,16476,16469,16469,16357,16357,16323,16323,16311,16311,16264,16264,16258,16258,16256,16256,16193,16193,16188,16188,16164,16164,16159,16159,16132,16132,16072,16072,16069,16069,16068,16068,16060,16060,16035,16035,15989,15989,15952,15952,15941,15941,15908,15908,15905,15905,15895,15895,15886,15886,15865,15865,15824,15824,15786,15786,15762,15762,15761,15761,15740,15740,15712,15712,15710,15710,15709,15709,15661,15661,15660,15660,15655,15655,15643,15643,15637,15637,15623,15623,15581,15581,15508,15508,15500,15500,15491,15491,15483,15483,15467,15467,15464,15464,15448,15448,15435,15435,15369,15369,15346,15346,15332,15332,15258,15258,15189,15189,15172,15172,15135,15135,15110,15110,15070,15070,15065,15065,15045,15045,15008,15008,14978,14978,14959,14959,14942,14942,14938,14938,14887,14887,14875,14875,14867,14867,14865,14865,14859,14859,14852,14852,14806,14806,14797,14797,14788,14788,14783,14783,14771,14771,14756,14756,14754,14754,14748,14748,14745,14745,14710,14710,14699,14699,14679,14679,14668,14668,14647,14647,14638,14638,14629,14629,14627,14627,14624,14624,14597,14597,14554,14554,14526,14526,14525,14525,14493,14493,14452,14452,14440,14440,14427,14427,14394,14394,14387,14387,14368,14368,14347,14347,14319,14319,14281,14281,14276,14276,14274,14274,14217,14217,14175,14175,14161,14161,14156,14156,14143,14143,14121,14121,14106,14106,14101,14101,14093,14093,14075,14075,14071,14071,13986,13986,13977,13977,13976,13976,13952,13952,13950,13950,13945,13945,13930,13930,13921,13921,13918,13918,13852,13852,13841,13841,13813,13813,13802,13802,13796,13796,13787,13787,13775,13775,13754,13754,13734,13734,13731,13731,13720,13720,13719,13719,13703,13703,13701,13701,13695,13695,13694,13694,13690,13690,13683,13683,13682,13682,13657,13657,13649,13649,13634,13634,13631,13631,13629,13629,13616,13616,13601,13601,13554,13554,13542,13542,13540,13540,13528,13528,13520,13520,13462,13462,13438,13438,13413,13413,13369,13369,13335,13335,13331,13331,13322,13322,13306,13306,13299,13299,13289,13289,13254,13254,13252,13252,13240,13240,13228,13228,13179,13179,13175,13175,13152,13152,13151,13151,13102,13102,13094,13094,13092,13092,13077,13077,13053,13053,13011,13011,13009,13009,12997,12997,12989,12989,12981,12981,12970,12970,12955,12955,12947,12947,12926,12926,12901,12901,12897,12897,12847,12847,12837,12837,12832,12832,12830,12830,12820,12820,12818,12818,12785,12785,12772,12772,12753,12753,12751,12751,12733,12733,12729,12729,12726,12726,12715,12715,12701,12701,12697,12697,12655,12655,12634,12634,12613,12613,12596,12596,12592,12592,12588,12588,12564,12564,12558,12558,12549,12549,12536,12536,12503,12503,12495,12495,12487,12487,12486,12486,12432,12432,12428,12428,12402,12402,12396,12396,12389,12389,12385,12385,12375,12375,12371,12371,12348,12348,12340,12340,12338,12338,12337,12337,12331,12331,12326,12326,12303,12303,12301,12301,12295,12295,12292,12292,12287,12287,12276,12276,12227,12227,12213,12213,12198,12198,12177,12177,12159,12159,12139,12139,12117,12117,12105,12105,12085,12085,12079,12079,12075,12075,12070,12070,12052,12052,12050,12050,12046,12046,12044,12044,12042,12042,12032,12032,12030,12030,12011,12011,12000,12000,11999,11999,11991,11991,11979,11979,11978,11978,11962,11962,11943,11943,11928,11928,11918,11918,11915,11915,11914,11914,11911,11911,11906,11906,11905,11905,11895,11895,11893,11893,11888,11888,11882,11882,11881,11881,11872,11872,11865,11865,11859,11859,11854,11854,11828,11828,11825,11825,11801,11801,11772,11772,11755,11755,11744,11744,11693,11693,11682,11682,11680,11680,11673,11673,11657,11657,11655,11655,11632,11632,11615,11615,11610,11610,11606,11606,11597,11597,11594,11594,11587,11587,11582,11582,11554,11554,11537,11537,11535,11535,11527,11527,11512,11512,11503,11503,11501,11501,11500,11500,11490,11490,11484,11484,11482,11482,11471,11471,11469,11469,11443,11443,11409,11409,11386,11386,11382,11382,11380,11380,11366,11366,11346,11346,11344,11344,11334,11334,11332,11332,11330,11330,11322,11322,11294,11294,11292,11292,11267,11267,11264,11264,11231,11231,11216,11216,11206,11206,11201,11201,11196,11196,11191,11191,11171,11171,11153,11153,11152,11152,11130,11130,11127,11127,11124,11124,11092,11092,11083,11083,11082,11082,11069,11069,11061,11061,11047,11047,11039,11039,11018,11018,11011,11011,10986,10986,10972,10972,10966,10966,10952,10952,10946,10946,10930,10930,10926,10926,10908,10908,10897,10897,10891,10891,10843,10843,10842,10842,10813,10813,10797,10797,10791,10791,10782,10782,10779,10779,10766,10766,10761,10761,10760,10760,10759,10759,10744,10744,10726,10726,10725,10725,10722,10722,10721,10721,10684,10684,10679,10679,10668,10668,10616,10616,10615,10615,10605,10605,10589,10589,10566,10566,10564,10564,10557,10557,10549,10549,10543,10543,10541,10541,10540,10540,10532,10532,10515,10515,10507,10507,10502,10502,10492,10492,10474,10474,10462,10462,10447,10447,10441,10441,10440,10440,10423,10423,10415,10415,10414,10414,10389,10389,10373,10373,10355,10355,10348,10348,10343,10343,10310,10310,10304,10304,10296,10296,10283,10283,10277,10277,10266,10266,10265,10265,10262,10262,10255,10255,10232,10232,10226,10226,10202,10202,10197,10197,10189,10189,10185,10185,10175,10175,10173,10173,10140,10140,10138,10138,10136,10136,10131,10131,10112,10112,10110,10110,10108,10108,10107,10107,10104,10104,10097,10097,10081,10081,10078,10078,10057,10057,10047,10047,10043,10043,10030,10030,10007,10007,9989,9989,9986,9986,9977,9977,9966,9966,9937,9937,9929,9929,9908,9908,9896,9896,9878,9878,9866,9866,9864,9864,9819,9819,9813,9813,9808,9808,9805,9805,9791,9791,9781,9781,9772,9772,9762,9762,9760,9760,9736,9736,9723,9723,9717,9717,9704,9704,9701,9701,9696,9696,9689,9689,9681,9681,9675,9675,9667,9667,9636,9636,9631,9631,9616,9616,9614,9614,9594,9594,9580,9580,9568,9568,9565,9565,9559,9559,9549,9549,9545,9545,9532,9532,9527,9527,9523,9523,9520,9520,9511,9511,9505,9505,9500,9500,9478,9478,9476,9476,9466,9466,9458,9458,9434,9434,9430,9430,9428,9428,9409,9409,9403,9403,9392,9392,9387,9387,9385,9385,9379,9379,9370,9370,9367,9367,9354,9354,9349,9349,9346,9346,9328,9328,9325,9325,9302,9302,9301,9301,9282,9282,9248,9248,9237,9237,9223,9223,9204,9204,9198,9198,9179,9179,9171,9171,9166,9166,9162,9162,9161,9161,9155,9155,9151,9151,9147,9147,9144,9144,9101,9101,9100,9100,9094,9094,9085,9085,9083,9083,9078,9078,9065,9065,9057,9057,9054,9054,9050,9050,9039,9039,8998,8998,8995,8995,8948,8948,8946,8946,8932,8932,8930,8930,8915,8915,8914,8914,8905,8905,8901,8901,8887,8887,8884,8884,8874,8874,8863,8863,8862,8862,8857,8857,8834,8834,8830,8830,8786,8786,8763,8763,8753,8753,8752,8752,8748,8748,8743,8743,8739,8739,8709,8709,8707,8707,8701,8701,8696,8696,8683,8683,8663,8663,8659,8659,8639,8639,8617,8617,8584,8584,8578,8578,8567,8567,8565,8565,8545,8545,8532,8532,8519,8519,8512,8512,8498,8498,8493,8493,8476,8476,8467,8467,8466,8466,8465,8465,8459,8459,8454,8454,8440,8440,8436,8436,8422,8422,8420,8420,8411,8411,8393,8393,8384,8384,8383,8383,8380,8380,8369,8369,8358,8358,8342,8342,8341,8341,8331,8331,8323,8323,8319,8319,8315,8315,8303,8303,8302,8302,8297,8297,8285,8285,8282,8282,8280,8280,8279,8279,8271,8271,8241,8241,8236,8236,8224,8224,8218,8218,8216,8216,8204,8204,8198,8198,8181,8181,8149,8149,8109,8109,8106,8106,8105,8105,8097,8097,8092,8092,8077,8077,8058,8058,8017,8017,8006,8006,8005,8005,7986,7986,7970,7970,7959,7959,7947,7947,7945,7945,7921,7921,7908,7908,7892,7892,7888,7888,7862,7862,7859,7859,7855,7855,7843,7843,7805,7805,7803,7803,7801,7801,7798,7798,7797,7797,7783,7783,7776,7776,7769,7769,7761,7761,7757,7757,7742,7742,7739,7739,7733,7733,7732,7732,7727,7727,7726,7726,7715,7715,7709,7709,7698,7698,7673,7673,7639,7639,7633,7633,7615,7615,7613,7613,7611,7611,7584,7584,7562,7562,7512,7512,7504,7504,7497,7497,7496,7496,7476,7476,7471,7471,7442,7442,7428,7428,7426,7426,7414,7414,7402,7402,7399,7399,7388,7388,7383,7383,7378,7378,7369,7369,7368,7368,7362,7362,7361,7361,7358,7358,7352,7352,7339,7339,7318,7318,7314,7314,7291,7291,7283,7283,7280,7280,7279,7279,7278,7278,7265,7265,7263,7263,7255,7255,7254,7254,7251,7251,7250,7250,7249,7249,7247,7247,7243,7243,7235,7235,7232,7232,7230,7230,7229,7229,7223,7223,7219,7219,7213,7213,7207,7207,7206,7206,7192,7192,7184,7184,7182,7182,7174,7174,7141,7141,7127,7127,7084,7084,7082,7082,7071,7071,7067,7067,7052,7052,7050,7050,7042,7042,7037,7037,7029,7029,7018,7018,7014,7014,7011,7011,6998,6998,6981,6981,6976,6976,6971,6971,6969,6969,6965,6965,6962,6962,6955,6955,6954,6954,6946,6946,6939,6939,6936,6936,6932,6932,6928,6928,6925,6925,6922,6922,6921,6921,6917,6917,6907,6907,6897,6897,6896,6896,6893,6893,6868,6868,6860,6860,6851,6851,6849,6849,6848,6848,6846,6846,6840,6840,6831,6831,6828,6828,6825,6825,6804,6804,6801,6801,6798,6798,6784,6784,6761,6761,6753,6753,6743,6743,6726,6726,6724,6724,6717,6717,6711,6711,6701,6701,6698,6698,6686,6686,6664,6664,6660,6660,6659,6659,6649,6649,6629,6629,6623,6623,6622,6622,6615,6615,6611,6611,6606,6606,6604,6604,6603,6603,6601,6601,6589,6589,6583,6583,6576,6576,6575,6575,6567,6567,6562,6562,6561,6561,6553,6553,6549,6549,6530,6530,6529,6529,6516,6516,6504,6504,6488,6488,6485,6485,6480,6480,6464,6464,6422,6422,6421,6421,6407,6407,6387,6387,6385,6385,6374,6374,6371,6371,6364,6364,6363,6363,6342,6342,6341,6341,6336,6336,6334,6334,6333,6333,6328,6328,6316,6316,6295,6295,6290,6290,6286,6286,6283,6283,6274,6274,6266,6266,6263,6263,6262,6262,6248,6248,6245,6245,6241,6241,6236,6236,6235,6235,6226,6226,6224,6224,6215,6215,6214,6214,6213,6213,6211,6211,6208,6208,6186,6186,6170,6170,6162,6162,6152,6152,6148,6148,6116,6116,6109,6109,6092,6092,6085,6085,6080,6080,6072,6072,6054,6054,6050,6050,6044,6044,6043,6043,6038,6038,6037,6037,6032,6032,6029,6029,6023,6023,6020,6020,6016,6016,6014,6014,6009,6009,5989,5989,5986,5986,5979,5979,5975,5975,5974,5974,5968,5968,5966,5966,5953,5953,5948,5948,5942,5942,5928,5928,5927,5927,5918,5918,5915,5915,5905,5905,5899,5899,5898,5898,5896,5896,5895,5895,5888,5888,5879,5879,5873,5873,5872,5872,5865,5865,5858,5858,5857,5857,5842,5842,5827,5827,5825,5825,5819,5819,5816,5816,5808,5808,5793,5793,5786,5786,5784,5784,5778,5778,5774,5774,5771,5771,5767,5767,5760,5760,5756,5756,5755,5755,5753,5753,5751,5751,5727,5727,5723,5723,5720,5720,5710,5710,5705,5705,5685,5685,5680,5680,5674,5674,5665,5665,5657,5657,5632,5632,5606,5606,5570,5570,5568,5568,5567,5567,5559,5559,5557,5557,5555,5555,5554,5554,5541,5541,5536,5536,5530,5530,5521,5521,5516,5516,5510,5510,5504,5504,5502,5502,5490,5490,5483,5483,5482,5482,5473,5473,5466,5466,5459,5459,5456,5456,5454,5454,5446,5446,5442,5442,5430,5430,5428,5428,5418,5418,5416,5416,5413,5413,5402,5402,5397,5397,5384,5384,5378,5378,5366,5366,5365,5365,5363,5363,5358,5358,5357,5357,5337,5337,5324,5324,5319,5319,5317,5317,5306,5306,5303,5303,5301,5301,5298,5298,5290,5290,5287,5287,5283,5283,5282,5282,5272,5272,5265,5265,5256,5256,5239,5239,5232,5232,5226,5226,5212,5212,5192,5192,5184,5184,5160,5160,5159,5159,5141,5141,5134,5134,5095,5095,5093,5093,5085,5085,5083,5083,5062,5062,5058,5058,5050,5050,5046,5046,5043,5043,5027,5027,5025,5025,5021,5021,5015,5015,5004,5004,4999,4999,4990,4990,4976,4976,4974,4974,4963,4963,4957,4957,4947,4947,4935,4935,4920,4920,4919,4919,4916,4916,4912,4912,4906,4906,4891,4891,4883,4883,4877,4877,4874,4874,4868,4868,4865,4865,4847,4847,4845,4845,4842,4842,4839,4839,4837,4837,4832,4832,4826,4826,4813,4813,4778,4778,4774,4774,4769,4769,4766,4766,4764,4764,4754,4754,4749,4749,4737,4737,4734,4734,4725,4725,4717,4717,4713,4713,4700,4700,4678,4678,4675,4675,4669,4669,4668,4668,4661,4661,4658,4658,4657,4657,4645,4645,4626,4626,4614,4614,4613,4613,4608,4608,4602,4602,4601,4601,4596,4596,4592,4592,4588,4588,4585,4585,4579,4579,4572,4572,4555,4555,4547,4547,4543,4543,4539,4539,4537,4537,4530,4530,4527,4527,4514,4514,4506,4506,4478,4478,4462,4462,4457,4457,4454,4454,4448,4448,4441,4441,4432,4432,4422,4422,4419,4419,4414,4414,4410,4410,4409,4409,4404,4404,4403,4403,4398,4398,4390,4390,4386,4386,4385,4385,4381,4381,4371,4371,4366,4366,4354,4354,4352,4352,4344,4344,4340,4340,4324,4324,4323,4323,4308,4308,4307,4307,4306,4306,4290,4290,4283,4283,4279,4279,4273,4273,4270,4270,4268,4268,4266,4266,4260,4260,4258,4258,4251,4251,4246,4246,4245,4245,4238,4238,4237,4237,4231,4231,4215,4215,4213,4213,4212,4212,4196,4196,4187,4187,4184,4184,4171,4171,4170,4170,4156,4156,4153,4153,4142,4142,4138,4138,4132,4132,4125,4125,4122,4122,4112,4112,4111,4111,4110,4110,4108,4108,4104,4104,4100,4100,4094,4094,4079,4079,4074,4074,4066,4066,4064,4064,4060,4060,4057,4057,4055,4055,4049,4049,4047,4047,4045,4045,4034,4034,4029,4029,4020,4020,4018,4018,4009,4009,4007,4007,3999,3999,3991,3991,3986,3986,3978,3978,3977,3977,3976,3976,3974,3974,3968,3968,3967,3967,3954,3954,3939,3939,3934,3934,3924,3924,3919,3919,3910,3910,3904,3904,3899,3899,3894,3894,3893,3893,3890,3890,3867,3867,3861,3861,3830,3830,3822,3822,3818,3818,3814,3814,3805,3805,3795,3795,3794,3794,3783,3783,3780,3780,3774,3774,3771,3771,3728,3728,3719,3719,3704,3704,3702,3702,3700,3700,3696,3696,3689,3689,3681,3681,3649,3649,3646,3646,3638,3638,3636,3636,3635,3635,3625,3625,3624,3624,3619,3619,3617,3617,3615,3615,3613,3613,3607,3607,3606,3606,3601,3601,3592,3592,3588,3588,3582,3582,3571,3571,3566,3566,3565,3565,3554,3554,3553,3553,3549,3549,3547,3547,3545,3545,3542,3542,3540,3540,3539,3539,3535,3535,3523,3523,3510,3510,3487,3487,3477,3477,3468,3468,3458,3458,3454,3454,3449,3449,3437,3437,3436,3436,3434,3434,3428,3428,3414,3414,3402,3402,3353,3353,3348,3348,3340,3340,3316,3316,3311,3311,3308,3308,3290,3290,3281,3281,3263,3263,3252,3252,3249,3249,3247,3247,3228,3228,3224,3224,3213,3213,3204,3204,3202,3202,3195,3195,3190,3190,3189,3189,3188,3188,3177,3177,3166,3166,3165,3165,3149,3149,3132,3132,3129,3129,3120,3120,3119,3119,3118,3118,3117,3117,3106,3106,3100,3100,3095,3095,3086,3086,3084,3084,3073,3073,3057,3057,3055,3055,3042,3042,3040,3040,3029,3029,3027,3027,3023,3023,3019,3019,3017,3017,2999,2999,2997,2997,2995,2995,2991,2991,2990,2990,2971,2971,2965,2965,2957,2957,2931,2931,2928,2928,2917,2917,2897,2897,2890,2890,2880,2880,2871,2871,2870,2870,2863,2863,2856,2856,2852,2852,2845,2845,2836,2836,2832,2832,2809,2809,2806,2806,2804,2804,2802,2802,2800,2800,2799,2799,2793,2793,2783,2783,2780,2780,2769,2769,2758,2758,2755,2755,2753,2753,2749,2749,2747,2747,2738,2738,2735,2735,2718,2718,2699,2699,2696,2696,2692,2692,2691,2691,2659,2659,2657,2657,2646,2646,2635,2635,2631,2631,2626,2626,2619,2619,2618,2618,2603,2603,2596,2596,2583,2583,2570,2570,2552,2552,2547,2547,2537,2537,2491,2491,2487,2487,2483,2483,2468,2468,2459,2459,2451,2451,2450,2450,2448,2448,2436,2436,2431,2431,2429,2429,2428,2428,2423,2423,2420,2420,2413,2413,2405,2405,2404,2404,2400,2400,2389,2389,2388,2388,2383,2383,2375,2375,2373,2373,2371,2371,2370,2370,2359,2359,2358,2358,2349,2349,2326,2326,2323,2323,2317,2317,2316,2316,2314,2314,2307,2307,2299,2299,2287,2287,2280,2280,2277,2277,2272,2272,2266,2266,2264,2264,2256,2256,2223,2223,2220,2220,2202,2202,2198,2198,2192,2192,2189,2189,2187,2187,2182,2182,2169,2169,2160,2160,2157,2157,2154,2154,2135,2135,2133,2133,2120,2120,2117,2117,2103,2103,2099,2099,2090,2090,2089,2089,2086,2086,2080,2080,2077,2077,2069,2069,2051,2051,2039,2039,2038,2038,2035,2035,2027,2027,2020,2020,2019,2019,2012,2012,2006,2006,2003,2003,1993,1993,1991,1991,1983,1983,1980,1980,1965,1965,1964,1964,1962,1962,1953,1953,1943,1943,1938,1938,1913,1913,1910,1910,1902,1902,1895,1895,1889,1889,1888,1888,1869,1869,1863,1863,1862,1862,1853,1853,1852,1852,1835,1835,1828,1828,1824,1824,1814,1814,1806,1806,1804,1804,1802,1802,1800,1800,1799,1799,1795,1795,1777,1777,1769,1769,1765,1765,1753,1753,1750,1750,1741,1741,1734,1734,1729,1729,1725,1725,1716,1716,1709,1709,1704,1704,1703,1703,1694,1694,1679,1679,1672,1672,1664,1664,1661,1661,1645,1645,1640,1640,1634,1634,1630,1630,1628,1628,1626,1626,1605,1605,1592,1592,1587,1587,1586,1586,1581,1581,1576,1576,1569,1569,1567,1567,1560,1560,1555,1555,1553,1553,1542,1542,1539,1539,1534,1534,1531,1531,1529,1529,1523,1523,1519,1519,1515,1515,1514,1514,1485,1485,1479,1479,1475,1475,1472,1472,1449,1449,1447,1447,1436,1436,1427,1427,1421,1421,1417,1417,1413,1413,1407,1407,1405,1405,1404,1404,1403,1403,1384,1384,1380,1380,1379,1379,1367,1367,1364,1364,1354,1354,1350,1350,1348,1348,1344,1344,1343,1343,1336,1336,1335,1335,1334,1334,1329,1329,1317,1317,1311,1311,1302,1302,1294,1294,1288,1288,1280,1280,1277,1277,1273,1273,1269,1269,1264,1264,1260,1260,1259,1259,1257,1257,1255,1255,1251,1251,1249,1249,1246,1246,1241,1241,1233,1233,1227,1227,1217,1217,1216,1216,1210,1210,1206,1206,1195,1195,1188,1188,1184,1184,1183,1183,1163,1163,1159,1159,1158,1158,1155,1155,1152,1152,1146,1146,1142,1142,1133,1133,1124,1124,1107,1107,1105,1105,1101,1101,1096,1096,1081,1081,1066,1066,1065,1065,1062,1062,1059,1059,1046,1046,1045,1045,1031,1031,1028,1028,1025,1025,1014,1014,1013,1013,1009,1009,1004,1004,1002,1002,1001,1001,1000,1000,995,995,988,988,987,987,984,984,981,981,972,972,968,968,965,965,964,964,963,963,957,957,953,953,948,948,945,945,942,942,926,926,921,921,914,914,911,911,904,904,899,899,896,896,895,895,891,891,884,884,880,880,876,876,868,868,867,867,864,864,862,862,859,859,858,858,854,854,846,846,845,845,843,843,840,840,836,836,831,831,830,830,815,815,812,812,810,810,809,809,802,802,801,801,798,798,796,796,793,793,788,788,787,787,784,784,783,783,782,782,780,780,777,777,772,772,767,767,763,763,762,762,761,761,760,760,758,758,755,755,754,754,751,751,749,749,747,747,742,742,740,740,739,739,737,737,736,736,735,735,734,734,733,733,732,732,731,731,730,730,729,729,728,728,726,726,725,725,723,723,722,722,721,721,720,720,714,714,713,713,710,710,706,706,704,704,703,703,702,702,699,699,697,697,696,696,693,693,692,692,691,691,690,690,688,688,684,684,682,682,681,681,679,679,676,676,672,672,670,670,667,667,664,664,663,663,662,662,660,660,659,659,656,656,651,651,650,650,648,648,646,646,643,643,641,641,639,639,638,638,633,633,632,632,631,631,629,629,628,628,627,627,625,625,624,624,623,623,622,622,620,620,619,619,618,618,616,616,612,612,610,610,609,609,607,607,606,606,604,604,599,599,598,598,597,597,596,596,595,595,594,594,593,593,590,590,589,589,588,588,587,587,585,585,583,583,582,582,581,581,580,580,579,579,578,578,576,576,575,575,574,574,573,573,571,571,570,570,569,569,568,568,567,567,565,565,564,564,562,562,561,561,560,560,558,558,557,557,555,555,552,552,551,551,550,550,549,549,546,546,545,545,543,543,542,542,541,541,540,540,539,539,538,538,536,536,535,535,533,533,532,532,531,531,529,529,527,527,525,525,524,524,522,522,521,521,520,520,519,519,518,518,516,516,515,515,511,511,510,510,509,509,508,508,507,507,505,505,504,504,503,503,502,502,501,501,499,499,498,498,497,497,494,494,493,493,492,492,491,491,489,489,488,488,487,487,486,486,484,484,483,483,480,480,479,479,478,478,477,477,474,474,472,472,470,470,469,469,468,468,467,467,466,466,465,465,464,464,463,463,462,462,461,461,460,460,457,457,455,455,454,454,453,453,452,452,451,451,449,449,448,448,447,447,446,446,445,445,444,444,443,443,439,439,438,438,436,436,434,434,432,432,430,430,429,429,428,428,427,427,424,424,422,422,421,421,420,420,419,419,418,418,417,417,416,416,414,414,412,412,411,411,410,410,409,409,406,406,405,405,403,403,401,401,0.0],[154093,154093,154093,137572,137572,130024,130024,95133,95133,90516,90516,88301,88301,82683,82683,81794,81794,80050,80050,79622,79622,75970,75970,73724,73724,73444,73444,72462,72462,71246,71246,70434,70434,69796,69796,67253,67253,66685,66685,66635,66635,66629,66629,65542,65542,65203,65203,65181,65181,63814,63814,63683,63683,63450,63450,62705,62705,61160,61160,60513,60513,59680,59680,59458,59458,58835,58835,58702,58702,58396,58396,58333,58333,57935,57935,57754,57754,57527,57527,57401,57401,57318,57318,57099,57099,57071,57071,57029,57029,55693,55693,54808,54808,54725,54725,54594,54594,54272,54272,54076,54076,53845,53845,52917,52917,52487,52487,52473,52473,52390,52390,52022,52022,51969,51969,51928,51928,51920,51920,51533,51533,51454,51454,51322,51322,51116,51116,51103,51103,51091,51091,51015,51015,50809,50809,50429,50429,50281,50281,49951,49951,49907,49907,49880,49880,49413,49413,49284,49284,48889,48889,48825,48825,48781,48781,48662,48662,48646,48646,48604,48604,48334,48334,48244,48244,48177,48177,47827,47827,47776,47776,47418,47418,47199,47199,47194,47194,46883,46883,46760,46760,46737,46737,46105,46105,46073,46073,46021,46021,45984,45984,45670,45670,45659,45659,45622,45622,45463,45463,45324,45324,45052,45052,44981,44981,44939,44939,44918,44918,44832,44832,44827,44827,44694,44694,44646,44646,44628,44628,44264,44264,44132,44132,44033,44033,44021,44021,43973,43973,43923,43923,43851,43851,43352,43352,43283,43283,43249,43249,42061,42061,42056,42056,41952,41952,41947,41947,41864,41864,41824,41824,41710,41710,41576,41576,41379,41379,41216,41216,41174,41174,41163,41163,41126,41126,41042,41042,40899,40899,40850,40850,40843,40843,40824,40824,40796,40796,40700,40700,40544,40544,40504,40504,40496,40496,40440,40440,40248,40248,40177,40177,40154,40154,39927,39927,39859,39859,39744,39744,39661,39661,39650,39650,39559,39559,39280,39280,39093,39093,39082,39082,38968,38968,38923,38923,38896,38896,38803,38803,38747,38747,38624,38624,38607,38607,38489,38489,38338,38338,38239,38239,38090,38090,38084,38084,38064,38064,37863,37863,37742,37742,37699,37699,37698,37698,37648,37648,37637,37637,37335,37335,37247,37247,36800,36800,36799,36799,36743,36743,36718,36718,36635,36635,36442,36442,36353,36353,36084,36084,35869,35869,35845,35845,35837,35837,35803,35803,35761,35761,35459,35459,35445,35445,35375,35375,35354,35354,35337,35337,35336,35336,35303,35303,35205,35205,35192,35192,35171,35171,35161,35161,35045,35045,34976,34976,34841,34841,34801,34801,34654,34654,34614,34614,34611,34611,34599,34599,34527,34527,34519,34519,34488,34488,34465,34465,34388,34388,34379,34379,34290,34290,34157,34157,33975,33975,33962,33962,33877,33877,33872,33872,33864,33864,33803,33803,33776,33776,33756,33756,33695,33695,33677,33677,33633,33633,33620,33620,33603,33603,33486,33486,33449,33449,33365,33365,33349,33349,33218,33218,33208,33208,33132,33132,33056,33056,32977,32977,32934,32934,32920,32920,32827,32827,32825,32825,32796,32796,32789,32789,32763,32763,32660,32660,32646,32646,32645,32645,32637,32637,32626,32626,32428,32428,32394,32394,32377,32377,32367,32367,32220,32220,32177,32177,32132,32132,32119,32119,32077,32077,32025,32025,32010,32010,32001,32001,31981,31981,31868,31868,31725,31725,31660,31660,31642,31642,31618,31618,31566,31566,31528,31528,31503,31503,31414,31414,31399,31399,31391,31391,31322,31322,31304,31304,31285,31285,31271,31271,31160,31160,31095,31095,30926,30926,30817,30817,30772,30772,30737,30737,30635,30635,30621,30621,30589,30589,30583,30583,30576,30576,30512,30512,30482,30482,30417,30417,30380,30380,30364,30364,30347,30347,30288,30288,30174,30174,30169,30169,30046,30046,29983,29983,29957,29957,29938,29938,29720,29720,29536,29536,29518,29518,29516,29516,29412,29412,29344,29344,29281,29281,29243,29243,29227,29227,29220,29220,29117,29117,29098,29098,29083,29083,28971,28971,28954,28954,28899,28899,28885,28885,28869,28869,28861,28861,28842,28842,28695,28695,28690,28690,28678,28678,28579,28579,28555,28555,28529,28529,28422,28422,28394,28394,28388,28388,28360,28360,28355,28355,28347,28347,28338,28338,28294,28294,28255,28255,28198,28198,28075,28075,28029,28029,27993,27993,27894,27894,27842,27842,27722,27722,27645,27645,27624,27624,27610,27610,27585,27585,27558,27558,27524,27524,27447,27447,27441,27441,27369,27369,27324,27324,27239,27239,27206,27206,27205,27205,27197,27197,27161,27161,27152,27152,27117,27117,27070,27070,27048,27048,27007,27007,27004,27004,27001,27001,26995,26995,26891,26891,26836,26836,26833,26833,26770,26770,26710,26710,26681,26681,26675,26675,26556,26556,26509,26509,26475,26475,26468,26468,26440,26440,26438,26438,26428,26428,26384,26384,26376,26376,26354,26354,26345,26345,26331,26331,26309,26309,26301,26301,26265,26265,26198,26198,26197,26197,26178,26178,26163,26163,26097,26097,26062,26062,26035,26035,25981,25981,25972,25972,25955,25955,25947,25947,25903,25903,25893,25893,25857,25857,25790,25790,25756,25756,25735,25735,25695,25695,25655,25655,25605,25605,25568,25568,25519,25519,25516,25516,25487,25487,25458,25458,25443,25443,25438,25438,25437,25437,25357,25357,25333,25333,25308,25308,25300,25300,25262,25262,25261,25261,25229,25229,25224,25224,25214,25214,25098,25098,25089,25089,25081,25081,24993,24993,24925,24925,24922,24922,24875,24875,24862,24862,24829,24829,24782,24782,24753,24753,24683,24683,24467,24467,24433,24433,24419,24419,24405,24405,24359,24359,24315,24315,24278,24278,24248,24248,24244,24244,24222,24222,24195,24195,24114,24114,24096,24096,24061,24061,24060,24060,23984,23984,23931,23931,23898,23898,23833,23833,23801,23801,23767,23767,23764,23764,23684,23684,23681,23681,23627,23627,23565,23565,23548,23548,23523,23523,23501,23501,23489,23489,23456,23456,23419,23419,23323,23323,23311,23311,23308,23308,23252,23252,23195,23195,23189,23189,23148,23148,23143,23143,23090,23090,23084,23084,23040,23040,23024,23024,23021,23021,22999,22999,22910,22910,22884,22884,22747,22747,22727,22727,22708,22708,22663,22663,22653,22653,22619,22619,22599,22599,22590,22590,22584,22584,22575,22575,22512,22512,22483,22483,22480,22480,22475,22475,22471,22471,22446,22446,22388,22388,22359,22359,22348,22348,22318,22318,22229,22229,22220,22220,22211,22211,22165,22165,22125,22125,22101,22101,22039,22039,22015,22015,21912,21912,21911,21911,21839,21839,21828,21828,21810,21810,21807,21807,21792,21792,21786,21786,21753,21753,21729,21729,21724,21724,21723,21723,21708,21708,21663,21663,21656,21656,21650,21650,21628,21628,21613,21613,21593,21593,21549,21549,21458,21458,21436,21436,21428,21428,21403,21403,21367,21367,21349,21349,21332,21332,21328,21328,21313,21313,21268,21268,21228,21228,21168,21168,21105,21105,21101,21101,21065,21065,21060,21060,20998,20998,20965,20965,20961,20961,20943,20943,20926,20926,20915,20915,20913,20913,20905,20905,20873,20873,20867,20867,20835,20835,20731,20731,20696,20696,20662,20662,20637,20637,20586,20586,20565,20565,20563,20563,20531,20531,20499,20499,20442,20442,20419,20419,20414,20414,20375,20375,20333,20333,20325,20325,20290,20290,20266,20266,20206,20206,20143,20143,20117,20117,20077,20077,20013,20013,19981,19981,19971,19971,19961,19961,19938,19938,19933,19933,19930,19930,19910,19910,19901,19901,19882,19882,19814,19814,19811,19811,19786,19786,19759,19759,19740,19740,19716,19716,19700,19700,19692,19692,19689,19689,19645,19645,19644,19644,19605,19605,19596,19596,19595,19595,19527,19527,19513,19513,19486,19486,19447,19447,19446,19446,19425,19425,19417,19417,19407,19407,19316,19316,19309,19309,19268,19268,19252,19252,19240,19240,19219,19219,19202,19202,19201,19201,19182,19182,19123,19123,19099,19099,19066,19066,19060,19060,19046,19046,19022,19022,18998,18998,18991,18991,18978,18978,18943,18943,18935,18935,18906,18906,18888,18888,18873,18873,18870,18870,18853,18853,18775,18775,18748,18748,18747,18747,18729,18729,18722,18722,18721,18721,18693,18693,18684,18684,18675,18675,18654,18654,18651,18651,18648,18648,18644,18644,18643,18643,18621,18621,18587,18587,18568,18568,18567,18567,18566,18566,18559,18559,18531,18531,18530,18530,18529,18529,18476,18476,18470,18470,18466,18466,18448,18448,18429,18429,18427,18427,18416,18416,18405,18405,18392,18392,18391,18391,18363,18363,18319,18319,18271,18271,18258,18258,18230,18230,18228,18228,18123,18123,18088,18088,18082,18082,18068,18068,18065,18065,18031,18031,18006,18006,17989,17989,17987,17987,17950,17950,17949,17949,17943,17943,17924,17924,17917,17917,17915,17915,17907,17907,17904,17904,17809,17809,17801,17801,17777,17777,17721,17721,17717,17717,17711,17711,17705,17705,17672,17672,17666,17666,17638,17638,17637,17637,17540,17540,17534,17534,17524,17524,17494,17494,17466,17466,17459,17459,17454,17454,17437,17437,17426,17426,17424,17424,17422,17422,17412,17412,17393,17393,17376,17376,17374,17374,17366,17366,17352,17352,17345,17345,17336,17336,17325,17325,17306,17306,17296,17296,17282,17282,17265,17265,17235,17235,17222,17222,17205,17205,17203,17203,17187,17187,17184,17184,17174,17174,17161,17161,17160,17160,17140,17140,17127,17127,17081,17081,17057,17057,17050,17050,17039,17039,17038,17038,17034,17034,17033,17033,17014,17014,16988,16988,16977,16977,16907,16907,16899,16899,16888,16888,16828,16828,16820,16820,16819,16819,16815,16815,16788,16788,16787,16787,16780,16780,16758,16758,16754,16754,16730,16730,16709,16709,16666,16666,16652,16652,16645,16645,16639,16639,16619,16619,16586,16586,16569,16569,16558,16558,16537,16537,16532,16532,16530,16530,16519,16519,16518,16518,16504,16504,16493,16493,16490,16490,16487,16487,16480,16480,16479,16479,16476,16476,16357,16357,16323,16323,16311,16311,16264,16264,16258,16258,16256,16256,16193,16193,16188,16188,16164,16164,16159,16159,16132,16132,16072,16072,16069,16069,16068,16068,16060,16060,16035,16035,15989,15989,15952,15952,15941,15941,15908,15908,15905,15905,15895,15895,15886,15886,15865,15865,15824,15824,15786,15786,15763,15763,15762,15762,15761,15761,15740,15740,15712,15712,15710,15710,15709,15709,15661,15661,15660,15660,15655,15655,15643,15643,15637,15637,15623,15623,15581,15581,15508,15508,15500,15500,15491,15491,15467,15467,15464,15464,15448,15448,15435,15435,15369,15369,15346,15346,15332,15332,15258,15258,15189,15189,15110,15110,15070,15070,15065,15065,15045,15045,15008,15008,14978,14978,14959,14959,14942,14942,14938,14938,14887,14887,14875,14875,14867,14867,14865,14865,14859,14859,14852,14852,14806,14806,14797,14797,14788,14788,14783,14783,14771,14771,14756,14756,14754,14754,14748,14748,14710,14710,14699,14699,14679,14679,14668,14668,14647,14647,14638,14638,14629,14629,14627,14627,14624,14624,14597,14597,14554,14554,14526,14526,14525,14525,14493,14493,14479,14479,14452,14452,14440,14440,14427,14427,14394,14394,14387,14387,14368,14368,14347,14347,14319,14319,14281,14281,14276,14276,14274,14274,14217,14217,14175,14175,14161,14161,14156,14156,14121,14121,14106,14106,14101,14101,14093,14093,14075,14075,14071,14071,13986,13986,13977,13977,13976,13976,13952,13952,13950,13950,13945,13945,13930,13930,13921,13921,13918,13918,13852,13852,13841,13841,13813,13813,13802,13802,13796,13796,13787,13787,13775,13775,13754,13754,13734,13734,13731,13731,13720,13720,13719,13719,13703,13703,13701,13701,13695,13695,13694,13694,13690,13690,13683,13683,13682,13682,13657,13657,13649,13649,13634,13634,13631,13631,13629,13629,13616,13616,13601,13601,13554,13554,13542,13542,13540,13540,13528,13528,13520,13520,13462,13462,13438,13438,13413,13413,13369,13369,13335,13335,13331,13331,13322,13322,13306,13306,13299,13299,13289,13289,13254,13254,13252,13252,13240,13240,13179,13179,13175,13175,13152,13152,13151,13151,13102,13102,13094,13094,13092,13092,13077,13077,13053,13053,13011,13011,13009,13009,12997,12997,12989,12989,12981,12981,12970,12970,12955,12955,12947,12947,12926,12926,12901,12901,12897,12897,12847,12847,12837,12837,12832,12832,12830,12830,12820,12820,12818,12818,12785,12785,12772,12772,12753,12753,12751,12751,12733,12733,12729,12729,12726,12726,12715,12715,12701,12701,12697,12697,12655,12655,12634,12634,12613,12613,12596,12596,12592,12592,12588,12588,12564,12564,12558,12558,12549,12549,12536,12536,12503,12503,12495,12495,12487,12487,12486,12486,12432,12432,12428,12428,12402,12402,12396,12396,12389,12389,12385,12385,12375,12375,12371,12371,12348,12348,12340,12340,12338,12338,12337,12337,12331,12331,12326,12326,12303,12303,12301,12301,12295,12295,12292,12292,12287,12287,12276,12276,12227,12227,12213,12213,12198,12198,12177,12177,12159,12159,12139,12139,12117,12117,12105,12105,12085,12085,12079,12079,12075,12075,12070,12070,12052,12052,12050,12050,12046,12046,12044,12044,12042,12042,12032,12032,12030,12030,12011,12011,12000,12000,11991,11991,11979,11979,11978,11978,11962,11962,11943,11943,11928,11928,11918,11918,11915,11915,11914,11914,11911,11911,11906,11906,11905,11905,11895,11895,11893,11893,11888,11888,11882,11882,11872,11872,11865,11865,11859,11859,11854,11854,11828,11828,11825,11825,11772,11772,11755,11755,11744,11744,11693,11693,11682,11682,11680,11680,11673,11673,11665,11665,11657,11657,11655,11655,11632,11632,11615,11615,11610,11610,11606,11606,11597,11597,11594,11594,11587,11587,11582,11582,11554,11554,11537,11537,11535,11535,11527,11527,11512,11512,11501,11501,11500,11500,11490,11490,11484,11484,11482,11482,11478,11478,11471,11471,11469,11469,11443,11443,11386,11386,11382,11382,11380,11380,11366,11366,11346,11346,11334,11334,11332,11332,11322,11322,11294,11294,11292,11292,11267,11267,11264,11264,11231,11231,11216,11216,11206,11206,11201,11201,11196,11196,11191,11191,11171,11171,11153,11153,11152,11152,11130,11130,11127,11127,11124,11124,11092,11092,11083,11083,11082,11082,11069,11069,11061,11061,11047,11047,11018,11018,11011,11011,10986,10986,10972,10972,10966,10966,10952,10952,10946,10946,10930,10930,10926,10926,10908,10908,10897,10897,10891,10891,10843,10843,10842,10842,10813,10813,10797,10797,10791,10791,10782,10782,10779,10779,10766,10766,10761,10761,10760,10760,10759,10759,10744,10744,10726,10726,10725,10725,10722,10722,10721,10721,10684,10684,10679,10679,10668,10668,10616,10616,10615,10615,10605,10605,10589,10589,10566,10566,10564,10564,10557,10557,10549,10549,10543,10543,10541,10541,10540,10540,10532,10532,10518,10518,10515,10515,10507,10507,10502,10502,10492,10492,10490,10490,10474,10474,10462,10462,10447,10447,10441,10441,10440,10440,10423,10423,10415,10415,10414,10414,10389,10389,10373,10373,10368,10368,10355,10355,10351,10351,10348,10348,10343,10343,10310,10310,10296,10296,10283,10283,10277,10277,10266,10266,10265,10265,10262,10262,10255,10255,10232,10232,10226,10226,10202,10202,10197,10197,10189,10189,10185,10185,10175,10175,10173,10173,10138,10138,10136,10136,10131,10131,10112,10112,10110,10110,10108,10108,10107,10107,10104,10104,10097,10097,10081,10081,10078,10078,10057,10057,10047,10047,10043,10043,10035,10035,10030,10030,10007,10007,9989,9989,9986,9986,9977,9977,9966,9966,9937,9937,9929,9929,9908,9908,9896,9896,9878,9878,9866,9866,9864,9864,9819,9819,9813,9813,9808,9808,9805,9805,9791,9791,9781,9781,9772,9772,9762,9762,9760,9760,9736,9736,9723,9723,9717,9717,9704,9704,9701,9701,9696,9696,9689,9689,9681,9681,9675,9675,9667,9667,9636,9636,9631,9631,9616,9616,9614,9614,9594,9594,9580,9580,9568,9568,9565,9565,9559,9559,9549,9549,9545,9545,9542,9542,9534,9534,9532,9532,9527,9527,9526,9526,9523,9523,9520,9520,9516,9516,9511,9511,9505,9505,9500,9500,9478,9478,9476,9476,9466,9466,9459,9459,9458,9458,9434,9434,9430,9430,9428,9428,9409,9409,9403,9403,9392,9392,9387,9387,9379,9379,9370,9370,9367,9367,9354,9354,9349,9349,9346,9346,9328,9328,9325,9325,9302,9302,9301,9301,9299,9299,9282,9282,9248,9248,9237,9237,9204,9204,9179,9179,9171,9171,9166,9166,9162,9162,9161,9161,9155,9155,9147,9147,9144,9144,9101,9101,9100,9100,9094,9094,9088,9088,9085,9085,9083,9083,9078,9078,9065,9065,9057,9057,9054,9054,9050,9050,9040,9040,9039,9039,8998,8998,8995,8995,8948,8948,8946,8946,8932,8932,8930,8930,8925,8925,8915,8915,8914,8914,8905,8905,8901,8901,8887,8887,8884,8884,8874,8874,8862,8862,8857,8857,8844,8844,8834,8834,8830,8830,8828,8828,8814,8814,8786,8786,8763,8763,8760,8760,8753,8753,8752,8752,8748,8748,8743,8743,8739,8739,8709,8709,8707,8707,8701,8701,8696,8696,8683,8683,8663,8663,8659,8659,8655,8655,8649,8649,8639,8639,8617,8617,8584,8584,8567,8567,8565,8565,8545,8545,8532,8532,8519,8519,8498,8498,8493,8493,8488,8488,8476,8476,8467,8467,8466,8466,8459,8459,8454,8454,8440,8440,8436,8436,8422,8422,8420,8420,8411,8411,8408,8408,8393,8393,8383,8383,8380,8380,8369,8369,8358,8358,8342,8342,8341,8341,8331,8331,8323,8323,8319,8319,8315,8315,8303,8303,8302,8302,8297,8297,8285,8285,8283,8283,8282,8282,8280,8280,8279,8279,8271,8271,8241,8241,8236,8236,8225,8225,8224,8224,8218,8218,8216,8216,8204,8204,8198,8198,8186,8186,8181,8181,8149,8149,8109,8109,8106,8106,8105,8105,8097,8097,8092,8092,8077,8077,8017,8017,8006,8006,8005,8005,7986,7986,7970,7970,7947,7947,7945,7945,7911,7911,7908,7908,7892,7892,7888,7888,7862,7862,7859,7859,7855,7855,7843,7843,7805,7805,7803,7803,7801,7801,7798,7798,7776,7776,7769,7769,7761,7761,7757,7757,7739,7739,7733,7733,7732,7732,7727,7727,7726,7726,7709,7709,7705,7705,7690,7690,7679,7679,7673,7673,7639,7639,7633,7633,7615,7615,7613,7613,7611,7611,7584,7584,7512,7512,7504,7504,7497,7497,7496,7496,7476,7476,7471,7471,7442,7442,7428,7428,7426,7426,7414,7414,7402,7402,7388,7388,7383,7383,7378,7378,7369,7369,7368,7368,7362,7362,7361,7361,7352,7352,7339,7339,7318,7318,7314,7314,7283,7283,7280,7280,7279,7279,7278,7278,7265,7265,7263,7263,7262,7262,7255,7255,7254,7254,7251,7251,7250,7250,7247,7247,7243,7243,7235,7235,7232,7232,7230,7230,7229,7229,7223,7223,7213,7213,7207,7207,7184,7184,7182,7182,7174,7174,7146,7146,7141,7141,7127,7127,7085,7085,7084,7084,7071,7071,7067,7067,7052,7052,7050,7050,7042,7042,7029,7029,7018,7018,7014,7014,6981,6981,6976,6976,6972,6972,6971,6971,6969,6969,6965,6965,6962,6962,6955,6955,6954,6954,6936,6936,6932,6932,6930,6930,6928,6928,6925,6925,6921,6921,6868,6868,6846,6846,6840,6840,6831,6831,6825,6825,6810,6810,6804,6804,6798,6798,6784,6784,6775,6775,6761,6761,6753,6753,6746,6746,6726,6726,6724,6724,6701,6701,6698,6698,6686,6686,6664,6664,6659,6659,6629,6629,6628,6628,6623,6623,6622,6622,6611,6611,6606,6606,6604,6604,6603,6603,6601,6601,6576,6576,6575,6575,6567,6567,6562,6562,6561,6561,6553,6553,6549,6549,6530,6530,6529,6529,6516,6516,6504,6504,6488,6488,6485,6485,6480,6480,6464,6464,6425,6425,6422,6422,6421,6421,6407,6407,6387,6387,6374,6374,6363,6363,6354,6354,6342,6342,6336,6336,6333,6333,6328,6328,6295,6295,6290,6290,6286,6286,6274,6274,6266,6266,6251,6251,6248,6248,6245,6245,6236,6236,6235,6235,6226,6226,6224,6224,6214,6214,6213,6213,6211,6211,6208,6208,6152,6152,6148,6148,6125,6125,6119,6119,6116,6116,6109,6109,6072,6072,6050,6050,6044,6044,6043,6043,6038,6038,6037,6037,6032,6032,6029,6029,6020,6020,6016,6016,6002,6002,5989,5989,5986,5986,5975,5975,5974,5974,5953,5953,5942,5942,5928,5928,5927,5927,5926,5926,5918,5918,5915,5915,5899,5899,5898,5898,5896,5896,5895,5895,5882,5882,5879,5879,5876,5876,5873,5873,5865,5865,5858,5858,5857,5857,5854,5854,5827,5827,5825,5825,5819,5819,5793,5793,5784,5784,5778,5778,5774,5774,5771,5771,5767,5767,5756,5756,5755,5755,5753,5753,5751,5751,5727,5727,5710,5710,5709,5709,5705,5705,5690,5690,5685,5685,5665,5665,5657,5657,5632,5632,5631,5631,5606,5606,5570,5570,5568,5568,5559,5559,5557,5557,5541,5541,5521,5521,5516,5516,5513,5513,5510,5510,5504,5504,5502,5502,5478,5478,5474,5474,5473,5473,5466,5466,5459,5459,5456,5456,5454,5454,5442,5442,5441,5441,5430,5430,5413,5413,5402,5402,5397,5397,5384,5384,5378,5378,5366,5366,5365,5365,5358,5358,5337,5337,5324,5324,5319,5319,5317,5317,5306,5306,5303,5303,5295,5295,5287,5287,5282,5282,5272,5272,5265,5265,5256,5256,5239,5239,5238,5238,5232,5232,5226,5226,5212,5212,5202,5202,5192,5192,5167,5167,5159,5159,5141,5141,5139,5139,5134,5134,5131,5131,5095,5095,5085,5085,5062,5062,5052,5052,5046,5046,5043,5043,5039,5039,5025,5025,5021,5021,4999,4999,4979,4979,4976,4976,4974,4974,4963,4963,4957,4957,4947,4947,4935,4935,4920,4920,4907,4907,4906,4906,4890,4890,4883,4883,4877,4877,4874,4874,4858,4858,4854,4854,4847,4847,4846,4846,4842,4842,4839,4839,4837,4837,4832,4832,4813,4813,4808,4808,4778,4778,4776,4776,4774,4774,4769,4769,4766,4766,4754,4754,4736,4736,4734,4734,4713,4713,4700,4700,4675,4675,4669,4669,4668,4668,4663,4663,4657,4657,4626,4626,4620,4620,4613,4613,4601,4601,4592,4592,4588,4588,4585,4585,4584,4584,4579,4579,4572,4572,4551,4551,4547,4547,4543,4543,4539,4539,4537,4537,4514,4514,4478,4478,4462,4462,4458,4458,4457,4457,4454,4454,4419,4419,4403,4403,4398,4398,4386,4386,4381,4381,4371,4371,4354,4354,4347,4347,4344,4344,4340,4340,4323,4323,4306,4306,4279,4279,4273,4273,4266,4266,4260,4260,4258,4258,4251,4251,4246,4246,4245,4245,4242,4242,4238,4238,4231,4231,4228,4228,4225,4225,4213,4213,4212,4212,4187,4187,4153,4153,4142,4142,4138,4138,4122,4122,4112,4112,4111,4111,4110,4110,4100,4100,4077,4077,4060,4060,4055,4055,4049,4049,4045,4045,4021,4021,4018,4018,4011,4011,3999,3999,3991,3991,3978,3978,3977,3977,3974,3974,3968,3968,3967,3967,3954,3954,3939,3939,3934,3934,3926,3926,3924,3924,3920,3920,3910,3910,3904,3904,3894,3894,3853,3853,3830,3830,3822,3822,3818,3818,3814,3814,3805,3805,3794,3794,3780,3780,3774,3774,3719,3719,3702,3702,3700,3700,3696,3696,3689,3689,3681,3681,3649,3649,3635,3635,3624,3624,3619,3619,3613,3613,3612,3612,3606,3606,3603,3603,3602,3602,3592,3592,3580,3580,3577,3577,3571,3571,3566,3566,3565,3565,3561,3561,3554,3554,3549,3549,3545,3545,3542,3542,3539,3539,3535,3535,3530,3530,3510,3510,3508,3508,3468,3468,3458,3458,3449,3449,3437,3437,3434,3434,3428,3428,3414,3414,3393,3393,3380,3380,3362,3362,3353,3353,3348,3348,3340,3340,3316,3316,3308,3308,3303,3303,3281,3281,3263,3263,3252,3252,3247,3247,3246,3246,3230,3230,3213,3213,3202,3202,3190,3190,3177,3177,3166,3166,3165,3165,3156,3156,3149,3149,3132,3132,3129,3129,3120,3120,3119,3119,3117,3117,3106,3106,3100,3100,3095,3095,3086,3086,3057,3057,3055,3055,3048,3048,3040,3040,3027,3027,3019,3019,3006,3006,2990,2990,2971,2971,2957,2957,2934,2934,2928,2928,2917,2917,2913,2913,2882,2882,2871,2871,2870,2870,2863,2863,2852,2852,2845,2845,2828,2828,2826,2826,2804,2804,2800,2800,2793,2793,2758,2758,2755,2755,2747,2747,2742,2742,2718,2718,2703,2703,2698,2698,2696,2696,2659,2659,2656,2656,2642,2642,2635,2635,2627,2627,2626,2626,2618,2618,2613,2613,2599,2599,2596,2596,2570,2570,2547,2547,2483,2483,2459,2459,2456,2456,2451,2451,2448,2448,2436,2436,2429,2429,2428,2428,2419,2419,2404,2404,2400,2400,2389,2389,2388,2388,2376,2376,2375,2375,2373,2373,2359,2359,2349,2349,2338,2338,2323,2323,2319,2319,2306,2306,2275,2275,2266,2266,2265,2265,2264,2264,2261,2261,2256,2256,2223,2223,2222,2222,2220,2220,2206,2206,2202,2202,2192,2192,2179,2179,2176,2176,2135,2135,2133,2133,2120,2120,2118,2118,2117,2117,2109,2109,2106,2106,2103,2103,2089,2089,2085,2085,2082,2082,2077,2077,2070,2070,2035,2035,2027,2027,2020,2020,2019,2019,2012,2012,2006,2006,2003,2003,1946,1946,1943,1943,1935,1935,1926,1926,1921,1921,1910,1910,1907,1907,1902,1902,1898,1898,1889,1889,1866,1866,1863,1863,1862,1862,1835,1835,1828,1828,1826,1826,1819,1819,1810,1810,1799,1799,1795,1795,1773,1773,1765,1765,1741,1741,1737,1737,1734,1734,1729,1729,1728,1728,1725,1725,1706,1706,1704,1704,1698,1698,1694,1694,1691,1691,1667,1667,1661,1661,1656,1656,1646,1646,1641,1641,1626,1626,1607,1607,1605,1605,1590,1590,1587,1587,1586,1586,1566,1566,1560,1560,1555,1555,1553,1553,1549,1549,1542,1542,1534,1534,1532,1532,1530,1530,1523,1523,1519,1519,1505,1505,1472,1472,1467,1467,1439,1439,1436,1436,1417,1417,1413,1413,1407,1407,1404,1404,1403,1403,1399,1399,1398,1398,1374,1374,1364,1364,1340,1340,1337,1337,1335,1335,1334,1334,1331,1331,1329,1329,1317,1317,1309,1309,1292,1292,1288,1288,1282,1282,1277,1277,1269,1269,1260,1260,1246,1246,1244,1244,1242,1242,1239,1239,1233,1233,1216,1216,1206,1206,1197,1197,1192,1192,1183,1183,1163,1163,1161,1161,1160,1160,1158,1158,1155,1155,1152,1152,1149,1149,1146,1146,1129,1129,1124,1124,1105,1105,1103,1103,1091,1091,1065,1065,1062,1062,1059,1059,1045,1045,1035,1035,1031,1031,1029,1029,1025,1025,1013,1013,1006,1006,1000,1000,995,995,989,989,987,987,984,984,981,981,968,968,964,964,958,958,954,954,951,951,948,948,942,942,933,933,932,932,923,923,921,921,919,919,917,917,914,914,904,904,899,899,895,895,889,889,879,879,869,869,868,868,867,867,865,865,847,847,845,845,843,843,840,840,823,823,821,821,812,812,809,809,806,806,804,804,798,798,796,796,794,794,793,793,784,784,782,782,780,780,771,771,770,770,769,769,768,768,762,762,757,757,742,742,739,739,735,735,734,734,731,731,726,726,725,725,715,715,713,713,711,711,704,704,702,702,701,701,699,699,696,696,695,695,690,690,688,688,687,687,686,686,685,685,679,679,678,678,672,672,671,671,670,670,663,663,658,658,652,652,651,651,650,650,648,648,630,630,629,629,627,627,625,625,624,624,623,623,616,616,614,614,612,612,607,607,597,597,595,595,590,590,589,589,581,581,580,580,577,577,576,576,574,574,572,572,571,571,570,570,569,569,568,568,564,564,559,559,558,558,555,555,543,543,537,537,536,536,524,524,521,521,517,517,508,508,506,506,505,505,501,501,499,499,493,493,489,489,487,487,480,480,477,477,472,472,469,469,468,468,467,467,466,466,464,464,462,462,460,460,457,457,453,453,449,449,448,448,447,447,444,444,439,439,438,438,436,436,435,435,432,432,430,430,429,429,427,427,424,424,422,422,420,420,419,419,418,418,417,417,412,412,410,410,406,406,405,405,404,404,402,402,0.0],[20600,20600,20600,15183,15183,14265,14265,13981,13981,13628,13628,13415,13415,13076,13076,12895,12895,12760,12760,12680,12680,12227,12227,12200,12200,12109,12109,12079,12079,11815,11815,11802,11802,11657,11657,11570,11570,11552,11552,11525,11525,11513,11513,11415,11415,11284,11284,11176,11176,11166,11166,11156,11156,11098,11098,11059,11059,10962,10962,10940,10940,10796,10796,10667,10667,10624,10624,10579,10579,10535,10535,10510,10510,10473,10473,10466,10466,10414,10414,10379,10379,10312,10312,10306,10306,10282,10282,10234,10234,10117,10117,10084,10084,10071,10071,10046,10046,9998,9998,9976,9976,9961,9961,9960,9960,9907,9907,9899,9899,9898,9898,9848,9848,9828,9828,9826,9826,9815,9815,9770,9770,9746,9746,9722,9722,9648,9648,9599,9599,9576,9576,9516,9516,9506,9506,9497,9497,9492,9492,9479,9479,9447,9447,9393,9393,9388,9388,9385,9385,9368,9368,9337,9337,9332,9332,9327,9327,9314,9314,9292,9292,9202,9202,9151,9151,9142,9142,9116,9116,9100,9100,9078,9078,9054,9054,9052,9052,9040,9040,9005,9005,8998,8998,8966,8966,8965,8965,8949,8949,8936,8936,8911,8911,8906,8906,8893,8893,8892,8892,8878,8878,8859,8859,8846,8846,8842,8842,8829,8829,8826,8826,8787,8787,8721,8721,8717,8717,8716,8716,8703,8703,8696,8696,8695,8695,8685,8685,8670,8670,8656,8656,8655,8655,8633,8633,8625,8625,8621,8621,8620,8620,8612,8612,8610,8610,8607,8607,8591,8591,8588,8588,8580,8580,8568,8568,8564,8564,8562,8562,8553,8553,8534,8534,8520,8520,8507,8507,8503,8503,8480,8480,8476,8476,8474,8474,8466,8466,8465,8465,8463,8463,8457,8457,8442,8442,8437,8437,8435,8435,8430,8430,8422,8422,8418,8418,8384,8384,8377,8377,8376,8376,8371,8371,8362,8362,8346,8346,8343,8343,8335,8335,8313,8313,8288,8288,8275,8275,8255,8255,8237,8237,8226,8226,8218,8218,8208,8208,8205,8205,8201,8201,8196,8196,8193,8193,8178,8178,8174,8174,8168,8168,8167,8167,8165,8165,8154,8154,8146,8146,8143,8143,8122,8122,8115,8115,8107,8107,8093,8093,8088,8088,8083,8083,8068,8068,8055,8055,8051,8051,8041,8041,8036,8036,8010,8010,7981,7981,7977,7977,7976,7976,7955,7955,7954,7954,7906,7906,7888,7888,7887,7887,7885,7885,7872,7872,7858,7858,7849,7849,7847,7847,7846,7846,7835,7835,7823,7823,7807,7807,7806,7806,7803,7803,7790,7790,7786,7786,7785,7785,7783,7783,7773,7773,7767,7767,7766,7766,7763,7763,7759,7759,7758,7758,7753,7753,7752,7752,7733,7733,7728,7728,7722,7722,7691,7691,7675,7675,7674,7674,7670,7670,7667,7667,7664,7664,7649,7649,7643,7643,7623,7623,7619,7619,7616,7616,7591,7591,7585,7585,7578,7578,7575,7575,7574,7574,7571,7571,7569,7569,7559,7559,7556,7556,7554,7554,7541,7541,7524,7524,7523,7523,7522,7522,7512,7512,7508,7508,7507,7507,7506,7506,7505,7505,7502,7502,7499,7499,7498,7498,7497,7497,7489,7489,7480,7480,7474,7474,7465,7465,7455,7455,7453,7453,7449,7449,7446,7446,7438,7438,7436,7436,7423,7423,7420,7420,7414,7414,7396,7396,7382,7382,7380,7380,7377,7377,7373,7373,7370,7370,7368,7368,7363,7363,7353,7353,7342,7342,7337,7337,7327,7327,7323,7323,7319,7319,7308,7308,7303,7303,7298,7298,7296,7296,7283,7283,7282,7282,7275,7275,7272,7272,7267,7267,7265,7265,7260,7260,7248,7248,7243,7243,7228,7228,7226,7226,7219,7219,7218,7218,7217,7217,7215,7215,7209,7209,7203,7203,7196,7196,7193,7193,7192,7192,7181,7181,7152,7152,7151,7151,7142,7142,7137,7137,7127,7127,7125,7125,7122,7122,7120,7120,7117,7117,7105,7105,7104,7104,7101,7101,7098,7098,7094,7094,7078,7078,7074,7074,7069,7069,7068,7068,7065,7065,7058,7058,7055,7055,7054,7054,7051,7051,7050,7050,7032,7032,7028,7028,7015,7015,7011,7011,7007,7007,7002,7002,6994,6994,6981,6981,6974,6974,6965,6965,6956,6956,6948,6948,6946,6946,6945,6945,6932,6932,6931,6931,6921,6921,6919,6919,6918,6918,6915,6915,6914,6914,6910,6910,6895,6895,6885,6885,6874,6874,6857,6857,6855,6855,6853,6853,6852,6852,6849,6849,6848,6848,6847,6847,6846,6846,6844,6844,6843,6843,6836,6836,6834,6834,6831,6831,6830,6830,6828,6828,6813,6813,6809,6809,6807,6807,6804,6804,6796,6796,6793,6793,6784,6784,6777,6777,6775,6775,6766,6766,6760,6760,6759,6759,6756,6756,6754,6754,6751,6751,6747,6747,6746,6746,6745,6745,6738,6738,6735,6735,6725,6725,6723,6723,6722,6722,6716,6716,6707,6707,6703,6703,6699,6699,6685,6685,6684,6684,6683,6683,6682,6682,6677,6677,6672,6672,6669,6669,6661,6661,6660,6660,6655,6655,6654,6654,6653,6653,6651,6651,6645,6645,6644,6644,6638,6638,6629,6629,6628,6628,6621,6621,6620,6620,6619,6619,6618,6618,6617,6617,6614,6614,6612,6612,6606,6606,6603,6603,6602,6602,6595,6595,6591,6591,6585,6585,6579,6579,6576,6576,6570,6570,6566,6566,6564,6564,6563,6563,6562,6562,6557,6557,6555,6555,6550,6550,6549,6549,6537,6537,6536,6536,6523,6523,6520,6520,6502,6502,6501,6501,6498,6498,6492,6492,6490,6490,6488,6488,6485,6485,6482,6482,6474,6474,6472,6472,6469,6469,6468,6468,6466,6466,6465,6465,6451,6451,6448,6448,6442,6442,6441,6441,6439,6439,6438,6438,6437,6437,6428,6428,6419,6419,6414,6414,6400,6400,6395,6395,6391,6391,6387,6387,6383,6383,6382,6382,6379,6379,6375,6375,6374,6374,6371,6371,6370,6370,6366,6366,6364,6364,6355,6355,6352,6352,6350,6350,6349,6349,6348,6348,6347,6347,6344,6344,6341,6341,6337,6337,6336,6336,6335,6335,6334,6334,6330,6330,6328,6328,6323,6323,6318,6318,6317,6317,6314,6314,6313,6313,6307,6307,6305,6305,6304,6304,6300,6300,6299,6299,6279,6279,6278,6278,6276,6276,6261,6261,6260,6260,6254,6254,6246,6246,6239,6239,6221,6221,6218,6218,6216,6216,6209,6209,6208,6208,6204,6204,6193,6193,6192,6192,6191,6191,6186,6186,6180,6180,6169,6169,6165,6165,6162,6162,6160,6160,6154,6154,6153,6153,6149,6149,6145,6145,6138,6138,6137,6137,6135,6135,6133,6133,6128,6128,6125,6125,6113,6113,6111,6111,6110,6110,6109,6109,6107,6107,6105,6105,6104,6104,6103,6103,6102,6102,6100,6100,6096,6096,6092,6092,6086,6086,6084,6084,6081,6081,6080,6080,6079,6079,6077,6077,6076,6076,6072,6072,6071,6071,6061,6061,6049,6049,6047,6047,6042,6042,6037,6037,6026,6026,6023,6023,6022,6022,6017,6017,6016,6016,6013,6013,6008,6008,6005,6005,6004,6004,6003,6003,5997,5997,5993,5993,5991,5991,5986,5986,5983,5983,5979,5979,5974,5974,5973,5973,5972,5972,5964,5964,5963,5963,5958,5958,5955,5955,5953,5953,5951,5951,5949,5949,5938,5938,5931,5931,5927,5927,5925,5925,5923,5923,5909,5909,5907,5907,5902,5902,5900,5900,5899,5899,5898,5898,5897,5897,5894,5894,5881,5881,5880,5880,5879,5879,5878,5878,5876,5876,5863,5863,5862,5862,5861,5861,5857,5857,5855,5855,5853,5853,5848,5848,5845,5845,5844,5844,5841,5841,5839,5839,5838,5838,5834,5834,5831,5831,5825,5825,5824,5824,5821,5821,5820,5820,5819,5819,5818,5818,5813,5813,5811,5811,5806,5806,5801,5801,5800,5800,5799,5799,5797,5797,5796,5796,5793,5793,5792,5792,5791,5791,5781,5781,5778,5778,5777,5777,5775,5775,5769,5769,5768,5768,5767,5767,5766,5766,5765,5765,5762,5762,5759,5759,5756,5756,5754,5754,5752,5752,5750,5750,5749,5749,5748,5748,5747,5747,5746,5746,5741,5741,5738,5738,5737,5737,5736,5736,5731,5731,5728,5728,5726,5726,5724,5724,5723,5723,5720,5720,5719,5719,5718,5718,5717,5717,5711,5711,5707,5707,5705,5705,5699,5699,5697,5697,5685,5685,5684,5684,5680,5680,5679,5679,5676,5676,5672,5672,5667,5667,5664,5664,5663,5663,5661,5661,5658,5658,5655,5655,5646,5646,5642,5642,5641,5641,5635,5635,5631,5631,5629,5629,5626,5626,5625,5625,5619,5619,5618,5618,5611,5611,5607,5607,5604,5604,5599,5599,5596,5596,5592,5592,5591,5591,5589,5589,5583,5583,5581,5581,5580,5580,5577,5577,5575,5575,5571,5571,5569,5569,5564,5564,5563,5563,5562,5562,5561,5561,5560,5560,5559,5559,5555,5555,5549,5549,5547,5547,5543,5543,5541,5541,5538,5538,5537,5537,5534,5534,5526,5526,5524,5524,5523,5523,5517,5517,5516,5516,5514,5514,5512,5512,5511,5511,5509,5509,5508,5508,5507,5507,5506,5506,5504,5504,5503,5503,5500,5500,5496,5496,5494,5494,5493,5493,5490,5490,5488,5488,5480,5480,5476,5476,5473,5473,5472,5472,5470,5470,5469,5469,5468,5468,5466,5466,5465,5465,5462,5462,5461,5461,5460,5460,5456,5456,5455,5455,5453,5453,5452,5452,5451,5451,5447,5447,5445,5445,5441,5441,5439,5439,5437,5437,5436,5436,5432,5432,5429,5429,5428,5428,5426,5426,5425,5425,5418,5418,5417,5417,5416,5416,5415,5415,5414,5414,5413,5413,5410,5410,5409,5409,5408,5408,5407,5407,5406,5406,5405,5405,5404,5404,5399,5399,5398,5398,5397,5397,5395,5395,5389,5389,5387,5387,5386,5386,5385,5385,5383,5383,5380,5380,5377,5377,5375,5375,5374,5374,5373,5373,5368,5368,5363,5363,5361,5361,5354,5354,5353,5353,5352,5352,5348,5348,5346,5346,5342,5342,5339,5339,5338,5338,5330,5330,5329,5329,5326,5326,5323,5323,5320,5320,5319,5319,5318,5318,5312,5312,5309,5309,5308,5308,5304,5304,5303,5303,5300,5300,5297,5297,5290,5290,5288,5288,5285,5285,5284,5284,5283,5283,5280,5280,5279,5279,5277,5277,5271,5271,5270,5270,5268,5268,5266,5266,5265,5265,5264,5264,5258,5258,5257,5257,5255,5255,5254,5254,5253,5253,5251,5251,5250,5250,5244,5244,5241,5241,5240,5240,5237,5237,5236,5236,5235,5235,5234,5234,5233,5233,5232,5232,5229,5229,5228,5228,5227,5227,5226,5226,5222,5222,5221,5221,5218,5218,5217,5217,5214,5214,5209,5209,5201,5201,5200,5200,5199,5199,5192,5192,5185,5185,5182,5182,5179,5179,5178,5178,5170,5170,5169,5169,5168,5168,5165,5165,5164,5164,5163,5163,5162,5162,5160,5160,5159,5159,5157,5157,5154,5154,5153,5153,5152,5152,5151,5151,5149,5149,5148,5148,5144,5144,5142,5142,5141,5141,5139,5139,5136,5136,5135,5135,5133,5133,5132,5132,5131,5131,5126,5126,5121,5121,5120,5120,5117,5117,5114,5114,5111,5111,5110,5110,5109,5109,5107,5107,5103,5103,5102,5102,5100,5100,5097,5097,5096,5096,5095,5095,5093,5093,5092,5092,5090,5090,5089,5089,5087,5087,5084,5084,5083,5083,5080,5080,5073,5073,5068,5068,5065,5065,5064,5064,5063,5063,5060,5060,5057,5057,5056,5056,5055,5055,5054,5054,5051,5051,5050,5050,5049,5049,5047,5047,5045,5045,5044,5044,5031,5031,5030,5030,5023,5023,5022,5022,5021,5021,5018,5018,5013,5013,5011,5011,5010,5010,5008,5008,5007,5007,5006,5006,5004,5004,5003,5003,5002,5002,5001,5001,4997,4997,4993,4993,4989,4989,4988,4988,4987,4987,4984,4984,4983,4983,4982,4982,4981,4981,4979,4979,4975,4975,4974,4974,4973,4973,4972,4972,4971,4971,4964,4964,4960,4960,4959,4959,4957,4957,4955,4955,4951,4951,4950,4950,4949,4949,4946,4946,4945,4945,4944,4944,4942,4942,4941,4941,4940,4940,4939,4939,4935,4935,4934,4934,4931,4931,4928,4928,4925,4925,4923,4923,4922,4922,4918,4918,4913,4913,4912,4912,4911,4911,4909,4909,4908,4908,4907,4907,4906,4906,4904,4904,4903,4903,4902,4902,4899,4899,4897,4897,4893,4893,4892,4892,4891,4891,4885,4885,4883,4883,4882,4882,4880,4880,4877,4877,4874,4874,4873,4873,4872,4872,4871,4871,4870,4870,4869,4869,4867,4867,4866,4866,4862,4862,4859,4859,4854,4854,4853,4853,4851,4851,4850,4850,4845,4845,4844,4844,4843,4843,4840,4840,4835,4835,4834,4834,4832,4832,4831,4831,4830,4830,4829,4829,4827,4827,4825,4825,4822,4822,4821,4821,4818,4818,4817,4817,4811,4811,4807,4807,4806,4806,4805,4805,4804,4804,4799,4799,4795,4795,4794,4794,4790,4790,4789,4789,4788,4788,4786,4786,4785,4785,4784,4784,4782,4782,4775,4775,4773,4773,4772,4772,4770,4770,4769,4769,4767,4767,4764,4764,4762,4762,4760,4760,4759,4759,4756,4756,4755,4755,4754,4754,4753,4753,4751,4751,4750,4750,4747,4747,4746,4746,4741,4741,4740,4740,4738,4738,4737,4737,4735,4735,4732,4732,4731,4731,4729,4729,4727,4727,4726,4726,4723,4723,4721,4721,4719,4719,4718,4718,4716,4716,4714,4714,4713,4713,4710,4710,4708,4708,4707,4707,4702,4702,4701,4701,4699,4699,4698,4698,4697,4697,4696,4696,4694,4694,4691,4691,4690,4690,4688,4688,4685,4685,4684,4684,4677,4677,4672,4672,4671,4671,4670,4670,4669,4669,4665,4665,4664,4664,4661,4661,4659,4659,4658,4658,4657,4657,4656,4656,4654,4654,4653,4653,4647,4647,4645,4645,4641,4641,4639,4639,4637,4637,4636,4636,4635,4635,4632,4632,4629,4629,4625,4625,4624,4624,4623,4623,4622,4622,4620,4620,4619,4619,4617,4617,4615,4615,4614,4614,4613,4613,4608,4608,4607,4607,4606,4606,4605,4605,4603,4603,4602,4602,4601,4601,4600,4600,4597,4597,4596,4596,4595,4595,4593,4593,4592,4592,4591,4591,4590,4590,4586,4586,4585,4585,4582,4582,4581,4581,4580,4580,4579,4579,4576,4576,4575,4575,4574,4574,4573,4573,4572,4572,4571,4571,4568,4568,4567,4567,4566,4566,4565,4565,4563,4563,4562,4562,4557,4557,4556,4556,4555,4555,4553,4553,4552,4552,4550,4550,4548,4548,4547,4547,4545,4545,4542,4542,4537,4537,4536,4536,4535,4535,4531,4531,4530,4530,4529,4529,4526,4526,4525,4525,4521,4521,4519,4519,4518,4518,4514,4514,4513,4513,4510,4510,4508,4508,4507,4507,4506,4506,4504,4504,4503,4503,4501,4501,4499,4499,4492,4492,4491,4491,4489,4489,4486,4486,4483,4483,4481,4481,4480,4480,4478,4478,4477,4477,4475,4475,4472,4472,4471,4471,4470,4470,4468,4468,4465,4465,4463,4463,4461,4461,4457,4457,4456,4456,4451,4451,4450,4450,4449,4449,4448,4448,4447,4447,4446,4446,4444,4444,4442,4442,4441,4441,4434,4434,4433,4433,4428,4428,4427,4427,4426,4426,4425,4425,4424,4424,4422,4422,4419,4419,4418,4418,4415,4415,4413,4413,4410,4410,4409,4409,4408,4408,4407,4407,4405,4405,4404,4404,4402,4402,4398,4398,4397,4397,4394,4394,4393,4393,4391,4391,4389,4389,4386,4386,4384,4384,4380,4380,4379,4379,4377,4377,4374,4374,4373,4373,4371,4371,4369,4369,4368,4368,4365,4365,4364,4364,4363,4363,4362,4362,4360,4360,4358,4358,4355,4355,4352,4352,4351,4351,4350,4350,4349,4349,4348,4348,4346,4346,4345,4345,4342,4342,4341,4341,4340,4340,4339,4339,4337,4337,4336,4336,4334,4334,4332,4332,4330,4330,4329,4329,4328,4328,4327,4327,4323,4323,4322,4322,4321,4321,4320,4320,4318,4318,4317,4317,4316,4316,4315,4315,4313,4313,4311,4311,4308,4308,4306,4306,4304,4304,4303,4303,4302,4302,4300,4300,4299,4299,4296,4296,4295,4295,4293,4293,4292,4292,4291,4291,4288,4288,4287,4287,4285,4285,4283,4283,4282,4282,4281,4281,4280,4280,4279,4279,4278,4278,4277,4277,4276,4276,4274,4274,4273,4273,4272,4272,4271,4271,4266,4266,4263,4263,4262,4262,4261,4261,4259,4259,4258,4258,4255,4255,4253,4253,4252,4252,4251,4251,4243,4243,4240,4240,4238,4238,4236,4236,4234,4234,4233,4233,4231,4231,4230,4230,4229,4229,4228,4228,4227,4227,4226,4226,4225,4225,4224,4224,4223,4223,4222,4222,4221,4221,4220,4220,4219,4219,4218,4218,4216,4216,4214,4214,4212,4212,4211,4211,4210,4210,4208,4208,4207,4207,4206,4206,4205,4205,4204,4204,4201,4201,4200,4200,4197,4197,4196,4196,4194,4194,4192,4192,4191,4191,4190,4190,4189,4189,4186,4186,4185,4185,4184,4184,4183,4183,4182,4182,4181,4181,4180,4180,4178,4178,4174,4174,4173,4173,4171,4171,4170,4170,4168,4168,4166,4166,4162,4162,4161,4161,4158,4158,4157,4157,4154,4154,4153,4153,4152,4152,4150,4150,4149,4149,4148,4148,4147,4147,4146,4146,4145,4145,4144,4144,4143,4143,4141,4141,4140,4140,4139,4139,4137,4137,4136,4136,4135,4135,4132,4132,4131,4131,4130,4130,4129,4129,4126,4126,4125,4125,4124,4124,4123,4123,4121,4121,4120,4120,4119,4119,4118,4118,4117,4117,4116,4116,4113,4113,4112,4112,4111,4111,4110,4110,4107,4107,4106,4106,4103,4103,4102,4102,4101,4101,4100,4100,4096,4096,4095,4095,4094,4094,4091,4091,4090,4090,4089,4089,4086,4086,4084,4084,4082,4082,4081,4081,4078,4078,4076,4076,4075,4075,4073,4073,4072,4072,4071,4071,4070,4070,4069,4069,4068,4068,4067,4067,4066,4066,4062,4062,4061,4061,4060,4060,4059,4059,4055,4055,4052,4052,4051,4051,4048,4048,4047,4047,4046,4046,4045,4045,4043,4043,4042,4042,4039,4039,4038,4038,4037,4037,4036,4036,4035,4035,4032,4032,4031,4031,4030,4030,4029,4029,4026,4026,4025,4025,4023,4023,4022,4022,4020,4020,4018,4018,4016,4016,4015,4015,4014,4014,4013,4013,4012,4012,4011,4011,4010,4010,4008,4008,4007,4007,4006,4006,4005,4005,4003,4003,4002,4002,4001,4001,4000,4000,3998,3998,3992,3992,3991,3991,3990,3990,3988,3988,3987,3987,3983,3983,3982,3982,3981,3981,3979,3979,3978,3978,3976,3976,3975,3975,3973,3973,3970,3970,3968,3968,3966,3966,3965,3965,3962,3962,3961,3961,3960,3960,3959,3959,3958,3958,3956,3956,3955,3955,3953,3953,3952,3952,3950,3950,3949,3949,3948,3948,3947,3947,3946,3946,3945,3945,3944,3944,3941,3941,3940,3940,3939,3939,3938,3938,3937,3937,3935,3935,3934,3934,3933,3933,3932,3932,3931,3931,3929,3929,3926,3926,3924,3924,3921,3921,3919,3919,3918,3918,3917,3917,3916,3916,3915,3915,3913,3913,3912,3912,3909,3909,3907,3907,3906,3906,3905,3905,3904,3904,3902,3902,3899,3899,3898,3898,3897,3897,3896,3896,3895,3895,3894,3894,3893,3893,3891,3891,3889,3889,3888,3888,3884,3884,3883,3883,3882,3882,3879,3879,3878,3878,3876,3876,3873,3873,3872,3872,3870,3870,3869,3869,3868,3868,3867,3867,3866,3866,3865,3865,3864,3864,3862,3862,3861,3861,3859,3859,3858,3858,3857,3857,3856,3856,3855,3855,3853,3853,3852,3852,3850,3850,3849,3849,3845,3845,3844,3844,3843,3843,3842,3842,3841,3841,3840,3840,3839,3839,3837,3837,3836,3836,3834,3834,3832,3832,3831,3831,3830,3830,3827,3827,3826,3826,3824,3824,3823,3823,3822,3822,3820,3820,3819,3819,3818,3818,3815,3815,3814,3814,3813,3813,3810,3810,3809,3809,3808,3808,3807,3807,3805,3805,3802,3802,3801,3801,3797,3797,3795,3795,3794,3794,3793,3793,3792,3792,3791,3791,3788,3788,3787,3787,3786,3786,3785,3785,3780,3780,3779,3779,3778,3778,3777,3777,3776,3776,3775,3775,3773,3773,3771,3771,3770,3770,3769,3769,3768,3768,3767,3767,3766,3766,3765,3765,3764,3764,3763,3763,3762,3762,3761,3761,3760,3760,3758,3758,3757,3757,3756,3756,3754,3754,3753,3753,3752,3752,3750,3750,3749,3749,3748,3748,3747,3747,3746,3746,3745,3745,3743,3743,3742,3742,3741,3741,3740,3740,3738,3738,3734,3734,3733,3733,3732,3732,3731,3731,3729,3729,3727,3727,3724,3724,3722,3722,3721,3721,3720,3720,3719,3719,3718,3718,3717,3717,3716,3716,3715,3715,3714,3714,3713,3713,3712,3712,3711,3711,3710,3710,3709,3709,3705,3705,3703,3703,3701,3701,3700,3700,3698,3698,3697,3697,3696,3696,3695,3695,3694,3694,3693,3693,3692,3692,3691,3691,3690,3690,3689,3689,3686,3686,3685,3685,3684,3684,3683,3683,3682,3682,3680,3680,3679,3679,3678,3678,3677,3677,3675,3675,3674,3674,3673,3673,3671,3671,3668,3668,3667,3667,3666,3666,3665,3665,3663,3663,3662,3662,3661,3661,3660,3660,3658,3658,3657,3657,3656,3656,3655,3655,3654,3654,3653,3653,3652,3652,3651,3651,3650,3650,3649,3649,3647,3647,3646,3646,3645,3645,3644,3644,3642,3642,3641,3641,3640,3640,3639,3639,3638,3638,3637,3637,3636,3636,3635,3635,3634,3634,3633,3633,3628,3628,3627,3627,3626,3626,3624,3624,3623,3623,3621,3621,3620,3620,3619,3619,3617,3617,3615,3615,3613,3613,3612,3612,3611,3611,3610,3610,3608,3608,3606,3606,3605,3605,3604,3604,3603,3603,3602,3602,3601,3601,3600,3600,3599,3599,3598,3598,3597,3597,3596,3596,3595,3595,3594,3594,3593,3593,3591,3591,3589,3589,3587,3587,3584,3584,3583,3583,3582,3582,3581,3581,3580,3580,3579,3579,3578,3578,3576,3576,3575,3575,3573,3573,3571,3571,3569,3569,3567,3567,3566,3566,3565,3565,3563,3563,3562,3562,3559,3559,3557,3557,3555,3555,3554,3554,3553,3553,3551,3551,3550,3550,3548,3548,3545,3545,3544,3544,3543,3543,3541,3541,3540,3540,3539,3539,3538,3538,3537,3537,3536,3536,3534,3534,3529,3529,3527,3527,3526,3526,3524,3524,3522,3522,3521,3521,3520,3520,3519,3519,3518,3518,3517,3517,3515,3515,3514,3514,3513,3513,3512,3512,3511,3511,3510,3510,3509,3509,3508,3508,3506,3506,3504,3504,3503,3503,3502,3502,3501,3501,3500,3500,3498,3498,3496,3496,3495,3495,3494,3494,3493,3493,3492,3492,3490,3490,3489,3489,3488,3488,3486,3486,3483,3483,3482,3482,3481,3481,3480,3480,3479,3479,3478,3478,3477,3477,3475,3475,3474,3474,3473,3473,3472,3472,3470,3470,3469,3469,3468,3468,3467,3467,3466,3466,3465,3465,3464,3464,3462,3462,3461,3461,3460,3460,3459,3459,3457,3457,3456,3456,3455,3455,3454,3454,3451,3451,3450,3450,3448,3448,3447,3447,3446,3446,3445,3445,3444,3444,3443,3443,3442,3442,3440,3440,3439,3439,3438,3438,3437,3437,3435,3435,3434,3434,3432,3432,3431,3431,3430,3430,3428,3428,3427,3427,3426,3426,3425,3425,3424,3424,3423,3423,3422,3422,3421,3421,3420,3420,3419,3419,3418,3418,3416,3416,3415,3415,3414,3414,3412,3412,3409,3409,3408,3408,3407,3407,3406,3406,3403,3403,3402,3402,3401,3401,3400,3400,3398,3398,3397,3397,3396,3396,3395,3395,3394,3394,3393,3393,3391,3391,3390,3390,3389,3389,3387,3387,3385,3385,3384,3384,3383,3383,3381,3381,3380,3380,3379,3379,3378,3378,3377,3377,3376,3376,3375,3375,3374,3374,3373,3373,3371,3371,3370,3370,3368,3368,3367,3367,3366,3366,3363,3363,3362,3362,3361,3361,3358,3358,3357,3357,3356,3356,3355,3355,3354,3354,3353,3353,3352,3352,3351,3351,3349,3349,3348,3348,3347,3347,3346,3346,3345,3345,3344,3344,3343,3343,3342,3342,3341,3341,3340,3340,3339,3339,3338,3338,3335,3335,3334,3334,3333,3333,3331,3331,3330,3330,3328,3328,3327,3327,3326,3326,3325,3325,3324,3324,3323,3323,3322,3322,3321,3321,3320,3320,3319,3319,3318,3318,3317,3317,3315,3315,3314,3314,3313,3313,3312,3312,3310,3310,3309,3309,3308,3308,3307,3307,3306,3306,3305,3305,3304,3304,3302,3302,3301,3301,3300,3300,3299,3299,3298,3298,3296,3296,3293,3293,3291,3291,3290,3290,3289,3289,3286,3286,3285,3285,3284,3284,3283,3283,3281,3281,3280,3280,3279,3279,3278,3278,3277,3277,3276,3276,3275,3275,3274,3274,3272,3272,3271,3271,3270,3270,3269,3269,3268,3268,3267,3267,3266,3266,3265,3265,3264,3264,3263,3263,3262,3262,3261,3261,3260,3260,3259,3259,3258,3258,3257,3257,3256,3256,3255,3255,3254,3254,3253,3253,3252,3252,3250,3250,3249,3249,3248,3248,3247,3247,3246,3246,3244,3244,3243,3243,3242,3242,3241,3241,3239,3239,3238,3238,3236,3236,3235,3235,3234,3234,3233,3233,3232,3232,3231,3231,3230,3230,3229,3229,3228,3228,3227,3227,3226,3226,3224,3224,3223,3223,3221,3221,3219,3219,3218,3218,3217,3217,3216,3216,3215,3215,3213,3213,3212,3212,3211,3211,3210,3210,3209,3209,3208,3208,3207,3207,3206,3206,3204,3204,3202,3202,3201,3201,3200,3200,3199,3199,3196,3196,3195,3195,3194,3194,3193,3193,3191,3191,3189,3189,3188,3188,3187,3187,3186,3186,3184,3184,3183,3183,3182,3182,3181,3181,3180,3180,3178,3178,3177,3177,3175,3175,3174,3174,3173,3173,3171,3171,3168,3168,3167,3167,3166,3166,3165,3165,3163,3163,3162,3162,3161,3161,3160,3160,3159,3159,3158,3158,3157,3157,3156,3156,3154,3154,3153,3153,3152,3152,3151,3151,3150,3150,3149,3149,3147,3147,3144,3144,3143,3143,3142,3142,3141,3141,3140,3140,3139,3139,3137,3137,3136,3136,3135,3135,3134,3134,3133,3133,3132,3132,3131,3131,3130,3130,3129,3129,3127,3127,3126,3126,3125,3125,3124,3124,3123,3123,3122,3122,3121,3121,3118,3118,3117,3117,3116,3116,3115,3115,3114,3114,3113,3113,3112,3112,3111,3111,3110,3110,3109,3109,3108,3108,3107,3107,3104,3104,3103,3103,3102,3102,3101,3101,3100,3100,3099,3099,3097,3097,3096,3096,3095,3095,3094,3094,3093,3093,3092,3092,3091,3091,3088,3088,3086,3086,3085,3085,3083,3083,3082,3082,3081,3081,3080,3080,3079,3079,3076,3076,3075,3075,3074,3074,3073,3073,3072,3072,3071,3071,3070,3070,3069,3069,3068,3068,3067,3067,3066,3066,3065,3065,3064,3064,3063,3063,3062,3062,3060,3060,3059,3059,3056,3056,3055,3055,3054,3054,3053,3053,3052,3052,3051,3051,3050,3050,3049,3049,3047,3047,3046,3046,3045,3045,3044,3044,3043,3043,3040,3040,3039,3039,3037,3037,3035,3035,3034,3034,3033,3033,3032,3032,3031,3031,3028,3028,3027,3027,3026,3026,3025,3025,3024,3024,3023,3023,3022,3022,3020,3020,3017,3017,3016,3016,3015,3015,3014,3014,3013,3013,3012,3012,3011,3011,3009,3009,3008,3008,3007,3007,3006,3006,3005,3005,3003,3003,3002,3002,3001,3001,3000,3000,2999,2999,2998,2998,2997,2997,2996,2996,2995,2995,2994,2994,2993,2993,2992,2992,2991,2991,2990,2990,2989,2989,2988,2988,2985,2985,2984,2984,2983,2983,2982,2982,2981,2981,2980,2980,2979,2979,2978,2978,2977,2977,2975,2975,2974,2974,2973,2973,2972,2972,2971,2971,2970,2970,2968,2968,2967,2967,2966,2966,2965,2965,2964,2964,2963,2963,2961,2961,2960,2960,2959,2959,2958,2958,2957,2957,2956,2956,2954,2954,2953,2953,2952,2952,2951,2951,2950,2950,2949,2949,2948,2948,2947,2947,2946,2946,2945,2945,2944,2944,2943,2943,2940,2940,2939,2939,2938,2938,2937,2937,2936,2936,2935,2935,2934,2934,2933,2933,2932,2932,2931,2931,2930,2930,2929,2929,2928,2928,2927,2927,2926,2926,2925,2925,2924,2924,2923,2923,2922,2922,2921,2921,2919,2919,2918,2918,2917,2917,2916,2916,2915,2915,2914,2914,2913,2913,2911,2911,2910,2910,2909,2909,2907,2907,2906,2906,2905,2905,2904,2904,2901,2901,2900,2900,2899,2899,2898,2898,2897,2897,2896,2896,2895,2895,2893,2893,2891,2891,2890,2890,2888,2888,2887,2887,2886,2886,2885,2885,2884,2884,2883,2883,2882,2882,2881,2881,2880,2880,2879,2879,2878,2878,2877,2877,2876,2876,2875,2875,2874,2874,2873,2873,2872,2872,2871,2871,2870,2870,2869,2869,2868,2868,2867,2867,2865,2865,2864,2864,2863,2863,2862,2862,2860,2860,2859,2859,2858,2858,2857,2857,2856,2856,2854,2854,2853,2853,2852,2852,2851,2851,2850,2850,2847,2847,2846,2846,2845,2845,2844,2844,2843,2843,2841,2841,2840,2840,2838,2838,2837,2837,2836,2836,2835,2835,2834,2834,2833,2833,2832,2832,2831,2831,2830,2830,2829,2829,2828,2828,2826,2826,2825,2825,2823,2823,2822,2822,2821,2821,2820,2820,2819,2819,2817,2817,2816,2816,2815,2815,2814,2814,2813,2813,2812,2812,2811,2811,2809,2809,2808,2808,2807,2807,2805,2805,2804,2804,2803,2803,2802,2802,2801,2801,2800,2800,2799,2799,2798,2798,2796,2796,2795,2795,2794,2794,2793,2793,2792,2792,2791,2791,2789,2789,2788,2788,2787,2787,2786,2786,2785,2785,2784,2784,2783,2783,2782,2782,2781,2781,2779,2779,2777,2777,2775,2775,2773,2773,2772,2772,2771,2771,2768,2768,2767,2767,2766,2766,2765,2765,2764,2764,2763,2763,2762,2762,2761,2761,2758,2758,2757,2757,2756,2756,2755,2755,2754,2754,2753,2753,2752,2752,2751,2751,2750,2750,2749,2749,2748,2748,2747,2747,2746,2746,2745,2745,2743,2743,2742,2742,2741,2741,2740,2740,2738,2738,2737,2737,2735,2735,2734,2734,2733,2733,2732,2732,2730,2730,2729,2729,2728,2728,2727,2727,2726,2726,2725,2725,2724,2724,2723,2723,2721,2721,2720,2720,2719,2719,2717,2717,2715,2715,2714,2714,2712,2712,2711,2711,2710,2710,2707,2707,2705,2705,2704,2704,2703,2703,2700,2700,2698,2698,2697,2697,2696,2696,2695,2695,2692,2692,2691,2691,2689,2689,2688,2688,2687,2687,2685,2685,2684,2684,2683,2683,2682,2682,2681,2681,2680,2680,2679,2679,2677,2677,2676,2676,2672,2672,2671,2671,2670,2670,2669,2669,2668,2668,2666,2666,2665,2665,2664,2664,2663,2663,2662,2662,2661,2661,2659,2659,2658,2658,2657,2657,2656,2656,2655,2655,2654,2654,2653,2653,2652,2652,2651,2651,2650,2650,2649,2649,2647,2647,2646,2646,2645,2645,2644,2644,2643,2643,2642,2642,2641,2641,2640,2640,2639,2639,2638,2638,2637,2637,2636,2636,2635,2635,2634,2634,2633,2633,2632,2632,2631,2631,2630,2630,2628,2628,2627,2627,2626,2626,2625,2625,2624,2624,2623,2623,2622,2622,2621,2621,2619,2619,2618,2618,2617,2617,2616,2616,2615,2615,2614,2614,2613,2613,2612,2612,2611,2611,2610,2610,2609,2609,2607,2607,2606,2606,2605,2605,2604,2604,2603,2603,2601,2601,2600,2600,2599,2599,2598,2598,2597,2597,2596,2596,2595,2595,2594,2594,2591,2591,2590,2590,2589,2589,2588,2588,2587,2587,2586,2586,2584,2584,2583,2583,2582,2582,2581,2581,2580,2580,2579,2579,2578,2578,2577,2577,2576,2576,2575,2575,2574,2574,2573,2573,2572,2572,2571,2571,2570,2570,2569,2569,2568,2568,2567,2567,2566,2566,2565,2565,2564,2564,2563,2563,2562,2562,2561,2561,2560,2560,2559,2559,2558,2558,2557,2557,2556,2556,2555,2555,2554,2554,2553,2553,2552,2552,2551,2551,2550,2550,2549,2549,2548,2548,2547,2547,2546,2546,2545,2545,2544,2544,2543,2543,2541,2541,2540,2540,2539,2539,2538,2538,2537,2537,2536,2536,2535,2535,2534,2534,2533,2533,2532,2532,2530,2530,2529,2529,2528,2528,2527,2527,2526,2526,2525,2525,2524,2524,2522,2522,2520,2520,2519,2519,2518,2518,2516,2516,2515,2515,2514,2514,2513,2513,2512,2512,2511,2511,2510,2510,2509,2509,2508,2508,2507,2507,2506,2506,2505,2505,2504,2504,2503,2503,2502,2502,2501,2501,2500,2500,2499,2499,2498,2498,2497,2497,2496,2496,2495,2495,2494,2494,2493,2493,2492,2492,2491,2491,2490,2490,2489,2489,2488,2488,2487,2487,2486,2486,2485,2485,2484,2484,2483,2483,2482,2482,2481,2481,2480,2480,2478,2478,2477,2477,2476,2476,2475,2475,2474,2474,2473,2473,2472,2472,2471,2471,2470,2470,2469,2469,2468,2468,2467,2467,2466,2466,2464,2464,2463,2463,2462,2462,2461,2461,2459,2459,2458,2458,2457,2457,2456,2456,2455,2455,2454,2454,2453,2453,2452,2452,2451,2451,2449,2449,2448,2448,2447,2447,2446,2446,2445,2445,2444,2444,2443,2443,2442,2442,2441,2441,2440,2440,2439,2439,2438,2438,2437,2437,2435,2435,2434,2434,2433,2433,2432,2432,2431,2431,2430,2430,2429,2429,2428,2428,2427,2427,2426,2426,2425,2425,2424,2424,2423,2423,2422,2422,2421,2421,2420,2420,2419,2419,2418,2418,2416,2416,2415,2415,2414,2414,2412,2412,2411,2411,2410,2410,2409,2409,2408,2408,2407,2407,2406,2406,2405,2405,2404,2404,2403,2403,2402,2402,2401,2401,2400,2400,2399,2399,2398,2398,2396,2396,2394,2394,2393,2393,2392,2392,2391,2391,2390,2390,2389,2389,2388,2388,2386,2386,2385,2385,2384,2384,2383,2383,2382,2382,2381,2381,2380,2380,2379,2379,2378,2378,2377,2377,2376,2376,2375,2375,2374,2374,2373,2373,2372,2372,2371,2371,2370,2370,2369,2369,2368,2368,2367,2367,2366,2366,2365,2365,2364,2364,2362,2362,2361,2361,2360,2360,2359,2359,2357,2357,2356,2356,2355,2355,2354,2354,2353,2353,2352,2352,2351,2351,2350,2350,2349,2349,2348,2348,2347,2347,2346,2346,2345,2345,2344,2344,2343,2343,2342,2342,2341,2341,2340,2340,2339,2339,2337,2337,2336,2336,2335,2335,2334,2334,2332,2332,2331,2331,2330,2330,2329,2329,2328,2328,2327,2327,2326,2326,2325,2325,2324,2324,2323,2323,2322,2322,2321,2321,2320,2320,2319,2319,2317,2317,2316,2316,2315,2315,2314,2314,2313,2313,2312,2312,2311,2311,2310,2310,2309,2309,2307,2307,2306,2306,2305,2305,2304,2304,2303,2303,2302,2302,2301,2301,2300,2300,2299,2299,2298,2298,2296,2296,2295,2295,2294,2294,2293,2293,2292,2292,2291,2291,2290,2290,2289,2289,2288,2288,2287,2287,2286,2286,2285,2285,2284,2284,2283,2283,2282,2282,2281,2281,2280,2280,2279,2279,2278,2278,2277,2277,2276,2276,2275,2275,2274,2274,2273,2273,2272,2272,2271,2271,2270,2270,2269,2269,2268,2268,2267,2267,2265,2265,2264,2264,2263,2263,2262,2262,2261,2261,2260,2260,2259,2259,2258,2258,2257,2257,2256,2256,2254,2254,2253,2253,2252,2252,2251,2251,2250,2250,2249,2249,2248,2248,2247,2247,2246,2246,2245,2245,2244,2244,2243,2243,2242,2242,2241,2241,2240,2240,2239,2239,2238,2238,2237,2237,2236,2236,2235,2235,2234,2234,2233,2233,2232,2232,2231,2231,2229,2229,2228,2228,2227,2227,2226,2226,2225,2225,2224,2224,2223,2223,2222,2222,2221,2221,2220,2220,2219,2219,2218,2218,2217,2217,2214,2214,2213,2213,2212,2212,2211,2211,2210,2210,2209,2209,2208,2208,2207,2207,2206,2206,2204,2204,2203,2203,2202,2202,2201,2201,2200,2200,2199,2199,2198,2198,2197,2197,2196,2196,2195,2195,2194,2194,2193,2193,2192,2192,2190,2190,2189,2189,2188,2188,2187,2187,2186,2186,2185,2185,2184,2184,2183,2183,2182,2182,2181,2181,2180,2180,2179,2179,2178,2178,2177,2177,2176,2176,2175,2175,2174,2174,2173,2173,2172,2172,2171,2171,2170,2170,2169,2169,2168,2168,2167,2167,2165,2165,2164,2164,2163,2163,2162,2162,2161,2161,2160,2160,2159,2159,2158,2158,2157,2157,2156,2156,2155,2155,2154,2154,2153,2153,2152,2152,2151,2151,2149,2149,2148,2148,2147,2147,2145,2145,2143,2143,2142,2142,2141,2141,2140,2140,2139,2139,2138,2138,2137,2137,2136,2136,2135,2135,2134,2134,2133,2133,2132,2132,2130,2130,2129,2129,2128,2128,2127,2127,2126,2126,2125,2125,2124,2124,2123,2123,2122,2122,2121,2121,2120,2120,2119,2119,2118,2118,2117,2117,2116,2116,2115,2115,2114,2114,2113,2113,2112,2112,2110,2110,2109,2109,2108,2108,2107,2107,2106,2106,2105,2105,2104,2104,2103,2103,2102,2102,2101,2101,2100,2100,2099,2099,2098,2098,2097,2097,2096,2096,2095,2095,2094,2094,2093,2093,2092,2092,2091,2091,2090,2090,2089,2089,2088,2088,2087,2087,2086,2086,2085,2085,2084,2084,2083,2083,2082,2082,2081,2081,2079,2079,2078,2078,2077,2077,2076,2076,2075,2075,2074,2074,2073,2073,2072,2072,2071,2071,2070,2070,2069,2069,2068,2068,2067,2067,2066,2066,2065,2065,2064,2064,2063,2063,2062,2062,2061,2061,2060,2060,2059,2059,2058,2058,2056,2056,2055,2055,2054,2054,2053,2053,2052,2052,2051,2051,2050,2050,2049,2049,2048,2048,2047,2047,2046,2046,2045,2045,2044,2044,2043,2043,2042,2042,2041,2041,2040,2040,2039,2039,2038,2038,2037,2037,2036,2036,2034,2034,2033,2033,2032,2032,2031,2031,2030,2030,2029,2029,2028,2028,2027,2027,2026,2026,2024,2024,2023,2023,2022,2022,2021,2021,2020,2020,2019,2019,2018,2018,2017,2017,2016,2016,2015,2015,2014,2014,2013,2013,2012,2012,2011,2011,2010,2010,2009,2009,2008,2008,2007,2007,2006,2006,2005,2005,2004,2004,2003,2003,2002,2002,2001,2001,2000,2000,1999,1999,1997,1997,1996,1996,1995,1995,1994,1994,1993,1993,1992,1992,1991,1991,1990,1990,1989,1989,1988,1988,1987,1987,1986,1986,1985,1985,1984,1984,1983,1983,1982,1982,1981,1981,1980,1980,1979,1979,1978,1978,1977,1977,1976,1976,1975,1975,1974,1974,1973,1973,1972,1972,1971,1971,1970,1970,1969,1969,1968,1968,1967,1967,1966,1966,1965,1965,1964,1964,1963,1963,1962,1962,1961,1961,1960,1960,1959,1959,1958,1958,1957,1957,1956,1956,1955,1955,1954,1954,1953,1953,1952,1952,1951,1951,1950,1950,1949,1949,1948,1948,1947,1947,1946,1946,1945,1945,1944,1944,1943,1943,1942,1942,1941,1941,1939,1939,1938,1938,1937,1937,1936,1936,1935,1935,1934,1934,1933,1933,1932,1932,1931,1931,1930,1930,1929,1929,1928,1928,1927,1927,1926,1926,1925,1925,1924,1924,1923,1923,1922,1922,1921,1921,1920,1920,1919,1919,1918,1918,1917,1917,1916,1916,1915,1915,1914,1914,1913,1913,1912,1912,1911,1911,1909,1909,1908,1908,1907,1907,1906,1906,1904,1904,1903,1903,1902,1902,1901,1901,1900,1900,1899,1899,1898,1898,1897,1897,1896,1896,1895,1895,1894,1894,1892,1892,1891,1891,1890,1890,1889,1889,1888,1888,1887,1887,1886,1886,1885,1885,1884,1884,1883,1883,1882,1882,1881,1881,1880,1880,1879,1879,1878,1878,1877,1877,1876,1876,1875,1875,1874,1874,1873,1873,1872,1872,1871,1871,1870,1870,1869,1869,1868,1868,1867,1867,1866,1866,1865,1865,1864,1864,1863,1863,1862,1862,1861,1861,1860,1860,1859,1859,1858,1858,1857,1857,1856,1856,1855,1855,1854,1854,1853,1853,1852,1852,1851,1851,1849,1849,1848,1848,1847,1847,1846,1846,1845,1845,1844,1844,1843,1843,1842,1842,1841,1841,1840,1840,1839,1839,1838,1838,1837,1837,1836,1836,1835,1835,1834,1834,1833,1833,1832,1832,1831,1831,1830,1830,1829,1829,1828,1828,1827,1827,1826,1826,1825,1825,1824,1824,1823,1823,1822,1822,1821,1821,1820,1820,1819,1819,1818,1818,1817,1817,1816,1816,1815,1815,1814,1814,1813,1813,1812,1812,1811,1811,1810,1810,1809,1809,1808,1808,1807,1807,1806,1806,1805,1805,1804,1804,1803,1803,1802,1802,1801,1801,1800,1800,1799,1799,1798,1798,1797,1797,1796,1796,1795,1795,1794,1794,1793,1793,1792,1792,1791,1791,1790,1790,1789,1789,1788,1788,1787,1787,1786,1786,1785,1785,1784,1784,1783,1783,1782,1782,1781,1781,1780,1780,1778,1778,1777,1777,1776,1776,1774,1774,1773,1773,1772,1772,1771,1771,1770,1770,1769,1769,1768,1768,1767,1767,1766,1766,1765,1765,1764,1764,1763,1763,1762,1762,1761,1761,1760,1760,1759,1759,1758,1758,1757,1757,1756,1756,1755,1755,1754,1754,1753,1753,1752,1752,1751,1751,1750,1750,1749,1749,1748,1748,1747,1747,1746,1746,1745,1745,1744,1744,1743,1743,1742,1742,1741,1741,1740,1740,1739,1739,1738,1738,1737,1737,1736,1736,1735,1735,1734,1734,1733,1733,1732,1732,1731,1731,1730,1730,1729,1729,1728,1728,1727,1727,1726,1726,1725,1725,1724,1724,1723,1723,1722,1722,1720,1720,1719,1719,1718,1718,1717,1717,1716,1716,1715,1715,1714,1714,1713,1713,1712,1712,1711,1711,1710,1710,1709,1709,1708,1708,1707,1707,1706,1706,1705,1705,1704,1704,1703,1703,1701,1701,1700,1700,1699,1699,1698,1698,1697,1697,1696,1696,1695,1695,1694,1694,1693,1693,1692,1692,1691,1691,1690,1690,1689,1689,1688,1688,1687,1687,1686,1686,1685,1685,1684,1684,1683,1683,1682,1682,1681,1681,1680,1680,1679,1679,1678,1678,1677,1677,1676,1676,1675,1675,1674,1674,1673,1673,1672,1672,1671,1671,1670,1670,1669,1669,1668,1668,1666,1666,1665,1665,1664,1664,1663,1663,1662,1662,1661,1661,1660,1660,1659,1659,1658,1658,1657,1657,1656,1656,1655,1655,1654,1654,1653,1653,1652,1652,1651,1651,1650,1650,1649,1649,1647,1647,1646,1646,1645,1645,1644,1644,1643,1643,1642,1642,1641,1641,1640,1640,1639,1639,1638,1638,1636,1636,1635,1635,1634,1634,1633,1633,1631,1631,1630,1630,1629,1629,1628,1628,1627,1627,1626,1626,1625,1625,1624,1624,1623,1623,1622,1622,1621,1621,1620,1620,1619,1619,1618,1618,1617,1617,1616,1616,1615,1615,1614,1614,1613,1613,1612,1612,1611,1611,1610,1610,1609,1609,1608,1608,1607,1607,1606,1606,1605,1605,1604,1604,1603,1603,1602,1602,1601,1601,1600,1600,1599,1599,1598,1598,1597,1597,1596,1596,1595,1595,1594,1594,1593,1593,1592,1592,1591,1591,1590,1590,1589,1589,1588,1588,1587,1587,1586,1586,1585,1585,1584,1584,1583,1583,1582,1582,1581,1581,1580,1580,1579,1579,1578,1578,1577,1577,1576,1576,1575,1575,1574,1574,1573,1573,1572,1572,1571,1571,1570,1570,1569,1569,1568,1568,1567,1567,1566,1566,1565,1565,1564,1564,1563,1563,1562,1562,1561,1561,1560,1560,1559,1559,1558,1558,1557,1557,1556,1556,1555,1555,1554,1554,1553,1553,1552,1552,1551,1551,1550,1550,1549,1549,1548,1548,1547,1547,1546,1546,1545,1545,1544,1544,1543,1543,1542,1542,1541,1541,1539,1539,1538,1538,1537,1537,1536,1536,1535,1535,1534,1534,1532,1532,1530,1530,1529,1529,1528,1528,1527,1527,1526,1526,1525,1525,1524,1524,1523,1523,1522,1522,1521,1521,1520,1520,1519,1519,1518,1518,1517,1517,1516,1516,1515,1515,1514,1514,1513,1513,1512,1512,1511,1511,1510,1510,1509,1509,1508,1508,1507,1507,1506,1506,1505,1505,1504,1504,1503,1503,1502,1502,1501,1501,1500,1500,1499,1499,1498,1498,1497,1497,1496,1496,1495,1495,1494,1494,1493,1493,1492,1492,1491,1491,1490,1490,1489,1489,1488,1488,1487,1487,1486,1486,1485,1485,1484,1484,1483,1483,1482,1482,1481,1481,1480,1480,1479,1479,1478,1478,1477,1477,1476,1476,1475,1475,1474,1474,1472,1472,1471,1471,1470,1470,1469,1469,1468,1468,1467,1467,1466,1466,1465,1465,1464,1464,1463,1463,1462,1462,1461,1461,1460,1460,1459,1459,1458,1458,1457,1457,1456,1456,1455,1455,1454,1454,1453,1453,1452,1452,1451,1451,1450,1450,1449,1449,1448,1448,1447,1447,1446,1446,1445,1445,1444,1444,1443,1443,1442,1442,1441,1441,1439,1439,1438,1438,1437,1437,1436,1436,1435,1435,1434,1434,1433,1433,1432,1432,1431,1431,1430,1430,1429,1429,1428,1428,1427,1427,1426,1426,1425,1425,1424,1424,1423,1423,1422,1422,1421,1421,1420,1420,1419,1419,1418,1418,1417,1417,1416,1416,1415,1415,1414,1414,1413,1413,1410,1410,1409,1409,1408,1408,1407,1407,1406,1406,1405,1405,1404,1404,1402,1402,1401,1401,1400,1400,1399,1399,1398,1398,1397,1397,1396,1396,1395,1395,1394,1394,1393,1393,1392,1392,1391,1391,1390,1390,1389,1389,1388,1388,1387,1387,1386,1386,1385,1385,1384,1384,1383,1383,1382,1382,1381,1381,1380,1380,1379,1379,1378,1378,1377,1377,1376,1376,1375,1375,1374,1374,1373,1373,1372,1372,1371,1371,1370,1370,1369,1369,1368,1368,1366,1366,1365,1365,1364,1364,1363,1363,1362,1362,1361,1361,1360,1360,1359,1359,1358,1358,1357,1357,1356,1356,1355,1355,1354,1354,1353,1353,1352,1352,1350,1350,1349,1349,1348,1348,1347,1347,1346,1346,1345,1345,1344,1344,1343,1343,1342,1342,1341,1341,1340,1340,1339,1339,1338,1338,1337,1337,1336,1336,1335,1335,1334,1334,1333,1333,1332,1332,1331,1331,1330,1330,1329,1329,1328,1328,1327,1327,1326,1326,1325,1325,1324,1324,1323,1323,1322,1322,1321,1321,1320,1320,1319,1319,1318,1318,1317,1317,1316,1316,1315,1315,1314,1314,1313,1313,1312,1312,1311,1311,1310,1310,1309,1309,1308,1308,1307,1307,1306,1306,1305,1305,1304,1304,1303,1303,1302,1302,1301,1301,1300,1300,1299,1299,1298,1298,1297,1297,1296,1296,1295,1295,1294,1294,1293,1293,1292,1292,1291,1291,1290,1290,1289,1289,1288,1288,1287,1287,1286,1286,1285,1285,1284,1284,1283,1283,1282,1282,1281,1281,1280,1280,1279,1279,1278,1278,1277,1277,1276,1276,1275,1275,1274,1274,1273,1273,1272,1272,1271,1271,1270,1270,1269,1269,1268,1268,1267,1267,1266,1266,1265,1265,1264,1264,1263,1263,1262,1262,1261,1261,1260,1260,1259,1259,1258,1258,1257,1257,1256,1256,1255,1255,1254,1254,1253,1253,1252,1252,1251,1251,1250,1250,1249,1249,1248,1248,1247,1247,1246,1246,1245,1245,1244,1244,1243,1243,1242,1242,1241,1241,1240,1240,1239,1239,1238,1238,1237,1237,1236,1236,1235,1235,1234,1234,1233,1233,1232,1232,1231,1231,1230,1230,1229,1229,1228,1228,1227,1227,1226,1226,1225,1225,1223,1223,1222,1222,1221,1221,1220,1220,1219,1219,1218,1218,1217,1217,1216,1216,1215,1215,1214,1214,1213,1213,1212,1212,1211,1211,1210,1210,1209,1209,1208,1208,1207,1207,1206,1206,1205,1205,1204,1204,1203,1203,1202,1202,1201,1201,1200,1200,1199,1199,1198,1198,1197,1197,1196,1196,1195,1195,1194,1194,1193,1193,1192,1192,1191,1191,1190,1190,1189,1189,1188,1188,1187,1187,1186,1186,1185,1185,1184,1184,1183,1183,1182,1182,1181,1181,1180,1180,1179,1179,1178,1178,1177,1177,1176,1176,1175,1175,1174,1174,1173,1173,1172,1172,1171,1171,1170,1170,1169,1169,1167,1167,1166,1166,1165,1165,1164,1164,1163,1163,1162,1162,1161,1161,1160,1160,1159,1159,1158,1158,1156,1156,1155,1155,1154,1154,1153,1153,1152,1152,1151,1151,1150,1150,1149,1149,1148,1148,1147,1147,1146,1146,1145,1145,1144,1144,1143,1143,1142,1142,1140,1140,1139,1139,1137,1137,1136,1136,1135,1135,1134,1134,1133,1133,1132,1132,1131,1131,1130,1130,1129,1129,1128,1128,1127,1127,1126,1126,1125,1125,1124,1124,1123,1123,1122,1122,1121,1121,1120,1120,1119,1119,1118,1118,1117,1117,1116,1116,1115,1115,1114,1114,1113,1113,1112,1112,1111,1111,1110,1110,1109,1109,1108,1108,1107,1107,1106,1106,1105,1105,1104,1104,1103,1103,1102,1102,1101,1101,1100,1100,1099,1099,1098,1098,1097,1097,1096,1096,1095,1095,1094,1094,1093,1093,1092,1092,1091,1091,1090,1090,1089,1089,1088,1088,1087,1087,1086,1086,1085,1085,1084,1084,1083,1083,1082,1082,1081,1081,1080,1080,1079,1079,1078,1078,1077,1077,1076,1076,1075,1075,1074,1074,1073,1073,1072,1072,1071,1071,1070,1070,1069,1069,1068,1068,1067,1067,1066,1066,1065,1065,1064,1064,1063,1063,1062,1062,1061,1061,1060,1060,1059,1059,1058,1058,1057,1057,1056,1056,1055,1055,1054,1054,1053,1053,1052,1052,1051,1051,1050,1050,1049,1049,1048,1048,1047,1047,1046,1046,1045,1045,1044,1044,1043,1043,1042,1042,1041,1041,1040,1040,1039,1039,1038,1038,1037,1037,1036,1036,1035,1035,1034,1034,1033,1033,1032,1032,1030,1030,1029,1029,1028,1028,1027,1027,1026,1026,1025,1025,1024,1024,1023,1023,1022,1022,1021,1021,1020,1020,1019,1019,1018,1018,1017,1017,1016,1016,1015,1015,1014,1014,1013,1013,1012,1012,1011,1011,1010,1010,1009,1009,1008,1008,1007,1007,1006,1006,1005,1005,1004,1004,1003,1003,1002,1002,1001,1001,1000,1000,999,999,998,998,997,997,996,996,995,995,994,994,993,993,992,992,991,991,990,990,989,989,988,988,987,987,986,986,985,985,984,984,983,983,982,982,981,981,980,980,979,979,978,978,977,977,976,976,975,975,974,974,973,973,972,972,971,971,970,970,969,969,968,968,967,967,966,966,965,965,964,964,963,963,962,962,961,961,960,960,959,959,958,958,957,957,956,956,955,955,954,954,953,953,952,952,951,951,950,950,949,949,948,948,947,947,946,946,945,945,944,944,943,943,942,942,941,941,940,940,939,939,938,938,937,937,936,936,935,935,934,934,933,933,932,932,931,931,930,930,929,929,928,928,927,927,926,926,925,925,924,924,923,923,922,922,921,921,920,920,919,919,918,918,917,917,916,916,915,915,914,914,913,913,912,912,911,911,910,910,909,909,908,908,907,907,906,906,905,905,904,904,903,903,902,902,901,901,900,900,899,899,898,898,897,897,896,896,895,895,894,894,893,893,892,892,891,891,890,890,889,889,888,888,887,887,886,886,885,885,884,884,883,883,882,882,881,881,880,880,879,879,878,878,877,877,876,876,875,875,874,874,873,873,872,872,871,871,870,870,869,869,868,868,867,867,866,866,865,865,864,864,863,863,862,862,861,861,860,860,859,859,858,858,857,857,856,856,855,855,854,854,853,853,852,852,851,851,850,850,849,849,848,848,847,847,846,846,845,845,844,844,843,843,842,842,841,841,840,840,839,839,838,838,837,837,836,836,835,835,834,834,833,833,832,832,831,831,830,830,829,829,828,828,827,827,826,826,825,825,824,824,823,823,822,822,821,821,820,820,819,819,818,818,817,817,816,816,815,815,814,814,813,813,812,812,811,811,810,810,809,809,808,808,807,807,806,806,805,805,804,804,803,803,802,802,801,801,800,800,799,799,798,798,797,797,796,796,795,795,794,794,793,793,792,792,791,791,790,790,789,789,788,788,787,787,786,786,785,785,784,784,783,783,782,782,780,780,779,779,778,778,777,777,776,776,775,775,774,774,773,773,772,772,771,771,770,770,768,768,767,767,766,766,765,765,764,764,763,763,762,762,761,761,760,760,759,759,758,758,757,757,756,756,755,755,753,753,752,752,750,750,749,749,748,748,747,747,746,746,745,745,744,744,743,743,741,741,740,740,739,739,738,738,737,737,736,736,735,735,734,734,733,733,732,732,731,731,730,730,729,729,728,728,727,727,726,726,725,725,724,724,723,723,722,722,721,721,720,720,719,719,718,718,717,717,716,716,715,715,713,713,712,712,711,711,710,710,709,709,708,708,707,707,706,706,705,705,704,704,703,703,702,702,701,701,700,700,699,699,698,698,697,697,696,696,695,695,694,694,693,693,692,692,691,691,690,690,689,689,688,688,687,687,686,686,685,685,684,684,682,682,681,681,680,680,679,679,678,678,677,677,676,676,675,675,674,674,673,673,672,672,671,671,670,670,669,669,668,668,667,667,666,666,665,665,664,664,663,663,662,662,661,661,660,660,659,659,658,658,657,657,656,656,655,655,654,654,653,653,652,652,651,651,650,650,649,649,648,648,647,647,646,646,645,645,644,644,643,643,642,642,641,641,640,640,639,639,638,638,637,637,636,636,635,635,634,634,633,633,632,632,631,631,630,630,629,629,628,628,627,627,626,626,625,625,624,624,623,623,622,622,621,621,620,620,619,619,618,618,617,617,616,616,615,615,614,614,613,613,612,612,611,611,610,610,609,609,608,608,607,607,606,606,605,605,604,604,603,603,602,602,601,601,600,600,599,599,598,598,597,597,596,596,595,595,594,594,593,593,592,592,591,591,590,590,589,589,588,588,587,587,586,586,585,585,584,584,583,583,582,582,581,581,580,580,579,579,578,578,577,577,576,576,575,575,574,574,573,573,572,572,571,571,570,570,569,569,568,568,567,567,566,566,565,565,564,564,563,563,562,562,561,561,560,560,559,559,558,558,557,557,556,556,555,555,554,554,553,553,552,552,551,551,550,550,549,549,548,548,547,547,546,546,545,545,544,544,543,543,542,542,541,541,540,540,539,539,538,538,537,537,536,536,535,535,534,534,533,533,532,532,531,531,530,530,529,529,528,528,527,527,526,526,525,525,524,524,523,523,522,522,521,521,520,520,519,519,518,518,517,517,516,516,515,515,514,514,513,513,512,512,511,511,510,510,509,509,508,508,506,506,505,505,504,504,503,503,502,502,501,501,499,499,498,498,497,497,496,496,495,495,494,494,493,493,492,492,491,491,490,490,489,489,488,488,487,487,486,486,485,485,484,484,483,483,482,482,481,481,480,480,479,479,478,478,477,477,476,476,475,475,474,474,473,473,472,472,470,470,469,469,468,468,467,467,466,466,465,465,463,463,462,462,461,461,460,460,459,459,458,458,457,457,456,456,455,455,453,453,452,452,451,451,449,449,448,448,445,445,444,444,443,443,442,442,441,441,440,440,437,437,435,435,433,433,432,432,430,430,429,429,428,428,427,427,426,426,425,425,424,424,422,422,421,421,420,420,419,419,417,417,414,414,412,412,411,411,409,409,407,407,406,406,405,405,403,403,400,400,0.0],[20600,20600,20600,16685,16685,15183,15183,14265,14265,13981,13981,13628,13628,13415,13415,13076,13076,12895,12895,12760,12760,12680,12680,12227,12227,12200,12200,12109,12109,12079,12079,11815,11815,11802,11802,11570,11570,11552,11552,11513,11513,11284,11284,11176,11176,11166,11166,11156,11156,11098,11098,11059,11059,10940,10940,10830,10830,10796,10796,10667,10667,10661,10661,10624,10624,10579,10579,10535,10535,10510,10510,10473,10473,10466,10466,10414,10414,10379,10379,10312,10312,10306,10306,10234,10234,10117,10117,10084,10084,10046,10046,9998,9998,9976,9976,9961,9961,9960,9960,9907,9907,9899,9899,9898,9898,9848,9848,9828,9828,9826,9826,9815,9815,9770,9770,9746,9746,9722,9722,9648,9648,9599,9599,9516,9516,9506,9506,9497,9497,9492,9492,9447,9447,9393,9393,9388,9388,9385,9385,9368,9368,9337,9337,9332,9332,9327,9327,9314,9314,9292,9292,9151,9151,9142,9142,9127,9127,9116,9116,9054,9054,9052,9052,9040,9040,8998,8998,8966,8966,8965,8965,8949,8949,8911,8911,8906,8906,8893,8893,8878,8878,8859,8859,8846,8846,8842,8842,8826,8826,8787,8787,8721,8721,8717,8717,8716,8716,8703,8703,8696,8696,8695,8695,8670,8670,8656,8656,8655,8655,8633,8633,8625,8625,8612,8612,8610,8610,8607,8607,8591,8591,8588,8588,8580,8580,8568,8568,8564,8564,8562,8562,8553,8553,8534,8534,8520,8520,8503,8503,8480,8480,8474,8474,8466,8466,8463,8463,8457,8457,8442,8442,8437,8437,8435,8435,8430,8430,8418,8418,8411,8411,8382,8382,8377,8377,8376,8376,8371,8371,8362,8362,8354,8354,8348,8348,8343,8343,8335,8335,8313,8313,8288,8288,8255,8255,8237,8237,8226,8226,8218,8218,8208,8208,8205,8205,8201,8201,8199,8199,8196,8196,8174,8174,8168,8168,8165,8165,8146,8146,8143,8143,8122,8122,8115,8115,8107,8107,8093,8093,8092,8092,8088,8088,8083,8083,8068,8068,8055,8055,8051,8051,8041,8041,8036,8036,8010,8010,7981,7981,7977,7977,7976,7976,7955,7955,7906,7906,7888,7888,7887,7887,7885,7885,7872,7872,7858,7858,7855,7855,7849,7849,7847,7847,7846,7846,7823,7823,7806,7806,7790,7790,7786,7786,7785,7785,7783,7783,7773,7773,7767,7767,7766,7766,7763,7763,7759,7759,7758,7758,7753,7753,7752,7752,7728,7728,7722,7722,7704,7704,7675,7675,7674,7674,7670,7670,7667,7667,7664,7664,7643,7643,7623,7623,7619,7619,7616,7616,7591,7591,7578,7578,7575,7575,7574,7574,7571,7571,7569,7569,7559,7559,7556,7556,7554,7554,7541,7541,7524,7524,7523,7523,7512,7512,7508,7508,7507,7507,7506,7506,7505,7505,7499,7499,7498,7498,7497,7497,7489,7489,7480,7480,7474,7474,7465,7465,7453,7453,7449,7449,7446,7446,7442,7442,7438,7438,7436,7436,7423,7423,7420,7420,7414,7414,7396,7396,7382,7382,7380,7380,7377,7377,7373,7373,7370,7370,7363,7363,7353,7353,7342,7342,7337,7337,7327,7327,7319,7319,7308,7308,7296,7296,7283,7283,7282,7282,7272,7272,7267,7267,7265,7265,7262,7262,7260,7260,7248,7248,7239,7239,7228,7228,7226,7226,7219,7219,7218,7218,7215,7215,7212,7212,7209,7209,7203,7203,7196,7196,7193,7193,7192,7192,7190,7190,7175,7175,7152,7152,7151,7151,7142,7142,7137,7137,7125,7125,7122,7122,7120,7120,7117,7117,7105,7105,7104,7104,7101,7101,7094,7094,7087,7087,7078,7078,7074,7074,7069,7069,7068,7068,7065,7065,7055,7055,7051,7051,7050,7050,7032,7032,7028,7028,7015,7015,7011,7011,7007,7007,7002,7002,6994,6994,6981,6981,6974,6974,6965,6965,6956,6956,6948,6948,6932,6932,6931,6931,6921,6921,6915,6915,6914,6914,6910,6910,6895,6895,6885,6885,6874,6874,6852,6852,6849,6849,6848,6848,6847,6847,6846,6846,6844,6844,6836,6836,6830,6830,6828,6828,6826,6826,6809,6809,6807,6807,6804,6804,6796,6796,6793,6793,6788,6788,6777,6777,6766,6766,6760,6760,6759,6759,6756,6756,6754,6754,6747,6747,6745,6745,6738,6738,6735,6735,6725,6725,6723,6723,6722,6722,6716,6716,6707,6707,6699,6699,6690,6690,6685,6685,6684,6684,6683,6683,6669,6669,6661,6661,6660,6660,6655,6655,6654,6654,6653,6653,6651,6651,6645,6645,6644,6644,6638,6638,6629,6629,6628,6628,6621,6621,6620,6620,6618,6618,6617,6617,6614,6614,6606,6606,6603,6603,6602,6602,6579,6579,6576,6576,6570,6570,6564,6564,6563,6563,6557,6557,6555,6555,6550,6550,6549,6549,6536,6536,6523,6523,6502,6502,6501,6501,6498,6498,6490,6490,6488,6488,6485,6485,6482,6482,6469,6469,6467,6467,6466,6466,6465,6465,6451,6451,6448,6448,6442,6442,6439,6439,6437,6437,6419,6419,6414,6414,6406,6406,6400,6400,6393,6393,6391,6391,6387,6387,6383,6383,6382,6382,6379,6379,6374,6374,6371,6371,6370,6370,6366,6366,6350,6350,6348,6348,6347,6347,6341,6341,6340,6340,6336,6336,6335,6335,6334,6334,6330,6330,6328,6328,6318,6318,6314,6314,6313,6313,6307,6307,6305,6305,6300,6300,6299,6299,6281,6281,6279,6279,6278,6278,6261,6261,6254,6254,6247,6247,6246,6246,6239,6239,6216,6216,6209,6209,6208,6208,6204,6204,6193,6193,6192,6192,6186,6186,6180,6180,6165,6165,6164,6164,6162,6162,6160,6160,6154,6154,6153,6153,6150,6150,6149,6149,6145,6145,6144,6144,6138,6138,6137,6137,6135,6135,6133,6133,6128,6128,6125,6125,6113,6113,6109,6109,6107,6107,6105,6105,6103,6103,6102,6102,6100,6100,6096,6096,6092,6092,6086,6086,6084,6084,6080,6080,6079,6079,6077,6077,6076,6076,6072,6072,6067,6067,6047,6047,6042,6042,6023,6023,6016,6016,6013,6013,6008,6008,6003,6003,5991,5991,5986,5986,5983,5983,5979,5979,5972,5972,5964,5964,5963,5963,5958,5958,5955,5955,5949,5949,5938,5938,5936,5936,5933,5933,5927,5927,5923,5923,5909,5909,5902,5902,5900,5900,5899,5899,5898,5898,5897,5897,5894,5894,5881,5881,5876,5876,5863,5863,5862,5862,5861,5861,5857,5857,5855,5855,5853,5853,5848,5848,5845,5845,5844,5844,5841,5841,5839,5839,5834,5834,5827,5827,5825,5825,5824,5824,5823,5823,5821,5821,5820,5820,5819,5819,5818,5818,5813,5813,5811,5811,5806,5806,5801,5801,5800,5800,5799,5799,5797,5797,5796,5796,5793,5793,5792,5792,5791,5791,5781,5781,5778,5778,5777,5777,5775,5775,5769,5769,5767,5767,5765,5765,5762,5762,5759,5759,5756,5756,5754,5754,5752,5752,5750,5750,5748,5748,5741,5741,5738,5738,5737,5737,5736,5736,5731,5731,5728,5728,5724,5724,5723,5723,5719,5719,5718,5718,5711,5711,5706,5706,5699,5699,5697,5697,5685,5685,5684,5684,5680,5680,5679,5679,5678,5678,5676,5676,5672,5672,5663,5663,5661,5661,5655,5655,5650,5650,5646,5646,5641,5641,5635,5635,5631,5631,5626,5626,5625,5625,5619,5619,5618,5618,5616,5616,5610,5610,5604,5604,5599,5599,5596,5596,5592,5592,5591,5591,5589,5589,5585,5585,5583,5583,5581,5581,5580,5580,5577,5577,5575,5575,5569,5569,5563,5563,5562,5562,5561,5561,5560,5560,5555,5555,5549,5549,5547,5547,5538,5538,5534,5534,5524,5524,5523,5523,5514,5514,5511,5511,5509,5509,5506,5506,5504,5504,5500,5500,5496,5496,5494,5494,5493,5493,5490,5490,5488,5488,5480,5480,5476,5476,5473,5473,5472,5472,5469,5469,5468,5468,5467,5467,5466,5466,5465,5465,5462,5462,5456,5456,5455,5455,5453,5453,5452,5452,5451,5451,5447,5447,5446,5446,5445,5445,5436,5436,5428,5428,5426,5426,5425,5425,5417,5417,5416,5416,5415,5415,5414,5414,5413,5413,5411,5411,5410,5410,5409,5409,5408,5408,5407,5407,5406,5406,5405,5405,5404,5404,5399,5399,5398,5398,5397,5397,5389,5389,5388,5388,5387,5387,5386,5386,5385,5385,5380,5380,5377,5377,5375,5375,5374,5374,5373,5373,5368,5368,5354,5354,5353,5353,5352,5352,5348,5348,5346,5346,5342,5342,5339,5339,5338,5338,5330,5330,5329,5329,5326,5326,5320,5320,5319,5319,5312,5312,5309,5309,5308,5308,5304,5304,5303,5303,5300,5300,5297,5297,5295,5295,5294,5294,5290,5290,5288,5288,5287,5287,5285,5285,5284,5284,5283,5283,5280,5280,5279,5279,5271,5271,5270,5270,5266,5266,5265,5265,5264,5264,5258,5258,5257,5257,5255,5255,5254,5254,5253,5253,5251,5251,5244,5244,5240,5240,5237,5237,5235,5235,5234,5234,5232,5232,5228,5228,5227,5227,5226,5226,5222,5222,5221,5221,5218,5218,5217,5217,5214,5214,5209,5209,5201,5201,5200,5200,5199,5199,5185,5185,5182,5182,5179,5179,5178,5178,5170,5170,5169,5169,5168,5168,5165,5165,5163,5163,5160,5160,5159,5159,5157,5157,5154,5154,5153,5153,5149,5149,5148,5148,5144,5144,5142,5142,5141,5141,5139,5139,5136,5136,5133,5133,5132,5132,5131,5131,5126,5126,5125,5125,5120,5120,5117,5117,5114,5114,5110,5110,5104,5104,5103,5103,5102,5102,5100,5100,5095,5095,5089,5089,5087,5087,5084,5084,5083,5083,5065,5065,5064,5064,5060,5060,5057,5057,5056,5056,5054,5054,5051,5051,5050,5050,5047,5047,5045,5045,5030,5030,5022,5022,5021,5021,5018,5018,5013,5013,5011,5011,5008,5008,5007,5007,5006,5006,5004,5004,5003,5003,5002,5002,5001,5001,4997,4997,4993,4993,4989,4989,4988,4988,4984,4984,4982,4982,4981,4981,4979,4979,4975,4975,4974,4974,4973,4973,4972,4972,4971,4971,4964,4964,4955,4955,4951,4951,4949,4949,4946,4946,4944,4944,4942,4942,4941,4941,4940,4940,4939,4939,4935,4935,4934,4934,4928,4928,4923,4923,4922,4922,4918,4918,4917,4917,4913,4913,4912,4912,4911,4911,4909,4909,4908,4908,4907,4907,4906,4906,4904,4904,4902,4902,4899,4899,4896,4896,4893,4893,4892,4892,4891,4891,4885,4885,4883,4883,4877,4877,4874,4874,4873,4873,4872,4872,4867,4867,4866,4866,4865,4865,4862,4862,4861,4861,4859,4859,4854,4854,4850,4850,4845,4845,4844,4844,4843,4843,4840,4840,4836,4836,4834,4834,4832,4832,4831,4831,4827,4827,4825,4825,4822,4822,4821,4821,4817,4817,4813,4813,4807,4807,4806,4806,4805,4805,4804,4804,4799,4799,4795,4795,4794,4794,4789,4789,4785,4785,4782,4782,4773,4773,4772,4772,4769,4769,4767,4767,4764,4764,4760,4760,4759,4759,4756,4756,4755,4755,4753,4753,4751,4751,4747,4747,4746,4746,4741,4741,4740,4740,4738,4738,4735,4735,4732,4732,4731,4731,4729,4729,4727,4727,4726,4726,4723,4723,4721,4721,4719,4719,4718,4718,4708,4708,4701,4701,4699,4699,4698,4698,4696,4696,4694,4694,4691,4691,4690,4690,4689,4689,4685,4685,4684,4684,4677,4677,4672,4672,4671,4671,4669,4669,4665,4665,4664,4664,4661,4661,4659,4659,4658,4658,4656,4656,4654,4654,4653,4653,4645,4645,4639,4639,4637,4637,4636,4636,4635,4635,4632,4632,4629,4629,4625,4625,4623,4623,4622,4622,4620,4620,4617,4617,4615,4615,4608,4608,4605,4605,4603,4603,4602,4602,4600,4600,4596,4596,4595,4595,4594,4594,4593,4593,4591,4591,4585,4585,4582,4582,4581,4581,4580,4580,4579,4579,4574,4574,4573,4573,4567,4567,4566,4566,4565,4565,4563,4563,4557,4557,4556,4556,4555,4555,4553,4553,4552,4552,4550,4550,4545,4545,4542,4542,4541,4541,4537,4537,4535,4535,4532,4532,4529,4529,4527,4527,4525,4525,4521,4521,4519,4519,4514,4514,4513,4513,4512,4512,4510,4510,4507,4507,4506,4506,4504,4504,4503,4503,4499,4499,4495,4495,4492,4492,4491,4491,4489,4489,4486,4486,4483,4483,4481,4481,4478,4478,4477,4477,4471,4471,4470,4470,4468,4468,4461,4461,4458,4458,4457,4457,4456,4456,4451,4451,4450,4450,4449,4449,4448,4448,4447,4447,4446,4446,4444,4444,4442,4442,4441,4441,4434,4434,4431,4431,4428,4428,4426,4426,4425,4425,4424,4424,4422,4422,4419,4419,4418,4418,4415,4415,4413,4413,4410,4410,4408,4408,4407,4407,4402,4402,4399,4399,4398,4398,4397,4397,4394,4394,4391,4391,4390,4390,4389,4389,4388,4388,4386,4386,4380,4380,4379,4379,4374,4374,4373,4373,4371,4371,4369,4369,4365,4365,4364,4364,4363,4363,4362,4362,4360,4360,4358,4358,4355,4355,4353,4353,4352,4352,4351,4351,4350,4350,4349,4349,4348,4348,4346,4346,4345,4345,4342,4342,4339,4339,4336,4336,4332,4332,4331,4331,4327,4327,4322,4322,4321,4321,4320,4320,4318,4318,4316,4316,4313,4313,4311,4311,4310,4310,4304,4304,4303,4303,4302,4302,4299,4299,4296,4296,4295,4295,4292,4292,4291,4291,4288,4288,4287,4287,4285,4285,4283,4283,4282,4282,4280,4280,4279,4279,4278,4278,4277,4277,4274,4274,4273,4273,4267,4267,4266,4266,4263,4263,4262,4262,4261,4261,4259,4259,4258,4258,4255,4255,4252,4252,4251,4251,4248,4248,4245,4245,4243,4243,4241,4241,4240,4240,4238,4238,4236,4236,4234,4234,4233,4233,4231,4231,4230,4230,4229,4229,4228,4228,4227,4227,4226,4226,4224,4224,4223,4223,4222,4222,4220,4220,4218,4218,4212,4212,4211,4211,4210,4210,4208,4208,4207,4207,4206,4206,4205,4205,4204,4204,4201,4201,4200,4200,4197,4197,4196,4196,4194,4194,4192,4192,4191,4191,4190,4190,4186,4186,4185,4185,4184,4184,4183,4183,4181,4181,4180,4180,4178,4178,4174,4174,4173,4173,4171,4171,4170,4170,4168,4168,4166,4166,4165,4165,4162,4162,4161,4161,4157,4157,4154,4154,4153,4153,4152,4152,4151,4151,4150,4150,4149,4149,4148,4148,4147,4147,4145,4145,4144,4144,4143,4143,4142,4142,4141,4141,4140,4140,4139,4139,4137,4137,4136,4136,4135,4135,4130,4130,4129,4129,4128,4128,4127,4127,4126,4126,4125,4125,4124,4124,4123,4123,4121,4121,4120,4120,4119,4119,4117,4117,4116,4116,4115,4115,4113,4113,4112,4112,4111,4111,4110,4110,4107,4107,4106,4106,4102,4102,4100,4100,4096,4096,4094,4094,4091,4091,4086,4086,4084,4084,4082,4082,4081,4081,4078,4078,4076,4076,4075,4075,4073,4073,4072,4072,4071,4071,4069,4069,4068,4068,4067,4067,4066,4066,4062,4062,4061,4061,4060,4060,4059,4059,4058,4058,4052,4052,4051,4051,4048,4048,4047,4047,4046,4046,4045,4045,4043,4043,4042,4042,4040,4040,4039,4039,4038,4038,4037,4037,4036,4036,4034,4034,4032,4032,4031,4031,4030,4030,4029,4029,4026,4026,4025,4025,4022,4022,4020,4020,4018,4018,4016,4016,4015,4015,4014,4014,4013,4013,4012,4012,4011,4011,4008,4008,4007,4007,4005,4005,4003,4003,4002,4002,3998,3998,3996,3996,3992,3992,3990,3990,3988,3988,3987,3987,3983,3983,3982,3982,3978,3978,3976,3976,3975,3975,3970,3970,3968,3968,3966,3966,3965,3965,3962,3962,3961,3961,3959,3959,3956,3956,3955,3955,3953,3953,3952,3952,3950,3950,3949,3949,3948,3948,3947,3947,3946,3946,3945,3945,3944,3944,3943,3943,3941,3941,3939,3939,3938,3938,3937,3937,3936,3936,3934,3934,3933,3933,3931,3931,3929,3929,3926,3926,3924,3924,3921,3921,3919,3919,3918,3918,3917,3917,3916,3916,3915,3915,3913,3913,3912,3912,3909,3909,3907,3907,3906,3906,3905,3905,3904,3904,3902,3902,3899,3899,3898,3898,3897,3897,3896,3896,3895,3895,3894,3894,3891,3891,3889,3889,3884,3884,3883,3883,3882,3882,3879,3879,3878,3878,3876,3876,3870,3870,3869,3869,3868,3868,3867,3867,3864,3864,3862,3862,3859,3859,3858,3858,3857,3857,3856,3856,3855,3855,3853,3853,3852,3852,3850,3850,3849,3849,3845,3845,3844,3844,3843,3843,3842,3842,3841,3841,3840,3840,3839,3839,3837,3837,3836,3836,3834,3834,3832,3832,3831,3831,3830,3830,3828,3828,3827,3827,3826,3826,3823,3823,3822,3822,3820,3820,3819,3819,3818,3818,3815,3815,3814,3814,3813,3813,3810,3810,3808,3808,3807,3807,3806,3806,3802,3802,3801,3801,3800,3800,3797,3797,3796,3796,3795,3795,3793,3793,3792,3792,3791,3791,3788,3788,3787,3787,3786,3786,3780,3780,3779,3779,3778,3778,3777,3777,3776,3776,3773,3773,3771,3771,3770,3770,3768,3768,3767,3767,3764,3764,3763,3763,3762,3762,3761,3761,3760,3760,3758,3758,3757,3757,3756,3756,3754,3754,3753,3753,3752,3752,3750,3750,3749,3749,3747,3747,3745,3745,3743,3743,3742,3742,3740,3740,3734,3734,3733,3733,3731,3731,3730,3730,3729,3729,3728,3728,3727,3727,3724,3724,3723,3723,3722,3722,3719,3719,3718,3718,3717,3717,3716,3716,3715,3715,3714,3714,3711,3711,3710,3710,3709,3709,3705,3705,3704,3704,3701,3701,3699,3699,3698,3698,3697,3697,3696,3696,3695,3695,3693,3693,3692,3692,3691,3691,3690,3690,3689,3689,3688,3688,3686,3686,3684,3684,3682,3682,3680,3680,3679,3679,3678,3678,3677,3677,3675,3675,3674,3674,3673,3673,3668,3668,3667,3667,3666,3666,3665,3665,3663,3663,3662,3662,3661,3661,3660,3660,3658,3658,3656,3656,3655,3655,3654,3654,3653,3653,3652,3652,3651,3651,3650,3650,3649,3649,3647,3647,3642,3642,3641,3641,3640,3640,3639,3639,3637,3637,3636,3636,3635,3635,3634,3634,3633,3633,3632,3632,3628,3628,3627,3627,3625,3625,3624,3624,3623,3623,3621,3621,3620,3620,3617,3617,3615,3615,3613,3613,3612,3612,3611,3611,3610,3610,3608,3608,3606,3606,3605,3605,3604,3604,3603,3603,3602,3602,3601,3601,3600,3600,3599,3599,3598,3598,3597,3597,3596,3596,3593,3593,3591,3591,3589,3589,3587,3587,3584,3584,3583,3583,3582,3582,3581,3581,3579,3579,3578,3578,3576,3576,3575,3575,3573,3573,3572,3572,3571,3571,3569,3569,3566,3566,3565,3565,3563,3563,3560,3560,3559,3559,3557,3557,3556,3556,3555,3555,3551,3551,3550,3550,3545,3545,3543,3543,3541,3541,3540,3540,3538,3538,3536,3536,3534,3534,3529,3529,3527,3527,3526,3526,3522,3522,3521,3521,3520,3520,3519,3519,3518,3518,3517,3517,3515,3515,3514,3514,3513,3513,3511,3511,3510,3510,3504,3504,3502,3502,3501,3501,3500,3500,3498,3498,3495,3495,3493,3493,3492,3492,3491,3491,3490,3490,3489,3489,3486,3486,3484,3484,3483,3483,3482,3482,3481,3481,3480,3480,3479,3479,3477,3477,3476,3476,3474,3474,3472,3472,3470,3470,3469,3469,3468,3468,3465,3465,3464,3464,3462,3462,3461,3461,3459,3459,3457,3457,3456,3456,3455,3455,3454,3454,3453,3453,3452,3452,3450,3450,3448,3448,3447,3447,3446,3446,3445,3445,3444,3444,3443,3443,3442,3442,3440,3440,3439,3439,3438,3438,3437,3437,3435,3435,3434,3434,3428,3428,3427,3427,3426,3426,3425,3425,3424,3424,3423,3423,3422,3422,3421,3421,3420,3420,3416,3416,3415,3415,3414,3414,3412,3412,3409,3409,3408,3408,3407,3407,3406,3406,3403,3403,3402,3402,3401,3401,3400,3400,3399,3399,3398,3398,3397,3397,3396,3396,3394,3394,3393,3393,3390,3390,3389,3389,3385,3385,3384,3384,3381,3381,3380,3380,3379,3379,3378,3378,3377,3377,3376,3376,3375,3375,3374,3374,3373,3373,3372,3372,3371,3371,3370,3370,3368,3368,3367,3367,3366,3366,3363,3363,3361,3361,3360,3360,3359,3359,3358,3358,3357,3357,3356,3356,3355,3355,3354,3354,3353,3353,3352,3352,3351,3351,3349,3349,3348,3348,3347,3347,3346,3346,3344,3344,3343,3343,3342,3342,3341,3341,3340,3340,3338,3338,3337,3337,3335,3335,3334,3334,3333,3333,3331,3331,3330,3330,3328,3328,3327,3327,3326,3326,3325,3325,3324,3324,3323,3323,3322,3322,3321,3321,3320,3320,3319,3319,3314,3314,3313,3313,3312,3312,3310,3310,3309,3309,3308,3308,3307,3307,3305,3305,3302,3302,3301,3301,3300,3300,3299,3299,3296,3296,3293,3293,3292,3292,3291,3291,3290,3290,3289,3289,3286,3286,3284,3284,3283,3283,3281,3281,3280,3280,3279,3279,3278,3278,3277,3277,3276,3276,3275,3275,3274,3274,3272,3272,3271,3271,3270,3270,3269,3269,3268,3268,3265,3265,3264,3264,3263,3263,3257,3257,3256,3256,3255,3255,3254,3254,3253,3253,3252,3252,3251,3251,3250,3250,3249,3249,3248,3248,3247,3247,3246,3246,3243,3243,3242,3242,3241,3241,3238,3238,3235,3235,3234,3234,3232,3232,3231,3231,3229,3229,3228,3228,3226,3226,3223,3223,3221,3221,3219,3219,3218,3218,3217,3217,3216,3216,3215,3215,3214,3214,3213,3213,3210,3210,3208,3208,3207,3207,3206,3206,3204,3204,3202,3202,3201,3201,3200,3200,3199,3199,3195,3195,3194,3194,3193,3193,3190,3190,3189,3189,3188,3188,3187,3187,3186,3186,3185,3185,3184,3184,3183,3183,3182,3182,3180,3180,3178,3178,3177,3177,3176,3176,3174,3174,3173,3173,3171,3171,3168,3168,3167,3167,3166,3166,3163,3163,3162,3162,3161,3161,3160,3160,3159,3159,3158,3158,3157,3157,3156,3156,3154,3154,3151,3151,3149,3149,3146,3146,3144,3144,3143,3143,3141,3141,3140,3140,3139,3139,3136,3136,3133,3133,3132,3132,3131,3131,3130,3130,3129,3129,3128,3128,3127,3127,3125,3125,3124,3124,3123,3123,3122,3122,3121,3121,3118,3118,3117,3117,3116,3116,3115,3115,3114,3114,3113,3113,3112,3112,3111,3111,3110,3110,3104,3104,3103,3103,3102,3102,3101,3101,3099,3099,3097,3097,3096,3096,3095,3095,3094,3094,3092,3092,3091,3091,3088,3088,3086,3086,3085,3085,3083,3083,3081,3081,3080,3080,3079,3079,3078,3078,3076,3076,3075,3075,3074,3074,3073,3073,3070,3070,3068,3068,3067,3067,3066,3066,3065,3065,3064,3064,3063,3063,3062,3062,3060,3060,3059,3059,3055,3055,3054,3054,3053,3053,3051,3051,3050,3050,3049,3049,3048,3048,3047,3047,3046,3046,3045,3045,3044,3044,3043,3043,3042,3042,3040,3040,3039,3039,3038,3038,3037,3037,3035,3035,3034,3034,3033,3033,3032,3032,3031,3031,3028,3028,3025,3025,3023,3023,3022,3022,3020,3020,3017,3017,3016,3016,3014,3014,3012,3012,3009,3009,3007,3007,3006,3006,3005,3005,3004,3004,3003,3003,3001,3001,2999,2999,2998,2998,2996,2996,2993,2993,2992,2992,2991,2991,2990,2990,2989,2989,2988,2988,2985,2985,2983,2983,2982,2982,2981,2981,2980,2980,2979,2979,2978,2978,2977,2977,2975,2975,2974,2974,2973,2973,2972,2972,2971,2971,2970,2970,2967,2967,2966,2966,2965,2965,2964,2964,2963,2963,2962,2962,2961,2961,2960,2960,2959,2959,2958,2958,2956,2956,2953,2953,2952,2952,2951,2951,2950,2950,2949,2949,2947,2947,2946,2946,2945,2945,2944,2944,2943,2943,2941,2941,2940,2940,2939,2939,2938,2938,2937,2937,2936,2936,2935,2935,2934,2934,2933,2933,2932,2932,2931,2931,2930,2930,2929,2929,2928,2928,2927,2927,2926,2926,2924,2924,2923,2923,2922,2922,2921,2921,2920,2920,2919,2919,2917,2917,2915,2915,2914,2914,2912,2912,2911,2911,2910,2910,2905,2905,2904,2904,2901,2901,2900,2900,2899,2899,2898,2898,2897,2897,2896,2896,2895,2895,2893,2893,2892,2892,2890,2890,2888,2888,2886,2886,2885,2885,2884,2884,2882,2882,2881,2881,2880,2880,2879,2879,2878,2878,2877,2877,2876,2876,2875,2875,2874,2874,2873,2873,2872,2872,2871,2871,2869,2869,2868,2868,2867,2867,2865,2865,2864,2864,2863,2863,2860,2860,2859,2859,2857,2857,2856,2856,2854,2854,2853,2853,2852,2852,2848,2848,2846,2846,2844,2844,2842,2842,2841,2841,2840,2840,2839,2839,2838,2838,2837,2837,2836,2836,2835,2835,2834,2834,2833,2833,2832,2832,2831,2831,2830,2830,2829,2829,2828,2828,2826,2826,2823,2823,2822,2822,2820,2820,2819,2819,2817,2817,2815,2815,2814,2814,2813,2813,2812,2812,2808,2808,2807,2807,2805,2805,2804,2804,2803,2803,2802,2802,2801,2801,2799,2799,2798,2798,2797,2797,2796,2796,2795,2795,2794,2794,2793,2793,2791,2791,2789,2789,2787,2787,2786,2786,2785,2785,2784,2784,2783,2783,2782,2782,2781,2781,2779,2779,2777,2777,2775,2775,2774,2774,2773,2773,2772,2772,2771,2771,2769,2769,2768,2768,2767,2767,2766,2766,2765,2765,2764,2764,2763,2763,2762,2762,2761,2761,2756,2756,2755,2755,2754,2754,2753,2753,2752,2752,2751,2751,2750,2750,2747,2747,2746,2746,2745,2745,2743,2743,2742,2742,2741,2741,2740,2740,2738,2738,2735,2735,2734,2734,2733,2733,2732,2732,2731,2731,2730,2730,2729,2729,2728,2728,2727,2727,2726,2726,2725,2725,2724,2724,2723,2723,2722,2722,2721,2721,2720,2720,2718,2718,2717,2717,2715,2715,2714,2714,2712,2712,2710,2710,2709,2709,2707,2707,2704,2704,2703,2703,2700,2700,2698,2698,2697,2697,2695,2695,2693,2693,2692,2692,2691,2691,2690,2690,2689,2689,2688,2688,2687,2687,2686,2686,2685,2685,2684,2684,2683,2683,2682,2682,2680,2680,2679,2679,2678,2678,2677,2677,2673,2673,2672,2672,2671,2671,2670,2670,2669,2669,2668,2668,2666,2666,2665,2665,2664,2664,2663,2663,2662,2662,2661,2661,2660,2660,2658,2658,2657,2657,2656,2656,2655,2655,2653,2653,2650,2650,2649,2649,2647,2647,2646,2646,2645,2645,2644,2644,2643,2643,2641,2641,2640,2640,2639,2639,2638,2638,2637,2637,2633,2633,2632,2632,2631,2631,2630,2630,2629,2629,2627,2627,2626,2626,2625,2625,2624,2624,2623,2623,2622,2622,2621,2621,2620,2620,2619,2619,2618,2618,2617,2617,2616,2616,2615,2615,2614,2614,2613,2613,2612,2612,2610,2610,2609,2609,2607,2607,2605,2605,2604,2604,2601,2601,2600,2600,2599,2599,2598,2598,2597,2597,2596,2596,2595,2595,2590,2590,2589,2589,2588,2588,2587,2587,2586,2586,2584,2584,2583,2583,2582,2582,2579,2579,2578,2578,2577,2577,2576,2576,2575,2575,2574,2574,2572,2572,2571,2571,2570,2570,2569,2569,2568,2568,2567,2567,2566,2566,2565,2565,2564,2564,2562,2562,2558,2558,2557,2557,2556,2556,2555,2555,2554,2554,2553,2553,2552,2552,2551,2551,2550,2550,2549,2549,2548,2548,2547,2547,2546,2546,2545,2545,2544,2544,2543,2543,2542,2542,2541,2541,2539,2539,2538,2538,2537,2537,2536,2536,2535,2535,2534,2534,2533,2533,2532,2532,2530,2530,2529,2529,2528,2528,2527,2527,2526,2526,2525,2525,2524,2524,2522,2522,2520,2520,2518,2518,2516,2516,2515,2515,2514,2514,2513,2513,2512,2512,2511,2511,2510,2510,2509,2509,2508,2508,2506,2506,2505,2505,2504,2504,2503,2503,2502,2502,2501,2501,2500,2500,2499,2499,2498,2498,2497,2497,2495,2495,2494,2494,2492,2492,2491,2491,2490,2490,2489,2489,2488,2488,2487,2487,2486,2486,2484,2484,2483,2483,2482,2482,2481,2481,2480,2480,2479,2479,2478,2478,2477,2477,2476,2476,2474,2474,2471,2471,2470,2470,2469,2469,2467,2467,2464,2464,2463,2463,2462,2462,2461,2461,2459,2459,2456,2456,2454,2454,2452,2452,2449,2449,2448,2448,2447,2447,2446,2446,2444,2444,2443,2443,2442,2442,2441,2441,2440,2440,2439,2439,2438,2438,2437,2437,2435,2435,2434,2434,2432,2432,2431,2431,2429,2429,2428,2428,2427,2427,2426,2426,2425,2425,2424,2424,2423,2423,2422,2422,2421,2421,2420,2420,2419,2419,2418,2418,2416,2416,2415,2415,2414,2414,2412,2412,2411,2411,2409,2409,2407,2407,2406,2406,2405,2405,2404,2404,2403,2403,2402,2402,2401,2401,2400,2400,2398,2398,2396,2396,2395,2395,2393,2393,2392,2392,2391,2391,2389,2389,2388,2388,2386,2386,2385,2385,2384,2384,2383,2383,2382,2382,2381,2381,2379,2379,2378,2378,2377,2377,2376,2376,2374,2374,2373,2373,2370,2370,2369,2369,2368,2368,2367,2367,2366,2366,2365,2365,2364,2364,2361,2361,2360,2360,2358,2358,2357,2357,2356,2356,2355,2355,2354,2354,2353,2353,2352,2352,2351,2351,2350,2350,2349,2349,2348,2348,2347,2347,2346,2346,2345,2345,2344,2344,2343,2343,2341,2341,2340,2340,2339,2339,2337,2337,2335,2335,2334,2334,2333,2333,2332,2332,2331,2331,2328,2328,2327,2327,2326,2326,2325,2325,2324,2324,2323,2323,2322,2322,2321,2321,2320,2320,2319,2319,2316,2316,2314,2314,2313,2313,2312,2312,2311,2311,2310,2310,2309,2309,2307,2307,2305,2305,2304,2304,2303,2303,2302,2302,2301,2301,2300,2300,2299,2299,2298,2298,2296,2296,2295,2295,2294,2294,2293,2293,2292,2292,2291,2291,2289,2289,2288,2288,2287,2287,2286,2286,2284,2284,2283,2283,2282,2282,2281,2281,2280,2280,2279,2279,2278,2278,2277,2277,2276,2276,2275,2275,2273,2273,2271,2271,2270,2270,2269,2269,2268,2268,2267,2267,2266,2266,2265,2265,2264,2264,2263,2263,2262,2262,2261,2261,2260,2260,2259,2259,2257,2257,2255,2255,2254,2254,2253,2253,2252,2252,2251,2251,2250,2250,2249,2249,2248,2248,2247,2247,2246,2246,2245,2245,2244,2244,2243,2243,2242,2242,2241,2241,2240,2240,2239,2239,2237,2237,2236,2236,2235,2235,2234,2234,2233,2233,2232,2232,2231,2231,2229,2229,2228,2228,2227,2227,2226,2226,2225,2225,2224,2224,2223,2223,2222,2222,2221,2221,2220,2220,2219,2219,2217,2217,2214,2214,2213,2213,2212,2212,2211,2211,2210,2210,2209,2209,2208,2208,2207,2207,2206,2206,2204,2204,2203,2203,2202,2202,2201,2201,2199,2199,2198,2198,2196,2196,2193,2193,2192,2192,2190,2190,2189,2189,2187,2187,2186,2186,2185,2185,2184,2184,2183,2183,2182,2182,2181,2181,2180,2180,2179,2179,2178,2178,2177,2177,2176,2176,2175,2175,2173,2173,2172,2172,2171,2171,2170,2170,2169,2169,2168,2168,2167,2167,2165,2165,2164,2164,2163,2163,2162,2162,2161,2161,2160,2160,2159,2159,2158,2158,2157,2157,2155,2155,2154,2154,2153,2153,2151,2151,2150,2150,2149,2149,2148,2148,2147,2147,2145,2145,2143,2143,2142,2142,2141,2141,2140,2140,2139,2139,2138,2138,2136,2136,2135,2135,2134,2134,2132,2132,2130,2130,2129,2129,2128,2128,2127,2127,2126,2126,2125,2125,2124,2124,2123,2123,2122,2122,2121,2121,2120,2120,2119,2119,2118,2118,2117,2117,2116,2116,2115,2115,2113,2113,2112,2112,2111,2111,2110,2110,2109,2109,2108,2108,2107,2107,2106,2106,2104,2104,2103,2103,2102,2102,2100,2100,2098,2098,2097,2097,2096,2096,2095,2095,2094,2094,2093,2093,2092,2092,2091,2091,2089,2089,2088,2088,2087,2087,2086,2086,2085,2085,2083,2083,2082,2082,2081,2081,2080,2080,2079,2079,2078,2078,2077,2077,2076,2076,2075,2075,2073,2073,2071,2071,2070,2070,2069,2069,2068,2068,2067,2067,2066,2066,2065,2065,2064,2064,2063,2063,2062,2062,2060,2060,2059,2059,2058,2058,2057,2057,2054,2054,2053,2053,2052,2052,2051,2051,2050,2050,2049,2049,2048,2048,2047,2047,2045,2045,2044,2044,2043,2043,2042,2042,2041,2041,2040,2040,2039,2039,2038,2038,2037,2037,2035,2035,2034,2034,2033,2033,2032,2032,2031,2031,2030,2030,2029,2029,2028,2028,2027,2027,2025,2025,2024,2024,2023,2023,2022,2022,2021,2021,2020,2020,2019,2019,2018,2018,2017,2017,2016,2016,2015,2015,2014,2014,2013,2013,2012,2012,2011,2011,2010,2010,2009,2009,2008,2008,2007,2007,2006,2006,2005,2005,2004,2004,2003,2003,2001,2001,2000,2000,1999,1999,1997,1997,1996,1996,1995,1995,1994,1994,1993,1993,1992,1992,1991,1991,1990,1990,1989,1989,1988,1988,1987,1987,1986,1986,1985,1985,1983,1983,1979,1979,1978,1978,1977,1977,1976,1976,1975,1975,1974,1974,1973,1973,1972,1972,1971,1971,1970,1970,1969,1969,1968,1968,1967,1967,1966,1966,1965,1965,1964,1964,1963,1963,1962,1962,1961,1961,1960,1960,1959,1959,1958,1958,1957,1957,1956,1956,1955,1955,1953,1953,1951,1951,1950,1950,1949,1949,1948,1948,1947,1947,1945,1945,1943,1943,1942,1942,1941,1941,1940,1940,1939,1939,1938,1938,1936,1936,1935,1935,1934,1934,1933,1933,1932,1932,1931,1931,1930,1930,1929,1929,1928,1928,1927,1927,1926,1926,1925,1925,1924,1924,1923,1923,1922,1922,1921,1921,1920,1920,1919,1919,1918,1918,1917,1917,1915,1915,1914,1914,1913,1913,1912,1912,1911,1911,1909,1909,1908,1908,1907,1907,1906,1906,1904,1904,1903,1903,1902,1902,1901,1901,1900,1900,1897,1897,1896,1896,1895,1895,1894,1894,1893,1893,1892,1892,1891,1891,1890,1890,1889,1889,1888,1888,1887,1887,1886,1886,1885,1885,1884,1884,1882,1882,1881,1881,1880,1880,1879,1879,1878,1878,1877,1877,1876,1876,1875,1875,1874,1874,1872,1872,1871,1871,1870,1870,1868,1868,1867,1867,1866,1866,1865,1865,1864,1864,1863,1863,1861,1861,1860,1860,1859,1859,1858,1858,1856,1856,1855,1855,1854,1854,1853,1853,1852,1852,1851,1851,1850,1850,1849,1849,1848,1848,1847,1847,1846,1846,1845,1845,1844,1844,1843,1843,1842,1842,1841,1841,1840,1840,1839,1839,1837,1837,1836,1836,1835,1835,1833,1833,1832,1832,1831,1831,1829,1829,1828,1828,1827,1827,1826,1826,1825,1825,1821,1821,1820,1820,1819,1819,1818,1818,1817,1817,1816,1816,1815,1815,1814,1814,1813,1813,1812,1812,1811,1811,1810,1810,1809,1809,1808,1808,1807,1807,1806,1806,1805,1805,1804,1804,1803,1803,1802,1802,1801,1801,1800,1800,1799,1799,1798,1798,1795,1795,1794,1794,1793,1793,1792,1792,1791,1791,1790,1790,1789,1789,1788,1788,1787,1787,1786,1786,1784,1784,1783,1783,1782,1782,1781,1781,1780,1780,1778,1778,1776,1776,1774,1774,1773,1773,1772,1772,1771,1771,1770,1770,1769,1769,1768,1768,1767,1767,1765,1765,1764,1764,1761,1761,1760,1760,1759,1759,1758,1758,1757,1757,1755,1755,1754,1754,1753,1753,1752,1752,1751,1751,1750,1750,1749,1749,1748,1748,1747,1747,1746,1746,1745,1745,1744,1744,1743,1743,1742,1742,1741,1741,1740,1740,1739,1739,1738,1738,1737,1737,1736,1736,1735,1735,1734,1734,1733,1733,1732,1732,1731,1731,1729,1729,1727,1727,1726,1726,1725,1725,1724,1724,1723,1723,1722,1722,1721,1721,1720,1720,1719,1719,1718,1718,1717,1717,1716,1716,1715,1715,1714,1714,1713,1713,1712,1712,1711,1711,1710,1710,1709,1709,1708,1708,1707,1707,1706,1706,1705,1705,1704,1704,1703,1703,1701,1701,1700,1700,1699,1699,1698,1698,1696,1696,1695,1695,1694,1694,1693,1693,1692,1692,1691,1691,1690,1690,1689,1689,1688,1688,1687,1687,1686,1686,1685,1685,1684,1684,1683,1683,1682,1682,1681,1681,1680,1680,1677,1677,1676,1676,1675,1675,1674,1674,1673,1673,1672,1672,1670,1670,1669,1669,1668,1668,1666,1666,1665,1665,1664,1664,1663,1663,1662,1662,1661,1661,1660,1660,1659,1659,1658,1658,1657,1657,1656,1656,1655,1655,1654,1654,1653,1653,1652,1652,1650,1650,1649,1649,1648,1648,1647,1647,1646,1646,1645,1645,1644,1644,1643,1643,1641,1641,1640,1640,1639,1639,1638,1638,1636,1636,1635,1635,1634,1634,1633,1633,1631,1631,1630,1630,1629,1629,1628,1628,1627,1627,1626,1626,1625,1625,1624,1624,1623,1623,1622,1622,1621,1621,1620,1620,1619,1619,1618,1618,1616,1616,1615,1615,1614,1614,1613,1613,1612,1612,1611,1611,1610,1610,1609,1609,1608,1608,1606,1606,1605,1605,1604,1604,1603,1603,1602,1602,1601,1601,1600,1600,1599,1599,1598,1598,1596,1596,1595,1595,1593,1593,1592,1592,1591,1591,1590,1590,1589,1589,1588,1588,1587,1587,1586,1586,1585,1585,1584,1584,1583,1583,1582,1582,1581,1581,1580,1580,1578,1578,1576,1576,1575,1575,1574,1574,1573,1573,1572,1572,1571,1571,1570,1570,1569,1569,1568,1568,1567,1567,1566,1566,1565,1565,1564,1564,1563,1563,1562,1562,1561,1561,1560,1560,1559,1559,1558,1558,1557,1557,1556,1556,1555,1555,1554,1554,1553,1553,1552,1552,1551,1551,1550,1550,1548,1548,1547,1547,1546,1546,1545,1545,1544,1544,1542,1542,1539,1539,1538,1538,1537,1537,1536,1536,1535,1535,1534,1534,1533,1533,1531,1531,1530,1530,1529,1529,1528,1528,1527,1527,1526,1526,1524,1524,1523,1523,1522,1522,1521,1521,1520,1520,1519,1519,1518,1518,1517,1517,1516,1516,1515,1515,1514,1514,1513,1513,1512,1512,1510,1510,1508,1508,1507,1507,1506,1506,1505,1505,1503,1503,1502,1502,1501,1501,1499,1499,1498,1498,1496,1496,1495,1495,1494,1494,1492,1492,1491,1491,1490,1490,1489,1489,1487,1487,1485,1485,1483,1483,1482,1482,1481,1481,1480,1480,1479,1479,1478,1478,1477,1477,1476,1476,1475,1475,1474,1474,1473,1473,1472,1472,1471,1471,1470,1470,1469,1469,1468,1468,1467,1467,1466,1466,1465,1465,1464,1464,1462,1462,1461,1461,1459,1459,1458,1458,1457,1457,1455,1455,1454,1454,1453,1453,1452,1452,1451,1451,1450,1450,1449,1449,1448,1448,1447,1447,1446,1446,1444,1444,1443,1443,1441,1441,1440,1440,1439,1439,1438,1438,1437,1437,1436,1436,1435,1435,1434,1434,1433,1433,1432,1432,1431,1431,1428,1428,1427,1427,1426,1426,1425,1425,1424,1424,1423,1423,1422,1422,1421,1421,1420,1420,1419,1419,1418,1418,1417,1417,1416,1416,1415,1415,1413,1413,1411,1411,1410,1410,1409,1409,1408,1408,1406,1406,1405,1405,1404,1404,1402,1402,1401,1401,1400,1400,1399,1399,1398,1398,1397,1397,1396,1396,1395,1395,1394,1394,1393,1393,1392,1392,1391,1391,1390,1390,1389,1389,1388,1388,1387,1387,1385,1385,1384,1384,1383,1383,1382,1382,1381,1381,1380,1380,1379,1379,1378,1378,1377,1377,1376,1376,1375,1375,1374,1374,1373,1373,1372,1372,1371,1371,1369,1369,1368,1368,1366,1366,1365,1365,1364,1364,1363,1363,1362,1362,1361,1361,1360,1360,1359,1359,1358,1358,1357,1357,1355,1355,1352,1352,1350,1350,1348,1348,1347,1347,1346,1346,1345,1345,1344,1344,1343,1343,1342,1342,1341,1341,1340,1340,1339,1339,1337,1337,1336,1336,1335,1335,1334,1334,1333,1333,1332,1332,1331,1331,1330,1330,1329,1329,1327,1327,1326,1326,1325,1325,1324,1324,1323,1323,1322,1322,1321,1321,1320,1320,1319,1319,1318,1318,1317,1317,1316,1316,1315,1315,1314,1314,1313,1313,1312,1312,1311,1311,1310,1310,1309,1309,1307,1307,1306,1306,1305,1305,1304,1304,1303,1303,1302,1302,1301,1301,1300,1300,1299,1299,1298,1298,1297,1297,1296,1296,1295,1295,1294,1294,1293,1293,1292,1292,1290,1290,1289,1289,1288,1288,1287,1287,1286,1286,1285,1285,1284,1284,1283,1283,1282,1282,1281,1281,1280,1280,1279,1279,1277,1277,1275,1275,1274,1274,1273,1273,1272,1272,1271,1271,1270,1270,1269,1269,1268,1268,1266,1266,1265,1265,1264,1264,1263,1263,1262,1262,1261,1261,1260,1260,1259,1259,1258,1258,1257,1257,1256,1256,1255,1255,1254,1254,1253,1253,1252,1252,1251,1251,1250,1250,1249,1249,1248,1248,1247,1247,1246,1246,1245,1245,1244,1244,1243,1243,1242,1242,1241,1241,1240,1240,1239,1239,1237,1237,1236,1236,1235,1235,1234,1234,1233,1233,1232,1232,1231,1231,1230,1230,1229,1229,1228,1228,1226,1226,1225,1225,1223,1223,1222,1222,1221,1221,1219,1219,1218,1218,1217,1217,1216,1216,1215,1215,1213,1213,1212,1212,1211,1211,1210,1210,1209,1209,1208,1208,1207,1207,1206,1206,1205,1205,1204,1204,1203,1203,1202,1202,1201,1201,1200,1200,1199,1199,1198,1198,1197,1197,1196,1196,1195,1195,1194,1194,1193,1193,1192,1192,1191,1191,1190,1190,1189,1189,1188,1188,1187,1187,1186,1186,1185,1185,1184,1184,1183,1183,1182,1182,1181,1181,1180,1180,1179,1179,1178,1178,1177,1177,1176,1176,1175,1175,1174,1174,1173,1173,1172,1172,1171,1171,1170,1170,1169,1169,1168,1168,1166,1166,1165,1165,1164,1164,1163,1163,1162,1162,1161,1161,1160,1160,1159,1159,1158,1158,1157,1157,1156,1156,1155,1155,1154,1154,1153,1153,1151,1151,1150,1150,1149,1149,1148,1148,1147,1147,1146,1146,1144,1144,1143,1143,1142,1142,1141,1141,1140,1140,1137,1137,1136,1136,1135,1135,1134,1134,1133,1133,1132,1132,1131,1131,1130,1130,1129,1129,1128,1128,1127,1127,1126,1126,1125,1125,1124,1124,1123,1123,1122,1122,1121,1121,1120,1120,1119,1119,1118,1118,1117,1117,1116,1116,1115,1115,1114,1114,1113,1113,1112,1112,1111,1111,1110,1110,1109,1109,1108,1108,1107,1107,1106,1106,1105,1105,1104,1104,1103,1103,1102,1102,1101,1101,1100,1100,1099,1099,1098,1098,1097,1097,1096,1096,1095,1095,1094,1094,1093,1093,1092,1092,1090,1090,1089,1089,1088,1088,1087,1087,1086,1086,1085,1085,1084,1084,1083,1083,1082,1082,1081,1081,1080,1080,1079,1079,1078,1078,1077,1077,1076,1076,1075,1075,1074,1074,1073,1073,1072,1072,1071,1071,1070,1070,1069,1069,1068,1068,1067,1067,1066,1066,1065,1065,1064,1064,1063,1063,1061,1061,1060,1060,1059,1059,1058,1058,1057,1057,1055,1055,1054,1054,1053,1053,1051,1051,1050,1050,1049,1049,1048,1048,1047,1047,1046,1046,1045,1045,1044,1044,1042,1042,1041,1041,1040,1040,1039,1039,1038,1038,1037,1037,1036,1036,1035,1035,1034,1034,1033,1033,1032,1032,1030,1030,1029,1029,1028,1028,1027,1027,1026,1026,1025,1025,1023,1023,1021,1021,1020,1020,1019,1019,1017,1017,1015,1015,1014,1014,1013,1013,1012,1012,1011,1011,1010,1010,1009,1009,1008,1008,1007,1007,1006,1006,1005,1005,1004,1004,1003,1003,1002,1002,1001,1001,1000,1000,999,999,998,998,997,997,996,996,995,995,994,994,993,993,992,992,991,991,990,990,989,989,988,988,986,986,985,985,984,984,983,983,982,982,981,981,980,980,979,979,978,978,977,977,976,976,975,975,974,974,972,972,971,971,970,970,969,969,968,968,966,966,965,965,964,964,963,963,962,962,960,960,959,959,956,956,955,955,954,954,953,953,952,952,951,951,950,950,949,949,948,948,947,947,946,946,945,945,944,944,943,943,942,942,941,941,940,940,939,939,938,938,937,937,936,936,935,935,934,934,933,933,932,932,931,931,930,930,929,929,928,928,927,927,926,926,925,925,924,924,923,923,922,922,921,921,920,920,919,919,918,918,917,917,916,916,915,915,914,914,913,913,912,912,911,911,909,909,908,908,907,907,906,906,905,905,904,904,903,903,902,902,901,901,900,900,899,899,898,898,897,897,896,896,895,895,894,894,893,893,892,892,891,891,890,890,889,889,888,888,887,887,886,886,885,885,884,884,883,883,881,881,880,880,879,879,878,878,877,877,876,876,875,875,874,874,873,873,872,872,871,871,870,870,869,869,868,868,867,867,866,866,865,865,864,864,863,863,862,862,860,860,859,859,858,858,857,857,856,856,855,855,854,854,853,853,852,852,851,851,850,850,849,849,848,848,847,847,846,846,845,845,844,844,843,843,842,842,840,840,839,839,838,838,837,837,836,836,835,835,834,834,833,833,832,832,831,831,830,830,829,829,828,828,827,827,826,826,825,825,824,824,823,823,822,822,821,821,820,820,819,819,818,818,817,817,815,815,814,814,813,813,811,811,810,810,809,809,807,807,806,806,805,805,804,804,803,803,802,802,801,801,800,800,799,799,798,798,797,797,795,795,794,794,792,792,791,791,790,790,789,789,788,788,787,787,785,785,784,784,783,783,782,782,781,781,780,780,779,779,778,778,777,777,776,776,775,775,774,774,773,773,772,772,771,771,770,770,769,769,768,768,767,767,766,766,765,765,764,764,763,763,762,762,761,761,760,760,759,759,757,757,756,756,755,755,753,753,752,752,750,750,749,749,748,748,747,747,746,746,745,745,743,743,741,741,740,740,739,739,738,738,737,737,736,736,735,735,734,734,733,733,732,732,731,731,730,730,728,728,727,727,726,726,725,725,724,724,723,723,722,722,721,721,720,720,719,719,718,718,716,716,715,715,714,714,713,713,712,712,711,711,709,709,708,708,706,706,705,705,704,704,703,703,702,702,701,701,700,700,699,699,698,698,697,697,696,696,695,695,694,694,693,693,692,692,691,691,690,690,689,689,688,688,687,687,686,686,685,685,684,684,683,683,682,682,681,681,680,680,679,679,678,678,677,677,676,676,675,675,674,674,672,672,671,671,670,670,669,669,668,668,667,667,666,666,665,665,664,664,663,663,662,662,660,660,659,659,658,658,657,657,656,656,655,655,654,654,653,653,652,652,651,651,650,650,649,649,648,648,647,647,646,646,645,645,644,644,643,643,642,642,641,641,640,640,639,639,638,638,637,637,636,636,635,635,634,634,633,633,632,632,631,631,630,630,629,629,628,628,627,627,626,626,625,625,624,624,623,623,622,622,621,621,620,620,619,619,618,618,617,617,615,615,614,614,613,613,612,612,611,611,610,610,608,608,607,607,605,605,604,604,603,603,602,602,601,601,600,600,599,599,598,598,597,597,596,596,595,595,594,594,593,593,592,592,591,591,590,590,589,589,588,588,587,587,586,586,585,585,584,584,583,583,582,582,581,581,580,580,579,579,578,578,577,577,576,576,575,575,574,574,573,573,572,572,571,571,570,570,569,569,568,568,567,567,566,566,565,565,564,564,563,563,562,562,560,560,559,559,558,558,557,557,556,556,555,555,554,554,553,553,552,552,551,551,550,550,549,549,548,548,547,547,546,546,545,545,544,544,543,543,542,542,541,541,540,540,539,539,538,538,537,537,536,536,535,535,534,534,533,533,531,531,529,529,528,528,527,527,526,526,524,524,523,523,520,520,519,519,518,518,517,517,515,515,514,514,513,513,512,512,511,511,510,510,509,509,508,508,506,506,505,505,504,504,502,502,501,501,500,500,499,499,498,498,496,496,495,495,494,494,493,493,492,492,491,491,489,489,488,488,487,487,485,485,484,484,483,483,482,482,481,481,476,476,474,474,473,473,472,472,469,469,468,468,467,467,466,466,464,464,462,462,461,461,460,460,458,458,456,456,455,455,454,454,452,452,449,449,448,448,446,446,442,442,440,440,438,438,435,435,433,433,432,432,430,430,428,428,426,426,424,424,423,423,422,422,420,420,419,419,417,417,408,408,406,406,0.0],[32673,32673,32673,29245,29245,27353,27353,25659,25659,25633,25633,23651,23651,23598,23598,23362,23362,23202,23202,22916,22916,22777,22777,22370,22370,22072,22072,21832,21832,21820,21820,21755,21755,21496,21496,21372,21372,21345,21345,21262,21262,20680,20680,20636,20636,20186,20186,20105,20105,20103,20103,19831,19831,19818,19818,19560,19560,18729,18729,18662,18662,18242,18242,18134,18134,17729,17729,17644,17644,17481,17481,17471,17471,17422,17422,17061,17061,16944,16944,16788,16788,16671,16671,16652,16652,16519,16519,16476,16476,16459,16459,16397,16397,16228,16228,16192,16192,16148,16148,16140,16140,16063,16063,16035,16035,15975,15975,15900,15900,15854,15854,15784,15784,15517,15517,15502,15502,15456,15456,15453,15453,15207,15207,15183,15183,15174,15174,15125,15125,15024,15024,14858,14858,14736,14736,14727,14727,14715,14715,14638,14638,14613,14613,14552,14552,14458,14458,14409,14409,14325,14325,14321,14321,14291,14291,14289,14289,14218,14218,14199,14199,14178,14178,14176,14176,14072,14072,14070,14070,13994,13994,13978,13978,13941,13941,13938,13938,13906,13906,13891,13891,13867,13867,13837,13837,13833,13833,13817,13817,13757,13757,13717,13717,13645,13645,13644,13644,13563,13563,13542,13542,13497,13497,13400,13400,13377,13377,13347,13347,13319,13319,13203,13203,13177,13177,13073,13073,13063,13063,12971,12971,12961,12961,12958,12958,12930,12930,12923,12923,12899,12899,12861,12861,12788,12788,12787,12787,12786,12786,12775,12775,12770,12770,12758,12758,12750,12750,12742,12742,12689,12689,12654,12654,12642,12642,12612,12612,12608,12608,12452,12452,12446,12446,12444,12444,12441,12441,12435,12435,12343,12343,12295,12295,12227,12227,12154,12154,12127,12127,12088,12088,12073,12073,12053,12053,12008,12008,12001,12001,11984,11984,11966,11966,11962,11962,11943,11943,11931,11931,11896,11896,11886,11886,11862,11862,11841,11841,11815,11815,11752,11752,11728,11728,11687,11687,11685,11685,11633,11633,11610,11610,11592,11592,11549,11549,11528,11528,11517,11517,11413,11413,11399,11399,11373,11373,11370,11370,11367,11367,11358,11358,11320,11320,11311,11311,11296,11296,11234,11234,11221,11221,11205,11205,11179,11179,11155,11155,11154,11154,11106,11106,11104,11104,11094,11094,11085,11085,11014,11014,11006,11006,10999,10999,10987,10987,10984,10984,10907,10907,10886,10886,10880,10880,10861,10861,10856,10856,10854,10854,10853,10853,10847,10847,10843,10843,10821,10821,10810,10810,10807,10807,10791,10791,10767,10767,10755,10755,10743,10743,10673,10673,10666,10666,10656,10656,10655,10655,10589,10589,10579,10579,10573,10573,10516,10516,10505,10505,10479,10479,10472,10472,10465,10465,10464,10464,10459,10459,10447,10447,10405,10405,10397,10397,10396,10396,10384,10384,10377,10377,10376,10376,10373,10373,10365,10365,10322,10322,10315,10315,10312,10312,10308,10308,10289,10289,10265,10265,10262,10262,10252,10252,10234,10234,10223,10223,10214,10214,10176,10176,10161,10161,10136,10136,10083,10083,10070,10070,10044,10044,10007,10007,9992,9992,9984,9984,9955,9955,9936,9936,9880,9880,9878,9878,9863,9863,9852,9852,9846,9846,9845,9845,9840,9840,9838,9838,9803,9803,9801,9801,9800,9800,9771,9771,9766,9766,9759,9759,9754,9754,9739,9739,9707,9707,9695,9695,9683,9683,9656,9656,9644,9644,9625,9625,9622,9622,9575,9575,9561,9561,9550,9550,9531,9531,9520,9520,9517,9517,9495,9495,9469,9469,9463,9463,9434,9434,9422,9422,9401,9401,9396,9396,9391,9391,9384,9384,9378,9378,9344,9344,9326,9326,9307,9307,9291,9291,9270,9270,9239,9239,9216,9216,9214,9214,9213,9213,9210,9210,9204,9204,9185,9185,9182,9182,9180,9180,9155,9155,9152,9152,9142,9142,9117,9117,9109,9109,9099,9099,9083,9083,9056,9056,9053,9053,9046,9046,9034,9034,9030,9030,8974,8974,8959,8959,8956,8956,8947,8947,8936,8936,8915,8915,8893,8893,8887,8887,8883,8883,8868,8868,8863,8863,8843,8843,8832,8832,8823,8823,8819,8819,8808,8808,8792,8792,8783,8783,8763,8763,8746,8746,8726,8726,8719,8719,8692,8692,8691,8691,8686,8686,8676,8676,8672,8672,8669,8669,8661,8661,8659,8659,8655,8655,8644,8644,8625,8625,8624,8624,8602,8602,8581,8581,8562,8562,8548,8548,8536,8536,8526,8526,8525,8525,8510,8510,8498,8498,8477,8477,8457,8457,8453,8453,8452,8452,8449,8449,8432,8432,8413,8413,8411,8411,8410,8410,8406,8406,8398,8398,8387,8387,8376,8376,8373,8373,8353,8353,8347,8347,8326,8326,8318,8318,8314,8314,8305,8305,8302,8302,8297,8297,8289,8289,8280,8280,8241,8241,8238,8238,8231,8231,8220,8220,8196,8196,8188,8188,8176,8176,8174,8174,8147,8147,8145,8145,8137,8137,8129,8129,8125,8125,8120,8120,8117,8117,8111,8111,8104,8104,8093,8093,8078,8078,8077,8077,8069,8069,8040,8040,8024,8024,8017,8017,8012,8012,8011,8011,8004,8004,7991,7991,7976,7976,7972,7972,7970,7970,7964,7964,7949,7949,7946,7946,7944,7944,7935,7935,7933,7933,7932,7932,7930,7930,7928,7928,7925,7925,7923,7923,7906,7906,7904,7904,7898,7898,7887,7887,7886,7886,7884,7884,7883,7883,7865,7865,7859,7859,7847,7847,7840,7840,7835,7835,7831,7831,7818,7818,7804,7804,7797,7797,7785,7785,7775,7775,7772,7772,7765,7765,7762,7762,7755,7755,7750,7750,7726,7726,7725,7725,7710,7710,7698,7698,7695,7695,7694,7694,7693,7693,7689,7689,7686,7686,7685,7685,7683,7683,7673,7673,7671,7671,7665,7665,7654,7654,7652,7652,7644,7644,7624,7624,7621,7621,7619,7619,7618,7618,7613,7613,7602,7602,7599,7599,7595,7595,7589,7589,7578,7578,7576,7576,7564,7564,7563,7563,7557,7557,7554,7554,7553,7553,7548,7548,7540,7540,7527,7527,7521,7521,7515,7515,7510,7510,7505,7505,7500,7500,7499,7499,7485,7485,7484,7484,7483,7483,7465,7465,7459,7459,7435,7435,7434,7434,7429,7429,7414,7414,7406,7406,7399,7399,7391,7391,7385,7385,7380,7380,7374,7374,7345,7345,7343,7343,7332,7332,7331,7331,7308,7308,7303,7303,7302,7302,7299,7299,7291,7291,7287,7287,7268,7268,7265,7265,7263,7263,7251,7251,7249,7249,7241,7241,7231,7231,7225,7225,7214,7214,7208,7208,7207,7207,7205,7205,7200,7200,7195,7195,7194,7194,7192,7192,7191,7191,7180,7180,7166,7166,7164,7164,7156,7156,7150,7150,7148,7148,7142,7142,7134,7134,7131,7131,7109,7109,7106,7106,7101,7101,7088,7088,7083,7083,7082,7082,7077,7077,7075,7075,7071,7071,7063,7063,7049,7049,7048,7048,7042,7042,7033,7033,7030,7030,7026,7026,7022,7022,7013,7013,7008,7008,7006,7006,7000,7000,6994,6994,6991,6991,6990,6990,6988,6988,6985,6985,6977,6977,6976,6976,6975,6975,6973,6973,6966,6966,6956,6956,6952,6952,6949,6949,6931,6931,6927,6927,6922,6922,6896,6896,6893,6893,6851,6851,6850,6850,6844,6844,6832,6832,6816,6816,6813,6813,6806,6806,6805,6805,6804,6804,6796,6796,6795,6795,6794,6794,6783,6783,6779,6779,6762,6762,6755,6755,6753,6753,6747,6747,6742,6742,6740,6740,6738,6738,6735,6735,6724,6724,6723,6723,6718,6718,6716,6716,6711,6711,6703,6703,6700,6700,6699,6699,6697,6697,6690,6690,6684,6684,6682,6682,6681,6681,6680,6680,6677,6677,6676,6676,6674,6674,6668,6668,6663,6663,6657,6657,6656,6656,6646,6646,6622,6622,6609,6609,6591,6591,6589,6589,6583,6583,6580,6580,6574,6574,6567,6567,6565,6565,6564,6564,6563,6563,6553,6553,6550,6550,6549,6549,6547,6547,6534,6534,6521,6521,6515,6515,6514,6514,6513,6513,6508,6508,6502,6502,6496,6496,6493,6493,6492,6492,6490,6490,6486,6486,6485,6485,6475,6475,6469,6469,6460,6460,6456,6456,6451,6451,6443,6443,6441,6441,6436,6436,6435,6435,6434,6434,6428,6428,6424,6424,6420,6420,6418,6418,6416,6416,6407,6407,6400,6400,6392,6392,6386,6386,6384,6384,6381,6381,6380,6380,6377,6377,6373,6373,6362,6362,6348,6348,6346,6346,6337,6337,6335,6335,6333,6333,6332,6332,6322,6322,6320,6320,6317,6317,6314,6314,6309,6309,6299,6299,6295,6295,6294,6294,6292,6292,6288,6288,6279,6279,6275,6275,6274,6274,6271,6271,6268,6268,6267,6267,6266,6266,6262,6262,6254,6254,6248,6248,6245,6245,6244,6244,6243,6243,6227,6227,6223,6223,6222,6222,6219,6219,6218,6218,6210,6210,6207,6207,6206,6206,6204,6204,6199,6199,6191,6191,6184,6184,6182,6182,6181,6181,6177,6177,6176,6176,6171,6171,6165,6165,6160,6160,6150,6150,6149,6149,6147,6147,6144,6144,6141,6141,6137,6137,6132,6132,6124,6124,6121,6121,6116,6116,6112,6112,6111,6111,6106,6106,6104,6104,6102,6102,6100,6100,6099,6099,6098,6098,6093,6093,6089,6089,6084,6084,6083,6083,6073,6073,6064,6064,6058,6058,6057,6057,6056,6056,6051,6051,6045,6045,6040,6040,6039,6039,6037,6037,6035,6035,6028,6028,6026,6026,6024,6024,6023,6023,6022,6022,6018,6018,6011,6011,6009,6009,6002,6002,5992,5992,5990,5990,5982,5982,5978,5978,5976,5976,5975,5975,5971,5971,5969,5969,5967,5967,5964,5964,5963,5963,5955,5955,5951,5951,5947,5947,5944,5944,5943,5943,5941,5941,5938,5938,5937,5937,5936,5936,5935,5935,5932,5932,5930,5930,5928,5928,5925,5925,5916,5916,5913,5913,5911,5911,5900,5900,5897,5897,5889,5889,5887,5887,5884,5884,5882,5882,5880,5880,5865,5865,5863,5863,5853,5853,5852,5852,5851,5851,5848,5848,5844,5844,5843,5843,5842,5842,5841,5841,5838,5838,5834,5834,5825,5825,5821,5821,5820,5820,5816,5816,5815,5815,5811,5811,5810,5810,5799,5799,5797,5797,5794,5794,5793,5793,5792,5792,5789,5789,5784,5784,5780,5780,5768,5768,5763,5763,5761,5761,5757,5757,5751,5751,5748,5748,5745,5745,5736,5736,5734,5734,5732,5732,5731,5731,5730,5730,5725,5725,5724,5724,5718,5718,5717,5717,5714,5714,5713,5713,5709,5709,5699,5699,5698,5698,5695,5695,5684,5684,5681,5681,5678,5678,5677,5677,5669,5669,5668,5668,5667,5667,5665,5665,5663,5663,5657,5657,5654,5654,5653,5653,5651,5651,5648,5648,5642,5642,5641,5641,5634,5634,5631,5631,5626,5626,5623,5623,5621,5621,5619,5619,5617,5617,5612,5612,5608,5608,5606,5606,5602,5602,5597,5597,5588,5588,5586,5586,5584,5584,5582,5582,5579,5579,5572,5572,5571,5571,5565,5565,5564,5564,5562,5562,5559,5559,5557,5557,5554,5554,5551,5551,5548,5548,5546,5546,5541,5541,5540,5540,5539,5539,5536,5536,5534,5534,5533,5533,5525,5525,5517,5517,5515,5515,5513,5513,5512,5512,5507,5507,5497,5497,5493,5493,5490,5490,5489,5489,5488,5488,5482,5482,5475,5475,5470,5470,5469,5469,5465,5465,5461,5461,5457,5457,5454,5454,5452,5452,5450,5450,5447,5447,5445,5445,5441,5441,5440,5440,5435,5435,5430,5430,5426,5426,5425,5425,5424,5424,5417,5417,5414,5414,5410,5410,5409,5409,5404,5404,5402,5402,5399,5399,5398,5398,5394,5394,5392,5392,5390,5390,5387,5387,5385,5385,5383,5383,5377,5377,5375,5375,5369,5369,5356,5356,5352,5352,5351,5351,5349,5349,5345,5345,5342,5342,5341,5341,5338,5338,5335,5335,5332,5332,5328,5328,5327,5327,5320,5320,5319,5319,5318,5318,5316,5316,5303,5303,5302,5302,5301,5301,5297,5297,5294,5294,5290,5290,5284,5284,5283,5283,5280,5280,5278,5278,5276,5276,5274,5274,5273,5273,5267,5267,5266,5266,5265,5265,5261,5261,5260,5260,5258,5258,5256,5256,5255,5255,5254,5254,5250,5250,5247,5247,5246,5246,5245,5245,5242,5242,5241,5241,5239,5239,5236,5236,5232,5232,5229,5229,5226,5226,5224,5224,5223,5223,5221,5221,5216,5216,5213,5213,5210,5210,5208,5208,5205,5205,5203,5203,5202,5202,5201,5201,5200,5200,5196,5196,5195,5195,5193,5193,5186,5186,5184,5184,5182,5182,5180,5180,5175,5175,5172,5172,5171,5171,5159,5159,5155,5155,5152,5152,5151,5151,5149,5149,5148,5148,5141,5141,5137,5137,5136,5136,5128,5128,5126,5126,5125,5125,5122,5122,5120,5120,5111,5111,5107,5107,5106,5106,5102,5102,5100,5100,5096,5096,5093,5093,5092,5092,5091,5091,5089,5089,5087,5087,5086,5086,5083,5083,5081,5081,5078,5078,5076,5076,5072,5072,5070,5070,5068,5068,5067,5067,5066,5066,5065,5065,5063,5063,5058,5058,5057,5057,5055,5055,5054,5054,5053,5053,5049,5049,5046,5046,5041,5041,5040,5040,5039,5039,5036,5036,5032,5032,5028,5028,5023,5023,5019,5019,5017,5017,5014,5014,5012,5012,5011,5011,5008,5008,5007,5007,5004,5004,5003,5003,5002,5002,5000,5000,4999,4999,4988,4988,4987,4987,4982,4982,4980,4980,4977,4977,4975,4975,4974,4974,4973,4973,4970,4970,4963,4963,4962,4962,4959,4959,4958,4958,4956,4956,4954,4954,4945,4945,4944,4944,4942,4942,4937,4937,4935,4935,4934,4934,4930,4930,4929,4929,4926,4926,4924,4924,4923,4923,4921,4921,4920,4920,4918,4918,4916,4916,4915,4915,4910,4910,4909,4909,4906,4906,4905,4905,4904,4904,4901,4901,4894,4894,4893,4893,4890,4890,4889,4889,4888,4888,4887,4887,4885,4885,4884,4884,4882,4882,4879,4879,4876,4876,4873,4873,4871,4871,4867,4867,4864,4864,4860,4860,4858,4858,4855,4855,4853,4853,4849,4849,4843,4843,4842,4842,4841,4841,4839,4839,4837,4837,4829,4829,4828,4828,4825,4825,4817,4817,4815,4815,4813,4813,4811,4811,4810,4810,4808,4808,4807,4807,4804,4804,4803,4803,4799,4799,4791,4791,4790,4790,4789,4789,4787,4787,4786,4786,4780,4780,4777,4777,4775,4775,4774,4774,4772,4772,4771,4771,4770,4770,4769,4769,4768,4768,4766,4766,4763,4763,4758,4758,4756,4756,4755,4755,4750,4750,4748,4748,4746,4746,4745,4745,4743,4743,4741,4741,4737,4737,4729,4729,4722,4722,4719,4719,4718,4718,4715,4715,4714,4714,4702,4702,4701,4701,4698,4698,4695,4695,4693,4693,4689,4689,4688,4688,4687,4687,4685,4685,4683,4683,4682,4682,4680,4680,4679,4679,4676,4676,4673,4673,4667,4667,4666,4666,4664,4664,4663,4663,4655,4655,4654,4654,4653,4653,4651,4651,4648,4648,4646,4646,4643,4643,4640,4640,4638,4638,4635,4635,4634,4634,4633,4633,4632,4632,4629,4629,4628,4628,4627,4627,4621,4621,4619,4619,4618,4618,4617,4617,4614,4614,4613,4613,4612,4612,4610,4610,4607,4607,4606,4606,4604,4604,4601,4601,4599,4599,4594,4594,4592,4592,4587,4587,4584,4584,4583,4583,4581,4581,4577,4577,4575,4575,4574,4574,4570,4570,4569,4569,4568,4568,4566,4566,4565,4565,4563,4563,4560,4560,4559,4559,4557,4557,4553,4553,4551,4551,4548,4548,4544,4544,4543,4543,4539,4539,4538,4538,4534,4534,4533,4533,4532,4532,4530,4530,4529,4529,4527,4527,4525,4525,4522,4522,4521,4521,4519,4519,4517,4517,4516,4516,4514,4514,4512,4512,4511,4511,4510,4510,4509,4509,4508,4508,4506,4506,4503,4503,4502,4502,4501,4501,4497,4497,4495,4495,4494,4494,4491,4491,4490,4490,4486,4486,4485,4485,4482,4482,4481,4481,4479,4479,4474,4474,4473,4473,4467,4467,4466,4466,4462,4462,4460,4460,4458,4458,4456,4456,4453,4453,4449,4449,4446,4446,4442,4442,4440,4440,4437,4437,4434,4434,4430,4430,4429,4429,4428,4428,4425,4425,4424,4424,4419,4419,4418,4418,4415,4415,4412,4412,4411,4411,4409,4409,4405,4405,4404,4404,4398,4398,4392,4392,4388,4388,4387,4387,4385,4385,4382,4382,4381,4381,4380,4380,4379,4379,4375,4375,4374,4374,4373,4373,4370,4370,4368,4368,4364,4364,4363,4363,4361,4361,4360,4360,4358,4358,4355,4355,4354,4354,4352,4352,4350,4350,4346,4346,4345,4345,4344,4344,4343,4343,4342,4342,4341,4341,4338,4338,4337,4337,4336,4336,4333,4333,4331,4331,4330,4330,4329,4329,4328,4328,4327,4327,4320,4320,4318,4318,4314,4314,4309,4309,4308,4308,4306,4306,4305,4305,4302,4302,4301,4301,4298,4298,4297,4297,4288,4288,4287,4287,4286,4286,4285,4285,4284,4284,4280,4280,4279,4279,4278,4278,4277,4277,4276,4276,4275,4275,4274,4274,4271,4271,4270,4270,4268,4268,4266,4266,4262,4262,4260,4260,4259,4259,4257,4257,4256,4256,4255,4255,4254,4254,4253,4253,4250,4250,4249,4249,4248,4248,4245,4245,4244,4244,4243,4243,4241,4241,4239,4239,4238,4238,4237,4237,4232,4232,4231,4231,4230,4230,4229,4229,4227,4227,4222,4222,4221,4221,4217,4217,4213,4213,4211,4211,4209,4209,4207,4207,4204,4204,4203,4203,4202,4202,4198,4198,4192,4192,4191,4191,4189,4189,4188,4188,4186,4186,4181,4181,4179,4179,4178,4178,4177,4177,4176,4176,4173,4173,4172,4172,4171,4171,4166,4166,4164,4164,4163,4163,4160,4160,4157,4157,4156,4156,4155,4155,4154,4154,4152,4152,4150,4150,4149,4149,4144,4144,4142,4142,4140,4140,4138,4138,4137,4137,4135,4135,4134,4134,4131,4131,4130,4130,4129,4129,4128,4128,4126,4126,4125,4125,4121,4121,4119,4119,4116,4116,4115,4115,4114,4114,4112,4112,4111,4111,4110,4110,4109,4109,4108,4108,4107,4107,4106,4106,4103,4103,4102,4102,4101,4101,4100,4100,4098,4098,4097,4097,4096,4096,4095,4095,4094,4094,4093,4093,4092,4092,4089,4089,4087,4087,4085,4085,4084,4084,4083,4083,4082,4082,4080,4080,4079,4079,4077,4077,4076,4076,4074,4074,4073,4073,4071,4071,4070,4070,4066,4066,4065,4065,4064,4064,4061,4061,4060,4060,4059,4059,4056,4056,4055,4055,4053,4053,4052,4052,4051,4051,4050,4050,4047,4047,4046,4046,4044,4044,4040,4040,4039,4039,4035,4035,4034,4034,4033,4033,4032,4032,4030,4030,4027,4027,4025,4025,4023,4023,4021,4021,4020,4020,4018,4018,4016,4016,4015,4015,4014,4014,4013,4013,4011,4011,4010,4010,4009,4009,4008,4008,4004,4004,4002,4002,4000,4000,3999,3999,3997,3997,3996,3996,3994,3994,3993,3993,3992,3992,3988,3988,3984,3984,3981,3981,3978,3978,3977,3977,3974,3974,3972,3972,3971,3971,3970,3970,3969,3969,3968,3968,3967,3967,3966,3966,3965,3965,3964,3964,3959,3959,3958,3958,3955,3955,3953,3953,3951,3951,3950,3950,3947,3947,3946,3946,3943,3943,3941,3941,3940,3940,3938,3938,3936,3936,3935,3935,3934,3934,3933,3933,3932,3932,3931,3931,3930,3930,3929,3929,3928,3928,3926,3926,3924,3924,3922,3922,3921,3921,3920,3920,3919,3919,3913,3913,3912,3912,3911,3911,3910,3910,3907,3907,3906,3906,3905,3905,3904,3904,3903,3903,3901,3901,3899,3899,3898,3898,3896,3896,3895,3895,3892,3892,3891,3891,3889,3889,3887,3887,3886,3886,3885,3885,3882,3882,3881,3881,3879,3879,3878,3878,3877,3877,3874,3874,3873,3873,3867,3867,3866,3866,3865,3865,3863,3863,3861,3861,3860,3860,3859,3859,3856,3856,3855,3855,3854,3854,3852,3852,3850,3850,3847,3847,3845,3845,3843,3843,3842,3842,3840,3840,3839,3839,3838,3838,3837,3837,3836,3836,3834,3834,3831,3831,3830,3830,3827,3827,3826,3826,3825,3825,3824,3824,3823,3823,3821,3821,3818,3818,3816,3816,3813,3813,3811,3811,3810,3810,3806,3806,3805,3805,3804,3804,3803,3803,3801,3801,3799,3799,3797,3797,3796,3796,3795,3795,3794,3794,3790,3790,3789,3789,3787,3787,3786,3786,3784,3784,3780,3780,3779,3779,3777,3777,3774,3774,3773,3773,3772,3772,3771,3771,3770,3770,3768,3768,3765,3765,3764,3764,3763,3763,3762,3762,3759,3759,3758,3758,3756,3756,3755,3755,3753,3753,3752,3752,3751,3751,3749,3749,3745,3745,3744,3744,3743,3743,3741,3741,3740,3740,3739,3739,3736,3736,3735,3735,3732,3732,3730,3730,3729,3729,3726,3726,3725,3725,3724,3724,3723,3723,3722,3722,3720,3720,3718,3718,3717,3717,3714,3714,3713,3713,3712,3712,3711,3711,3710,3710,3708,3708,3707,3707,3706,3706,3705,3705,3704,3704,3703,3703,3702,3702,3701,3701,3700,3700,3699,3699,3698,3698,3696,3696,3694,3694,3693,3693,3692,3692,3691,3691,3690,3690,3689,3689,3688,3688,3684,3684,3683,3683,3681,3681,3680,3680,3675,3675,3674,3674,3672,3672,3669,3669,3667,3667,3666,3666,3665,3665,3663,3663,3662,3662,3661,3661,3658,3658,3657,3657,3655,3655,3653,3653,3652,3652,3650,3650,3648,3648,3647,3647,3644,3644,3643,3643,3642,3642,3641,3641,3640,3640,3639,3639,3637,3637,3636,3636,3635,3635,3634,3634,3633,3633,3632,3632,3631,3631,3629,3629,3628,3628,3625,3625,3624,3624,3623,3623,3622,3622,3620,3620,3619,3619,3618,3618,3614,3614,3611,3611,3609,3609,3607,3607,3606,3606,3604,3604,3603,3603,3601,3601,3600,3600,3599,3599,3597,3597,3596,3596,3595,3595,3594,3594,3593,3593,3592,3592,3590,3590,3589,3589,3588,3588,3587,3587,3586,3586,3585,3585,3583,3583,3582,3582,3581,3581,3578,3578,3576,3576,3574,3574,3572,3572,3571,3571,3570,3570,3568,3568,3567,3567,3566,3566,3564,3564,3562,3562,3561,3561,3559,3559,3557,3557,3556,3556,3555,3555,3554,3554,3553,3553,3552,3552,3551,3551,3550,3550,3548,3548,3547,3547,3546,3546,3544,3544,3543,3543,3542,3542,3541,3541,3540,3540,3538,3538,3536,3536,3535,3535,3530,3530,3529,3529,3528,3528,3527,3527,3526,3526,3525,3525,3524,3524,3521,3521,3518,3518,3517,3517,3516,3516,3515,3515,3514,3514,3509,3509,3508,3508,3507,3507,3506,3506,3505,3505,3504,3504,3503,3503,3502,3502,3501,3501,3500,3500,3498,3498,3497,3497,3496,3496,3494,3494,3493,3493,3492,3492,3490,3490,3489,3489,3488,3488,3485,3485,3484,3484,3481,3481,3477,3477,3476,3476,3473,3473,3472,3472,3471,3471,3470,3470,3469,3469,3468,3468,3467,3467,3466,3466,3465,3465,3464,3464,3463,3463,3461,3461,3459,3459,3458,3458,3456,3456,3455,3455,3454,3454,3453,3453,3452,3452,3450,3450,3449,3449,3448,3448,3447,3447,3445,3445,3442,3442,3440,3440,3439,3439,3438,3438,3437,3437,3436,3436,3435,3435,3433,3433,3432,3432,3430,3430,3429,3429,3428,3428,3426,3426,3424,3424,3423,3423,3420,3420,3419,3419,3418,3418,3416,3416,3415,3415,3414,3414,3413,3413,3411,3411,3410,3410,3408,3408,3407,3407,3406,3406,3405,3405,3403,3403,3402,3402,3401,3401,3399,3399,3397,3397,3396,3396,3395,3395,3394,3394,3393,3393,3392,3392,3391,3391,3390,3390,3389,3389,3387,3387,3386,3386,3385,3385,3384,3384,3381,3381,3377,3377,3376,3376,3375,3375,3374,3374,3373,3373,3371,3371,3370,3370,3369,3369,3368,3368,3366,3366,3365,3365,3364,3364,3363,3363,3362,3362,3361,3361,3360,3360,3359,3359,3357,3357,3356,3356,3355,3355,3354,3354,3353,3353,3352,3352,3351,3351,3349,3349,3348,3348,3346,3346,3345,3345,3341,3341,3340,3340,3339,3339,3338,3338,3337,3337,3334,3334,3333,3333,3332,3332,3331,3331,3330,3330,3329,3329,3327,3327,3326,3326,3325,3325,3324,3324,3323,3323,3322,3322,3321,3321,3319,3319,3318,3318,3317,3317,3316,3316,3315,3315,3314,3314,3313,3313,3312,3312,3311,3311,3310,3310,3309,3309,3308,3308,3306,3306,3305,3305,3304,3304,3303,3303,3302,3302,3301,3301,3300,3300,3297,3297,3296,3296,3292,3292,3291,3291,3290,3290,3288,3288,3285,3285,3284,3284,3283,3283,3282,3282,3281,3281,3280,3280,3278,3278,3277,3277,3276,3276,3275,3275,3273,3273,3272,3272,3271,3271,3270,3270,3266,3266,3265,3265,3263,3263,3262,3262,3261,3261,3260,3260,3259,3259,3258,3258,3257,3257,3255,3255,3254,3254,3253,3253,3252,3252,3251,3251,3250,3250,3249,3249,3247,3247,3246,3246,3245,3245,3244,3244,3242,3242,3241,3241,3240,3240,3239,3239,3238,3238,3237,3237,3236,3236,3235,3235,3234,3234,3231,3231,3230,3230,3229,3229,3228,3228,3227,3227,3226,3226,3225,3225,3224,3224,3223,3223,3221,3221,3220,3220,3218,3218,3217,3217,3215,3215,3212,3212,3211,3211,3210,3210,3208,3208,3207,3207,3206,3206,3205,3205,3203,3203,3201,3201,3200,3200,3199,3199,3198,3198,3197,3197,3195,3195,3194,3194,3193,3193,3191,3191,3190,3190,3188,3188,3187,3187,3186,3186,3183,3183,3182,3182,3181,3181,3179,3179,3178,3178,3177,3177,3176,3176,3175,3175,3173,3173,3172,3172,3171,3171,3170,3170,3169,3169,3168,3168,3167,3167,3166,3166,3164,3164,3163,3163,3162,3162,3160,3160,3159,3159,3158,3158,3157,3157,3156,3156,3155,3155,3153,3153,3152,3152,3151,3151,3150,3150,3149,3149,3148,3148,3146,3146,3145,3145,3144,3144,3143,3143,3142,3142,3141,3141,3140,3140,3139,3139,3137,3137,3135,3135,3134,3134,3132,3132,3131,3131,3130,3130,3129,3129,3128,3128,3127,3127,3126,3126,3125,3125,3124,3124,3123,3123,3122,3122,3121,3121,3120,3120,3119,3119,3117,3117,3116,3116,3115,3115,3114,3114,3113,3113,3112,3112,3111,3111,3110,3110,3109,3109,3108,3108,3107,3107,3106,3106,3105,3105,3104,3104,3103,3103,3102,3102,3101,3101,3100,3100,3098,3098,3097,3097,3095,3095,3094,3094,3093,3093,3092,3092,3091,3091,3090,3090,3089,3089,3088,3088,3087,3087,3086,3086,3085,3085,3084,3084,3083,3083,3082,3082,3081,3081,3080,3080,3079,3079,3078,3078,3077,3077,3076,3076,3075,3075,3074,3074,3071,3071,3070,3070,3069,3069,3068,3068,3067,3067,3066,3066,3065,3065,3064,3064,3063,3063,3062,3062,3061,3061,3060,3060,3058,3058,3057,3057,3056,3056,3055,3055,3054,3054,3052,3052,3051,3051,3050,3050,3049,3049,3048,3048,3047,3047,3046,3046,3045,3045,3044,3044,3043,3043,3041,3041,3040,3040,3039,3039,3038,3038,3037,3037,3036,3036,3035,3035,3034,3034,3033,3033,3032,3032,3031,3031,3030,3030,3029,3029,3027,3027,3026,3026,3025,3025,3024,3024,3023,3023,3022,3022,3021,3021,3020,3020,3019,3019,3018,3018,3017,3017,3016,3016,3015,3015,3014,3014,3013,3013,3012,3012,3011,3011,3010,3010,3009,3009,3008,3008,3006,3006,3005,3005,3004,3004,3003,3003,3002,3002,3001,3001,3000,3000,2999,2999,2998,2998,2997,2997,2996,2996,2994,2994,2993,2993,2992,2992,2991,2991,2990,2990,2989,2989,2988,2988,2987,2987,2986,2986,2985,2985,2984,2984,2983,2983,2982,2982,2981,2981,2980,2980,2979,2979,2978,2978,2977,2977,2976,2976,2975,2975,2974,2974,2973,2973,2972,2972,2971,2971,2970,2970,2969,2969,2968,2968,2967,2967,2965,2965,2964,2964,2963,2963,2957,2957,2954,2954,2953,2953,2952,2952,2951,2951,2949,2949,2948,2948,2947,2947,2945,2945,2943,2943,2941,2941,2940,2940,2939,2939,2938,2938,2937,2937,2936,2936,2935,2935,2934,2934,2933,2933,2932,2932,2931,2931,2930,2930,2929,2929,2927,2927,2926,2926,2925,2925,2924,2924,2923,2923,2922,2922,2921,2921,2920,2920,2919,2919,2918,2918,2917,2917,2914,2914,2913,2913,2911,2911,2910,2910,2909,2909,2908,2908,2907,2907,2906,2906,2905,2905,2904,2904,2903,2903,2902,2902,2901,2901,2900,2900,2899,2899,2898,2898,2897,2897,2896,2896,2895,2895,2894,2894,2893,2893,2892,2892,2891,2891,2889,2889,2888,2888,2887,2887,2886,2886,2885,2885,2883,2883,2882,2882,2881,2881,2880,2880,2879,2879,2877,2877,2876,2876,2875,2875,2874,2874,2873,2873,2872,2872,2871,2871,2870,2870,2869,2869,2868,2868,2867,2867,2866,2866,2865,2865,2864,2864,2863,2863,2862,2862,2861,2861,2860,2860,2859,2859,2858,2858,2857,2857,2856,2856,2855,2855,2854,2854,2853,2853,2851,2851,2850,2850,2849,2849,2848,2848,2847,2847,2846,2846,2845,2845,2844,2844,2843,2843,2842,2842,2841,2841,2840,2840,2839,2839,2838,2838,2837,2837,2836,2836,2835,2835,2834,2834,2833,2833,2832,2832,2831,2831,2830,2830,2829,2829,2828,2828,2827,2827,2826,2826,2825,2825,2822,2822,2821,2821,2820,2820,2818,2818,2817,2817,2816,2816,2815,2815,2814,2814,2813,2813,2812,2812,2811,2811,2810,2810,2808,2808,2807,2807,2806,2806,2805,2805,2804,2804,2803,2803,2802,2802,2800,2800,2799,2799,2797,2797,2796,2796,2795,2795,2794,2794,2793,2793,2792,2792,2791,2791,2790,2790,2789,2789,2787,2787,2786,2786,2785,2785,2784,2784,2783,2783,2782,2782,2781,2781,2780,2780,2779,2779,2778,2778,2777,2777,2776,2776,2775,2775,2774,2774,2772,2772,2771,2771,2770,2770,2769,2769,2768,2768,2767,2767,2766,2766,2765,2765,2764,2764,2763,2763,2762,2762,2761,2761,2760,2760,2759,2759,2758,2758,2757,2757,2756,2756,2755,2755,2751,2751,2750,2750,2749,2749,2748,2748,2747,2747,2746,2746,2745,2745,2744,2744,2743,2743,2742,2742,2741,2741,2740,2740,2739,2739,2738,2738,2737,2737,2736,2736,2735,2735,2734,2734,2733,2733,2732,2732,2731,2731,2730,2730,2729,2729,2728,2728,2727,2727,2725,2725,2724,2724,2723,2723,2722,2722,2721,2721,2720,2720,2719,2719,2718,2718,2717,2717,2716,2716,2715,2715,2714,2714,2713,2713,2712,2712,2711,2711,2710,2710,2709,2709,2708,2708,2707,2707,2705,2705,2704,2704,2703,2703,2702,2702,2701,2701,2700,2700,2699,2699,2698,2698,2697,2697,2694,2694,2693,2693,2691,2691,2690,2690,2689,2689,2688,2688,2687,2687,2686,2686,2685,2685,2684,2684,2683,2683,2682,2682,2681,2681,2680,2680,2679,2679,2677,2677,2676,2676,2675,2675,2674,2674,2673,2673,2672,2672,2671,2671,2670,2670,2668,2668,2667,2667,2666,2666,2665,2665,2664,2664,2663,2663,2662,2662,2661,2661,2660,2660,2659,2659,2658,2658,2657,2657,2656,2656,2655,2655,2654,2654,2653,2653,2652,2652,2651,2651,2650,2650,2649,2649,2648,2648,2647,2647,2646,2646,2645,2645,2643,2643,2642,2642,2641,2641,2640,2640,2639,2639,2638,2638,2637,2637,2635,2635,2634,2634,2633,2633,2632,2632,2631,2631,2630,2630,2629,2629,2628,2628,2627,2627,2626,2626,2625,2625,2624,2624,2623,2623,2622,2622,2621,2621,2620,2620,2619,2619,2618,2618,2617,2617,2615,2615,2614,2614,2613,2613,2612,2612,2611,2611,2610,2610,2608,2608,2607,2607,2606,2606,2605,2605,2604,2604,2603,2603,2602,2602,2601,2601,2600,2600,2599,2599,2598,2598,2597,2597,2596,2596,2595,2595,2594,2594,2593,2593,2592,2592,2591,2591,2590,2590,2589,2589,2588,2588,2587,2587,2586,2586,2585,2585,2584,2584,2583,2583,2582,2582,2581,2581,2580,2580,2579,2579,2578,2578,2577,2577,2576,2576,2575,2575,2574,2574,2573,2573,2571,2571,2570,2570,2569,2569,2568,2568,2567,2567,2566,2566,2565,2565,2564,2564,2563,2563,2562,2562,2561,2561,2560,2560,2559,2559,2558,2558,2557,2557,2556,2556,2555,2555,2554,2554,2553,2553,2552,2552,2551,2551,2550,2550,2549,2549,2548,2548,2547,2547,2546,2546,2545,2545,2544,2544,2543,2543,2542,2542,2541,2541,2540,2540,2539,2539,2538,2538,2537,2537,2536,2536,2535,2535,2534,2534,2533,2533,2532,2532,2531,2531,2530,2530,2529,2529,2528,2528,2526,2526,2525,2525,2524,2524,2523,2523,2522,2522,2521,2521,2520,2520,2519,2519,2518,2518,2517,2517,2516,2516,2515,2515,2514,2514,2513,2513,2512,2512,2511,2511,2510,2510,2509,2509,2508,2508,2507,2507,2506,2506,2504,2504,2503,2503,2502,2502,2501,2501,2500,2500,2499,2499,2498,2498,2497,2497,2496,2496,2495,2495,2494,2494,2493,2493,2491,2491,2490,2490,2489,2489,2488,2488,2487,2487,2486,2486,2485,2485,2484,2484,2483,2483,2481,2481,2479,2479,2478,2478,2477,2477,2476,2476,2475,2475,2474,2474,2473,2473,2472,2472,2471,2471,2470,2470,2469,2469,2468,2468,2467,2467,2465,2465,2464,2464,2463,2463,2462,2462,2461,2461,2460,2460,2459,2459,2458,2458,2457,2457,2456,2456,2455,2455,2454,2454,2453,2453,2452,2452,2451,2451,2450,2450,2449,2449,2448,2448,2447,2447,2446,2446,2445,2445,2444,2444,2443,2443,2442,2442,2441,2441,2440,2440,2439,2439,2438,2438,2437,2437,2436,2436,2435,2435,2434,2434,2433,2433,2432,2432,2431,2431,2430,2430,2429,2429,2428,2428,2427,2427,2426,2426,2425,2425,2424,2424,2423,2423,2422,2422,2421,2421,2420,2420,2419,2419,2418,2418,2417,2417,2416,2416,2415,2415,2414,2414,2413,2413,2412,2412,2411,2411,2410,2410,2409,2409,2408,2408,2407,2407,2406,2406,2405,2405,2404,2404,2403,2403,2402,2402,2401,2401,2400,2400,2399,2399,2398,2398,2397,2397,2396,2396,2395,2395,2394,2394,2393,2393,2392,2392,2391,2391,2390,2390,2389,2389,2388,2388,2387,2387,2386,2386,2385,2385,2384,2384,2383,2383,2382,2382,2381,2381,2380,2380,2379,2379,2378,2378,2377,2377,2376,2376,2375,2375,2374,2374,2373,2373,2372,2372,2371,2371,2370,2370,2369,2369,2368,2368,2367,2367,2366,2366,2365,2365,2364,2364,2363,2363,2362,2362,2361,2361,2360,2360,2359,2359,2358,2358,2357,2357,2356,2356,2355,2355,2354,2354,2353,2353,2352,2352,2351,2351,2350,2350,2349,2349,2348,2348,2347,2347,2346,2346,2345,2345,2344,2344,2343,2343,2342,2342,2341,2341,2340,2340,2339,2339,2338,2338,2337,2337,2336,2336,2335,2335,2334,2334,2333,2333,2332,2332,2331,2331,2330,2330,2329,2329,2328,2328,2327,2327,2326,2326,2325,2325,2324,2324,2323,2323,2322,2322,2321,2321,2320,2320,2319,2319,2318,2318,2317,2317,2316,2316,2315,2315,2314,2314,2313,2313,2312,2312,2311,2311,2310,2310,2309,2309,2308,2308,2307,2307,2306,2306,2305,2305,2304,2304,2303,2303,2302,2302,2301,2301,2300,2300,2299,2299,2298,2298,2297,2297,2296,2296,2295,2295,2294,2294,2293,2293,2292,2292,2291,2291,2290,2290,2289,2289,2288,2288,2286,2286,2285,2285,2284,2284,2283,2283,2282,2282,2281,2281,2280,2280,2279,2279,2278,2278,2277,2277,2276,2276,2275,2275,2274,2274,2273,2273,2272,2272,2271,2271,2270,2270,2269,2269,2268,2268,2267,2267,2266,2266,2265,2265,2264,2264,2263,2263,2262,2262,2261,2261,2260,2260,2259,2259,2258,2258,2257,2257,2256,2256,2255,2255,2254,2254,2253,2253,2252,2252,2251,2251,2250,2250,2249,2249,2248,2248,2247,2247,2246,2246,2245,2245,2244,2244,2243,2243,2242,2242,2241,2241,2240,2240,2239,2239,2238,2238,2237,2237,2236,2236,2235,2235,2234,2234,2233,2233,2232,2232,2231,2231,2230,2230,2229,2229,2228,2228,2227,2227,2226,2226,2225,2225,2224,2224,2223,2223,2222,2222,2221,2221,2220,2220,2219,2219,2218,2218,2217,2217,2216,2216,2215,2215,2214,2214,2213,2213,2211,2211,2210,2210,2209,2209,2208,2208,2207,2207,2206,2206,2205,2205,2204,2204,2203,2203,2202,2202,2201,2201,2200,2200,2199,2199,2198,2198,2197,2197,2196,2196,2195,2195,2193,2193,2192,2192,2191,2191,2190,2190,2189,2189,2188,2188,2187,2187,2186,2186,2185,2185,2184,2184,2183,2183,2182,2182,2181,2181,2180,2180,2179,2179,2178,2178,2177,2177,2176,2176,2175,2175,2174,2174,2173,2173,2172,2172,2171,2171,2170,2170,2169,2169,2168,2168,2167,2167,2166,2166,2165,2165,2164,2164,2163,2163,2162,2162,2161,2161,2160,2160,2159,2159,2158,2158,2157,2157,2156,2156,2155,2155,2154,2154,2153,2153,2152,2152,2151,2151,2150,2150,2149,2149,2148,2148,2147,2147,2146,2146,2145,2145,2144,2144,2143,2143,2142,2142,2141,2141,2140,2140,2139,2139,2138,2138,2137,2137,2136,2136,2135,2135,2134,2134,2133,2133,2132,2132,2131,2131,2130,2130,2129,2129,2128,2128,2127,2127,2126,2126,2125,2125,2124,2124,2123,2123,2122,2122,2121,2121,2120,2120,2119,2119,2118,2118,2117,2117,2116,2116,2115,2115,2114,2114,2113,2113,2112,2112,2111,2111,2110,2110,2109,2109,2108,2108,2107,2107,2106,2106,2105,2105,2104,2104,2103,2103,2102,2102,2101,2101,2100,2100,2099,2099,2098,2098,2097,2097,2096,2096,2095,2095,2094,2094,2093,2093,2092,2092,2091,2091,2090,2090,2089,2089,2088,2088,2087,2087,2086,2086,2085,2085,2084,2084,2083,2083,2082,2082,2081,2081,2080,2080,2079,2079,2078,2078,2077,2077,2076,2076,2075,2075,2074,2074,2073,2073,2072,2072,2071,2071,2070,2070,2069,2069,2068,2068,2067,2067,2066,2066,2065,2065,2064,2064,2063,2063,2062,2062,2061,2061,2060,2060,2059,2059,2058,2058,2057,2057,2056,2056,2055,2055,2054,2054,2053,2053,2052,2052,2051,2051,2050,2050,2049,2049,2048,2048,2047,2047,2046,2046,2045,2045,2044,2044,2043,2043,2042,2042,2041,2041,2040,2040,2039,2039,2038,2038,2037,2037,2036,2036,2035,2035,2034,2034,2033,2033,2032,2032,2031,2031,2030,2030,2029,2029,2028,2028,2027,2027,2026,2026,2025,2025,2024,2024,2023,2023,2022,2022,2021,2021,2020,2020,2019,2019,2018,2018,2017,2017,2016,2016,2015,2015,2014,2014,2013,2013,2012,2012,2011,2011,2010,2010,2009,2009,2008,2008,2007,2007,2006,2006,2005,2005,2004,2004,2003,2003,2002,2002,2001,2001,2000,2000,1999,1999,1998,1998,1997,1997,1996,1996,1995,1995,1994,1994,1993,1993,1992,1992,1991,1991,1990,1990,1989,1989,1988,1988,1987,1987,1986,1986,1985,1985,1984,1984,1983,1983,1982,1982,1981,1981,1980,1980,1979,1979,1978,1978,1977,1977,1976,1976,1975,1975,1974,1974,1973,1973,1972,1972,1971,1971,1970,1970,1969,1969,1968,1968,1967,1967,1966,1966,1965,1965,1964,1964,1963,1963,1962,1962,1961,1961,1960,1960,1959,1959,1958,1958,1957,1957,1956,1956,1955,1955,1954,1954,1953,1953,1952,1952,1951,1951,1950,1950,1949,1949,1948,1948,1947,1947,1946,1946,1945,1945,1944,1944,1943,1943,1942,1942,1941,1941,1940,1940,1939,1939,1938,1938,1937,1937,1936,1936,1935,1935,1934,1934,1933,1933,1932,1932,1931,1931,1930,1930,1929,1929,1928,1928,1927,1927,1926,1926,1925,1925,1924,1924,1923,1923,1922,1922,1921,1921,1920,1920,1919,1919,1918,1918,1917,1917,1916,1916,1915,1915,1914,1914,1913,1913,1912,1912,1911,1911,1910,1910,1909,1909,1908,1908,1907,1907,1906,1906,1905,1905,1904,1904,1903,1903,1902,1902,1901,1901,1900,1900,1899,1899,1898,1898,1897,1897,1896,1896,1895,1895,1894,1894,1893,1893,1892,1892,1891,1891,1890,1890,1889,1889,1888,1888,1887,1887,1886,1886,1885,1885,1884,1884,1883,1883,1882,1882,1881,1881,1880,1880,1879,1879,1878,1878,1877,1877,1876,1876,1875,1875,1874,1874,1873,1873,1872,1872,1871,1871,1870,1870,1869,1869,1868,1868,1867,1867,1866,1866,1865,1865,1864,1864,1863,1863,1862,1862,1861,1861,1860,1860,1859,1859,1858,1858,1857,1857,1856,1856,1855,1855,1854,1854,1853,1853,1852,1852,1851,1851,1850,1850,1849,1849,1848,1848,1847,1847,1846,1846,1845,1845,1844,1844,1843,1843,1842,1842,1841,1841,1840,1840,1839,1839,1838,1838,1837,1837,1836,1836,1835,1835,1834,1834,1833,1833,1832,1832,1831,1831,1830,1830,1829,1829,1828,1828,1827,1827,1826,1826,1825,1825,1824,1824,1823,1823,1822,1822,1821,1821,1820,1820,1819,1819,1818,1818,1817,1817,1816,1816,1815,1815,1814,1814,1813,1813,1812,1812,1811,1811,1810,1810,1809,1809,1808,1808,1807,1807,1806,1806,1805,1805,1804,1804,1803,1803,1802,1802,1801,1801,1800,1800,1799,1799,1798,1798,1797,1797,1796,1796,1795,1795,1794,1794,1793,1793,1792,1792,1791,1791,1790,1790,1789,1789,1788,1788,1787,1787,1786,1786,1785,1785,1784,1784,1783,1783,1782,1782,1781,1781,1780,1780,1779,1779,1778,1778,1777,1777,1776,1776,1775,1775,1774,1774,1773,1773,1772,1772,1771,1771,1770,1770,1769,1769,1768,1768,1767,1767,1766,1766,1765,1765,1764,1764,1763,1763,1762,1762,1761,1761,1760,1760,1759,1759,1758,1758,1757,1757,1756,1756,1755,1755,1754,1754,1753,1753,1752,1752,1751,1751,1750,1750,1749,1749,1748,1748,1747,1747,1746,1746,1745,1745,1744,1744,1743,1743,1742,1742,1741,1741,1740,1740,1739,1739,1738,1738,1737,1737,1736,1736,1735,1735,1734,1734,1733,1733,1732,1732,1731,1731,1730,1730,1729,1729,1728,1728,1727,1727,1726,1726,1725,1725,1724,1724,1723,1723,1722,1722,1721,1721,1720,1720,1719,1719,1718,1718,1717,1717,1716,1716,1715,1715,1714,1714,1713,1713,1712,1712,1711,1711,1710,1710,1709,1709,1708,1708,1707,1707,1706,1706,1705,1705,1704,1704,1703,1703,1702,1702,1701,1701,1700,1700,1699,1699,1698,1698,1697,1697,1696,1696,1695,1695,1694,1694,1693,1693,1692,1692,1691,1691,1690,1690,1689,1689,1688,1688,1687,1687,1686,1686,1685,1685,1684,1684,1683,1683,1682,1682,1681,1681,1680,1680,1679,1679,1678,1678,1677,1677,1676,1676,1675,1675,1674,1674,1673,1673,1672,1672,1671,1671,1670,1670,1669,1669,1668,1668,1667,1667,1666,1666,1665,1665,1664,1664,1663,1663,1662,1662,1661,1661,1660,1660,1659,1659,1658,1658,1657,1657,1656,1656,1655,1655,1654,1654,1653,1653,1652,1652,1651,1651,1650,1650,1649,1649,1648,1648,1647,1647,1646,1646,1645,1645,1644,1644,1643,1643,1642,1642,1641,1641,1640,1640,1639,1639,1638,1638,1637,1637,1636,1636,1635,1635,1634,1634,1633,1633,1632,1632,1631,1631,1630,1630,1629,1629,1628,1628,1627,1627,1626,1626,1625,1625,1624,1624,1623,1623,1622,1622,1621,1621,1620,1620,1619,1619,1618,1618,1617,1617,1616,1616,1615,1615,1614,1614,1613,1613,1612,1612,1611,1611,1610,1610,1609,1609,1608,1608,1607,1607,1606,1606,1605,1605,1604,1604,1603,1603,1602,1602,1601,1601,1600,1600,1599,1599,1598,1598,1597,1597,1596,1596,1595,1595,1594,1594,1593,1593,1592,1592,1591,1591,1590,1590,1589,1589,1588,1588,1587,1587,1586,1586,1585,1585,1584,1584,1583,1583,1582,1582,1581,1581,1580,1580,1579,1579,1578,1578,1577,1577,1576,1576,1575,1575,1574,1574,1573,1573,1572,1572,1571,1571,1570,1570,1569,1569,1568,1568,1567,1567,1566,1566,1565,1565,1564,1564,1563,1563,1562,1562,1561,1561,1560,1560,1559,1559,1558,1558,1557,1557,1556,1556,1555,1555,1554,1554,1553,1553,1552,1552,1551,1551,1550,1550,1549,1549,1548,1548,1547,1547,1546,1546,1545,1545,1544,1544,1543,1543,1542,1542,1541,1541,1540,1540,1539,1539,1538,1538,1537,1537,1536,1536,1535,1535,1534,1534,1533,1533,1532,1532,1531,1531,1530,1530,1529,1529,1528,1528,1527,1527,1526,1526,1525,1525,1524,1524,1523,1523,1522,1522,1521,1521,1520,1520,1519,1519,1518,1518,1517,1517,1516,1516,1515,1515,1514,1514,1513,1513,1512,1512,1511,1511,1510,1510,1509,1509,1508,1508,1507,1507,1506,1506,1505,1505,1504,1504,1503,1503,1502,1502,1501,1501,1500,1500,1499,1499,1498,1498,1497,1497,1496,1496,1495,1495,1494,1494,1493,1493,1492,1492,1491,1491,1490,1490,1489,1489,1488,1488,1487,1487,1486,1486,1485,1485,1484,1484,1483,1483,1482,1482,1481,1481,1480,1480,1479,1479,1478,1478,1477,1477,1476,1476,1475,1475,1474,1474,1473,1473,1472,1472,1471,1471,1470,1470,1469,1469,1468,1468,1467,1467,1466,1466,1465,1465,1464,1464,1463,1463,1462,1462,1461,1461,1460,1460,1459,1459,1458,1458,1457,1457,1456,1456,1455,1455,1454,1454,1453,1453,1452,1452,1451,1451,1450,1450,1449,1449,1448,1448,1447,1447,1446,1446,1445,1445,1444,1444,1443,1443,1442,1442,1441,1441,1440,1440,1439,1439,1438,1438,1437,1437,1436,1436,1435,1435,1434,1434,1433,1433,1432,1432,1431,1431,1430,1430,1429,1429,1428,1428,1427,1427,1426,1426,1425,1425,1424,1424,1423,1423,1422,1422,1421,1421,1420,1420,1419,1419,1418,1418,1417,1417,1416,1416,1415,1415,1414,1414,1413,1413,1412,1412,1411,1411,1410,1410,1409,1409,1408,1408,1407,1407,1406,1406,1405,1405,1404,1404,1403,1403,1402,1402,1401,1401,1400,1400,1399,1399,1398,1398,1397,1397,1396,1396,1395,1395,1394,1394,1393,1393,1392,1392,1391,1391,1390,1390,1389,1389,1388,1388,1387,1387,1386,1386,1385,1385,1384,1384,1383,1383,1382,1382,1381,1381,1380,1380,1379,1379,1378,1378,1377,1377,1376,1376,1375,1375,1374,1374,1373,1373,1372,1372,1371,1371,1370,1370,1369,1369,1368,1368,1367,1367,1366,1366,1365,1365,1364,1364,1363,1363,1362,1362,1361,1361,1360,1360,1359,1359,1358,1358,1357,1357,1356,1356,1355,1355,1354,1354,1353,1353,1352,1352,1351,1351,1350,1350,1349,1349,1348,1348,1347,1347,1346,1346,1345,1345,1344,1344,1343,1343,1342,1342,1341,1341,1340,1340,1339,1339,1338,1338,1337,1337,1336,1336,1335,1335,1334,1334,1333,1333,1332,1332,1331,1331,1330,1330,1329,1329,1328,1328,1327,1327,1326,1326,1325,1325,1324,1324,1323,1323,1322,1322,1321,1321,1320,1320,1319,1319,1318,1318,1317,1317,1316,1316,1315,1315,1314,1314,1313,1313,1312,1312,1311,1311,1310,1310,1309,1309,1308,1308,1307,1307,1306,1306,1305,1305,1304,1304,1303,1303,1302,1302,1301,1301,1300,1300,1299,1299,1298,1298,1297,1297,1296,1296,1295,1295,1294,1294,1293,1293,1292,1292,1291,1291,1290,1290,1289,1289,1288,1288,1287,1287,1286,1286,1285,1285,1284,1284,1283,1283,1282,1282,1281,1281,1280,1280,1279,1279,1278,1278,1277,1277,1276,1276,1275,1275,1274,1274,1273,1273,1272,1272,1271,1271,1270,1270,1269,1269,1268,1268,1267,1267,1266,1266,1265,1265,1264,1264,1263,1263,1262,1262,1261,1261,1260,1260,1259,1259,1258,1258,1257,1257,1256,1256,1255,1255,1254,1254,1253,1253,1252,1252,1251,1251,1250,1250,1249,1249,1248,1248,1247,1247,1246,1246,1245,1245,1244,1244,1243,1243,1242,1242,1241,1241,1240,1240,1239,1239,1238,1238,1237,1237,1236,1236,1235,1235,1234,1234,1233,1233,1232,1232,1231,1231,1230,1230,1229,1229,1228,1228,1227,1227,1226,1226,1225,1225,1224,1224,1223,1223,1222,1222,1221,1221,1220,1220,1219,1219,1218,1218,1217,1217,1216,1216,1215,1215,1214,1214,1213,1213,1212,1212,1211,1211,1210,1210,1209,1209,1208,1208,1207,1207,1206,1206,1205,1205,1204,1204,1203,1203,1202,1202,1201,1201,1200,1200,1199,1199,1198,1198,1197,1197,1196,1196,1195,1195,1194,1194,1193,1193,1192,1192,1191,1191,1190,1190,1189,1189,1188,1188,1187,1187,1186,1186,1185,1185,1184,1184,1183,1183,1182,1182,1181,1181,1180,1180,1179,1179,1178,1178,1177,1177,1176,1176,1175,1175,1174,1174,1173,1173,1172,1172,1171,1171,1170,1170,1169,1169,1168,1168,1167,1167,1166,1166,1165,1165,1164,1164,1163,1163,1162,1162,1161,1161,1160,1160,1159,1159,1158,1158,1157,1157,1156,1156,1155,1155,1154,1154,1153,1153,1152,1152,1151,1151,1150,1150,1149,1149,1148,1148,1147,1147,1146,1146,1145,1145,1144,1144,1143,1143,1142,1142,1141,1141,1140,1140,1139,1139,1138,1138,1137,1137,1136,1136,1135,1135,1134,1134,1133,1133,1132,1132,1131,1131,1130,1130,1129,1129,1128,1128,1127,1127,1126,1126,1125,1125,1124,1124,1123,1123,1122,1122,1121,1121,1120,1120,1119,1119,1118,1118,1117,1117,1116,1116,1115,1115,1114,1114,1113,1113,1112,1112,1111,1111,1110,1110,1109,1109,1108,1108,1107,1107,1106,1106,1105,1105,1104,1104,1103,1103,1102,1102,1101,1101,1100,1100,1099,1099,1098,1098,1097,1097,1096,1096,1095,1095,1094,1094,1093,1093,1092,1092,1091,1091,1090,1090,1089,1089,1088,1088,1087,1087,1086,1086,1085,1085,1084,1084,1083,1083,1082,1082,1081,1081,1080,1080,1079,1079,1078,1078,1077,1077,1076,1076,1075,1075,1074,1074,1073,1073,1072,1072,1071,1071,1070,1070,1069,1069,1068,1068,1067,1067,1066,1066,1065,1065,1064,1064,1063,1063,1062,1062,1061,1061,1060,1060,1059,1059,1058,1058,1057,1057,1056,1056,1055,1055,1054,1054,1053,1053,1052,1052,1051,1051,1050,1050,1049,1049,1048,1048,1047,1047,1046,1046,1045,1045,1044,1044,1043,1043,1042,1042,1041,1041,1040,1040,1039,1039,1038,1038,1037,1037,1036,1036,1035,1035,1034,1034,1033,1033,1032,1032,1031,1031,1030,1030,1029,1029,1028,1028,1027,1027,1026,1026,1025,1025,1024,1024,1023,1023,1022,1022,1021,1021,1020,1020,1019,1019,1018,1018,1017,1017,1016,1016,1015,1015,1014,1014,1013,1013,1012,1012,1011,1011,1010,1010,1009,1009,1008,1008,1007,1007,1006,1006,1005,1005,1004,1004,1003,1003,1002,1002,1001,1001,1000,1000,999,999,998,998,997,997,996,996,995,995,994,994,993,993,992,992,991,991,990,990,989,989,988,988,987,987,986,986,985,985,984,984,983,983,982,982,981,981,980,980,979,979,978,978,977,977,976,976,975,975,974,974,973,973,972,972,971,971,970,970,969,969,968,968,967,967,966,966,965,965,964,964,963,963,962,962,961,961,960,960,959,959,958,958,957,957,956,956,955,955,954,954,953,953,952,952,951,951,950,950,949,949,948,948,947,947,946,946,945,945,944,944,943,943,942,942,941,941,940,940,939,939,938,938,937,937,936,936,935,935,934,934,933,933,932,932,931,931,930,930,929,929,928,928,927,927,926,926,925,925,924,924,923,923,922,922,921,921,920,920,919,919,918,918,917,917,916,916,915,915,914,914,913,913,912,912,911,911,910,910,909,909,908,908,907,907,906,906,905,905,904,904,903,903,902,902,901,901,900,900,899,899,898,898,897,897,896,896,895,895,894,894,893,893,892,892,891,891,890,890,889,889,888,888,887,887,886,886,885,885,884,884,883,883,882,882,881,881,880,880,879,879,878,878,877,877,876,876,875,875,874,874,873,873,872,872,871,871,870,870,869,869,868,868,867,867,866,866,865,865,864,864,863,863,862,862,861,861,860,860,859,859,858,858,857,857,856,856,855,855,854,854,853,853,852,852,851,851,850,850,849,849,848,848,847,847,846,846,845,845,844,844,843,843,842,842,841,841,840,840,839,839,838,838,837,837,836,836,835,835,834,834,833,833,832,832,831,831,830,830,829,829,828,828,827,827,826,826,825,825,824,824,823,823,822,822,821,821,820,820,819,819,818,818,817,817,816,816,815,815,814,814,813,813,812,812,811,811,810,810,809,809,808,808,807,807,806,806,805,805,804,804,803,803,802,802,801,801,800,800,799,799,798,798,797,797,796,796,795,795,794,794,793,793,792,792,791,791,790,790,789,789,788,788,787,787,786,786,785,785,784,784,783,783,782,782,781,781,780,780,779,779,778,778,777,777,776,776,775,775,774,774,773,773,772,772,771,771,770,770,769,769,768,768,767,767,766,766,765,765,764,764,763,763,762,762,761,761,760,760,759,759,758,758,757,757,756,756,755,755,754,754,753,753,752,752,751,751,750,750,749,749,748,748,747,747,746,746,745,745,744,744,743,743,742,742,741,741,740,740,739,739,738,738,737,737,736,736,735,735,734,734,733,733,732,732,731,731,730,730,729,729,728,728,727,727,726,726,725,725,724,724,723,723,722,722,721,721,720,720,719,719,718,718,717,717,716,716,715,715,714,714,713,713,712,712,711,711,710,710,709,709,708,708,707,707,706,706,705,705,704,704,703,703,702,702,701,701,700,700,699,699,698,698,697,697,696,696,695,695,694,694,693,693,692,692,691,691,690,690,689,689,688,688,687,687,686,686,685,685,684,684,683,683,682,682,681,681,680,680,679,679,678,678,677,677,676,676,675,675,674,674,673,673,672,672,671,671,670,670,669,669,668,668,667,667,666,666,665,665,664,664,663,663,662,662,661,661,660,660,659,659,658,658,657,657,656,656,655,655,654,654,653,653,652,652,651,651,650,650,649,649,648,648,647,647,646,646,645,645,644,644,643,643,642,642,641,641,640,640,639,639,638,638,637,637,636,636,635,635,634,634,633,633,632,632,631,631,630,630,629,629,628,628,627,627,626,626,625,625,624,624,623,623,622,622,621,621,620,620,619,619,618,618,617,617,616,616,615,615,614,614,613,613,612,612,611,611,610,610,609,609,608,608,607,607,606,606,605,605,604,604,603,603,602,602,601,601,600,600,599,599,598,598,597,597,596,596,595,595,594,594,593,593,592,592,591,591,590,590,589,589,588,588,587,587,586,586,585,585,584,584,583,583,582,582,581,581,580,580,579,579,578,578,577,577,576,576,575,575,574,574,573,573,572,572,571,571,570,570,569,569,568,568,567,567,566,566,565,565,564,564,563,563,562,562,561,561,560,560,559,559,558,558,557,557,556,556,555,555,554,554,553,553,552,552,551,551,550,550,549,549,548,548,547,547,546,546,545,545,544,544,543,543,542,542,541,541,540,540,539,539,538,538,537,537,536,536,535,535,534,534,533,533,532,532,531,531,530,530,529,529,528,528,527,527,526,526,525,525,524,524,523,523,522,522,521,521,520,520,519,519,518,518,517,517,516,516,515,515,514,514,513,513,512,512,511,511,510,510,509,509,508,508,507,507,506,506,505,505,504,504,503,503,502,502,501,501,500,500,499,499,498,498,497,497,496,496,495,495,494,494,493,493,492,492,491,491,490,490,489,489,488,488,487,487,486,486,485,485,484,484,483,483,482,482,481,481,480,480,479,479,478,478,477,477,476,476,475,475,474,474,473,473,472,472,471,471,470,470,469,469,468,468,467,467,466,466,465,465,464,464,463,463,462,462,461,461,460,460,459,459,458,458,457,457,456,456,455,455,454,454,453,453,452,452,451,451,450,450,449,449,448,448,447,447,446,446,445,445,444,444,443,443,442,442,441,441,440,440,439,439,438,438,437,437,436,436,435,435,434,434,433,433,432,432,431,431,430,430,429,429,428,428,427,427,426,426,425,425,424,424,423,423,422,422,421,421,420,420,419,419,418,418,417,417,416,416,415,415,414,414,413,413,412,412,411,411,410,410,409,409,408,408,407,407,406,406,405,405,404,404,403,403,402,402,401,401,400,400,0.0],[29245,29245,29245,27353,27353,25659,25659,25633,25633,23651,23651,23598,23598,23362,23362,23202,23202,22916,22916,22777,22777,22370,22370,22072,22072,21832,21832,21820,21820,21755,21755,21496,21496,21372,21372,21345,21345,21262,21262,20680,20680,20636,20636,20186,20186,20105,20105,20103,20103,19831,19831,19818,19818,19560,19560,18729,18729,18662,18662,18242,18242,17729,17729,17644,17644,17481,17481,17471,17471,17422,17422,17061,17061,16944,16944,16788,16788,16671,16671,16652,16652,16476,16476,16459,16459,16397,16397,16228,16228,16192,16192,16148,16148,16063,16063,16035,16035,15975,15975,15900,15900,15854,15854,15784,15784,15517,15517,15502,15502,15456,15456,15453,15453,15207,15207,15174,15174,15125,15125,15024,15024,14858,14858,14736,14736,14727,14727,14638,14638,14613,14613,14552,14552,14458,14458,14409,14409,14325,14325,14321,14321,14291,14291,14289,14289,14218,14218,14199,14199,14178,14178,14176,14176,14072,14072,14070,14070,13994,13994,13978,13978,13941,13941,13938,13938,13906,13906,13891,13891,13837,13837,13833,13833,13817,13817,13717,13717,13645,13645,13644,13644,13563,13563,13542,13542,13400,13400,13377,13377,13347,13347,13319,13319,13203,13203,13177,13177,13073,13073,13063,13063,12971,12971,12961,12961,12958,12958,12930,12930,12923,12923,12899,12899,12861,12861,12788,12788,12787,12787,12786,12786,12775,12775,12770,12770,12763,12763,12758,12758,12750,12750,12742,12742,12689,12689,12654,12654,12642,12642,12612,12612,12608,12608,12446,12446,12444,12444,12441,12441,12435,12435,12343,12343,12295,12295,12227,12227,12154,12154,12127,12127,12088,12088,12073,12073,12053,12053,12008,12008,12001,12001,11984,11984,11966,11966,11962,11962,11943,11943,11931,11931,11896,11896,11886,11886,11862,11862,11841,11841,11752,11752,11728,11728,11687,11687,11685,11685,11633,11633,11610,11610,11592,11592,11549,11549,11528,11528,11517,11517,11482,11482,11413,11413,11399,11399,11373,11373,11370,11370,11367,11367,11320,11320,11311,11311,11296,11296,11234,11234,11221,11221,11205,11205,11179,11179,11155,11155,11154,11154,11106,11106,11104,11104,11094,11094,11085,11085,11014,11014,11006,11006,10999,10999,10987,10987,10907,10907,10880,10880,10861,10861,10856,10856,10854,10854,10847,10847,10843,10843,10821,10821,10807,10807,10791,10791,10767,10767,10755,10755,10743,10743,10673,10673,10666,10666,10656,10656,10655,10655,10589,10589,10579,10579,10573,10573,10516,10516,10505,10505,10479,10479,10472,10472,10465,10465,10464,10464,10459,10459,10447,10447,10397,10397,10396,10396,10384,10384,10377,10377,10376,10376,10373,10373,10365,10365,10322,10322,10315,10315,10312,10312,10308,10308,10289,10289,10265,10265,10262,10262,10252,10252,10234,10234,10223,10223,10214,10214,10176,10176,10161,10161,10136,10136,10044,10044,10007,10007,9992,9992,9984,9984,9955,9955,9936,9936,9880,9880,9878,9878,9863,9863,9852,9852,9846,9846,9845,9845,9840,9840,9838,9838,9803,9803,9801,9801,9771,9771,9766,9766,9759,9759,9754,9754,9739,9739,9707,9707,9695,9695,9683,9683,9656,9656,9644,9644,9625,9625,9622,9622,9561,9561,9536,9536,9531,9531,9520,9520,9517,9517,9495,9495,9463,9463,9434,9434,9422,9422,9401,9401,9396,9396,9391,9391,9384,9384,9378,9378,9307,9307,9270,9270,9242,9242,9239,9239,9216,9216,9214,9214,9213,9213,9204,9204,9185,9185,9182,9182,9180,9180,9152,9152,9142,9142,9117,9117,9109,9109,9099,9099,9083,9083,9056,9056,9053,9053,9046,9046,9034,9034,9030,9030,8974,8974,8959,8959,8947,8947,8936,8936,8915,8915,8893,8893,8887,8887,8863,8863,8843,8843,8832,8832,8823,8823,8819,8819,8808,8808,8783,8783,8768,8768,8763,8763,8746,8746,8726,8726,8719,8719,8692,8692,8691,8691,8686,8686,8676,8676,8672,8672,8669,8669,8661,8661,8659,8659,8655,8655,8645,8645,8644,8644,8625,8625,8624,8624,8602,8602,8581,8581,8562,8562,8548,8548,8536,8536,8510,8510,8499,8499,8498,8498,8457,8457,8453,8453,8452,8452,8449,8449,8432,8432,8413,8413,8410,8410,8406,8406,8398,8398,8387,8387,8376,8376,8353,8353,8347,8347,8318,8318,8305,8305,8302,8302,8297,8297,8289,8289,8280,8280,8241,8241,8238,8238,8231,8231,8220,8220,8176,8176,8174,8174,8147,8147,8145,8145,8137,8137,8125,8125,8120,8120,8117,8117,8111,8111,8104,8104,8093,8093,8078,8078,8077,8077,8069,8069,8040,8040,8024,8024,8017,8017,8015,8015,8012,8012,8011,8011,7991,7991,7976,7976,7972,7972,7970,7970,7964,7964,7949,7949,7946,7946,7944,7944,7935,7935,7933,7933,7932,7932,7930,7930,7928,7928,7925,7925,7923,7923,7906,7906,7904,7904,7898,7898,7887,7887,7886,7886,7884,7884,7883,7883,7865,7865,7859,7859,7847,7847,7840,7840,7835,7835,7831,7831,7818,7818,7804,7804,7785,7785,7775,7775,7772,7772,7765,7765,7762,7762,7755,7755,7750,7750,7725,7725,7710,7710,7698,7698,7694,7694,7693,7693,7689,7689,7686,7686,7685,7685,7683,7683,7673,7673,7671,7671,7665,7665,7654,7654,7652,7652,7644,7644,7624,7624,7621,7621,7619,7619,7618,7618,7613,7613,7602,7602,7599,7599,7595,7595,7578,7578,7564,7564,7563,7563,7557,7557,7554,7554,7548,7548,7540,7540,7527,7527,7521,7521,7515,7515,7510,7510,7505,7505,7500,7500,7499,7499,7485,7485,7484,7484,7483,7483,7465,7465,7460,7460,7459,7459,7435,7435,7434,7434,7429,7429,7414,7414,7406,7406,7399,7399,7391,7391,7389,7389,7385,7385,7380,7380,7374,7374,7345,7345,7343,7343,7332,7332,7331,7331,7308,7308,7303,7303,7302,7302,7299,7299,7291,7291,7287,7287,7268,7268,7265,7265,7263,7263,7251,7251,7249,7249,7241,7241,7231,7231,7225,7225,7214,7214,7208,7208,7207,7207,7205,7205,7195,7195,7194,7194,7192,7192,7191,7191,7180,7180,7166,7166,7164,7164,7156,7156,7150,7150,7148,7148,7142,7142,7134,7134,7131,7131,7109,7109,7106,7106,7101,7101,7088,7088,7083,7083,7082,7082,7077,7077,7075,7075,7071,7071,7063,7063,7049,7049,7048,7048,7042,7042,7033,7033,7030,7030,7026,7026,7022,7022,7016,7016,7013,7013,7008,7008,7006,7006,7000,7000,6995,6995,6994,6994,6991,6991,6990,6990,6988,6988,6985,6985,6977,6977,6976,6976,6975,6975,6973,6973,6966,6966,6956,6956,6952,6952,6949,6949,6926,6926,6922,6922,6896,6896,6893,6893,6851,6851,6850,6850,6844,6844,6832,6832,6816,6816,6813,6813,6806,6806,6805,6805,6804,6804,6800,6800,6796,6796,6795,6795,6794,6794,6783,6783,6779,6779,6755,6755,6753,6753,6742,6742,6740,6740,6738,6738,6737,6737,6735,6735,6724,6724,6723,6723,6716,6716,6711,6711,6703,6703,6700,6700,6699,6699,6697,6697,6684,6684,6682,6682,6681,6681,6680,6680,6677,6677,6676,6676,6674,6674,6668,6668,6663,6663,6661,6661,6657,6657,6656,6656,6646,6646,6639,6639,6631,6631,6622,6622,6609,6609,6589,6589,6583,6583,6580,6580,6574,6574,6567,6567,6565,6565,6564,6564,6563,6563,6553,6553,6550,6550,6549,6549,6547,6547,6545,6545,6534,6534,6531,6531,6521,6521,6515,6515,6513,6513,6502,6502,6496,6496,6493,6493,6492,6492,6490,6490,6486,6486,6485,6485,6469,6469,6460,6460,6456,6456,6451,6451,6443,6443,6441,6441,6436,6436,6435,6435,6434,6434,6428,6428,6424,6424,6416,6416,6407,6407,6400,6400,6392,6392,6386,6386,6384,6384,6381,6381,6380,6380,6377,6377,6373,6373,6362,6362,6348,6348,6346,6346,6337,6337,6335,6335,6333,6333,6332,6332,6322,6322,6320,6320,6314,6314,6309,6309,6299,6299,6295,6295,6292,6292,6288,6288,6279,6279,6275,6275,6271,6271,6267,6267,6266,6266,6262,6262,6260,6260,6254,6254,6246,6246,6245,6245,6244,6244,6243,6243,6227,6227,6223,6223,6222,6222,6219,6219,6218,6218,6207,6207,6206,6206,6199,6199,6191,6191,6184,6184,6182,6182,6181,6181,6177,6177,6176,6176,6171,6171,6165,6165,6160,6160,6150,6150,6149,6149,6147,6147,6144,6144,6141,6141,6132,6132,6121,6121,6116,6116,6112,6112,6111,6111,6106,6106,6104,6104,6102,6102,6100,6100,6099,6099,6098,6098,6093,6093,6084,6084,6083,6083,6073,6073,6058,6058,6057,6057,6056,6056,6051,6051,6045,6045,6040,6040,6039,6039,6037,6037,6035,6035,6028,6028,6026,6026,6023,6023,6022,6022,6018,6018,6011,6011,6009,6009,6006,6006,6002,6002,5992,5992,5990,5990,5982,5982,5978,5978,5976,5976,5975,5975,5971,5971,5969,5969,5967,5967,5964,5964,5955,5955,5947,5947,5944,5944,5941,5941,5938,5938,5937,5937,5936,5936,5935,5935,5932,5932,5930,5930,5925,5925,5916,5916,5911,5911,5900,5900,5897,5897,5887,5887,5884,5884,5882,5882,5865,5865,5863,5863,5853,5853,5852,5852,5851,5851,5848,5848,5844,5844,5843,5843,5842,5842,5838,5838,5834,5834,5825,5825,5821,5821,5820,5820,5816,5816,5815,5815,5813,5813,5810,5810,5799,5799,5797,5797,5794,5794,5793,5793,5792,5792,5789,5789,5784,5784,5768,5768,5763,5763,5761,5761,5757,5757,5751,5751,5748,5748,5745,5745,5736,5736,5732,5732,5731,5731,5730,5730,5718,5718,5717,5717,5714,5714,5713,5713,5709,5709,5699,5699,5698,5698,5695,5695,5686,5686,5684,5684,5681,5681,5678,5678,5677,5677,5669,5669,5668,5668,5665,5665,5663,5663,5654,5654,5653,5653,5651,5651,5648,5648,5642,5642,5641,5641,5634,5634,5631,5631,5626,5626,5623,5623,5621,5621,5618,5618,5617,5617,5612,5612,5608,5608,5606,5606,5602,5602,5597,5597,5588,5588,5587,5587,5586,5586,5584,5584,5582,5582,5579,5579,5572,5572,5571,5571,5565,5565,5562,5562,5559,5559,5557,5557,5554,5554,5551,5551,5548,5548,5546,5546,5541,5541,5539,5539,5534,5534,5528,5528,5521,5521,5517,5517,5515,5515,5512,5512,5507,5507,5497,5497,5490,5490,5489,5489,5488,5488,5482,5482,5475,5475,5470,5470,5469,5469,5465,5465,5454,5454,5452,5452,5447,5447,5445,5445,5440,5440,5435,5435,5430,5430,5426,5426,5425,5425,5424,5424,5417,5417,5411,5411,5410,5410,5409,5409,5404,5404,5402,5402,5399,5399,5394,5394,5392,5392,5390,5390,5387,5387,5385,5385,5383,5383,5377,5377,5375,5375,5369,5369,5356,5356,5352,5352,5351,5351,5349,5349,5345,5345,5342,5342,5341,5341,5338,5338,5335,5335,5332,5332,5328,5328,5327,5327,5320,5320,5319,5319,5318,5318,5316,5316,5311,5311,5303,5303,5302,5302,5301,5301,5297,5297,5296,5296,5290,5290,5284,5284,5283,5283,5282,5282,5280,5280,5278,5278,5276,5276,5274,5274,5269,5269,5267,5267,5266,5266,5265,5265,5261,5261,5260,5260,5258,5258,5256,5256,5255,5255,5254,5254,5250,5250,5247,5247,5246,5246,5245,5245,5242,5242,5241,5241,5239,5239,5236,5236,5232,5232,5229,5229,5226,5226,5224,5224,5223,5223,5221,5221,5216,5216,5213,5213,5210,5210,5205,5205,5203,5203,5202,5202,5201,5201,5200,5200,5196,5196,5195,5195,5193,5193,5186,5186,5184,5184,5182,5182,5181,5181,5180,5180,5175,5175,5172,5172,5171,5171,5166,5166,5152,5152,5151,5151,5149,5149,5148,5148,5141,5141,5136,5136,5129,5129,5128,5128,5126,5126,5125,5125,5122,5122,5120,5120,5111,5111,5107,5107,5106,5106,5103,5103,5102,5102,5100,5100,5096,5096,5093,5093,5092,5092,5091,5091,5089,5089,5081,5081,5078,5078,5076,5076,5072,5072,5070,5070,5068,5068,5067,5067,5066,5066,5065,5065,5058,5058,5057,5057,5055,5055,5054,5054,5053,5053,5049,5049,5046,5046,5041,5041,5040,5040,5039,5039,5036,5036,5034,5034,5032,5032,5023,5023,5019,5019,5012,5012,5011,5011,5008,5008,5007,5007,5004,5004,5003,5003,5002,5002,5000,5000,4999,4999,4988,4988,4987,4987,4985,4985,4977,4977,4975,4975,4974,4974,4973,4973,4970,4970,4962,4962,4959,4959,4958,4958,4954,4954,4945,4945,4944,4944,4942,4942,4937,4937,4935,4935,4934,4934,4932,4932,4931,4931,4930,4930,4929,4929,4926,4926,4924,4924,4923,4923,4921,4921,4920,4920,4916,4916,4915,4915,4910,4910,4909,4909,4906,4906,4905,4905,4904,4904,4901,4901,4894,4894,4893,4893,4890,4890,4889,4889,4888,4888,4887,4887,4884,4884,4882,4882,4879,4879,4876,4876,4873,4873,4871,4871,4867,4867,4864,4864,4863,4863,4860,4860,4858,4858,4849,4849,4848,4848,4843,4843,4842,4842,4841,4841,4839,4839,4831,4831,4828,4828,4825,4825,4824,4824,4817,4817,4815,4815,4814,4814,4813,4813,4811,4811,4810,4810,4808,4808,4804,4804,4803,4803,4799,4799,4793,4793,4791,4791,4790,4790,4789,4789,4787,4787,4786,4786,4780,4780,4775,4775,4774,4774,4772,4772,4771,4771,4770,4770,4769,4769,4768,4768,4758,4758,4756,4756,4750,4750,4748,4748,4746,4746,4745,4745,4743,4743,4741,4741,4737,4737,4729,4729,4722,4722,4719,4719,4718,4718,4715,4715,4714,4714,4702,4702,4701,4701,4698,4698,4695,4695,4693,4693,4689,4689,4688,4688,4687,4687,4685,4685,4683,4683,4682,4682,4680,4680,4679,4679,4676,4676,4667,4667,4666,4666,4663,4663,4655,4655,4653,4653,4646,4646,4643,4643,4640,4640,4638,4638,4635,4635,4634,4634,4633,4633,4632,4632,4629,4629,4628,4628,4627,4627,4621,4621,4619,4619,4618,4618,4614,4614,4613,4613,4610,4610,4608,4608,4607,4607,4606,4606,4604,4604,4602,4602,4601,4601,4599,4599,4594,4594,4592,4592,4584,4584,4581,4581,4577,4577,4575,4575,4574,4574,4570,4570,4569,4569,4566,4566,4565,4565,4563,4563,4560,4560,4559,4559,4553,4553,4551,4551,4549,4549,4548,4548,4544,4544,4543,4543,4539,4539,4534,4534,4533,4533,4530,4530,4529,4529,4527,4527,4525,4525,4522,4522,4519,4519,4517,4517,4515,4515,4514,4514,4512,4512,4510,4510,4509,4509,4508,4508,4506,4506,4503,4503,4502,4502,4501,4501,4497,4497,4495,4495,4494,4494,4491,4491,4486,4486,4482,4482,4481,4481,4479,4479,4474,4474,4473,4473,4467,4467,4466,4466,4462,4462,4460,4460,4458,4458,4456,4456,4453,4453,4450,4450,4447,4447,4446,4446,4442,4442,4440,4440,4437,4437,4434,4434,4430,4430,4429,4429,4428,4428,4425,4425,4418,4418,4415,4415,4412,4412,4411,4411,4410,4410,4409,4409,4398,4398,4392,4392,4387,4387,4382,4382,4381,4381,4380,4380,4379,4379,4378,4378,4375,4375,4374,4374,4373,4373,4370,4370,4368,4368,4364,4364,4363,4363,4361,4361,4360,4360,4355,4355,4354,4354,4352,4352,4350,4350,4346,4346,4345,4345,4343,4343,4342,4342,4338,4338,4337,4337,4336,4336,4333,4333,4331,4331,4328,4328,4327,4327,4318,4318,4314,4314,4309,4309,4308,4308,4306,4306,4305,4305,4302,4302,4301,4301,4298,4298,4297,4297,4288,4288,4287,4287,4286,4286,4285,4285,4284,4284,4282,4282,4280,4280,4279,4279,4278,4278,4277,4277,4276,4276,4275,4275,4274,4274,4271,4271,4270,4270,4268,4268,4266,4266,4262,4262,4260,4260,4259,4259,4257,4257,4256,4256,4255,4255,4254,4254,4253,4253,4250,4250,4248,4248,4245,4245,4244,4244,4243,4243,4241,4241,4239,4239,4238,4238,4237,4237,4235,4235,4232,4232,4230,4230,4229,4229,4227,4227,4222,4222,4221,4221,4217,4217,4213,4213,4207,4207,4203,4203,4202,4202,4200,4200,4198,4198,4194,4194,4193,4193,4192,4192,4191,4191,4189,4189,4188,4188,4186,4186,4183,4183,4181,4181,4179,4179,4178,4178,4177,4177,4176,4176,4173,4173,4172,4172,4171,4171,4170,4170,4166,4166,4164,4164,4160,4160,4157,4157,4156,4156,4155,4155,4154,4154,4152,4152,4150,4150,4144,4144,4142,4142,4140,4140,4138,4138,4137,4137,4135,4135,4131,4131,4130,4130,4128,4128,4126,4126,4125,4125,4121,4121,4119,4119,4117,4117,4116,4116,4115,4115,4114,4114,4112,4112,4111,4111,4110,4110,4109,4109,4108,4108,4107,4107,4106,4106,4103,4103,4102,4102,4101,4101,4099,4099,4098,4098,4097,4097,4096,4096,4095,4095,4094,4094,4093,4093,4092,4092,4087,4087,4085,4085,4084,4084,4083,4083,4082,4082,4079,4079,4077,4077,4076,4076,4074,4074,4073,4073,4071,4071,4070,4070,4066,4066,4065,4065,4064,4064,4062,4062,4060,4060,4059,4059,4056,4056,4055,4055,4053,4053,4052,4052,4051,4051,4050,4050,4047,4047,4046,4046,4044,4044,4040,4040,4033,4033,4030,4030,4028,4028,4027,4027,4025,4025,4023,4023,4021,4021,4020,4020,4018,4018,4016,4016,4015,4015,4014,4014,4013,4013,4010,4010,4009,4009,4004,4004,4002,4002,3997,3997,3996,3996,3995,3995,3994,3994,3992,3992,3988,3988,3984,3984,3982,3982,3978,3978,3977,3977,3975,3975,3974,3974,3972,3972,3971,3971,3970,3970,3969,3969,3968,3968,3967,3967,3966,3966,3965,3965,3964,3964,3960,3960,3958,3958,3953,3953,3951,3951,3950,3950,3946,3946,3943,3943,3940,3940,3938,3938,3936,3936,3935,3935,3934,3934,3933,3933,3932,3932,3931,3931,3930,3930,3929,3929,3928,3928,3926,3926,3924,3924,3923,3923,3922,3922,3921,3921,3920,3920,3919,3919,3913,3913,3912,3912,3911,3911,3910,3910,3907,3907,3906,3906,3905,3905,3904,3904,3903,3903,3901,3901,3900,3900,3899,3899,3898,3898,3896,3896,3894,3894,3892,3892,3891,3891,3887,3887,3886,3886,3885,3885,3882,3882,3881,3881,3879,3879,3878,3878,3877,3877,3874,3874,3873,3873,3867,3867,3865,3865,3863,3863,3861,3861,3859,3859,3856,3856,3855,3855,3854,3854,3852,3852,3850,3850,3847,3847,3845,3845,3844,3844,3843,3843,3842,3842,3840,3840,3839,3839,3838,3838,3837,3837,3836,3836,3831,3831,3830,3830,3828,3828,3827,3827,3826,3826,3825,3825,3824,3824,3821,3821,3818,3818,3815,3815,3813,3813,3811,3811,3810,3810,3806,3806,3805,3805,3804,3804,3801,3801,3799,3799,3797,3797,3790,3790,3789,3789,3787,3787,3786,3786,3784,3784,3780,3780,3779,3779,3777,3777,3774,3774,3773,3773,3772,3772,3771,3771,3770,3770,3768,3768,3765,3765,3763,3763,3762,3762,3759,3759,3756,3756,3755,3755,3753,3753,3752,3752,3751,3751,3750,3750,3749,3749,3745,3745,3744,3744,3743,3743,3741,3741,3740,3740,3739,3739,3736,3736,3735,3735,3733,3733,3732,3732,3730,3730,3729,3729,3728,3728,3725,3725,3724,3724,3723,3723,3722,3722,3720,3720,3718,3718,3717,3717,3714,3714,3713,3713,3710,3710,3708,3708,3707,3707,3706,3706,3705,3705,3704,3704,3703,3703,3702,3702,3700,3700,3699,3699,3698,3698,3696,3696,3694,3694,3693,3693,3692,3692,3691,3691,3690,3690,3688,3688,3684,3684,3683,3683,3682,3682,3681,3681,3680,3680,3673,3673,3672,3672,3669,3669,3667,3667,3666,3666,3665,3665,3663,3663,3662,3662,3661,3661,3657,3657,3655,3655,3653,3653,3652,3652,3650,3650,3648,3648,3647,3647,3644,3644,3643,3643,3642,3642,3641,3641,3640,3640,3639,3639,3637,3637,3636,3636,3635,3635,3634,3634,3633,3633,3632,3632,3630,3630,3629,3629,3625,3625,3624,3624,3622,3622,3619,3619,3618,3618,3614,3614,3611,3611,3609,3609,3607,3607,3606,3606,3604,3604,3603,3603,3602,3602,3601,3601,3600,3600,3599,3599,3595,3595,3594,3594,3593,3593,3592,3592,3590,3590,3589,3589,3588,3588,3586,3586,3585,3585,3583,3583,3581,3581,3578,3578,3576,3576,3574,3574,3573,3573,3571,3571,3570,3570,3568,3568,3567,3567,3566,3566,3564,3564,3561,3561,3559,3559,3557,3557,3556,3556,3555,3555,3554,3554,3553,3553,3552,3552,3551,3551,3550,3550,3547,3547,3546,3546,3545,3545,3544,3544,3543,3543,3542,3542,3541,3541,3540,3540,3538,3538,3536,3536,3530,3530,3528,3528,3527,3527,3526,3526,3525,3525,3524,3524,3521,3521,3518,3518,3517,3517,3516,3516,3515,3515,3509,3509,3508,3508,3507,3507,3505,3505,3504,3504,3503,3503,3502,3502,3501,3501,3500,3500,3499,3499,3497,3497,3494,3494,3493,3493,3492,3492,3490,3490,3489,3489,3488,3488,3484,3484,3481,3481,3479,3479,3478,3478,3477,3477,3474,3474,3473,3473,3472,3472,3471,3471,3470,3470,3469,3469,3468,3468,3467,3467,3465,3465,3464,3464,3463,3463,3461,3461,3459,3459,3458,3458,3456,3456,3455,3455,3454,3454,3453,3453,3452,3452,3450,3450,3449,3449,3445,3445,3442,3442,3440,3440,3439,3439,3437,3437,3436,3436,3435,3435,3433,3433,3432,3432,3430,3430,3429,3429,3428,3428,3426,3426,3423,3423,3422,3422,3421,3421,3420,3420,3419,3419,3418,3418,3415,3415,3414,3414,3413,3413,3411,3411,3410,3410,3407,3407,3406,3406,3405,3405,3403,3403,3402,3402,3401,3401,3399,3399,3397,3397,3396,3396,3394,3394,3393,3393,3392,3392,3391,3391,3390,3390,3389,3389,3387,3387,3386,3386,3385,3385,3384,3384,3381,3381,3377,3377,3376,3376,3375,3375,3374,3374,3373,3373,3371,3371,3370,3370,3369,3369,3368,3368,3366,3366,3365,3365,3364,3364,3363,3363,3362,3362,3361,3361,3360,3360,3359,3359,3356,3356,3355,3355,3354,3354,3352,3352,3350,3350,3349,3349,3348,3348,3346,3346,3345,3345,3344,3344,3341,3341,3340,3340,3339,3339,3338,3338,3337,3337,3335,3335,3334,3334,3333,3333,3332,3332,3331,3331,3330,3330,3329,3329,3326,3326,3325,3325,3324,3324,3321,3321,3319,3319,3318,3318,3317,3317,3316,3316,3315,3315,3314,3314,3313,3313,3312,3312,3311,3311,3310,3310,3309,3309,3308,3308,3307,3307,3306,3306,3305,3305,3304,3304,3303,3303,3302,3302,3301,3301,3300,3300,3297,3297,3296,3296,3294,3294,3293,3293,3292,3292,3290,3290,3288,3288,3285,3285,3284,3284,3283,3283,3282,3282,3281,3281,3278,3278,3277,3277,3276,3276,3275,3275,3274,3274,3273,3273,3272,3272,3271,3271,3270,3270,3267,3267,3266,3266,3265,3265,3263,3263,3262,3262,3261,3261,3260,3260,3258,3258,3257,3257,3255,3255,3254,3254,3252,3252,3251,3251,3250,3250,3249,3249,3247,3247,3246,3246,3244,3244,3243,3243,3242,3242,3241,3241,3239,3239,3238,3238,3237,3237,3236,3236,3235,3235,3234,3234,3231,3231,3230,3230,3228,3228,3227,3227,3226,3226,3225,3225,3224,3224,3223,3223,3221,3221,3220,3220,3218,3218,3217,3217,3216,3216,3215,3215,3211,3211,3208,3208,3207,3207,3206,3206,3205,3205,3203,3203,3201,3201,3200,3200,3199,3199,3198,3198,3197,3197,3196,3196,3195,3195,3194,3194,3193,3193,3192,3192,3190,3190,3188,3188,3183,3183,3182,3182,3181,3181,3179,3179,3178,3178,3177,3177,3175,3175,3173,3173,3172,3172,3171,3171,3170,3170,3169,3169,3167,3167,3166,3166,3164,3164,3163,3163,3162,3162,3160,3160,3159,3159,3158,3158,3157,3157,3156,3156,3155,3155,3153,3153,3152,3152,3151,3151,3150,3150,3149,3149,3148,3148,3146,3146,3145,3145,3144,3144,3143,3143,3142,3142,3141,3141,3139,3139,3137,3137,3135,3135,3134,3134,3132,3132,3131,3131,3130,3130,3129,3129,3128,3128,3126,3126,3125,3125,3124,3124,3123,3123,3122,3122,3121,3121,3120,3120,3119,3119,3117,3117,3116,3116,3115,3115,3114,3114,3113,3113,3112,3112,3111,3111,3110,3110,3109,3109,3108,3108,3107,3107,3106,3106,3105,3105,3104,3104,3103,3103,3102,3102,3101,3101,3100,3100,3098,3098,3097,3097,3095,3095,3094,3094,3092,3092,3091,3091,3089,3089,3088,3088,3087,3087,3085,3085,3084,3084,3083,3083,3082,3082,3081,3081,3080,3080,3079,3079,3078,3078,3077,3077,3076,3076,3075,3075,3074,3074,3071,3071,3070,3070,3069,3069,3068,3068,3067,3067,3066,3066,3065,3065,3064,3064,3063,3063,3062,3062,3061,3061,3060,3060,3058,3058,3057,3057,3056,3056,3055,3055,3054,3054,3052,3052,3051,3051,3050,3050,3049,3049,3048,3048,3047,3047,3046,3046,3045,3045,3044,3044,3043,3043,3042,3042,3040,3040,3039,3039,3038,3038,3037,3037,3036,3036,3035,3035,3034,3034,3033,3033,3032,3032,3031,3031,3030,3030,3029,3029,3028,3028,3026,3026,3025,3025,3024,3024,3023,3023,3022,3022,3021,3021,3020,3020,3019,3019,3018,3018,3017,3017,3016,3016,3015,3015,3014,3014,3013,3013,3012,3012,3011,3011,3010,3010,3009,3009,3008,3008,3006,3006,3005,3005,3004,3004,3003,3003,3002,3002,3000,3000,2999,2999,2998,2998,2997,2997,2996,2996,2994,2994,2993,2993,2992,2992,2991,2991,2990,2990,2989,2989,2988,2988,2987,2987,2986,2986,2985,2985,2984,2984,2983,2983,2982,2982,2981,2981,2979,2979,2978,2978,2977,2977,2976,2976,2975,2975,2973,2973,2972,2972,2970,2970,2969,2969,2968,2968,2967,2967,2966,2966,2965,2965,2963,2963,2962,2962,2957,2957,2956,2956,2954,2954,2953,2953,2952,2952,2951,2951,2949,2949,2948,2948,2947,2947,2945,2945,2942,2942,2941,2941,2939,2939,2938,2938,2937,2937,2936,2936,2935,2935,2934,2934,2933,2933,2932,2932,2931,2931,2930,2930,2929,2929,2927,2927,2926,2926,2925,2925,2924,2924,2923,2923,2922,2922,2921,2921,2920,2920,2918,2918,2917,2917,2915,2915,2913,2913,2912,2912,2911,2911,2910,2910,2907,2907,2906,2906,2905,2905,2904,2904,2903,2903,2902,2902,2901,2901,2900,2900,2899,2899,2898,2898,2897,2897,2896,2896,2895,2895,2894,2894,2892,2892,2889,2889,2888,2888,2887,2887,2886,2886,2885,2885,2884,2884,2883,2883,2882,2882,2881,2881,2880,2880,2879,2879,2877,2877,2876,2876,2875,2875,2874,2874,2873,2873,2872,2872,2871,2871,2870,2870,2869,2869,2868,2868,2867,2867,2866,2866,2865,2865,2864,2864,2863,2863,2862,2862,2861,2861,2860,2860,2859,2859,2858,2858,2857,2857,2856,2856,2855,2855,2854,2854,2853,2853,2851,2851,2850,2850,2849,2849,2848,2848,2847,2847,2846,2846,2845,2845,2844,2844,2842,2842,2841,2841,2840,2840,2839,2839,2838,2838,2837,2837,2835,2835,2834,2834,2833,2833,2832,2832,2831,2831,2830,2830,2829,2829,2827,2827,2826,2826,2825,2825,2822,2822,2821,2821,2820,2820,2817,2817,2816,2816,2815,2815,2814,2814,2813,2813,2812,2812,2808,2808,2807,2807,2806,2806,2805,2805,2804,2804,2803,2803,2802,2802,2801,2801,2800,2800,2799,2799,2797,2797,2796,2796,2795,2795,2794,2794,2793,2793,2792,2792,2791,2791,2790,2790,2788,2788,2787,2787,2786,2786,2785,2785,2784,2784,2783,2783,2782,2782,2781,2781,2779,2779,2778,2778,2777,2777,2776,2776,2775,2775,2774,2774,2773,2773,2772,2772,2771,2771,2770,2770,2769,2769,2767,2767,2766,2766,2765,2765,2764,2764,2763,2763,2762,2762,2761,2761,2760,2760,2759,2759,2758,2758,2757,2757,2756,2756,2755,2755,2753,2753,2751,2751,2750,2750,2749,2749,2748,2748,2747,2747,2746,2746,2745,2745,2744,2744,2743,2743,2742,2742,2741,2741,2740,2740,2739,2739,2738,2738,2737,2737,2736,2736,2735,2735,2734,2734,2732,2732,2731,2731,2730,2730,2729,2729,2728,2728,2727,2727,2725,2725,2724,2724,2723,2723,2722,2722,2721,2721,2720,2720,2719,2719,2718,2718,2717,2717,2716,2716,2715,2715,2714,2714,2713,2713,2712,2712,2711,2711,2710,2710,2709,2709,2708,2708,2707,2707,2705,2705,2704,2704,2703,2703,2702,2702,2701,2701,2700,2700,2699,2699,2698,2698,2697,2697,2695,2695,2694,2694,2693,2693,2690,2690,2689,2689,2688,2688,2687,2687,2686,2686,2685,2685,2684,2684,2683,2683,2682,2682,2681,2681,2680,2680,2679,2679,2677,2677,2676,2676,2675,2675,2674,2674,2673,2673,2671,2671,2670,2670,2669,2669,2668,2668,2667,2667,2666,2666,2665,2665,2663,2663,2662,2662,2661,2661,2660,2660,2659,2659,2658,2658,2657,2657,2656,2656,2655,2655,2654,2654,2653,2653,2652,2652,2651,2651,2650,2650,2649,2649,2648,2648,2647,2647,2646,2646,2645,2645,2643,2643,2642,2642,2641,2641,2640,2640,2639,2639,2638,2638,2637,2637,2635,2635,2634,2634,2633,2633,2632,2632,2631,2631,2630,2630,2629,2629,2628,2628,2627,2627,2626,2626,2625,2625,2624,2624,2623,2623,2622,2622,2621,2621,2620,2620,2619,2619,2618,2618,2617,2617,2614,2614,2613,2613,2612,2612,2611,2611,2610,2610,2608,2608,2607,2607,2606,2606,2605,2605,2604,2604,2603,2603,2602,2602,2601,2601,2600,2600,2599,2599,2598,2598,2597,2597,2596,2596,2595,2595,2594,2594,2593,2593,2592,2592,2591,2591,2590,2590,2589,2589,2588,2588,2587,2587,2586,2586,2585,2585,2584,2584,2583,2583,2581,2581,2580,2580,2579,2579,2578,2578,2577,2577,2576,2576,2575,2575,2574,2574,2573,2573,2572,2572,2571,2571,2570,2570,2569,2569,2568,2568,2567,2567,2566,2566,2565,2565,2564,2564,2563,2563,2562,2562,2561,2561,2560,2560,2559,2559,2558,2558,2557,2557,2556,2556,2555,2555,2554,2554,2553,2553,2552,2552,2551,2551,2550,2550,2549,2549,2548,2548,2547,2547,2546,2546,2545,2545,2544,2544,2543,2543,2542,2542,2541,2541,2540,2540,2539,2539,2538,2538,2537,2537,2536,2536,2535,2535,2534,2534,2533,2533,2532,2532,2531,2531,2530,2530,2529,2529,2528,2528,2527,2527,2526,2526,2525,2525,2524,2524,2522,2522,2521,2521,2520,2520,2519,2519,2518,2518,2517,2517,2516,2516,2515,2515,2514,2514,2513,2513,2512,2512,2511,2511,2510,2510,2509,2509,2508,2508,2507,2507,2506,2506,2505,2505,2504,2504,2503,2503,2502,2502,2501,2501,2500,2500,2499,2499,2498,2498,2497,2497,2496,2496,2495,2495,2494,2494,2493,2493,2492,2492,2490,2490,2489,2489,2488,2488,2487,2487,2486,2486,2485,2485,2484,2484,2483,2483,2481,2481,2479,2479,2478,2478,2477,2477,2476,2476,2475,2475,2474,2474,2473,2473,2472,2472,2471,2471,2470,2470,2469,2469,2468,2468,2467,2467,2465,2465,2464,2464,2463,2463,2462,2462,2460,2460,2459,2459,2458,2458,2457,2457,2456,2456,2455,2455,2454,2454,2453,2453,2452,2452,2451,2451,2450,2450,2449,2449,2448,2448,2447,2447,2446,2446,2445,2445,2443,2443,2442,2442,2441,2441,2440,2440,2439,2439,2438,2438,2437,2437,2436,2436,2435,2435,2434,2434,2433,2433,2431,2431,2430,2430,2429,2429,2428,2428,2427,2427,2425,2425,2424,2424,2423,2423,2422,2422,2421,2421,2420,2420,2419,2419,2418,2418,2417,2417,2416,2416,2415,2415,2414,2414,2413,2413,2412,2412,2411,2411,2410,2410,2409,2409,2408,2408,2407,2407,2405,2405,2404,2404,2403,2403,2402,2402,2401,2401,2400,2400,2399,2399,2398,2398,2397,2397,2396,2396,2395,2395,2394,2394,2393,2393,2392,2392,2391,2391,2390,2390,2389,2389,2388,2388,2387,2387,2386,2386,2385,2385,2384,2384,2383,2383,2382,2382,2381,2381,2380,2380,2379,2379,2378,2378,2377,2377,2376,2376,2375,2375,2374,2374,2373,2373,2372,2372,2371,2371,2370,2370,2369,2369,2367,2367,2366,2366,2365,2365,2364,2364,2363,2363,2362,2362,2361,2361,2360,2360,2359,2359,2358,2358,2357,2357,2356,2356,2355,2355,2354,2354,2353,2353,2352,2352,2351,2351,2350,2350,2349,2349,2348,2348,2347,2347,2346,2346,2345,2345,2344,2344,2343,2343,2342,2342,2341,2341,2340,2340,2339,2339,2338,2338,2337,2337,2336,2336,2335,2335,2334,2334,2333,2333,2332,2332,2331,2331,2330,2330,2329,2329,2328,2328,2327,2327,2326,2326,2325,2325,2324,2324,2323,2323,2322,2322,2321,2321,2320,2320,2319,2319,2318,2318,2317,2317,2316,2316,2315,2315,2314,2314,2313,2313,2312,2312,2310,2310,2309,2309,2308,2308,2307,2307,2306,2306,2305,2305,2304,2304,2303,2303,2302,2302,2301,2301,2300,2300,2299,2299,2298,2298,2297,2297,2296,2296,2295,2295,2294,2294,2293,2293,2292,2292,2291,2291,2290,2290,2289,2289,2288,2288,2286,2286,2285,2285,2284,2284,2283,2283,2282,2282,2281,2281,2280,2280,2279,2279,2278,2278,2277,2277,2276,2276,2275,2275,2274,2274,2273,2273,2272,2272,2271,2271,2270,2270,2269,2269,2268,2268,2267,2267,2266,2266,2265,2265,2264,2264,2263,2263,2262,2262,2261,2261,2260,2260,2259,2259,2258,2258,2257,2257,2256,2256,2255,2255,2254,2254,2253,2253,2252,2252,2251,2251,2250,2250,2249,2249,2248,2248,2247,2247,2246,2246,2245,2245,2244,2244,2243,2243,2242,2242,2241,2241,2240,2240,2239,2239,2238,2238,2237,2237,2236,2236,2235,2235,2234,2234,2233,2233,2232,2232,2231,2231,2230,2230,2229,2229,2228,2228,2227,2227,2226,2226,2225,2225,2224,2224,2223,2223,2222,2222,2221,2221,2220,2220,2219,2219,2218,2218,2217,2217,2216,2216,2215,2215,2214,2214,2213,2213,2212,2212,2211,2211,2210,2210,2209,2209,2208,2208,2207,2207,2206,2206,2205,2205,2204,2204,2203,2203,2202,2202,2201,2201,2200,2200,2199,2199,2198,2198,2197,2197,2196,2196,2195,2195,2194,2194,2193,2193,2192,2192,2191,2191,2190,2190,2189,2189,2188,2188,2187,2187,2186,2186,2185,2185,2184,2184,2183,2183,2182,2182,2181,2181,2178,2178,2177,2177,2176,2176,2175,2175,2174,2174,2173,2173,2172,2172,2171,2171,2170,2170,2169,2169,2168,2168,2167,2167,2166,2166,2165,2165,2164,2164,2163,2163,2162,2162,2161,2161,2160,2160,2159,2159,2158,2158,2157,2157,2156,2156,2155,2155,2154,2154,2153,2153,2152,2152,2151,2151,2150,2150,2149,2149,2148,2148,2147,2147,2146,2146,2145,2145,2144,2144,2143,2143,2142,2142,2141,2141,2140,2140,2139,2139,2138,2138,2137,2137,2136,2136,2135,2135,2134,2134,2133,2133,2132,2132,2131,2131,2130,2130,2129,2129,2128,2128,2127,2127,2126,2126,2125,2125,2124,2124,2123,2123,2122,2122,2121,2121,2120,2120,2119,2119,2118,2118,2117,2117,2116,2116,2115,2115,2114,2114,2113,2113,2112,2112,2111,2111,2110,2110,2109,2109,2108,2108,2107,2107,2106,2106,2105,2105,2104,2104,2103,2103,2102,2102,2101,2101,2100,2100,2099,2099,2098,2098,2097,2097,2096,2096,2095,2095,2094,2094,2093,2093,2092,2092,2091,2091,2090,2090,2089,2089,2088,2088,2087,2087,2086,2086,2085,2085,2084,2084,2083,2083,2082,2082,2081,2081,2080,2080,2079,2079,2078,2078,2077,2077,2076,2076,2075,2075,2074,2074,2073,2073,2072,2072,2071,2071,2070,2070,2069,2069,2068,2068,2067,2067,2066,2066,2065,2065,2064,2064,2063,2063,2062,2062,2061,2061,2060,2060,2059,2059,2058,2058,2057,2057,2056,2056,2055,2055,2054,2054,2053,2053,2052,2052,2051,2051,2050,2050,2049,2049,2048,2048,2047,2047,2046,2046,2045,2045,2044,2044,2043,2043,2042,2042,2041,2041,2040,2040,2039,2039,2038,2038,2037,2037,2036,2036,2035,2035,2034,2034,2033,2033,2032,2032,2031,2031,2030,2030,2029,2029,2028,2028,2027,2027,2026,2026,2025,2025,2024,2024,2023,2023,2022,2022,2021,2021,2020,2020,2019,2019,2018,2018,2017,2017,2016,2016,2015,2015,2014,2014,2013,2013,2012,2012,2011,2011,2010,2010,2009,2009,2008,2008,2007,2007,2006,2006,2005,2005,2004,2004,2003,2003,2002,2002,2001,2001,2000,2000,1999,1999,1998,1998,1997,1997,1996,1996,1995,1995,1994,1994,1993,1993,1992,1992,1991,1991,1990,1990,1989,1989,1988,1988,1987,1987,1986,1986,1985,1985,1984,1984,1983,1983,1982,1982,1981,1981,1980,1980,1979,1979,1978,1978,1977,1977,1976,1976,1975,1975,1974,1974,1973,1973,1972,1972,1971,1971,1970,1970,1969,1969,1968,1968,1967,1967,1966,1966,1965,1965,1964,1964,1963,1963,1962,1962,1961,1961,1960,1960,1959,1959,1958,1958,1957,1957,1956,1956,1955,1955,1954,1954,1953,1953,1952,1952,1951,1951,1950,1950,1949,1949,1948,1948,1947,1947,1946,1946,1945,1945,1944,1944,1943,1943,1942,1942,1941,1941,1940,1940,1939,1939,1938,1938,1937,1937,1936,1936,1935,1935,1934,1934,1933,1933,1932,1932,1931,1931,1930,1930,1929,1929,1928,1928,1927,1927,1926,1926,1925,1925,1924,1924,1923,1923,1922,1922,1921,1921,1920,1920,1919,1919,1918,1918,1917,1917,1916,1916,1915,1915,1914,1914,1913,1913,1912,1912,1911,1911,1910,1910,1909,1909,1908,1908,1907,1907,1906,1906,1905,1905,1904,1904,1903,1903,1902,1902,1901,1901,1900,1900,1899,1899,1898,1898,1897,1897,1896,1896,1895,1895,1894,1894,1893,1893,1892,1892,1891,1891,1890,1890,1889,1889,1888,1888,1887,1887,1886,1886,1885,1885,1884,1884,1883,1883,1882,1882,1881,1881,1880,1880,1879,1879,1878,1878,1877,1877,1876,1876,1875,1875,1874,1874,1873,1873,1872,1872,1871,1871,1870,1870,1869,1869,1868,1868,1867,1867,1866,1866,1865,1865,1864,1864,1863,1863,1862,1862,1861,1861,1860,1860,1859,1859,1858,1858,1857,1857,1856,1856,1855,1855,1854,1854,1853,1853,1852,1852,1851,1851,1850,1850,1849,1849,1848,1848,1847,1847,1846,1846,1845,1845,1844,1844,1843,1843,1842,1842,1841,1841,1840,1840,1839,1839,1838,1838,1837,1837,1836,1836,1835,1835,1834,1834,1833,1833,1832,1832,1831,1831,1830,1830,1829,1829,1828,1828,1827,1827,1826,1826,1825,1825,1824,1824,1823,1823,1822,1822,1821,1821,1820,1820,1819,1819,1818,1818,1817,1817,1816,1816,1815,1815,1814,1814,1813,1813,1812,1812,1811,1811,1810,1810,1809,1809,1808,1808,1807,1807,1806,1806,1805,1805,1804,1804,1803,1803,1802,1802,1801,1801,1800,1800,1799,1799,1798,1798,1797,1797,1796,1796,1795,1795,1794,1794,1793,1793,1792,1792,1791,1791,1790,1790,1789,1789,1788,1788,1787,1787,1786,1786,1785,1785,1784,1784,1783,1783,1782,1782,1781,1781,1780,1780,1779,1779,1778,1778,1777,1777,1776,1776,1775,1775,1774,1774,1773,1773,1772,1772,1771,1771,1770,1770,1769,1769,1768,1768,1767,1767,1766,1766,1765,1765,1764,1764,1763,1763,1762,1762,1761,1761,1760,1760,1759,1759,1758,1758,1757,1757,1756,1756,1755,1755,1754,1754,1753,1753,1752,1752,1751,1751,1750,1750,1749,1749,1748,1748,1747,1747,1746,1746,1745,1745,1744,1744,1743,1743,1742,1742,1741,1741,1740,1740,1739,1739,1738,1738,1737,1737,1736,1736,1735,1735,1734,1734,1733,1733,1732,1732,1731,1731,1730,1730,1729,1729,1728,1728,1727,1727,1726,1726,1725,1725,1724,1724,1723,1723,1722,1722,1721,1721,1720,1720,1719,1719,1718,1718,1717,1717,1716,1716,1715,1715,1714,1714,1713,1713,1712,1712,1711,1711,1710,1710,1709,1709,1708,1708,1707,1707,1706,1706,1705,1705,1704,1704,1703,1703,1702,1702,1701,1701,1700,1700,1699,1699,1698,1698,1697,1697,1696,1696,1695,1695,1694,1694,1693,1693,1692,1692,1691,1691,1690,1690,1689,1689,1688,1688,1687,1687,1686,1686,1685,1685,1684,1684,1683,1683,1682,1682,1681,1681,1680,1680,1679,1679,1678,1678,1677,1677,1676,1676,1675,1675,1674,1674,1673,1673,1672,1672,1671,1671,1670,1670,1669,1669,1668,1668,1667,1667,1666,1666,1665,1665,1664,1664,1663,1663,1662,1662,1661,1661,1660,1660,1659,1659,1658,1658,1657,1657,1656,1656,1655,1655,1654,1654,1653,1653,1652,1652,1651,1651,1650,1650,1649,1649,1648,1648,1647,1647,1646,1646,1645,1645,1644,1644,1643,1643,1642,1642,1641,1641,1640,1640,1639,1639,1638,1638,1637,1637,1636,1636,1635,1635,1634,1634,1633,1633,1632,1632,1631,1631,1630,1630,1629,1629,1628,1628,1627,1627,1626,1626,1625,1625,1624,1624,1623,1623,1622,1622,1621,1621,1620,1620,1619,1619,1618,1618,1617,1617,1616,1616,1615,1615,1614,1614,1613,1613,1612,1612,1611,1611,1610,1610,1609,1609,1608,1608,1607,1607,1606,1606,1605,1605,1604,1604,1603,1603,1602,1602,1601,1601,1600,1600,1599,1599,1598,1598,1597,1597,1596,1596,1595,1595,1594,1594,1593,1593,1592,1592,1591,1591,1590,1590,1589,1589,1588,1588,1587,1587,1586,1586,1585,1585,1584,1584,1583,1583,1582,1582,1581,1581,1580,1580,1579,1579,1578,1578,1577,1577,1576,1576,1575,1575,1574,1574,1573,1573,1572,1572,1571,1571,1570,1570,1569,1569,1568,1568,1567,1567,1566,1566,1565,1565,1564,1564,1563,1563,1562,1562,1561,1561,1560,1560,1559,1559,1558,1558,1557,1557,1556,1556,1555,1555,1554,1554,1553,1553,1552,1552,1551,1551,1550,1550,1549,1549,1548,1548,1547,1547,1546,1546,1545,1545,1544,1544,1543,1543,1542,1542,1541,1541,1540,1540,1539,1539,1538,1538,1537,1537,1536,1536,1535,1535,1534,1534,1533,1533,1532,1532,1531,1531,1530,1530,1529,1529,1528,1528,1527,1527,1526,1526,1525,1525,1524,1524,1523,1523,1522,1522,1521,1521,1520,1520,1519,1519,1518,1518,1517,1517,1516,1516,1515,1515,1514,1514,1513,1513,1512,1512,1511,1511,1510,1510,1509,1509,1508,1508,1507,1507,1506,1506,1505,1505,1504,1504,1503,1503,1502,1502,1501,1501,1500,1500,1499,1499,1498,1498,1497,1497,1496,1496,1495,1495,1494,1494,1493,1493,1492,1492,1491,1491,1490,1490,1489,1489,1488,1488,1487,1487,1486,1486,1485,1485,1484,1484,1483,1483,1482,1482,1481,1481,1480,1480,1479,1479,1478,1478,1477,1477,1476,1476,1475,1475,1474,1474,1473,1473,1472,1472,1471,1471,1470,1470,1469,1469,1468,1468,1467,1467,1466,1466,1465,1465,1464,1464,1463,1463,1462,1462,1461,1461,1460,1460,1459,1459,1458,1458,1457,1457,1456,1456,1455,1455,1454,1454,1453,1453,1452,1452,1451,1451,1450,1450,1449,1449,1448,1448,1447,1447,1446,1446,1445,1445,1444,1444,1443,1443,1442,1442,1441,1441,1440,1440,1439,1439,1438,1438,1437,1437,1436,1436,1435,1435,1434,1434,1433,1433,1432,1432,1431,1431,1430,1430,1429,1429,1428,1428,1427,1427,1426,1426,1425,1425,1424,1424,1423,1423,1422,1422,1421,1421,1420,1420,1419,1419,1418,1418,1417,1417,1416,1416,1415,1415,1414,1414,1413,1413,1412,1412,1411,1411,1410,1410,1409,1409,1408,1408,1407,1407,1406,1406,1405,1405,1404,1404,1403,1403,1402,1402,1401,1401,1400,1400,1399,1399,1398,1398,1397,1397,1396,1396,1395,1395,1394,1394,1393,1393,1392,1392,1391,1391,1390,1390,1389,1389,1388,1388,1387,1387,1386,1386,1385,1385,1384,1384,1383,1383,1382,1382,1381,1381,1380,1380,1379,1379,1378,1378,1377,1377,1376,1376,1375,1375,1374,1374,1373,1373,1372,1372,1371,1371,1370,1370,1369,1369,1368,1368,1367,1367,1366,1366,1365,1365,1364,1364,1363,1363,1362,1362,1361,1361,1360,1360,1359,1359,1358,1358,1357,1357,1356,1356,1355,1355,1354,1354,1353,1353,1352,1352,1351,1351,1350,1350,1349,1349,1348,1348,1347,1347,1346,1346,1345,1345,1344,1344,1343,1343,1342,1342,1341,1341,1340,1340,1339,1339,1338,1338,1337,1337,1336,1336,1335,1335,1334,1334,1333,1333,1332,1332,1331,1331,1330,1330,1329,1329,1328,1328,1327,1327,1326,1326,1325,1325,1324,1324,1323,1323,1322,1322,1321,1321,1320,1320,1319,1319,1318,1318,1317,1317,1316,1316,1315,1315,1314,1314,1313,1313,1312,1312,1311,1311,1310,1310,1309,1309,1308,1308,1307,1307,1306,1306,1305,1305,1304,1304,1303,1303,1302,1302,1301,1301,1300,1300,1299,1299,1298,1298,1297,1297,1296,1296,1295,1295,1294,1294,1293,1293,1292,1292,1291,1291,1290,1290,1289,1289,1288,1288,1287,1287,1286,1286,1285,1285,1284,1284,1283,1283,1282,1282,1281,1281,1280,1280,1279,1279,1278,1278,1277,1277,1276,1276,1275,1275,1274,1274,1273,1273,1272,1272,1271,1271,1270,1270,1269,1269,1268,1268,1267,1267,1266,1266,1265,1265,1264,1264,1263,1263,1262,1262,1261,1261,1260,1260,1259,1259,1258,1258,1257,1257,1256,1256,1255,1255,1254,1254,1253,1253,1252,1252,1251,1251,1250,1250,1249,1249,1248,1248,1247,1247,1246,1246,1245,1245,1244,1244,1243,1243,1242,1242,1241,1241,1240,1240,1239,1239,1238,1238,1237,1237,1236,1236,1235,1235,1234,1234,1233,1233,1232,1232,1231,1231,1230,1230,1229,1229,1228,1228,1227,1227,1226,1226,1225,1225,1224,1224,1223,1223,1222,1222,1221,1221,1220,1220,1219,1219,1218,1218,1217,1217,1216,1216,1215,1215,1214,1214,1213,1213,1212,1212,1211,1211,1210,1210,1209,1209,1208,1208,1207,1207,1206,1206,1205,1205,1204,1204,1203,1203,1202,1202,1201,1201,1200,1200,1199,1199,1198,1198,1197,1197,1196,1196,1195,1195,1194,1194,1193,1193,1192,1192,1191,1191,1190,1190,1189,1189,1188,1188,1187,1187,1186,1186,1185,1185,1184,1184,1183,1183,1182,1182,1181,1181,1180,1180,1179,1179,1178,1178,1177,1177,1176,1176,1175,1175,1174,1174,1173,1173,1172,1172,1171,1171,1170,1170,1169,1169,1168,1168,1167,1167,1166,1166,1165,1165,1164,1164,1163,1163,1162,1162,1161,1161,1160,1160,1159,1159,1158,1158,1157,1157,1156,1156,1155,1155,1154,1154,1153,1153,1152,1152,1151,1151,1150,1150,1149,1149,1148,1148,1147,1147,1146,1146,1145,1145,1144,1144,1143,1143,1142,1142,1141,1141,1140,1140,1139,1139,1138,1138,1137,1137,1136,1136,1135,1135,1134,1134,1133,1133,1132,1132,1131,1131,1130,1130,1129,1129,1128,1128,1127,1127,1126,1126,1125,1125,1124,1124,1123,1123,1122,1122,1121,1121,1120,1120,1119,1119,1118,1118,1117,1117,1116,1116,1115,1115,1114,1114,1113,1113,1112,1112,1111,1111,1110,1110,1109,1109,1108,1108,1107,1107,1106,1106,1105,1105,1104,1104,1103,1103,1102,1102,1101,1101,1100,1100,1099,1099,1098,1098,1097,1097,1096,1096,1095,1095,1094,1094,1093,1093,1092,1092,1091,1091,1090,1090,1089,1089,1088,1088,1087,1087,1086,1086,1085,1085,1084,1084,1083,1083,1082,1082,1081,1081,1080,1080,1079,1079,1078,1078,1077,1077,1076,1076,1075,1075,1074,1074,1073,1073,1072,1072,1071,1071,1070,1070,1069,1069,1068,1068,1067,1067,1066,1066,1065,1065,1064,1064,1063,1063,1062,1062,1061,1061,1060,1060,1059,1059,1058,1058,1057,1057,1056,1056,1055,1055,1054,1054,1053,1053,1052,1052,1051,1051,1050,1050,1049,1049,1048,1048,1047,1047,1046,1046,1045,1045,1044,1044,1043,1043,1042,1042,1041,1041,1040,1040,1039,1039,1038,1038,1037,1037,1036,1036,1035,1035,1034,1034,1033,1033,1032,1032,1031,1031,1030,1030,1029,1029,1028,1028,1027,1027,1026,1026,1025,1025,1024,1024,1023,1023,1022,1022,1021,1021,1020,1020,1019,1019,1018,1018,1017,1017,1016,1016,1015,1015,1014,1014,1013,1013,1012,1012,1011,1011,1010,1010,1009,1009,1008,1008,1007,1007,1006,1006,1005,1005,1004,1004,1003,1003,1002,1002,1001,1001,1000,1000,999,999,998,998,997,997,996,996,995,995,994,994,993,993,992,992,991,991,990,990,989,989,988,988,987,987,986,986,985,985,984,984,983,983,982,982,981,981,980,980,979,979,978,978,977,977,976,976,975,975,974,974,973,973,972,972,971,971,970,970,969,969,968,968,967,967,966,966,965,965,964,964,963,963,962,962,961,961,960,960,959,959,958,958,957,957,956,956,955,955,954,954,953,953,952,952,951,951,950,950,949,949,948,948,947,947,946,946,945,945,944,944,943,943,942,942,941,941,940,940,939,939,938,938,937,937,936,936,935,935,934,934,933,933,932,932,931,931,930,930,929,929,928,928,927,927,926,926,925,925,924,924,923,923,922,922,921,921,920,920,919,919,918,918,917,917,916,916,915,915,914,914,913,913,912,912,911,911,910,910,909,909,908,908,907,907,906,906,905,905,904,904,903,903,902,902,901,901,900,900,899,899,898,898,897,897,896,896,895,895,894,894,893,893,892,892,891,891,890,890,889,889,888,888,887,887,886,886,885,885,884,884,883,883,882,882,881,881,880,880,879,879,878,878,877,877,876,876,875,875,874,874,873,873,872,872,871,871,870,870,869,869,868,868,867,867,866,866,865,865,864,864,863,863,862,862,861,861,860,860,859,859,858,858,857,857,856,856,855,855,854,854,853,853,852,852,851,851,850,850,849,849,848,848,847,847,846,846,845,845,844,844,843,843,842,842,841,841,840,840,839,839,838,838,837,837,836,836,835,835,834,834,833,833,832,832,831,831,830,830,829,829,828,828,827,827,826,826,825,825,824,824,823,823,822,822,821,821,820,820,819,819,818,818,817,817,816,816,815,815,814,814,813,813,812,812,811,811,810,810,809,809,808,808,807,807,806,806,805,805,804,804,803,803,802,802,801,801,800,800,799,799,798,798,797,797,796,796,795,795,794,794,793,793,792,792,791,791,790,790,789,789,788,788,787,787,786,786,785,785,784,784,783,783,782,782,781,781,780,780,779,779,778,778,777,777,776,776,775,775,774,774,773,773,772,772,771,771,770,770,769,769,768,768,767,767,766,766,765,765,764,764,763,763,762,762,761,761,760,760,759,759,758,758,757,757,756,756,755,755,754,754,753,753,752,752,751,751,750,750,749,749,748,748,747,747,746,746,745,745,744,744,743,743,742,742,741,741,740,740,739,739,738,738,737,737,736,736,735,735,734,734,733,733,732,732,731,731,730,730,729,729,728,728,727,727,726,726,725,725,724,724,723,723,722,722,721,721,720,720,719,719,718,718,717,717,716,716,715,715,714,714,713,713,712,712,711,711,710,710,709,709,708,708,707,707,706,706,705,705,704,704,703,703,702,702,701,701,700,700,699,699,698,698,697,697,696,696,695,695,694,694,693,693,692,692,691,691,690,690,689,689,688,688,687,687,686,686,685,685,684,684,683,683,682,682,681,681,680,680,679,679,678,678,677,677,676,676,675,675,674,674,673,673,672,672,671,671,670,670,669,669,668,668,667,667,666,666,665,665,664,664,663,663,662,662,661,661,660,660,659,659,658,658,657,657,656,656,655,655,654,654,653,653,652,652,651,651,650,650,649,649,648,648,647,647,646,646,645,645,644,644,643,643,642,642,641,641,640,640,639,639,638,638,637,637,636,636,635,635,634,634,633,633,632,632,631,631,630,630,629,629,628,628,627,627,626,626,625,625,624,624,623,623,622,622,621,621,620,620,619,619,618,618,617,617,616,616,615,615,614,614,613,613,612,612,611,611,610,610,609,609,608,608,607,607,606,606,605,605,604,604,603,603,602,602,601,601,600,600,599,599,598,598,597,597,596,596,595,595,594,594,593,593,592,592,591,591,590,590,589,589,588,588,587,587,586,586,585,585,584,584,583,583,582,582,581,581,580,580,579,579,578,578,577,577,576,576,575,575,574,574,573,573,572,572,571,571,570,570,569,569,568,568,567,567,566,566,565,565,564,564,563,563,562,562,561,561,560,560,559,559,558,558,557,557,556,556,555,555,554,554,553,553,552,552,551,551,550,550,549,549,548,548,547,547,546,546,545,545,544,544,543,543,542,542,541,541,540,540,539,539,538,538,537,537,536,536,535,535,534,534,533,533,532,532,531,531,530,530,529,529,528,528,527,527,526,526,525,525,524,524,523,523,522,522,521,521,520,520,519,519,518,518,517,517,516,516,515,515,514,514,513,513,512,512,511,511,510,510,509,509,508,508,507,507,506,506,505,505,504,504,503,503,502,502,501,501,500,500,499,499,498,498,497,497,496,496,495,495,494,494,493,493,492,492,491,491,490,490,489,489,488,488,487,487,486,486,485,485,484,484,483,483,482,482,481,481,480,480,479,479,478,478,477,477,476,476,475,475,474,474,473,473,472,472,471,471,470,470,469,469,468,468,467,467,466,466,465,465,464,464,463,463,462,462,461,461,460,460,459,459,458,458,457,457,456,456,455,455,454,454,453,453,452,452,451,451,450,450,449,449,448,448,447,447,446,446,445,445,444,444,443,443,442,442,441,441,440,440,439,439,438,438,437,437,436,436,435,435,434,434,433,433,432,432,431,431,430,430,429,429,428,428,427,427,426,426,425,425,424,424,423,423,422,422,421,421,420,420,419,419,418,418,417,417,416,416,415,415,414,414,413,413,412,412,411,411,410,410,409,409,408,408,407,407,406,406,405,405,404,404,403,403,402,402,401,401,400,400,0.0]],"coord\_x":[[0.0,1e-10,0.8595666825705376,0.8595666826705376,1.5178504034722655,1.5178504035722655,2.1584106734582944,2.1584106735582944,2.663857428154537,2.663857428254537,3.1581591473318618,3.1581591474318618,3.617833680747277,3.617833680847277,4.066769157895855,4.066769157995855,4.50698254576246,4.50698254586246,4.939094449573201,4.939094449673201,5.360255256998184,5.360255257098184,5.773792963562431,5.773792963662431,6.181127203720673,6.181127203820673,6.585552356881518,6.585552356981518,6.976986173975763,6.976986174075763,7.3658031056999045,7.3658031057999045,7.754276118694577,7.754276118794577,8.137859279678512,8.137859279778512,8.518419576040262,8.518419576140262,8.8962130074356,8.8962130075356,9.268635617769965,9.268635617869965,9.638430999313792,9.638430999413792,10.00682484738865,10.00682484748865,10.364554628994826,10.364554629094826,10.715372419895186,10.715372419995186,11.064669727991573,11.064669728091573,11.40781787263924,11.40781787273924,11.746301134670636,11.746301134770636,12.081016805808513,12.081016805908513,12.413485368856845,12.413485368956845,12.74465583264056,12.74465583274056,13.075484963549915,13.075484963649915,13.40459450081192,13.40459450091192,13.73293862496172,13.73293862506172,14.059620044276564,14.059620044376564,14.381959812863734,14.381959812963734,14.70397117785209,14.70397117795209,15.024906827115183,15.024906827215183,15.331341000928141,15.331341001028141,15.628316116753117,15.628316116853117,15.91860679712185,15.91860679722185,16.2081087916824,16.208108791782397,16.496545413938126,16.496545414038124,16.78354430075336,16.783544300853357,17.068955472531794,17.068955472631792,17.351597193488704,17.351597193588702,17.633258875359378,17.633258875459376,17.914881769403422,17.91488176950342,18.194399777388906,18.194399777488904,18.471256940467434,18.471256940567432,18.747640892061057,18.747640892161055,19.022866380565933,19.02286638066593,19.297952232894936,19.297952232994934,19.57190289483118,19.571902894931178,19.84486834597097,19.844868346070967,20.117195090898885,20.117195090998884,20.38795222177576,20.387952221875757,20.65506846865945,20.65506846875945,20.919350618343895,20.919350618443893,21.18217693160209,21.182176931702088,21.443159530167712,21.44315953026771,21.699009206342367,21.699009206442366,21.953935732243185,21.953935732343183,22.205935070160844,22.205935070260843,22.45512099772013,22.455120997820128,22.704138844697344,22.704138844797342,22.952693823610083,22.95269382371008,23.200591995325187,23.200591995425185,23.4482574400805,23.448257440180498,23.694650644122284,23.694650644222282,23.94053184885253,23.940531848952528,24.185645054615456,24.185645054715454,24.42905418186169,24.42905418196169,24.67105143221852,24.671051432318517,24.910403352799047,24.910403352899046,25.14809256854462,25.148092568644618,25.384956896510484,25.384956896610483,25.62175399224352,25.621753992343518,25.857043534448128,25.857043534548126,26.09165816846934,26.09165816856934,26.325116925256918,26.325116925356916,26.556918148919756,26.556918149019754,26.78733594009939,26.787335940199387,27.017107267501824,27.017107267601823,27.243868659557616,27.243868659657615,27.46985170922566,27.46985170932566,27.69402466031755,27.694024660417547,27.9173054913969,27.917305491496897,28.140451858010593,28.14045185811059,28.362638872378923,28.36263887247892,28.584256998623317,28.584256998723315,28.804225349308297,28.804225349408295,29.02124582516925,29.021245825269247,29.237984442823343,29.23798444292334,29.454022293742952,29.45402229384295,29.66895081282089,29.668950812920887,29.881934768857004,29.881934768957002,30.094642038396483,30.09464203849648,30.306348582008855,30.306348582108853,30.514253918611296,30.514253918711294,30.72206875028493,30.722068750384928,30.929645683288552,30.92964568338855,31.13585211308451,31.13585211318451,31.342045613604927,31.342045613704926,31.547070307616167,31.547070307716165,31.75191916348001,31.75191916358001,31.95657925192091,31.95657925202091,32.16077388644222,32.16077388654222,32.36337822007163,32.36337822017163,32.56316914334266,32.563169143442664,32.76210414857268,32.762104148672684,32.95992465025081,32.959924650350814,33.15733917267686,33.157339172776865,33.35419515039941,33.354195150499415,33.54950995847712,33.54950995857712,33.74454032249285,33.744540322592854,33.93816915303962,33.93816915313962,34.131679034251384,34.131679034351386,34.32390891718429,34.32390891728429,34.515957790259584,34.515957790359586,34.70660771572102,34.70660771582102,34.896569803723516,34.89656980382352,35.08469076288854,35.084690762988544,35.27139984516417,35.27139984526417,35.45730989821118,35.45730989831118,35.64238730591315,35.64238730601315,35.82656742189237,35.82656742199237,36.01073978030627,36.01073978040627,36.19473888642787,36.19473888652787,36.37773468076727,36.37773468086727,36.56042793005893,36.56042793015893,36.741564494575094,36.741564494675096,36.92204425189363,36.92204425199363,37.10247229210999,37.10247229220999,37.28275293858514,37.28275293868514,37.4629766962479,37.4629766963479,37.64279706051369,37.642797060613695,37.82143568899476,37.82143568909476,38.000014842808326,38.00001484290833,38.1785681380708,38.1785681381708,38.356795615589576,38.35679561568958,38.53421372045929,38.53421372055929,38.711176714829854,38.711176714929856,38.88564435902043,38.885644359120434,39.06005770025373,39.06005770035373,39.234318476035604,39.234318476135606,39.40792244461984,39.407922444719844,39.581146292503085,39.58114629260309,39.75370040391316,39.75370040401316,39.92623124262724,39.92623124272724,40.09855004122241,40.09855004132241,40.27043958786952,40.27043958796952,40.441980044076935,40.44198004417694,40.61268526908421,40.61268526918421,40.783072433913105,40.78307243401311,40.95318291224536,40.95318291234536,41.1225253916103,41.122525391710305,41.29107401345684,41.291074013556845,41.45943386788044,41.459433867980444,41.627279137137386,41.62727913723739,41.793337580514155,41.79333758061416,41.95931069067233,41.959310690772334,42.12463733705331,42.12463733715331,42.28833230886064,42.28833230896064,42.44998445513201,42.44998445523201,42.608657696318055,42.60865769641806,42.7662733227646,42.7662733228646,42.92277961736947,42.92277961746947,43.07732841965698,43.07732841975698,43.23126178842859,43.23126178852859,43.38499087464661,43.38499087474661,43.53852602173148,43.53852602183148,43.692037896120354,43.692037896220356,43.84513344783672,43.845133447936725,43.997910939374705,43.99791093947471,44.15043243125692,44.15043243135692,44.3019454396467,44.3019454397467,44.45309901417635,44.453099014276354,44.60313808515412,44.60313808525412,44.752817722271764,44.752817722371766,44.90219740019679,44.90219740029679,45.20004394919343,45.20004394929343,45.3484978909895,45.3484978910895,45.496933731799814,45.496933731899816,45.64471276541249,45.644712765512494,45.79225648621027,45.79225648631027,45.93970711622413,45.93970711632413,46.087015524207004,46.087015524307006,46.234313588769446,46.23431358886945,46.381358239531224,46.381358239631226,46.52767367915232,46.527673679252324,46.67371501813189,46.67371501823189,46.81969171073373,46.81969171083373,46.964212566909325,46.96421256700933,47.10765512150455,47.10765512160455,47.250916666242155,47.25091666634216,47.394051504079435,47.39405150417944,47.536242504802,47.536242504902,47.678386960132606,47.67838696023261,47.82029868850342,47.82029868860342,47.96082957024614,47.96082957034614,48.10122081581298,48.10122081591298,48.24155517256743,48.24155517266743,48.3813102977775,48.381310297877505,48.52104473614671,48.521044736246715,48.6607429725444,48.6607429726444,48.80043603723187,48.80043603733187,48.939950677916826,48.93995067801683,49.07876713772241,49.07876713782241,49.216722507776765,49.21672250787677,49.354644261714704,49.354644261814705,49.492454823882966,49.49245482398297,49.630138679150896,49.6301386792509,49.767592393314146,49.76759239341415,49.90475131999499,49.904751320094995,50.041899903255405,50.04189990335541,50.178815759556024,50.178815759656025,50.31567214118914,50.31567214128914,50.451470908082754,50.451470908182756,50.58647581745797,50.586475817557975,50.72143676729634,50.72143676739634,50.85618567701579,50.85618567711579,50.99027777953759,50.990277779637594,51.123221762391104,51.123221762491106,51.25604421005449,51.25604421015449,51.38872960739712,51.38872960749712,51.520900419573096,51.5209004196731,51.65300141366113,51.65300141376113,51.78499638768971,51.78499638778971,51.91639402918815,51.91639402928815,52.04745809537756,52.04745809547756,52.17760159714824,52.17760159724824,52.30770631109228,52.307706311192284,52.43779292405056,52.437792924150564,52.567801961355585,52.56780196145559,52.69748000920668,52.69748000930668,52.82577203871946,52.82577203881946,52.95282802949023,52.95282802959023,53.07906947590173,53.079069476001735,53.204726519058646,53.20472651915865,53.330365461229796,53.3303654613298,53.45582080768822,53.45582080778822,53.5809968817949,53.580996881894905,53.70597643091331,53.70597643101331,53.830803414580295,53.8308034146803,53.95479775290225,53.954797753002254,54.0781430415919,54.0781430416919,54.20138231022209,54.20138231032209,54.32449487195195,54.32449487205195,54.447209211995045,54.447209212095046,54.56951498693096,54.56951498703096,54.69163975200925,54.69163975210925,54.81349817401134,54.813498174111345,54.93499974800842,54.934999748108424,55.056333241423424,55.056333241523426,55.17760208846071,55.17760208856071,55.29886834964288,55.29886834974288,55.419423500670135,55.41942350077014,55.53981057111532,55.53981057121532,55.66000887413756,55.66000887423756,55.779886531126316,55.77988653122632,55.89921340097689,55.89921340107689,56.0179041504707,56.0179041505707,56.136357001294506,56.13635700139451,56.25452799391145,56.254527994011454,56.37257745133828,56.37257745143828,56.49054674725674,56.49054674735674,56.726203480886795,56.7262034809868,56.84376938340828,56.84376938350828,56.96085948858975,56.96085948868975,57.0774169076188,57.0774169077188,57.193865720733285,57.19386572083329,57.31029126115179,57.31029126125179,57.42623066080984,57.426230660909845,57.54210541409017,57.542105414190175,57.6574785114794,57.6574785115794,57.77278954834601,57.77278954844601,57.8879454339061,57.8879454340061,58.00298237013118,58.00298237023118,58.11780726623734,58.117807266337344,58.232445980775665,58.23244598087567,58.346802837107134,58.346802837207136,58.46114935001817,58.46114935011817,58.57513125735886,58.575131257458864,58.68907954858314,58.68907954868314,58.8030226680972,58.8030226681972,58.916950272480605,58.91695027258061,59.03083133147205,59.03083133157205,59.14466067336132,59.144660673461324,59.25793664225731,59.257936642357315,59.37114020721026,59.37114020731026,59.48422999453842,59.48422999463842,59.59675606545285,59.59675606555285,59.70888132882543,59.70888132892543,59.8208591984568,59.8208591985568,59.93241815956055,59.93241815966055,60.04369784831255,60.04369784841255,60.15477325451095,60.15477325461095,60.265825388013376,60.26582538811338,60.376849077109604,60.376849077209606,60.48740989814136,60.48740989824136,60.59777936589506,60.59777936599506,60.708120389242566,60.70812038934257,60.81783046394352,60.817830464043524,60.927232821886534,60.927232821986536,61.03656019003139,61.03656019013139,61.1455798414183,61.1455798415183,61.25458914938477,61.25458914948477,61.36254860017707,61.36254860027707,61.470233950327845,61.47023395042785,61.57738920018131,61.57738920028131,61.684438429975316,61.68443843007532,61.791474730493775,61.79147473059378,61.89825761721158,61.89825761731158,62.004334565484676,62.00433456558468,62.11013999897135,62.11013999907135,62.21591698805182,62.215916988151825,62.3215465833911,62.3215465834911,62.42713997675885,62.427139976858854,62.53252391586279,62.53252391596279,62.63785613786455,62.637856137964555,62.74296856218207,62.74296856228207,62.84780947171316,62.847809471813164,62.95220820002065,62.95220820012065,63.05621646420671,63.056216464306715,63.15988598137352,63.15988598147352,63.26349860972793,63.263498609827934,63.36660441048102,63.36660441058102,63.4693559490842,63.469355949184205,63.57196009394618,63.57196009404618,63.674517693416206,63.67451769351621,63.776871010332634,63.776871010432636,63.878642509849584,63.878642509949586,63.98039073667056,63.98039073677056,64.08178211548652,64.08178211558652,64.18300799957551,64.18300799967551,64.28401150012515,64.28401150022515,64.38488312206422,64.38488312216423,64.48573147130733,64.48573147140733,64.58617125544326,64.58617125554326,64.68656190833211,64.68656190843211,64.78680775333487,64.78680775343487,64.88702515393143,64.88702515403143,64.9866581512734,64.9866581513734,65.08565761411373,65.08565761421373,65.18435194605121,65.18435194615121,65.28303334871313,65.28303334881313,65.38129325699234,65.38129325709234,65.47903858010488,65.47903858020489,65.57667529730287,65.57667529740287,65.67392413623453,65.67392413633453,65.77099196530858,65.77099196540858,65.86774173420424,65.86774173430425,66.06087408057013,66.06087408067013,66.15685326464337,66.15685326474338,66.25233596453572,66.25233596463572,66.34730149340632,66.34730149350632,66.44214290123169,66.44214290133169,66.53663521861738,66.53663521871738,66.63079913240423,66.63079913250424,66.72451569325729,66.72451569335729,66.81795556761368,66.81795556771368,66.91124546237378,66.91124546247379,67.00440606437843,67.00440606447843,67.09745806046851,67.09745806056851,67.19045833945643,67.19045833955643,67.2833034671378,67.2833034672378,67.37547368663579,67.37547368673579,67.46755081534985,67.46755081544985,67.5592400657976,67.5592400658976,67.65076382151838,67.65076382161838,67.74180402233382,67.74180402243383,67.83235808238881,67.83235808248881,67.92264579936759,67.92264579946759,68.01275767819897,68.01275767829897,68.1028566277548,68.1028566278548,68.19276939574281,68.19276939584282,68.28246236604656,68.28246236614656,68.37192260939052,68.37192260949053,68.46135440832829,68.46135440842829,68.55077844970073,68.55077844980073,68.63964136051456,68.63964136061456,68.72763283815674,68.72763283825674,68.81520023556105,68.81520023566105,68.90256076455668,68.90256076465668,68.98972218270892,68.98972218280892,69.07683964132431,69.07683964142431,69.16365714074709,69.16365714084709,69.25038154938593,69.25038154948594,69.33705424092263,69.33705424102263,69.42367521535712,69.42367521545712,69.50975057436368,69.50975057446368,69.59564750936771,69.59564750946771,69.6813246466875,69.6813246467875,69.76691127907849,69.76691127917849,69.85219019471153,69.85219019481153,69.9373630902851,69.9373630903851,70.02220499640475,70.02220499650475,70.10687882194233,70.10687882204233,70.19153971820437,70.19153971830437,70.2761980286113,70.2761980287113,70.36070635942191,70.36070635952191,70.44518365997122,70.44518366007122,70.52910758752725,70.52910758762725,70.61289705061762,70.61289705071762,70.69667617028756,70.69667617038756,70.77999242189301,70.77999242199301,70.86328798665761,70.86328798675761,70.9465266626098,70.9465266627098,71.02950158134091,71.02950158144091,71.11239116685341,71.11239116695342,71.19476616719928,71.19476616729928,71.27687741032403,71.27687741042404,71.35885160312803,71.35885160322803,71.44071201830724,71.44071201840724,71.52210180785664,71.52210180795664,71.6032407694605,71.6032407695605,71.68399702450824,71.68399702460825,71.76468863317828,71.76468863327828,71.84535696915232,71.84535696925232,71.92595031532822,71.92595031542822,72.0061868134991,72.0061868135991,72.08618282714487,72.08618282724487,72.16600300264324,72.16600300274324,72.24578180445987,72.24578180455987,72.32504602110984,72.32504602120984,72.40415767230839,72.40415767240839,72.48306762680846,72.48306762690846,72.56168279382612,72.56168279392612,72.64005489046356,72.64005489056356,72.71840630026013,72.71840630036013,72.79668530611366,72.79668530621366,72.87441352482901,72.87441352492901,72.95209261229729,72.9520926123973,73.02957000306708,73.02957000316708,73.106907757661,73.10690775776101,73.18419379515275,73.18419379525275,73.26134278232372,73.26134278242372,73.33840902213122,73.33840902223122,73.41541578727121,73.41541578737122,73.49229325965577,73.49229325975577,73.56908539882174,73.56908539892174,73.64574565937716,73.64574565947716,73.72234903112017,73.72234903122018,73.79893430187744,73.79893430197744,73.87537993645881,73.87537993655882,73.95149458158627,73.95149458168628,74.02717221720035,74.02717221730035,74.1026378126955,74.1026378127955,74.17794308517391,74.17794308527391,74.25313458002752,74.25313458012752,74.32810110548668,74.32810110558668,74.40273146978168,74.40273146988169,74.47724029888658,74.47724029898659,74.55167930990353,74.55167931000354,74.62609504822451,74.62609504832452,74.70047199871887,74.70047199881887,74.77480240382125,74.77480240392126,74.8491198796481,74.8491198797481,74.92324600219689,74.92324600229689,74.99736695303547,74.99736695313547,75.0714775604536,75.0714775605536,75.14556489517577,75.14556489527577,75.21959534108554,75.21959534118554,75.29326635313518,75.29326635323518,75.36687789051733,75.36687789061733,75.44047391276881,75.44047391286881,75.51376997582769,75.51376997592769,75.586696261606,75.586696261706,75.65890626551916,75.65890626561917,75.73089130003788,75.73089130013788,75.8027341125256,75.8027341126256,75.87405458228136,75.87405458238136,75.94529747638384,75.94529747648384,76.01644469384729,76.01644469394729,76.0873979721776,76.0873979722776,76.1581469679543,76.1581469680543,76.22885459004927,76.22885459014927,76.29951308089716,76.29951308099716,76.37012761220821,76.37012761230821,76.44073697180903,76.44073697190903,76.51120152352377,76.51120152362377,76.58164021668742,76.58164021678742,76.65166258717855,76.65166258727855,76.72162806885729,76.72162806895729,76.7911461976022,76.7911461977022,76.86002562013525,76.86002562023525,76.92886884069677,76.92886884079677,76.99768878856231,76.99768878866232,77.06631221143958,77.06631221153958,77.13488391721468,77.13488391731468,77.20325392629128,77.20325392639128,77.2713317337406,77.2713317338406,77.33936558165307,77.33936558175307,77.40738132857977,77.40738132867978,77.47536087353495,77.47536087363495,77.54332748921459,77.54332748931459,77.61111826674683,77.61111826684683,77.67883664033603,77.67883664043603,77.74644123630043,77.7464412364
[truncated: 6,078,135 more chars]
