# Supplementary material for: Sequencing genomes from mixed DNA samples - evaluating the metagenome skimming approach in lichenized fungi
Source: Sci Rep. 2017 Nov 2;7:14881. doi: 10.1038/s41598-017-14576-6 (PMC5668418; doi:10.1038/s41598-017-14576-6)
Supplement: Supplementary file 2 — Supplementary_S6-S7 [file 41598_2017_14576_MOESM2_ESM.zip › Supplementary_S7.html]

|  |
| --- |
| QUAST **Quality Assessment Tool for Genome Assemblies** by Center for Algorithmic Biotechnology |

Loading...

Aligned to
""
|
 bp
|
 % G+C
|
 chromosomes
  
 reads
|
 mapped
|
 properly paired
  
 genes
|
 operons

Unfortunately, JavaScript in your
browser is disabled or is not supported.
We need JavaScript to build report and plots.

Worst
Median
Best

Show heatmap

Combined reference size:
 bp

|  |  |
| --- | --- |
|  |  |
|  | |  |
| Contigs are ordered from largest (contig #1) to smallest.  Contigs are broken into nonoverlapping 100 bp windows. Plot shows numbers of windows for each GC percentage. |

{"minContig":400,"report":[["Statistics without reference",[{"values":[3558,1829,4794,3702,6006,4036,8384,6340,10229,8029,21159,10238],"quality":"Less is better","isMain":true,"metricName":"# contigs"},{"values":[3558,1829,4794,3702,6006,4036,8384,6340,10229,8029,21159,10238],"quality":"Less is better","isMain":false,"metricName":"# contigs (>= 0 bp)"},{"values":[2534,1506,3481,2509,4045,2777,6081,4628,7594,6208,9922,5003],"quality":"Less is better","isMain":false,"metricName":"# contigs (>= 1000 bp)"},{"values":[1578,1002,1787,1214,2323,1627,2095,1595,3001,2437,2027,1369],"quality":"Less is better","isMain":false,"metricName":"# contigs (>= 5000 bp)"},{"values":[1031,745,1000,838,1276,978,799,650,1033,804,658,497],"quality":"Less is better","isMain":false,"metricName":"# contigs (>= 10000 bp)"},{"values":[493,409,382,373,315,256,126,117,72,52,137,116],"quality":"Less is better","isMain":false,"metricName":"# contigs (>= 25000 bp)"},{"values":[191,178,134,134,75,62,13,13,3,2,37,30],"quality":"Less is better","isMain":false,"metricName":"# contigs (>= 50000 bp)"},{"values":[422176,422176,279110,279110,157150,157150,85407,85407,56465,50623,138422,131237],"quality":"More is better","isMain":true,"metricName":"Largest contig"},{"values":[42531237,32487727,39683347,32909228,41129353,30248078,34558729,27225176,43649842,34956607,47465490,28484594],"quality":"More is better","isMain":true,"metricName":"Total length"},{"values":[42531237,32487727,39683347,32909228,41129353,30248078,34558729,27225176,43649842,34956607,47465490,28484594],"quality":"More is better","isMain":false,"metricName":"Total length (>= 0 bp)"},{"values":[41798193,32263499,38690790,31999028,39998335,29484808,33043024,26119952,41941765,33771674,40144691,25086668],"quality":"More is better","isMain":true,"metricName":"Total length (>= 1000 bp)"},{"values":[39499685,31122896,34783414,29485685,35493646,26640675,23343378,18625236,29706986,23439947,23091580,16900669],"quality":"More is better","isMain":false,"metricName":"Total length (>= 5000 bp)"},{"values":[35601447,29256101,29163915,26693003,27849759,21806994,14386161,12108026,15943237,12150712,13756610,10885086],"quality":"More is better","isMain":true,"metricName":"Total length (>= 10000 bp)"},{"values":[27004203,23793388,19507223,19153186,13178980,10822175,4542986,4268707,2294595,1641147,6310645,5301914],"quality":"More is better","isMain":false,"metricName":"Total length (>= 25000 bp)"},{"values":[16604018,15778336,10976473,10976473,5005449,4185952,801747,801747,157592,101127,2855552,2293013],"quality":"More is better","isMain":true,"metricName":"Total length (>= 50000 bp)"},{"values":[36386,48187,24051,31063,15817,17504,7992,8543,7533,7180,4784,6760],"quality":"More is better","isMain":false,"metricName":"N50"},{"values":[15825,23192,9426,13690,8166,9222,3894,3991,4187,4186,1664,2546],"quality":"More is better","isMain":false,"metricName":"N75"},{"values":[302,188,396,277,691,465,1124,813,1715,1439,2158,910],"quality":"Less is better","isMain":false,"metricName":"L50"},{"values":[741,433,1062,667,1609,1070,2678,1996,3662,3044,6345,2591],"quality":"Less is better","isMain":false,"metricName":"L75"},{"values":["46.99","49.34","47.34","49.66","46.93","49.51","47.28","49.73","47.29","49.75","47.73","50.14"],"quality":"Equal","isMain":false,"metricName":"GC (%)"}]],["Misassemblies",[{"values":[581,368,302,203,610,306,543,353,608,286,2637,1161],"quality":"Less is better","isMain":true,"metricName":"# misassemblies"},{"values":[286,202,199,141,233,138,415,298,247,154,359,161],"quality":"Less is better","isMain":false,"metricName":" # relocations"},{"values":[289,164,100,58,371,165,127,54,360,128,2237,982],"quality":"Less is better","isMain":false,"metricName":" # translocations"},{"values":[6,2,3,4,6,3,1,1,1,4,41,18],"quality":"Less is better","isMain":false,"metricName":" # inversions"},{"values":[377,257,242,171,440,255,450,305,477,261,2168,1009],"quality":"Less is better","isMain":false,"metricName":"# misassembled contigs"},{"values":[13130548,11187013,5781657,5127492,6033644,3839953,3656921,3001882,3352659,1935616,10843804,7188253],"quality":"Less is better","isMain":true,"metricName":"Misassembled contigs length"},{"values":[920,846,1430,1440,546,454,959,840,631,566,644,461],"quality":"Less is better","isMain":false,"metricName":"# local misassemblies"}]],["Unaligned",[{"values":[1039,119,1181,830,1813,1030,1054,744,551,273,1759,470],"quality":"Less is better","isMain":false,"metricName":"# fully unaligned contigs"},{"values":[2028106,299803,2257202,1323423,2072736,1121844,2072568,911476,1621920,603593,2830261,552437],"quality":"Less is better","isMain":false,"metricName":"Fully unaligned length"},{"values":[710,203,956,250,1045,366,1221,497,957,311,2335,635],"quality":"Less is better","isMain":false,"metricName":"# partially unaligned contigs"},{"values":[214,21,170,25,325,20,153,28,326,17,288,7],"quality":"Less is better","isMain":false,"metricName":" # with misassembly"},{"values":[310,70,237,61,462,113,293,96,534,136,643,111],"quality":"Less is better","isMain":false,"metricName":" # both parts are significant"},{"values":[5326559,522298,4786906,530873,5465603,479055,4252329,475465,4585204,416117,3771627,333967],"quality":"Less is better","isMain":false,"metricName":"Partially unaligned length"}]],["Mismatches",[{"values":[155996,125181,124603,104596,143940,104392,89881,68801,134029,106611,146066,95893],"quality":"Less is better","isMain":false,"metricName":"# mismatches"},{"values":[20893,19632,14834,14689,17488,14718,8632,8461,15206,14544,18655,15050],"quality":"Less is better","isMain":false,"metricName":"# indels"},{"values":[90964,85657,59979,59309,76704,65549,41424,39859,64818,61862,79415,63163],"quality":"Less is better","isMain":false,"metricName":"Indels length"},{"values":["473.13","413.73","402.20","353.63","455.33","377.88","338.76","279.45","452.68","384.44","491.17","411.55"],"quality":"Less is better","isMain":true,"metricName":"# mismatches per 100 kbp"},{"values":["63.37","64.88","47.88","49.66","55.32","53.28","32.53","34.37","51.36","52.45","62.73","64.59"],"quality":"Less is better","isMain":true,"metricName":"# indels per 100 kbp"},{"values":[16786,15773,12086,11968,14063,11746,6765,6664,12250,11739,15106,12218],"quality":"Less is better","isMain":false,"metricName":" # short indels"},{"values":[4107,3859,2748,2721,3425,2972,1867,1797,2956,2805,3549,2832],"quality":"Less is better","isMain":false,"metricName":" # long indels"},{"values":[165869,160822,587012,576870,0,0,17593,13880,2,2,6747,2412],"quality":"Less is better","isMain":false,"metricName":"# N's"},{"values":["389.99","495.02","1479.24","1752.91","0.00","0.00","50.91","50.98","0.00","0.01","14.21","8.47"],"quality":"Less is better","isMain":true,"metricName":"# N's per 100 kbp"}]],["Genome statistics",[{"values":["87.236","80.055","81.968","78.258","83.642","73.093","70.201","65.141","78.338","73.373","78.684","61.649"],"quality":"More is better","isMain":true,"metricName":"Genome fraction (%)"},{"values":["1.067","1.047","1.054","1.050","1.063","1.037","1.064","1.050","1.265","1.224","1.375","1.185"],"quality":"Less is better","isMain":true,"metricName":"Duplication ratio"},{"values":["6695 + 1100 part","6476 + 1011 part","6125 + 1636 part","5970 + 1504 part","6275 + 1428 part","5643 + 1182 part","4410 + 2752 part","4161 + 2494 part","4824 + 2680 part","4660 + 2529 part","4773 + 2867 part","4029 + 2611 part"],"quality":"More is better","isMain":true,"metricName":"# genes"},{"values":[422007,422007,277309,277309,157150,157150,67830,67830,50623,50623,114888,114888],"quality":"More is better","isMain":false,"metricName":"Largest alignment"},{"values":[42272,38234,26071,25513,17694,12688,7002,4866,8630,6593,6647,3900],"quality":"More is better","isMain":false,"metricName":"NG50"},{"values":[22156,12614,10777,7296,9679,2675,2859,null,5392,3407,3393,468],"quality":"More is better","isMain":false,"metricName":"NG75"},{"values":[25605,38906,19769,27157,11224,16241,5535,7744,5426,6808,2866,5806],"quality":"More is better","isMain":false,"metricName":"NA50"},{"values":[3268,16624,2560,10486,2377,8137,1213,3384,1768,3768,682,1941],"quality":"More is better","isMain":false,"metricName":"NA75"},{"values":[30783,29815,21546,21592,12692,11714,4688,4273,6496,6240,4561,3229],"quality":"More is better","isMain":true,"metricName":"NGA50"},{"values":[9713,6960,5275,3077,4708,null,null,null,3293,2935,1654,null],"quality":"More is better","isMain":false,"metricName":"NGA75"},{"values":[241,250,359,363,591,719,1340,1651,1352,1645,1298,1818],"quality":"Less is better","isMain":false,"metricName":"LG50"},{"values":[551,666,921,1029,1327,2085,3405,null,2739,3604,3306,9926],"quality":"Less is better","isMain":false,"metricName":"LG75"},{"values":[388,227,458,311,872,501,1420,885,2172,1511,3152,1114],"quality":"Less is better","isMain":false,"metricName":"LA50"},{"values":[1421,543,1625,786,2639,1165,4542,2213,5461,3233,12516,3168],"quality":"Less is better","isMain":false,"metricName":"LA75"},{"values":[303,305,413,411,732,778,1738,1815,1678,1729,1816,2191],"quality":"Less is better","isMain":false,"metricName":"LGA50"},{"values":[829,906,1255,1337,1915,null,null,null,3692,3874,5277,null],"quality":"Less is better","isMain":false,"metricName":"LGA75"}]],["Predicted genes",[]],["Reference statistics",[{"values":[37795223,37795223,37795223,37795223,37795223,37795223,37795223,37795223,37795223,37795223,37795223,37795223],"quality":"Equal","isMain":false,"metricName":"Reference length"},{"values":["47.86","47.86","47.86","47.86","47.86","47.86","47.86","47.86","47.86","47.86","47.86","47.86"],"quality":"Equal","isMain":false,"metricName":"Reference GC (%)"},{"values":[8842,8842,8842,8842,8842,8842,8842,8842,8842,8842,8842,8842],"quality":"Equal","isMain":false,"metricName":"Reference genes"}]]],"referenceName":"Pseudevernia\_furfuracea\_reference","date":"13 September 2017, Wednesday, 16:56:22","order":[0,1,2,3,4,5,6,7,8,9,10,11],"assembliesNames":["SPAdes MEGAN","SPAdes MetaWatt","metaSPAdes MEGAN","metaSPAdes MetaWatt","IDBA-UD MEGAN","IDBA-UD MetaWatt","MetaVelvet MEGAN","MetaVelvet MetaWatt","omega MEGAN","omega MetaWatt","mira MEGAN","mira MetaWatt"]}

{{ qualities }}

{{ mainMetrics }}

{"lists\_of\_lengths":[[2766500,1852973,1564434,1402837,1230560,1105076,1061431,979684,921797,885298,841011,788317,745209,706489,669742,645349,616330,583164,565015,542777,525449,509634,493857,475437,462154,444840,431946,423867,413531,401488,392045,384756,375412,364877,355732,346458,333374,323358,317282,309158,300117,290382,283732,278319,270420,263938,256187,250593,246819,241388,233517,228141,222852,219211,214161,210299,205005,200554,195857,191143,186945,183610,179657,174984,171841,169639,165936,160956,157142,153750,151722,148174,144931,140719,137677,134320,132194,128877,126354,124351,121927,120136,117436,114800,112463,109902,107682,106353,104567,103211,101884,100018,98817,97053,94300,92922,91641,90199,88785,87656,86280,85020,84088,83126,81291,79673,78446,77506,76527,75267,73396,71793,70382,68362,65986,63969,62607,61327,59674,58125,56499,55013,53781,52206,51248,50158,48483,47179,46373,45212,44267,43232,42133,40841,39670,38630,37696,36713,35931,35127,34594,33974,33345,32595,31776,30966,30140,29223,28491,28165,27662,27115,26509,25978,25382,24989,24403,23802,23436,23011,22273,21836,21472,20845,20248,19794,19313,18879,18348,17778,17358,17017,16632,16278,15997,15582,15156,14773,14524,14340,14143,13888,13670,13471,13332,13176,12942,12758,12619,12446,12261,12124,11989,11815,11666,11588,11479,11323,11188,11103,11007,10925,10803,10693,10611,10526,10457,10399,10286,10184,10113,10068,10008,9942,9878,9838,9786,9725,9683,9631,9548,9487,9436,9389,9342,9300,9245,9199,9168,9143,9105,9049,9010,8972,8941,8905,8870,8843,8816,8779,8742,8711,8679,8576,8338,8131,8000,7882,7708,7527,7380,7281,7093,6235,813],[2766500,1852973,1564434,1395078,1218106,1099669,1054864,967977,916411,872571,805526,752574,710424,669827,641589,603432,568383,545641,522479,500752,474306,454230,434125,419580,404707,392327,382377,370360,356981,345590,331196,318488,307196,293009,282858,271845,257611,248696,241853,231847,222627,215045,207590,201986,193827,186319,181306,176190,171049,165859,157341,151960,146276,138059,132030,126889,123769,119653,115462,111203,107148,103808,100169,97064,93081,90362,86705,83137,79647,76439,72135,69737,64612,62121,59268,55292,52129,49826,46824,44940,42732,39947,37672,35392,34032,31497,29127,28057,26945,26106,25168,24454,23738,23225,22685,21977,21452,20695,19958,19298,18391,17552,16894,16188,15457,15000,14502,14031,13591,13188,12744,12278,11776,11368,11031,10580,10206,9733,9349,9097,8948,8636,8356,8154,8029,7922,7751,7541,7391,6829,3781],[2356929,1538945,1221806,1097262,1006263,939954,866777,804732,739335,698640,655976,617219,587035,557894,539759,523378,503559,477839,464629,451528,435172,419685,406194,392440,379417,366705,357684,347806,334756,327326,316841,308409,301177,293902,287201,281540,277031,270010,263726,258581,254991,251560,245820,240836,234303,228826,223089,218963,214803,210481,201990,196273,191170,187248,184567,182382,178887,173638,170727,167270,164234,161266,158717,156356,154091,151566,148679,146694,144679,143060,141518,139978,138303,136304,134063,132466,131234,129507,128281,126711,124837,122974,120914,118427,116687,115322,113957,112125,110337,108879,107545,106352,104856,103683,102821,101734,100354,99272,98479,97448,96466,95140,93937,92822,92121,91451,90360,89153,87764,86733,85563,84754,84057,83080,82218,81536,80500,79573,78855,77838,76656,75542,74742,73897,72884,71991,71054,70056,69265,68408,67304,66484,65590,64629,63272,62155,61166,60328,59189,58542,57550,56691,55929,55312,54768,53738,52881,52227,51291,50451,49533,48604,47752,46959,46289,45634,45138,44507,43704,43075,42343,41480,41034,40548,39742,38896,38331,37679,37204,36606,35913,35156,34725,34420,33908,33515,33093,32461,31732,31126,30631,30141,29524,28993,28488,28073,27558,27144,26731,26353,25995,25748,25251,24943,24553,24253,24045,23660,23380,23176,22956,22677,22280,21961,21626,21415,21036,20754,20547,20287,20047,19770,19491,19277,19139,18901,18618,18401,18126,17910,17664,17523,17421,17269,17070,16898,16768,16604,16485,16354,16198,16030,15824,15699,15486,15375,15282,15192,15088,14982,14852,14755,14668,14622,14509,14360,14203,14111,14017,13832,13693,13637,13537,13435,13342,13241,13175,13100,13008,12962,12875,12785,12738,12674,12623,12551,12466,12401,12317,12242,12172,12121,12058,12002,11932,11879,11815,11748,11690,11646,11581,11518,11460,11386,11303,11227,11152,11091,11039,10991,10948,10900,10852,10809,10749,10696,10637,10561,10489,10436,10367,10285,10207,10131,10052,9989,9876,9823,9744,9700,9655,9602,9519,9459,9404,9345,9286,9221,9173,9144,9109,9052,9015,8984,8928,8884,8849,8819,8780,8742,8683,8528,8352,8130,7994,7777,7567,7376,7094,6683,6307,5958,2448],[2356929,1538945,1221806,1097262,1006263,939954,866777,804732,739335,698640,654199,613275,582486,551644,532984,517468,487030,470684,456705,441037,423137,409998,398367,384468,371250,360700,351202,339508,328936,318934,309548,299414,291592,285296,278861,270954,263436,258277,254247,248818,242592,234698,226071,219401,214253,207368,198532,193010,186845,183169,179447,173062,168825,164007,159078,154429,150958,147143,144006,141001,138396,135449,132459,130894,128757,126903,123608,118504,115744,113481,110362,107911,105228,101787,99254,97612,95094,93110,91462,88179,84847,81615,79458,76642,73565,71921,70294,68376,66088,63549,61010,58620,55943,55060,53119,50937,49178,46958,45443,44248,42520,41128,39443,38379,37434,36620,35633,34836,33955,33373,32590,31810,31133,30549,29868,28993,28472,27901,27329,26927,26451,26106,25702,25268,25001,24643,24182,23848,23467,23145,22873,22630,22262,21925,21560,21273,20976,20688,20362,20099,19870,19657,19308,19032,18665,18463,18259,18031,17821,17683,17524,17379,17234,17077,16915,16771,16645,16502,16377,16207,16034,15874,15734,15620,15512,15418,15342,15252,15180,15033,14918,14758,14637,14545,14466,14394,14271,14149,14056,13966,13856,13735,13671,13591,13498,13427,13329,13229,13161,13054,12965,12904,12831,12763,12708,12636,12548,12467,12415,12356,12312,12251,12194,12113,12057,11993,11922,11867,11801,11706,11644,11554,11494,11455,11386,11288,11186,11121,11066,11013,10949,10890,10837,10787,10742,10691,10623,10559,10452,10378,10314,10220,10085,9975,9895,9821,9726,9652,9580,9494,9417,9354,9275,9200,9135,9081,9019,8922,8859,8800,8752,8700,8595,8358,8166,7992,7838,7638,7380,6999,6745,6490,6078,5785,2422],[1362209,967500,875380,809624,738520,694071,652114,616965,577176,549702,522707,493549,477263,465636,447891,432426,417518,404125,390673,378431,368884,360414,350432,342580,334911,326799,318894,311280,306749,302078,296368,290623,286923,282534,278007,272696,267849,264677,260475,256824,253981,249779,246554,242427,238724,234331,229795,226710,224089,221103,217549,214126,211770,209531,206079,203519,201516,198443,196171,193587,191034,188061,186305,184610,182908,180407,178128,175690,173971,172251,170030,168176,166670,164655,162858,161007,159742,158466,157106,155374,154303,152843,151795,150127,147956,146249,145046,144085,143348,142052,141210,139541,138225,137094,135917,133996,132839,132095,131110,130138,128748,127002,125847,124619,123401,122511,121295,120469,119831,118896,118098,117129,116460,115676,114734,113783,112526,111385,110102,109377,108399,107218,106296,105457,104624,103992,102768,101726,100926,100228,98930,98014,97285,96365,95398,94675,93726,93120,92575,91858,90829,89556,88539,87906,87486,87012,86368,85422,84450,83530,82749,82105,81292,80100,79090,78194,77590,76579,75617,74785,74156,73381,72505,71774,71190,70290,69596,68984,68282,67588,66916,66241,65568,64966,63961,63193,62384,61482,60766,60176,59757,59397,58833,58067,57288,56414,55790,55121,54357,53638,52970,52309,51746,51316,50827,50178,49668,48922,48365,47936,47505,47000,46483,45699,45220,44757,44371,43816,43156,42673,42335,41857,41394,40934,40428,39958,39629,39173,38581,38025,37549,37223,36858,36389,35879,35550,34937,34364,33804,33422,33086,32684,32164,31796,31494,31038,30432,29923,29497,29079,28694,28350,27964,27616,27252,26879,26258,25780,25307,24999,24622,24069,23772,23428,23126,22669,22172,21901,21566,21214,20925,20683,20362,20141,19986,19634,19314,18981,18717,18443,18159,17923,17657,17451,17103,16905,16623,16242,15991,15713,15526,15333,15147,14975,14750,14564,14396,14202,14042,13901,13657,13441,13263,13092,12927,12771,12670,12481,12322,12152,12026,11831,11626,11523,11399,11244,11127,11010,10889,10755,10645,10528,10423,10304,10191,10095,9977,9834,9719,9614,9502,9425,9323,9208,9111,9019,8945,8848,8743,8663,8582,8518,8455,8410,8349,8291,8235,8179,8118,8047,7976,7930,7884,7833,7792,7747,7712,7677,7651,7633,7596,7553,7499,7452,7411,7388,7348,7297,7259,7217,7186,7152,7132,7105,7066,7027,6989,6955,6921,6895,6858,6838,6820,6793,6778,6760,6734,6709,6694,6682,6662,6639,6618,6599,6579,6556,6540,6519,6507,6492,6477,6465,6448,6440,6421,6404,6392,6376,6367,6353,6341,6328,6314,6294,6280,6264,6251,6244,6235,6218,6206,6191,6183,6173,6160,6150,6139,6129,6118,6105,6095,6087,6075,6062,6060,6048,6036,6034,5989],[1314386,953806,851585,760197,698058,641899,598563,558617,527533,494771,475524,458564,440549,420497,404330,387145,373000,362318,347350,336936,326424,315254,308398,302483,294793,287452,280599,272705,266769,262201,258120,252715,248085,242996,237749,232580,228086,224460,221046,216805,212881,209641,205224,202156,198174,195086,191338,187817,185337,182998,180155,177328,174634,172643,170281,168150,165724,162467,160207,158507,156357,154483,152733,150944,148297,146303,144909,143684,142272,140884,138781,137152,135050,132942,131823,130529,128627,126542,124861,123157,121869,120497,119230,118076,116903,115706,114463,113023,111374,109713,108082,106504,105396,104425,103430,101701,100424,98307,96853,95215,93658,92297,89886,88135,87136,85551,83656,82274,80726,79454,77851,75977,74447,73338,72231,71392,69693,68620,67546,66731,65738,64347,62960,61312,60115,59362,58291,56929,55763,54302,53158,52078,51216,50265,49356,48279,47354,46358,45298,44466,43634,42809,42140,41159,40483,39857,39238,38405,37684,36985,35972,35265,34491,33634,32930,32368,31868,31291,30292,29371,28923,28230,27646,27185,26639,25972,25199,24586,23926,23463,22988,22291,21783,21216,20801,20474,20147,19861,19434,19027,18680,18454,18229,17895,17524,17210,17004,16740,16429,16184,15844,15667,15402,15236,15002,14772,14519,14178,13959,13684,13461,13221,13034,12842,12637,12443,12235,12040,11873,11723,11599,11452,11262,11078,10937,10812,10639,10465,10324,10214,10095,9988,9823,9678,9506,9360,9243,9109,9021,8913,8830,8743,8638,8476,8391,8317,8246,8157,8080,8020,7948,7886,7808,7754,7709,7660,7606,7510,7438,7394,7332,7284,7220,7171,7106,7041,7001,6956,6907,6836,6786,6732,6690,6652,6630,6596,6567,6532,6501,6474,6455,6434,6408,6379,6366,6337,6299,6274,6239,6213,6181,6158,6134,6121,6091,6076,6061,6038,1720],[850809,633439,551653,490753,453136,428509,400004,376648,358035,343598,327819,314481,304770,296293,285517,275465,266187,258129,250572,243590,237634,231346,226500,222252,219013,214624,211199,209297,206253,201946,197449,193146,189945,187608,184874,182441,180409,178041,175990,173905,171763,169545,166462,164271,162275,160597,159435,157659,156152,153829,151485,148998,146642,145656,144322,142626,141164,139366,138029,136720,135701,134584,133256,131755,130192,128791,127201,125667,123708,122545,121438,120256,119254,118179,117570,116584,115276,114312,113542,112608,111700,110800,109988,109238,108002,106586,105762,104925,103948,103294,102525,101498,100726,100016,98968,98165,97452,96729,95958,95182,94616,93904,93270,92533,91952,91132,90193,89639,89041,88461,87765,86846,86134,85611,85084,84388,83915,83487,83178,82888,82461,81943,81613,81246,80776,80389,79986,79611,79247,78673,78264,77917,77536,77125,76713,76320,75758,75324,74934,74639,74229,73642,72962,72476,72039,71640,71171,70858,70557,70114,69599,69181,68762,68462,68130,67623,67225,66739,66193,65842,65459,64975,64571,64100,63638,63315,62795,62342,62030,61748,61409,61197,60869,60403,59963,59712,59321,59083,58793,58566,58361,58090,57690,57415,56985,56725,56378,55920,55532,55156,54855,54463,54196,53933,53551,52964,52497,52082,51841,51590,51278,51001,50696,50258,50018,49851,49632,49314,48918,48578,48233,47968,47748,47441,47157,46913,46661,46461,46294,46100,45767,45484,45346,45084,44808,44550,44332,44025,43759,43491,43293,42985,42686,42429,42098,41839,41641,41385,41151,40847,40556,40319,40130,39939,39714,39495,39316,39083,38916,38791,38580,38232,37995,37791,37601,37423,37233,37080,36811,36481,36228,36047,35869,35687,35536,35281,35038,34855,34701,34480,34244,33995,33801,33591,33371,33223,33033,32852,32712,32453,32258,32038,31888,31743,31565,31353,31191,31032,30822,30651,30463,30309,30083,29959,29840,29703,29568,29481,29289,29046,28876,28742,28623,28459,28266,28078,27915,27706,27567,27391,27241,27153,27040,26918,26751,26634,26473,26328,26151,26028,25883,25718,25553,25448,25345,25252,25092,24945,24826,24693,24497,24325,24145,24020,23870,23742,23596,23444,23314,23202,23050,22932,22850,22730,22619,22536,22449,22321,22170,22047,21931,21804,21677,21556,21394,21305,21234,21119,20963,20881,20768,20682,20619,20546,20470,20324,20178,20015,19885,19746,19649,19559,19434,19330,19232,19140,19012,18910,18763,18648,18575,18482,18366,18296,18150,18013,17924,17828,17755,17707,17609,17522,17432,17332,17240,17151,17096,17039,16977,16915,16790,16683,16594,16505,16405,16307,16241,16144,16079,15966,15837,15756,15673,15583,15507,15424,15349,15293,15190,15127,15059,15010,14946,14811,14702,14614,14534,14459,14400,14348,14260,14179,14133,14066,13986,13914,13862,13805,13719,13651,13588,13555,13509,13446,13354,13272,13181,13068,12992,12921,12844,12765,12696,12644,12605,12520,12464,12397,12294,12217,12178,12124,12038,11986,11941,11854,11799,11747,11675,11620,11554,11489,11428,11369,11294,11205,11100,11023,10963,10896,10825,10757,10686,10652,10602,10540,10478,10420,10380,10337,10301,10254,10206,10146,10073,10023,9967,9897,9845,9789,9745,9694,9641,9584,9542,9482,9431,9399,9356,9306,9261,9204,9155,9119,9063,9005,8959,8915,8871,8830,8799,8741,8688,8648,8616,8577,8528,8479,8435,8387,8322,8268,8213,8160,8117,8054,7998,7961,7915,7858,7805,7742,7712,7679,7621,7573,7515,7467,7431,7371,7319,7290,7251,7220,7182,7135,7103,7076,7008,6966,6910,6851,6809,6761,6728,6696,6673,6638,6594,6553,6527,6495,6465,6415,6381,6349,6314,6282,6251,6204,6162,6144,6119,6097,6074,6033,5990,5963,5925,5887,5857,5826,5805,5781,5763,5718,5684,5651,4811],[850809,633439,551653,486369,447331,418812,387864,366449,347266,327590,312733,302110,290947,276425,265512,256276,246825,239695,231524,225825,221493,217013,211731,209622,206208,201329,195875,190984,187376,183475,180533,177792,174873,172441,169303,165715,162870,160458,158290,155527,152534,149248,146596,145437,143737,141934,139636,137606,135933,134890,133249,131385,129704,127752,126085,123696,121996,120487,118925,117798,116479,114878,113852,112678,111219,109750,108005,106362,104944,103625,102555,101028,99632,98488,97589,96524,95387,94483,93624,92651,91775,90719,89721,88893,88185,86986,86020,85460,84554,83813,83277,82778,82040,81550,80790,80094,79560,78891,78189,77655,77132,76624,75939,75286,74848,74360,73750,73052,72456,71935,71411,70951,70604,70194,69629,69124,68591,68272,67610,67217,66625,66031,65443,64828,64311,63706,63262,62644,62098,61711,61323,60936,60381,59858,59352,58980,58568,58297,57835,57526,56979,56621,55927,55390,55028,54544,54235,53948,53623,52814,52248,51909,51621,51211,50907,50433,50037,49784,49329,48788,48359,48014,47679,47219,46814,46462,46201,45808,45483,45248,44958,44620,44307,43995,43623,43340,43048,42739,42317,41900,41638,41281,40898,40567,40356,40145,39884,39587,39365,39068,38860,38641,38258,37917,37705,37542,37376,37150,36824,36488,36259,35964,35727,35582,35425,35131,34914,34693,34394,34026,33675,33488,33286,33001,32801,32660,32439,32287,32057,31909,31733,31521,31320,31149,30978,30786,30641,30460,30229,30008,29849,29674,29522,29385,29123,28893,28723,28596,28370,28160,27935,27661,27429,27251,27077,26866,26710,26581,26424,26208,26054,25859,25635,25458,25322,25168,24869,24629,24404,24186,23978,23780,23607,23389,23194,23004,22821,22650,22489,22347,22160,21985,21801,21602,21431,21279,21174,20986,20793,20667,20531,20382,20213,20037,19890,19714,19539,19322,19190,19026,18909,18803,18678,18513,18393,18266,18092,17933,17763,17650,17512,17357,17244,17140,17034,16947,16833,16721,16608,16496,16348,16231,16104,15987,15884,15745,15614,15503,15375,15276,15143,15038,14939,14722,14609,14508,14394,14291,14196,14124,14018,13894,13831,13762,13680,13590,13467,13322,13217,13099,12963,12871,12745,12640,12563,12462,12346,12260,12158,12031,11933,11825,11736,11652,11568,11480,11383,11280,11175,11083,10986,10916,10833,10761,10661,10567,10494,10418,10339,10256,10171,10099,10034,9963,9902,9828,9755,9667,9565,9472,9409,9356,9294,9228,9140,9059,8963,8904,8846,8790,8708,8641,8610,8550,8487,8420,8349,8299,8238,8180,8137,8075,8025,7956,7881,7827,7759,7717,7669,7603,7561,7506,7437,7355,7305,7269,7220,7151,7118,7060,6998,6953,6893,6832,6790,6731,6666,6616,6564,6514,6457,6381,6352,6328,6307,6276,6231,6183,6149,6121,6086,6053,6016,5981,5937,5872,5813,5764,5734,5709,5675,5646,4804],[598568,478501,418187,381952,366493,348206,339639,327390,317495,308781,299913,288863,281640,273677,265041,256821,252923,249537,243607,237394,232766,229576,225821,220105,216958,214223,210917,207607,205182,202918,200732,197241,194512,191655,189552,188179,187169,185269,183535,181609,180310,178477,176794,175810,174243,173063,171786,170601,168957,167311,165317,163635,161952,160664,159539,158023,157038,155780,154543,153602,152483,151369,150620,149395,148370,147369,146188,145381,144607,143725,143162,141830,140903,140212,139258,138332,137202,136345,135421,134384,133526,132710,131992,131461,130540,129599,128808,127833,126830,125683,124656,124108,123354,122717,122152,121580,120916,120458,119915,119173,118497,118076,117397,116571,116031,115219,114357,113686,113151,112805,112166,111689,111110,110743,110133,109575,108938,108160,107648,107112,106595,105966,105456,104918,104437,103823,103252,102817,102407,101981,101552,100863,100190,99598,99168,98752,98311,97733,97227,96777,96478,96013,95447,94957,94102,93721,93416,92961,92522,92100,91708,91311,91043,90660,90220,89837,89487,89126,88765,88479,88237,87863,87516,87177,86857,86480,85921,85672,85272,84785,84276,83806,83511,83201,82852,82421,81963,81492,81043,80624,80269,79875,79560,79253,79001,78822,78417,78052,77776,77514,77209,76891,76606,76292,75935,75527,75099,74625,74374,74078,73735,73422,73103,72800,72514,72263,71946,71660,71387,71193,71002,70802,70434,70167,69948,69711,69533,69357,69040,68771,68583,68403,68090,67831,67577,67290,67046,66696,66509,66292,66076,65787,65511,65277,65097,64856,64605,64385,64081,63743,63524,63276,63056,62892,62710,62453,62177,61922,61672,61348,61115,60868,60648,60465,60275,60057,59896,59654,59395,59134,58894,58620,58377,58115,57790,57550,57315,57058,56730,56433,56216,55981,55805,55611,55387,55137,54821,54548,54318,54093,53883,53677,53449,53148,52867,52588,52338,52140,51939,51684,51473,51304,51134,50857,50653,50472,50271,50047,49834,49616,49423,49162,48796,48563,48375,48170,47981,47794,47603,47432,47317,47163,46931,46700,46385,46177,46050,45838,45682,45501,45308,45114,44908,44696,44509,44357,44202,44023,43866,43791,43625,43307,43129,42937,42684,42458,42230,42055,41935,41806,41672,41510,41328,41178,41016,40823,40578,40449,40316,40152,39965,39787,39617,39382,39173,38934,38813,38645,38433,38286,38151,38020,37767,37484,37313,37145,37014,36873,36703,36555,36407,36303,36113,35915,35710,35439,35258,35141,34988,34834,34707,34565,34424,34211,34026,33826,33624,33479,33358,33223,33087,32929,32795,32643,32465,32342,32143,31961,31845,31749,31679,31512,31413,31313,31165,30988,30782,30639,30478,30295,30149,29971,29798,29640,29485,29296,29171,29048,28916,28776,28632,28467,28351,28233,28089,27899,27734,27602,27478,27252,27120,26986,26842,26710,26586,26426,26203,26013,25890,25757,25616,25516,25355,25192,25052,24890,24730,24570,24365,24168,24020,23840,23699,23530,23385,23285,23160,23043,22912,22739,22656,22501,22350,22252,22172,22080,21954,21848,21711,21600,21468,21352,21240,21119,20993,20910,20774,20605,20492,20369,20235,20142,19972,19850,19719,19613,19509,19390,19261,19112,19016,18931,18868,18780,18671,18563,18465,18285,18123,18023,17929,17856,17747,17604,17473,17373,17244,17153,17023,16960,16867,16766,16696,16636,16572,16468,16377,16303,16194,16053,15968,15880,15784,15684,15620,15521,15437,15322,15235,15151,15031,14931,14853,14770,14706,14635,14497,14422,14321,14241,14173,14095,13997,13902,13794,13697,13613,13534,13459,13346,13248,13137,13052,12947,12846,12763,12691,12586,12512,12434,12381,12304,12239,12161,12087,12029,11951,11896,11834,11771,11702,11643,11577,11497,11431,11365,11334,11264,11171,11093,11024,10953,10876,10774,10668,10595,10531,10473,10403,10334,10255,10214,10135,10057,10021,9949,9877,9837,9799,9764,9699,9651,9603,9569,9508,9443,9396,9364,9324,9298,9265,9231,9186,9118,9072,9035,8994,8956,8917,8878,8849,8816,8784,8754,8731,8702,8653,8608,8571,8525,8505,8477,8435,8402,8369,8327,8286,8256,8227,8193,8166,8158,8142,8119,8097,8084,8069,8047,8030,8019,8008,7998,7980,7960,7944,7933,7917,7902,7890,7877,7865,7854,7844,7834,7813,7803,7789,7781,7769,7756,7743,7729,7713,7700,7688,7677,7661,7652,7641,7630,7619,7616,7603,7593,7574,7563,7550,7546,7533,7522,7507,7494,7480,7460,7447,7434,7423,7417,7399,7387,7371,7355,7343,7332,7317,7299,7282,7267,7251,7233,7212,7202,7183,7163,7139,7115,7088,7052,7023,6987,6953,6910,6872,6825,6777,6698,6489,6363,6132,5925,3677],[558807,437987,383990,359999,341802,325867,309821,295240,283518,273226,260648,252535,247511,237657,232207,228477,223018,217289,213728,209666,206237,203606,201359,197271,193762,190362,188349,186806,184384,182508,180514,177890,176147,174535,172851,171177,169112,166889,164367,161960,160421,158875,157481,156123,154509,153287,151790,150753,149515,148265,146979,145796,144833,143788,143028,141510,140640,139685,138565,137071,135604,134161,132915,131959,131151,129891,128793,127416,126148,124970,124057,123098,122311,121692,120989,120458,119653,118666,118166,117362,116527,115708,114681,113680,113105,112419,111721,111024,110349,109512,108597,107923,107138,106380,105663,105084,104421,103797,103084,102583,102077,101652,100776,100055,99509,99069,98569,98002,97296,96781,96423,95840,95207,94308,93696,93357,92745,92322,91846,91389,91069,90668,90084,89560,89087,88692,88300,87828,87465,87114,86648,86037,85662,85244,84712,84043,83586,83262,82922,82395,81856,81167,80685,80299,79908,79564,79231,78948,78627,78218,77871,77622,77220,76880,76570,76282,75872,75434,74913,74468,74177,73836,73481,73136,72841,72540,72258,71884,71501,71264,71042,70727,70371,70108,69854,69574,69367,69027,68738,68552,68264,67869,67523,67182,66879,66558,66340,66113,65841,65605,65338,65126,64865,64621,64410,64085,63738,63503,63241,63030,62875,62644,62402,62129,61881,61615,61358,61199,60940,60700,60458,60213,59949,59793,59448,59161,58877,58598,58360,58097,57742,57518,57255,56877,56540,56235,55952,55755,55520,55304,55065,54686,54429,54206,54046,53804,53594,53268,53019,52664,52366,52086,51781,51500,51301,50949,50636,50356,50145,49915,49700,49476,49050,48652,48395,48181,47895,47674,47459,47234,46949,46669,46329,46146,45867,45652,45433,45212,44977,44716,44497,44278,44085,43891,43752,43497,43172,42917,42624,42447,42164,41984,41812,41629,41443,41237,41048,40910,40693,40511,40355,40178,39967,39767,39566,39322,39064,38822,38651,38450,38273,38095,37931,37631,37426,37176,37010,36849,36669,36450,36224,35966,35724,35443,35248,35065,34833,34624,34348,34085,33897,33669,33470,33280,33140,32985,32799,32609,32421,32260,32090,31937,31803,31676,31479,31370,31258,31108,30876,30712,30551,30322,30123,29897,29643,29455,29257,29140,28957,28800,28635,28401,28221,28014,27835,27668,27515,27342,27106,26903,26701,26549,26387,26150,25946,25807,25646,25508,25319,25146,24934,24726,24580,24411,24131,23966,23760,23525,23281,23155,23047,22898,22715,22550,22365,22206,22088,21937,21737,21577,21446,21286,21134,20974,20853,20654,20484,20285,20074,19922,19716,19567,19415,19220,19091,18996,18879,18784,18622,18407,18211,18063,17930,17808,17627,17463,17354,17222,17070,16967,16842,16722,16569,16492,16401,16306,16159,15945,15798,15689,15601,15468,15344,15234,15110,14971,14811,14730,14609,14450,14318,14223,14106,13989,13838,13724,13596,13490,13379,13259,13138,13016,12876,12784,12713,12615,12488,12371,12258,12116,12010,11885,11800,11683,11587,11526,11433,11342,11222,11108,11025,10948,10797,10621,10515,10433,10366,10281,10185,10088,10006,9901,9798,9746,9662,9579,9527,9452,9388,9334,9293,9245,9171,9108,9047,8994,8936,8894,8844,8800,8747,8690,8631,8589,8520,8467,8419,8379,8341,8297,8267,8218,8175,8146,8109,8084,8055,8029,8006,7984,7961,7938,7906,7883,7866,7849,7835,7813,7794,7777,7755,7736,7705,7689,7670,7654,7633,7614,7595,7573,7557,7545,7530,7511,7490,7475,7454,7439,7424,7405,7388,7369,7354,7339,7318,7300,7280,7261,7245,7223,7200,7177,7159,7128,7089,7030,6975,6931,6882,6796,6650,6430,6073,2887],[1429716,929608,739875,623808,555108,487608,453715,419186,385594,361157,339752,315193,301797,290165,279385,266438,256530,246535,237594,230718,223189,212910,206765,199673,194137,191194,187813,182159,179444,175937,172441,170155,167794,166199,164659,161689,159429,157729,156075,153925,152360,150838,148399,146082,143825,141806,140502,139187,137791,136163,133518,132252,131189,129769,128541,126782,125619,124708,123427,121937,120562,119123,117828,117127,115798,114899,113636,112557,111514,110656,109721,108661,107737,106971,106109,105284,104607,103685,103074,102068,101394,100836,100225,99719,98953,98192,97538,96838,96066,95446,94719,93956,93142,92370,91599,91027,90395,89926,89487,89028,88650,87976,87291,86696,86270,85891,85423,84910,84568,84200,83725,83270,82848,82505,82097,81800,81325,80603,80156,79781,79425,78993,78611,78220,77731,77426,76967,76570,76300,75878,75423,75043,74568,74123,73829,73447,73020,72622,72291,72032,71589,71167,70795,70452,70100,69820,69578,69293,68853,68444,68123,67876,67508,67227,66826,66446,66192,65912,65636,65318,64941,64572,64330,64021,63727,63476,63132,62814,62592,62282,61982,61708,61434,61089,60840,60583,60407,60230,59984,59660,59465,59246,59025,58849,58562,58291,58062,57728,57370,57153,56932,56809,56555,56247,56034,55836,55630,55432,55254,55003,54710,54494,54290,53996,53778,53496,53288,53090,52874,52620,52313,52099,51849,51621,51432,51239,51035,50874,50686,50547,50379,50197,50036,49837,49653,49422,49204,49000,48797,48638,48506,48290,48100,47911,47776,47614,47397,47145,46941,46806,46642,46468,46316,46187,46076,45916,45786,45591,45478,45288,45071,44943,44838,44719,44576,44447,44301,44164,43981,43794,43679,43554,43352,43149,42978,42824,42700,42527,42356,42218,42049,41859,41741,41577,41437,41245,41067,40840,40689,40497,40389,40260,40099,39950,39814,39644,39510,39367,39264,39149,39010,38824,38685,38541,38423,38281,38133,38007,37880,37720,37587,37449,37303,37183,37056,36959,36858,36752,36597,36437,36303,36178,36027,35906,35800,35661,35572,35451,35372,35262,35130,35002,34910,34795,34681,34529,34419,34332,34218,34096,33981,33817,33700,33623,33553,33489,33413,33293,33160,33001,32888,32785,32658,32536,32460,32393,32304,32213,32102,31979,31889,31785,31664,31508,31386,31208,31080,30968,30882,30786,30681,30607,30529,30453,30381,30312,30226,30136,30039,29949,29869,29771,29678,29598,29485,29407,29339,29253,29159,29091,29018,28939,28803,28674,28571,28514,28442,28358,28275,28197,28111,28003,27902,27829,27775,27641,27554,27464,27363,27265,27200,27130,27043,26966,26884,26802,26700,26597,26504,26412,26340,26258,26190,26118,26044,25954,25864,25778,25713,25626,25534,25432,25334,25234,25152,25080,25011,24927,24845,24791,24744,24657,24570,24500,24437,24365,24303,24239,24164,24101,24035,23978,23895,23857,23805,23757,23694,23638,23588,23528,23469,23389,23314,23260,23174,23124,23054,22988,22932,22869,22826,22796,22747,22688,22651,22619,22570,22502,22454,22410,22356,22290,22236,22192,22149,22074,22027,21962,21870,21812,21747,21700,21641,21586,21519,21475,21430,21360,21288,21246,21214,21160,21090,21033,20973,20914,20866,20809,20761,20713,20653,20601,20545,20505,20462,20409,20363,20311,20269,20230,20190,20155,20125,20094,20049,20002,19946,19894,19837,19780,19735,19690,19640,19593,19550,19498,19434,19385,19345,19297,19253,19209,19178,19139,19112,19074,19045,19004,18968,18916,18885,18832,18797,18767,18736,18701,18669,18618,18580,18534,18501,18463,18428,18397,18340,18291,18242,18207,18171,18128,18099,18071,18035,18001,17966,17919,17880,17853,17809,17772,17742,17697,17666,17631,17577,17523,17492,17469,17439,17412,17387,17345,17306,17269,17227,17183,17146,17108,17074,17057,17034,17001,16961,16918,16887,16851,16818,16787,16754,16722,16691,16663,16627,16604,16571,16526,16499,16474,16448,16417,16391,16362,16339,16308,16286,16254,16218,16190,16164,16133,16106,16081,16057,16030,15999,15979,15935,15905,15880,15843,15816,15783,15757,15732,15707,15683,15659,15633,15604,15574,15546,15526,15505,15481,15464,15431,15400,15373,15343,15321,15298,15274,15249,15224,15196,15184,15160,15138,15117,15086,15067,15048,15020,14989,14966,14946,14919,14904,14890,14859,14836,14813,14798,14778,14749,14739,14714,14702,14686,14664,14636,14614,14591,14569,14546,14525,14489,14453,14427,14411,14393,14374,14356,14340,14315,14295,14280,14264,14240,14217,14201,14173,14161,14138,14113,14087,14062,14028,13999,13964,13944,13910,13888,13870,13851,13825,13804,13781,13745,13717,13692,13667,13651,13628,13603,13580,13553,13536,13514,13488,13467,13448,13426,13409,13387,13371,13346,13319,13297,13269,13251,13231,13209,13193,13167,13135,13115,13097,13078,13060,13042,13027,13005,12984,12967,12949,12930,12910,12888,12866,12842,12824,12803,12789,12773,12757,12735,12712,12698,12683,12656,12633,12622,12600,12580,12564,12551,12533,12512,12479,12465,12443,12425,12408,12390,12363,12344,12320,12298,12279,12259,12245,12226,12210,12194,12180,12161,12145,12125,12111,12090,12071,12054,12041,12028,12008,11989,11972,11954,11936,11917,11901,11877,11860,11839,11823,11799,11785,11774,11760,11741,11726,11710,11689,11669,11648,11634,11623,11611,11602,11588,11578,11560,11544,11533,11521,11507,11494,11487,11479,11466,11460,11447,11434,11421,11410,11396,11385,11374,11361,11348,11340,11324,11312,11304,11296,11284,11270,11261,11253,11242,11242,11230,11227,11214,11206,11200,11192,11186,11177,11166,11158,11149,11144,11137,11130,11123,11114,11102,11102,11089,11088,11077,11074,11065,11060,11050,11039,11032,11018,11014,11004,10996,10990,10980,10976,10962,10957,10948,10939,10934,10923,10920,10917,10906,10906,10892,10887,10878,10868,10864,10853,10842,10836,10826,10822,10810,10808,10796,10786,10777,10766,10760,10752,10739,10738,10726,10724,10711,10705,10696,10692,10682,10670,10659,10654,10641,10632,10626,10614,10611,10598,10595,10584,10572,10565,10556,10542,10541,10528,10521,10514,10501,10494,10486,10472,10465,10458,10445,10433,10423,10413,10402,10395,10388,10374,10368,10357,10345,10332,10323,10312,10304,10291,10283,10275,10259,10241,10227,10213,10199,10192,10181,10167,10151,10138,10124,10119,10104,10085,10071,10066,10052,10042,10029,10014,10000,9991,9974,9966,9944,9939,9926,9915,9904,9889,9872,9855,9839,9828,9815,9800,9786,9777,9767,9751,9742,9729,9716,9703,9692,9680,9667,9655,9643,9630,9618,9603,9590,9579,9569,9554,9542,9525,9509,9502,9491,9478,9466,9455,9444,9431,9422,9411,9394,9381,9377,9366,9353,9339,9329,9322,9310,9298,9286,9273,9262,9244,9235,9222,9212,9196,9184,9174,9163,9150,9139,9128,9112,9098,9086,9072,9066,9055,9044,9031,9025,9015,9002,8993,8976,8964,8950,8946,8933,8921,8907,8899,8877,8863,8854,8846,8834,8823,8809,8797,8790,8778,8770,8755,8737,8727,8722,8708,8702,8689,8678,8663,8652,8638,8631,8624,8614,8606,8596,8589,8580,8568,8559,8550,8540,8526,8520,8510,8498,8493,8478,8465,8456,8442,8434,8417,8413,8400,8390,8377,8367,8357,8339,8330,8317,8310,8302,8288,8277,8272,8260,8247,8241,8229,8218,8209,8195,8185,8176,8165,8161,8148,8140,8134,8118,8106,8099,8092,8079,8078,8067,8059,8049,8036,8025,8018,8008,7995,7983,7974,7966,7954,7945,7937,7924,7913,7905,7896,7883,7874,7865,7854,7844,7835,7826,7819,7812,7808,7798,7790,7784,7775,7768,7756,7755,7742,7742,7732,7728,7717,7709,7700,7693,7686,7682,7672,7661,7658,7644,7644,7635,7630,7626,7616,7616,7607,7602,7602,7591,7588,7582,7574,7574,7566,7560,7560,7548,7546,7542,7532,7532,7518,7518,7515,7504,7504,7495,7490,7490,7485,7476,7476,7465,7462,7456,7448,7448,7448,7438,7434,7429,7420,7420,7420,7410,7406,7406,7402,7392,7392,7392,7381,7378,7378,7378,7368,7364,7364,7364,7350,7350,7350,7350,7336,7336,7336,7336,7323,7322,7322,7318,7308,7308,7308,7306,7294,7294,7294,7283,7280,7280,7280,7270,7266,7266,7266,7263,7252,7252,7252,7252,7241,7238,7238,7238,7237,7224,7224,7224,7222,7210,7210,7210,7205,7196,7196,7196,7196,7182,7182,7182,7182,7169,7168,7168,7168,7162,7154,7154,7154,7154,7140,7140,7140,7140,7138,7126,7126,7126,7123,7112,7112,7112,7106,7098,7098,7098,7098,7085,7084,7084,7084,7077,7070,7070,7070,7070,7063,7056,7056,7056,7049,7042,7042,7042,7042,7032,7028,7028,7028,7023,7014,7014,7014,7014,7006,7000,7000,7000,6989,6986,6986,6981,6972,6972,6972,6964,6958,6958,6958,6947,6944,6944,6935,6930,6930,6930,6922,6916,6916,6915,6902,6902,6894,6888,6888,6881,6874,6874,6874,6862,6860,6860,6854,6846,6844,6832,6832,6832,6832,6819,6818,6816,6804,6804,6804,6791,6790,6788,6776,6776,6763,6762,6755,6748,6748,6738,6734,6731,6720,6720,6711,6706,6706,6692,6692,6678,6678,6666,6664,6651,6645,6636,6628,6622,6615,6608,6604,6594,6589,6580,6579,6566,6553,6543,6536,6524,6520,6509,6496,6487,6472,6464,6448,6432,6424,6406,6388,6378,6354,6334,6313,6293,6271,6250,6233,6211,6186,6155,6131,6105,6078,6042,6018,5989,5973,5956,5918,5893,5856,5819,5773,5728,5694,5664],[1334934,856914,668360,583598,508895,455788,413863,377281,349704,326062,301729,286610,272182,259143,247896,237476,228553,219560,207299,198595,192980,188763,182009,178014,172855,169730,167047,165186,161955,158674,155738,152254,148761,144868,141644,139929,137953,135475,132714,130965,128973,126485,125223,123922,121801,119720,117859,116446,115144,113148,111498,110354,108751,107317,106020,105003,103830,102539,101416,100696,99962,98609,97391,96207,95244,93769,92489,91603,90573,89608,88835,87949,86888,86083,85522,84748,84131,83288,82681,82088,81610,80378,79671,79084,78361,77566,76989,76385,75845,75270,74644,73979,73385,72534,72082,71302,70635,70169,69723,69364,68846,68181,67741,67211,66666,66253,65845,65328,64729,64126,63638,63145,62551,62097,61608,61195,60875,60546,60159,59774,59320,58803,58435,57933,57187,56680,56115,55749,55412,54856,54384,53931,53566,53269,52916,52517,51964,51433,51040,50706,50366,50006,49590,49166,48738,48422,47990,47723,47327,46956,46639,46274,46032,45711,45397,45166,44922,44601,44316,43952,43727,43302,42945,42628,42263,41909,41670,41400,40869,40407,40126,39931,39637,39269,38763,38464,38192,37864,37499,37233,36977,36776,36446,36111,35803,35543,35303,35077,34829,34587,34426,34209,33923,33621,33459,33193,32898,32589,32447,32221,31815,31485,31226,30944,30712,30484,30285,30130,29954,29783,29547,29355,29185,29007,28721,28463,28257,28007,27851,27658,27360,27199,27058,26853,26602,26429,26262,26079,25857,25655,25443,25228,25012,24834,24712,24531,24408,24305,24181,24009,23838,23679,23548,23418,23316,23192,23052,22926,22817,22727,22621,22430,22281,22189,22058,21909,21777,21649,21521,21423,21257,21122,20944,20788,20696,20572,20423,20316,20240,20156,20073,19974,19847,19752,19679,19586,19499,19404,19311,19194,19088,19002,18881,18778,18722,18640,18546,18483,18381,18266,18142,18035,17968,17879,17819,17719,17619,17520,17470,17420,17382,17296,17216,17148,17077,17033,16957,16893,16829,16765,16700,16645,16592,16517,16469,16403,16336,16259,16170,16077,16010,15930,15846,15770,15716,15662,15597,15531,15456,15393,15325,15286,15235,15182,15136,15062,15009,14961,14897,14842,14795,14751,14706,14673,14613,14565,14522,14461,14414,14375,14330,14284,14239,14202,14158,14120,14072,13987,13923,13869,13834,13799,13751,13678,13633,13592,13551,13511,13469,13424,13363,13325,13293,13242,13187,13151,13120,13082,13030,12982,12944,12905,12842,12793,12753,12709,12686,12644,12610,12562,12530,12485,12441,12397,12365,12318,12263,12235,12197,12158,12114,12063,12028,11990,11970,11937,11899,11850,11804,11775,11748,11703,11644,11617,11589,11560,11532,11497,11470,11443,11414,11387,11364,11335,11308,11284,11255,11238,11222,11198,11186,11168,11157,11144,11128,11108,11096,11078,11057,11038,11013,10987,10970,10955,10942,10925,10909,10891,10871,10852,10830,10809,10782,10763,10742,10729,10703,10689,10663,10642,10625,10612,10598,10576,10560,10544,10525,10506,10481,10461,10437,10411,10389,10368,10348,10322,10300,10279,10252,10226,10201,10185,10157,10135,10102,10075,10047,10025,10002,9975,9945,9919,9895,9850,9821,9795,9775,9752,9734,9710,9684,9651,9627,9599,9573,9540,9514,9485,9463,9437,9414,9388,9359,9329,9300,9269,9241,9223,9200,9173,9147,9118,9087,9060,9032,9011,8981,8962,8945,8922,8898,8866,8841,8819,8798,8774,8743,8714,8694,8679,8656,8640,8618,8590,8573,8550,8530,8504,8481,8454,8438,8422,8403,8379,8358,8325,8303,8284,8269,8248,8235,8214,8182,8157,8136,8114,8097,8079,8054,8028,8010,7991,7968,7948,7930,7906,7884,7861,7846,7822,7800,7789,7775,7767,7749,7735,7715,7698,7684,7668,7646,7628,7615,7602,7588,7577,7569,7560,7544,7528,7518,7506,7490,7474,7461,7448,7442,7434,7422,7415,7406,7394,7392,7379,7375,7364,7350,7349,7336,7336,7326,7322,7317,7308,7300,7294,7280,7278,7266,7266,7252,7252,7240,7238,7225,7223,7210,7208,7196,7194,7182,7176,7166,7154,7154,7141,7140,7128,7124,7112,7111,7098,7098,7085,7084,7077,7070,7066,7056,7056,7043,7042,7030,7028,7016,7009,7000,7000,6988,6981,6972,6964,6958,6945,6938,6930,6919,6916,6909,6902,6888,6888,6874,6874,6865,6857,6846,6834,6832,6825,6817,6804,6793,6781,6770,6759,6743,6734,6724,6709,6696,6683,6668,6653,6635,6619,6608,6594,6569,6549,6538,6521,6502,6476,6453,6419,6390,6357,6320,6275,6246,6171,6115,6059,6009,5964,5903,5843,5782,5712,5629,1600]],"filenames":["SPAdes MEGAN","SPAdes MetaWatt","metaSPAdes MEGAN","metaSPAdes MetaWatt","IDBA-UD MEGAN","IDBA-UD MetaWatt","MetaVelvet MEGAN","MetaVelvet MetaWatt","omega MEGAN","omega MetaWatt","mira MEGAN","mira MetaWatt"]}

{"assemblies\_lengths":[42531237,32487727,39683347,32909228,41129353,30248078,34558729,27225176,43649842,34956607,47465490,28484594],"filenames":["SPAdes MEGAN","SPAdes MetaWatt","metaSPAdes MEGAN","metaSPAdes MetaWatt","IDBA-UD MEGAN","IDBA-UD MetaWatt","MetaVelvet MEGAN","MetaVelvet MetaWatt","omega MEGAN","omega MetaWatt","mira MEGAN","mira MetaWatt"]}

{"reflen":[3053396,2571993,2475134,2016333,1838764,1697380,1670865,1482537,1354612,1178799,1157191,1116932,1109772,1060420,1016036,947025,891529,879049,859355,811843,788710,722111,713509,675010,648997,614230,514486,447612,443437,377661,310071,284163,277523,234680,220229,198034,181446,180704,137591,136892,133632,133044,89667,51966,51088,39765]}

{"tickX":14}

{"coord\_y":[[422176,422176,422176,267682,267682,223384,223384,218535,218535,198367,198367,181933,181933,179084,179084,175245,175245,155784,155784,154599,154599,152033,152033,149358,149358,144827,144827,143493,143493,141354,141354,141268,141268,139546,139546,139245,139245,135878,135878,135578,135578,133590,133590,131144,131144,130984,130984,127794,127794,126413,126413,125676,125676,122869,122869,121634,121634,120296,120296,120077,120077,118729,118729,117926,117926,114179,114179,113203,113203,111768,111768,111224,111224,109605,109605,109331,109331,105649,105649,104421,104421,104344,104344,103682,103682,103493,103493,102751,102751,101797,101797,101710,101710,101525,101525,101495,101495,100169,100169,100083,100083,99650,99650,99441,99441,98763,98763,98555,98555,97753,97753,95652,95652,94992,94992,93587,93587,93521,93521,92259,92259,90122,90122,89968,89968,86218,86218,85406,85406,84936,84936,84186,84186,83997,83997,83963,83963,83837,83837,83568,83568,82538,82538,80031,80031,79894,79894,79844,79844,79114,79114,79078,79078,79038,79038,78800,78800,78763,78763,78421,78421,77718,77718,77495,77495,77200,77200,77142,77142,77131,77131,77086,77086,77002,77002,76915,76915,76820,76820,76715,76715,76476,76476,75709,75709,75524,75524,75476,75476,74473,74473,74419,74419,74219,74219,73466,73466,73433,73433,72840,72840,71804,71804,70733,70733,70485,70485,69938,69938,69933,69933,69766,69766,69589,69589,68597,68597,68363,68363,68119,68119,68074,68074,68010,68010,67453,67453,67113,67113,66804,66804,66429,66429,66301,66301,66243,66243,65926,65926,65351,65351,65323,65323,65274,65274,65067,65067,65034,65034,64851,64851,64628,64628,64601,64601,64579,64579,64474,64474,64429,64429,64060,64060,63290,63290,63061,63061,62888,62888,62563,62563,62514,62514,62365,62365,62177,62177,62150,62150,62147,62147,61962,61962,61208,61208,61150,61150,61106,61106,60934,60934,60757,60757,60359,60359,60287,60287,60221,60221,59479,59479,58924,58924,58529,58529,58330,58330,57765,57765,57591,57591,57578,57578,57500,57500,57215,57215,56987,56987,56799,56799,56644,56644,56033,56033,55913,55913,55848,55848,55451,55451,55149,55149,54883,54883,54726,54726,54417,54417,54027,54027,54018,54018,53866,53866,53799,53799,53599,53599,53557,53557,53419,53419,53255,53255,52390,52390,52369,52369,52331,52331,52115,52115,52047,52047,51723,51723,51684,51684,51400,51400,50944,50944,50776,50776,50557,50557,50407,50407,50000,50000,49944,49944,49882,49882,49772,49772,49173,49173,48827,48827,48651,48651,48618,48618,48441,48441,48390,48390,48187,48187,48157,48157,47986,47986,47826,47826,47606,47606,47599,47599,47171,47171,47048,47048,47043,47043,47019,47019,46960,46960,46926,46926,46862,46862,46834,46834,46682,46682,46375,46375,45986,45986,45872,45872,45663,45663,45591,45591,45529,45529,45471,45471,45318,45318,45280,45280,45252,45252,44874,44874,44792,44792,44728,44728,44715,44715,44415,44415,44186,44186,44142,44142,44027,44027,43563,43563,43519,43519,42986,42986,42599,42599,42532,42532,42519,42519,42413,42413,42272,42272,42264,42264,41952,41952,41942,41942,41865,41865,41707,41707,41123,41123,41079,41079,41060,41060,41059,41059,41011,41011,40898,40898,40842,40842,40786,40786,40744,40744,40693,40693,40686,40686,40556,40556,40340,40340,40259,40259,40188,40188,40086,40086,40069,40069,40058,40058,39943,39943,39765,39765,39593,39593,39391,39391,39255,39255,39232,39232,39208,39208,38733,38733,38721,38721,38681,38681,38659,38659,38481,38481,38234,38234,38230,38230,38196,38196,38163,38163,38029,38029,37919,37919,37918,37918,37749,37749,37736,37736,37735,37735,37597,37597,37567,37567,37503,37503,37451,37451,37209,37209,37184,37184,36988,36988,36864,36864,36843,36843,36708,36708,36645,36645,36610,36610,36583,36583,36479,36479,36391,36391,36386,36386,36378,36378,36274,36274,36197,36197,36133,36133,36079,36079,35928,35928,35793,35793,35792,35792,35751,35751,35738,35738,35734,35734,35658,35658,35410,35410,35372,35372,35182,35182,34832,34832,34801,34801,34640,34640,34591,34591,34563,34563,34366,34366,34273,34273,34202,34202,34182,34182,34086,34086,34045,34045,33983,33983,33917,33917,33833,33833,33767,33767,33765,33765,33741,33741,33722,33722,33555,33555,33532,33532,33516,33516,33502,33502,33390,33390,33317,33317,33300,33300,33086,33086,32946,32946,32826,32826,32796,32796,32667,32667,32465,32465,32412,32412,32399,32399,32360,32360,32282,32282,31988,31988,31850,31850,31828,31828,31764,31764,31724,31724,31689,31689,31683,31683,31676,31676,31670,31670,31643,31643,31410,31410,31273,31273,31177,31177,31123,31123,31114,31114,31073,31073,31015,31015,30901,30901,30872,30872,30869,30869,30841,30841,30650,30650,30635,30635,30592,30592,30548,30548,30536,30536,30494,30494,30460,30460,30417,30417,30414,30414,30407,30407,30402,30402,30308,30308,30287,30287,30203,30203,30177,30177,30126,30126,30092,30092,30043,30043,30037,30037,29991,29991,29876,29876,29861,29861,29752,29752,29645,29645,29567,29567,29529,29529,29524,29524,29422,29422,29383,29383,29308,29308,29260,29260,29250,29250,29163,29163,29137,29137,29136,29136,28979,28979,28929,28929,28927,28927,28711,28711,28662,28662,28562,28562,28500,28500,28494,28494,28478,28478,28333,28333,28330,28330,28310,28310,28210,28210,28204,28204,28188,28188,28157,28157,28085,28085,28053,28053,28025,28025,27922,27922,27919,27919,27918,27918,27899,27899,27879,27879,27801,27801,27785,27785,27777,27777,27769,27769,27734,27734,27628,27628,27533,27533,27472,27472,27454,27454,27448,27448,27408,27408,27390,27390,27347,27347,27272,27272,27264,27264,27260,27260,27233,27233,27170,27170,27156,27156,27113,27113,27044,27044,26892,26892,26815,26815,26809,26809,26631,26631,26566,26566,26539,26539,26502,26502,26472,26472,26470,26470,26320,26320,26318,26318,26308,26308,26266,26266,26264,26264,26220,26220,26112,26112,26043,26043,25998,25998,25897,25897,25878,25878,25789,25789,25732,25732,25659,25659,25607,25607,25534,25534,25503,25503,25493,25493,25482,25482,25459,25459,25424,25424,25385,25385,25293,25293,25283,25283,25225,25225,25199,25199,25186,25186,25102,25102,25060,25060,25023,25023,24984,24984,24973,24973,24797,24797,24724,24724,24624,24624,24615,24615,24593,24593,24548,24548,24501,24501,24487,24487,24427,24427,24183,24183,24173,24173,24078,24078,24009,24009,23955,23955,23895,23895,23894,23894,23757,23757,23715,23715,23616,23616,23579,23579,23557,23557,23500,23500,23463,23463,23402,23402,23359,23359,23192,23192,23170,23170,23165,23165,23141,23141,23129,23129,23108,23108,23063,23063,23029,23029,22960,22960,22946,22946,22883,22883,22811,22811,22809,22809,22796,22796,22791,22791,22758,22758,22750,22750,22740,22740,22722,22722,22720,22720,22700,22700,22694,22694,22568,22568,22501,22501,22380,22380,22353,22353,22252,22252,22250,22250,22234,22234,22231,22231,22156,22156,22100,22100,22083,22083,22070,22070,22067,22067,22028,22028,21973,21973,21929,21929,21907,21907,21878,21878,21828,21828,21755,21755,21684,21684,21634,21634,21553,21553,21521,21521,21501,21501,21396,21396,21395,21395,21337,21337,21214,21214,21114,21114,21093,21093,21092,21092,21051,21051,20991,20991,20972,20972,20866,20866,20864,20864,20846,20846,20794,20794,20697,20697,20679,20679,20627,20627,20602,20602,20488,20488,20467,20467,20438,20438,20413,20413,20399,20399,20389,20389,20311,20311,20309,20309,20305,20305,20271,20271,20227,20227,20206,20206,20191,20191,20182,20182,20177,20177,20175,20175,20139,20139,20110,20110,20024,20024,19966,19966,19965,19965,19961,19961,19850,19850,19844,19844,19831,19831,19828,19828,19767,19767,19721,19721,19679,19679,19634,19634,19600,19600,19552,19552,19474,19474,19459,19459,19406,19406,19370,19370,19331,19331,19289,19289,19257,19257,19163,19163,19154,19154,19141,19141,19112,19112,19091,19091,19038,19038,19033,19033,19009,19009,19007,19007,18987,18987,18867,18867,18854,18854,18816,18816,18813,18813,18685,18685,18608,18608,18580,18580,18550,18550,18438,18438,18406,18406,18378,18378,18368,18368,18366,18366,18348,18348,18347,18347,18291,18291,18248,18248,18242,18242,18231,18231,18216,18216,18173,18173,18135,18135,18110,18110,18096,18096,18083,18083,18005,18005,17926,17926,17898,17898,17873,17873,17862,17862,17827,17827,17817,17817,17803,17803,17775,17775,17764,17764,17754,17754,17738,17738,17733,17733,17722,17722,17704,17704,17697,17697,17665,17665,17659,17659,17631,17631,17592,17592,17588,17588,17560,17560,17556,17556,17519,17519,17455,17455,17447,17447,17412,17412,17406,17406,17392,17392,17391,17391,17381,17381,17366,17366,17334,17334,17297,17297,17069,17069,17053,17053,17019,17019,16924,16924,16897,16897,16888,16888,16853,16853,16814,16814,16766,16766,16731,16731,16723,16723,16694,16694,16676,16676,16598,16598,16593,16593,16585,16585,16580,16580,16555,16555,16461,16461,16445,16445,16436,16436,16424,16424,16400,16400,16353,16353,16342,16342,16335,16335,16289,16289,16246,16246,16197,16197,16189,16189,16170,16170,16159,16159,16156,16156,16125,16125,16071,16071,16068,16068,15962,15962,15955,15955,15937,15937,15876,15876,15855,15855,15846,15846,15842,15842,15840,15840,15835,15835,15825,15825,15815,15815,15811,15811,15782,15782,15754,15754,15718,15718,15690,15690,15664,15664,15656,15656,15647,15647,15639,15639,15606,15606,15582,15582,15578,15578,15544,15544,15540,15540,15437,15437,15420,15420,15403,15403,15384,15384,15356,15356,15351,15351,15345,15345,15326,15326,15325,15325,15212,15212,15207,15207,15136,15136,15134,15134,15125,15125,15121,15121,15095,15095,15078,15078,15066,15066,15056,15056,15030,15030,15022,15022,15016,15016,15015,15015,15009,15009,14979,14979,14948,14948,14937,14937,14927,14927,14795,14795,14762,14762,14724,14724,14718,14718,14701,14701,14642,14642,14636,14636,14632,14632,14619,14619,14618,14618,14612,14612,14536,14536,14517,14517,14493,14493,14475,14475,14424,14424,14411,14411,14406,14406,14379,14379,14375,14375,14322,14322,14310,14310,14290,14290,14247,14247,14226,14226,14154,14154,14129,14129,14113,14113,14112,14112,14110,14110,14076,14076,14056,14056,14045,14045,14009,14009,13973,13973,13962,13962,13904,13904,13890,13890,13871,13871,13846,13846,13827,13827,13795,13795,13742,13742,13681,13681,13669,13669,13642,13642,13628,13628,13621,13621,13618,13618,13606,13606,13567,13567,13538,13538,13528,13528,13487,13487,13471,13471,13460,13460,13420,13420,13386,13386,13372,13372,13349,13349,13315,13315,13311,13311,13288,13288,13277,13277,13276,13276,13273,13273,13260,13260,13247,13247,13169,13169,13167,13167,13157,13157,13154,13154,13136,13136,13127,13127,13115,13115,13109,13109,13075,13075,13061,13061,13055,13055,13028,13028,13010,13010,13001,13001,12983,12983,12977,12977,12957,12957,12893,12893,12870,12870,12858,12858,12854,12854,12797,12797,12796,12796,12738,12738,12663,12663,12638,12638,12632,12632,12631,12631,12614,12614,12604,12604,12559,12559,12544,12544,12513,12513,12493,12493,12480,12480,12460,12460,12440,12440,12431,12431,12424,12424,12416,12416,12375,12375,12358,12358,12322,12322,12310,12310,12306,12306,12303,12303,12300,12300,12289,12289,12272,12272,12264,12264,12253,12253,12229,12229,12220,12220,12212,12212,12203,12203,12200,12200,12194,12194,12188,12188,12165,12165,12153,12153,12147,12147,12143,12143,12130,12130,12075,12075,12070,12070,12056,12056,12042,12042,12020,12020,12018,12018,11996,11996,11951,11951,11950,11950,11916,11916,11878,11878,11821,11821,11795,11795,11793,11793,11791,11791,11779,11779,11778,11778,11747,11747,11723,11723,11688,11688,11687,11687,11669,11669,11557,11557,11551,11551,11525,11525,11524,11524,11451,11451,11400,11400,11389,11389,11388,11388,11378,11378,11371,11371,11345,11345,11342,11342,11288,11288,11280,11280,11252,11252,11237,11237,11232,11232,11216,11216,11215,11215,11201,11201,11189,11189,11165,11165,11119,11119,11061,11061,11032,11032,11029,11029,11027,11027,11019,11019,11002,11002,10994,10994,10989,10989,10976,10976,10970,10970,10958,10958,10948,10948,10941,10941,10933,10933,10932,10932,10909,10909,10899,10899,10892,10892,10873,10873,10868,10868,10844,10844,10839,10839,10822,10822,10812,10812,10781,10781,10768,10768,10743,10743,10737,10737,10666,10666,10629,10629,10628,10628,10606,10606,10586,10586,10585,10585,10556,10556,10512,10512,10501,10501,10495,10495,10468,10468,10462,10462,10461,10461,10441,10441,10428,10428,10420,10420,10386,10386,10372,10372,10341,10341,10324,10324,10297,10297,10295,10295,10278,10278,10240,10240,10220,10220,10185,10185,10178,10178,10136,10136,10113,10113,10108,10108,10089,10089,10033,10033,10022,10022,10013,10013,9982,9982,9970,9970,9967,9967,9964,9964,9959,9959,9945,9945,9933,9933,9913,9913,9891,9891,9879,9879,9853,9853,9840,9840,9828,9828,9813,9813,9806,9806,9773,9773,9753,9753,9739,9739,9711,9711,9709,9709,9702,9702,9623,9623,9618,9618,9599,9599,9582,9582,9579,9579,9577,9577,9573,9573,9558,9558,9552,9552,9551,9551,9550,9550,9547,9547,9540,9540,9502,9502,9494,9494,9489,9489,9477,9477,9475,9475,9473,9473,9447,9447,9438,9438,9405,9405,9384,9384,9366,9366,9361,9361,9343,9343,9329,9329,9271,9271,9266,9266,9261,9261,9247,9247,9228,9228,9224,9224,9212,9212,9179,9179,9144,9144,9140,9140,9129,9129,9127,9127,9120,9120,9111,9111,9104,9104,9091,9091,9089,9089,9057,9057,9039,9039,9027,9027,9017,9017,8999,8999,8984,8984,8979,8979,8963,8963,8952,8952,8942,8942,8940,8940,8939,8939,8921,8921,8911,8911,8909,8909,8903,8903,8879,8879,8877,8877,8846,8846,8841,8841,8834,8834,8822,8822,8787,8787,8779,8779,8773,8773,8770,8770,8768,8768,8747,8747,8739,8739,8691,8691,8684,8684,8682,8682,8670,8670,8662,8662,8657,8657,8653,8653,8652,8652,8651,8651,8640,8640,8624,8624,8612,8612,8590,8590,8583,8583,8573,8573,8572,8572,8560,8560,8555,8555,8545,8545,8542,8542,8534,8534,8498,8498,8459,8459,8458,8458,8456,8456,8454,8454,8422,8422,8418,8418,8386,8386,8345,8345,8324,8324,8323,8323,8312,8312,8294,8294,8287,8287,8285,8285,8271,8271,8262,8262,8238,8238,8212,8212,8211,8211,8209,8209,8195,8195,8175,8175,8171,8171,8152,8152,8147,8147,8137,8137,8135,8135,8127,8127,8114,8114,8113,8113,8096,8096,8093,8093,8062,8062,8050,8050,8008,8008,8004,8004,7980,7980,7968,7968,7958,7958,7945,7945,7934,7934,7930,7930,7913,7913,7899,7899,7890,7890,7881,7881,7864,7864,7831,7831,7825,7825,7816,7816,7814,7814,7798,7798,7776,7776,7731,7731,7730,7730,7719,7719,7718,7718,7714,7714,7712,7712,7700,7700,7689,7689,7688,7688,7687,7687,7665,7665,7663,7663,7658,7658,7650,7650,7640,7640,7639,7639,7633,7633,7623,7623,7611,7611,7602,7602,7598,7598,7597,7597,7584,7584,7578,7578,7571,7571,7570,7570,7554,7554,7553,7553,7550,7550,7547,7547,7527,7527,7525,7525,7502,7502,7487,7487,7479,7479,7469,7469,7433,7433,7424,7424,7409,7409,7396,7396,7395,7395,7393,7393,7391,7391,7390,7390,7383,7383,7381,7381,7374,7374,7373,7373,7371,7371,7359,7359,7357,7357,7356,7356,7349,7349,7341,7341,7338,7338,7335,7335,7334,7334,7333,7333,7328,7328,7318,7318,7304,7304,7275,7275,7255,7255,7238,7238,7228,7228,7205,7205,7198,7198,7195,7195,7192,7192,7191,7191,7180,7180,7174,7174,7158,7158,7143,7143,7142,7142,7135,7135,7133,7133,7121,7121,7119,7119,7107,7107,7102,7102,7099,7099,7081,7081,7080,7080,7079,7079,7076,7076,7072,7072,7070,7070,7063,7063,7061,7061,7054,7054,7049,7049,7017,7017,7010,7010,7006,7006,6983,6983,6980,6980,6979,6979,6976,6976,6962,6962,6960,6960,6958,6958,6952,6952,6926,6926,6922,6922,6912,6912,6872,6872,6843,6843,6828,6828,6822,6822,6813,6813,6790,6790,6759,6759,6744,6744,6734,6734,6729,6729,6725,6725,6702,6702,6698,6698,6696,6696,6695,6695,6687,6687,6680,6680,6667,6667,6652,6652,6650,6650,6645,6645,6639,6639,6631,6631,6630,6630,6624,6624,6615,6615,6603,6603,6602,6602,6597,6597,6585,6585,6583,6583,6569,6569,6566,6566,6553,6553,6551,6551,6544,6544,6534,6534,6530,6530,6529,6529,6515,6515,6495,6495,6490,6490,6477,6477,6473,6473,6469,6469,6467,6467,6459,6459,6456,6456,6440,6440,6438,6438,6431,6431,6421,6421,6419,6419,6397,6397,6393,6393,6388,6388,6384,6384,6381,6381,6378,6378,6349,6349,6348,6348,6338,6338,6328,6328,6323,6323,6320,6320,6319,6319,6313,6313,6309,6309,6307,6307,6291,6291,6281,6281,6279,6279,6278,6278,6275,6275,6273,6273,6272,6272,6263,6263,6259,6259,6257,6257,6243,6243,6237,6237,6234,6234,6214,6214,6198,6198,6187,6187,6183,6183,6182,6182,6179,6179,6178,6178,6177,6177,6166,6166,6155,6155,6153,6153,6139,6139,6133,6133,6126,6126,6124,6124,6123,6123,6113,6113,6098,6098,6090,6090,6074,6074,6073,6073,6072,6072,6066,6066,6065,6065,6058,6058,6056,6056,6048,6048,6043,6043,6041,6041,6028,6028,6025,6025,6023,6023,6022,6022,6017,6017,6015,6015,6012,6012,6008,6008,5986,5986,5975,5975,5974,5974,5971,5971,5970,5970,5966,5966,5963,5963,5959,5959,5952,5952,5947,5947,5932,5932,5929,5929,5928,5928,5924,5924,5916,5916,5894,5894,5854,5854,5852,5852,5823,5823,5813,5813,5808,5808,5804,5804,5802,5802,5801,5801,5796,5796,5783,5783,5762,5762,5757,5757,5723,5723,5714,5714,5711,5711,5701,5701,5695,5695,5693,5693,5689,5689,5688,5688,5684,5684,5683,5683,5678,5678,5677,5677,5669,5669,5668,5668,5667,5667,5644,5644,5643,5643,5642,5642,5624,5624,5618,5618,5603,5603,5586,5586,5583,5583,5576,5576,5567,5567,5559,5559,5558,5558,5555,5555,5552,5552,5540,5540,5539,5539,5538,5538,5536,5536,5535,5535,5533,5533,5532,5532,5530,5530,5521,5521,5520,5520,5517,5517,5506,5506,5502,5502,5501,5501,5500,5500,5479,5479,5473,5473,5470,5470,5458,5458,5457,5457,5456,5456,5442,5442,5433,5433,5427,5427,5423,5423,5416,5416,5413,5413,5411,5411,5408,5408,5406,5406,5394,5394,5387,5387,5359,5359,5356,5356,5343,5343,5337,5337,5333,5333,5310,5310,5305,5305,5300,5300,5286,5286,5284,5284,5272,5272,5260,5260,5229,5229,5211,5211,5199,5199,5195,5195,5189,5189,5188,5188,5178,5178,5169,5169,5154,5154,5153,5153,5151,5151,5150,5150,5148,5148,5144,5144,5136,5136,5132,5132,5099,5099,5095,5095,5094,5094,5086,5086,5082,5082,5072,5072,5068,5068,5060,5060,5056,5056,5051,5051,5026,5026,5022,5022,5020,5020,5015,5015,4992,4992,4984,4984,4981,4981,4967,4967,4951,4951,4945,4945,4933,4933,4916,4916,4897,4897,4885,4885,4871,4871,4870,4870,4861,4861,4849,4849,4845,4845,4829,4829,4816,4816,4807,4807,4798,4798,4774,4774,4772,4772,4745,4745,4724,4724,4695,4695,4667,4667,4661,4661,4657,4657,4643,4643,4615,4615,4612,4612,4600,4600,4596,4596,4595,4595,4591,4591,4588,4588,4586,4586,4572,4572,4566,4566,4563,4563,4557,4557,4546,4546,4541,4541,4535,4535,4533,4533,4518,4518,4512,4512,4492,4492,4488,4488,4486,4486,4477,4477,4476,4476,4472,4472,4462,4462,4453,4453,4451,4451,4441,4441,4438,4438,4436,4436,4433,4433,4426,4426,4414,4414,4410,4410,4403,4403,4393,4393,4388,4388,4357,4357,4349,4349,4344,4344,4326,4326,4324,4324,4320,4320,4303,4303,4286,4286,4285,4285,4284,4284,4279,4279,4278,4278,4259,4259,4258,4258,4238,4238,4232,4232,4224,4224,4215,4215,4213,4213,4208,4208,4186,4186,4183,4183,4170,4170,4167,4167,4162,4162,4158,4158,4156,4156,4145,4145,4125,4125,4121,4121,4119,4119,4114,4114,4111,4111,4110,4110,4108,4108,4096,4096,4086,4086,4059,4059,4032,4032,4031,4031,4017,4017,3993,3993,3969,3969,3967,3967,3960,3960,3952,3952,3951,3951,3948,3948,3938,3938,3936,3936,3932,3932,3931,3931,3924,3924,3922,3922,3921,3921,3917,3917,3909,3909,3901,3901,3894,3894,3893,3893,3885,3885,3875,3875,3871,3871,3857,3857,3832,3832,3827,3827,3823,3823,3814,3814,3813,3813,3804,3804,3797,3797,3796,3796,3781,3781,3771,3771,3769,3769,3758,3758,3756,3756,3739,3739,3729,3729,3728,3728,3699,3699,3697,3697,3694,3694,3691,3691,3679,3679,3674,3674,3673,3673,3661,3661,3659,3659,3657,3657,3656,3656,3653,3653,3651,3651,3642,3642,3639,3639,3622,3622,3618,3618,3616,3616,3615,3615,3607,3607,3602,3602,3598,3598,3596,3596,3592,3592,3590,3590,3582,3582,3551,3551,3544,3544,3532,3532,3515,3515,3514,3514,3503,3503,3502,3502,3496,3496,3486,3486,3462,3462,3459,3459,3458,3458,3449,3449,3447,3447,3443,3443,3428,3428,3420,3420,3416,3416,3388,3388,3383,3383,3382,3382,3380,3380,3379,3379,3375,3375,3368,3368,3358,3358,3356,3356,3351,3351,3350,3350,3349,3349,3348,3348,3334,3334,3331,3331,3326,3326,3310,3310,3307,3307,3299,3299,3282,3282,3281,3281,3278,3278,3268,3268,3266,3266,3265,3265,3260,3260,3254,3254,3246,3246,3239,3239,3221,3221,3219,3219,3216,3216,3208,3208,3206,3206,3203,3203,3194,3194,3185,3185,3182,3182,3173,3173,3171,3171,3169,3169,3167,3167,3158,3158,3152,3152,3148,3148,3145,3145,3144,3144,3140,3140,3139,3139,3136,3136,3135,3135,3116,3116,3112,3112,3099,3099,3098,3098,3094,3094,3088,3088,3079,3079,3069,3069,3059,3059,3053,3053,3052,3052,3042,3042,3038,3038,3032,3032,3028,3028,3027,3027,3026,3026,3017,3017,3009,3009,3003,3003,2999,2999,2997,2997,2994,2994,2991,2991,2987,2987,2985,2985,2981,2981,2974,2974,2944,2944,2935,2935,2932,2932,2920,2920,2909,2909,2903,2903,2892,2892,2891,2891,2888,2888,2878,2878,2874,2874,2873,2873,2872,2872,2868,2868,2867,2867,2847,2847,2846,2846,2840,2840,2839,2839,2815,2815,2811,2811,2803,2803,2802,2802,2795,2795,2792,2792,2790,2790,2777,2777,2776,2776,2772,2772,2771,2771,2761,2761,2760,2760,2749,2749,2748,2748,2747,2747,2743,2743,2728,2728,2721,2721,2720,2720,2715,2715,2711,2711,2704,2704,2691,2691,2688,2688,2685,2685,2684,2684,2679,2679,2678,2678,2674,2674,2662,2662,2657,2657,2652,2652,2650,2650,2647,2647,2628,2628,2620,2620,2612,2612,2611,2611,2608,2608,2605,2605,2594,2594,2589,2589,2582,2582,2580,2580,2577,2577,2572,2572,2569,2569,2562,2562,2561,2561,2553,2553,2552,2552,2550,2550,2536,2536,2533,2533,2522,2522,2521,2521,2520,2520,2518,2518,2512,2512,2506,2506,2505,2505,2504,2504,2500,2500,2499,2499,2498,2498,2497,2497,2492,2492,2491,2491,2488,2488,2486,2486,2476,2476,2475,2475,2474,2474,2473,2473,2468,2468,2461,2461,2460,2460,2455,2455,2453,2453,2451,2451,2445,2445,2442,2442,2441,2441,2439,2439,2431,2431,2411,2411,2407,2407,2406,2406,2405,2405,2403,2403,2401,2401,2398,2398,2392,2392,2391,2391,2388,2388,2383,2383,2377,2377,2371,2371,2367,2367,2366,2366,2363,2363,2362,2362,2352,2352,2344,2344,2338,2338,2334,2334,2329,2329,2326,2326,2321,2321,2316,2316,2315,2315,2314,2314,2309,2309,2295,2295,2293,2293,2289,2289,2281,2281,2278,2278,2272,2272,2268,2268,2266,2266,2261,2261,2260,2260,2254,2254,2250,2250,2249,2249,2248,2248,2242,2242,2234,2234,2226,2226,2224,2224,2218,2218,2208,2208,2207,2207,2201,2201,2198,2198,2186,2186,2183,2183,2178,2178,2175,2175,2169,2169,2161,2161,2156,2156,2151,2151,2147,2147,2143,2143,2142,2142,2131,2131,2122,2122,2121,2121,2120,2120,2109,2109,2108,2108,2104,2104,2097,2097,2088,2088,2087,2087,2076,2076,2074,2074,2072,2072,2060,2060,2057,2057,2050,2050,2046,2046,2044,2044,2042,2042,2041,2041,2039,2039,2038,2038,2035,2035,2031,2031,2029,2029,2028,2028,2027,2027,2026,2026,2021,2021,2020,2020,2015,2015,2012,2012,2011,2011,2008,2008,2007,2007,2004,2004,2003,2003,1985,1985,1981,1981,1980,1980,1978,1978,1977,1977,1975,1975,1971,1971,1967,1967,1964,1964,1962,1962,1958,1958,1953,1953,1947,1947,1946,1946,1941,1941,1939,1939,1938,1938,1937,1937,1925,1925,1923,1923,1920,1920,1910,1910,1909,1909,1907,1907,1904,1904,1901,1901,1898,1898,1897,1897,1891,1891,1887,1887,1885,1885,1883,1883,1882,1882,1877,1877,1876,1876,1870,1870,1867,1867,1864,1864,1850,1850,1849,1849,1844,1844,1842,1842,1841,1841,1840,1840,1835,1835,1834,1834,1828,1828,1826,1826,1825,1825,1817,1817,1811,1811,1808,1808,1807,1807,1804,1804,1803,1803,1802,1802,1799,1799,1797,1797,1791,1791,1790,1790,1789,1789,1785,1785,1784,1784,1780,1780,1776,1776,1775,1775,1772,1772,1767,1767,1763,1763,1762,1762,1753,1753,1752,1752,1746,1746,1745,1745,1743,1743,1733,1733,1731,1731,1726,1726,1725,1725,1724,1724,1722,1722,1714,1714,1705,1705,1703,1703,1702,1702,1701,1701,1700,1700,1698,1698,1697,1697,1695,1695,1693,1693,1691,1691,1690,1690,1686,1686,1684,1684,1683,1683,1682,1682,1678,1678,1676,1676,1675,1675,1671,1671,1670,1670,1666,1666,1665,1665,1663,1663,1661,1661,1658,1658,1657,1657,1652,1652,1648,1648,1647,1647,1645,1645,1630,1630,1629,1629,1625,1625,1623,1623,1621,1621,1618,1618,1601,1601,1598,1598,1597,1597,1594,1594,1582,1582,1580,1580,1578,1578,1577,1577,1576,1576,1575,1575,1573,1573,1572,1572,1561,1561,1558,1558,1555,1555,1552,1552,1549,1549,1548,1548,1547,1547,1543,1543,1542,1542,1541,1541,1540,1540,1538,1538,1537,1537,1535,1535,1531,1531,1530,1530,1525,1525,1524,1524,1523,1523,1516,1516,1512,1512,1504,1504,1501,1501,1498,1498,1495,1495,1493,1493,1491,1491,1483,1483,1479,1479,1477,1477,1474,1474,1472,1472,1465,1465,1460,1460,1459,1459,1457,1457,1456,1456,1449,1449,1447,1447,1446,1446,1438,1438,1437,1437,1434,1434,1433,1433,1429,1429,1428,1428,1424,1424,1419,1419,1418,1418,1417,1417,1416,1416,1415,1415,1412,1412,1409,1409,1404,1404,1399,1399,1397,1397,1396,1396,1393,1393,1388,1388,1387,1387,1384,1384,1383,1383,1378,1378,1377,1377,1366,1366,1363,1363,1362,1362,1361,1361,1358,1358,1352,1352,1351,1351,1350,1350,1347,1347,1345,1345,1342,1342,1340,1340,1339,1339,1338,1338,1337,1337,1329,1329,1328,1328,1322,1322,1318,1318,1315,1315,1306,1306,1303,1303,1294,1294,1292,1292,1290,1290,1288,1288,1285,1285,1281,1281,1277,1277,1275,1275,1274,1274,1267,1267,1266,1266,1265,1265,1264,1264,1263,1263,1261,1261,1259,1259,1257,1257,1254,1254,1253,1253,1251,1251,1248,1248,1242,1242,1238,1238,1234,1234,1233,1233,1230,1230,1227,1227,1224,1224,1223,1223,1222,1222,1221,1221,1220,1220,1218,1218,1217,1217,1214,1214,1210,1210,1208,1208,1207,1207,1206,1206,1205,1205,1204,1204,1203,1203,1200,1200,1197,1197,1191,1191,1190,1190,1187,1187,1184,1184,1181,1181,1175,1175,1174,1174,1170,1170,1168,1168,1166,1166,1164,1164,1160,1160,1159,1159,1158,1158,1156,1156,1155,1155,1154,1154,1151,1151,1150,1150,1146,1146,1145,1145,1139,1139,1136,1136,1135,1135,1132,1132,1130,1130,1129,1129,1122,1122,1121,1121,1118,1118,1117,1117,1115,1115,1112,1112,1108,1108,1104,1104,1102,1102,1092,1092,1091,1091,1089,1089,1087,1087,1086,1086,1084,1084,1082,1082,1081,1081,1080,1080,1077,1077,1076,1076,1074,1074,1073,1073,1067,1067,1065,1065,1063,1063,1059,1059,1054,1054,1053,1053,1051,1051,1048,1048,1046,1046,1044,1044,1043,1043,1042,1042,1041,1041,1040,1040,1035,1035,1033,1033,1032,1032,1031,1031,1030,1030,1028,1028,1026,1026,1025,1025,1024,1024,1023,1023,1022,1022,1021,1021,1019,1019,1018,1018,1017,1017,1016,1016,1015,1015,1014,1014,1011,1011,1010,1010,1007,1007,1005,1005,1004,1004,999,999,998,998,997,997,994,994,992,992,990,990,989,989,988,988,986,986,984,984,982,982,981,981,978,978,977,977,973,973,972,972,971,971,970,970,969,969,968,968,967,967,966,966,965,965,961,961,959,959,958,958,957,957,956,956,955,955,954,954,953,953,952,952,951,951,950,950,949,949,948,948,946,946,942,942,941,941,940,940,939,939,938,938,936,936,933,933,929,929,928,928,927,927,926,926,924,924,922,922,919,919,918,918,917,917,916,916,914,914,913,913,912,912,911,911,910,910,909,909,908,908,907,907,904,904,903,903,902,902,900,900,899,899,897,897,895,895,894,894,893,893,892,892,889,889,888,888,887,887,886,886,884,884,882,882,881,881,880,880,878,878,876,876,875,875,873,873,872,872,871,871,870,870,867,867,865,865,864,864,863,863,862,862,861,861,859,859,858,858,857,857,854,854,853,853,850,850,849,849,848,848,847,847,845,845,844,844,843,843,840,840,837,837,836,836,835,835,834,834,833,833,831,831,830,830,829,829,828,828,827,827,826,826,824,824,821,821,819,819,818,818,816,816,815,815,814,814,813,813,812,812,811,811,809,809,808,808,807,807,806,806,804,804,803,803,802,802,801,801,800,800,799,799,798,798,797,797,796,796,795,795,794,794,793,793,792,792,791,791,790,790,789,789,788,788,787,787,786,786,785,785,784,784,782,782,781,781,779,779,776,776,775,775,774,774,773,773,772,772,771,771,769,769,768,768,767,767,766,766,765,765,764,764,763,763,762,762,761,761,760,760,759,759,758,758,757,757,756,756,755,755,754,754,753,753,752,752,751,751,750,750,748,748,747,747,746,746,745,745,744,744,743,743,741,741,740,740,739,739,738,738,737,737,736,736,735,735,733,733,732,732,730,730,729,729,728,728,727,727,726,726,725,725,724,724,723,723,722,722,721,721,720,720,719,719,718,718,717,717,716,716,715,715,714,714,713,713,711,711,710,710,708,708,707,707,706,706,705,705,704,704,703,703,702,702,701,701,700,700,698,698,697,697,696,696,695,695,694,694,693,693,692,692,691,691,690,690,689,689,688,688,687,687,686,686,685,685,684,684,683,683,682,682,681,681,680,680,679,679,678,678,677,677,676,676,675,675,674,674,673,673,672,672,671,671,670,670,669,669,668,668,667,667,666,666,665,665,664,664,663,663,662,662,661,661,660,660,659,659,658,658,657,657,656,656,655,655,654,654,653,653,652,652,651,651,650,650,649,649,648,648,647,647,646,646,645,645,644,644,643,643,642,642,641,641,640,640,639,639,638,638,637,637,636,636,635,635,634,634,633,633,632,632,631,631,630,630,629,629,628,628,627,627,626,626,625,625,624,624,623,623,622,622,621,621,620,620,619,619,617,617,616,616,615,615,613,613,611,611,609,609,608,608,607,607,601,601,599,599,598,598,596,596,595,595,593,593,591,591,590,590,589,589,588,588,587,587,584,584,583,583,582,582,581,581,576,576,575,575,573,573,572,572,571,571,570,570,568,568,567,567,564,564,562,562,561,561,560,560,558,558,555,555,554,554,552,552,551,551,547,547,546,546,544,544,542,542,540,540,539,539,537,537,534,534,531,531,530,530,529,529,528,528,527,527,525,525,523,523,522,522,520,520,519,519,516,516,513,513,512,512,511,511,509,509,494,494,486,486,482,482,480,480,475,475,472,472,469,469,457,457,453,453,452,452,445,445,431,431,430,430,424,424,422,422,413,413,412,412,410,410,403,403,0.0],[422176,422176,422176,267682,267682,223384,223384,218535,218535,198367,198367,181933,181933,179084,179084,175245,175245,155784,155784,154599,154599,152033,152033,149358,149358,144827,144827,143493,143493,141354,141354,141268,141268,139546,139546,139245,139245,135878,135878,135578,135578,133590,133590,131144,131144,130984,130984,127794,127794,126413,126413,125676,125676,122869,122869,121634,121634,120296,120296,120077,120077,118729,118729,117926,117926,114179,114179,113203,113203,111768,111768,111224,111224,109605,109605,109331,109331,105649,105649,104421,104421,104344,104344,103682,103682,103493,103493,101797,101797,101710,101710,101525,101525,101495,101495,100169,100169,100083,100083,99650,99650,99441,99441,98763,98763,98555,98555,97753,97753,95652,95652,94992,94992,93587,93587,93521,93521,92259,92259,90122,90122,89968,89968,86218,86218,85406,85406,84936,84936,84186,84186,83997,83997,83963,83963,83837,83837,83568,83568,82538,82538,80031,80031,79894,79894,79844,79844,79114,79114,79078,79078,79038,79038,78800,78800,78763,78763,78421,78421,77718,77718,77495,77495,77200,77200,77142,77142,77131,77131,77086,77086,77002,77002,76915,76915,76820,76820,76715,76715,76476,76476,75524,75524,75476,75476,74473,74473,74419,74419,74219,74219,73466,73466,73433,73433,72840,72840,71804,71804,70733,70733,70485,70485,69938,69938,69933,69933,69766,69766,69589,69589,68597,68597,68363,68363,68119,68119,68074,68074,68010,68010,67453,67453,67113,67113,66804,66804,66429,66429,66301,66301,66243,66243,65926,65926,65351,65351,65323,65323,65274,65274,65067,65067,65034,65034,64851,64851,64628,64628,64601,64601,64579,64579,64474,64474,64429,64429,63290,63290,63061,63061,62888,62888,62563,62563,62177,62177,62150,62150,62147,62147,61962,61962,61208,61208,61106,61106,60757,60757,60359,60359,60221,60221,59479,59479,58924,58924,58529,58529,57765,57765,57591,57591,57578,57578,57215,57215,56987,56987,56799,56799,56644,56644,56033,56033,55913,55913,55848,55848,55451,55451,55149,55149,54726,54726,54417,54417,54027,54027,54018,54018,53866,53866,53599,53599,53557,53557,53419,53419,53255,53255,52390,52390,52369,52369,52331,52331,52115,52115,52047,52047,51723,51723,51684,51684,51400,51400,50944,50944,50776,50776,50557,50557,50407,50407,50000,50000,49944,49944,49882,49882,49772,49772,49173,49173,48827,48827,48651,48651,48618,48618,48441,48441,48390,48390,48187,48187,48157,48157,47826,47826,47606,47606,47171,47171,47048,47048,47019,47019,46960,46960,46926,46926,46862,46862,46834,46834,46682,46682,46375,46375,45986,45986,45872,45872,45663,45663,45591,45591,45529,45529,45471,45471,45318,45318,45280,45280,45252,45252,44874,44874,44792,44792,44728,44728,44715,44715,44186,44186,44142,44142,43519,43519,42986,42986,42519,42519,42413,42413,42272,42272,42264,42264,41952,41952,41865,41865,41079,41079,41060,41060,41059,41059,41011,41011,40898,40898,40842,40842,40786,40786,40744,40744,40686,40686,40556,40556,40340,40340,40188,40188,40086,40086,40069,40069,40058,40058,39943,39943,39765,39765,39593,39593,39391,39391,39255,39255,39232,39232,39208,39208,38733,38733,38721,38721,38659,38659,38481,38481,38234,38234,38230,38230,38196,38196,38029,38029,37919,37919,37918,37918,37749,37749,37567,37567,37503,37503,37451,37451,37209,37209,37184,37184,36988,36988,36864,36864,36843,36843,36645,36645,36610,36610,36583,36583,36479,36479,36391,36391,36378,36378,36274,36274,36079,36079,35793,35793,35738,35738,35734,35734,35658,35658,35372,35372,34832,34832,34801,34801,34640,34640,34591,34591,34366,34366,34202,34202,34086,34086,34045,34045,33917,33917,33833,33833,33767,33767,33765,33765,33741,33741,33555,33555,33532,33532,33516,33516,33390,33390,33317,33317,33300,33300,33086,33086,32946,32946,32826,32826,32796,32796,32412,32412,32360,32360,32282,32282,31988,31988,31828,31828,31724,31724,31689,31689,31676,31676,31670,31670,31643,31643,31410,31410,31273,31273,31123,31123,31114,31114,31073,31073,31015,31015,30869,30869,30841,30841,30592,30592,30548,30548,30494,30494,30460,30460,30414,30414,30402,30402,30203,30203,30177,30177,30126,30126,30043,30043,30037,30037,29991,29991,29876,29876,29861,29861,29752,29752,29645,29645,29529,29529,29524,29524,29422,29422,29383,29383,29260,29260,29250,29250,29163,29163,29137,29137,29136,29136,28927,28927,28662,28662,28562,28562,28500,28500,28494,28494,28478,28478,28333,28333,28330,28330,28210,28210,28204,28204,28188,28188,28157,28157,28085,28085,28053,28053,28025,28025,27922,27922,27919,27919,27879,27879,27801,27801,27785,27785,27769,27769,27734,27734,27628,27628,27533,27533,27454,27454,27448,27448,27408,27408,27264,27264,27260,27260,27233,27233,27170,27170,27156,27156,27113,27113,27044,27044,26932,26932,26892,26892,26815,26815,26631,26631,26566,26566,26539,26539,26502,26502,26472,26472,26470,26470,26320,26320,26318,26318,26308,26308,26264,26264,26220,26220,26043,26043,25878,25878,25791,25791,25732,25732,25659,25659,25607,25607,25534,25534,25493,25493,25482,25482,25459,25459,25385,25385,25293,25293,25283,25283,25199,25199,25186,25186,25102,25102,25060,25060,25023,25023,24984,24984,24973,24973,24724,24724,24624,24624,24615,24615,24593,24593,24548,24548,24501,24501,24487,24487,24183,24183,24173,24173,24078,24078,24009,24009,24007,24007,23955,23955,23894,23894,23757,23757,23715,23715,23616,23616,23579,23579,23463,23463,23402,23402,23359,23359,23192,23192,23170,23170,23141,23141,23108,23108,23063,23063,23029,23029,22883,22883,22811,22811,22796,22796,22791,22791,22722,22722,22694,22694,22568,22568,22380,22380,22252,22252,22250,22250,22234,22234,22231,22231,22100,22100,22083,22083,22070,22070,22067,22067,22028,22028,21973,21973,21907,21907,21878,21878,21828,21828,21634,21634,21610,21610,21553,21553,21521,21521,21396,21396,21214,21214,21114,21114,21093,21093,21092,21092,21051,21051,20972,20972,20864,20864,20697,20697,20602,20602,20488,20488,20467,20467,20438,20438,20413,20413,20399,20399,20389,20389,20309,20309,20305,20305,20227,20227,20206,20206,20191,20191,20177,20177,20139,20139,20110,20110,19966,19966,19850,19850,19844,19844,19831,19831,19767,19767,19721,19721,19679,19679,19552,19552,19406,19406,19331,19331,19289,19289,19257,19257,19141,19141,19033,19033,19007,19007,18987,18987,18867,18867,18813,18813,18608,18608,18550,18550,18438,18438,18406,18406,18378,18378,18368,18368,18366,18366,18348,18348,18231,18231,18216,18216,18096,18096,17926,17926,17898,17898,17873,17873,17827,17827,17817,17817,17803,17803,17775,17775,17764,17764,17754,17754,17738,17738,17722,17722,17704,17704,17697,17697,17665,17665,17659,17659,17631,17631,17560,17560,17519,17519,17455,17455,17447,17447,17406,17406,17375,17375,17366,17366,17297,17297,17069,17069,17019,17019,16924,16924,16897,16897,16888,16888,16853,16853,16766,16766,16731,16731,16694,16694,16676,16676,16598,16598,16593,16593,16585,16585,16461,16461,16436,16436,16424,16424,16353,16353,16342,16342,16335,16335,16197,16197,16159,16159,16071,16071,16068,16068,15955,15955,15937,15937,15904,15904,15846,15846,15825,15825,15811,15811,15782,15782,15718,15718,15690,15690,15664,15664,15606,15606,15582,15582,15578,15578,15544,15544,15540,15540,15437,15437,15403,15403,15384,15384,15325,15325,15212,15212,15136,15136,15125,15125,15095,15095,15078,15078,15066,15066,15030,15030,15016,15016,14948,14948,14937,14937,14927,14927,14795,14795,14774,14774,14762,14762,14724,14724,14701,14701,14642,14642,14636,14636,14632,14632,14619,14619,14618,14618,14517,14517,14493,14493,14479,14479,14475,14475,14424,14424,14411,14411,14406,14406,14375,14375,14322,14322,14310,14310,14290,14290,14247,14247,14113,14113,14112,14112,14056,14056,13973,13973,13935,13935,13871,13871,13846,13846,13827,13827,13795,13795,13742,13742,13669,13669,13642,13642,13628,13628,13618,13618,13567,13567,13528,13528,13460,13460,13439,13439,13315,13315,13311,13311,13288,13288,13277,13277,13273,13273,13247,13247,13167,13167,13157,13157,13154,13154,13136,13136,13115,13115,13061,13061,13028,13028,13010,13010,13001,13001,12983,12983,12977,12977,12957,12957,12893,12893,12879,12879,12858,12858,12854,12854,12848,12848,12842,12842,12797,12797,12796,12796,12738,12738,12663,12663,12638,12638,12632,12632,12631,12631,12614,12614,12604,12604,12493,12493,12480,12480,12424,12424,12358,12358,12322,12322,12310,12310,12306,12306,12303,12303,12272,12272,12264,12264,12253,12253,12229,12229,12220,12220,12200,12200,12194,12194,12165,12165,12147,12147,12130,12130,12056,12056,12044,12044,12042,12042,12020,12020,11996,11996,11951,11951,11950,11950,11916,11916,11795,11795,11791,11791,11747,11747,11539,11539,11525,11525,11487,11487,11451,11451,11389,11389,11378,11378,11371,11371,11347,11347,11342,11342,11252,11252,11215,11215,11201,11201,11189,11189,11119,11119,11061,11061,11032,11032,10994,10994,10958,10958,10948,10948,10941,10941,10909,10909,10899,10899,10892,10892,10873,10873,10839,10839,10822,10822,10812,10812,10781,10781,10768,10768,10737,10737,10666,10666,10643,10643,10628,10628,10585,10585,10556,10556,10461,10461,10428,10428,10386,10386,10372,10372,10341,10341,10324,10324,10313,10313,10295,10295,10278,10278,10185,10185,10136,10136,10013,10013,9964,9964,9959,9959,9879,9879,9840,9840,9828,9828,9813,9813,9806,9806,9753,9753,9702,9702,9599,9599,9582,9582,9558,9558,9552,9552,9551,9551,9550,9550,9502,9502,9494,9494,9447,9447,9438,9438,9405,9405,9384,9384,9343,9343,9329,9329,9247,9247,9230,9230,9224,9224,9140,9140,9129,9129,9127,9127,9111,9111,9104,9104,9057,9057,9039,9039,9027,9027,9017,9017,8999,8999,8984,8984,8979,8979,8952,8952,8942,8942,8940,8940,8909,8909,8890,8890,8889,8889,8879,8879,8841,8841,8787,8787,8779,8779,8773,8773,8768,8768,8739,8739,8691,8691,8684,8684,8682,8682,8670,8670,8645,8645,8583,8583,8572,8572,8555,8555,8545,8545,8536,8536,8498,8498,8459,8459,8454,8454,8422,8422,8348,8348,8345,8345,8324,8324,8323,8323,8312,8312,8294,8294,8285,8285,8266,8266,8262,8262,8212,8212,8209,8209,8175,8175,8171,8171,8147,8147,8137,8137,8135,8135,8113,8113,8093,8093,8062,8062,8050,8050,8008,8008,7934,7934,7913,7913,7864,7864,7831,7831,7830,7830,7825,7825,7815,7815,7730,7730,7719,7719,7711,7711,7700,7700,7689,7689,7687,7687,7663,7663,7658,7658,7650,7650,7639,7639,7623,7623,7597,7597,7570,7570,7553,7553,7547,7547,7527,7527,7502,7502,7479,7479,7424,7424,7396,7396,7393,7393,7391,7391,7381,7381,7357,7357,7349,7349,7335,7335,7334,7334,7333,7333,7309,7309,7275,7275,7205,7205,7198,7198,7180,7180,7133,7133,7107,7107,7099,7099,7079,7079,7072,7072,7063,7063,7062,7062,7054,7054,7049,7049,7017,7017,7010,7010,7006,7006,6995,6995,6980,6980,6979,6979,6960,6960,6958,6958,6926,6926,6828,6828,6813,6813,6790,6790,6753,6753,6744,6744,6729,6729,6708,6708,6702,6702,6698,6698,6687,6687,6639,6639,6602,6602,6597,6597,6585,6585,6551,6551,6544,6544,6534,6534,6529,6529,6524,6524,6515,6515,6513,6513,6495,6495,6477,6477,6473,6473,6459,6459,6421,6421,6384,6384,6380,6380,6338,6338,6320,6320,6309,6309,6289,6289,6275,6275,6273,6273,6263,6263,6257,6257,6243,6243,6155,6155,6139,6139,6126,6126,6124,6124,6123,6123,6073,6073,6056,6056,6048,6048,6043,6043,6017,6017,6015,6015,6008,6008,5966,5966,5932,5932,5929,5929,5916,5916,5911,5911,5854,5854,5852,5852,5823,5823,5808,5808,5796,5796,5779,5779,5778,5778,5695,5695,5688,5688,5684,5684,5669,5669,5667,5667,5644,5644,5642,5642,5624,5624,5618,5618,5555,5555,5540,5540,5538,5538,5520,5520,5501,5501,5491,5491,5473,5473,5458,5458,5457,5457,5456,5456,5439,5439,5416,5416,5411,5411,5406,5406,5333,5333,5229,5229,5209,5209,5199,5199,5188,5188,5178,5178,5154,5154,5153,5153,5150,5150,5144,5144,5136,5136,5134,5134,5095,5095,5094,5094,5072,5072,5068,5068,5060,5060,5056,5056,5054,5054,5051,5051,5043,5043,5022,5022,5015,5015,4992,4992,4951,4951,4931,4931,4849,4849,4829,4829,4816,4816,4772,4772,4745,4745,4695,4695,4657,4657,4622,4622,4615,4615,4596,4596,4595,4595,4588,4588,4572,4572,4563,4563,4541,4541,4533,4533,4518,4518,4488,4488,4477,4477,4462,4462,4451,4451,4443,4443,4441,4441,4438,4438,4433,4433,4429,4429,4427,4427,4426,4426,4410,4410,4403,4403,4393,4393,4324,4324,4320,4320,4303,4303,4286,4286,4285,4285,4238,4238,4232,4232,4209,4209,4200,4200,4186,4186,4183,4183,4170,4170,4168,4168,4164,4164,4156,4156,4111,4111,4017,4017,3980,3980,3955,3955,3948,3948,3931,3931,3928,3928,3909,3909,3894,3894,3891,3891,3888,3888,3857,3857,3827,3827,3823,3823,3781,3781,3771,3771,3769,3769,3739,3739,3728,3728,3727,3727,3716,3716,3699,3699,3697,3697,3684,3684,3679,3679,3659,3659,3657,3657,3656,3656,3642,3642,3639,3639,3615,3615,3596,3596,3592,3592,3583,3583,3582,3582,3539,3539,3514,3514,3512,3512,3496,3496,3449,3449,3411,3411,3386,3386,3375,3375,3370,3370,3368,3368,3363,3363,3358,3358,3351,3351,3350,3350,3334,3334,3326,3326,3317,3317,3310,3310,3309,3309,3307,3307,3278,3278,3265,3265,3260,3260,3257,3257,3254,3254,3239,3239,3219,3219,3203,3203,3171,3171,3169,3169,3168,3168,3167,3167,3150,3150,3140,3140,3135,3135,3127,3127,3112,3112,3107,3107,3098,3098,3088,3088,3059,3059,3038,3038,3032,3032,3017,3017,2997,2997,2970,2970,2935,2935,2932,2932,2920,2920,2896,2896,2875,2875,2874,2874,2870,2870,2868,2868,2851,2851,2847,2847,2840,2840,2803,2803,2802,2802,2792,2792,2777,2777,2774,2774,2747,2747,2729,2729,2722,2722,2721,2721,2720,2720,2715,2715,2698,2698,2691,2691,2685,2685,2657,2657,2620,2620,2611,2611,2582,2582,2580,2580,2577,2577,2569,2569,2552,2552,2538,2538,2530,2530,2524,2524,2521,2521,2519,2519,2512,2512,2497,2497,2494,2494,2493,2493,2486,2486,2478,2478,2477,2477,2461,2461,2455,2455,2453,2453,2445,2445,2444,2444,2439,2439,2419,2419,2411,2411,2402,2402,2398,2398,2383,2383,2367,2367,2358,2358,2337,2337,2315,2315,2268,2268,2266,2266,2265,2265,2264,2264,2256,2256,2218,2218,2198,2198,2193,2193,2183,2183,2178,2178,2170,2170,2143,2143,2131,2131,2093,2093,2087,2087,2081,2081,2076,2076,2058,2058,2057,2057,2051,2051,2050,2050,2046,2046,2042,2042,2041,2041,2039,2039,2038,2038,2028,2028,2021,2021,2011,2011,2008,2008,2007,2007,1998,1998,1992,1992,1977,1977,1971,1971,1964,1964,1962,1962,1958,1958,1953,1953,1947,1947,1938,1938,1928,1928,1921,1921,1920,1920,1919,1919,1909,1909,1896,1896,1891,1891,1887,1887,1886,1886,1885,1885,1876,1876,1873,1873,1870,1870,1867,1867,1849,1849,1848,1848,1844,1844,1841,1841,1840,1840,1828,1828,1826,1826,1805,1805,1804,1804,1803,1803,1802,1802,1799,1799,1798,1798,1789,1789,1784,1784,1780,1780,1775,1775,1772,1772,1769,1769,1767,1767,1763,1763,1762,1762,1759,1759,1748,1748,1743,1743,1731,1731,1724,1724,1721,1721,1714,1714,1705,1705,1704,1704,1703,1703,1700,1700,1697,1697,1696,1696,1694,1694,1693,1693,1691,1691,1682,1682,1680,1680,1678,1678,1671,1671,1666,1666,1663,1663,1658,1658,1657,1657,1656,1656,1655,1655,1652,1652,1648,1648,1641,1641,1640,1640,1639,1639,1635,1635,1633,1633,1629,1629,1623,1623,1621,1621,1616,1616,1613,1613,1610,1610,1608,1608,1603,1603,1594,1594,1589,1589,1588,1588,1584,1584,1578,1578,1577,1577,1567,1567,1564,1564,1561,1561,1558,1558,1552,1552,1548,1548,1541,1541,1533,1533,1532,1532,1530,1530,1525,1525,1524,1524,1519,1519,1517,1517,1516,1516,1510,1510,1502,1502,1501,1501,1498,1498,1491,1491,1486,1486,1481,1481,1479,1479,1475,1475,1462,1462,1460,1460,1456,1456,1449,1449,1445,1445,1438,1438,1434,1434,1433,1433,1426,1426,1418,1418,1417,1417,1412,1412,1409,1409,1402,1402,1393,1393,1388,1388,1384,1384,1383,1383,1379,1379,1362,1362,1361,1361,1350,1350,1344,1344,1343,1343,1338,1338,1335,1335,1331,1331,1330,1330,1329,1329,1313,1313,1309,1309,1303,1303,1302,1302,1296,1296,1292,1292,1283,1283,1267,1267,1266,1266,1264,1264,1261,1261,1260,1260,1259,1259,1252,1252,1243,1243,1234,1234,1233,1233,1230,1230,1224,1224,1221,1221,1220,1220,1218,1218,1217,1217,1209,1209,1194,1194,1193,1193,1188,1188,1184,1184,1182,1182,1179,1179,1178,1178,1175,1175,1174,1174,1155,1155,1154,1154,1152,1152,1150,1150,1147,1147,1145,1145,1139,1139,1136,1136,1130,1130,1129,1129,1122,1122,1115,1115,1113,1113,1112,1112,1110,1110,1108,1108,1106,1106,1096,1096,1093,1093,1092,1092,1087,1087,1084,1084,1082,1082,1081,1081,1079,1079,1075,1075,1073,1073,1072,1072,1071,1071,1069,1069,1063,1063,1060,1060,1052,1052,1051,1051,1048,1048,1046,1046,1044,1044,1043,1043,1040,1040,1037,1037,1035,1035,1031,1031,1024,1024,1018,1018,1017,1017,1016,1016,1011,1011,1010,1010,1008,1008,1002,1002,1000,1000,997,997,992,992,989,989,988,988,986,986,984,984,983,983,982,982,980,980,975,975,969,969,968,968,965,965,964,964,955,955,954,954,952,952,950,950,949,949,947,947,944,944,942,942,938,938,936,936,935,935,933,933,931,931,928,928,926,926,919,919,917,917,916,916,913,913,909,909,907,907,903,903,897,897,892,892,891,891,888,888,886,886,884,884,881,881,879,879,878,878,876,876,874,874,869,869,861,861,859,859,857,857,856,856,848,848,846,846,844,844,843,843,841,841,840,840,839,839,834,834,833,833,829,829,826,826,824,824,821,821,819,819,817,817,811,811,809,809,802,802,801,801,800,800,799,799,797,797,795,795,790,790,789,789,786,786,785,785,782,782,777,777,775,775,773,773,767,767,766,766,758,758,757,757,754,754,751,751,750,750,748,748,747,747,746,746,745,745,743,743,742,742,738,738,736,736,735,735,734,734,730,730,729,729,728,728,721,721,718,718,716,716,715,715,714,714,702,702,701,701,696,696,694,694,685,685,684,684,681,681,680,680,674,674,672,672,671,671,669,669,668,668,667,667,665,665,662,662,658,658,657,657,656,656,655,655,649,649,647,647,646,646,645,645,644,644,643,643,642,642,640,640,639,639,638,638,636,636,634,634,632,632,631,631,629,629,622,622,618,618,615,615,614,614,613,613,608,608,607,607,606,606,604,604,603,603,600,600,597,597,596,596,595,595,593,593,590,590,589,589,588,588,587,587,586,586,583,583,582,582,581,581,580,580,578,578,577,577,576,576,575,575,574,574,572,572,571,571,570,570,569,569,568,568,567,567,565,565,564,564,563,563,562,562,561,561,558,558,557,557,555,555,552,552,551,551,549,549,547,547,546,546,544,544,539,539,537,537,536,536,535,535,534,534,533,533,532,532,531,531,530,530,528,528,524,524,523,523,522,522,519,519,517,517,516,516,514,514,503,503,492,492,485,485,474,474,452,452,448,448,438,438,435,435,434,434,428,428,423,423,421,421,417,417,414,414,411,411,405,405,0.0],[279110,279110,279110,216386,216386,209534,209534,176309,176309,176264,176264,168662,168662,158836,158836,153922,153922,152912,152912,146402,146402,133949,133949,130297,130297,130117,130117,124229,124229,121442,121442,120371,120371,118878,118878,118800,118800,118545,118545,116707,116707,108492,108492,107134,107134,104532,104532,102212,102212,101870,101870,101516,101516,99467,99467,98979,98979,96616,96616,93428,93428,90675,90675,88974,88974,87285,87285,87189,87189,86921,86921,86401,86401,85957,85957,84370,84370,84141,84141,83948,83948,83038,83038,82863,82863,82519,82519,81695,81695,81670,81670,80691,80691,80440,80440,78735,78735,77566,77566,77262,77262,77041,77041,76650,76650,76612,76612,76410,76410,75682,75682,74289,74289,73777,73777,73699,73699,73677,73677,73523,73523,72639,72639,72443,72443,72081,72081,71972,71972,71433,71433,71072,71072,70907,70907,69838,69838,69650,69650,69552,69552,69499,69499,69424,69424,69134,69134,68715,68715,68565,68565,68327,68327,68269,68269,67670,67670,66407,66407,65392,65392,65212,65212,65094,65094,64143,64143,64103,64103,63958,63958,63819,63819,63345,63345,62867,62867,62809,62809,62488,62488,62017,62017,61931,61931,61898,61898,61681,61681,60420,60420,60041,60041,59774,59774,59729,59729,59712,59712,59536,59536,59477,59477,59471,59471,59235,59235,57910,57910,57554,57554,57093,57093,56666,56666,56408,56408,56359,56359,55546,55546,55353,55353,54412,54412,54348,54348,53976,53976,53781,53781,53701,53701,53044,53044,52762,52762,52751,52751,52665,52665,52646,52646,52214,52214,52004,52004,51955,51955,51820,51820,51668,51668,51384,51384,51104,51104,50845,50845,50527,50527,50322,50322,50169,50169,50115,50115,50004,50004,49554,49554,49432,49432,49176,49176,49102,49102,48582,48582,48324,48324,48247,48247,47707,47707,47608,47608,47401,47401,47376,47376,47367,47367,46875,46875,46849,46849,46545,46545,46516,46516,46064,46064,45868,45868,45810,45810,45743,45743,45590,45590,45507,45507,45285,45285,45145,45145,44958,44958,44523,44523,44200,44200,43749,43749,43406,43406,43224,43224,43124,43124,42880,42880,42878,42878,42750,42750,42729,42729,42666,42666,42662,42662,42452,42452,42443,42443,41997,41997,41845,41845,41588,41588,41582,41582,41522,41522,41450,41450,41447,41447,41355,41355,41297,41297,41082,41082,41028,41028,40856,40856,40483,40483,40163,40163,40101,40101,39572,39572,39482,39482,39434,39434,39251,39251,39217,39217,39201,39201,39070,39070,38954,38954,38938,38938,38927,38927,38851,38851,38837,38837,38737,38737,38701,38701,38582,38582,38575,38575,38521,38521,38447,38447,38419,38419,38177,38177,38041,38041,38006,38006,37915,37915,37849,37849,37634,37634,37548,37548,37451,37451,37375,37375,37366,37366,37320,37320,37276,37276,37177,37177,37176,37176,37175,37175,37072,37072,37044,37044,37041,37041,36957,36957,36942,36942,36823,36823,36560,36560,36552,36552,36307,36307,35805,35805,35399,35399,35208,35208,35197,35197,35157,35157,34987,34987,34624,34624,34561,34561,34496,34496,34430,34430,34420,34420,34340,34340,34222,34222,34184,34184,34157,34157,33998,33998,33950,33950,33911,33911,33868,33868,33856,33856,33446,33446,33403,33403,33395,33395,33377,33377,33373,33373,33357,33357,33353,33353,33338,33338,33252,33252,33143,33143,33048,33048,32946,32946,32918,32918,32865,32865,32861,32861,32835,32835,32821,32821,32670,32670,32492,32492,32385,32385,32366,32366,32318,32318,32079,32079,32064,32064,32051,32051,32034,32034,31895,31895,31781,31781,31737,31737,31633,31633,31561,31561,31519,31519,31499,31499,31409,31409,31215,31215,31063,31063,31004,31004,30979,30979,30872,30872,30743,30743,30570,30570,30564,30564,30541,30541,30527,30527,30514,30514,30505,30505,30310,30310,30299,30299,30183,30183,30065,30065,29780,29780,29714,29714,29686,29686,29573,29573,29545,29545,29529,29529,29455,29455,29405,29405,29387,29387,29262,29262,29222,29222,29163,29163,29073,29073,29070,29070,28888,28888,28887,28887,28849,28849,28838,28838,28814,28814,28756,28756,28580,28580,28517,28517,28436,28436,28310,28310,28234,28234,28191,28191,28187,28187,28100,28100,27996,27996,27982,27982,27947,27947,27794,27794,27706,27706,27611,27611,27429,27429,27406,27406,27377,27377,27297,27297,27234,27234,27219,27219,27213,27213,27172,27172,27170,27170,27124,27124,26963,26963,26874,26874,26864,26864,26819,26819,26685,26685,26682,26682,26648,26648,26538,26538,26363,26363,26228,26228,26221,26221,26163,26163,26131,26131,26071,26071,26025,26025,26011,26011,25934,25934,25880,25880,25810,25810,25806,25806,25772,25772,25770,25770,25750,25750,25708,25708,25678,25678,25537,25537,25513,25513,25506,25506,25494,25494,25482,25482,25237,25237,25224,25224,25207,25207,25187,25187,25123,25123,25108,25108,25049,25049,24908,24908,24901,24901,24799,24799,24743,24743,24740,24740,24684,24684,24680,24680,24678,24678,24659,24659,24547,24547,24475,24475,24231,24231,24188,24188,24051,24051,23921,23921,23866,23866,23851,23851,23848,23848,23842,23842,23829,23829,23703,23703,23694,23694,23630,23630,23627,23627,23611,23611,23599,23599,23560,23560,23532,23532,23501,23501,23463,23463,23452,23452,23339,23339,23293,23293,23277,23277,23254,23254,23243,23243,23135,23135,23067,23067,22896,22896,22893,22893,22844,22844,22775,22775,22724,22724,22677,22677,22656,22656,22549,22549,22514,22514,22501,22501,22460,22460,22448,22448,22444,22444,22366,22366,22344,22344,22254,22254,22224,22224,22211,22211,22200,22200,22100,22100,22094,22094,21913,21913,21893,21893,21715,21715,21705,21705,21703,21703,21687,21687,21683,21683,21679,21679,21676,21676,21613,21613,21593,21593,21569,21569,21454,21454,21452,21452,21448,21448,21430,21430,21427,21427,21406,21406,21391,21391,21356,21356,21262,21262,21249,21249,21214,21214,21112,21112,21067,21067,21060,21060,21022,21022,20990,20990,20977,20977,20845,20845,20820,20820,20729,20729,20710,20710,20681,20681,20676,20676,20591,20591,20583,20583,20579,20579,20577,20577,20538,20538,20490,20490,20468,20468,20461,20461,20426,20426,20420,20420,20372,20372,20339,20339,20334,20334,20229,20229,20228,20228,20167,20167,20143,20143,20136,20136,20125,20125,20112,20112,20042,20042,20039,20039,20033,20033,20017,20017,19974,19974,19961,19961,19947,19947,19941,19941,19913,19913,19890,19890,19817,19817,19814,19814,19812,19812,19811,19811,19738,19738,19719,19719,19682,19682,19678,19678,19669,19669,19600,19600,19577,19577,19574,19574,19554,19554,19536,19536,19422,19422,19415,19415,19290,19290,19287,19287,19185,19185,19132,19132,19032,19032,19011,19011,19006,19006,18989,18989,18985,18985,18973,18973,18922,18922,18900,18900,18898,18898,18883,18883,18851,18851,18813,18813,18802,18802,18772,18772,18768,18768,18752,18752,18732,18732,18675,18675,18608,18608,18569,18569,18565,18565,18559,18559,18545,18545,18503,18503,18459,18459,18412,18412,18393,18393,18369,18369,18360,18360,18351,18351,18323,18323,18318,18318,18309,18309,18307,18307,18298,18298,18260,18260,18225,18225,18223,18223,18209,18209,18176,18176,18172,18172,18171,18171,18109,18109,18108,18108,18106,18106,18090,18090,18060,18060,18053,18053,18049,18049,18034,18034,18009,18009,17986,17986,17976,17976,17941,17941,17932,17932,17885,17885,17868,17868,17848,17848,17829,17829,17765,17765,17716,17716,17667,17667,17647,17647,17617,17617,17616,17616,17590,17590,17528,17528,17520,17520,17512,17512,17439,17439,17405,17405,17400,17400,17398,17398,17379,17379,17359,17359,17328,17328,17302,17302,17280,17280,17272,17272,17269,17269,17245,17245,17223,17223,17186,17186,17061,17061,16998,16998,16984,16984,16950,16950,16927,16927,16813,16813,16799,16799,16787,16787,16780,16780,16767,16767,16765,16765,16732,16732,16724,16724,16711,16711,16671,16671,16665,16665,16593,16593,16569,16569,16533,16533,16464,16464,16454,16454,16443,16443,16439,16439,16429,16429,16368,16368,16366,16366,16325,16325,16287,16287,16223,16223,16182,16182,16164,16164,16149,16149,16096,16096,16063,16063,16016,16016,15955,15955,15951,15951,15948,15948,15937,15937,15890,15890,15885,15885,15860,15860,15858,15858,15841,15841,15834,15834,15798,15798,15757,15757,15726,15726,15713,15713,15702,15702,15691,15691,15663,15663,15628,15628,15619,15619,15599,15599,15530,15530,15529,15529,15517,15517,15491,15491,15455,15455,15412,15412,15404,15404,15382,15382,15365,15365,15356,15356,15342,15342,15331,15331,15307,15307,15279,15279,15266,15266,15259,15259,15233,15233,15223,15223,15201,15201,15183,15183,15181,15181,15144,15144,15110,15110,15098,15098,15047,15047,14944,14944,14943,14943,14937,14937,14902,14902,14834,14834,14734,14734,14710,14710,14671,14671,14664,14664,14612,14612,14578,14578,14489,14489,14409,14409,14356,14356,14322,14322,14309,14309,14256,14256,14241,14241,14132,14132,14116,14116,14106,14106,14102,14102,14077,14077,14076,14076,14070,14070,14048,14048,14047,14047,14036,14036,14015,14015,13969,13969,13891,13891,13873,13873,13847,13847,13799,13799,13736,13736,13726,13726,13699,13699,13690,13690,13669,13669,13665,13665,13664,13664,13657,13657,13645,13645,13606,13606,13558,13558,13556,13556,13500,13500,13493,13493,13486,13486,13473,13473,13429,13429,13416,13416,13378,13378,13357,13357,13330,13330,13325,13325,13309,13309,13295,13295,13273,13273,13268,13268,13260,13260,13257,13257,13241,13241,13235,13235,13223,13223,13187,13187,13184,13184,13171,13171,13160,13160,13132,13132,13131,13131,13117,13117,13109,13109,13089,13089,13066,13066,13065,13065,13060,13060,13054,13054,13046,13046,13022,13022,13008,13008,12989,12989,12988,12988,12980,12980,12975,12975,12974,12974,12970,12970,12941,12941,12867,12867,12855,12855,12851,12851,12820,12820,12716,12716,12682,12682,12681,12681,12676,12676,12667,12667,12608,12608,12583,12583,12498,12498,12465,12465,12458,12458,12445,12445,12434,12434,12433,12433,12405,12405,12395,12395,12387,12387,12365,12365,12334,12334,12328,12328,12326,12326,12312,12312,12309,12309,12299,12299,12288,12288,12242,12242,12217,12217,12188,12188,12165,12165,12155,12155,12142,12142,12065,12065,12049,12049,12008,12008,12006,12006,12003,12003,11995,11995,11991,11991,11981,11981,11979,11979,11960,11960,11935,11935,11926,11926,11923,11923,11918,11918,11908,11908,11888,11888,11857,11857,11815,11815,11808,11808,11802,11802,11792,11792,11769,11769,11763,11763,11745,11745,11729,11729,11702,11702,11680,11680,11672,11672,11664,11664,11656,11656,11637,11637,11608,11608,11598,11598,11583,11583,11575,11575,11549,11549,11527,11527,11519,11519,11502,11502,11495,11495,11494,11494,11477,11477,11455,11455,11443,11443,11441,11441,11423,11423,11409,11409,11406,11406,11377,11377,11371,11371,11350,11350,11349,11349,11335,11335,11329,11329,11327,11327,11275,11275,11274,11274,11255,11255,11237,11237,11225,11225,11224,11224,11223,11223,11213,11213,11212,11212,11193,11193,11186,11186,11182,11182,11158,11158,11140,11140,11102,11102,11101,11101,11099,11099,11098,11098,11086,11086,11066,11066,11057,11057,11053,11053,11039,11039,11023,11023,11000,11000,10987,10987,10980,10980,10979,10979,10977,10977,10926,10926,10918,10918,10910,10910,10902,10902,10874,10874,10873,10873,10872,10872,10839,10839,10828,10828,10820,10820,10811,10811,10804,10804,10777,10777,10768,10768,10748,10748,10740,10740,10732,10732,10695,10695,10691,10691,10667,10667,10640,10640,10624,10624,10605,10605,10570,10570,10568,10568,10563,10563,10552,10552,10539,10539,10538,10538,10537,10537,10506,10506,10505,10505,10504,10504,10500,10500,10498,10498,10496,10496,10487,10487,10475,10475,10438,10438,10437,10437,10436,10436,10400,10400,10392,10392,10383,10383,10379,10379,10372,10372,10355,10355,10331,10331,10328,10328,10320,10320,10311,10311,10310,10310,10309,10309,10284,10284,10274,10274,10272,10272,10257,10257,10251,10251,10249,10249,10224,10224,10210,10210,10204,10204,10202,10202,10197,10197,10184,10184,10178,10178,10173,10173,10145,10145,10141,10141,10118,10118,10111,10111,10110,10110,10106,10106,10104,10104,10103,10103,10092,10092,10088,10088,10087,10087,10077,10077,10063,10063,10053,10053,10032,10032,10022,10022,10019,10019,10011,10011,9995,9995,9981,9981,9979,9979,9973,9973,9967,9967,9965,9965,9954,9954,9947,9947,9946,9946,9932,9932,9918,9918,9913,9913,9905,9905,9894,9894,9860,9860,9853,9853,9849,9849,9830,9830,9827,9827,9815,9815,9814,9814,9804,9804,9801,9801,9796,9796,9793,9793,9751,9751,9743,9743,9737,9737,9731,9731,9717,9717,9715,9715,9686,9686,9685,9685,9680,9680,9665,9665,9661,9661,9648,9648,9605,9605,9597,9597,9593,9593,9587,9587,9568,9568,9559,9559,9556,9556,9551,9551,9548,9548,9538,9538,9527,9527,9525,9525,9516,9516,9513,9513,9504,9504,9497,9497,9483,9483,9462,9462,9455,9455,9452,9452,9438,9438,9435,9435,9426,9426,9418,9418,9415,9415,9410,9410,9408,9408,9396,9396,9393,9393,9388,9388,9384,9384,9380,9380,9369,9369,9366,9366,9365,9365,9355,9355,9347,9347,9338,9338,9335,9335,9331,9331,9319,9319,9313,9313,9299,9299,9263,9263,9255,9255,9241,9241,9239,9239,9219,9219,9215,9215,9209,9209,9207,9207,9200,9200,9197,9197,9186,9186,9182,9182,9178,9178,9174,9174,9166,9166,9162,9162,9160,9160,9158,9158,9156,9156,9141,9141,9140,9140,9137,9137,9119,9119,9105,9105,9079,9079,9061,9061,9051,9051,9020,9020,9014,9014,9011,9011,9005,9005,8998,8998,8992,8992,8991,8991,8984,8984,8970,8970,8960,8960,8949,8949,8925,8925,8919,8919,8917,8917,8908,8908,8883,8883,8874,8874,8869,8869,8846,8846,8842,8842,8838,8838,8831,8831,8826,8826,8825,8825,8801,8801,8793,8793,8775,8775,8769,8769,8767,8767,8760,8760,8758,8758,8755,8755,8749,8749,8727,8727,8722,8722,8717,8717,8696,8696,8687,8687,8684,8684,8683,8683,8673,8673,8628,8628,8610,8610,8589,8589,8577,8577,8571,8571,8542,8542,8535,8535,8532,8532,8519,8519,8506,8506,8493,8493,8487,8487,8486,8486,8458,8458,8456,8456,8431,8431,8428,8428,8414,8414,8402,8402,8401,8401,8391,8391,8388,8388,8377,8377,8369,8369,8367,8367,8339,8339,8338,8338,8313,8313,8310,8310,8293,8293,8286,8286,8277,8277,8272,8272,8270,8270,8269,8269,8264,8264,8256,8256,8245,8245,8244,8244,8242,8242,8213,8213,8208,8208,8203,8203,8202,8202,8195,8195,8188,8188,8185,8185,8179,8179,8147,8147,8133,8133,8122,8122,8121,8121,8104,8104,8095,8095,8086,8086,8076,8076,8039,8039,8031,8031,8023,8023,8020,8020,8007,8007,8006,8006,8004,8004,8003,8003,8000,8000,7998,7998,7975,7975,7973,7973,7945,7945,7942,7942,7935,7935,7916,7916,7904,7904,7893,7893,7881,7881,7862,7862,7858,7858,7855,7855,7850,7850,7835,7835,7832,7832,7829,7829,7826,7826,7825,7825,7813,7813,7796,7796,7783,7783,7782,7782,7776,7776,7763,7763,7762,7762,7760,7760,7750,7750,7747,7747,7733,7733,7730,7730,7728,7728,7720,7720,7705,7705,7700,7700,7698,7698,7685,7685,7666,7666,7665,7665,7664,7664,7650,7650,7637,7637,7632,7632,7624,7624,7621,7621,7618,7618,7615,7615,7613,7613,7607,7607,7606,7606,7598,7598,7595,7595,7588,7588,7580,7580,7573,7573,7558,7558,7556,7556,7553,7553,7550,7550,7537,7537,7524,7524,7503,7503,7490,7490,7479,7479,7475,7475,7472,7472,7471,7471,7466,7466,7448,7448,7440,7440,7432,7432,7430,7430,7425,7425,7420,7420,7418,7418,7409,7409,7408,7408,7387,7387,7384,7384,7379,7379,7378,7378,7375,7375,7374,7374,7368,7368,7356,7356,7355,7355,7354,7354,7347,7347,7333,7333,7320,7320,7318,7318,7299,7299,7297,7297,7296,7296,7290,7290,7284,7284,7277,7277,7273,7273,7270,7270,7267,7267,7256,7256,7249,7249,7237,7237,7227,7227,7214,7214,7213,7213,7196,7196,7195,7195,7185,7185,7181,7181,7177,7177,7174,7174,7156,7156,7143,7143,7137,7137,7135,7135,7129,7129,7127,7127,7121,7121,7111,7111,7107,7107,7099,7099,7089,7089,7087,7087,7085,7085,7081,7081,7076,7076,7074,7074,7073,7073,7072,7072,7070,7070,7068,7068,7062,7062,7056,7056,7051,7051,7040,7040,7032,7032,7031,7031,7028,7028,7016,7016,7010,7010,7009,7009,6992,6992,6990,6990,6984,6984,6978,6978,6972,6972,6966,6966,6963,6963,6962,6962,6958,6958,6957,6957,6952,6952,6948,6948,6944,6944,6938,6938,6936,6936,6928,6928,6926,6926,6923,6923,6912,6912,6904,6904,6890,6890,6889,6889,6882,6882,6879,6879,6875,6875,6868,6868,6867,6867,6863,6863,6860,6860,6842,6842,6841,6841,6831,6831,6823,6823,6815,6815,6799,6799,6797,6797,6794,6794,6791,6791,6776,6776,6764,6764,6758,6758,6756,6756,6753,6753,6749,6749,6741,6741,6734,6734,6729,6729,6726,6726,6721,6721,6707,6707,6690,6690,6686,6686,6675,6675,6674,6674,6670,6670,6668,6668,6647,6647,6645,6645,6642,6642,6639,6639,6631,6631,6628,6628,6627,6627,6624,6624,6621,6621,6618,6618,6615,6615,6610,6610,6607,6607,6600,6600,6598,6598,6593,6593,6592,6592,6590,6590,6587,6587,6583,6583,6572,6572,6569,6569,6567,6567,6562,6562,6560,6560,6558,6558,6557,6557,6554,6554,6553,6553,6548,6548,6545,6545,6543,6543,6527,6527,6522,6522,6520,6520,6516,6516,6510,6510,6503,6503,6500,6500,6497,6497,6494,6494,6471,6471,6461,6461,6458,6458,6450,6450,6447,6447,6440,6440,6439,6439,6438,6438,6434,6434,6417,6417,6416,6416,6407,6407,6400,6400,6396,6396,6393,6393,6390,6390,6364,6364,6355,6355,6353,6353,6349,6349,6315,6315,6310,6310,6309,6309,6306,6306,6305,6305,6295,6295,6286,6286,6281,6281,6280,6280,6271,6271,6268,6268,6257,6257,6256,6256,6240,6240,6235,6235,6227,6227,6223,6223,6222,6222,6221,6221,6220,6220,6206,6206,6189,6189,6188,6188,6187,6187,6180,6180,6177,6177,6174,6174,6172,6172,6168,6168,6166,6166,6158,6158,6142,6142,6139,6139,6129,6129,6121,6121,6115,6115,6107,6107,6103,6103,6089,6089,6080,6080,6079,6079,6068,6068,6067,6067,6064,6064,6063,6063,6061,6061,6059,6059,6056,6056,6055,6055,6053,6053,6052,6052,6049,6049,6048,6048,6044,6044,6034,6034,6026,6026,6020,6020,6017,6017,6016,6016,6011,6011,6003,6003,6000,6000,5989,5989,5986,5986,5983,5983,5981,5981,5971,5971,5963,5963,5957,5957,5942,5942,5928,5928,5925,5925,5924,5924,5916,5916,5915,5915,5908,5908,5903,5903,5900,5900,5896,5896,5887,5887,5883,5883,5882,5882,5877,5877,5876,5876,5875,5875,5865,5865,5858,5858,5854,5854,5853,5853,5851,5851,5837,5837,5836,5836,5831,5831,5828,5828,5826,5826,5821,5821,5813,5813,5808,5808,5803,5803,5802,5802,5801,5801,5797,5797,5782,5782,5767,5767,5764,5764,5761,5761,5750,5750,5737,5737,5735,5735,5730,5730,5729,5729,5721,5721,5708,5708,5701,5701,5699,5699,5674,5674,5673,5673,5671,5671,5662,5662,5661,5661,5659,5659,5658,5658,5653,5653,5651,5651,5650,5650,5644,5644,5639,5639,5635,5635,5629,5629,5627,5627,5619,5619,5617,5617,5615,5615,5610,5610,5606,5606,5596,5596,5586,5586,5573,5573,5569,5569,5558,5558,5553,5553,5552,5552,5547,5547,5543,5543,5541,5541,5540,5540,5539,5539,5535,5535,5534,5534,5533,5533,5521,5521,5517,5517,5500,5500,5492,5492,5465,5465,5460,5460,5454,5454,5445,5445,5436,5436,5435,5435,5434,5434,5430,5430,5428,5428,5424,5424,5414,5414,5406,5406,5393,5393,5390,5390,5372,5372,5368,5368,5366,5366,5363,5363,5358,5358,5357,5357,5355,5355,5354,5354,5353,5353,5352,5352,5351,5351,5348,5348,5341,5341,5330,5330,5325,5325,5318,5318,5312,5312,5305,5305,5297,5297,5291,5291,5288,5288,5287,5287,5284,5284,5283,5283,5281,5281,5280,5280,5275,5275,5274,5274,5265,5265,5261,5261,5247,5247,5244,5244,5243,5243,5241,5241,5233,5233,5226,5226,5225,5225,5207,5207,5191,5191,5188,5188,5182,5182,5178,5178,5174,5174,5169,5169,5160,5160,5158,5158,5153,5153,5149,5149,5140,5140,5136,5136,5132,5132,5130,5130,5127,5127,5116,5116,5115,5115,5114,5114,5108,5108,5106,5106,5093,5093,5086,5086,5085,5085,5080,5080,5069,5069,5068,5068,5057,5057,5056,5056,5054,5054,5046,5046,5032,5032,5027,5027,5018,5018,5015,5015,5012,5012,5011,5011,5009,5009,5000,5000,4993,4993,4989,4989,4985,4985,4984,4984,4974,4974,4973,4973,4970,4970,4967,4967,4964,4964,4959,4959,4949,4949,4946,4946,4945,4945,4933,4933,4920,4920,4913,4913,4911,4911,4910,4910,4909,4909,4907,4907,4906,4906,4903,4903,4901,4901,4883,4883,4880,4880,4874,4874,4865,4865,4861,4861,4855,4855,4853,4853,4845,4845,4834,4834,4832,4832,4810,4810,4808,4808,4807,4807,4805,4805,4798,4798,4797,4797,4785,4785,4783,4783,4778,4778,4777,4777,4774,4774,4770,4770,4769,4769,4765,4765,4760,4760,4751,4751,4749,4749,4739,4739,4723,4723,4713,4713,4707,4707,4706,4706,4700,4700,4694,4694,4691,4691,4686,4686,4683,4683,4681,4681,4679,4679,4678,4678,4674,4674,4667,4667,4666,4666,4665,4665,4664,4664,4661,4661,4648,4648,4639,4639,4627,4627,4624,4624,4601,4601,4591,4591,4588,4588,4584,4584,4580,4580,4566,4566,4560,4560,4547,4547,4545,4545,4533,4533,4532,4532,4526,4526,4513,4513,4506,4506,4495,4495,4492,4492,4489,4489,4486,4486,4482,4482,4480,4480,4477,4477,4472,4472,4471,4471,4460,4460,4459,4459,4455,4455,4431,4431,4421,4421,4418,4418,4404,4404,4403,4403,4402,4402,4401,4401,4399,4399,4393,4393,4390,4390,4388,4388,4374,4374,4370,4370,4363,4363,4353,4353,4344,4344,4337,4337,4334,4334,4330,4330,4327,4327,4321,4321,4318,4318,4314,4314,4313,4313,4310,4310,4309,4309,4307,4307,4301,4301,4299,4299,4297,4297,4285,4285,4275,4275,4258,4258,4238,4238,4236,4236,4224,4224,4213,4213,4211,4211,4208,4208,4206,4206,4205,4205,4204,4204,4201,4201,4199,4199,4194,4194,4192,4192,4190,4190,4186,4186,4185,4185,4184,4184,4183,4183,4173,4173,4170,4170,4167,4167,4165,4165,4153,4153,4144,4144,4142,4142,4134,4134,4130,4130,4129,4129,4119,4119,4113,4113,4110,4110,4105,4105,4095,4095,4083,4083,4082,4082,4081,4081,4075,4075,4062,4062,4061,4061,4059,4059,4058,4058,4055,4055,4050,4050,4043,4043,4041,4041,4034,4034,4033,4033,4032,4032,4026,4026,4018,4018,4001,4001,3999,3999,3997,3997,3995,3995,3993,3993,3989,3989,3988,3988,3986,3986,3984,3984,3981,3981,3979,3979,3971,3971,3969,3969,3966,3966,3962,3962,3961,3961,3951,3951,3945,3945,3942,3942,3936,3936,3933,3933,3932,3932,3931,3931,3924,3924,3923,3923,3922,3922,3920,3920,3918,3918,3911,3911,3901,3901,3897,3897,3893,3893,3892,3892,3889,3889,3885,3885,3876,3876,3870,3870,3864,3864,3861,3861,3858,3858,3853,3853,3830,3830,3824,3824,3813,3813,3807,3807,3799,3799,3795,3795,3793,3793,3791,3791,3790,3790,3789,3789,3787,3787,3784,3784,3781,3781,3771,3771,3759,3759,3752,3752,3751,3751,3747,3747,3746,3746,3744,3744,3743,3743,3735,3735,3732,3732,3724,3724,3712,3712,3711,3711,3698,3698,3690,3690,3689,3689,3668,3668,3664,3664,3663,3663,3660,3660,3654,3654,3651,3651,3648,3648,3647,3647,3645,3645,3642,3642,3640,3640,3619,3619,3611,3611,3610,3610,3604,3604,3602,3602,3596,3596,3591,3591,3588,3588,3582,3582,3568,3568,3566,3566,3561,3561,3552,3552,3549,3549,3546,3546,3541,3541,3530,3530,3525,3525,3523,3523,3519,3519,3514,3514,3512,3512,3503,3503,3496,3496,3488,3488,3487,3487,3486,3486,3466,3466,3464,3464,3462,3462,3458,3458,3457,3457,3447,3447,3444,3444,3443,3443,3440,3440,3436,3436,3421,3421,3419,3419,3399,3399,3388,3388,3383,3383,3369,3369,3368,3368,3367,3367,3366,3366,3365,3365,3361,3361,3356,3356,3351,3351,3350,3350,3349,3349,3348,3348,3343,3343,3341,3341,3339,3339,3337,3337,3325,3325,3324,3324,3322,3322,3321,3321,3307,3307,3303,3303,3282,3282,3281,3281,3280,3280,3279,3279,3274,3274,3273,3273,3269,3269,3266,3266,3262,3262,3260,3260,3257,3257,3256,3256,3254,3254,3250,3250,3245,3245,3244,3244,3240,3240,3231,3231,3230,3230,3229,3229,3224,3224,3223,3223,3221,3221,3217,3217,3216,3216,3214,3214,3203,3203,3202,3202,3201,3201,3195,3195,3194,3194,3186,3186,3185,3185,3180,3180,3178,3178,3174,3174,3173,3173,3169,3169,3167,3167,3158,3158,3157,3157,3155,3155,3146,3146,3144,3144,3143,3143,3121,3121,3119,3119,3117,3117,3112,3112,3104,3104,3100,3100,3096,3096,3094,3094,3088,3088,3087,3087,3086,3086,3083,3083,3082,3082,3081,3081,3069,3069,3068,3068,3067,3067,3065,3065,3050,3050,3049,3049,3047,3047,3045,3045,3043,3043,3033,3033,3028,3028,3025,3025,3017,3017,3013,3013,3000,3000,2991,2991,2989,2989,2981,2981,2977,2977,2976,2976,2971,2971,2969,2969,2964,2964,2962,2962,2956,2956,2954,2954,2949,2949,2948,2948,2946,2946,2944,2944,2942,2942,2941,2941,2940,2940,2939,2939,2936,2936,2935,2935,2921,2921,2920,2920,2913,2913,2912,2912,2909,2909,2905,2905,2901,2901,2900,2900,2888,2888,2886,2886,2884,2884,2883,2883,2879,2879,2876,2876,2868,2868,2867,2867,2864,2864,2856,2856,2850,2850,2845,2845,2837,2837,2830,2830,2829,2829,2828,2828,2825,2825,2815,2815,2813,2813,2802,2802,2799,2799,2790,2790,2787,2787,2782,2782,2779,2779,2777,2777,2772,2772,2770,2770,2767,2767,2760,2760,2756,2756,2753,2753,2749,2749,2748,2748,2744,2744,2743,2743,2740,2740,2738,2738,2737,2737,2728,2728,2726,2726,2725,2725,2721,2721,2719,2719,2715,2715,2710,2710,2703,2703,2696,2696,2690,2690,2689,2689,2688,2688,2685,2685,2684,2684,2679,2679,2675,2675,2673,2673,2670,2670,2668,2668,2665,2665,2663,2663,2657,2657,2655,2655,2651,2651,2649,2649,2638,2638,2637,2637,2634,2634,2629,2629,2626,2626,2624,2624,2620,2620,2615,2615,2613,2613,2611,2611,2609,2609,2606,2606,2604,2604,2593,2593,2582,2582,2581,2581,2580,2580,2579,2579,2577,2577,2561,2561,2560,2560,2552,2552,2542,2542,2537,2537,2530,2530,2522,2522,2521,2521,2520,2520,2515,2515,2512,2512,2506,2506,2500,2500,2499,2499,2493,2493,2491,2491,2489,2489,2487,2487,2486,2486,2485,2485,2483,2483,2481,2481,2480,2480,2476,2476,2474,2474,2473,2473,2472,2472,2468,2468,2465,2465,2461,2461,2460,2460,2452,2452,2447,2447,2446,2446,2444,2444,2439,2439,2435,2435,2431,2431,2430,2430,2429,2429,2428,2428,2423,2423,2416,2416,2411,2411,2410,2410,2409,2409,2407,2407,2404,2404,2403,2403,2400,2400,2399,2399,2398,2398,2392,2392,2391,2391,2387,2387,2384,2384,2383,2383,2382,2382,2381,2381,2378,2378,2371,2371,2367,2367,2366,2366,2365,2365,2364,2364,2363,2363,2358,2358,2347,2347,2341,2341,2338,2338,2333,2333,2329,2329,2326,2326,2325,2325,2321,2321,2318,2318,2314,2314,2305,2305,2300,2300,2294,2294,2292,2292,2291,2291,2284,2284,2281,2281,2278,2278,2273,2273,2271,2271,2266,2266,2262,2262,2253,2253,2250,2250,2249,2249,2248,2248,2242,2242,2239,2239,2234,2234,2232,2232,2230,2230,2223,2223,2219,2219,2217,2217,2216,2216,2214,2214,2211,2211,2208,2208,2207,2207,2202,2202,2198,2198,2191,2191,2185,2185,2183,2183,2179,2179,2178,2178,2176,2176,2172,2172,2169,2169,2168,2168,2167,2167,2161,2161,2156,2156,2153,2153,2151,2151,2150,2150,2147,2147,2143,2143,2142,2142,2141,2141,2136,2136,2129,2129,2122,2122,2121,2121,2114,2114,2100,2100,2099,2099,2097,2097,2093,2093,2089,2089,2087,2087,2086,2086,2085,2085,2083,2083,2079,2079,2074,2074,2072,2072,2070,2070,2068,2068,2065,2065,2059,2059,2057,2057,2055,2055,2050,2050,2044,2044,2042,2042,2041,2041,2039,2039,2037,2037,2036,2036,2034,2034,2031,2031,2028,2028,2027,2027,2026,2026,2021,2021,2020,2020,2012,2012,2011,2011,2009,2009,2008,2008,2005,2005,2004,2004,1999,1999,1995,1995,1994,1994,1992,1992,1982,1982,1978,1978,1977,1977,1976,1976,1975,1975,1973,1973,1972,1972,1967,1967,1964,1964,1954,1954,1949,1949,1947,1947,1946,1946,1944,1944,1940,1940,1939,1939,1938,1938,1937,1937,1936,1936,1934,1934,1931,1931,1929,1929,1925,1925,1922,1922,1918,1918,1913,1913,1910,1910,1907,1907,1903,1903,1901,1901,1897,1897,1893,1893,1891,1891,1887,1887,1885,1885,1884,1884,1883,1883,1882,1882,1881,1881,1880,1880,1877,1877,1876,1876,1874,1874,1872,1872,1867,1867,1862,1862,1861,1861,1860,1860,1858,1858,1856,1856,1851,1851,1849,1849,1848,1848,1847,1847,1846,1846,1845,1845,1844,1844,1843,1843,1842,1842,1840,1840,1838,1838,1837,1837,1836,1836,1834,1834,1830,1830,1828,1828,1816,1816,1814,1814,1810,1810,1809,1809,1807,1807,1806,1806,1804,1804,1802,1802,1796,1796,1795,1795,1794,1794,1793,1793,1791,1791,1790,1790,1789,1789,1788,1788,1786,1786,1785,1785,1780,1780,1777,1777,1775,1775,1774,1774,1773,1773,1769,1769,1767,1767,1766,1766,1763,1763,1762,1762,1750,1750,1749,1749,1747,1747,1746,1746,1744,1744,1743,1743,1741,1741,1740,1740,1739,1739,1738,1738,1737,1737,1733,1733,1731,1731,1730,1730,1729,1729,1728,1728,1727,1727,1726,1726,1725,1725,1724,1724,1723,1723,1722,1722,1717,1717,1716,1716,1711,1711,1706,1706,1703,1703,1698,1698,1697,1697,1693,1693,1691,1691,1690,1690,1689,1689,1688,1688,1686,1686,1683,1683,1680,1680,1676,1676,1675,1675,1671,1671,1670,1670,1668,1668,1666,1666,1665,1665,1663,1663,1662,1662,1661,1661,1660,1660,1659,1659,1658,1658,1657,1657,1656,1656,1653,1653,1652,1652,1651,1651,1649,1649,1648,1648,1647,1647,1646,1646,1645,1645,1644,1644,1643,1643,1637,1637,1636,1636,1633,1633,1629,1629,1628,1628,1626,1626,1625,1625,1624,1624,1623,1623,1622,1622,1621,1621,1615,1615,1613,1613,1609,1609,1605,1605,1600,1600,1599,1599,1598,1598,1595,1595,1593,1593,1583,1583,1581,1581,1580,1580,1579,1579,1578,1578,1577,1577,1575,1575,1574,1574,1572,1572,1570,1570,1569,1569,1567,1567,1561,1561,1558,1558,1556,1556,1553,1553,1551,1551,1549,1549,1547,1547,1545,1545,1544,1544,1542,1542,1540,1540,1539,1539,1537,1537,1536,1536,1535,1535,1533,1533,1531,1531,1530,1530,1528,1528,1526,1526,1525,1525,1519,1519,1513,1513,1512,1512,1511,1511,1509,1509,1508,1508,1504,1504,1502,1502,1501,1501,1495,1495,1494,1494,1493,1493,1492,1492,1491,1491,1490,1490,1488,1488,1486,1486,1484,1484,1483,1483,1482,1482,1479,1479,1477,1477,1474,1474,1472,1472,1470,1470,1469,1469,1468,1468,1467,1467,1465,1465,1460,1460,1457,1457,1456,1456,1455,1455,1454,1454,1451,1451,1449,1449,1447,1447,1446,1446,1444,1444,1443,1443,1442,1442,1441,1441,1438,1438,1437,1437,1436,1436,1434,1434,1431,1431,1428,1428,1427,1427,1425,1425,1424,1424,1418,1418,1417,1417,1415,1415,1414,1414,1412,1412,1410,1410,1409,1409,1408,1408,1406,1406,1402,1402,1399,1399,1398,1398,1397,1397,1396,1396,1393,1393,1391,1391,1388,1388,1387,1387,1386,1386,1384,1384,1383,1383,1382,1382,1381,1381,1378,1378,1374,1374,1373,1373,1371,1371,1370,1370,1369,1369,1368,1368,1367,1367,1366,1366,1365,1365,1364,1364,1362,1362,1361,1361,1358,1358,1357,1357,1354,1354,1350,1350,1349,1349,1348,1348,1345,1345,1342,1342,1341,1341,1340,1340,1339,1339,1335,1335,1331,1331,1330,1330,1329,1329,1328,1328,1327,1327,1324,1324,1323,1323,1322,1322,1321,1321,1320,1320,1318,1318,1314,1314,1313,1313,1309,1309,1307,1307,1305,1305,1304,1304,1299,1299,1297,1297,1296,1296,1295,1295,1294,1294,1293,1293,1292,1292,1291,1291,1290,1290,1289,1289,1287,1287,1285,1285,1283,1283,1282,1282,1280,1280,1277,1277,1275,1275,1274,1274,1272,1272,1267,1267,1266,1266,1264,1264,1261,1261,1259,1259,1258,1258,1257,1257,1256,1256,1254,1254,1253,1253,1251,1251,1249,1249,1248,1248,1247,1247,1246,1246,1245,1245,1244,1244,1242,1242,1241,1241,1238,1238,1237,1237,1235,1235,1234,1234,1233,1233,1232,1232,1231,1231,1230,1230,1226,1226,1224,1224,1222,1222,1219,1219,1218,1218,1217,1217,1216,1216,1215,1215,1214,1214,1211,1211,1208,1208,1206,1206,1205,1205,1204,1204,1203,1203,1199,1199,1198,1198,1196,1196,1193,1193,1192,1192,1191,1191,1190,1190,1189,1189,1188,1188,1187,1187,1186,1186,1184,1184,1183,1183,1182,1182,1181,1181,1180,1180,1179,1179,1177,1177,1175,1175,1174,1174,1172,1172,1170,1170,1169,1169,1168,1168,1167,1167,1166,1166,1164,1164,1161,1161,1160,1160,1159,1159,1158,1158,1157,1157,1156,1156,1154,1154,1153,1153,1152,1152,1150,1150,1149,1149,1148,1148,1147,1147,1145,1145,1144,1144,1143,1143,1142,1142,1136,1136,1134,1134,1132,1132,1131,1131,1130,1130,1129,1129,1128,1128,1127,1127,1126,1126,1125,1125,1124,1124,1122,1122,1121,1121,1119,1119,1118,1118,1111,1111,1110,1110,1109,1109,1108,1108,1106,1106,1105,1105,1104,1104,1103,1103,1101,1101,1099,1099,1098,1098,1095,1095,1094,1094,1093,1093,1092,1092,1091,1091,1089,1089,1088,1088,1087,1087,1086,1086,1084,1084,1083,1083,1082,1082,1081,1081,1080,1080,1079,1079,1078,1078,1075,1075,1074,1074,1073,1073,1071,1071,1070,1070,1069,1069,1067,1067,1066,1066,1064,1064,1063,1063,1062,1062,1061,1061,1060,1060,1059,1059,1058,1058,1057,1057,1056,1056,1055,1055,1054,1054,1053,1053,1052,1052,1051,1051,1050,1050,1049,1049,1048,1048,1047,1047,1046,1046,1045,1045,1044,1044,1042,1042,1041,1041,1040,1040,1036,1036,1035,1035,1034,1034,1033,1033,1032,1032,1031,1031,1030,1030,1029,1029,1026,1026,1024,1024,1022,1022,1021,1021,1020,1020,1019,1019,1018,1018,1017,1017,1016,1016,1015,1015,1014,1014,1013,1013,1011,1011,1010,1010,1009,1009,1007,1007,1006,1006,1005,1005,1004,1004,1003,1003,1002,1002,1001,1001,1000,1000,999,999,998,998,996,996,992,992,991,991,989,989,988,988,987,987,984,984,982,982,981,981,980,980,978,978,977,977,976,976,975,975,974,974,973,973,972,972,971,971,970,970,969,969,968,968,966,966,965,965,964,964,963,963,962,962,961,961,960,960,959,959,958,958,957,957,956,956,955,955,954,954,953,953,952,952,951,951,950,950,948,948,947,947,946,946,945,945,944,944,943,943,942,942,941,941,940,940,939,939,938,938,937,937,935,935,934,934,932,932,931,931,930,930,929,929,928,928,927,927,926,926,925,925,924,924,923,923,922,922,919,919,918,918,917,917,916,916,915,915,914,914,913,913,912,912,911,911,910,910,909,909,908,908,907,907,906,906,905,905,904,904,903,903,902,902,901,901,900,900,899,899,898,898,897,897,896,896,895,895,894,894,893,893,892,892,890,890,889,889,888,888,887,887,886,886,885,885,884,884,883,883,882,882,881,881,880,880,879,879,878,878,877,877,876,876,875,875,874,874,873,873,872,872,871,871,870,870,869,869,868,868,867,867,866,866,865,865,864,864,863,863,862,862,861,861,860,860,859,859,858,858,857,857,856,856,855,855,854,854,853,853,852,852,851,851,850,850,849,849,848,848,847,847,846,846,845,845,844,844,843,843,842,842,841,841,840,840,839,839,838,838,837,837,836,836,835,835,834,834,833,833,832,832,831,831,829,829,828,828,827,827,826,826,824,824,823,823,822,822,821,821,820,820,819,819,818,818,816,816,815,815,813,813,812,812,811,811,810,810,809,809,808,808,807,807,804,804,803,803,802,802,801,801,800,800,799,799,797,797,796,796,795,795,794,794,793,793,792,792,791,791,790,790,789,789,788,788,787,787,786,786,785,785,784,784,783,783,782,782,781,781,780,780,779,779,778,778,777,777,776,776,775,775,774,774,773,773,772,772,771,771,770,770,769,769,768,768,767,767,766,766,765,765,764,764,763,763,762,762,761,761,760,760,759,759,758,758,757,757,756,756,755,755,754,754,753,753,752,752,750,750,749,749,748,748,747,747,746,746,745,745,744,744,743,743,741,741,740,740,739,739,737,737,736,736,735,735,734,734,733,733,732,732,730,730,729,729,728,728,727,727,726,726,724,724,723,723,722,722,721,721,720,720,719,719,718,718,717,717,716,716,715,715,714,714,713,713,712,712,711,711,710,710,708,708,707,707,706,706,705,705,704,704,703,703,702,702,701,701,700,700,698,698,697,697,695,695,694,694,693,693,692,692,691,691,690,690,689,689,688,688,687,687,686,686,685,685,684,684,683,683,682,682,681,681,680,680,679,679,678,678,677,677,676,676,675,675,674,674,673,673,672,672,671,671,670,670,669,669,668,668,667,667,666,666,665,665,664,664,663,663,662,662,661,661,660,660,659,659,658,658,657,657,656,656,655,655,654,654,653,653,652,652,651,651,650,650,649,649,648,648,647,647,646,646,645,645,644,644,643,643,642,642,641,641,640,640,639,639,638,638,637,637,636,636,635,635,634,634,633,633,632,632,631,631,630,630,629,629,628,628,627,627,626,626,625,625,624,624,623,623,622,622,621,621,620,620,619,619,618,618,615,615,614,614,613,613,612,612,610,610,609,609,607,607,606,606,605,605,604,604,603,603,602,602,601,601,598,598,595,595,594,594,591,591,590,590,589,589,588,588,584,584,583,583,582,582,580,580,578,578,575,575,574,574,573,573,572,572,571,571,570,570,569,569,567,567,566,566,561,561,559,559,558,558,556,556,555,555,553,553,551,551,550,550,549,549,548,548,546,546,544,544,542,542,539,539,538,538,537,537,535,535,534,534,532,532,531,531,530,530,529,529,526,526,523,523,522,522,521,521,520,520,519,519,517,517,515,515,514,514,513,513,512,512,510,510,507,507,506,506,505,505,491,491,490,490,489,489,487,487,485,485,481,481,480,480,479,479,478,478,476,476,475,475,469,469,467,467,463,463,459,459,458,458,457,457,455,455,454,454,453,453,446,446,445,445,444,444,440,440,438,438,436,436,434,434,433,433,430,430,427,427,424,424,423,423,420,420,418,418,414,414,413,413,411,411,409,409,407,407,405,405,403,403,0.0],[279110,279110,279110,216386,216386,209534,209534,176309,176309,176264,176264,168662,168662,158836,158836,153922,153922,152912,152912,146402,146402,133949,133949,130297,130297,130117,130117,124229,124229,121442,121442,120371,120371,118878,118878,118800,118800,118545,118545,116707,116707,108492,108492,107134,107134,104532,104532,102212,102212,101870,101870,101516,101516,99467,99467,98979,98979,96616,96616,93428,93428,90675,90675,88974,88974,87285,87285,87189,87189,86921,86921,86401,86401,85957,85957,84370,84370,84141,84141,83948,83948,83038,83038,82863,82863,82519,82519,81695,81695,81670,81670,80691,80691,80440,80440,78735,78735,77566,77566,77262,77262,77041,77041,76650,76650,76612,76612,76410,76410,75682,75682,74289,74289,73777,73777,73699,73699,73677,73677,73523,73523,72639,72639,72443,72443,72081,72081,71972,71972,71433,71433,71072,71072,70907,70907,69838,69838,69650,69650,69552,69552,69499,69499,69424,69424,69134,69134,68715,68715,68565,68565,68327,68327,68269,68269,67670,67670,66407,66407,65392,65392,65212,65212,65094,65094,64143,64143,64103,64103,63958,63958,63819,63819,63345,63345,62867,62867,62809,62809,62488,62488,62017,62017,61931,61931,61898,61898,61681,61681,60420,60420,60041,60041,59774,59774,59729,59729,59712,59712,59536,59536,59477,59477,59471,59471,59235,59235,57910,57910,57554,57554,57093,57093,56666,56666,56408,56408,56359,56359,55546,55546,55353,55353,54412,54412,54348,54348,53976,53976,53781,53781,53701,53701,53044,53044,52762,52762,52751,52751,52665,52665,52646,52646,52214,52214,52004,52004,51955,51955,51820,51820,51668,51668,51384,51384,51104,51104,50845,50845,50527,50527,50322,50322,50169,50169,50115,50115,50004,50004,49554,49554,49432,49432,49176,49176,49102,49102,48582,48582,48324,48324,48247,48247,47707,47707,47608,47608,47401,47401,47376,47376,46875,46875,46849,46849,46545,46545,46516,46516,46064,46064,45868,45868,45810,45810,45743,45743,45590,45590,45507,45507,45285,45285,45145,45145,44958,44958,44523,44523,44200,44200,43406,43406,43224,43224,43124,43124,42880,42880,42878,42878,42750,42750,42729,42729,42666,42666,42662,42662,42452,42452,41997,41997,41845,41845,41773,41773,41588,41588,41582,41582,41522,41522,41447,41447,41355,41355,41297,41297,41082,41082,41028,41028,40856,40856,40483,40483,40163,40163,40101,40101,39572,39572,39482,39482,39434,39434,39251,39251,39217,39217,39201,39201,39070,39070,38954,38954,38938,38938,38927,38927,38851,38851,38737,38737,38701,38701,38582,38582,38521,38521,38447,38447,38177,38177,38041,38041,38006,38006,37915,37915,37849,37849,37634,37634,37548,37548,37451,37451,37375,37375,37366,37366,37276,37276,37177,37177,37176,37176,37175,37175,37072,37072,37044,37044,37041,37041,36957,36957,36942,36942,36823,36823,36560,36560,36552,36552,36307,36307,35805,35805,35399,35399,35208,35208,35197,35197,35157,35157,34987,34987,34624,34624,34561,34561,34496,34496,34430,34430,34420,34420,34340,34340,34222,34222,34184,34184,34157,34157,34007,34007,33998,33998,33950,33950,33868,33868,33856,33856,33403,33403,33395,33395,33377,33377,33373,33373,33357,33357,33353,33353,33338,33338,33252,33252,33143,33143,33048,33048,32946,32946,32918,32918,32865,32865,32835,32835,32821,32821,32492,32492,32425,32425,32385,32385,32366,32366,32318,32318,32079,32079,32064,32064,32051,32051,32034,32034,31895,31895,31781,31781,31737,31737,31633,31633,31561,31561,31519,31519,31499,31499,31409,31409,31063,31063,31004,31004,30979,30979,30872,30872,30743,30743,30570,30570,30564,30564,30541,30541,30527,30527,30505,30505,30310,30310,30299,30299,30183,30183,30065,30065,29780,29780,29714,29714,29686,29686,29650,29650,29573,29573,29545,29545,29539,29539,29529,29529,29455,29455,29405,29405,29387,29387,29262,29262,29222,29222,29163,29163,29073,29073,29070,29070,28888,28888,28887,28887,28849,28849,28838,28838,28814,28814,28756,28756,28580,28580,28559,28559,28517,28517,28436,28436,28310,28310,28234,28234,28191,28191,28187,28187,28100,28100,27996,27996,27982,27982,27947,27947,27794,27794,27706,27706,27611,27611,27429,27429,27406,27406,27377,27377,27297,27297,27234,27234,27219,27219,27172,27172,27170,27170,27124,27124,26963,26963,26874,26874,26864,26864,26819,26819,26685,26685,26682,26682,26648,26648,26538,26538,26363,26363,26228,26228,26221,26221,26163,26163,26131,26131,26071,26071,26025,26025,26011,26011,25934,25934,25880,25880,25810,25810,25806,25806,25772,25772,25770,25770,25750,25750,25708,25708,25678,25678,25537,25537,25513,25513,25506,25506,25494,25494,25482,25482,25237,25237,25224,25224,25207,25207,25187,25187,25123,25123,25108,25108,25049,25049,24908,24908,24901,24901,24799,24799,24743,24743,24740,24740,24684,24684,24680,24680,24678,24678,24659,24659,24547,24547,24475,24475,24231,24231,24188,24188,24051,24051,23921,23921,23866,23866,23851,23851,23848,23848,23829,23829,23703,23703,23694,23694,23630,23630,23627,23627,23611,23611,23560,23560,23532,23532,23501,23501,23463,23463,23452,23452,23339,23339,23293,23293,23277,23277,23254,23254,23243,23243,23135,23135,23067,23067,22896,22896,22893,22893,22844,22844,22775,22775,22724,22724,22677,22677,22656,22656,22549,22549,22514,22514,22501,22501,22460,22460,22448,22448,22444,22444,22366,22366,22344,22344,22254,22254,22224,22224,22211,22211,22100,22100,21913,21913,21715,21715,21703,21703,21687,21687,21679,21679,21613,21613,21569,21569,21454,21454,21452,21452,21448,21448,21430,21430,21427,21427,21406,21406,21391,21391,21356,21356,21293,21293,21249,21249,21214,21214,21112,21112,21067,21067,21060,21060,21022,21022,20990,20990,20977,20977,20845,20845,20820,20820,20729,20729,20710,20710,20681,20681,20676,20676,20591,20591,20579,20579,20577,20577,20538,20538,20531,20531,20490,20490,20468,20468,20461,20461,20426,20426,20372,20372,20339,20339,20334,20334,20229,20229,20228,20228,20167,20167,20136,20136,20112,20112,20042,20042,20033,20033,20017,20017,19974,19974,19947,19947,19941,19941,19913,19913,19890,19890,19817,19817,19814,19814,19812,19812,19811,19811,19738,19738,19719,19719,19682,19682,19678,19678,19600,19600,19577,19577,19536,19536,19415,19415,19290,19290,19287,19287,19132,19132,19032,19032,19011,19011,19006,19006,18989,18989,18985,18985,18973,18973,18950,18950,18922,18922,18900,18900,18898,18898,18851,18851,18813,18813,18772,18772,18768,18768,18732,18732,18675,18675,18608,18608,18589,18589,18569,18569,18565,18565,18559,18559,18545,18545,18503,18503,18459,18459,18412,18412,18393,18393,18369,18369,18360,18360,18351,18351,18318,18318,18309,18309,18307,18307,18298,18298,18225,18225,18223,18223,18209,18209,18176,18176,18172,18172,18171,18171,18109,18109,18108,18108,18106,18106,18060,18060,18049,18049,18034,18034,17986,17986,17976,17976,17941,17941,17932,17932,17885,17885,17868,17868,17848,17848,17765,17765,17716,17716,17647,17647,17616,17616,17590,17590,17528,17528,17520,17520,17512,17512,17439,17439,17400,17400,17398,17398,17379,17379,17359,17359,17328,17328,17302,17302,17280,17280,17272,17272,17269,17269,17245,17245,17223,17223,17186,17186,17061,17061,16984,16984,16927,16927,16813,16813,16799,16799,16787,16787,16780,16780,16767,16767,16765,16765,16724,16724,16665,16665,16593,16593,16569,16569,16464,16464,16443,16443,16439,16439,16366,16366,16325,16325,16287,16287,16223,16223,16149,16149,16096,16096,16063,16063,15955,15955,15948,15948,15937,15937,15885,15885,15860,15860,15834,15834,15798,15798,15757,15757,15713,15713,15702,15702,15691,15691,15663,15663,15628,15628,15619,15619,15599,15599,15529,15529,15517,15517,15491,15491,15412,15412,15404,15404,15382,15382,15365,15365,15356,15356,15342,15342,15331,15331,15279,15279,15266,15266,15259,15259,15242,15242,15233,15233,15201,15201,15181,15181,15144,15144,15110,15110,14998,14998,14944,14944,14943,14943,14937,14937,14834,14834,14734,14734,14710,14710,14671,14671,14664,14664,14612,14612,14578,14578,14489,14489,14409,14409,14356,14356,14322,14322,14309,14309,14256,14256,14241,14241,14132,14132,14102,14102,14087,14087,14077,14077,14076,14076,14070,14070,14048,14048,14047,14047,14036,14036,14015,14015,13969,13969,13891,13891,13873,13873,13847,13847,13791,13791,13699,13699,13690,13690,13669,13669,13665,13665,13664,13664,13645,13645,13556,13556,13500,13500,13493,13493,13486,13486,13473,13473,13416,13416,13325,13325,13309,13309,13273,13273,13268,13268,13241,13241,13235,13235,13223,13223,13187,13187,13171,13171,13160,13160,13132,13132,13131,13131,13117,13117,13066,13066,13065,13065,13060,13060,13054,13054,13046,13046,13022,13022,12996,12996,12989,12989,12988,12988,12975,12975,12974,12974,12970,12970,12941,12941,12867,12867,12855,12855,12820,12820,12716,12716,12682,12682,12608,12608,12583,12583,12498,12498,12458,12458,12445,12445,12433,12433,12405,12405,12395,12395,12365,12365,12334,12334,12328,12328,12326,12326,12312,12312,12309,12309,12299,12299,12288,12288,12217,12217,12210,12210,12188,12188,12165,12165,12155,12155,12049,12049,12006,12006,12003,12003,11995,11995,11991,11991,11981,11981,11979,11979,11960,11960,11926,11926,11918,11918,11808,11808,11802,11802,11769,11769,11763,11763,11745,11745,11702,11702,11680,11680,11672,11672,11664,11664,11656,11656,11637,11637,11608,11608,11583,11583,11527,11527,11502,11502,11477,11477,11455,11455,11448,11448,11406,11406,11371,11371,11350,11350,11327,11327,11275,11275,11255,11255,11237,11237,11225,11225,11223,11223,11213,11213,11186,11186,11101,11101,11086,11086,11053,11053,11023,11023,11000,11000,10987,10987,10980,10980,10979,10979,10977,10977,10926,10926,10918,10918,10910,10910,10902,10902,10874,10874,10820,10820,10811,10811,10804,10804,10777,10777,10768,10768,10740,10740,10739,10739,10732,10732,10695,10695,10691,10691,10667,10667,10640,10640,10552,10552,10539,10539,10538,10538,10537,10537,10504,10504,10501,10501,10487,10487,10475,10475,10436,10436,10400,10400,10392,10392,10379,10379,10372,10372,10355,10355,10331,10331,10328,10328,10309,10309,10272,10272,10257,10257,10251,10251,10249,10249,10197,10197,10178,10178,10173,10173,10145,10145,10141,10141,10106,10106,10103,10103,10088,10088,10077,10077,10063,10063,10053,10053,10032,10032,10019,10019,10011,10011,9995,9995,9981,9981,9973,9973,9965,9965,9954,9954,9947,9947,9905,9905,9894,9894,9860,9860,9853,9853,9830,9830,9815,9815,9814,9814,9804,9804,9801,9801,9796,9796,9793,9793,9751,9751,9715,9715,9714,9714,9711,9711,9680,9680,9665,9665,9661,9661,9646,9646,9605,9605,9597,9597,9559,9559,9556,9556,9551,9551,9525,9525,9516,9516,9504,9504,9462,9462,9455,9455,9452,9452,9438,9438,9435,9435,9426,9426,9418,9418,9415,9415,9410,9410,9408,9408,9396,9396,9393,9393,9384,9384,9380,9380,9369,9369,9366,9366,9338,9338,9335,9335,9331,9331,9319,9319,9313,9313,9299,9299,9263,9263,9255,9255,9239,9239,9219,9219,9215,9215,9213,9213,9209,9209,9207,9207,9200,9200,9178,9178,9174,9174,9166,9166,9162,9162,9160,9160,9158,9158,9156,9156,9141,9141,9140,9140,9105,9105,9079,9079,9061,9061,9020,9020,9011,9011,8992,8992,8991,8991,8984,8984,8925,8925,8919,8919,8917,8917,8900,8900,8883,8883,8874,8874,8869,8869,8846,8846,8842,8842,8826,8826,8801,8801,8767,8767,8755,8755,8722,8722,8687,8687,8684,8684,8589,8589,8571,8571,8493,8493,8475,8475,8456,8456,8431,8431,8428,8428,8414,8414,8401,8401,8391,8391,8388,8388,8369,8369,8338,8338,8323,8323,8313,8313,8293,8293,8272,8272,8270,8270,8269,8269,8264,8264,8256,8256,8245,8245,8243,8243,8242,8242,8213,8213,8203,8203,8188,8188,8179,8179,8147,8147,8133,8133,8122,8122,8121,8121,8116,8116,8095,8095,8086,8086,8076,8076,8039,8039,8020,8020,8012,8012,8007,8007,8000,8000,7975,7975,7942,7942,7916,7916,7893,7893,7881,7881,7855,7855,7851,7851,7832,7832,7825,7825,7813,7813,7796,7796,7776,7776,7763,7763,7750,7750,7747,7747,7733,7733,7730,7730,7720,7720,7700,7700,7698,7698,7685,7685,7666,7666,7664,7664,7650,7650,7642,7642,7621,7621,7618,7618,7613,7613,7588,7588,7558,7558,7556,7556,7553,7553,7537,7537,7475,7475,7448,7448,7430,7430,7409,7409,7374,7374,7356,7356,7333,7333,7318,7318,7309,7309,7296,7296,7273,7273,7267,7267,7227,7227,7214,7214,7195,7195,7177,7177,7156,7156,7139,7139,7135,7135,7129,7129,7111,7111,7107,7107,7099,7099,7087,7087,7074,7074,7072,7072,7070,7070,7062,7062,7056,7056,7051,7051,7036,7036,7032,7032,7016,7016,7010,7010,6990,6990,6978,6978,6972,6972,6963,6963,6962,6962,6958,6958,6957,6957,6944,6944,6926,6926,6868,6868,6867,6867,6860,6860,6842,6842,6831,6831,6823,6823,6797,6797,6791,6791,6776,6776,6764,6764,6758,6758,6754,6754,6753,6753,6749,6749,6729,6729,6726,6726,6707,6707,6697,6697,6686,6686,6675,6675,6642,6642,6631,6631,6628,6628,6627,6627,6621,6621,6618,6618,6615,6615,6610,6610,6607,6607,6601,6601,6593,6593,6562,6562,6560,6560,6554,6554,6553,6553,6543,6543,6527,6527,6497,6497,6461,6461,6434,6434,6417,6417,6416,6416,6407,6407,6396,6396,6393,6393,6390,6390,6322,6322,6309,6309,6280,6280,6257,6257,6221,6221,6206,6206,6189,6189,6187,6187,6180,6180,6168,6168,6166,6166,6139,6139,6068,6068,6061,6061,6049,6049,6048,6048,6036,6036,6026,6026,6017,6017,5998,5998,5957,5957,5934,5934,5925,5925,5908,5908,5887,5887,5877,5877,5851,5851,5836,5836,5831,5831,5824,5824,5808,5808,5802,5802,5801,5801,5764,5764,5761,5761,5740,5740,5730,5730,5708,5708,5701,5701,5699,5699,5671,5671,5662,5662,5661,5661,5660,5660,5658,5658,5639,5639,5635,5635,5627,5627,5619,5619,5543,5543,5541,5541,5540,5540,5539,5539,5533,5533,5508,5508,5500,5500,5445,5445,5430,5430,5363,5363,5362,5362,5361,5361,5358,5358,5350,5350,5330,5330,5312,5312,5287,5287,5283,5283,5275,5275,5261,5261,5247,5247,5233,5233,5226,5226,5207,5207,5188,5188,5183,5183,5182,5182,5169,5169,5160,5160,5158,5158,5153,5153,5149,5149,5140,5140,5136,5136,5134,5134,5132,5132,5121,5121,5108,5108,5093,5093,5086,5086,5085,5085,5069,5069,5068,5068,5058,5058,5057,5057,5036,5036,5032,5032,5027,5027,5012,5012,5006,5006,4984,4984,4964,4964,4951,4951,4945,4945,4933,4933,4920,4920,4913,4913,4911,4911,4910,4910,4907,4907,4902,4902,4901,4901,4883,4883,4880,4880,4861,4861,4837,4837,4810,4810,4808,4808,4798,4798,4797,4797,4783,4783,4777,4777,4769,4769,4751,4751,4707,4707,4678,4678,4674,4674,4666,4666,4655,4655,4648,4648,4627,4627,4625,4625,4584,4584,4570,4570,4566,4566,4551,4551,4532,4532,4530,4530,4527,4527,4506,4506,4492,4492,4486,4486,4482,4482,4471,4471,4469,4469,4431,4431,4421,4421,4403,4403,4402,4402,4370,4370,4353,4353,4344,4344,4330,4330,4321,4321,4310,4310,4299,4299,4258,4258,4245,4245,4238,4238,4236,4236,4224,4224,4213,4213,4206,4206,4204,4204,4200,4200,4170,4170,4167,4167,4165,4165,4134,4134,4113,4113,4105,4105,4081,4081,4033,4033,4026,4026,4001,4001,3999,3999,3993,3993,3988,3988,3984,3984,3981,3981,3979,3979,3972,3972,3971,3971,3969,3969,3966,3966,3962,3962,3961,3961,3955,3955,3951,3951,3942,3942,3933,3933,3932,3932,3931,3931,3928,3928,3924,3924,3923,3923,3920,3920,3901,3901,3897,3897,3893,3893,3885,3885,3828,3828,3807,3807,3799,3799,3795,3795,3793,3793,3789,3789,3784,3784,3771,3771,3747,3747,3743,3743,3742,3742,3724,3724,3713,3713,3698,3698,3690,3690,3664,3664,3651,3651,3642,3642,3627,3627,3606,3606,3596,3596,3582,3582,3581,3581,3567,3567,3552,3552,3546,3546,3538,3538,3537,3537,3536,3536,3530,3530,3523,3523,3514,3514,3512,3512,3496,3496,3478,3478,3462,3462,3458,3458,3444,3444,3440,3440,3399,3399,3369,3369,3366,3366,3356,3356,3349,3349,3346,3346,3341,3341,3309,3309,3307,3307,3297,3297,3294,3294,3282,3282,3269,3269,3262,3262,3261,3261,3260,3260,3258,3258,3256,3256,3254,3254,3244,3244,3231,3231,3225,3225,3221,3221,3217,3217,3203,3203,3201,3201,3195,3195,3194,3194,3178,3178,3169,3169,3167,3167,3165,3165,3163,3163,3151,3151,3150,3150,3144,3144,3131,3131,3121,3121,3119,3119,3112,3112,3086,3086,3069,3069,3065,3065,3060,3060,3043,3043,3038,3038,3028,3028,3023,3023,3019,3019,3017,3017,3001,3001,2981,2981,2978,2978,2977,2977,2976,2976,2970,2970,2949,2949,2945,2945,2941,2941,2940,2940,2936,2936,2935,2935,2928,2928,2921,2921,2920,2920,2909,2909,2881,2881,2870,2870,2868,2868,2867,2867,2843,2843,2829,2829,2825,2825,2815,2815,2804,2804,2800,2800,2793,2793,2790,2790,2787,2787,2778,2778,2774,2774,2772,2772,2770,2770,2767,2767,2760,2760,2754,2754,2744,2744,2743,2743,2740,2740,2736,2736,2728,2728,2721,2721,2719,2719,2715,2715,2710,2710,2694,2694,2690,2690,2689,2689,2688,2688,2685,2685,2684,2684,2679,2679,2675,2675,2673,2673,2659,2659,2657,2657,2655,2655,2651,2651,2637,2637,2634,2634,2633,2633,2624,2624,2620,2620,2617,2617,2612,2612,2611,2611,2609,2609,2595,2595,2594,2594,2591,2591,2588,2588,2582,2582,2577,2577,2565,2565,2561,2561,2560,2560,2552,2552,2529,2529,2524,2524,2523,2523,2522,2522,2519,2519,2512,2512,2510,2510,2506,2506,2499,2499,2494,2494,2493,2493,2491,2491,2486,2486,2485,2485,2483,2483,2482,2482,2481,2481,2476,2476,2474,2474,2461,2461,2459,2459,2457,2457,2446,2446,2444,2444,2417,2417,2416,2416,2411,2411,2410,2410,2409,2409,2408,2408,2407,2407,2406,2406,2404,2404,2403,2403,2400,2400,2398,2398,2392,2392,2388,2388,2387,2387,2383,2383,2382,2382,2377,2377,2367,2367,2366,2366,2364,2364,2356,2356,2355,2355,2350,2350,2338,2338,2333,2333,2326,2326,2324,2324,2314,2314,2312,2312,2306,2306,2294,2294,2292,2292,2285,2285,2281,2281,2278,2278,2266,2266,2265,2265,2264,2264,2262,2262,2256,2256,2253,2253,2250,2250,2249,2249,2248,2248,2242,2242,2239,2239,2237,2237,2234,2234,2232,2232,2223,2223,2215,2215,2208,2208,2207,2207,2201,2201,2199,2199,2198,2198,2195,2195,2183,2183,2182,2182,2181,2181,2180,2180,2178,2178,2173,2173,2172,2172,2168,2168,2161,2161,2156,2156,2150,2150,2143,2143,2142,2142,2133,2133,2129,2129,2122,2122,2121,2121,2120,2120,2105,2105,2097,2097,2093,2093,2084,2084,2083,2083,2081,2081,2079,2079,2076,2076,2074,2074,2068,2068,2062,2062,2057,2057,2055,2055,2051,2051,2050,2050,2044,2044,2042,2042,2041,2041,2037,2037,2036,2036,2031,2031,2028,2028,2026,2026,2024,2024,2022,2022,2021,2021,2020,2020,2019,2019,2013,2013,2012,2012,2009,2009,1999,1999,1987,1987,1986,1986,1981,1981,1980,1980,1978,1978,1975,1975,1973,1973,1967,1967,1962,1962,1960,1960,1957,1957,1954,1954,1953,1953,1946,1946,1944,1944,1940,1940,1939,1939,1938,1938,1934,1934,1931,1931,1928,1928,1921,1921,1918,1918,1915,1915,1913,1913,1910,1910,1909,1909,1907,1907,1903,1903,1901,1901,1897,1897,1891,1891,1890,1890,1888,1888,1887,1887,1885,1885,1884,1884,1882,1882,1880,1880,1876,1876,1874,1874,1873,1873,1871,1871,1869,1869,1867,1867,1860,1860,1859,1859,1857,1857,1854,1854,1849,1849,1848,1848,1847,1847,1846,1846,1844,1844,1840,1840,1836,1836,1835,1835,1834,1834,1828,1828,1826,1826,1814,1814,1810,1810,1809,1809,1806,1806,1805,1805,1804,1804,1803,1803,1802,1802,1799,1799,1798,1798,1796,1796,1795,1795,1794,1794,1791,1791,1790,1790,1789,1789,1788,1788,1786,1786,1782,1782,1780,1780,1775,1775,1774,1774,1773,1773,1772,1772,1769,1769,1767,1767,1766,1766,1765,1765,1763,1763,1757,1757,1752,1752,1748,1748,1739,1739,1736,1736,1734,1734,1733,1733,1731,1731,1729,1729,1728,1728,1727,1727,1724,1724,1723,1723,1722,1722,1721,1721,1717,1717,1712,1712,1711,1711,1710,1710,1706,1706,1705,1705,1703,1703,1697,1697,1696,1696,1695,1695,1694,1694,1693,1693,1691,1691,1686,1686,1676,1676,1675,1675,1673,1673,1671,1671,1667,1667,1666,1666,1663,1663,1662,1662,1661,1661,1656,1656,1655,1655,1653,1653,1652,1652,1651,1651,1649,1649,1648,1648,1647,1647,1644,1644,1640,1640,1639,1639,1636,1636,1633,1633,1631,1631,1629,1629,1628,1628,1625,1625,1623,1623,1622,1622,1621,1621,1619,1619,1617,1617,1616,1616,1614,1614,1613,1613,1610,1610,1609,1609,1608,1608,1604,1604,1601,1601,1600,1600,1598,1598,1593,1593,1587,1587,1585,1585,1581,1581,1580,1580,1579,1579,1578,1578,1577,1577,1573,1573,1570,1570,1567,1567,1564,1564,1562,1562,1561,1561,1558,1558,1554,1554,1552,1552,1549,1549,1548,1548,1547,1547,1541,1541,1539,1539,1538,1538,1536,1536,1535,1535,1533,1533,1531,1531,1530,1530,1529,1529,1526,1526,1525,1525,1522,1522,1519,1519,1516,1516,1512,1512,1510,1510,1509,1509,1508,1508,1503,1503,1502,1502,1501,1501,1499,1499,1498,1498,1496,1496,1495,1495,1494,1494,1493,1493,1492,1492,1490,1490,1486,1486,1481,1481,1480,1480,1477,1477,1472,1472,1470,1470,1469,1469,1468,1468,1465,1465,1460,1460,1456,1456,1455,1455,1454,1454,1451,1451,1447,1447,1446,1446,1445,1445,1444,1444,1442,1442,1438,1438,1436,1436,1434,1434,1433,1433,1432,1432,1431,1431,1427,1427,1426,1426,1425,1425,1420,1420,1418,1418,1417,1417,1414,1414,1412,1412,1411,1411,1410,1410,1408,1408,1406,1406,1400,1400,1399,1399,1397,1397,1393,1393,1388,1388,1387,1387,1384,1384,1383,1383,1382,1382,1378,1378,1374,1374,1371,1371,1370,1370,1369,1369,1364,1364,1362,1362,1361,1361,1356,1356,1355,1355,1354,1354,1352,1352,1349,1349,1344,1344,1343,1343,1342,1342,1336,1336,1333,1333,1332,1332,1330,1330,1329,1329,1328,1328,1326,1326,1324,1324,1323,1323,1322,1322,1321,1321,1320,1320,1318,1318,1317,1317,1313,1313,1312,1312,1309,1309,1307,1307,1306,1306,1303,1303,1302,1302,1301,1301,1299,1299,1298,1298,1294,1294,1292,1292,1291,1291,1290,1290,1289,1289,1288,1288,1285,1285,1283,1283,1280,1280,1279,1279,1278,1278,1276,1276,1275,1275,1274,1274,1273,1273,1272,1272,1271,1271,1270,1270,1269,1269,1267,1267,1266,1266,1264,1264,1261,1261,1260,1260,1259,1259,1258,1258,1257,1257,1256,1256,1254,1254,1253,1253,1252,1252,1249,1249,1248,1248,1247,1247,1243,1243,1242,1242,1240,1240,1239,1239,1235,1235,1234,1234,1233,1233,1232,1232,1231,1231,1230,1230,1229,1229,1227,1227,1226,1226,1224,1224,1223,1223,1222,1222,1221,1221,1220,1220,1219,1219,1217,1217,1216,1216,1215,1215,1214,1214,1209,1209,1208,1208,1207,1207,1206,1206,1205,1205,1204,1204,1203,1203,1201,1201,1200,1200,1199,1199,1198,1198,1197,1197,1193,1193,1192,1192,1191,1191,1190,1190,1189,1189,1188,1188,1187,1187,1184,1184,1183,1183,1182,1182,1181,1181,1179,1179,1177,1177,1175,1175,1174,1174,1173,1173,1172,1172,1171,1171,1170,1170,1169,1169,1168,1168,1167,1167,1166,1166,1163,1163,1162,1162,1161,1161,1159,1159,1158,1158,1157,1157,1156,1156,1155,1155,1154,1154,1152,1152,1150,1150,1149,1149,1148,1148,1147,1147,1146,1146,1145,1145,1144,1144,1143,1143,1142,1142,1139,1139,1136,1136,1135,1135,1134,1134,1132,1132,1131,1131,1129,1129,1128,1128,1126,1126,1125,1125,1124,1124,1123,1123,1122,1122,1121,1121,1120,1120,1119,1119,1118,1118,1117,1117,1116,1116,1115,1115,1114,1114,1113,1113,1112,1112,1111,1111,1110,1110,1109,1109,1108,1108,1106,1106,1105,1105,1104,1104,1103,1103,1102,1102,1101,1101,1100,1100,1099,1099,1098,1098,1096,1096,1095,1095,1094,1094,1093,1093,1092,1092,1089,1089,1088,1088,1087,1087,1086,1086,1084,1084,1083,1083,1082,1082,1081,1081,1079,1079,1078,1078,1076,1076,1075,1075,1074,1074,1073,1073,1072,1072,1071,1071,1070,1070,1069,1069,1067,1067,1066,1066,1065,1065,1064,1064,1063,1063,1061,1061,1059,1059,1057,1057,1056,1056,1055,1055,1054,1054,1053,1053,1052,1052,1051,1051,1050,1050,1049,1049,1048,1048,1046,1046,1045,1045,1044,1044,1043,1043,1042,1042,1041,1041,1040,1040,1039,1039,1038,1038,1037,1037,1035,1035,1034,1034,1033,1033,1032,1032,1031,1031,1030,1030,1029,1029,1028,1028,1027,1027,1026,1026,1024,1024,1022,1022,1020,1020,1019,1019,1018,1018,1017,1017,1016,1016,1014,1014,1013,1013,1012,1012,1010,1010,1009,1009,1008,1008,1007,1007,1006,1006,1005,1005,1004,1004,1003,1003,1002,1002,1001,1001,1000,1000,999,999,998,998,997,997,994,994,993,993,992,992,991,991,989,989,988,988,987,987,985,985,983,983,982,982,981,981,980,980,979,979,978,978,977,977,976,976,975,975,973,973,972,972,971,971,970,970,969,969,968,968,967,967,966,966,964,964,963,963,962,962,961,961,960,960,959,959,958,958,956,956,955,955,954,954,952,952,951,951,950,950,949,949,948,948,947,947,946,946,945,945,944,944,943,943,942,942,941,941,940,940,939,939,938,938,936,936,934,934,933,933,932,932,931,931,930,930,929,929,928,928,927,927,926,926,925,925,924,924,923,923,922,922,921,921,920,920,919,919,918,918,917,917,916,916,915,915,914,914,913,913,912,912,911,911,910,910,909,909,908,908,907,907,906,906,905,905,904,904,903,903,902,902,901,901,900,900,899,899,898,898,897,897,896,896,895,895,893,893,892,892,891,891,890,890,889,889,888,888,887,887,886,886,885,885,884,884,882,882,881,881,880,880,879,879,878,878,877,877,876,876,875,875,874,874,873,873,872,872,871,871,870,870,869,869,868,868,867,867,866,866,865,865,864,864,863,863,862,862,861,861,860,860,859,859,857,857,856,856,855,855,854,854,853,853,852,852,851,851,850,850,849,849,848,848,847,847,846,846,845,845,844,844,843,843,842,842,841,841,840,840,839,839,838,838,837,837,836,836,835,835,834,834,833,833,832,832,831,831,829,829,828,828,827,827,826,826,825,825,824,824,823,823,822,822,821,821,820,820,819,819,818,818,817,817,816,816,815,815,813,813,812,812,811,811,810,810,809,809,808,808,807,807,805,805,804,804,803,803,802,802,801,801,799,799,798,798,797,797,796,796,795,795,794,794,793,793,792,792,791,791,790,790,789,789,787,787,786,786,785,785,784,784,782,782,781,781,780,780,779,779,778,778,777,777,776,776,775,775,774,774,773,773,772,772,771,771,770,770,769,769,768,768,767,767,766,766,765,765,764,764,763,763,762,762,761,761,760,760,759,759,758,758,757,757,756,756,754,754,753,753,752,752,751,751,750,750,749,749,748,748,747,747,746,746,745,745,744,744,743,743,742,742,741,741,740,740,739,739,738,738,737,737,736,736,735,735,734,734,733,733,732,732,730,730,729,729,728,728,726,726,725,725,724,724,722,722,721,721,720,720,719,719,718,718,717,717,716,716,715,715,714,714,713,713,712,712,711,711,710,710,709,709,708,708,707,707,706,706,705,705,703,703,702,702,701,701,700,700,699,699,698,698,697,697,696,696,695,695,694,694,693,693,692,692,691,691,690,690,689,689,688,688,687,687,686,686,685,685,684,684,683,683,682,682,681,681,680,680,679,679,678,678,677,677,676,676,675,675,674,674,673,673,672,672,671,671,670,670,669,669,668,668,667,667,666,666,665,665,664,664,663,663,662,662,661,661,660,660,658,658,657,657,656,656,655,655,654,654,653,653,652,652,651,651,650,650,649,649,648,648,647,647,646,646,645,645,644,644,643,643,642,642,640,640,639,639,638,638,637,637,636,636,634,634,633,633,632,632,631,631,630,630,629,629,628,628,627,627,626,626,625,625,624,624,623,623,622,622,621,621,620,620,619,619,618,618,615,615,614,614,613,613,612,612,611,611,610,610,606,606,605,605,604,604,602,602,601,601,597,597,595,595,594,594,592,592,590,590,589,589,588,588,587,587,586,586,584,584,583,583,582,582,580,580,578,578,577,577,574,574,573,573,572,572,571,571,570,570,568,568,566,566,564,564,563,563,562,562,561,561,558,558,557,557,555,555,554,554,552,552,551,551,550,550,549,549,548,548,547,547,546,546,544,544,542,542,539,539,538,538,536,536,534,534,533,533,532,532,531,531,530,530,527,527,526,526,524,524,521,521,518,518,515,515,509,509,506,506,505,505,503,503,502,502,500,500,499,499,497,497,496,496,493,493,492,492,490,490,489,489,487,487,486,486,485,485,483,483,481,481,480,480,478,478,476,476,475,475,472,472,471,471,469,469,468,468,466,466,463,463,458,458,457,457,454,454,447,447,446,446,441,441,440,440,436,436,435,435,434,434,433,433,432,432,431,431,430,430,429,429,428,428,427,427,424,424,422,422,421,421,418,418,416,416,414,414,410,410,409,409,407,407,406,406,405,405,403,403,402,402,401,401,0.0],[157150,157150,157150,157077,157077,120630,120630,102562,102562,91333,91333,88313,88313,87787,87787,85906,85906,83907,83907,79385,79385,79295,79295,78612,78612,76466,76466,73786,73786,72807,72807,71598,71598,71370,71370,69874,69874,69846,69846,69629,69629,69329,69329,68492,68492,68456,68456,68335,68335,67386,67386,67118,67118,67102,67102,66158,66158,65580,65580,65579,65579,64902,64902,63900,63900,63296,63296,62369,62369,62159,62159,61678,61678,61625,61625,61233,61233,61149,61149,60908,60908,60589,60589,60413,60413,60096,60096,59831,59831,59809,59809,58859,58859,58674,58674,58414,58414,58386,58386,58152,58152,57541,57541,56779,56779,56753,56753,55788,55788,55670,55670,54872,54872,54810,54810,54748,54748,54177,54177,53761,53761,53411,53411,53381,53381,52502,52502,52198,52198,52058,52058,51912,51912,51743,51743,51742,51742,51137,51137,50940,50940,50747,50747,50699,50699,50460,50460,50203,50203,50107,50107,49904,49904,49846,49846,49609,49609,49381,49381,49174,49174,49011,49011,48399,48399,48359,48359,48172,48172,47988,47988,47595,47595,47560,47560,47443,47443,47404,47404,47095,47095,46896,46896,46371,46371,46199,46199,45997,45997,45534,45534,45528,45528,45330,45330,45174,45174,45085,45085,45046,45046,44787,44787,44687,44687,44677,44677,44504,44504,44227,44227,44184,44184,44017,44017,44003,44003,43716,43716,42831,42831,42682,42682,42519,42519,42229,42229,41879,41879,41853,41853,41791,41791,41745,41745,41441,41441,41191,41191,41110,41110,40952,40952,40852,40852,40766,40766,40531,40531,40468,40468,40368,40368,40161,40161,40024,40024,39831,39831,39577,39577,39562,39562,39535,39535,39495,39495,39145,39145,39005,39005,38886,38886,38841,38841,38788,38788,38675,38675,38177,38177,38149,38149,38107,38107,37830,37830,37799,37799,37713,37713,37602,37602,37506,37506,37478,37478,37219,37219,37142,37142,37023,37023,36753,36753,36197,36197,36189,36189,36149,36149,35859,35859,35827,35827,35618,35618,35550,35550,35137,35137,35123,35123,35111,35111,34967,34967,34913,34913,34842,34842,34837,34837,34815,34815,34801,34801,34667,34667,34660,34660,34432,34432,34242,34242,34240,34240,34232,34232,34003,34003,33968,33968,33967,33967,33966,33966,33852,33852,33751,33751,33666,33666,33617,33617,33582,33582,33581,33581,33524,33524,33454,33454,33439,33439,33379,33379,33377,33377,33376,33376,33246,33246,33207,33207,32937,32937,32917,32917,32903,32903,32714,32714,32660,32660,32349,32349,32241,32241,32201,32201,32159,32159,32134,32134,32102,32102,32034,32034,31938,31938,31912,31912,31709,31709,31599,31599,31477,31477,31376,31376,31340,31340,31329,31329,31325,31325,31269,31269,30973,30973,30956,30956,30934,30934,30824,30824,30814,30814,30727,30727,30719,30719,30559,30559,30352,30352,30305,30305,30245,30245,30081,30081,30050,30050,29993,29993,29922,29922,29895,29895,29830,29830,29819,29819,29788,29788,29653,29653,29646,29646,29604,29604,29524,29524,29468,29468,29262,29262,29238,29238,29218,29218,29194,29194,29148,29148,28936,28936,28841,28841,28839,28839,28731,28731,28699,28699,28559,28559,28547,28547,28484,28484,28429,28429,28406,28406,28405,28405,28355,28355,28150,28150,28002,28002,27977,27977,27926,27926,27851,27851,27814,27814,27811,27811,27633,27633,27476,27476,27455,27455,27412,27412,27333,27333,27309,27309,27268,27268,27226,27226,27225,27225,27085,27085,26943,26943,26941,26941,26923,26923,26919,26919,26892,26892,26867,26867,26754,26754,26746,26746,26602,26602,26591,26591,26511,26511,26488,26488,26479,26479,26474,26474,26321,26321,26244,26244,26228,26228,26205,26205,26198,26198,26191,26191,26178,26178,26174,26174,26143,26143,26067,26067,25987,25987,25967,25967,25817,25817,25783,25783,25726,25726,25721,25721,25716,25716,25667,25667,25661,25661,25484,25484,25388,25388,25287,25287,25284,25284,25258,25258,25195,25195,25169,25169,25122,25122,25090,25090,25084,25084,24980,24980,24956,24956,24906,24906,24877,24877,24844,24844,24839,24839,24828,24828,24775,24775,24690,24690,24618,24618,24609,24609,24568,24568,24540,24540,24539,24539,24536,24536,24393,24393,24321,24321,24277,24277,24248,24248,24244,24244,24222,24222,24192,24192,24177,24177,24171,24171,24137,24137,24113,24113,24079,24079,23988,23988,23855,23855,23844,23844,23810,23810,23657,23657,23654,23654,23651,23651,23583,23583,23535,23535,23493,23493,23475,23475,23456,23456,23437,23437,23422,23422,23405,23405,23380,23380,23351,23351,23235,23235,23193,23193,23155,23155,23133,23133,23129,23129,23105,23105,23098,23098,23006,23006,22982,22982,22953,22953,22758,22758,22749,22749,22668,22668,22664,22664,22595,22595,22589,22589,22588,22588,22573,22573,22566,22566,22547,22547,22333,22333,22282,22282,22275,22275,22268,22268,22255,22255,22247,22247,22190,22190,22188,22188,22144,22144,22112,22112,22088,22088,22083,22083,22026,22026,22015,22015,21973,21973,21962,21962,21942,21942,21936,21936,21924,21924,21898,21898,21884,21884,21873,21873,21859,21859,21845,21845,21808,21808,21804,21804,21795,21795,21791,21791,21729,21729,21726,21726,21704,21704,21619,21619,21583,21583,21546,21546,21541,21541,21536,21536,21434,21434,21412,21412,21331,21331,21326,21326,21310,21310,21291,21291,21271,21271,21246,21246,21221,21221,21209,21209,21114,21114,21096,21096,21088,21088,21080,21080,21059,21059,21041,21041,21016,21016,21010,21010,20928,20928,20893,20893,20846,20846,20773,20773,20770,20770,20763,20763,20699,20699,20695,20695,20691,20691,20674,20674,20648,20648,20620,20620,20613,20613,20609,20609,20604,20604,20602,20602,20560,20560,20552,20552,20532,20532,20484,20484,20469,20469,20461,20461,20445,20445,20442,20442,20421,20421,20398,20398,20344,20344,20328,20328,20313,20313,20237,20237,20236,20236,20228,20228,20226,20226,20222,20222,20180,20180,20157,20157,20130,20130,20123,20123,20077,20077,20073,20073,20004,20004,20001,20001,19967,19967,19966,19966,19955,19955,19931,19931,19930,19930,19884,19884,19867,19867,19830,19830,19770,19770,19752,19752,19739,19739,19730,19730,19685,19685,19642,19642,19596,19596,19581,19581,19548,19548,19532,19532,19498,19498,19485,19485,19480,19480,19448,19448,19416,19416,19406,19406,19387,19387,19358,19358,19319,19319,19292,19292,19222,19222,19162,19162,19158,19158,19149,19149,19147,19147,19143,19143,19116,19116,19094,19094,19088,19088,19056,19056,19038,19038,19026,19026,19018,19018,18991,18991,18973,18973,18965,18965,18959,18959,18941,18941,18939,18939,18926,18926,18903,18903,18902,18902,18837,18837,18800,18800,18788,18788,18735,18735,18734,18734,18679,18679,18658,18658,18640,18640,18635,18635,18630,18630,18623,18623,18613,18613,18604,18604,18593,18593,18535,18535,18511,18511,18485,18485,18470,18470,18459,18459,18452,18452,18354,18354,18333,18333,18312,18312,18304,18304,18293,18293,18286,18286,18277,18277,18264,18264,18258,18258,18254,18254,18247,18247,18227,18227,18225,18225,18118,18118,18111,18111,18086,18086,18083,18083,18057,18057,18041,18041,18029,18029,17995,17995,17982,17982,17961,17961,17947,17947,17904,17904,17899,17899,17857,17857,17852,17852,17824,17824,17809,17809,17777,17777,17761,17761,17748,17748,17745,17745,17713,17713,17701,17701,17695,17695,17694,17694,17685,17685,17660,17660,17659,17659,17639,17639,17548,17548,17543,17543,17542,17542,17512,17512,17504,17504,17487,17487,17474,17474,17464,17464,17431,17431,17392,17392,17334,17334,17305,17305,17256,17256,17253,17253,17242,17242,17216,17216,17214,17214,17196,17196,17176,17176,17158,17158,17147,17147,17115,17115,17113,17113,17102,17102,17096,17096,17084,17084,17036,17036,16998,16998,16993,16993,16987,16987,16982,16982,16960,16960,16953,16953,16908,16908,16859,16859,16806,16806,16804,16804,16782,16782,16777,16777,16757,16757,16745,16745,16732,16732,16706,16706,16695,16695,16607,16607,16605,16605,16548,16548,16547,16547,16539,16539,16503,16503,16475,16475,16456,16456,16424,16424,16394,16394,16383,16383,16377,16377,16368,16368,16356,16356,16336,16336,16324,16324,16313,16313,16298,16298,16271,16271,16265,16265,16243,16243,16230,16230,16216,16216,16179,16179,16170,16170,16157,16157,16136,16136,16134,16134,16130,16130,16111,16111,16106,16106,16085,16085,16075,16075,16066,16066,16065,16065,16008,16008,16006,16006,15981,15981,15975,15975,15968,15968,15965,15965,15961,15961,15915,15915,15913,15913,15870,15870,15857,15857,15848,15848,15833,15833,15817,15817,15803,15803,15802,15802,15798,15798,15785,15785,15778,15778,15764,15764,15761,15761,15730,15730,15657,15657,15634,15634,15633,15633,15628,15628,15618,15618,15605,15605,15582,15582,15552,15552,15541,15541,15502,15502,15486,15486,15460,15460,15456,15456,15431,15431,15421,15421,15412,15412,15409,15409,15378,15378,15351,15351,15327,15327,15320,15320,15307,15307,15279,15279,15273,15273,15255,15255,15228,15228,15204,15204,15193,15193,15190,15190,15180,15180,15177,15177,15176,15176,15173,15173,15161,15161,15138,15138,15113,15113,15101,15101,15093,15093,15092,15092,15087,15087,15083,15083,15058,15058,15034,15034,15020,15020,15001,15001,14987,14987,14980,14980,14972,14972,14969,14969,14967,14967,14940,14940,14938,14938,14924,14924,14886,14886,14879,14879,14836,14836,14823,14823,14782,14782,14772,14772,14771,14771,14761,14761,14726,14726,14704,14704,14703,14703,14695,14695,14642,14642,14629,14629,14625,14625,14610,14610,14601,14601,14597,14597,14593,14593,14550,14550,14544,14544,14531,14531,14530,14530,14523,14523,14510,14510,14507,14507,14503,14503,14492,14492,14488,14488,14484,14484,14452,14452,14446,14446,14429,14429,14419,14419,14399,14399,14387,14387,14374,14374,14368,14368,14362,14362,14340,14340,14305,14305,14263,14263,14261,14261,14248,14248,14240,14240,14234,14234,14219,14219,14211,14211,14210,14210,14150,14150,14131,14131,14122,14122,14101,14101,14094,14094,14072,14072,14065,14065,14055,14055,14054,14054,14045,14045,14044,14044,14032,14032,14011,14011,13996,13996,13992,13992,13978,13978,13956,13956,13955,13955,13943,13943,13929,13929,13911,13911,13909,13909,13871,13871,13864,13864,13862,13862,13850,13850,13795,13795,13793,13793,13790,13790,13764,13764,13756,13756,13750,13750,13743,13743,13725,13725,13717,13717,13700,13700,13695,13695,13688,13688,13680,13680,13642,13642,13638,13638,13635,13635,13602,13602,13600,13600,13598,13598,13572,13572,13542,13542,13532,13532,13500,13500,13460,13460,13456,13456,13453,13453,13445,13445,13438,13438,13430,13430,13422,13422,13417,13417,13390,13390,13388,13388,13368,13368,13362,13362,13354,13354,13337,13337,13333,13333,13319,13319,13316,13316,13313,13313,13312,13312,13299,13299,13294,13294,13291,13291,13286,13286,13284,13284,13283,13283,13278,13278,13260,13260,13248,13248,13213,13213,13212,13212,13207,13207,13204,13204,13179,13179,13160,13160,13156,13156,13132,13132,13130,13130,13117,13117,13114,13114,13113,13113,13104,13104,13086,13086,13084,13084,13080,13080,13077,13077,13076,13076,13066,13066,13065,13065,13057,13057,13055,13055,13052,13052,12999,12999,12994,12994,12981,12981,12947,12947,12936,12936,12935,12935,12931,12931,12928,12928,12909,12909,12901,12901,12871,12871,12838,12838,12818,12818,12809,12809,12802,12802,12801,12801,12796,12796,12790,12790,12781,12781,12773,12773,12758,12758,12747,12747,12739,12739,12737,12737,12692,12692,12688,12688,12685,12685,12661,12661,12645,12645,12636,12636,12625,12625,12599,12599,12591,12591,12569,12569,12558,12558,12540,12540,12521,12521,12514,12514,12497,12497,12490,12490,12486,12486,12476,12476,12463,12463,12454,12454,12450,12450,12444,12444,12442,12442,12438,12438,12434,12434,12422,12422,12420,12420,12419,12419,12400,12400,12386,12386,12380,12380,12364,12364,12359,12359,12358,12358,12350,12350,12336,12336,12335,12335,12305,12305,12273,12273,12269,12269,12247,12247,12243,12243,12229,12229,12225,12225,12219,12219,12196,12196,12177,12177,12175,12175,12162,12162,12158,12158,12138,12138,12135,12135,12134,12134,12124,12124,12123,12123,12115,12115,12098,12098,12076,12076,12064,12064,12056,12056,12054,12054,12043,12043,12033,12033,12023,12023,12005,12005,11999,11999,11992,11992,11990,11990,11986,11986,11983,11983,11975,11975,11973,11973,11971,11971,11966,11966,11961,11961,11951,11951,11932,11932,11923,11923,11908,11908,11894,11894,11888,11888,11874,11874,11863,11863,11857,11857,11855,11855,11827,11827,11820,11820,11809,11809,11796,11796,11790,11790,11771,11771,11770,11770,11760,11760,11747,11747,11744,11744,11743,11743,11734,11734,11729,11729,11723,11723,11719,11719,11711,11711,11704,11704,11699,11699,11654,11654,11647,11647,11639,11639,11637,11637,11616,11616,11612,11612,11602,11602,11595,11595,11590,11590,11576,11576,11574,11574,11554,11554,11552,11552,11527,11527,11526,11526,11508,11508,11482,11482,11479,11479,11475,11475,11473,11473,11468,11468,11460,11460,11456,11456,11449,11449,11448,11448,11443,11443,11426,11426,11425,11425,11420,11420,11412,11412,11409,11409,11406,11406,11391,11391,11390,11390,11376,11376,11357,11357,11356,11356,11342,11342,11336,11336,11333,11333,11329,11329,11325,11325,11316,11316,11313,11313,11309,11309,11307,11307,11302,11302,11294,11294,11288,11288,11273,11273,11257,11257,11249,11249,11246,11246,11244,11244,11234,11234,11225,11225,11223,11223,11217,11217,11207,11207,11206,11206,11201,11201,11164,11164,11160,11160,11144,11144,11140,11140,11138,11138,11130,11130,11116,11116,11099,11099,11095,11095,11082,11082,11079,11079,11077,11077,11071,11071,11070,11070,11067,11067,11066,11066,11057,11057,11051,11051,11048,11048,11040,11040,11031,11031,11019,11019,11016,11016,11011,11011,11009,11009,11008,11008,11001,11001,10995,10995,10986,10986,10980,10980,10977,10977,10947,10947,10944,10944,10941,10941,10938,10938,10929,10929,10910,10910,10892,10892,10889,10889,10881,10881,10879,10879,10870,10870,10866,10866,10865,10865,10863,10863,10862,10862,10860,10860,10859,10859,10858,10858,10849,10849,10843,10843,10837,10837,10830,10830,10823,10823,10820,10820,10816,10816,10810,10810,10808,10808,10807,10807,10789,10789,10784,10784,10781,10781,10766,10766,10740,10740,10709,10709,10687,10687,10666,10666,10661,10661,10625,10625,10617,10617,10615,10615,10613,10613,10599,10599,10589,10589,10585,10585,10584,10584,10577,10577,10561,10561,10536,10536,10534,10534,10522,10522,10520,10520,10504,10504,10498,10498,10494,10494,10484,10484,10472,10472,10465,10465,10460,10460,10453,10453,10445,10445,10430,10430,10419,10419,10415,10415,10414,10414,10402,10402,10398,10398,10395,10395,10390,10390,10377,10377,10374,10374,10367,10367,10352,10352,10345,10345,10338,10338,10326,10326,10325,10325,10323,10323,10317,10317,10314,10314,10311,10311,10305,10305,10304,10304,10285,10285,10277,10277,10272,10272,10266,10266,10263,10263,10262,10262,10257,10257,10255,10255,10252,10252,10250,10250,10249,10249,10240,10240,10239,10239,10236,10236,10232,10232,10224,10224,10218,10218,10217,10217,10196,10196,10195,10195,10184,10184,10182,10182,10164,10164,10158,10158,10121,10121,10118,10118,10116,10116,10115,10115,10114,10114,10113,10113,10112,10112,10109,10109,10107,10107,10105,10105,10102,10102,10101,10101,10100,10100,10085,10085,10082,10082,10077,10077,10075,10075,10071,10071,10050,10050,10046,10046,10028,10028,10007,10007,9994,9994,9981,9981,9978,9978,9974,9974,9971,9971,9961,9961,9955,9955,9948,9948,9938,9938,9929,9929,9919,9919,9915,9915,9907,9907,9904,9904,9898,9898,9875,9875,9865,9865,9851,9851,9848,9848,9847,9847,9845,9845,9840,9840,9836,9836,9824,9824,9814,9814,9803,9803,9800,9800,9799,9799,9797,9797,9794,9794,9793,9793,9788,9788,9775,9775,9768,9768,9765,9765,9764,9764,9757,9757,9747,9747,9746,9746,9719,9719,9707,9707,9690,9690,9684,9684,9681,9681,9679,9679,9671,9671,9669,9669,9638,9638,9617,9617,9616,9616,9613,9613,9589,9589,9581,9581,9575,9575,9554,9554,9553,9553,9550,9550,9547,9547,9532,9532,9531,9531,9521,9521,9519,9519,9513,9513,9505,9505,9495,9495,9489,9489,9488,9488,9487,9487,9485,9485,9482,9482,9479,9479,9471,9471,9462,9462,9461,9461,9458,9458,9457,9457,9455,9455,9453,9453,9451,9451,9449,9449,9440,9440,9435,9435,9425,9425,9415,9415,9402,9402,9401,9401,9393,9393,9392,9392,9385,9385,9378,9378,9374,9374,9365,9365,9364,9364,9362,9362,9361,9361,9353,9353,9351,9351,9350,9350,9348,9348,9332,9332,9323,9323,9308,9308,9303,9303,9298,9298,9285,9285,9282,9282,9278,9278,9274,9274,9263,9263,9242,9242,9240,9240,9236,9236,9231,9231,9230,9230,9222,9222,9197,9197,9189,9189,9181,9181,9179,9179,9178,9178,9174,9174,9125,9125,9124,9124,9122,9122,9094,9094,9086,9086,9085,9085,9081,9081,9078,9078,9073,9073,9067,9067,9059,9059,9050,9050,9047,9047,9046,9046,9037,9037,9034,9034,9016,9016,9005,9005,8994,8994,8990,8990,8979,8979,8976,8976,8974,8974,8970,8970,8962,8962,8955,8955,8948,8948,8945,8945,8932,8932,8930,8930,8929,8929,8905,8905,8902,8902,8898,8898,8888,8888,8881,8881,8874,8874,8867,8867,8853,8853,8850,8850,8833,8833,8831,8831,8825,8825,8821,8821,8817,8817,8808,8808,8807,8807,8805,8805,8799,8799,8798,8798,8794,8794,8789,8789,8787,8787,8781,8781,8769,8769,8746,8746,8744,8744,8743,8743,8729,8729,8725,8725,8723,8723,8719,8719,8718,8718,8709,8709,8702,8702,8700,8700,8697,8697,8689,8689,8685,8685,8671,8671,8651,8651,8649,8649,8636,8636,8631,8631,8629,8629,8625,8625,8621,8621,8615,8615,8613,8613,8609,8609,8608,8608,8604,8604,8603,8603,8602,8602,8601,8601,8596,8596,8592,8592,8589,8589,8588,8588,8586,8586,8584,8584,8571,8571,8559,8559,8545,8545,8537,8537,8531,8531,8525,8525,8524,8524,8520,8520,8517,8517,8514,8514,8511,8511,8510,8510,8504,8504,8501,8501,8490,8490,8488,8488,8484,8484,8479,8479,8478,8478,8477,8477,8475,8475,8468,8468,8467,8467,8465,8465,8461,8461,8454,8454,8443,8443,8441,8441,8435,8435,8428,8428,8426,8426,8424,8424,8412,8412,8411,8411,8407,8407,8405,8405,8399,8399,8395,8395,8386,8386,8384,8384,8366,8366,8364,8364,8353,8353,8352,8352,8344,8344,8343,8343,8342,8342,8340,8340,8337,8337,8334,8334,8330,8330,8329,8329,8328,8328,8327,8327,8318,8318,8310,8310,8308,8308,8302,8302,8295,8295,8294,8294,8292,8292,8290,8290,8283,8283,8275,8275,8274,8274,8271,8271,8267,8267,8263,8263,8261,8261,8245,8245,8240,8240,8237,8237,8233,8233,8224,8224,8219,8219,8217,8217,8209,8209,8205,8205,8204,8204,8197,8197,8191,8191,8178,8178,8175,8175,8173,8173,8166,8166,8159,8159,8155,8155,8151,8151,8142,8142,8140,8140,8138,8138,8135,8135,8128,8128,8127,8127,8112,8112,8108,8108,8106,8106,8095,8095,8091,8091,8060,8060,8057,8057,8054,8054,8043,8043,8037,8037,8036,8036,8031,8031,8029,8029,8026,8026,8014,8014,8008,8008,8004,8004,7997,7997,7991,7991,7986,7986,7984,7984,7980,7980,7961,7961,7950,7950,7944,7944,7932,7932,7926,7926,7914,7914,7911,7911,7905,7905,7897,7897,7895,7895,7878,7878,7875,7875,7864,7864,7861,7861,7853,7853,7847,7847,7844,7844,7837,7837,7833,7833,7826,7826,7818,7818,7814,7814,7813,7813,7811,7811,7807,7807,7802,7802,7800,7800,7796,7796,7791,7791,7775,7775,7770,7770,7762,7762,7752,7752,7750,7750,7749,7749,7748,7748,7737,7737,7719,7719,7702,7702,7699,7699,7696,7696,7695,7695,7686,7686,7683,7683,7670,7670,7663,7663,7658,7658,7656,7656,7655,7655,7646,7646,7637,7637,7635,7635,7626,7626,7625,7625,7624,7624,7623,7623,7616,7616,7606,7606,7604,7604,7600,7600,7597,7597,7594,7594,7583,7583,7580,7580,7572,7572,7571,7571,7565,7565,7561,7561,7557,7557,7539,7539,7535,7535,7534,7534,7532,7532,7530,7530,7529,7529,7525,7525,7522,7522,7517,7517,7514,7514,7509,7509,7501,7501,7495,7495,7485,7485,7480,7480,7478,7478,7477,7477,7470,7470,7460,7460,7459,7459,7458,7458,7454,7454,7450,7450,7448,7448,7444,7444,7443,7443,7437,7437,7436,7436,7432,7432,7430,7430,7417,7417,7412,7412,7411,7411,7407,7407,7403,7403,7400,7400,7383,7383,7382,7382,7371,7371,7353,7353,7339,7339,7338,7338,7334,7334,7322,7322,7317,7317,7315,7315,7313,7313,7302,7302,7299,7299,7298,7298,7292,7292,7285,7285,7282,7282,7280,7280,7272,7272,7271,7271,7259,7259,7250,7250,7242,7242,7239,7239,7232,7232,7226,7226,7219,7219,7218,7218,7217,7217,7216,7216,7214,7214,7208,7208,7204,7204,7203,7203,7201,7201,7196,7196,7193,7193,7192,7192,7189,7189,7188,7188,7187,7187,7183,7183,7181,7181,7176,7176,7155,7155,7145,7145,7128,7128,7111,7111,7108,7108,7107,7107,7106,7106,7104,7104,7097,7097,7093,7093,7075,7075,7070,7070,7063,7063,7059,7059,7057,7057,7056,7056,7043,7043,7039,7039,7036,7036,7032,7032,7027,7027,7026,7026,7017,7017,7016,7016,7014,7014,7009,7009,7004,7004,6994,6994,6993,6993,6988,6988,6985,6985,6984,6984,6981,6981,6976,6976,6968,6968,6966,6966,6963,6963,6961,6961,6960,6960,6952,6952,6944,6944,6941,6941,6938,6938,6930,6930,6928,6928,6927,6927,6914,6914,6910,6910,6905,6905,6901,6901,6895,6895,6889,6889,6888,6888,6882,6882,6875,6875,6872,6872,6868,6868,6865,6865,6851,6851,6850,6850,6843,6843,6838,6838,6834,6834,6832,6832,6828,6828,6826,6826,6823,6823,6811,6811,6798,6798,6795,6795,6792,6792,6791,6791,6789,6789,6786,6786,6781,6781,6778,6778,6775,6775,6770,6770,6768,6768,6763,6763,6760,6760,6757,6757,6748,6748,6731,6731,6730,6730,6728,6728,6725,6725,6715,6715,6708,6708,6705,6705,6701,6701,6696,6696,6691,6691,6689,6689,6686,6686,6674,6674,6671,6671,6669,6669,6668,6668,6664,6664,6663,6663,6662,6662,6661,6661,6658,6658,6656,6656,6652,6652,6645,6645,6640,6640,6638,6638,6636,6636,6632,6632,6629,6629,6620,6620,6619,6619,6618,6618,6614,6614,6613,6613,6611,6611,6609,6609,6604,6604,6601,6601,6600,6600,6596,6596,6593,6593,6589,6589,6585,6585,6584,6584,6581,6581,6580,6580,6572,6572,6571,6571,6543,6543,6539,6539,6532,6532,6529,6529,6521,6521,6518,6518,6509,6509,6504,6504,6493,6493,6490,6490,6487,6487,6484,6484,6476,6476,6474,6474,6466,6466,6465,6465,6461,6461,6431,6431,6429,6429,6422,6422,6417,6417,6415,6415,6408,6408,6403,6403,6389,6389,6386,6386,6385,6385,6381,6381,6368,6368,6362,6362,6360,6360,6350,6350,6348,6348,6339,6339,6331,6331,6330,6330,6325,6325,6323,6323,6321,6321,6319,6319,6312,6312,6310,6310,6306,6306,6304,6304,6299,6299,6294,6294,6292,6292,6291,6291,6286,6286,6284,6284,6276,6276,6275,6275,6274,6274,6268,6268,6266,6266,6265,6265,6263,6263,6262,6262,6261,6261,6259,6259,6255,6255,6251,6251,6247,6247,6242,6242,6239,6239,6235,6235,6234,6234,6233,6233,6232,6232,6230,6230,6227,6227,6221,6221,6218,6218,6215,6215,6211,6211,6204,6204,6199,6199,6194,6194,6189,6189,6187,6187,6186,6186,6184,6184,6179,6179,6175,6175,6174,6174,6167,6167,6158,6158,6157,6157,6154,6154,6147,6147,6142,6142,6134,6134,6133,6133,6124,6124,6112,6112,6101,6101,6097,6097,6090,6090,6087,6087,6082,6082,6071,6071,6063,6063,6061,6061,6058,6058,6051,6051,6049,6049,6044,6044,6040,6040,6038,6038,6037,6037,6028,6028,6019,6019,6015,6015,6011,6011,6008,6008,6003,6003,5999,5999,5997,5997,5985,5985,5973,5973,5971,5971,5969,5969,5968,5968,5967,5967,5965,5965,5953,5953,5949,5949,5947,5947,5944,5944,5943,5943,5938,5938,5924,5924,5917,5917,5915,5915,5913,5913,5909,5909,5908,5908,5904,5904,5897,5897,5896,5896,5895,5895,5886,5886,5877,5877,5874,5874,5873,5873,5872,5872,5870,5870,5867,5867,5847,5847,5846,5846,5844,5844,5840,5840,5837,5837,5835,5835,5834,5834,5827,5827,5825,5825,5823,5823,5822,5822,5801,5801,5796,5796,5784,5784,5779,5779,5778,5778,5767,5767,5766,5766,5746,5746,5740,5740,5738,5738,5737,5737,5732,5732,5728,5728,5714,5714,5710,5710,5708,5708,5705,5705,5699,5699,5689,5689,5688,5688,5687,5687,5681,5681,5667,5667,5666,5666,5655,5655,5654,5654,5653,5653,5648,5648,5646,5646,5624,5624,5623,5623,5621,5621,5612,5612,5609,5609,5608,5608,5599,5599,5593,5593,5584,5584,5575,5575,5573,5573,5572,5572,5566,5566,5564,5564,5562,5562,5559,5559,5555,5555,5554,5554,5553,5553,5549,5549,5540,5540,5536,5536,5535,5535,5532,5532,5523,5523,5518,5518,5494,5494,5486,5486,5485,5485,5483,5483,5478,5478,5476,5476,5472,5472,5467,5467,5464,5464,5461,5461,5457,5457,5453,5453,5439,5439,5434,5434,5433,5433,5425,5425,5420,5420,5413,5413,5404,5404,5401,5401,5399,5399,5379,5379,5376,5376,5372,5372,5366,5366,5361,5361,5359,5359,5358,5358,5355,5355,5351,5351,5348,5348,5344,5344,5341,5341,5340,5340,5337,5337,5330,5330,5328,5328,5321,5321,5315,5315,5311,5311,5309,5309,5307,5307,5302,5302,5300,5300,5299,5299,5293,5293,5291,5291,5287,5287,5286,5286,5281,5281,5277,5277,5271,5271,5260,5260,5254,5254,5248,5248,5237,5237,5229,5229,5222,5222,5221,5221,5219,5219,5214,5214,5204,5204,5200,5200,5199,5199,5198,5198,5193,5193,5181,5181,5180,5180,5173,5173,5171,5171,5156,5156,5150,5150,5146,5146,5144,5144,5139,5139,5137,5137,5134,5134,5132,5132,5126,5126,5122,5122,5120,5120,5118,5118,5115,5115,5111,5111,5109,5109,5106,5106,5103,5103,5099,5099,5089,5089,5086,5086,5083,5083,5078,5078,5076,5076,5073,5073,5069,5069,5059,5059,5057,5057,5056,5056,5051,5051,5048,5048,5034,5034,5020,5020,5013,5013,5005,5005,5004,5004,5003,5003,5001,5001,5000,5000,4997,4997,4993,4993,4985,4985,4982,4982,4981,4981,4977,4977,4970,4970,4969,4969,4968,4968,4965,4965,4959,4959,4957,4957,4956,4956,4953,4953,4949,4949,4942,4942,4933,4933,4927,4927,4925,4925,4924,4924,4923,4923,4922,4922,4917,4917,4915,4915,4913,4913,4908,4908,4905,4905,4889,4889,4887,4887,4886,4886,4884,4884,4878,4878,4876,4876,4874,4874,4871,4871,4867,4867,4858,4858,4850,4850,4849,4849,4848,4848,4847,4847,4843,4843,4841,4841,4836,4836,4830,4830,4825,4825,4822,4822,4813,4813,4812,4812,4807,4807,4804,4804,4800,4800,4798,4798,4793,4793,4787,4787,4784,4784,4783,4783,4778,4778,4777,4777,4776,4776,4765,4765,4756,4756,4752,4752,4751,4751,4747,4747,4741,4741,4740,4740,4738,4738,4727,4727,4716,4716,4715,4715,4713,4713,4711,4711,4708,4708,4700,4700,4695,4695,4688,4688,4684,4684,4680,4680,4676,4676,4673,4673,4671,4671,4669,4669,4668,4668,4667,4667,4655,4655,4651,4651,4650,4650,4646,4646,4644,4644,4642,4642,4640,4640,4633,4633,4625,4625,4619,4619,4615,4615,4612,4612,4609,4609,4604,4604,4595,4595,4586,4586,4580,4580,4575,4575,4564,4564,4562,4562,4558,4558,4555,4555,4544,4544,4540,4540,4531,4531,4530,4530,4529,4529,4523,4523,4522,4522,4521,4521,4519,4519,4513,4513,4507,4507,4505,4505,4504,4504,4499,4499,4496,4496,4494,4494,4485,4485,4471,4471,4467,4467,4461,4461,4459,4459,4456,4456,4445,4445,4444,4444,4432,4432,4421,4421,4419,4419,4416,4416,4409,4409,4403,4403,4402,4402,4400,4400,4388,4388,4382,4382,4381,4381,4380,4380,4378,4378,4375,4375,4374,4374,4372,4372,4359,4359,4356,4356,4353,4353,4350,4350,4343,4343,4339,4339,4336,4336,4333,4333,4332,4332,4330,4330,4329,4329,4317,4317,4312,4312,4310,4310,4309,4309,4305,4305,4301,4301,4299,4299,4298,4298,4296,4296,4290,4290,4284,4284,4283,4283,4281,4281,4279,4279,4278,4278,4274,4274,4273,4273,4272,4272,4270,4270,4269,4269,4266,4266,4264,4264,4260,4260,4258,4258,4256,4256,4255,4255,4251,4251,4248,4248,4247,4247,4246,4246,4241,4241,4232,4232,4230,4230,4228,4228,4226,4226,4222,4222,4219,4219,4210,4210,4207,4207,4205,4205,4201,4201,4198,4198,4196,4196,4186,4186,4184,4184,4183,4183,4178,4178,4165,4165,4164,4164,4161,4161,4159,4159,4132,4132,4130,4130,4126,4126,4125,4125,4124,4124,4119,4119,4117,4117,4111,4111,4110,4110,4106,4106,4100,4100,4094,4094,4092,4092,4086,4086,4078,4078,4074,4074,4071,4071,4068,4068,4064,4064,4056,4056,4053,4053,4052,4052,4049,4049,4048,4048,4047,4047,4033,4033,4026,4026,4024,4024,4008,4008,3999,3999,3997,3997,3996,3996,3993,3993,3992,3992,3991,3991,3990,3990,3988,3988,3987,3987,3986,3986,3983,3983,3977,3977,3975,3975,3974,3974,3971,3971,3966,3966,3949,3949,3946,3946,3940,3940,3939,3939,3931,3931,3926,3926,3925,3925,3918,3918,3915,3915,3909,3909,3908,3908,3907,3907,3906,3906,3904,3904,3903,3903,3892,3892,3884,3884,3873,3873,3871,3871,3865,3865,3863,3863,3862,3862,3856,3856,3854,3854,3853,3853,3848,3848,3842,3842,3837,3837,3835,3835,3828,3828,3826,3826,3823,3823,3822,3822,3820,3820,3818,3818,3817,3817,3815,3815,3809,3809,3806,3806,3802,3802,3794,3794,3786,3786,3785,3785,3782,3782,3781,3781,3774,3774,3764,3764,3762,3762,3760,3760,3759,3759,3756,3756,3753,3753,3744,3744,3743,3743,3740,3740,3728,3728,3727,3727,3726,3726,3725,3725,3723,3723,3720,3720,3719,3719,3714,3714,3710,3710,3708,3708,3702,3702,3700,3700,3697,3697,3690,3690,3687,3687,3686,3686,3684,3684,3683,3683,3680,3680,3674,3674,3673,3673,3670,3670,3669,3669,3668,3668,3667,3667,3664,3664,3663,3663,3662,3662,3660,3660,3658,3658,3657,3657,3654,3654,3643,3643,3641,3641,3640,3640,3636,3636,3635,3635,3634,3634,3628,3628,3625,3625,3617,3617,3613,3613,3612,3612,3608,3608,3604,3604,3599,3599,3594,3594,3591,3591,3588,3588,3586,3586,3584,3584,3580,3580,3578,3578,3574,3574,3573,3573,3570,3570,3569,3569,3568,3568,3562,3562,3558,3558,3554,3554,3547,3547,3543,3543,3541,3541,3536,3536,3535,3535,3534,3534,3533,3533,3525,3525,3522,3522,3516,3516,3512,3512,3505,3505,3498,3498,3491,3491,3487,3487,3483,3483,3481,3481,3478,3478,3477,3477,3475,3475,3472,3472,3470,3470,3468,3468,3466,3466,3464,3464,3458,3458,3452,3452,3450,3450,3449,3449,3445,3445,3444,3444,3442,3442,3441,3441,3438,3438,3437,3437,3436,3436,3435,3435,3422,3422,3417,3417,3416,3416,3415,3415,3406,3406,3404,3404,3402,3402,3400,3400,3398,3398,3397,3397,3396,3396,3394,3394,3393,3393,3390,3390,3387,3387,3384,3384,3383,3383,3379,3379,3372,3372,3370,3370,3366,3366,3362,3362,3356,3356,3354,3354,3353,3353,3350,3350,3347,3347,3346,3346,3345,3345,3344,3344,3336,3336,3335,3335,3331,3331,3330,3330,3325,3325,3312,3312,3311,3311,3307,3307,3296,3296,3293,3293,3287,3287,3285,3285,3274,3274,3273,3273,3272,3272,3271,3271,3254,3254,3253,3253,3252,3252,3250,3250,3249,3249,3248,3248,3243,3243,3241,3241,3238,3238,3237,3237,3228,3228,3225,3225,3224,3224,3220,3220,3219,3219,3214,3214,3210,3210,3208,3208,3207,3207,3204,3204,3203,3203,3202,3202,3201,3201,3197,3197,3194,3194,3193,3193,3190,3190,3185,3185,3183,3183,3182,3182,3180,3180,3175,3175,3173,3173,3171,3171,3170,3170,3163,3163,3157,3157,3155,3155,3154,3154,3152,3152,3147,3147,3146,3146,3141,3141,3138,3138,3132,3132,3126,3126,3125,3125,3120,3120,3114,3114,3113,3113,3107,3107,3101,3101,3095,3095,3093,3093,3091,3091,3090,3090,3089,3089,3088,3088,3084,3084,3076,3076,3068,3068,3066,3066,3059,3059,3057,3057,3056,3056,3054,3054,3051,3051,3050,3050,3049,3049,3048,3048,3047,3047,3044,3044,3043,3043,3042,3042,3040,3040,3038,3038,3033,3033,3031,3031,3030,3030,3028,3028,3025,3025,3024,3024,3022,3022,3021,3021,3020,3020,3019,3019,3018,3018,3016,3016,3010,3010,3005,3005,3002,3002,2996,2996,2995,2995,2988,2988,2986,2986,2984,2984,2982,2982,2978,2978,2977,2977,2976,2976,2975,2975,2963,2963,2962,2962,2960,2960,2958,2958,2957,2957,2953,2953,2952,2952,2950,2950,2942,2942,2941,2941,2939,2939,2936,2936,2935,2935,2933,2933,2921,2921,2920,2920,2915,2915,2913,2913,2912,2912,2907,2907,2906,2906,2904,2904,2900,2900,2898,2898,2896,2896,2890,2890,2888,2888,2887,2887,2879,2879,2876,2876,2874,2874,2871,2871,2863,2863,2861,2861,2859,2859,2858,2858,2857,2857,2856,2856,2855,2855,2849,2849,2848,2848,2846,2846,2844,2844,2840,2840,2834,2834,2833,2833,2832,2832,2830,2830,2829,2829,2826,2826,2823,2823,2822,2822,2820,2820,2817,2817,2816,2816,2810,2810,2807,2807,2804,2804,2799,2799,2797,2797,2792,2792,2791,2791,2786,2786,2784,2784,2782,2782,2781,2781,2777,2777,2776,2776,2769,2769,2767,2767,2765,2765,2764,2764,2763,2763,2749,2749,2748,2748,2747,2747,2746,2746,2739,2739,2738,2738,2733,2733,2730,2730,2729,2729,2728,2728,2725,2725,2724,2724,2722,2722,2720,2720,2713,2713,2704,2704,2702,2702,2701,2701,2699,2699,2698,2698,2696,2696,2689,2689,2687,2687,2686,2686,2685,2685,2681,2681,2679,2679,2673,2673,2672,2672,2671,2671,2670,2670,2668,2668,2667,2667,2665,2665,2664,2664,2663,2663,2661,2661,2660,2660,2657,2657,2655,2655,2653,2653,2652,2652,2651,2651,2646,2646,2642,2642,2639,2639,2638,2638,2636,2636,2635,2635,2634,2634,2633,2633,2632,2632,2630,2630,2628,2628,2627,2627,2620,2620,2619,2619,2613,2613,2607,2607,2606,2606,2603,2603,2602,2602,2599,2599,2598,2598,2597,2597,2595,2595,2590,2590,2587,2587,2586,2586,2582,2582,2575,2575,2570,2570,2565,2565,2562,2562,2560,2560,2557,2557,2555,2555,2553,2553,2552,2552,2550,2550,2549,2549,2548,2548,2545,2545,2543,2543,2541,2541,2538,2538,2537,2537,2536,2536,2535,2535,2534,2534,2531,2531,2522,2522,2519,2519,2514,2514,2506,2506,2503,2503,2502,2502,2498,2498,2494,2494,2493,2493,2492,2492,2490,2490,2486,2486,2485,2485,2481,2481,2474,2474,2473,2473,2472,2472,2470,2470,2461,2461,2458,2458,2454,2454,2447,2447,2446,2446,2441,2441,2436,2436,2430,2430,2429,2429,2425,2425,2422,2422,2419,2419,2418,2418,2417,2417,2414,2414,2413,2413,2411,2411,2405,2405,2404,2404,2403,2403,2402,2402,2401,2401,2400,2400,2399,2399,2393,2393,2388,2388,2387,2387,2385,2385,2382,2382,2380,2380,2379,2379,2376,2376,2375,2375,2371,2371,2366,2366,2365,2365,2362,2362,2361,2361,2359,2359,2358,2358,2357,2357,2350,2350,2344,2344,2342,2342,2340,2340,2338,2338,2335,2335,2334,2334,2331,2331,2328,2328,2326,2326,2324,2324,2320,2320,2318,2318,2316,2316,2309,2309,2307,2307,2292,2292,2286,2286,2284,2284,2283,2283,2280,2280,2279,2279,2277,2277,2276,2276,2275,2275,2274,2274,2271,2271,2270,2270,2269,2269,2268,2268,2267,2267,2262,2262,2261,2261,2260,2260,2258,2258,2257,2257,2255,2255,2251,2251,2250,2250,2249,2249,2248,2248,2246,2246,2235,2235,2234,2234,2232,2232,2231,2231,2227,2227,2222,2222,2221,2221,2216,2216,2213,2213,2212,2212,2211,2211,2204,2204,2203,2203,2198,2198,2186,2186,2185,2185,2173,2173,2170,2170,2168,2168,2167,2167,2160,2160,2159,2159,2155,2155,2152,2152,2151,2151,2150,2150,2149,2149,2147,2147,2142,2142,2141,2141,2136,2136,2133,2133,2132,2132,2131,2131,2122,2122,2121,2121,2120,2120,2118,2118,2108,2108,2106,2106,2105,2105,2104,2104,2103,2103,2100,2100,2090,2090,2089,2089,2088,2088,2087,2087,2083,2083,2082,2082,2081,2081,2080,2080,2079,2079,2077,2077,2075,2075,2072,2072,2071,2071,2069,2069,2068,2068,2067,2067,2066,2066,2062,2062,2058,2058,2057,2057,2053,2053,2047,2047,2045,2045,2039,2039,2038,2038,2037,2037,2034,2034,2032,2032,2031,2031,2030,2030,2027,2027,2025,2025,2020,2020,2019,2019,2018,2018,2016,2016,2012,2012,2008,2008,2007,2007,2006,2006,2000,2000,1999,1999,1997,1997,1992,1992,1990,1990,1989,1989,1986,1986,1981,1981,1979,1979,1978,1978,1977,1977,1975,1975,1972,1972,1971,1971,1970,1970,1968,1968,1967,1967,1966,1966,1963,1963,1955,1955,1952,1952,1950,1950,1949,1949,1948,1948,1947,1947,1943,1943,1942,1942,1941,1941,1939,1939,1934,1934,1931,1931,1930,1930,1925,1925,1923,1923,1920,1920,1919,1919,1918,1918,1917,1917,1913,1913,1909,1909,1904,1904,1902,1902,1893,1893,1885,1885,1884,1884,1875,1875,1872,1872,1870,1870,1867,1867,1863,1863,1860,1860,1859,1859,1855,1855,1854,1854,1846,1846,1845,1845,1844,1844,1843,1843,1840,1840,1835,1835,1832,1832,1831,1831,1829,1829,1827,1827,1821,1821,1820,1820,1818,1818,1815,1815,1811,1811,1810,1810,1808,1808,1802,1802,1801,1801,1798,1798,1795,1795,1791,1791,1789,1789,1786,1786,1785,1785,1782,1782,1781,1781,1780,1780,1779,1779,1778,1778,1776,1776,1772,1772,1769,1769,1767,1767,1765,1765,1760,1760,1759,1759,1754,1754,1753,1753,1752,1752,1749,1749,1748,1748,1745,1745,1732,1732,1726,1726,1725,1725,1724,1724,1722,1722,1719,1719,1716,1716,1713,1713,1711,1711,1707,1707,1703,1703,1702,1702,1701,1701,1700,1700,1699,1699,1697,1697,1696,1696,1693,1693,1692,1692,1691,1691,1689,1689,1687,1687,1679,1679,1676,1676,1674,1674,1673,1673,1671,1671,1668,1668,1666,1666,1665,1665,1664,1664,1663,1663,1659,1659,1657,1657,1656,1656,1655,1655,1653,1653,1652,1652,1650,1650,1648,1648,1647,1647,1644,1644,1641,1641,1640,1640,1638,1638,1634,1634,1633,1633,1631,1631,1629,1629,1624,1624,1615,1615,1614,1614,1605,1605,1603,1603,1602,1602,1601,1601,1600,1600,1599,1599,1598,1598,1591,1591,1587,1587,1584,1584,1583,1583,1581,1581,1579,1579,1576,1576,1575,1575,1574,1574,1570,1570,1569,1569,1564,1564,1562,1562,1558,1558,1554,1554,1550,1550,1549,1549,1548,1548,1547,1547,1545,1545,1542,1542,1538,1538,1532,1532,1531,1531,1529,1529,1527,1527,1525,1525,1518,1518,1517,1517,1514,1514,1513,1513,1509,1509,1507,1507,1506,1506,1505,1505,1502,1502,1499,1499,1498,1498,1497,1497,1496,1496,1494,1494,1493,1493,1492,1492,1490,1490,1489,1489,1487,1487,1484,1484,1479,1479,1475,1475,1474,1474,1472,1472,1470,1470,1469,1469,1468,1468,1467,1467,1466,1466,1464,1464,1459,1459,1457,1457,1452,1452,1451,1451,1446,1446,1445,1445,1444,1444,1443,1443,1442,1442,1440,1440,1439,1439,1438,1438,1436,1436,1435,1435,1434,1434,1433,1433,1430,1430,1429,1429,1426,1426,1424,1424,1422,1422,1421,1421,1418,1418,1411,1411,1407,1407,1406,1406,1404,1404,1403,1403,1399,1399,1395,1395,1394,1394,1390,1390,1389,1389,1388,1388,1387,1387,1380,1380,1379,1379,1378,1378,1376,1376,1374,1374,1373,1373,1371,1371,1365,1365,1364,1364,1363,1363,1361,1361,1360,1360,1356,1356,1354,1354,1349,1349,1348,1348,1347,1347,1346,1346,1345,1345,1344,1344,1341,1341,1340,1340,1337,1337,1336,1336,1335,1335,1334,1334,1329,1329,1328,1328,1327,1327,1325,1325,1324,1324,1323,1323,1321,1321,1317,1317,1313,1313,1312,1312,1310,1310,1308,1308,1305,1305,1304,1304,1302,1302,1301,1301,1300,1300,1299,1299,1296,1296,1295,1295,1293,1293,1292,1292,1291,1291,1289,1289,1288,1288,1287,1287,1286,1286,1285,1285,1278,1278,1277,1277,1275,1275,1274,1274,1273,1273,1270,1270,1269,1269,1268,1268,1267,1267,1266,1266,1264,1264,1259,1259,1257,1257,1256,1256,1255,1255,1253,1253,1252,1252,1250,1250,1249,1249,1247,1247,1246,1246,1242,1242,1241,1241,1239,1239,1237,1237,1233,1233,1225,1225,1224,1224,1222,1222,1221,1221,1220,1220,1219,1219,1218,1218,1216,1216,1215,1215,1211,1211,1210,1210,1209,1209,1207,1207,1206,1206,1204,1204,1202,1202,1201,1201,1200,1200,1199,1199,1198,1198,1193,1193,1187,1187,1185,1185,1184,1184,1180,1180,1179,1179,1177,1177,1176,1176,1174,1174,1172,1172,1170,1170,1169,1169,1161,1161,1159,1159,1157,1157,1154,1154,1150,1150,1149,1149,1148,1148,1146,1146,1145,1145,1144,1144,1142,1142,1139,1139,1138,1138,1135,1135,1133,1133,1130,1130,1127,1127,1126,1126,1125,1125,1124,1124,1121,1121,1120,1120,1117,1117,1116,1116,1115,1115,1114,1114,1113,1113,1112,1112,1110,1110,1108,1108,1107,1107,1106,1106,1104,1104,1103,1103,1101,1101,1099,1099,1097,1097,1096,1096,1095,1095,1093,1093,1092,1092,1091,1091,1089,1089,1088,1088,1087,1087,1085,1085,1084,1084,1083,1083,1080,1080,1078,1078,1077,1077,1076,1076,1075,1075,1074,1074,1073,1073,1071,1071,1069,1069,1068,1068,1067,1067,1066,1066,1063,1063,1061,1061,1056,1056,1054,1054,1053,1053,1052,1052,1050,1050,1049,1049,1047,1047,1046,1046,1041,1041,1040,1040,1038,1038,1037,1037,1036,1036,1033,1033,1032,1032,1030,1030,1029,1029,1027,1027,1026,1026,1025,1025,1024,1024,1023,1023,1021,1021,1020,1020,1018,1018,1017,1017,1016,1016,1015,1015,1012,1012,1011,1011,1010,1010,1008,1008,1006,1006,1005,1005,1004,1004,1003,1003,1002,1002,1001,1001,1000,1000,998,998,997,997,996,996,995,995,994,994,993,993,992,992,989,989,988,988,986,986,984,984,982,982,981,981,979,979,978,978,977,977,975,975,972,972,971,971,969,969,968,968,967,967,966,966,962,962,960,960,957,957,956,956,955,955,954,954,953,953,952,952,950,950,949,949,948,948,946,946,944,944,943,943,940,940,939,939,938,938,936,936,935,935,934,934,933,933,932,932,931,931,929,929,928,928,926,926,924,924,922,922,921,921,919,919,916,916,915,915,914,914,913,913,912,912,911,911,910,910,909,909,908,908,907,907,904,904,902,902,899,899,898,898,897,897,895,895,893,893,892,892,891,891,889,889,888,888,887,887,884,884,883,883,882,882,881,881,880,880,879,879,877,877,876,876,875,875,874,874,872,872,871,871,870,870,868,868,867,867,866,866,865,865,864,864,863,863,862,862,861,861,860,860,859,859,856,856,855,855,854,854,850,850,849,849,848,848,847,847,846,846,843,843,842,842,840,840,839,839,837,837,834,834,832,832,831,831,829,829,828,828,827,827,826,826,825,825,824,824,823,823,822,822,821,821,820,820,819,819,817,817,816,816,815,815,814,814,812,812,811,811,809,809,808,808,807,807,806,806,805,805,804,804,803,803,802,802,801,801,800,800,799,799,798,798,796,796,794,794,793,793,792,792,790,790,788,788,787,787,786,786,785,785,784,784,781,781,780,780,779,779,778,778,777,777,776,776,774,774,770,770,769,769,768,768,767,767,766,766,765,765,764,764,763,763,762,762,761,761,760,760,759,759,757,757,756,756,755,755,754,754,753,753,752,752,751,751,749,749,748,748,747,747,746,746,744,744,742,742,741,741,740,740,739,739,736,736,734,734,733,733,732,732,731,731,730,730,729,729,728,728,727,727,726,726,725,725,724,724,723,723,722,722,721,721,720,720,718,718,717,717,716,716,715,715,713,713,712,712,710,710,708,708,706,706,705,705,704,704,703,703,702,702,701,701,699,699,698,698,695,695,694,694,693,693,692,692,691,691,690,690,689,689,686,686,685,685,684,684,683,683,681,681,680,680,679,679,678,678,677,677,676,676,675,675,673,673,672,672,671,671,670,670,668,668,667,667,666,666,665,665,663,663,662,662,661,661,660,660,659,659,658,658,655,655,654,654,653,653,652,652,651,651,649,649,647,647,646,646,645,645,644,644,643,643,642,642,641,641,640,640,639,639,638,638,637,637,636,636,634,634,633,633,632,632,631,631,630,630,628,628,627,627,626,626,625,625,624,624,623,623,622,622,621,621,620,620,619,619,618,618,617,617,616,616,615,615,614,614,613,613,612,612,611,611,610,610,609,609,608,608,607,607,606,606,605,605,604,604,603,603,602,602,601,601,600,600,599,599,598,598,597,597,596,596,595,595,594,594,593,593,592,592,591,591,590,590,589,589,588,588,587,587,586,586,585,585,584,584,583,583,582,582,581,581,580,580,579,579,578,578,576,576,575,575,574,574,573,573,572,572,571,571,570,570,569,569,568,568,567,567,566,566,565,565,564,564,563,563,562,562,561,561,560,560,559,559,558,558,557,557,556,556,555,555,554,554,553,553,552,552,551,551,550,550,549,549,548,548,547,547,546,546,545,545,544,544,543,543,542,542,541,541,540,540,539,539,538,538,537,537,536,536,535,535,534,534,533,533,532,532,531,531,530,530,529,529,528,528,527,527,526,526,525,525,524,524,523,523,522,522,521,521,520,520,519,519,518,518,517,517,516,516,515,515,514,514,513,513,512,512,511,511,510,510,509,509,508,508,507,507,506,506,505,505,504,504,503,503,502,502,501,501,500,500,499,499,498,498,497,497,496,496,495,495,494,494,493,493,492,492,491,491,490,490,489,489,488,488,487,487,486,486,485,485,484,484,483,483,482,482,481,481,480,480,479,479,478,478,477,477,476,476,475,475,474,474,473,473,472,472,471,471,470,470,469,469,468,468,467,467,466,466,465,465,464,464,463,463,462,462,461,461,460,460,459,459,458,458,457,457,456,456,455,455,454,454,453,453,452,452,451,451,450,450,449,449,448,448,447,447,446,446,445,445,444,444,443,443,442,442,441,441,440,440,439,439,438,438,437,437,436,436,435,435,434,434,433,433,432,432,431,431,430,430,419,419,410,410,0.0],[157150,157150,157150,157077,157077,102562,102562,91333,91333,88313,88313,87787,87787,85906,85906,83907,83907,79385,79385,79295,79295,78612,78612,76466,76466,73786,73786,72807,72807,71598,71598,71370,71370,69874,69874,69629,69629,69329,69329,68492,68492,68456,68456,68335,68335,67386,67386,67118,67118,66158,66158,65580,65580,65579,65579,64902,64902,63900,63900,63296,63296,62369,62369,62159,62159,61625,61625,61233,61233,61149,61149,60589,60589,60413,60413,59831,59831,59809,59809,58674,58674,58386,58386,58152,58152,57541,57541,56779,56779,55788,55788,55670,55670,54872,54872,54810,54810,54748,54748,54177,54177,53761,53761,53381,53381,52502,52502,52198,52198,52058,52058,51912,51912,51743,51743,51742,51742,50940,50940,50747,50747,50699,50699,50107,50107,49904,49904,49846,49846,49609,49609,49381,49381,49174,49174,49011,49011,47595,47595,47560,47560,47443,47443,47404,47404,47095,47095,46896,46896,46371,46371,46199,46199,45534,45534,45528,45528,45330,45330,45085,45085,45046,45046,44787,44787,44677,44677,44504,44504,44227,44227,44184,44184,44017,44017,44003,44003,43716,43716,42831,42831,42682,42682,42519,42519,42229,42229,41879,41879,41853,41853,41791,41791,41441,41441,41191,41191,41110,41110,40952,40952,40852,40852,40766,40766,40531,40531,40024,40024,39831,39831,39577,39577,39562,39562,39535,39535,39145,39145,39005,39005,38886,38886,38841,38841,38788,38788,38675,38675,38177,38177,38149,38149,38107,38107,37830,37830,37799,37799,37713,37713,37506,37506,37478,37478,37219,37219,37142,37142,36753,36753,36197,36197,36189,36189,36149,36149,35859,35859,35827,35827,35618,35618,35550,35550,35137,35137,35123,35123,35111,35111,34913,34913,34842,34842,34837,34837,34815,34815,34801,34801,34667,34667,34432,34432,34240,34240,34232,34232,34003,34003,33968,33968,33967,33967,33966,33966,33852,33852,33751,33751,33666,33666,33617,33617,33582,33582,33581,33581,33524,33524,33454,33454,33379,33379,33246,33246,33207,33207,32937,32937,32917,32917,32714,32714,32349,32349,32241,32241,32201,32201,32159,32159,32134,32134,32102,32102,32034,32034,31938,31938,31912,31912,31709,31709,31599,31599,31477,31477,31376,31376,31351,31351,31340,31340,31329,31329,31325,31325,31269,31269,30956,30956,30934,30934,30814,30814,30727,30727,30352,30352,30245,30245,30081,30081,30050,30050,29993,29993,29895,29895,29830,29830,29819,29819,29788,29788,29653,29653,29646,29646,29604,29604,29524,29524,29468,29468,29238,29238,29218,29218,29194,29194,29148,29148,28839,28839,28731,28731,28699,28699,28547,28547,28484,28484,28429,28429,28406,28406,28405,28405,28355,28355,28150,28150,27977,27977,27926,27926,27814,27814,27811,27811,27633,27633,27476,27476,27455,27455,27412,27412,27333,27333,27309,27309,27268,27268,27226,27226,26943,26943,26923,26923,26919,26919,26892,26892,26867,26867,26746,26746,26602,26602,26591,26591,26511,26511,26488,26488,26479,26479,26474,26474,26321,26321,26244,26244,26198,26198,26178,26178,26174,26174,26143,26143,26067,26067,25987,25987,25967,25967,25817,25817,25726,25726,25721,25721,25716,25716,25670,25670,25667,25667,25287,25287,25284,25284,25258,25258,25169,25169,25122,25122,24877,24877,24844,24844,24839,24839,24690,24690,24618,24618,24609,24609,24568,24568,24540,24540,24539,24539,24393,24393,24321,24321,24277,24277,24245,24245,24244,24244,24192,24192,24177,24177,24137,24137,24113,24113,24079,24079,23988,23988,23855,23855,23844,23844,23810,23810,23654,23654,23651,23651,23583,23583,23535,23535,23475,23475,23437,23437,23422,23422,23380,23380,23351,23351,23235,23235,23133,23133,23129,23129,23105,23105,23006,23006,22982,22982,22953,22953,22749,22749,22668,22668,22595,22595,22589,22589,22588,22588,22573,22573,22566,22566,22547,22547,22333,22333,22282,22282,22275,22275,22268,22268,22247,22247,22190,22190,22144,22144,22112,22112,22088,22088,22083,22083,22026,22026,22015,22015,21973,21973,21962,21962,21942,21942,21898,21898,21873,21873,21845,21845,21808,21808,21804,21804,21795,21795,21791,21791,21729,21729,21726,21726,21704,21704,21546,21546,21541,21541,21536,21536,21434,21434,21412,21412,21331,21331,21326,21326,21310,21310,21291,21291,21246,21246,21221,21221,21114,21114,21096,21096,21088,21088,21080,21080,21059,21059,21016,21016,20893,20893,20846,20846,20770,20770,20763,20763,20695,20695,20691,20691,20674,20674,20609,20609,20604,20604,20602,20602,20538,20538,20532,20532,20484,20484,20469,20469,20461,20461,20421,20421,20344,20344,20328,20328,20236,20236,20228,20228,20226,20226,20157,20157,20130,20130,20123,20123,20077,20077,20004,20004,20001,20001,19967,19967,19966,19966,19884,19884,19830,19830,19770,19770,19739,19739,19730,19730,19685,19685,19581,19581,19548,19548,19498,19498,19485,19485,19480,19480,19448,19448,19416,19416,19358,19358,19319,19319,19222,19222,19196,19196,19158,19158,19149,19149,19116,19116,19094,19094,19088,19088,19056,19056,19038,19038,19026,19026,19018,19018,18991,18991,18973,18973,18965,18965,18939,18939,18926,18926,18903,18903,18837,18837,18800,18800,18788,18788,18735,18735,18734,18734,18679,18679,18658,18658,18640,18640,18635,18635,18630,18630,18623,18623,18613,18613,18604,18604,18593,18593,18589,18589,18535,18535,18485,18485,18470,18470,18459,18459,18452,18452,18333,18333,18293,18293,18264,18264,18254,18254,18247,18247,18225,18225,18118,18118,18111,18111,18086,18086,18083,18083,18057,18057,18029,18029,17995,17995,17982,17982,17970,17970,17961,17961,17947,17947,17904,17904,17857,17857,17852,17852,17824,17824,17809,17809,17777,17777,17701,17701,17695,17695,17694,17694,17685,17685,17660,17660,17659,17659,17639,17639,17548,17548,17542,17542,17512,17512,17504,17504,17487,17487,17474,17474,17464,17464,17431,17431,17392,17392,17256,17256,17242,17242,17216,17216,17214,17214,17147,17147,17115,17115,17113,17113,17102,17102,17096,17096,17084,17084,17036,17036,16998,16998,16993,16993,16987,16987,16982,16982,16960,16960,16953,16953,16859,16859,16804,16804,16782,16782,16777,16777,16745,16745,16732,16732,16726,16726,16706,16706,16665,16665,16607,16607,16605,16605,16548,16548,16547,16547,16539,16539,16503,16503,16456,16456,16424,16424,16383,16383,16377,16377,16368,16368,16356,16356,16336,16336,16324,16324,16313,16313,16298,16298,16271,16271,16265,16265,16230,16230,16216,16216,16179,16179,16170,16170,16136,16136,16134,16134,16106,16106,16085,16085,16075,16075,16066,16066,16065,16065,16008,16008,16006,16006,15981,15981,15965,15965,15961,15961,15957,15957,15915,15915,15913,15913,15870,15870,15857,15857,15848,15848,15833,15833,15817,15817,15803,15803,15798,15798,15785,15785,15764,15764,15761,15761,15730,15730,15634,15634,15633,15633,15628,15628,15618,15618,15605,15605,15552,15552,15541,15541,15502,15502,15486,15486,15460,15460,15456,15456,15431,15431,15412,15412,15409,15409,15378,15378,15327,15327,15320,15320,15307,15307,15279,15279,15273,15273,15255,15255,15204,15204,15193,15193,15180,15180,15177,15177,15176,15176,15165,15165,15138,15138,15113,15113,15101,15101,15087,15087,15058,15058,15034,15034,15020,15020,14987,14987,14980,14980,14972,14972,14969,14969,14967,14967,14940,14940,14938,14938,14924,14924,14886,14886,14879,14879,14836,14836,14823,14823,14761,14761,14726,14726,14720,14720,14695,14695,14629,14629,14625,14625,14610,14610,14601,14601,14593,14593,14544,14544,14531,14531,14530,14530,14523,14523,14510,14510,14507,14507,14503,14503,14484,14484,14452,14452,14446,14446,14430,14430,14419,14419,14368,14368,14362,14362,14340,14340,14305,14305,14263,14263,14248,14248,14234,14234,14219,14219,14211,14211,14210,14210,14150,14150,14131,14131,14122,14122,14072,14072,14065,14065,14054,14054,14045,14045,14044,14044,14032,14032,14011,14011,13996,13996,13992,13992,13978,13978,13956,13956,13871,13871,13864,13864,13862,13862,13850,13850,13795,13795,13790,13790,13764,13764,13756,13756,13750,13750,13717,13717,13688,13688,13680,13680,13646,13646,13642,13642,13641,13641,13638,13638,13635,13635,13602,13602,13598,13598,13581,13581,13542,13542,13510,13510,13456,13456,13445,13445,13438,13438,13430,13430,13422,13422,13417,13417,13390,13390,13388,13388,13368,13368,13362,13362,13333,13333,13316,13316,13312,13312,13294,13294,13291,13291,13286,13286,13284,13284,13283,13283,13260,13260,13212,13212,13204,13204,13179,13179,13160,13160,13156,13156,13132,13132,13130,13130,13117,13117,13114,13114,13104,13104,13086,13086,13084,13084,13077,13077,13076,13076,13066,13066,13057,13057,13055,13055,13052,13052,12999,12999,12981,12981,12947,12947,12936,12936,12935,12935,12931,12931,12928,12928,12923,12923,12901,12901,12838,12838,12818,12818,12809,12809,12802,12802,12801,12801,12796,12796,12790,12790,12781,12781,12773,12773,12758,12758,12692,12692,12688,12688,12685,12685,12661,12661,12645,12645,12636,12636,12625,12625,12599,12599,12591,12591,12569,12569,12558,12558,12540,12540,12521,12521,12497,12497,12490,12490,12486,12486,12476,12476,12463,12463,12450,12450,12442,12442,12438,12438,12434,12434,12420,12420,12419,12419,12400,12400,12386,12386,12364,12364,12358,12358,12350,12350,12336,12336,12335,12335,12305,12305,12273,12273,12269,12269,12247,12247,12243,12243,12229,12229,12225,12225,12219,12219,12196,12196,12177,12177,12162,12162,12158,12158,12146,12146,12138,12138,12135,12135,12134,12134,12124,12124,12123,12123,12115,12115,12098,12098,12076,12076,12064,12064,12054,12054,12043,12043,12023,12023,11999,11999,11986,11986,11983,11983,11975,11975,11971,11971,11966,11966,11961,11961,11951,11951,11932,11932,11923,11923,11908,11908,11894,11894,11888,11888,11874,11874,11857,11857,11827,11827,11809,11809,11790,11790,11771,11771,11770,11770,11747,11747,11734,11734,11723,11723,11719,11719,11654,11654,11647,11647,11616,11616,11612,11612,11595,11595,11590,11590,11576,11576,11554,11554,11552,11552,11527,11527,11526,11526,11482,11482,11479,11479,11473,11473,11460,11460,11456,11456,11449,11449,11448,11448,11443,11443,11426,11426,11425,11425,11412,11412,11391,11391,11390,11390,11376,11376,11357,11357,11356,11356,11342,11342,11329,11329,11325,11325,11316,11316,11313,11313,11307,11307,11302,11302,11288,11288,11257,11257,11249,11249,11244,11244,11234,11234,11225,11225,11217,11217,11207,11207,11206,11206,11201,11201,11164,11164,11160,11160,11144,11144,11099,11099,11095,11095,11082,11082,11079,11079,11077,11077,11067,11067,11066,11066,11057,11057,11051,11051,11048,11048,11040,11040,11031,11031,11011,11011,11009,11009,11008,11008,11001,11001,10986,10986,10980,10980,10977,10977,10947,10947,10938,10938,10929,10929,10928,10928,10910,10910,10894,10894,10892,10892,10879,10879,10870,10870,10866,10866,10863,10863,10860,10860,10859,10859,10858,10858,10843,10843,10830,10830,10816,10816,10810,10810,10808,10808,10795,10795,10781,10781,10766,10766,10709,10709,10695,10695,10687,10687,10666,10666,10661,10661,10625,10625,10617,10617,10615,10615,10613,10613,10599,10599,10589,10589,10584,10584,10577,10577,10561,10561,10536,10536,10534,10534,10520,10520,10512,10512,10504,10504,10498,10498,10484,10484,10472,10472,10460,10460,10453,10453,10445,10445,10430,10430,10419,10419,10415,10415,10414,10414,10402,10402,10395,10395,10390,10390,10377,10377,10367,10367,10352,10352,10350,10350,10345,10345,10326,10326,10323,10323,10317,10317,10314,10314,10311,10311,10285,10285,10277,10277,10272,10272,10263,10263,10262,10262,10257,10257,10255,10255,10252,10252,10250,10250,10249,10249,10240,10240,10239,10239,10236,10236,10232,10232,10196,10196,10195,10195,10184,10184,10182,10182,10164,10164,10158,10158,10147,10147,10121,10121,10116,10116,10115,10115,10114,10114,10112,10112,10107,10107,10105,10105,10102,10102,10100,10100,10082,10082,10077,10077,10075,10075,10073,10073,10071,10071,10050,10050,10046,10046,10028,10028,9994,9994,9974,9974,9971,9971,9961,9961,9955,9955,9948,9948,9938,9938,9929,9929,9917,9917,9915,9915,9907,9907,9898,9898,9875,9875,9847,9847,9845,9845,9840,9840,9836,9836,9824,9824,9814,9814,9803,9803,9802,9802,9799,9799,9797,9797,9793,9793,9775,9775,9768,9768,9765,9765,9764,9764,9719,9719,9707,9707,9684,9684,9681,9681,9679,9679,9671,9671,9617,9617,9613,9613,9589,9589,9581,9581,9575,9575,9553,9553,9550,9550,9547,9547,9519,9519,9513,9513,9495,9495,9489,9489,9488,9488,9487,9487,9485,9485,9482,9482,9479,9479,9471,9471,9458,9458,9457,9457,9455,9455,9453,9453,9449,9449,9445,9445,9440,9440,9435,9435,9415,9415,9401,9401,9392,9392,9378,9378,9374,9374,9365,9365,9364,9364,9362,9362,9361,9361,9353,9353,9351,9351,9350,9350,9348,9348,9332,9332,9323,9323,9308,9308,9298,9298,9274,9274,9263,9263,9240,9240,9232,9232,9231,9231,9230,9230,9222,9222,9217,9217,9197,9197,9189,9189,9178,9178,9125,9125,9122,9122,9094,9094,9087,9087,9081,9081,9073,9073,9067,9067,9059,9059,9053,9053,9047,9047,9046,9046,9034,9034,9005,9005,8994,8994,8990,8990,8979,8979,8976,8976,8974,8974,8955,8955,8948,8948,8929,8929,8905,8905,8902,8902,8888,8888,8867,8867,8853,8853,8842,8842,8833,8833,8831,8831,8821,8821,8817,8817,8807,8807,8799,8799,8798,8798,8794,8794,8789,8789,8781,8781,8780,8780,8769,8769,8746,8746,8743,8743,8729,8729,8725,8725,8719,8719,8718,8718,8709,8709,8702,8702,8700,8700,8697,8697,8689,8689,8685,8685,8671,8671,8636,8636,8631,8631,8629,8629,8615,8615,8613,8613,8609,8609,8608,8608,8602,8602,8601,8601,8596,8596,8592,8592,8588,8588,8586,8586,8545,8545,8537,8537,8531,8531,8525,8525,8524,8524,8520,8520,8514,8514,8510,8510,8498,8498,8490,8490,8488,8488,8484,8484,8478,8478,8477,8477,8475,8475,8467,8467,8465,8465,8451,8451,8443,8443,8441,8441,8428,8428,8412,8412,8411,8411,8407,8407,8405,8405,8399,8399,8395,8395,8386,8386,8384,8384,8374,8374,8366,8366,8364,8364,8353,8353,8352,8352,8343,8343,8342,8342,8340,8340,8337,8337,8334,8334,8318,8318,8310,8310,8308,8308,8294,8294,8292,8292,8290,8290,8276,8276,8275,8275,8274,8274,8263,8263,8257,8257,8245,8245,8237,8237,8231,8231,8219,8219,8217,8217,8209,8209,8205,8205,8204,8204,8191,8191,8178,8178,8175,8175,8171,8171,8166,8166,8159,8159,8155,8155,8151,8151,8142,8142,8140,8140,8138,8138,8131,8131,8127,8127,8108,8108,8106,8106,8057,8057,8054,8054,8036,8036,8029,8029,8026,8026,8023,8023,8014,8014,8008,8008,8004,8004,7997,7997,7986,7986,7984,7984,7961,7961,7944,7944,7932,7932,7929,7929,7914,7914,7911,7911,7905,7905,7895,7895,7878,7878,7875,7875,7861,7861,7853,7853,7837,7837,7833,7833,7826,7826,7818,7818,7811,7811,7807,7807,7802,7802,7800,7800,7770,7770,7752,7752,7750,7750,7749,7749,7748,7748,7737,7737,7730,7730,7699,7699,7696,7696,7695,7695,7686,7686,7663,7663,7658,7658,7656,7656,7646,7646,7635,7635,7626,7626,7623,7623,7616,7616,7606,7606,7604,7604,7600,7600,7594,7594,7583,7583,7579,7579,7571,7571,7565,7565,7561,7561,7557,7557,7539,7539,7534,7534,7532,7532,7530,7530,7529,7529,7525,7525,7522,7522,7517,7517,7514,7514,7509,7509,7495,7495,7485,7485,7478,7478,7477,7477,7470,7470,7466,7466,7459,7459,7454,7454,7450,7450,7448,7448,7444,7444,7443,7443,7437,7437,7436,7436,7432,7432,7430,7430,7417,7417,7412,7412,7411,7411,7401,7401,7400,7400,7382,7382,7361,7361,7353,7353,7339,7339,7334,7334,7322,7322,7317,7317,7315,7315,7313,7313,7310,7310,7299,7299,7294,7294,7292,7292,7232,7232,7226,7226,7219,7219,7217,7217,7216,7216,7213,7213,7208,7208,7204,7204,7201,7201,7196,7196,7190,7190,7189,7189,7183,7183,7176,7176,7134,7134,7128,7128,7111,7111,7108,7108,7107,7107,7104,7104,7057,7057,7035,7035,7027,7027,7026,7026,7016,7016,7009,7009,6994,6994,6993,6993,6988,6988,6982,6982,6981,6981,6976,6976,6968,6968,6966,6966,6963,6963,6941,6941,6938,6938,6927,6927,6920,6920,6910,6910,6889,6889,6882,6882,6872,6872,6851,6851,6850,6850,6838,6838,6832,6832,6828,6828,6826,6826,6823,6823,6811,6811,6798,6798,6797,6797,6792,6792,6781,6781,6778,6778,6775,6775,6768,6768,6748,6748,6731,6731,6727,6727,6725,6725,6715,6715,6691,6691,6689,6689,6671,6671,6664,6664,6663,6663,6662,6662,6656,6656,6652,6652,6640,6640,6632,6632,6629,6629,6620,6620,6618,6618,6611,6611,6609,6609,6601,6601,6596,6596,6543,6543,6539,6539,6529,6529,6521,6521,6518,6518,6493,6493,6476,6476,6465,6465,6429,6429,6415,6415,6386,6386,6385,6385,6378,6378,6368,6368,6362,6362,6360,6360,6358,6358,6339,6339,6331,6331,6323,6323,6321,6321,6310,6310,6306,6306,6304,6304,6294,6294,6276,6276,6275,6275,6268,6268,6265,6265,6262,6262,6261,6261,6256,6256,6255,6255,6251,6251,6247,6247,6242,6242,6235,6235,6234,6234,6221,6221,6211,6211,6204,6204,6189,6189,6186,6186,6184,6184,6175,6175,6174,6174,6166,6166,6150,6150,6134,6134,6101,6101,6097,6097,6087,6087,6082,6082,6061,6061,6058,6058,6049,6049,6043,6043,6034,6034,6028,6028,6015,6015,6008,6008,6003,6003,5999,5999,5976,5976,5971,5971,5968,5968,5944,5944,5943,5943,5938,5938,5915,5915,5914,5914,5913,5913,5909,5909,5908,5908,5897,5897,5896,5896,5880,5880,5877,5877,5873,5873,5867,5867,5846,5846,5840,5840,5837,5837,5834,5834,5827,5827,5809,5809,5784,5784,5779,5779,5778,5778,5767,5767,5766,5766,5754,5754,5746,5746,5740,5740,5738,5738,5714,5714,5710,5710,5708,5708,5699,5699,5688,5688,5687,5687,5681,5681,5671,5671,5667,5667,5666,5666,5655,5655,5654,5654,5653,5653,5648,5648,5621,5621,5600,5600,5599,5599,5593,5593,5572,5572,5564,5564,5563,5563,5554,5554,5549,5549,5540,5540,5532,5532,5523,5523,5518,5518,5483,5483,5478,5478,5464,5464,5461,5461,5453,5453,5434,5434,5420,5420,5405,5405,5399,5399,5379,5379,5372,5372,5366,5366,5361,5361,5358,5358,5348,5348,5340,5340,5330,5330,5315,5315,5311,5311,5309,5309,5305,5305,5300,5300,5299,5299,5293,5293,5291,5291,5287,5287,5281,5281,5271,5271,5265,5265,5260,5260,5254,5254,5248,5248,5237,5237,5229,5229,5222,5222,5214,5214,5204,5204,5200,5200,5199,5199,5193,5193,5181,5181,5178,5178,5173,5173,5171,5171,5156,5156,5146,5146,5144,5144,5142,5142,5139,5139,5137,5137,5134,5134,5132,5132,5126,5126,5122,5122,5120,5120,5118,5118,5111,5111,5107,5107,5106,5106,5103,5103,5099,5099,5086,5086,5057,5057,5048,5048,5036,5036,5013,5013,5000,5000,4993,4993,4982,4982,4977,4977,4970,4970,4968,4968,4959,4959,4957,4957,4953,4953,4952,4952,4951,4951,4949,4949,4933,4933,4924,4924,4923,4923,4917,4917,4915,4915,4908,4908,4905,4905,4889,4889,4884,4884,4878,4878,4874,4874,4871,4871,4850,4850,4849,4849,4848,4848,4844,4844,4843,4843,4841,4841,4836,4836,4830,4830,4825,4825,4817,4817,4813,4813,4804,4804,4800,4800,4798,4798,4793,4793,4784,4784,4778,4778,4777,4777,4776,4776,4775,4775,4765,4765,4761,4761,4756,4756,4751,4751,4747,4747,4727,4727,4722,4722,4721,4721,4716,4716,4713,4713,4700,4700,4680,4680,4676,4676,4673,4673,4670,4670,4669,4669,4668,4668,4655,4655,4644,4644,4640,4640,4615,4615,4612,4612,4609,4609,4594,4594,4582,4582,4580,4580,4575,4575,4563,4563,4562,4562,4558,4558,4555,4555,4549,4549,4530,4530,4516,4516,4514,4514,4513,4513,4505,4505,4504,4504,4486,4486,4485,4485,4471,4471,4459,4459,4441,4441,4432,4432,4421,4421,4419,4419,4402,4402,4400,4400,4382,4382,4381,4381,4375,4375,4374,4374,4372,4372,4353,4353,4340,4340,4339,4339,4333,4333,4330,4330,4329,4329,4305,4305,4301,4301,4299,4299,4296,4296,4290,4290,4284,4284,4278,4278,4274,4274,4272,4272,4270,4270,4264,4264,4258,4258,4256,4256,4255,4255,4251,4251,4247,4247,4241,4241,4222,4222,4219,4219,4210,4210,4205,4205,4200,4200,4198,4198,4186,4186,4183,4183,4165,4165,4164,4164,4161,4161,4159,4159,4157,4157,4143,4143,4132,4132,4117,4117,4111,4111,4091,4091,4074,4074,4068,4068,4064,4064,4056,4056,4052,4052,4049,4049,4043,4043,4033,4033,4028,4028,4026,4026,4024,4024,4015,4015,4014,4014,4009,4009,4004,4004,3991,3991,3988,3988,3983,3983,3977,3977,3949,3949,3946,3946,3940,3940,3931,3931,3908,3908,3907,3907,3904,3904,3903,3903,3899,3899,3892,3892,3873,3873,3865,3865,3856,3856,3853,3853,3848,3848,3835,3835,3828,3828,3822,3822,3821,3821,3815,3815,3809,3809,3806,3806,3799,3799,3794,3794,3786,3786,3782,3782,3774,3774,3762,3762,3760,3760,3757,3757,3753,3753,3744,3744,3740,3740,3737,3737,3736,3736,3726,3726,3718,3718,3702,3702,3700,3700,3698,3698,3694,3694,3690,3690,3683,3683,3674,3674,3673,3673,3670,3670,3669,3669,3664,3664,3663,3663,3662,3662,3657,3657,3654,3654,3653,3653,3640,3640,3635,3635,3628,3628,3624,3624,3622,3622,3608,3608,3606,3606,3604,3604,3592,3592,3591,3591,3588,3588,3580,3580,3579,3579,3573,3573,3570,3570,3566,3566,3562,3562,3559,3559,3558,3558,3554,3554,3547,3547,3538,3538,3525,3525,3522,3522,3516,3516,3512,3512,3511,3511,3510,3510,3505,3505,3504,3504,3495,3495,3490,3490,3489,3489,3468,3468,3466,3466,3452,3452,3449,3449,3446,3446,3441,3441,3438,3438,3436,3436,3435,3435,3417,3417,3402,3402,3400,3400,3398,3398,3393,3393,3386,3386,3379,3379,3377,3377,3372,3372,3370,3370,3366,3366,3362,3362,3356,3356,3354,3354,3347,3347,3346,3346,3337,3337,3336,3336,3311,3311,3303,3303,3298,3298,3293,3293,3287,3287,3272,3272,3264,3264,3254,3254,3253,3253,3252,3252,3250,3250,3248,3248,3243,3243,3241,3241,3232,3232,3210,3210,3208,3208,3204,3204,3202,3202,3201,3201,3197,3197,3194,3194,3193,3193,3182,3182,3173,3173,3171,3171,3170,3170,3168,3168,3167,3167,3159,3159,3155,3155,3154,3154,3138,3138,3133,3133,3132,3132,3126,3126,3125,3125,3120,3120,3116,3116,3114,3114,3104,3104,3101,3101,3098,3098,3093,3093,3090,3090,3089,3089,3088,3088,3084,3084,3061,3061,3058,3058,3057,3057,3056,3056,3051,3051,3040,3040,3036,3036,3035,3035,3033,3033,3031,3031,3030,3030,3024,3024,3023,3023,3022,3022,3021,3021,3020,3020,3019,3019,3008,3008,3005,3005,2988,2988,2986,2986,2984,2984,2982,2982,2962,2962,2952,2952,2950,2950,2942,2942,2939,2939,2936,2936,2935,2935,2933,2933,2921,2921,2920,2920,2913,2913,2912,2912,2895,2895,2893,2893,2890,2890,2889,2889,2888,2888,2887,2887,2876,2876,2874,2874,2871,2871,2863,2863,2861,2861,2859,2859,2858,2858,2856,2856,2854,2854,2851,2851,2847,2847,2844,2844,2840,2840,2834,2834,2833,2833,2831,2831,2826,2826,2825,2825,2823,2823,2817,2817,2808,2808,2806,2806,2804,2804,2799,2799,2792,2792,2791,2791,2787,2787,2783,2783,2782,2782,2769,2769,2767,2767,2765,2765,2764,2764,2763,2763,2759,2759,2732,2732,2729,2729,2725,2725,2720,2720,2711,2711,2709,2709,2708,2708,2707,2707,2704,2704,2701,2701,2699,2699,2689,2689,2686,2686,2685,2685,2679,2679,2675,2675,2673,2673,2668,2668,2663,2663,2657,2657,2655,2655,2646,2646,2642,2642,2639,2639,2633,2633,2632,2632,2628,2628,2621,2621,2613,2613,2610,2610,2606,2606,2603,2603,2597,2597,2587,2587,2562,2562,2554,2554,2552,2552,2550,2550,2549,2549,2548,2548,2545,2545,2541,2541,2539,2539,2522,2522,2519,2519,2517,2517,2514,2514,2513,2513,2512,2512,2503,2503,2502,2502,2499,2499,2498,2498,2494,2494,2485,2485,2481,2481,2474,2474,2473,2473,2461,2461,2449,2449,2447,2447,2446,2446,2443,2443,2442,2442,2437,2437,2435,2435,2430,2430,2429,2429,2418,2418,2404,2404,2403,2403,2399,2399,2396,2396,2393,2393,2382,2382,2380,2380,2376,2376,2371,2371,2369,2369,2366,2366,2363,2363,2362,2362,2359,2359,2357,2357,2355,2355,2344,2344,2342,2342,2340,2340,2335,2335,2334,2334,2333,2333,2331,2331,2326,2326,2324,2324,2321,2321,2320,2320,2317,2317,2316,2316,2309,2309,2305,2305,2297,2297,2295,2295,2292,2292,2291,2291,2286,2286,2283,2283,2280,2280,2279,2279,2277,2277,2274,2274,2271,2271,2270,2270,2269,2269,2258,2258,2255,2255,2250,2250,2249,2249,2246,2246,2240,2240,2235,2235,2233,2233,2231,2231,2227,2227,2224,2224,2222,2222,2221,2221,2216,2216,2213,2213,2181,2181,2177,2177,2171,2171,2170,2170,2160,2160,2152,2152,2151,2151,2150,2150,2147,2147,2142,2142,2141,2141,2121,2121,2120,2120,2110,2110,2106,2106,2104,2104,2103,2103,2100,2100,2098,2098,2090,2090,2089,2089,2087,2087,2082,2082,2080,2080,2077,2077,2073,2073,2068,2068,2067,2067,2065,2065,2064,2064,2062,2062,2058,2058,2057,2057,2046,2046,2044,2044,2039,2039,2025,2025,2024,2024,2023,2023,2020,2020,2016,2016,2012,2012,2011,2011,2005,2005,2004,2004,2003,2003,1999,1999,1990,1990,1989,1989,1986,1986,1984,1984,1981,1981,1977,1977,1975,1975,1972,1972,1971,1971,1970,1970,1968,1968,1967,1967,1961,1961,1955,1955,1954,1954,1950,1950,1946,1946,1945,1945,1943,1943,1942,1942,1941,1941,1939,1939,1931,1931,1930,1930,1923,1923,1922,1922,1919,1919,1918,1918,1917,1917,1912,1912,1910,1910,1899,1899,1894,1894,1890,1890,1887,1887,1885,1885,1878,1878,1875,1875,1874,1874,1867,1867,1863,1863,1862,1862,1860,1860,1859,1859,1858,1858,1846,1846,1844,1844,1840,1840,1826,1826,1823,1823,1822,1822,1820,1820,1815,1815,1807,1807,1802,1802,1801,1801,1798,1798,1795,1795,1793,1793,1791,1791,1783,1783,1782,1782,1780,1780,1778,1778,1769,1769,1765,1765,1764,1764,1763,1763,1759,1759,1758,1758,1753,1753,1749,1749,1745,1745,1743,1743,1732,1732,1728,1728,1726,1726,1725,1725,1722,1722,1717,1717,1716,1716,1709,1709,1707,1707,1703,1703,1702,1702,1695,1695,1694,1694,1693,1693,1689,1689,1688,1688,1687,1687,1686,1686,1679,1679,1676,1676,1674,1674,1672,1672,1670,1670,1668,1668,1666,1666,1665,1665,1664,1664,1657,1657,1653,1653,1652,1652,1650,1650,1646,1646,1636,1636,1635,1635,1634,1634,1632,1632,1631,1631,1627,1627,1625,1625,1611,1611,1608,1608,1602,1602,1600,1600,1599,1599,1595,1595,1592,1592,1591,1591,1587,1587,1586,1586,1583,1583,1581,1581,1575,1575,1574,1574,1569,1569,1558,1558,1555,1555,1554,1554,1550,1550,1546,1546,1542,1542,1540,1540,1536,1536,1529,1529,1527,1527,1523,1523,1522,1522,1518,1518,1517,1517,1515,1515,1514,1514,1512,1512,1509,1509,1505,1505,1499,1499,1497,1497,1496,1496,1494,1494,1493,1493,1490,1490,1489,1489,1485,1485,1484,1484,1481,1481,1477,1477,1472,1472,1470,1470,1469,1469,1467,1467,1466,1466,1465,1465,1462,1462,1458,1458,1457,1457,1456,1456,1454,1454,1452,1452,1446,1446,1443,1443,1442,1442,1439,1439,1438,1438,1437,1437,1436,1436,1435,1435,1434,1434,1430,1430,1426,1426,1425,1425,1421,1421,1416,1416,1414,1414,1408,1408,1406,1406,1405,1405,1404,1404,1403,1403,1399,1399,1395,1395,1390,1390,1389,1389,1387,1387,1384,1384,1383,1383,1381,1381,1376,1376,1373,1373,1372,1372,1371,1371,1365,1365,1362,1362,1361,1361,1360,1360,1357,1357,1356,1356,1353,1353,1352,1352,1349,1349,1347,1347,1344,1344,1339,1339,1337,1337,1336,1336,1335,1335,1334,1334,1333,1333,1331,1331,1330,1330,1329,1329,1327,1327,1324,1324,1323,1323,1321,1321,1318,1318,1316,1316,1315,1315,1314,1314,1313,1313,1311,1311,1309,1309,1306,1306,1305,1305,1304,1304,1303,1303,1302,1302,1301,1301,1300,1300,1296,1296,1294,1294,1293,1293,1292,1292,1291,1291,1287,1287,1286,1286,1282,1282,1277,1277,1275,1275,1271,1271,1269,1269,1268,1268,1264,1264,1263,1263,1261,1261,1257,1257,1255,1255,1253,1253,1252,1252,1249,1249,1247,1247,1246,1246,1243,1243,1242,1242,1241,1241,1237,1237,1234,1234,1233,1233,1232,1232,1229,1229,1228,1228,1227,1227,1225,1225,1224,1224,1223,1223,1222,1222,1221,1221,1220,1220,1219,1219,1218,1218,1216,1216,1215,1215,1214,1214,1211,1211,1210,1210,1209,1209,1207,1207,1206,1206,1201,1201,1200,1200,1198,1198,1196,1196,1194,1194,1193,1193,1190,1190,1183,1183,1180,1180,1179,1179,1177,1177,1175,1175,1174,1174,1172,1172,1171,1171,1168,1168,1166,1166,1165,1165,1164,1164,1161,1161,1160,1160,1159,1159,1158,1158,1157,1157,1156,1156,1155,1155,1154,1154,1150,1150,1148,1148,1144,1144,1142,1142,1137,1137,1136,1136,1135,1135,1132,1132,1131,1131,1128,1128,1127,1127,1126,1126,1124,1124,1123,1123,1122,1122,1121,1121,1120,1120,1119,1119,1116,1116,1114,1114,1113,1113,1110,1110,1107,1107,1105,1105,1104,1104,1101,1101,1099,1099,1097,1097,1096,1096,1093,1093,1092,1092,1091,1091,1090,1090,1088,1088,1087,1087,1086,1086,1085,1085,1082,1082,1080,1080,1075,1075,1074,1074,1073,1073,1071,1071,1069,1069,1068,1068,1067,1067,1066,1066,1065,1065,1063,1063,1062,1062,1061,1061,1055,1055,1054,1054,1053,1053,1052,1052,1051,1051,1044,1044,1043,1043,1042,1042,1041,1041,1040,1040,1038,1038,1036,1036,1033,1033,1030,1030,1027,1027,1026,1026,1025,1025,1024,1024,1016,1016,1012,1012,1011,1011,1010,1010,1009,1009,1005,1005,1003,1003,1002,1002,1001,1001,999,999,998,998,997,997,991,991,990,990,988,988,987,987,982,982,981,981,979,979,978,978,977,977,975,975,973,973,972,972,971,971,970,970,968,968,967,967,965,965,964,964,963,963,962,962,959,959,958,958,957,957,954,954,952,952,951,951,950,950,949,949,946,946,945,945,943,943,941,941,940,940,939,939,938,938,936,936,935,935,934,934,932,932,930,930,929,929,927,927,926,926,922,922,921,921,920,920,919,919,917,917,915,915,914,914,913,913,912,912,910,910,908,908,907,907,905,905,904,904,902,902,897,897,896,896,895,895,892,892,891,891,890,890,889,889,887,887,884,884,883,883,880,880,879,879,878,878,877,877,875,875,872,872,871,871,868,868,866,866,865,865,863,863,862,862,860,860,859,859,858,858,857,857,856,856,854,854,853,853,852,852,851,851,850,850,849,849,848,848,847,847,844,844,842,842,840,840,839,839,837,837,836,836,835,835,834,834,833,833,832,832,831,831,830,830,829,829,828,828,825,825,824,824,822,822,821,821,820,820,819,819,817,817,816,816,814,814,811,811,809,809,807,807,806,806,805,805,804,804,803,803,802,802,801,801,800,800,798,798,796,796,795,795,794,794,793,793,790,790,787,787,786,786,785,785,784,784,783,783,782,782,781,781,780,780,778,778,777,777,776,776,775,775,774,774,773,773,771,771,770,770,769,769,768,768,767,767,766,766,764,764,762,762,761,761,759,759,756,756,755,755,754,754,753,753,751,751,749,749,748,748,746,746,745,745,742,742,741,741,740,740,739,739,736,736,734,734,733,733,732,732,731,731,730,730,728,728,726,726,725,725,723,723,722,722,721,721,720,720,719,719,718,718,717,717,716,716,714,714,713,713,711,711,708,708,706,706,705,705,704,704,703,703,702,702,700,700,699,699,698,698,697,697,691,691,689,689,688,688,687,687,686,686,685,685,684,684,683,683,681,681,680,680,679,679,677,677,676,676,673,673,672,672,671,671,670,670,668,668,667,667,666,666,664,664,663,663,661,661,660,660,659,659,658,658,657,657,655,655,653,653,652,652,651,651,650,650,649,649,647,647,646,646,645,645,644,644,643,643,642,642,641,641,640,640,639,639,638,638,637,637,636,636,635,635,634,634,633,633,632,632,631,631,630,630,629,629,627,627,626,626,625,625,624,624,623,623,621,621,619,619,618,618,616,616,614,614,613,613,612,612,610,610,609,609,608,608,607,607,606,606,604,604,603,603,602,602,601,601,600,600,598,598,597,597,596,596,595,595,594,594,593,593,592,592,591,591,590,590,589,589,588,588,587,587,585,585,584,584,583,583,582,582,581,581,580,580,579,579,578,578,577,577,576,576,575,575,574,574,573,573,572,572,571,571,570,570,569,569,568,568,567,567,566,566,565,565,564,564,563,563,562,562,561,561,560,560,559,559,558,558,557,557,556,556,555,555,554,554,553,553,552,552,551,551,550,550,549,549,548,548,547,547,546,546,545,545,544,544,542,542,541,541,540,540,539,539,538,538,537,537,536,536,535,535,534,534,533,533,532,532,531,531,530,530,529,529,528,528,527,527,526,526,525,525,524,524,523,523,522,522,521,521,520,520,519,519,518,518,517,517,516,516,515,515,514,514,513,513,512,512,511,511,510,510,509,509,508,508,507,507,506,506,505,505,504,504,503,503,502,502,501,501,500,500,499,499,498,498,497,497,496,496,495,495,494,494,493,493,491,491,490,490,489,489,488,488,487,487,486,486,485,485,484,484,483,483,482,482,481,481,480,480,479,479,478,478,477,477,476,476,475,475,474,474,473,473,472,472,471,471,470,470,469,469,468,468,467,467,466,466,465,465,464,464,463,463,462,462,461,461,460,460,459,459,458,458,457,457,456,456,455,455,454,454,453,453,452,452,451,451,450,450,449,449,448,448,447,447,446,446,445,445,444,444,443,443,442,442,441,441,440,440,439,439,438,438,437,437,436,436,435,435,434,434,433,433,432,432,431,431,430,430,0.0],[85407,85407,85407,82898,82898,67830,67830,64715,64715,62382,62382,57426,57426,55669,55669,55648,55648,54736,54736,54279,54279,54161,54161,53673,53673,52923,52923,49062,49062,48901,48901,48671,48671,47264,47264,47074,47074,46869,46869,46446,46446,46133,46133,45258,45258,44854,44854,44815,44815,44148,44148,41164,41164,40974,40974,40868,40868,40800,40800,40369,40369,40246,40246,40190,40190,40162,40162,40147,40147,39357,39357,39344,39344,39036,39036,38966,38966,38765,38765,38416,38416,38379,38379,37476,37476,37475,37475,37179,37179,36970,36970,36283,36283,35865,35865,35380,35380,34775,34775,34773,34773,34517,34517,33742,33742,33676,33676,33517,33517,33435,33435,33166,33166,33006,33006,32992,32992,32961,32961,32959,32959,32877,32877,32843,32843,32511,32511,32493,32493,32253,32253,32159,32159,31959,31959,31499,31499,31316,31316,31308,31308,31290,31290,31137,31137,31072,31072,30939,30939,30861,30861,30824,30824,30725,30725,30644,30644,30489,30489,30334,30334,30301,30301,30097,30097,30014,30014,29782,29782,29662,29662,29422,29422,29232,29232,29161,29161,28947,28947,28840,28840,28428,28428,28244,28244,28241,28241,28156,28156,28149,28149,28006,28006,27770,27770,27746,27746,27599,27599,27352,27352,27345,27345,27280,27280,26998,26998,26972,26972,26930,26930,26770,26770,26723,26723,26718,26718,26561,26561,26552,26552,26526,26526,26322,26322,26005,26005,25901,25901,25882,25882,25876,25876,25867,25867,25843,25843,25707,25707,25582,25582,25391,25391,25360,25360,25289,25289,25128,25128,25125,25125,25079,25079,24987,24987,24976,24976,24856,24856,24839,24839,24825,24825,24685,24685,24643,24643,24519,24519,24350,24350,24328,24328,24325,24325,24259,24259,24033,24033,23973,23973,23885,23885,23813,23813,23736,23736,23612,23612,23562,23562,23533,23533,23351,23351,23308,23308,23296,23296,23266,23266,23176,23176,23126,23126,23081,23081,23074,23074,23007,23007,22940,22940,22736,22736,22694,22694,22662,22662,22578,22578,22550,22550,22504,22504,22316,22316,22278,22278,22086,22086,22054,22054,22051,22051,22025,22025,21992,21992,21968,21968,21935,21935,21927,21927,21867,21867,21852,21852,21841,21841,21736,21736,21726,21726,21713,21713,21672,21672,21601,21601,21478,21478,21462,21462,21461,21461,21432,21432,21378,21378,21277,21277,21254,21254,21203,21203,21147,21147,21134,21134,21132,21132,21109,21109,21035,21035,20925,20925,20906,20906,20900,20900,20871,20871,20848,20848,20776,20776,20594,20594,20529,20529,20448,20448,20301,20301,20236,20236,20160,20160,20075,20075,20030,20030,19985,19985,19888,19888,19867,19867,19830,19830,19781,19781,19779,19779,19765,19765,19756,19756,19670,19670,19646,19646,19636,19636,19620,19620,19592,19592,19517,19517,19506,19506,19500,19500,19346,19346,19321,19321,19261,19261,19189,19189,19122,19122,18990,18990,18952,18952,18944,18944,18927,18927,18912,18912,18886,18886,18799,18799,18794,18794,18744,18744,18671,18671,18532,18532,18528,18528,18505,18505,18475,18475,18464,18464,18462,18462,18419,18419,18398,18398,18377,18377,18365,18365,18334,18334,18318,18318,18281,18281,18264,18264,18151,18151,18056,18056,18013,18013,17963,17963,17938,17938,17921,17921,17886,17886,17867,17867,17782,17782,17761,17761,17707,17707,17632,17632,17631,17631,17611,17611,17589,17589,17488,17488,17481,17481,17461,17461,17430,17430,17427,17427,17421,17421,17358,17358,17321,17321,17299,17299,17273,17273,17232,17232,17199,17199,17150,17150,17144,17144,17121,17121,17071,17071,17035,17035,17012,17012,17007,17007,16995,16995,16928,16928,16923,16923,16906,16906,16799,16799,16789,16789,16754,16754,16750,16750,16736,16736,16687,16687,16658,16658,16641,16641,16616,16616,16538,16538,16520,16520,16465,16465,16450,16450,16337,16337,16324,16324,16319,16319,16305,16305,16300,16300,16280,16280,16264,16264,16235,16235,16225,16225,16216,16216,16194,16194,16192,16192,16184,16184,16135,16135,16075,16075,16044,16044,16021,16021,15996,15996,15978,15978,15975,15975,15948,15948,15914,15914,15882,15882,15881,15881,15875,15875,15872,15872,15815,15815,15794,15794,15776,15776,15775,15775,15771,15771,15754,15754,15752,15752,15747,15747,15734,15734,15709,15709,15694,15694,15689,15689,15603,15603,15588,15588,15582,15582,15559,15559,15545,15545,15498,15498,15490,15490,15480,15480,15473,15473,15415,15415,15409,15409,15363,15363,15301,15301,15250,15250,15248,15248,15244,15244,15166,15166,15153,15153,15142,15142,15132,15132,15125,15125,15124,15124,15120,15120,15110,15110,15109,15109,15106,15106,15096,15096,15081,15081,15069,15069,15064,15064,15027,15027,15018,15018,15009,15009,15005,15005,14997,14997,14987,14987,14980,14980,14967,14967,14960,14960,14952,14952,14949,14949,14933,14933,14911,14911,14909,14909,14878,14878,14860,14860,14815,14815,14811,14811,14810,14810,14794,14794,14792,14792,14768,14768,14758,14758,14757,14757,14699,14699,14689,14689,14677,14677,14629,14629,14628,14628,14626,14626,14582,14582,14566,14566,14536,14536,14468,14468,14461,14461,14453,14453,14431,14431,14414,14414,14398,14398,14372,14372,14366,14366,14308,14308,14283,14283,14267,14267,14257,14257,14203,14203,14202,14202,14158,14158,14137,14137,14136,14136,14117,14117,14048,14048,14043,14043,14002,14002,13965,13965,13958,13958,13956,13956,13894,13894,13888,13888,13880,13880,13870,13870,13863,13863,13824,13824,13794,13794,13777,13777,13769,13769,13754,13754,13740,13740,13730,13730,13698,13698,13665,13665,13643,13643,13639,13639,13626,13626,13614,13614,13594,13594,13572,13572,13556,13556,13554,13554,13544,13544,13542,13542,13531,13531,13528,13528,13518,13518,13484,13484,13483,13483,13474,13474,13455,13455,13440,13440,13429,13429,13424,13424,13370,13370,13358,13358,13357,13357,13346,13346,13339,13339,13336,13336,13314,13314,13283,13283,13272,13272,13258,13258,13256,13256,13244,13244,13193,13193,13182,13182,13146,13146,13137,13137,13125,13125,13106,13106,13102,13102,13089,13089,13080,13080,13076,13076,13057,13057,13047,13047,13043,13043,13040,13040,13034,13034,13030,13030,13025,13025,12998,12998,12992,12992,12979,12979,12951,12951,12947,12947,12941,12941,12916,12916,12898,12898,12890,12890,12886,12886,12882,12882,12879,12879,12862,12862,12861,12861,12836,12836,12835,12835,12813,12813,12784,12784,12780,12780,12778,12778,12744,12744,12719,12719,12711,12711,12692,12692,12687,12687,12680,12680,12677,12677,12669,12669,12655,12655,12652,12652,12641,12641,12640,12640,12612,12612,12610,12610,12606,12606,12602,12602,12557,12557,12554,12554,12533,12533,12529,12529,12509,12509,12498,12498,12497,12497,12488,12488,12468,12468,12460,12460,12455,12455,12452,12452,12435,12435,12430,12430,12404,12404,12403,12403,12397,12397,12394,12394,12392,12392,12381,12381,12346,12346,12338,12338,12331,12331,12290,12290,12287,12287,12272,12272,12258,12258,12234,12234,12233,12233,12231,12231,12229,12229,12225,12225,12214,12214,12197,12197,12187,12187,12184,12184,12182,12182,12171,12171,12136,12136,12084,12084,12075,12075,12070,12070,12034,12034,12009,12009,12007,12007,11995,11995,11973,11973,11970,11970,11963,11963,11952,11952,11925,11925,11922,11922,11886,11886,11863,11863,11857,11857,11844,11844,11839,11839,11825,11825,11818,11818,11805,11805,11794,11794,11787,11787,11782,11782,11761,11761,11749,11749,11734,11734,11714,11714,11682,11682,11676,11676,11670,11670,11666,11666,11664,11664,11660,11660,11652,11652,11622,11622,11614,11614,11603,11603,11594,11594,11591,11591,11555,11555,11552,11552,11541,11541,11534,11534,11532,11532,11515,11515,11508,11508,11497,11497,11489,11489,11481,11481,11480,11480,11462,11462,11461,11461,11447,11447,11443,11443,11442,11442,11435,11435,11429,11429,11427,11427,11419,11419,11413,11413,11401,11401,11397,11397,11395,11395,11383,11383,11380,11380,11375,11375,11373,11373,11371,11371,11361,11361,11345,11345,11330,11330,11310,11310,11262,11262,11258,11258,11246,11246,11245,11245,11243,11243,11242,11242,11240,11240,11236,11236,11214,11214,11213,11213,11200,11200,11194,11194,11192,11192,11191,11191,11175,11175,11161,11161,11158,11158,11145,11145,11142,11142,11137,11137,11130,11130,11115,11115,11111,11111,11101,11101,11094,11094,11054,11054,11029,11029,11024,11024,11019,11019,11002,11002,10982,10982,10978,10978,10973,10973,10955,10955,10952,10952,10932,10932,10926,10926,10909,10909,10906,10906,10905,10905,10895,10895,10855,10855,10852,10852,10820,10820,10816,10816,10797,10797,10792,10792,10789,10789,10778,10778,10777,10777,10763,10763,10740,10740,10712,10712,10684,10684,10679,10679,10669,10669,10667,10667,10656,10656,10637,10637,10634,10634,10630,10630,10597,10597,10591,10591,10578,10578,10545,10545,10506,10506,10505,10505,10490,10490,10484,10484,10474,10474,10470,10470,10464,10464,10447,10447,10444,10444,10442,10442,10437,10437,10429,10429,10428,10428,10426,10426,10421,10421,10420,10420,10416,10416,10412,10412,10411,10411,10406,10406,10405,10405,10404,10404,10394,10394,10391,10391,10363,10363,10359,10359,10350,10350,10346,10346,10335,10335,10324,10324,10318,10318,10314,10314,10305,10305,10296,10296,10294,10294,10291,10291,10283,10283,10278,10278,10253,10253,10234,10234,10224,10224,10223,10223,10210,10210,10208,10208,10205,10205,10198,10198,10193,10193,10173,10173,10159,10159,10150,10150,10130,10130,10126,10126,10122,10122,10118,10118,10112,10112,10111,10111,10098,10098,10096,10096,10089,10089,10072,10072,10063,10063,10051,10051,10050,10050,10047,10047,10023,10023,10001,10001,9993,9993,9991,9991,9984,9984,9972,9972,9969,9969,9964,9964,9949,9949,9948,9948,9935,9935,9921,9921,9916,9916,9914,9914,9909,9909,9908,9908,9904,9904,9898,9898,9867,9867,9865,9865,9863,9863,9858,9858,9852,9852,9846,9846,9836,9836,9832,9832,9827,9827,9821,9821,9820,9820,9817,9817,9800,9800,9791,9791,9782,9782,9765,9765,9764,9764,9751,9751,9744,9744,9743,9743,9740,9740,9735,9735,9728,9728,9727,9727,9725,9725,9712,9712,9704,9704,9701,9701,9699,9699,9697,9697,9695,9695,9678,9678,9675,9675,9674,9674,9671,9671,9668,9668,9667,9667,9665,9665,9638,9638,9635,9635,9627,9627,9612,9612,9611,9611,9595,9595,9592,9592,9590,9590,9576,9576,9570,9570,9559,9559,9553,9553,9549,9549,9548,9548,9544,9544,9522,9522,9515,9515,9512,9512,9507,9507,9502,9502,9491,9491,9485,9485,9484,9484,9478,9478,9474,9474,9462,9462,9458,9458,9429,9429,9422,9422,9393,9393,9390,9390,9375,9375,9365,9365,9355,9355,9348,9348,9344,9344,9342,9342,9329,9329,9321,9321,9319,9319,9316,9316,9315,9315,9301,9301,9284,9284,9281,9281,9274,9274,9271,9271,9266,9266,9258,9258,9250,9250,9249,9249,9229,9229,9221,9221,9215,9215,9206,9206,9199,9199,9195,9195,9193,9193,9167,9167,9163,9163,9155,9155,9154,9154,9147,9147,9145,9145,9125,9125,9096,9096,9087,9087,9085,9085,9082,9082,9072,9072,9067,9067,9057,9057,9056,9056,9049,9049,9046,9046,9045,9045,9043,9043,9036,9036,9033,9033,9016,9016,9012,9012,8961,8961,8947,8947,8946,8946,8911,8911,8905,8905,8900,8900,8896,8896,8895,8895,8878,8878,8871,8871,8867,8867,8851,8851,8850,8850,8846,8846,8827,8827,8824,8824,8815,8815,8803,8803,8801,8801,8796,8796,8784,8784,8783,8783,8769,8769,8768,8768,8767,8767,8765,8765,8756,8756,8753,8753,8751,8751,8750,8750,8745,8745,8744,8744,8739,8739,8737,8737,8718,8718,8715,8715,8713,8713,8707,8707,8706,8706,8697,8697,8695,8695,8662,8662,8659,8659,8656,8656,8653,8653,8650,8650,8648,8648,8640,8640,8637,8637,8632,8632,8625,8625,8620,8620,8612,8612,8608,8608,8594,8594,8584,8584,8578,8578,8569,8569,8559,8559,8554,8554,8549,8549,8547,8547,8543,8543,8539,8539,8537,8537,8534,8534,8532,8532,8528,8528,8522,8522,8514,8514,8509,8509,8493,8493,8492,8492,8490,8490,8489,8489,8477,8477,8469,8469,8466,8466,8450,8450,8447,8447,8446,8446,8445,8445,8440,8440,8434,8434,8425,8425,8421,8421,8420,8420,8418,8418,8411,8411,8410,8410,8409,8409,8408,8408,8402,8402,8400,8400,8397,8397,8395,8395,8390,8390,8385,8385,8377,8377,8367,8367,8366,8366,8352,8352,8350,8350,8343,8343,8338,8338,8332,8332,8326,8326,8323,8323,8322,8322,8316,8316,8313,8313,8304,8304,8295,8295,8279,8279,8274,8274,8262,8262,8255,8255,8251,8251,8234,8234,8226,8226,8224,8224,8221,8221,8213,8213,8212,8212,8207,8207,8206,8206,8203,8203,8201,8201,8199,8199,8179,8179,8178,8178,8176,8176,8164,8164,8159,8159,8147,8147,8142,8142,8140,8140,8137,8137,8136,8136,8132,8132,8130,8130,8123,8123,8120,8120,8112,8112,8107,8107,8105,8105,8102,8102,8095,8095,8088,8088,8085,8085,8084,8084,8082,8082,8072,8072,8067,8067,8064,8064,8061,8061,8045,8045,8043,8043,8038,8038,8036,8036,8033,8033,8028,8028,8018,8018,8015,8015,8006,8006,8000,8000,7997,7997,7993,7993,7992,7992,7989,7989,7988,7988,7985,7985,7980,7980,7970,7970,7967,7967,7962,7962,7949,7949,7948,7948,7933,7933,7932,7932,7927,7927,7925,7925,7923,7923,7915,7915,7914,7914,7913,7913,7907,7907,7904,7904,7899,7899,7881,7881,7875,7875,7873,7873,7871,7871,7869,7869,7864,7864,7858,7858,7857,7857,7856,7856,7855,7855,7850,7850,7848,7848,7842,7842,7836,7836,7834,7834,7830,7830,7827,7827,7825,7825,7823,7823,7819,7819,7810,7810,7803,7803,7802,7802,7797,7797,7774,7774,7765,7765,7764,7764,7763,7763,7756,7756,7753,7753,7741,7741,7737,7737,7722,7722,7716,7716,7706,7706,7704,7704,7698,7698,7686,7686,7684,7684,7672,7672,7664,7664,7645,7645,7637,7637,7634,7634,7624,7624,7623,7623,7621,7621,7619,7619,7604,7604,7603,7603,7594,7594,7582,7582,7580,7580,7575,7575,7568,7568,7566,7566,7564,7564,7560,7560,7558,7558,7554,7554,7552,7552,7548,7548,7540,7540,7538,7538,7535,7535,7524,7524,7518,7518,7517,7517,7513,7513,7512,7512,7508,7508,7507,7507,7503,7503,7489,7489,7488,7488,7483,7483,7477,7477,7476,7476,7467,7467,7463,7463,7460,7460,7442,7442,7440,7440,7430,7430,7429,7429,7418,7418,7417,7417,7413,7413,7412,7412,7404,7404,7402,7402,7400,7400,7398,7398,7391,7391,7386,7386,7384,7384,7376,7376,7370,7370,7368,7368,7366,7366,7363,7363,7356,7356,7354,7354,7349,7349,7346,7346,7340,7340,7339,7339,7318,7318,7317,7317,7315,7315,7309,7309,7308,7308,7305,7305,7297,7297,7295,7295,7289,7289,7288,7288,7277,7277,7259,7259,7244,7244,7242,7242,7237,7237,7234,7234,7230,7230,7227,7227,7226,7226,7225,7225,7223,7223,7215,7215,7211,7211,7207,7207,7206,7206,7205,7205,7193,7193,7192,7192,7185,7185,7184,7184,7182,7182,7180,7180,7175,7175,7168,7168,7164,7164,7156,7156,7155,7155,7154,7154,7153,7153,7149,7149,7139,7139,7133,7133,7131,7131,7126,7126,7120,7120,7108,7108,7089,7089,7083,7083,7082,7082,7076,7076,7066,7066,7059,7059,7052,7052,7050,7050,7047,7047,7039,7039,7038,7038,7037,7037,7034,7034,7031,7031,7026,7026,7018,7018,7010,7010,7005,7005,7003,7003,7002,7002,7000,7000,6994,6994,6985,6985,6982,6982,6981,6981,6978,6978,6972,6972,6968,6968,6966,6966,6965,6965,6955,6955,6952,6952,6946,6946,6942,6942,6939,6939,6930,6930,6929,6929,6927,6927,6922,6922,6917,6917,6913,6913,6908,6908,6907,6907,6906,6906,6898,6898,6896,6896,6894,6894,6892,6892,6890,6890,6889,6889,6881,6881,6876,6876,6874,6874,6863,6863,6861,6861,6860,6860,6854,6854,6842,6842,6835,6835,6828,6828,6825,6825,6816,6816,6815,6815,6814,6814,6810,6810,6800,6800,6799,6799,6798,6798,6797,6797,6795,6795,6794,6794,6793,6793,6790,6790,6789,6789,6787,6787,6786,6786,6782,6782,6769,6769,6762,6762,6760,6760,6750,6750,6743,6743,6740,6740,6735,6735,6734,6734,6732,6732,6724,6724,6715,6715,6711,6711,6710,6710,6709,6709,6708,6708,6704,6704,6700,6700,6694,6694,6693,6693,6692,6692,6688,6688,6683,6683,6680,6680,6671,6671,6669,6669,6668,6668,6663,6663,6660,6660,6659,6659,6645,6645,6641,6641,6640,6640,6634,6634,6632,6632,6625,6625,6618,6618,6615,6615,6612,6612,6608,6608,6605,6605,6602,6602,6599,6599,6597,6597,6593,6593,6587,6587,6584,6584,6583,6583,6579,6579,6577,6577,6575,6575,6571,6571,6568,6568,6566,6566,6565,6565,6553,6553,6550,6550,6547,6547,6546,6546,6537,6537,6536,6536,6532,6532,6531,6531,6521,6521,6520,6520,6508,6508,6502,6502,6492,6492,6485,6485,6478,6478,6477,6477,6467,6467,6461,6461,6459,6459,6458,6458,6449,6449,6448,6448,6443,6443,6442,6442,6439,6439,6438,6438,6435,6435,6431,6431,6427,6427,6425,6425,6422,6422,6421,6421,6419,6419,6414,6414,6408,6408,6402,6402,6401,6401,6400,6400,6396,6396,6395,6395,6392,6392,6390,6390,6383,6383,6381,6381,6378,6378,6375,6375,6361,6361,6359,6359,6356,6356,6355,6355,6353,6353,6351,6351,6350,6350,6349,6349,6347,6347,6343,6343,6342,6342,6338,6338,6331,6331,6329,6329,6322,6322,6317,6317,6314,6314,6313,6313,6310,6310,6305,6305,6296,6296,6295,6295,6294,6294,6290,6290,6287,6287,6277,6277,6271,6271,6267,6267,6265,6265,6260,6260,6259,6259,6257,6257,6243,6243,6241,6241,6231,6231,6228,6228,6222,6222,6219,6219,6211,6211,6204,6204,6196,6196,6190,6190,6186,6186,6182,6182,6180,6180,6178,6178,6175,6175,6168,6168,6162,6162,6159,6159,6158,6158,6155,6155,6148,6148,6145,6145,6144,6144,6141,6141,6140,6140,6138,6138,6133,6133,6131,6131,6126,6126,6123,6123,6121,6121,6114,6114,6111,6111,6103,6103,6102,6102,6100,6100,6099,6099,6097,6097,6090,6090,6087,6087,6086,6086,6085,6085,6082,6082,6077,6077,6075,6075,6073,6073,6072,6072,6068,6068,6053,6053,6052,6052,6051,6051,6034,6034,6031,6031,6030,6030,6029,6029,6028,6028,6027,6027,6025,6025,6021,6021,6017,6017,6014,6014,6011,6011,6009,6009,6004,6004,6000,6000,5992,5992,5991,5991,5990,5990,5988,5988,5983,5983,5981,5981,5980,5980,5976,5976,5972,5972,5969,5969,5967,5967,5964,5964,5963,5963,5962,5962,5961,5961,5957,5957,5956,5956,5955,5955,5954,5954,5947,5947,5946,5946,5945,5945,5944,5944,5941,5941,5938,5938,5937,5937,5936,5936,5933,5933,5932,5932,5929,5929,5928,5928,5926,5926,5925,5925,5923,5923,5921,5921,5916,5916,5914,5914,5913,5913,5910,5910,5907,5907,5905,5905,5904,5904,5903,5903,5902,5902,5894,5894,5891,5891,5890,5890,5886,5886,5885,5885,5881,5881,5880,5880,5879,5879,5876,5876,5870,5870,5866,5866,5863,5863,5859,5859,5853,5853,5852,5852,5851,5851,5850,5850,5849,5849,5848,5848,5840,5840,5838,5838,5836,5836,5834,5834,5832,5832,5829,5829,5827,5827,5826,5826,5825,5825,5821,5821,5817,5817,5816,5816,5813,5813,5807,5807,5802,5802,5801,5801,5798,5798,5796,5796,5792,5792,5790,5790,5788,5788,5787,5787,5776,5776,5775,5775,5773,5773,5768,5768,5767,5767,5766,5766,5761,5761,5759,5759,5757,5757,5756,5756,5755,5755,5752,5752,5748,5748,5746,5746,5742,5742,5740,5740,5737,5737,5735,5735,5728,5728,5725,5725,5720,5720,5718,5718,5716,5716,5715,5715,5714,5714,5712,5712,5711,5711,5710,5710,5709,5709,5708,5708,5707,5707,5703,5703,5702,5702,5699,5699,5698,5698,5691,5691,5690,5690,5687,5687,5683,5683,5681,5681,5680,5680,5679,5679,5678,5678,5677,5677,5676,5676,5673,5673,5669,5669,5666,5666,5665,5665,5661,5661,5660,5660,5659,5659,5655,5655,5653,5653,5651,5651,5638,5638,5634,5634,5628,5628,5625,5625,5623,5623,5620,5620,5616,5616,5615,5615,5612,5612,5602,5602,5601,5601,5600,5600,5599,5599,5597,5597,5596,5596,5594,5594,5592,5592,5591,5591,5588,5588,5587,5587,5584,5584,5581,5581,5580,5580,5579,5579,5578,5578,5576,5576,5573,5573,5570,5570,5562,5562,5560,5560,5559,5559,5555,5555,5550,5550,5549,5549,5546,5546,5544,5544,5543,5543,5542,5542,5541,5541,5539,5539,5537,5537,5535,5535,5532,5532,5531,5531,5528,5528,5525,5525,5522,5522,5520,5520,5517,5517,5515,5515,5506,5506,5505,5505,5499,5499,5497,5497,5496,5496,5494,5494,5493,5493,5491,5491,5487,5487,5485,5485,5482,5482,5478,5478,5475,5475,5473,5473,5472,5472,5469,5469,5465,5465,5464,5464,5463,5463,5458,5458,5453,5453,5451,5451,5450,5450,5449,5449,5445,5445,5444,5444,5441,5441,5437,5437,5435,5435,5423,5423,5422,5422,5421,5421,5419,5419,5418,5418,5414,5414,5410,5410,5404,5404,5403,5403,5400,5400,5398,5398,5396,5396,5395,5395,5394,5394,5392,5392,5391,5391,5390,5390,5387,5387,5385,5385,5384,5384,5378,5378,5375,5375,5371,5371,5370,5370,5367,5367,5366,5366,5365,5365,5363,5363,5358,5358,5356,5356,5355,5355,5350,5350,5349,5349,5346,5346,5345,5345,5344,5344,5343,5343,5342,5342,5340,5340,5338,5338,5335,5335,5334,5334,5333,5333,5330,5330,5329,5329,5328,5328,5327,5327,5325,5325,5321,5321,5320,5320,5319,5319,5316,5316,5314,5314,5308,5308,5307,5307,5299,5299,5296,5296,5295,5295,5293,5293,5292,5292,5291,5291,5286,5286,5276,5276,5274,5274,5269,5269,5268,5268,5264,5264,5262,5262,5254,5254,5251,5251,5250,5250,5248,5248,5247,5247,5246,5246,5233,5233,5232,5232,5229,5229,5228,5228,5227,5227,5216,5216,5215,5215,5210,5210,5206,5206,5198,5198,5196,5196,5192,5192,5191,5191,5189,5189,5188,5188,5187,5187,5185,5185,5184,5184,5183,5183,5181,5181,5180,5180,5173,5173,5171,5171,5169,5169,5167,5167,5166,5166,5162,5162,5158,5158,5155,5155,5153,5153,5152,5152,5150,5150,5147,5147,5139,5139,5138,5138,5136,5136,5133,5133,5132,5132,5131,5131,5130,5130,5129,5129,5128,5128,5120,5120,5117,5117,5116,5116,5113,5113,5112,5112,5107,5107,5105,5105,5102,5102,5100,5100,5090,5090,5089,5089,5088,5088,5083,5083,5081,5081,5080,5080,5079,5079,5077,5077,5074,5074,5072,5072,5071,5071,5070,5070,5067,5067,5066,5066,5064,5064,5063,5063,5060,5060,5058,5058,5057,5057,5054,5054,5052,5052,5051,5051,5050,5050,5045,5045,5043,5043,5041,5041,5040,5040,5039,5039,5036,5036,5034,5034,5033,5033,5031,5031,5029,5029,5028,5028,5024,5024,5015,5015,5013,5013,5012,5012,5006,5006,5005,5005,5004,5004,4999,4999,4997,4997,4993,4993,4992,4992,4990,4990,4984,4984,4983,4983,4982,4982,4977,4977,4976,4976,4973,4973,4972,4972,4968,4968,4960,4960,4957,4957,4956,4956,4953,4953,4950,4950,4949,4949,4948,4948,4945,4945,4944,4944,4941,4941,4940,4940,4938,4938,4936,4936,4931,4931,4928,4928,4921,4921,4920,4920,4919,4919,4915,4915,4914,4914,4912,4912,4911,4911,4907,4907,4905,4905,4902,4902,4901,4901,4898,4898,4896,4896,4895,4895,4894,4894,4893,4893,4892,4892,4888,4888,4887,4887,4886,4886,4883,4883,4882,4882,4880,4880,4878,4878,4873,4873,4871,4871,4870,4870,4867,4867,4866,4866,4865,4865,4864,4864,4847,4847,4845,4845,4844,4844,4840,4840,4839,4839,4834,4834,4833,4833,4831,4831,4829,4829,4828,4828,4822,4822,4821,4821,4820,4820,4816,4816,4814,4814,4813,4813,4805,4805,4804,4804,4802,4802,4800,4800,4799,4799,4795,4795,4794,4794,4792,4792,4790,4790,4788,4788,4787,4787,4783,4783,4780,4780,4776,4776,4772,4772,4766,4766,4764,4764,4753,4753,4751,4751,4749,4749,4744,4744,4739,4739,4738,4738,4736,4736,4734,4734,4731,4731,4729,4729,4726,4726,4725,4725,4722,4722,4721,4721,4720,4720,4717,4717,4713,4713,4711,4711,4710,4710,4705,4705,4704,4704,4702,4702,4701,4701,4700,4700,4699,4699,4698,4698,4695,4695,4691,4691,4689,4689,4685,4685,4683,4683,4681,4681,4678,4678,4677,4677,4675,4675,4674,4674,4671,4671,4670,4670,4669,4669,4663,4663,4658,4658,4656,4656,4645,4645,4643,4643,4642,4642,4637,4637,4636,4636,4635,4635,4633,4633,4630,4630,4627,4627,4624,4624,4623,4623,4613,4613,4612,4612,4611,4611,4610,4610,4609,4609,4603,4603,4601,4601,4600,4600,4593,4593,4591,4591,4590,4590,4589,4589,4587,4587,4583,4583,4582,4582,4578,4578,4576,4576,4561,4561,4560,4560,4558,4558,4553,4553,4551,4551,4550,4550,4549,4549,4546,4546,4545,4545,4541,4541,4540,4540,4539,4539,4536,4536,4534,4534,4533,4533,4531,4531,4528,4528,4524,4524,4522,4522,4521,4521,4519,4519,4518,4518,4514,4514,4512,4512,4511,4511,4509,4509,4499,4499,4497,4497,4489,4489,4488,4488,4487,4487,4486,4486,4485,4485,4476,4476,4475,4475,4474,4474,4473,4473,4470,4470,4468,4468,4467,4467,4463,4463,4454,4454,4453,4453,4452,4452,4451,4451,4450,4450,4447,4447,4445,4445,4443,4443,4441,4441,4438,4438,4437,4437,4436,4436,4434,4434,4431,4431,4429,4429,4427,4427,4425,4425,4424,4424,4422,4422,4420,4420,4419,4419,4418,4418,4417,4417,4415,4415,4412,4412,4410,4410,4409,4409,4408,4408,4403,4403,4401,4401,4399,4399,4397,4397,4393,4393,4388,4388,4387,4387,4385,4385,4380,4380,4379,4379,4376,4376,4374,4374,4373,4373,4372,4372,4370,4370,4368,4368,4367,4367,4366,4366,4362,4362,4358,4358,4357,4357,4351,4351,4347,4347,4346,4346,4342,4342,4340,4340,4339,4339,4337,4337,4334,4334,4333,4333,4330,4330,4328,4328,4326,4326,4324,4324,4318,4318,4317,4317,4314,4314,4312,4312,4309,4309,4308,4308,4302,4302,4299,4299,4296,4296,4285,4285,4283,4283,4282,4282,4281,4281,4279,4279,4276,4276,4275,4275,4274,4274,4273,4273,4272,4272,4268,4268,4267,4267,4265,4265,4263,4263,4251,4251,4247,4247,4245,4245,4243,4243,4242,4242,4240,4240,4239,4239,4237,4237,4236,4236,4235,4235,4234,4234,4233,4233,4231,4231,4229,4229,4226,4226,4225,4225,4223,4223,4222,4222,4221,4221,4219,4219,4217,4217,4214,4214,4213,4213,4211,4211,4210,4210,4209,4209,4207,4207,4205,4205,4201,4201,4199,4199,4193,4193,4190,4190,4189,4189,4188,4188,4187,4187,4186,4186,4185,4185,4184,4184,4181,4181,4180,4180,4177,4177,4175,4175,4174,4174,4172,4172,4171,4171,4169,4169,4166,4166,4162,4162,4161,4161,4160,4160,4159,4159,4158,4158,4155,4155,4152,4152,4145,4145,4143,4143,4136,4136,4130,4130,4129,4129,4128,4128,4127,4127,4121,4121,4120,4120,4118,4118,4116,4116,4113,4113,4108,4108,4107,4107,4106,4106,4104,4104,4103,4103,4102,4102,4099,4099,4098,4098,4097,4097,4095,4095,4093,4093,4082,4082,4079,4079,4078,4078,4076,4076,4072,4072,4070,4070,4069,4069,4067,4067,4066,4066,4065,4065,4063,4063,4062,4062,4060,4060,4056,4056,4055,4055,4054,4054,4051,4051,4049,4049,4048,4048,4044,4044,4043,4043,4039,4039,4036,4036,4033,4033,4032,4032,4031,4031,4030,4030,4027,4027,4019,4019,4018,4018,4017,4017,4015,4015,4013,4013,4007,4007,4001,4001,3999,3999,3998,3998,3994,3994,3991,3991,3989,3989,3988,3988,3987,3987,3980,3980,3978,3978,3977,3977,3976,3976,3972,3972,3970,3970,3969,3969,3968,3968,3967,3967,3964,3964,3958,3958,3956,3956,3955,3955,3953,3953,3950,3950,3949,3949,3947,3947,3944,3944,3943,3943,3942,3942,3941,3941,3940,3940,3936,3936,3935,3935,3934,3934,3933,3933,3931,3931,3930,3930,3929,3929,3928,3928,3926,3926,3925,3925,3923,3923,3918,3918,3907,3907,3906,3906,3903,3903,3902,3902,3900,3900,3898,3898,3896,3896,3894,3894,3893,3893,3891,3891,3889,3889,3888,3888,3887,3887,3885,3885,3883,3883,3879,3879,3878,3878,3875,3875,3873,3873,3871,3871,3870,3870,3869,3869,3867,3867,3866,3866,3865,3865,3860,3860,3859,3859,3856,3856,3855,3855,3853,3853,3852,3852,3850,3850,3849,3849,3848,3848,3843,3843,3841,3841,3840,3840,3836,3836,3835,3835,3832,3832,3827,3827,3824,3824,3819,3819,3818,3818,3817,3817,3814,3814,3805,3805,3804,3804,3799,3799,3790,3790,3788,3788,3786,3786,3785,3785,3784,3784,3781,3781,3779,3779,3775,3775,3774,3774,3769,3769,3768,3768,3759,3759,3758,3758,3757,3757,3754,3754,3748,3748,3746,3746,3743,3743,3739,3739,3736,3736,3732,3732,3730,3730,3726,3726,3724,3724,3721,3721,3720,3720,3719,3719,3718,3718,3717,3717,3716,3716,3714,3714,3711,3711,3709,3709,3708,3708,3707,3707,3706,3706,3705,3705,3703,3703,3700,3700,3699,3699,3698,3698,3696,3696,3695,3695,3693,3693,3692,3692,3691,3691,3689,3689,3685,3685,3684,3684,3683,3683,3682,3682,3680,3680,3677,3677,3673,3673,3665,3665,3662,3662,3660,3660,3659,3659,3657,3657,3656,3656,3655,3655,3654,3654,3653,3653,3652,3652,3651,3651,3650,3650,3649,3649,3647,3647,3645,3645,3644,3644,3643,3643,3642,3642,3635,3635,3633,3633,3630,3630,3629,3629,3628,3628,3627,3627,3625,3625,3624,3624,3621,3621,3619,3619,3615,3615,3611,3611,3609,3609,3607,3607,3603,3603,3598,3598,3594,3594,3593,3593,3592,3592,3589,3589,3587,3587,3581,3581,3579,3579,3578,3578,3577,3577,3576,3576,3575,3575,3573,3573,3572,3572,3571,3571,3570,3570,3569,3569,3566,3566,3565,3565,3564,3564,3563,3563,3562,3562,3561,3561,3560,3560,3559,3559,3558,3558,3557,3557,3555,3555,3551,3551,3550,3550,3549,3549,3548,3548,3545,3545,3544,3544,3543,3543,3542,3542,3541,3541,3538,3538,3536,3536,3535,3535,3529,3529,3526,3526,3524,3524,3522,3522,3518,3518,3515,3515,3514,3514,3513,3513,3512,3512,3506,3506,3505,3505,3501,3501,3500,3500,3496,3496,3495,3495,3494,3494,3493,3493,3492,3492,3489,3489,3485,3485,3482,3482,3480,3480,3479,3479,3475,3475,3473,3473,3471,3471,3470,3470,3465,3465,3461,3461,3460,3460,3458,3458,3456,3456,3453,3453,3452,3452,3450,3450,3449,3449,3448,3448,3443,3443,3442,3442,3440,3440,3436,3436,3435,3435,3432,3432,3431,3431,3429,3429,3427,3427,3426,3426,3425,3425,3424,3424,3422,3422,3421,3421,3420,3420,3419,3419,3418,3418,3417,3417,3413,3413,3412,3412,3410,3410,3408,3408,3407,3407,3404,3404,3402,3402,3401,3401,3397,3397,3396,3396,3394,3394,3393,3393,3392,3392,3391,3391,3386,3386,3385,3385,3381,3381,3380,3380,3379,3379,3376,3376,3375,3375,3374,3374,3370,3370,3369,3369,3367,3367,3366,3366,3364,3364,3363,3363,3360,3360,3359,3359,3357,3357,3356,3356,3355,3355,3354,3354,3352,3352,3348,3348,3346,3346,3345,3345,3344,3344,3343,3343,3341,3341,3338,3338,3337,3337,3335,3335,3334,3334,3332,3332,3331,3331,3330,3330,3329,3329,3327,3327,3326,3326,3325,3325,3322,3322,3321,3321,3320,3320,3317,3317,3316,3316,3315,3315,3314,3314,3313,3313,3312,3312,3311,3311,3310,3310,3308,3308,3307,3307,3305,3305,3304,3304,3302,3302,3301,3301,3300,3300,3299,3299,3298,3298,3297,3297,3296,3296,3292,3292,3291,3291,3288,3288,3286,3286,3285,3285,3281,3281,3279,3279,3275,3275,3274,3274,3273,3273,3272,3272,3269,3269,3263,3263,3262,3262,3261,3261,3260,3260,3259,3259,3258,3258,3253,3253,3252,3252,3251,3251,3250,3250,3249,3249,3248,3248,3247,3247,3245,3245,3244,3244,3242,3242,3241,3241,3240,3240,3239,3239,3238,3238,3235,3235,3233,3233,3232,3232,3231,3231,3229,3229,3225,3225,3224,3224,3222,3222,3218,3218,3214,3214,3213,3213,3212,3212,3211,3211,3210,3210,3208,3208,3207,3207,3206,3206,3205,3205,3200,3200,3198,3198,3196,3196,3190,3190,3188,3188,3187,3187,3186,3186,3184,3184,3183,3183,3181,3181,3180,3180,3179,3179,3176,3176,3175,3175,3173,3173,3170,3170,3169,3169,3166,3166,3165,3165,3164,3164,3160,3160,3155,3155,3151,3151,3150,3150,3149,3149,3148,3148,3147,3147,3146,3146,3145,3145,3142,3142,3141,3141,3138,3138,3136,3136,3134,3134,3133,3133,3125,3125,3121,3121,3120,3120,3115,3115,3114,3114,3113,3113,3109,3109,3107,3107,3106,3106,3104,3104,3101,3101,3100,3100,3099,3099,3098,3098,3097,3097,3095,3095,3093,3093,3092,3092,3091,3091,3089,3089,3086,3086,3084,3084,3081,3081,3077,3077,3076,3076,3074,3074,3073,3073,3072,3072,3071,3071,3070,3070,3068,3068,3066,3066,3065,3065,3064,3064,3062,3062,3055,3055,3053,3053,3052,3052,3050,3050,3049,3049,3046,3046,3044,3044,3043,3043,3042,3042,3040,3040,3039,3039,3038,3038,3037,3037,3036,3036,3031,3031,3030,3030,3027,3027,3025,3025,3023,3023,3022,3022,3020,3020,3019,3019,3018,3018,3017,3017,3013,3013,3008,3008,3005,3005,3004,3004,3001,3001,2998,2998,2997,2997,2995,2995,2994,2994,2993,2993,2991,2991,2989,2989,2988,2988,2986,2986,2985,2985,2984,2984,2983,2983,2980,2980,2979,2979,2975,2975,2974,2974,2973,2973,2971,2971,2969,2969,2968,2968,2967,2967,2966,2966,2964,2964,2963,2963,2962,2962,2959,2959,2958,2958,2957,2957,2956,2956,2955,2955,2953,2953,2950,2950,2947,2947,2946,2946,2943,2943,2942,2942,2940,2940,2939,2939,2938,2938,2937,2937,2935,2935,2933,2933,2932,2932,2931,2931,2930,2930,2929,2929,2923,2923,2920,2920,2916,2916,2914,2914,2912,2912,2908,2908,2906,2906,2905,2905,2904,2904,2903,2903,2899,2899,2897,2897,2895,2895,2894,2894,2893,2893,2892,2892,2889,2889,2886,2886,2885,2885,2883,2883,2882,2882,2881,2881,2880,2880,2879,2879,2877,2877,2875,2875,2873,2873,2872,2872,2871,2871,2870,2870,2869,2869,2866,2866,2865,2865,2864,2864,2863,2863,2862,2862,2861,2861,2860,2860,2859,2859,2858,2858,2857,2857,2856,2856,2852,2852,2851,2851,2847,2847,2846,2846,2845,2845,2843,2843,2840,2840,2838,2838,2837,2837,2835,2835,2834,2834,2833,2833,2832,2832,2831,2831,2830,2830,2828,2828,2825,2825,2824,2824,2822,2822,2820,2820,2819,2819,2818,2818,2816,2816,2815,2815,2814,2814,2811,2811,2808,2808,2807,2807,2806,2806,2805,2805,2800,2800,2798,2798,2796,2796,2795,2795,2793,2793,2791,2791,2788,2788,2787,2787,2786,2786,2785,2785,2783,2783,2782,2782,2781,2781,2780,2780,2778,2778,2777,2777,2776,2776,2775,2775,2774,2774,2772,2772,2771,2771,2770,2770,2769,2769,2768,2768,2765,2765,2764,2764,2763,2763,2761,2761,2759,2759,2758,2758,2756,2756,2754,2754,2748,2748,2746,2746,2744,2744,2737,2737,2736,2736,2732,2732,2731,2731,2728,2728,2723,2723,2722,2722,2721,2721,2720,2720,2718,2718,2717,2717,2716,2716,2715,2715,2710,2710,2709,2709,2706,2706,2705,2705,2703,2703,2701,2701,2700,2700,2697,2697,2696,2696,2692,2692,2691,2691,2690,2690,2689,2689,2687,2687,2686,2686,2684,2684,2683,2683,2682,2682,2681,2681,2679,2679,2678,2678,2677,2677,2674,2674,2673,2673,2672,2672,2671,2671,2670,2670,2668,2668,2667,2667,2665,2665,2664,2664,2663,2663,2662,2662,2661,2661,2660,2660,2659,2659,2658,2658,2657,2657,2655,2655,2654,2654,2653,2653,2652,2652,2650,2650,2648,2648,2646,2646,2644,2644,2643,2643,2642,2642,2637,2637,2635,2635,2633,2633,2630,2630,2629,2629,2624,2624,2620,2620,2617,2617,2616,2616,2610,2610,2609,2609,2606,2606,2605,2605,2603,2603,2600,2600,2599,2599,2598,2598,2597,2597,2596,2596,2595,2595,2592,2592,2591,2591,2590,2590,2589,2589,2588,2588,2584,2584,2583,2583,2582,2582,2581,2581,2579,2579,2578,2578,2577,2577,2575,2575,2573,2573,2572,2572,2569,2569,2568,2568,2567,2567,2566,2566,2565,2565,2563,2563,2562,2562,2560,2560,2558,2558,2557,2557,2556,2556,2555,2555,2552,2552,2551,2551,2549,2549,2548,2548,2547,2547,2546,2546,2545,2545,2544,2544,2542,2542,2541,2541,2539,2539,2538,2538,2536,2536,2534,2534,2532,2532,2531,2531,2530,2530,2520,2520,2519,2519,2513,2513,2512,2512,2511,2511,2509,2509,2505,2505,2504,2504,2503,2503,2502,2502,2501,2501,2500,2500,2498,2498,2496,2496,2495,2495,2494,2494,2491,2491,2489,2489,2488,2488,2487,2487,2486,2486,2485,2485,2484,2484,2483,2483,2482,2482,2481,2481,2480,2480,2478,2478,2477,2477,2476,2476,2473,2473,2468,2468,2467,2467,2465,2465,2464,2464,2462,2462,2461,2461,2459,2459,2457,2457,2456,2456,2452,2452,2448,2448,2447,2447,2445,2445,2444,2444,2442,2442,2441,2441,2440,2440,2438,2438,2436,2436,2433,2433,2432,2432,2431,2431,2430,2430,2429,2429,2426,2426,2425,2425,2423,2423,2421,2421,2420,2420,2419,2419,2418,2418,2415,2415,2413,2413,2411,2411,2409,2409,2408,2408,2406,2406,2405,2405,2404,2404,2403,2403,2402,2402,2400,2400,2398,2398,2397,2397,2394,2394,2392,2392,2389,2389,2388,2388,2387,2387,2386,2386,2385,2385,2384,2384,2382,2382,2381,2381,2379,2379,2377,2377,2376,2376,2375,2375,2374,2374,2373,2373,2372,2372,2371,2371,2369,2369,2368,2368,2367,2367,2366,2366,2364,2364,2362,2362,2360,2360,2358,2358,2357,2357,2355,2355,2353,2353,2352,2352,2350,2350,2349,2349,2348,2348,2345,2345,2344,2344,2342,2342,2341,2341,2340,2340,2339,2339,2338,2338,2337,2337,2335,2335,2333,2333,2331,2331,2330,2330,2329,2329,2325,2325,2324,2324,2322,2322,2321,2321,2320,2320,2319,2319,2313,2313,2312,2312,2311,2311,2310,2310,2309,2309,2307,2307,2306,2306,2304,2304,2302,2302,2300,2300,2299,2299,2298,2298,2297,2297,2296,2296,2293,2293,2288,2288,2287,2287,2286,2286,2283,2283,2282,2282,2281,2281,2280,2280,2279,2279,2278,2278,2277,2277,2275,2275,2274,2274,2273,2273,2272,2272,2271,2271,2270,2270,2269,2269,2268,2268,2267,2267,2266,2266,2265,2265,2262,2262,2261,2261,2260,2260,2259,2259,2258,2258,2257,2257,2254,2254,2252,2252,2251,2251,2250,2250,2249,2249,2247,2247,2246,2246,2243,2243,2240,2240,2239,2239,2238,2238,2236,2236,2235,2235,2233,2233,2232,2232,2231,2231,2230,2230,2229,2229,2228,2228,2226,2226,2224,2224,2222,2222,2220,2220,2217,2217,2214,2214,2213,2213,2211,2211,2209,2209,2208,2208,2205,2205,2203,2203,2202,2202,2201,2201,2198,2198,2196,2196,2195,2195,2194,2194,2193,2193,2192,2192,2191,2191,2188,2188,2185,2185,2182,2182,2181,2181,2180,2180,2179,2179,2178,2178,2177,2177,2175,2175,2174,2174,2173,2173,2172,2172,2169,2169,2168,2168,2166,2166,2165,2165,2164,2164,2163,2163,2162,2162,2161,2161,2160,2160,2159,2159,2157,2157,2152,2152,2151,2151,2149,2149,2148,2148,2146,2146,2145,2145,2143,2143,2142,2142,2141,2141,2140,2140,2139,2139,2138,2138,2137,2137,2135,2135,2134,2134,2132,2132,2131,2131,2130,2130,2129,2129,2127,2127,2126,2126,2124,2124,2123,2123,2122,2122,2121,2121,2120,2120,2119,2119,2118,2118,2117,2117,2116,2116,2114,2114,2113,2113,2112,2112,2111,2111,2109,2109,2108,2108,2107,2107,2106,2106,2105,2105,2104,2104,2103,2103,2102,2102,2101,2101,2100,2100,2099,2099,2095,2095,2094,2094,2093,2093,2092,2092,2091,2091,2088,2088,2087,2087,2086,2086,2084,2084,2082,2082,2081,2081,2080,2080,2076,2076,2074,2074,2073,2073,2072,2072,2071,2071,2070,2070,2069,2069,2068,2068,2067,2067,2066,2066,2063,2063,2062,2062,2061,2061,2060,2060,2059,2059,2058,2058,2056,2056,2055,2055,2054,2054,2053,2053,2051,2051,2050,2050,2048,2048,2047,2047,2046,2046,2045,2045,2044,2044,2043,2043,2042,2042,2041,2041,2039,2039,2038,2038,2035,2035,2034,2034,2031,2031,2030,2030,2029,2029,2027,2027,2026,2026,2024,2024,2023,2023,2022,2022,2021,2021,2020,2020,2019,2019,2018,2018,2017,2017,2015,2015,2014,2014,2013,2013,2010,2010,2009,2009,2008,2008,2007,2007,2006,2006,2004,2004,2003,2003,2001,2001,2000,2000,1999,1999,1998,1998,1996,1996,1995,1995,1993,1993,1992,1992,1990,1990,1989,1989,1987,1987,1986,1986,1984,1984,1983,1983,1982,1982,1980,1980,1979,1979,1978,1978,1977,1977,1976,1976,1975,1975,1974,1974,1973,1973,1972,1972,1971,1971,1970,1970,1969,1969,1968,1968,1967,1967,1966,1966,1964,1964,1962,1962,1960,1960,1957,1957,1956,1956,1955,1955,1954,1954,1953,1953,1952,1952,1951,1951,1950,1950,1949,1949,1948,1948,1947,1947,1946,1946,1945,1945,1944,1944,1942,1942,1941,1941,1940,1940,1939,1939,1938,1938,1937,1937,1936,1936,1935,1935,1934,1934,1933,1933,1932,1932,1931,1931,1930,1930,1929,1929,1927,1927,1926,1926,1925,1925,1924,1924,1923,1923,1920,1920,1919,1919,1918,1918,1917,1917,1916,1916,1914,1914,1913,1913,1912,1912,1911,1911,1909,1909,1908,1908,1907,1907,1906,1906,1904,1904,1903,1903,1901,1901,1900,1900,1899,1899,1898,1898,1895,1895,1893,1893,1892,1892,1889,1889,1887,1887,1885,1885,1884,1884,1883,1883,1882,1882,1881,1881,1879,1879,1877,1877,1876,1876,1874,1874,1873,1873,1871,1871,1869,1869,1868,1868,1867,1867,1866,1866,1864,1864,1863,1863,1862,1862,1861,1861,1859,1859,1858,1858,1857,1857,1854,1854,1853,1853,1852,1852,1851,1851,1850,1850,1849,1849,1847,1847,1846,1846,1845,1845,1843,1843,1842,1842,1841,1841,1838,1838,1835,1835,1834,1834,1833,1833,1832,1832,1830,1830,1829,1829,1828,1828,1827,1827,1824,1824,1823,1823,1822,1822,1821,1821,1820,1820,1819,1819,1818,1818,1817,1817,1816,1816,1814,1814,1813,1813,1812,1812,1811,1811,1810,1810,1809,1809,1808,1808,1807,1807,1806,1806,1805,1805,1804,1804,1803,1803,1802,1802,1801,1801,1800,1800,1799,1799,1796,1796,1794,1794,1793,1793,1792,1792,1791,1791,1789,1789,1788,1788,1786,1786,1785,1785,1784,1784,1783,1783,1782,1782,1781,1781,1780,1780,1779,1779,1778,1778,1777,1777,1776,1776,1775,1775,1774,1774,1773,1773,1772,1772,1770,1770,1769,1769,1768,1768,1767,1767,1765,1765,1764,1764,1763,1763,1759,1759,1758,1758,1755,1755,1754,1754,1752,1752,1751,1751,1750,1750,1747,1747,1746,1746,1744,1744,1743,1743,1742,1742,1741,1741,1740,1740,1735,1735,1733,1733,1732,1732,1730,1730,1729,1729,1727,1727,1726,1726,1725,1725,1724,1724,1723,1723,1722,1722,1721,1721,1719,1719,1717,1717,1716,1716,1715,1715,1714,1714,1713,1713,1711,1711,1710,1710,1709,1709,1707,1707,1706,1706,1705,1705,1704,1704,1703,1703,1702,1702,1701,1701,1700,1700,1699,1699,1698,1698,1697,1697,1695,1695,1694,1694,1692,1692,1689,1689,1688,1688,1687,1687,1686,1686,1683,1683,1682,1682,1681,1681,1679,1679,1678,1678,1677,1677,1676,1676,1675,1675,1674,1674,1670,1670,1669,1669,1668,1668,1667,1667,1666,1666,1664,1664,1663,1663,1661,1661,1660,1660,1659,1659,1658,1658,1657,1657,1656,1656,1654,1654,1653,1653,1652,1652,1651,1651,1650,1650,1648,1648,1647,1647,1645,1645,1643,1643,1642,1642,1641,1641,1640,1640,1639,1639,1638,1638,1637,1637,1636,1636,1635,1635,1634,1634,1633,1633,1632,1632,1631,1631,1630,1630,1627,1627,1626,1626,1625,1625,1623,1623,1622,1622,1620,1620,1619,1619,1618,1618,1617,1617,1616,1616,1615,1615,1614,1614,1613,1613,1612,1612,1611,1611,1610,1610,1609,1609,1608,1608,1607,1607,1606,1606,1605,1605,1604,1604,1603,1603,1601,1601,1600,1600,1599,1599,1597,1597,1596,1596,1593,1593,1592,1592,1591,1591,1589,1589,1587,1587,1586,1586,1585,1585,1584,1584,1583,1583,1582,1582,1581,1581,1580,1580,1579,1579,1578,1578,1575,1575,1574,1574,1573,1573,1572,1572,1571,1571,1570,1570,1569,1569,1568,1568,1567,1567,1566,1566,1565,1565,1564,1564,1560,1560,1559,1559,1558,1558,1557,1557,1556,1556,1555,1555,1554,1554,1551,1551,1550,1550,1549,1549,1548,1548,1547,1547,1546,1546,1545,1545,1544,1544,1543,1543,1542,1542,1541,1541,1540,1540,1538,1538,1537,1537,1536,1536,1535,1535,1534,1534,1533,1533,1531,1531,1529,1529,1528,1528,1527,1527,1526,1526,1525,1525,1524,1524,1523,1523,1522,1522,1521,1521,1520,1520,1518,1518,1517,1517,1516,1516,1515,1515,1514,1514,1513,1513,1512,1512,1511,1511,1510,1510,1508,1508,1507,1507,1506,1506,1505,1505,1502,1502,1499,1499,1498,1498,1496,1496,1495,1495,1494,1494,1493,1493,1492,1492,1491,1491,1490,1490,1489,1489,1488,1488,1487,1487,1486,1486,1485,1485,1484,1484,1483,1483,1482,1482,1481,1481,1480,1480,1479,1479,1478,1478,1477,1477,1476,1476,1475,1475,1474,1474,1473,1473,1472,1472,1471,1471,1470,1470,1469,1469,1468,1468,1467,1467,1466,1466,1465,1465,1464,1464,1463,1463,1462,1462,1461,1461,1460,1460,1459,1459,1458,1458,1457,1457,1454,1454,1452,1452,1451,1451,1450,1450,1449,1449,1448,1448,1445,1445,1444,1444,1443,1443,1441,1441,1440,1440,1439,1439,1436,1436,1434,1434,1433,1433,1432,1432,1431,1431,1430,1430,1429,1429,1428,1428,1426,1426,1424,1424,1423,1423,1422,1422,1421,1421,1420,1420,1418,1418,1417,1417,1416,1416,1413,1413,1412,1412,1411,1411,1410,1410,1408,1408,1407,1407,1406,1406,1405,1405,1404,1404,1403,1403,1402,1402,1401,1401,1400,1400,1399,1399,1398,1398,1396,1396,1395,1395,1394,1394,1391,1391,1390,1390,1389,1389,1388,1388,1387,1387,1386,1386,1385,1385,1384,1384,1383,1383,1382,1382,1381,1381,1380,1380,1379,1379,1378,1378,1377,1377,1376,1376,1375,1375,1374,1374,1373,1373,1372,1372,1371,1371,1370,1370,1369,1369,1368,1368,1366,1366,1365,1365,1364,1364,1363,1363,1360,1360,1359,1359,1357,1357,1356,1356,1355,1355,1354,1354,1353,1353,1352,1352,1351,1351,1349,1349,1348,1348,1346,1346,1345,1345,1343,1343,1342,1342,1341,1341,1339,1339,1338,1338,1337,1337,1336,1336,1334,1334,1333,1333,1332,1332,1331,1331,1330,1330,1329,1329,1328,1328,1327,1327,1325,1325,1323,1323,1322,1322,1321,1321,1319,1319,1318,1318,1317,1317,1316,1316,1315,1315,1314,1314,1313,1313,1311,1311,1310,1310,1309,1309,1308,1308,1307,1307,1304,1304,1303,1303,1301,1301,1300,1300,1298,1298,1295,1295,1292,1292,1291,1291,1289,1289,1288,1288,1287,1287,1286,1286,1285,1285,1284,1284,1283,1283,1282,1282,1281,1281,1280,1280,1279,1279,1278,1278,1277,1277,1276,1276,1275,1275,1274,1274,1273,1273,1272,1272,1271,1271,1270,1270,1269,1269,1268,1268,1266,1266,1265,1265,1264,1264,1263,1263,1262,1262,1261,1261,1260,1260,1258,1258,1257,1257,1256,1256,1255,1255,1254,1254,1253,1253,1252,1252,1251,1251,1248,1248,1247,1247,1246,1246,1245,1245,1244,1244,1243,1243,1242,1242,1240,1240,1239,1239,1238,1238,1237,1237,1236,1236,1235,1235,1234,1234,1233,1233,1232,1232,1231,1231,1230,1230,1229,1229,1228,1228,1227,1227,1226,1226,1225,1225,1224,1224,1223,1223,1222,1222,1221,1221,1220,1220,1219,1219,1218,1218,1217,1217,1216,1216,1215,1215,1214,1214,1213,1213,1212,1212,1211,1211,1210,1210,1209,1209,1208,1208,1207,1207,1206,1206,1205,1205,1204,1204,1201,1201,1200,1200,1199,1199,1198,1198,1197,1197,1196,1196,1195,1195,1194,1194,1193,1193,1192,1192,1191,1191,1190,1190,1189,1189,1188,1188,1186,1186,1185,1185,1184,1184,1183,1183,1182,1182,1181,1181,1180,1180,1179,1179,1178,1178,1177,1177,1176,1176,1175,1175,1174,1174,1173,1173,1172,1172,1170,1170,1168,1168,1167,1167,1166,1166,1165,1165,1164,1164,1163,1163,1162,1162,1161,1161,1160,1160,1159,1159,1158,1158,1157,1157,1155,1155,1154,1154,1153,1153,1152,1152,1151,1151,1150,1150,1149,1149,1148,1148,1147,1147,1146,1146,1145,1145,1144,1144,1143,1143,1142,1142,1141,1141,1140,1140,1139,1139,1136,1136,1135,1135,1134,1134,1133,1133,1131,1131,1130,1130,1129,1129,1128,1128,1127,1127,1126,1126,1125,1125,1124,1124,1123,1123,1122,1122,1121,1121,1120,1120,1119,1119,1118,1118,1117,1117,1116,1116,1114,1114,1113,1113,1112,1112,1111,1111,1110,1110,1109,1109,1108,1108,1107,1107,1106,1106,1105,1105,1104,1104,1103,1103,1102,1102,1101,1101,1100,1100,1099,1099,1098,1098,1097,1097,1096,1096,1095,1095,1094,1094,1093,1093,1092,1092,1091,1091,1090,1090,1089,1089,1087,1087,1086,1086,1085,1085,1084,1084,1083,1083,1082,1082,1081,1081,1080,1080,1079,1079,1078,1078,1077,1077,1076,1076,1075,1075,1074,1074,1073,1073,1072,1072,1071,1071,1070,1070,1069,1069,1068,1068,1067,1067,1064,1064,1063,1063,1062,1062,1060,1060,1059,1059,1058,1058,1057,1057,1056,1056,1055,1055,1054,1054,1053,1053,1052,1052,1051,1051,1050,1050,1049,1049,1048,1048,1047,1047,1046,1046,1045,1045,1044,1044,1043,1043,1042,1042,1041,1041,1040,1040,1039,1039,1038,1038,1037,1037,1036,1036,1035,1035,1034,1034,1033,1033,1032,1032,1031,1031,1030,1030,1029,1029,1028,1028,1027,1027,1026,1026,1025,1025,1024,1024,1023,1023,1022,1022,1021,1021,1020,1020,1019,1019,1018,1018,1017,1017,1016,1016,1015,1015,1014,1014,1013,1013,1012,1012,1011,1011,1010,1010,1009,1009,1008,1008,1007,1007,1006,1006,1005,1005,1004,1004,1003,1003,1002,1002,1001,1001,1000,1000,999,999,998,998,997,997,996,996,995,995,994,994,993,993,992,992,991,991,990,990,989,989,988,988,987,987,986,986,985,985,984,984,982,982,981,981,980,980,979,979,978,978,977,977,976,976,975,975,974,974,973,973,972,972,971,971,970,970,969,969,968,968,967,967,966,966,965,965,964,964,963,963,962,962,961,961,960,960,959,959,958,958,957,957,956,956,955,955,954,954,953,953,952,952,951,951,950,950,949,949,948,948,947,947,946,946,945,945,944,944,943,943,942,942,940,940,938,938,937,937,936,936,935,935,934,934,933,933,932,932,931,931,930,930,929,929,928,928,927,927,926,926,925,925,924,924,923,923,922,922,921,921,919,919,918,918,917,917,916,916,915,915,914,914,913,913,912,912,911,911,910,910,909,909,908,908,907,907,906,906,905,905,904,904,903,903,902,902,901,901,900,900,898,898,895,895,894,894,893,893,892,892,891,891,890,890,889,889,888,888,887,887,886,886,885,885,883,883,881,881,880,880,879,879,878,878,877,877,876,876,874,874,873,873,872,872,871,871,870,870,869,869,868,868,867,867,866,866,864,864,863,863,862,862,861,861,859,859,858,858,857,857,856,856,855,855,854,854,853,853,852,852,851,851,849,849,848,848,847,847,846,846,845,845,844,844,843,843,842,842,841,841,840,840,838,838,837,837,836,836,835,835,834,834,833,833,832,832,831,831,830,830,829,829,828,828,827,827,826,826,823,823,822,822,821,821,820,820,819,819,818,818,817,817,816,816,815,815,814,814,813,813,812,812,811,811,810,810,809,809,808,808,807,807,806,806,804,804,803,803,802,802,801,801,800,800,799,799,798,798,797,797,795,795,794,794,793,793,792,792,791,791,790,790,789,789,787,787,786,786,785,785,784,784,783,783,782,782,781,781,780,780,779,779,778,778,777,777,776,776,775,775,774,774,773,773,772,772,771,771,770,770,768,768,767,767,766,766,765,765,764,764,763,763,762,762,761,761,760,760,759,759,758,758,757,757,756,756,755,755,754,754,753,753,752,752,750,750,749,749,748,748,747,747,746,746,745,745,744,744,743,743,742,742,741,741,740,740,739,739,738,738,737,737,736,736,735,735,734,734,733,733,732,732,731,731,730,730,729,729,728,728,727,727,726,726,725,725,724,724,723,723,721,721,720,720,719,719,718,718,717,717,716,716,715,715,714,714,713,713,712,712,711,711,710,710,709,709,708,708,707,707,706,706,705,705,704,704,703,703,702,702,701,701,700,700,699,699,698,698,697,697,696,696,695,695,694,694,693,693,691,691,690,690,689,689,688,688,687,687,686,686,685,685,684,684,683,683,682,682,681,681,680,680,679,679,678,678,677,677,676,676,675,675,674,674,673,673,672,672,671,671,670,670,669,669,668,668,667,667,666,666,665,665,664,664,663,663,662,662,661,661,660,660,659,659,658,658,657,657,656,656,655,655,654,654,653,653,652,652,651,651,650,650,649,649,648,648,647,647,646,646,645,645,644,644,643,643,642,642,641,641,640,640,638,638,637,637,635,635,634,634,633,633,632,632,631,631,630,630,629,629,628,628,627,627,626,626,625,625,623,623,622,622,621,621,620,620,619,619,618,618,617,617,616,616,615,615,614,614,613,613,612,612,611,611,610,610,609,609,608,608,607,607,606,606,605,605,604,604,603,603,602,602,601,601,600,600,599,599,597,597,596,596,595,595,594,594,593,593,592,592,591,591,590,590,589,589,588,588,587,587,586,586,585,585,584,584,583,583,582,582,581,581,580,580,579,579,578,578,577,577,575,575,574,574,573,573,572,572,571,571,570,570,569,569,568,568,567,567,566,566,565,565,564,564,563,563,562,562,561,561,560,560,559,559,558,558,557,557,556,556,555,555,553,553,552,552,551,551,550,550,549,549,548,548,547,547,546,546,545,545,544,544,543,543,542,542,541,541,540,540,539,539,538,538,537,537,536,536,535,535,534,534,533,533,532,532,531,531,530,530,529,529,528,528,527,527,526,526,525,525,524,524,523,523,522,522,521,521,520,520,519,519,518,518,517,517,516,516,515,515,514,514,513,513,512,512,511,511,510,510,509,509,508,508,507,507,506,506,505,505,504,504,503,503,502,502,501,501,500,500,499,499,498,498,497,497,496,496,495,495,494,494,493,493,492,492,491,491,490,490,489,489,488,488,487,487,486,486,485,485,484,484,483,483,482,482,481,481,480,480,479,479,478,478,477,477,476,476,475,475,474,474,473,473,472,472,471,471,470,470,469,469,468,468,467,467,466,466,465,465,464,464,463,463,462,462,461,461,460,460,459,459,458,458,457,457,456,456,455,455,454,454,453,453,452,452,451,451,450,450,448,448,447,447,446,446,445,445,444,444,443,443,442,442,441,441,440,440,439,439,438,438,437,437,436,436,435,435,434,434,433,433,432,432,431,431,430,430,429,429,428,428,427,427,426,426,425,425,424,424,423,423,422,422,421,421,420,420,419,419,418,418,417,417,416,416,415,415,414,414,413,413,412,412,411,411,410,410,409,409,408,408,407,407,406,406,405,405,404,404,403,403,402,402,401,401,400,400,0.0],[85407,85407,85407,82898,82898,67830,67830,64715,64715,62382,62382,57426,57426,55669,55669,55648,55648,54736,54736,54279,54279,54161,54161,53673,53673,52923,52923,49062,49062,48901,48901,48671,48671,47264,47264,47074,47074,46869,46869,46446,46446,46133,46133,45258,45258,44854,44854,44815,44815,44148,44148,41164,41164,40974,40974,40868,40868,40800,40800,40369,40369,40246,40246,40190,40190,40162,40162,40147,40147,39357,39357,39344,39344,39036,39036,38966,38966,38765,38765,38416,38416,38379,38379,37476,37476,37475,37475,37179,37179,36970,36970,36283,36283,35380,35380,34775,34775,34773,34773,33742,33742,33676,33676,33517,33517,33435,33435,33166,33166,33006,33006,32992,32992,32961,32961,32959,32959,32843,32843,32511,32511,32493,32493,32253,32253,32159,32159,31959,31959,31499,31499,31316,31316,31308,31308,31137,31137,31072,31072,30861,30861,30824,30824,30725,30725,30644,30644,30489,30489,30334,30334,30301,30301,30097,30097,30014,30014,29782,29782,29422,29422,29232,29232,29161,29161,28947,28947,28840,28840,28428,28428,28244,28244,28241,28241,28156,28156,28149,28149,28006,28006,27770,27770,27746,27746,27599,27599,27345,27345,27280,27280,26998,26998,26972,26972,26930,26930,26770,26770,26723,26723,26718,26718,26561,26561,26552,26552,26526,26526,26322,26322,26005,26005,25882,25882,25867,25867,25843,25843,25707,25707,25582,25582,25391,25391,25360,25360,25289,25289,25128,25128,25125,25125,25079,25079,24987,24987,24976,24976,24856,24856,24685,24685,24519,24519,24350,24350,24328,24328,24325,24325,24259,24259,24033,24033,23885,23885,23813,23813,23612,23612,23562,23562,23351,23351,23308,23308,23296,23296,23266,23266,23176,23176,23126,23126,23081,23081,23074,23074,23007,23007,22940,22940,22736,22736,22694,22694,22662,22662,22578,22578,22504,22504,22316,22316,22278,22278,22054,22054,22051,22051,22025,22025,21992,21992,21968,21968,21935,21935,21927,21927,21867,21867,21852,21852,21736,21736,21713,21713,21672,21672,21601,21601,21478,21478,21462,21462,21461,21461,21432,21432,21378,21378,21277,21277,21254,21254,21203,21203,21147,21147,21132,21132,21109,21109,20925,20925,20900,20900,20871,20871,20776,20776,20594,20594,20529,20529,20448,20448,20301,20301,20236,20236,20075,20075,20030,20030,19985,19985,19830,19830,19781,19781,19779,19779,19765,19765,19756,19756,19670,19670,19646,19646,19636,19636,19620,19620,19506,19506,19346,19346,19321,19321,19261,19261,19189,19189,19122,19122,18990,18990,18952,18952,18944,18944,18927,18927,18912,18912,18886,18886,18799,18799,18794,18794,18744,18744,18671,18671,18532,18532,18528,18528,18505,18505,18475,18475,18464,18464,18462,18462,18377,18377,18365,18365,18334,18334,18264,18264,18056,18056,18013,18013,17963,17963,17938,17938,17921,17921,17886,17886,17867,17867,17761,17761,17707,17707,17632,17632,17631,17631,17611,17611,17589,17589,17481,17481,17461,17461,17430,17430,17427,17427,17421,17421,17358,17358,17299,17299,17273,17273,17232,17232,17199,17199,17150,17150,17144,17144,17121,17121,17071,17071,17012,17012,17007,17007,16995,16995,16928,16928,16906,16906,16799,16799,16789,16789,16754,16754,16687,16687,16658,16658,16616,16616,16538,16538,16520,16520,16465,16465,16450,16450,16324,16324,16319,16319,16305,16305,16300,16300,16264,16264,16235,16235,16225,16225,16216,16216,16194,16194,16192,16192,16184,16184,16135,16135,16075,16075,16021,16021,15996,15996,15978,15978,15975,15975,15948,15948,15914,15914,15882,15882,15881,15881,15875,15875,15872,15872,15815,15815,15794,15794,15776,15776,15775,15775,15771,15771,15747,15747,15734,15734,15709,15709,15689,15689,15603,15603,15588,15588,15582,15582,15559,15559,15545,15545,15498,15498,15480,15480,15473,15473,15415,15415,15409,15409,15363,15363,15250,15250,15244,15244,15166,15166,15153,15153,15142,15142,15132,15132,15125,15125,15124,15124,15120,15120,15109,15109,15106,15106,15096,15096,15081,15081,15069,15069,15064,15064,15027,15027,15018,15018,15009,15009,15005,15005,14997,14997,14987,14987,14980,14980,14960,14960,14952,14952,14949,14949,14933,14933,14909,14909,14878,14878,14860,14860,14815,14815,14811,14811,14794,14794,14792,14792,14768,14768,14758,14758,14757,14757,14699,14699,14689,14689,14629,14629,14628,14628,14626,14626,14582,14582,14566,14566,14536,14536,14468,14468,14461,14461,14453,14453,14431,14431,14414,14414,14366,14366,14308,14308,14288,14288,14267,14267,14257,14257,14206,14206,14203,14203,14202,14202,14158,14158,14137,14137,14117,14117,14043,14043,14002,14002,13894,13894,13888,13888,13880,13880,13870,13870,13863,13863,13824,13824,13794,13794,13777,13777,13769,13769,13754,13754,13740,13740,13730,13730,13643,13643,13639,13639,13614,13614,13594,13594,13556,13556,13554,13554,13544,13544,13542,13542,13528,13528,13484,13484,13483,13483,13474,13474,13455,13455,13440,13440,13424,13424,13370,13370,13358,13358,13339,13339,13336,13336,13327,13327,13314,13314,13258,13258,13256,13256,13244,13244,13182,13182,13146,13146,13137,13137,13109,13109,13102,13102,13089,13089,13080,13080,13043,13043,13034,13034,13030,13030,13025,13025,12998,12998,12979,12979,12951,12951,12947,12947,12941,12941,12898,12898,12890,12890,12886,12886,12882,12882,12879,12879,12862,12862,12861,12861,12836,12836,12835,12835,12813,12813,12784,12784,12780,12780,12778,12778,12744,12744,12711,12711,12692,12692,12687,12687,12680,12680,12655,12655,12640,12640,12612,12612,12610,12610,12606,12606,12602,12602,12601,12601,12554,12554,12529,12529,12509,12509,12498,12498,12497,12497,12488,12488,12468,12468,12455,12455,12435,12435,12430,12430,12404,12404,12403,12403,12397,12397,12394,12394,12392,12392,12381,12381,12346,12346,12338,12338,12331,12331,12290,12290,12287,12287,12258,12258,12234,12234,12233,12233,12229,12229,12225,12225,12197,12197,12187,12187,12184,12184,12182,12182,12171,12171,12084,12084,12075,12075,12070,12070,12034,12034,12009,12009,11970,11970,11963,11963,11952,11952,11925,11925,11922,11922,11886,11886,11863,11863,11857,11857,11844,11844,11839,11839,11825,11825,11805,11805,11794,11794,11787,11787,11782,11782,11761,11761,11734,11734,11714,11714,11682,11682,11676,11676,11670,11670,11652,11652,11622,11622,11614,11614,11603,11603,11594,11594,11591,11591,11555,11555,11541,11541,11532,11532,11515,11515,11508,11508,11481,11481,11462,11462,11461,11461,11447,11447,11443,11443,11435,11435,11429,11429,11427,11427,11413,11413,11397,11397,11395,11395,11383,11383,11380,11380,11373,11373,11345,11345,11330,11330,11310,11310,11262,11262,11258,11258,11246,11246,11245,11245,11240,11240,11213,11213,11194,11194,11192,11192,11175,11175,11161,11161,11137,11137,11130,11130,11115,11115,11111,11111,11101,11101,11094,11094,11054,11054,11032,11032,11029,11029,11002,11002,10978,10978,10973,10973,10955,10955,10952,10952,10926,10926,10909,10909,10906,10906,10905,10905,10895,10895,10855,10855,10852,10852,10820,10820,10816,10816,10792,10792,10777,10777,10763,10763,10740,10740,10679,10679,10669,10669,10667,10667,10656,10656,10637,10637,10634,10634,10630,10630,10597,10597,10591,10591,10578,10578,10545,10545,10506,10506,10505,10505,10490,10490,10484,10484,10470,10470,10464,10464,10447,10447,10444,10444,10442,10442,10437,10437,10429,10429,10428,10428,10426,10426,10421,10421,10420,10420,10412,10412,10411,10411,10405,10405,10404,10404,10394,10394,10391,10391,10363,10363,10359,10359,10350,10350,10346,10346,10335,10335,10324,10324,10314,10314,10305,10305,10296,10296,10294,10294,10291,10291,10283,10283,10278,10278,10253,10253,10234,10234,10224,10224,10223,10223,10210,10210,10208,10208,10205,10205,10198,10198,10193,10193,10173,10173,10159,10159,10126,10126,10122,10122,10118,10118,10112,10112,10111,10111,10089,10089,10072,10072,10063,10063,10051,10051,10050,10050,10047,10047,10023,10023,10006,10006,10001,10001,9991,9991,9969,9969,9935,9935,9921,9921,9916,9916,9914,9914,9908,9908,9904,9904,9900,9900,9898,9898,9865,9865,9863,9863,9852,9852,9846,9846,9832,9832,9827,9827,9820,9820,9817,9817,9800,9800,9791,9791,9751,9751,9744,9744,9740,9740,9735,9735,9728,9728,9725,9725,9712,9712,9704,9704,9701,9701,9697,9697,9695,9695,9678,9678,9675,9675,9674,9674,9671,9671,9668,9668,9667,9667,9665,9665,9662,9662,9638,9638,9635,9635,9627,9627,9611,9611,9595,9595,9592,9592,9590,9590,9559,9559,9553,9553,9549,9549,9548,9548,9544,9544,9522,9522,9515,9515,9512,9512,9507,9507,9502,9502,9491,9491,9485,9485,9484,9484,9478,9478,9462,9462,9458,9458,9436,9436,9429,9429,9422,9422,9393,9393,9375,9375,9365,9365,9355,9355,9348,9348,9344,9344,9342,9342,9329,9329,9327,9327,9321,9321,9319,9319,9316,9316,9315,9315,9284,9284,9271,9271,9266,9266,9258,9258,9250,9250,9249,9249,9229,9229,9221,9221,9206,9206,9199,9199,9195,9195,9167,9167,9163,9163,9155,9155,9154,9154,9147,9147,9096,9096,9087,9087,9085,9085,9082,9082,9072,9072,9067,9067,9057,9057,9049,9049,9046,9046,9045,9045,9043,9043,9037,9037,9036,9036,9033,9033,9016,9016,8950,8950,8946,8946,8911,8911,8900,8900,8896,8896,8895,8895,8878,8878,8871,8871,8851,8851,8850,8850,8846,8846,8827,8827,8815,8815,8803,8803,8801,8801,8796,8796,8784,8784,8783,8783,8769,8769,8767,8767,8745,8745,8739,8739,8737,8737,8718,8718,8715,8715,8713,8713,8706,8706,8697,8697,8692,8692,8677,8677,8662,8662,8659,8659,8656,8656,8653,8653,8648,8648,8632,8632,8625,8625,8612,8612,8608,8608,8594,8594,8584,8584,8578,8578,8569,8569,8554,8554,8549,8549,8543,8543,8537,8537,8532,8532,8522,8522,8521,8521,8514,8514,8492,8492,8490,8490,8489,8489,8477,8477,8466,8466,8450,8450,8447,8447,8445,8445,8440,8440,8434,8434,8425,8425,8421,8421,8418,8418,8411,8411,8409,8409,8408,8408,8402,8402,8400,8400,8390,8390,8385,8385,8367,8367,8350,8350,8343,8343,8338,8338,8332,8332,8326,8326,8322,8322,8313,8313,8304,8304,8295,8295,8279,8279,8274,8274,8251,8251,8234,8234,8226,8226,8224,8224,8221,8221,8212,8212,8207,8207,8206,8206,8203,8203,8201,8201,8199,8199,8179,8179,8178,8178,8176,8176,8164,8164,8159,8159,8158,8158,8147,8147,8140,8140,8137,8137,8132,8132,8130,8130,8123,8123,8120,8120,8105,8105,8102,8102,8095,8095,8088,8088,8085,8085,8084,8084,8082,8082,8064,8064,8061,8061,8045,8045,8043,8043,8038,8038,8036,8036,8028,8028,8018,8018,8006,8006,8000,8000,7997,7997,7993,7993,7989,7989,7985,7985,7980,7980,7949,7949,7927,7927,7923,7923,7915,7915,7914,7914,7913,7913,7907,7907,7899,7899,7881,7881,7875,7875,7873,7873,7864,7864,7858,7858,7842,7842,7836,7836,7827,7827,7819,7819,7803,7803,7802,7802,7797,7797,7774,7774,7765,7765,7764,7764,7756,7756,7753,7753,7741,7741,7737,7737,7722,7722,7716,7716,7698,7698,7686,7686,7684,7684,7682,7682,7664,7664,7637,7637,7634,7634,7624,7624,7623,7623,7619,7619,7604,7604,7603,7603,7594,7594,7582,7582,7575,7575,7568,7568,7566,7566,7564,7564,7554,7554,7518,7518,7517,7517,7513,7513,7512,7512,7508,7508,7507,7507,7489,7489,7483,7483,7477,7477,7469,7469,7467,7467,7463,7463,7430,7430,7429,7429,7418,7418,7417,7417,7413,7413,7412,7412,7404,7404,7402,7402,7398,7398,7391,7391,7386,7386,7381,7381,7376,7376,7368,7368,7366,7366,7363,7363,7354,7354,7346,7346,7343,7343,7340,7340,7339,7339,7317,7317,7297,7297,7289,7289,7288,7288,7277,7277,7259,7259,7237,7237,7231,7231,7230,7230,7227,7227,7225,7225,7215,7215,7207,7207,7206,7206,7205,7205,7192,7192,7185,7185,7184,7184,7180,7180,7175,7175,7154,7154,7149,7149,7131,7131,7126,7126,7108,7108,7089,7089,7083,7083,7082,7082,7076,7076,7066,7066,7059,7059,7055,7055,7052,7052,7050,7050,7047,7047,7039,7039,7038,7038,7037,7037,7034,7034,7031,7031,7010,7010,7005,7005,7003,7003,7002,7002,7000,7000,6985,6985,6982,6982,6981,6981,6978,6978,6965,6965,6955,6955,6952,6952,6946,6946,6942,6942,6930,6930,6929,6929,6922,6922,6913,6913,6908,6908,6907,6907,6906,6906,6894,6894,6881,6881,6876,6876,6874,6874,6863,6863,6861,6861,6860,6860,6854,6854,6846,6846,6842,6842,6828,6828,6825,6825,6814,6814,6810,6810,6800,6800,6798,6798,6797,6797,6795,6795,6794,6794,6790,6790,6787,6787,6786,6786,6782,6782,6772,6772,6755,6755,6750,6750,6743,6743,6740,6740,6735,6735,6734,6734,6732,6732,6724,6724,6711,6711,6710,6710,6709,6709,6708,6708,6704,6704,6700,6700,6694,6694,6693,6693,6692,6692,6683,6683,6671,6671,6669,6669,6668,6668,6663,6663,6660,6660,6659,6659,6645,6645,6641,6641,6634,6634,6625,6625,6618,6618,6615,6615,6612,6612,6611,6611,6605,6605,6599,6599,6597,6597,6593,6593,6587,6587,6584,6584,6577,6577,6568,6568,6566,6566,6562,6562,6553,6553,6550,6550,6547,6547,6537,6537,6536,6536,6532,6532,6529,6529,6521,6521,6520,6520,6502,6502,6492,6492,6485,6485,6483,6483,6478,6478,6477,6477,6467,6467,6461,6461,6458,6458,6449,6449,6448,6448,6439,6439,6438,6438,6431,6431,6427,6427,6425,6425,6419,6419,6402,6402,6401,6401,6396,6396,6395,6395,6392,6392,6390,6390,6383,6383,6378,6378,6375,6375,6361,6361,6355,6355,6353,6353,6351,6351,6350,6350,6349,6349,6343,6343,6342,6342,6338,6338,6331,6331,6329,6329,6322,6322,6317,6317,6313,6313,6310,6310,6305,6305,6296,6296,6295,6295,6290,6290,6287,6287,6277,6277,6271,6271,6265,6265,6260,6260,6259,6259,6257,6257,6243,6243,6241,6241,6231,6231,6182,6182,6180,6180,6178,6178,6175,6175,6169,6169,6168,6168,6162,6162,6158,6158,6153,6153,6148,6148,6145,6145,6144,6144,6141,6141,6140,6140,6138,6138,6133,6133,6132,6132,6131,6131,6126,6126,6123,6123,6121,6121,6119,6119,6114,6114,6103,6103,6102,6102,6099,6099,6097,6097,6087,6087,6085,6085,6082,6082,6077,6077,6073,6073,6053,6053,6052,6052,6051,6051,6034,6034,6031,6031,6030,6030,6029,6029,6028,6028,6025,6025,6021,6021,6017,6017,6014,6014,6009,6009,6004,6004,5991,5991,5988,5988,5983,5983,5981,5981,5980,5980,5976,5976,5972,5972,5967,5967,5964,5964,5963,5963,5962,5962,5961,5961,5956,5956,5954,5954,5950,5950,5947,5947,5945,5945,5944,5944,5942,5942,5941,5941,5938,5938,5937,5937,5936,5936,5933,5933,5928,5928,5923,5923,5914,5914,5913,5913,5910,5910,5904,5904,5903,5903,5902,5902,5891,5891,5890,5890,5886,5886,5885,5885,5876,5876,5870,5870,5866,5866,5859,5859,5853,5853,5852,5852,5851,5851,5850,5850,5848,5848,5840,5840,5838,5838,5836,5836,5834,5834,5832,5832,5829,5829,5827,5827,5826,5826,5825,5825,5821,5821,5816,5816,5807,5807,5801,5801,5798,5798,5792,5792,5790,5790,5788,5788,5773,5773,5768,5768,5761,5761,5760,5760,5759,5759,5757,5757,5756,5756,5752,5752,5746,5746,5742,5742,5737,5737,5728,5728,5725,5725,5720,5720,5718,5718,5716,5716,5714,5714,5712,5712,5710,5710,5709,5709,5708,5708,5707,5707,5702,5702,5699,5699,5698,5698,5690,5690,5687,5687,5681,5681,5678,5678,5676,5676,5673,5673,5669,5669,5666,5666,5665,5665,5660,5660,5659,5659,5655,5655,5653,5653,5642,5642,5634,5634,5625,5625,5623,5623,5620,5620,5618,5618,5616,5616,5615,5615,5612,5612,5602,5602,5596,5596,5592,5592,5587,5587,5584,5584,5581,5581,5580,5580,5576,5576,5573,5573,5570,5570,5563,5563,5560,5560,5559,5559,5558,5558,5549,5549,5546,5546,5544,5544,5543,5543,5541,5541,5539,5539,5535,5535,5532,5532,5531,5531,5528,5528,5522,5522,5520,5520,5514,5514,5506,5506,5499,5499,5497,5497,5496,5496,5494,5494,5493,5493,5491,5491,5487,5487,5485,5485,5482,5482,5478,5478,5473,5473,5472,5472,5469,5469,5465,5465,5464,5464,5463,5463,5458,5458,5453,5453,5451,5451,5445,5445,5437,5437,5435,5435,5423,5423,5422,5422,5421,5421,5419,5419,5418,5418,5410,5410,5404,5404,5403,5403,5398,5398,5395,5395,5394,5394,5391,5391,5388,5388,5387,5387,5381,5381,5378,5378,5371,5371,5370,5370,5367,5367,5366,5366,5365,5365,5363,5363,5358,5358,5356,5356,5355,5355,5349,5349,5346,5346,5345,5345,5344,5344,5342,5342,5338,5338,5335,5335,5330,5330,5329,5329,5328,5328,5327,5327,5321,5321,5320,5320,5316,5316,5314,5314,5310,5310,5308,5308,5307,5307,5299,5299,5296,5296,5293,5293,5292,5292,5291,5291,5286,5286,5276,5276,5274,5274,5272,5272,5269,5269,5268,5268,5264,5264,5262,5262,5254,5254,5251,5251,5250,5250,5247,5247,5246,5246,5234,5234,5233,5233,5232,5232,5229,5229,5227,5227,5216,5216,5210,5210,5206,5206,5196,5196,5191,5191,5189,5189,5187,5187,5185,5185,5184,5184,5183,5183,5180,5180,5175,5175,5173,5173,5171,5171,5169,5169,5167,5167,5166,5166,5162,5162,5158,5158,5153,5153,5150,5150,5147,5147,5142,5142,5138,5138,5136,5136,5132,5132,5131,5131,5130,5130,5129,5129,5128,5128,5120,5120,5117,5117,5116,5116,5112,5112,5107,5107,5105,5105,5102,5102,5100,5100,5090,5090,5088,5088,5083,5083,5081,5081,5080,5080,5074,5074,5072,5072,5071,5071,5070,5070,5067,5067,5066,5066,5064,5064,5063,5063,5060,5060,5058,5058,5057,5057,5054,5054,5050,5050,5045,5045,5043,5043,5041,5041,5040,5040,5039,5039,5036,5036,5034,5034,5033,5033,5032,5032,5031,5031,5029,5029,5028,5028,5024,5024,5015,5015,5012,5012,5011,5011,5005,5005,5004,5004,5003,5003,5001,5001,5000,5000,4999,4999,4997,4997,4987,4987,4984,4984,4977,4977,4976,4976,4973,4973,4968,4968,4960,4960,4957,4957,4956,4956,4953,4953,4951,4951,4950,4950,4949,4949,4945,4945,4944,4944,4941,4941,4940,4940,4938,4938,4936,4936,4928,4928,4921,4921,4920,4920,4915,4915,4907,4907,4901,4901,4899,4899,4898,4898,4897,4897,4896,4896,4895,4895,4894,4894,4893,4893,4892,4892,4890,4890,4886,4886,4885,4885,4883,4883,4882,4882,4880,4880,4873,4873,4871,4871,4870,4870,4867,4867,4866,4866,4847,4847,4844,4844,4839,4839,4834,4834,4833,4833,4831,4831,4829,4829,4828,4828,4822,4822,4821,4821,4820,4820,4816,4816,4814,4814,4813,4813,4812,4812,4805,4805,4804,4804,4802,4802,4800,4800,4799,4799,4795,4795,4794,4794,4790,4790,4788,4788,4787,4787,4783,4783,4780,4780,4766,4766,4764,4764,4759,4759,4753,4753,4751,4751,4744,4744,4739,4739,4738,4738,4736,4736,4734,4734,4733,4733,4729,4729,4726,4726,4725,4725,4722,4722,4720,4720,4713,4713,4711,4711,4710,4710,4705,4705,4702,4702,4701,4701,4700,4700,4698,4698,4691,4691,4689,4689,4685,4685,4681,4681,4678,4678,4674,4674,4670,4670,4669,4669,4663,4663,4661,4661,4658,4658,4656,4656,4645,4645,4643,4643,4642,4642,4637,4637,4635,4635,4630,4630,4629,4629,4627,4627,4624,4624,4613,4613,4612,4612,4611,4611,4610,4610,4609,4609,4602,4602,4601,4601,4600,4600,4590,4590,4589,4589,4587,4587,4582,4582,4578,4578,4576,4576,4573,4573,4561,4561,4558,4558,4553,4553,4552,4552,4551,4551,4550,4550,4549,4549,4547,4547,4546,4546,4545,4545,4541,4541,4540,4540,4539,4539,4536,4536,4533,4533,4531,4531,4524,4524,4522,4522,4521,4521,4520,4520,4514,4514,4512,4512,4511,4511,4509,4509,4499,4499,4491,4491,4489,4489,4488,4488,4487,4487,4486,4486,4485,4485,4474,4474,4473,4473,4470,4470,4468,4468,4463,4463,4454,4454,4450,4450,4447,4447,4445,4445,4443,4443,4437,4437,4436,4436,4434,4434,4431,4431,4427,4427,4425,4425,4424,4424,4422,4422,4418,4418,4417,4417,4415,4415,4412,4412,4410,4410,4401,4401,4399,4399,4398,4398,4397,4397,4393,4393,4388,4388,4385,4385,4380,4380,4379,4379,4378,4378,4376,4376,4373,4373,4372,4372,4367,4367,4366,4366,4364,4364,4362,4362,4352,4352,4347,4347,4346,4346,4342,4342,4340,4340,4337,4337,4334,4334,4333,4333,4330,4330,4328,4328,4317,4317,4314,4314,4313,4313,4312,4312,4308,4308,4302,4302,4296,4296,4288,4288,4285,4285,4282,4282,4281,4281,4279,4279,4276,4276,4275,4275,4273,4273,4272,4272,4268,4268,4265,4265,4255,4255,4251,4251,4243,4243,4242,4242,4240,4240,4237,4237,4236,4236,4235,4235,4234,4234,4233,4233,4231,4231,4229,4229,4226,4226,4225,4225,4222,4222,4221,4221,4219,4219,4217,4217,4211,4211,4209,4209,4205,4205,4199,4199,4193,4193,4192,4192,4190,4190,4189,4189,4188,4188,4187,4187,4186,4186,4185,4185,4183,4183,4181,4181,4180,4180,4177,4177,4175,4175,4174,4174,4172,4172,4171,4171,4169,4169,4160,4160,4159,4159,4156,4156,4155,4155,4152,4152,4145,4145,4136,4136,4135,4135,4130,4130,4128,4128,4127,4127,4126,4126,4125,4125,4124,4124,4121,4121,4120,4120,4118,4118,4116,4116,4115,4115,4114,4114,4113,4113,4110,4110,4108,4108,4107,4107,4104,4104,4103,4103,4099,4099,4098,4098,4095,4095,4079,4079,4078,4078,4076,4076,4072,4072,4070,4070,4069,4069,4067,4067,4063,4063,4062,4062,4060,4060,4056,4056,4055,4055,4054,4054,4051,4051,4049,4049,4044,4044,4043,4043,4033,4033,4031,4031,4030,4030,4023,4023,4017,4017,4007,4007,4004,4004,4001,4001,3999,3999,3998,3998,3991,3991,3989,3989,3980,3980,3978,3978,3977,3977,3975,3975,3970,3970,3969,3969,3964,3964,3958,3958,3956,3956,3955,3955,3950,3950,3949,3949,3947,3947,3944,3944,3942,3942,3941,3941,3940,3940,3936,3936,3935,3935,3934,3934,3931,3931,3930,3930,3929,3929,3926,3926,3925,3925,3924,3924,3923,3923,3906,3906,3902,3902,3900,3900,3896,3896,3894,3894,3893,3893,3891,3891,3888,3888,3887,3887,3886,3886,3885,3885,3883,3883,3878,3878,3875,3875,3873,3873,3871,3871,3869,3869,3867,3867,3865,3865,3864,3864,3860,3860,3859,3859,3858,3858,3856,3856,3855,3855,3853,3853,3850,3850,3849,3849,3848,3848,3843,3843,3841,3841,3838,3838,3836,3836,3835,3835,3832,3832,3830,3830,3827,3827,3824,3824,3822,3822,3818,3818,3817,3817,3805,3805,3790,3790,3788,3788,3781,3781,3774,3774,3769,3769,3766,3766,3759,3759,3758,3758,3757,3757,3754,3754,3748,3748,3746,3746,3743,3743,3742,3742,3740,3740,3739,3739,3736,3736,3732,3732,3729,3729,3724,3724,3721,3721,3720,3720,3719,3719,3717,3717,3716,3716,3714,3714,3713,3713,3709,3709,3707,3707,3703,3703,3700,3700,3699,3699,3698,3698,3696,3696,3695,3695,3693,3693,3692,3692,3691,3691,3689,3689,3685,3685,3683,3683,3682,3682,3678,3678,3677,3677,3673,3673,3665,3665,3664,3664,3660,3660,3659,3659,3657,3657,3656,3656,3655,3655,3654,3654,3653,3653,3652,3652,3651,3651,3649,3649,3647,3647,3644,3644,3643,3643,3642,3642,3639,3639,3635,3635,3633,3633,3629,3629,3628,3628,3625,3625,3621,3621,3619,3619,3615,3615,3612,3612,3611,3611,3609,3609,3607,3607,3604,3604,3599,3599,3597,3597,3595,3595,3594,3594,3593,3593,3589,3589,3587,3587,3579,3579,3578,3578,3577,3577,3576,3576,3575,3575,3573,3573,3572,3572,3571,3571,3569,3569,3565,3565,3564,3564,3561,3561,3560,3560,3559,3559,3558,3558,3557,3557,3551,3551,3549,3549,3544,3544,3543,3543,3541,3541,3538,3538,3531,3531,3527,3527,3526,3526,3518,3518,3515,3515,3513,3513,3512,3512,3506,3506,3505,3505,3501,3501,3500,3500,3497,3497,3496,3496,3495,3495,3492,3492,3490,3490,3489,3489,3480,3480,3472,3472,3471,3471,3470,3470,3468,3468,3467,3467,3466,3466,3465,3465,3460,3460,3458,3458,3456,3456,3455,3455,3453,3453,3452,3452,3449,3449,3448,3448,3443,3443,3442,3442,3441,3441,3440,3440,3436,3436,3431,3431,3426,3426,3425,3425,3424,3424,3422,3422,3420,3420,3418,3418,3413,3413,3412,3412,3410,3410,3408,3408,3407,3407,3406,3406,3402,3402,3401,3401,3397,3397,3396,3396,3394,3394,3393,3393,3385,3385,3378,3378,3376,3376,3375,3375,3374,3374,3370,3370,3369,3369,3364,3364,3363,3363,3360,3360,3359,3359,3356,3356,3355,3355,3354,3354,3352,3352,3346,3346,3345,3345,3344,3344,3341,3341,3335,3335,3334,3334,3330,3330,3327,3327,3325,3325,3322,3322,3321,3321,3320,3320,3317,3317,3316,3316,3314,3314,3313,3313,3312,3312,3311,3311,3310,3310,3308,3308,3307,3307,3301,3301,3300,3300,3299,3299,3297,3297,3291,3291,3288,3288,3286,3286,3281,3281,3279,3279,3275,3275,3274,3274,3263,3263,3262,3262,3260,3260,3253,3253,3252,3252,3251,3251,3250,3250,3249,3249,3248,3248,3245,3245,3244,3244,3240,3240,3238,3238,3235,3235,3233,3233,3232,3232,3231,3231,3230,3230,3225,3225,3223,3223,3222,3222,3218,3218,3217,3217,3216,3216,3213,3213,3212,3212,3211,3211,3210,3210,3208,3208,3205,3205,3200,3200,3196,3196,3193,3193,3192,3192,3190,3190,3188,3188,3187,3187,3186,3186,3184,3184,3183,3183,3181,3181,3180,3180,3179,3179,3176,3176,3170,3170,3168,3168,3166,3166,3165,3165,3164,3164,3160,3160,3158,3158,3155,3155,3150,3150,3149,3149,3148,3148,3147,3147,3145,3145,3143,3143,3141,3141,3138,3138,3136,3136,3134,3134,3133,3133,3125,3125,3121,3121,3120,3120,3115,3115,3114,3114,3113,3113,3112,3112,3111,3111,3107,3107,3106,3106,3104,3104,3101,3101,3099,3099,3097,3097,3095,3095,3093,3093,3092,3092,3091,3091,3090,3090,3086,3086,3085,3085,3084,3084,3083,3083,3082,3082,3081,3081,3077,3077,3076,3076,3074,3074,3073,3073,3068,3068,3067,3067,3066,3066,3065,3065,3062,3062,3055,3055,3053,3053,3052,3052,3050,3050,3046,3046,3043,3043,3042,3042,3039,3039,3036,3036,3031,3031,3028,3028,3027,3027,3026,3026,3025,3025,3024,3024,3022,3022,3020,3020,3019,3019,3018,3018,3017,3017,3016,3016,3008,3008,3007,3007,3004,3004,3001,3001,2998,2998,2995,2995,2989,2989,2988,2988,2986,2986,2985,2985,2984,2984,2983,2983,2980,2980,2979,2979,2976,2976,2974,2974,2973,2973,2969,2969,2968,2968,2966,2966,2963,2963,2962,2962,2958,2958,2957,2957,2953,2953,2946,2946,2945,2945,2940,2940,2939,2939,2938,2938,2937,2937,2933,2933,2930,2930,2929,2929,2924,2924,2923,2923,2920,2920,2911,2911,2908,2908,2906,2906,2905,2905,2904,2904,2903,2903,2899,2899,2898,2898,2897,2897,2893,2893,2892,2892,2890,2890,2889,2889,2886,2886,2885,2885,2883,2883,2882,2882,2881,2881,2880,2880,2879,2879,2878,2878,2877,2877,2875,2875,2873,2873,2872,2872,2869,2869,2867,2867,2866,2866,2865,2865,2864,2864,2862,2862,2861,2861,2860,2860,2859,2859,2858,2858,2857,2857,2852,2852,2851,2851,2850,2850,2847,2847,2846,2846,2845,2845,2843,2843,2840,2840,2839,2839,2833,2833,2832,2832,2831,2831,2830,2830,2828,2828,2824,2824,2822,2822,2820,2820,2819,2819,2818,2818,2816,2816,2815,2815,2814,2814,2812,2812,2811,2811,2810,2810,2807,2807,2806,2806,2805,2805,2801,2801,2798,2798,2795,2795,2791,2791,2790,2790,2789,2789,2788,2788,2786,2786,2785,2785,2783,2783,2782,2782,2781,2781,2778,2778,2777,2777,2775,2775,2774,2774,2772,2772,2771,2771,2769,2769,2768,2768,2767,2767,2765,2765,2764,2764,2761,2761,2760,2760,2759,2759,2758,2758,2756,2756,2754,2754,2752,2752,2748,2748,2746,2746,2744,2744,2742,2742,2737,2737,2732,2732,2731,2731,2729,2729,2723,2723,2722,2722,2721,2721,2716,2716,2715,2715,2713,2713,2710,2710,2709,2709,2706,2706,2705,2705,2703,2703,2701,2701,2700,2700,2697,2697,2696,2696,2694,2694,2693,2693,2692,2692,2691,2691,2690,2690,2689,2689,2687,2687,2686,2686,2684,2684,2683,2683,2681,2681,2679,2679,2678,2678,2677,2677,2676,2676,2674,2674,2673,2673,2672,2672,2671,2671,2670,2670,2668,2668,2667,2667,2666,2666,2664,2664,2663,2663,2662,2662,2661,2661,2658,2658,2657,2657,2656,2656,2655,2655,2654,2654,2653,2653,2652,2652,2646,2646,2644,2644,2643,2643,2642,2642,2638,2638,2635,2635,2633,2633,2630,2630,2628,2628,2626,2626,2624,2624,2620,2620,2617,2617,2616,2616,2610,2610,2609,2609,2605,2605,2604,2604,2603,2603,2600,2600,2599,2599,2598,2598,2597,2597,2596,2596,2595,2595,2594,2594,2593,2593,2591,2591,2588,2588,2585,2585,2584,2584,2583,2583,2581,2581,2578,2578,2577,2577,2575,2575,2574,2574,2573,2573,2572,2572,2569,2569,2568,2568,2565,2565,2562,2562,2560,2560,2558,2558,2557,2557,2556,2556,2555,2555,2554,2554,2552,2552,2551,2551,2549,2549,2548,2548,2546,2546,2545,2545,2544,2544,2543,2543,2542,2542,2541,2541,2539,2539,2538,2538,2536,2536,2533,2533,2532,2532,2531,2531,2530,2530,2528,2528,2527,2527,2520,2520,2519,2519,2518,2518,2513,2513,2512,2512,2510,2510,2505,2505,2504,2504,2503,2503,2502,2502,2501,2501,2496,2496,2495,2495,2494,2494,2493,2493,2492,2492,2491,2491,2489,2489,2488,2488,2486,2486,2485,2485,2484,2484,2483,2483,2478,2478,2477,2477,2476,2476,2472,2472,2468,2468,2465,2465,2464,2464,2462,2462,2461,2461,2459,2459,2458,2458,2457,2457,2455,2455,2452,2452,2450,2450,2448,2448,2446,2446,2442,2442,2441,2441,2440,2440,2433,2433,2432,2432,2431,2431,2430,2430,2425,2425,2424,2424,2421,2421,2418,2418,2413,2413,2411,2411,2409,2409,2406,2406,2405,2405,2404,2404,2402,2402,2401,2401,2399,2399,2398,2398,2397,2397,2396,2396,2395,2395,2394,2394,2393,2393,2392,2392,2390,2390,2389,2389,2388,2388,2387,2387,2386,2386,2384,2384,2383,2383,2382,2382,2381,2381,2379,2379,2376,2376,2374,2374,2372,2372,2371,2371,2370,2370,2368,2368,2367,2367,2363,2363,2361,2361,2358,2358,2357,2357,2355,2355,2354,2354,2352,2352,2351,2351,2349,2349,2348,2348,2346,2346,2345,2345,2344,2344,2343,2343,2342,2342,2340,2340,2339,2339,2338,2338,2337,2337,2336,2336,2333,2333,2331,2331,2330,2330,2329,2329,2328,2328,2327,2327,2325,2325,2322,2322,2321,2321,2320,2320,2319,2319,2318,2318,2316,2316,2314,2314,2313,2313,2312,2312,2311,2311,2310,2310,2309,2309,2308,2308,2307,2307,2304,2304,2302,2302,2301,2301,2300,2300,2299,2299,2298,2298,2295,2295,2294,2294,2287,2287,2286,2286,2283,2283,2282,2282,2281,2281,2280,2280,2279,2279,2278,2278,2277,2277,2275,2275,2274,2274,2272,2272,2271,2271,2270,2270,2268,2268,2267,2267,2266,2266,2265,2265,2264,2264,2262,2262,2260,2260,2259,2259,2258,2258,2257,2257,2256,2256,2252,2252,2251,2251,2250,2250,2249,2249,2247,2247,2246,2246,2243,2243,2240,2240,2239,2239,2238,2238,2237,2237,2236,2236,2235,2235,2233,2233,2232,2232,2231,2231,2230,2230,2228,2228,2227,2227,2226,2226,2224,2224,2222,2222,2221,2221,2220,2220,2218,2218,2216,2216,2214,2214,2213,2213,2212,2212,2211,2211,2209,2209,2208,2208,2205,2205,2202,2202,2201,2201,2200,2200,2199,2199,2198,2198,2197,2197,2196,2196,2195,2195,2194,2194,2192,2192,2191,2191,2190,2190,2188,2188,2187,2187,2185,2185,2183,2183,2182,2182,2181,2181,2179,2179,2175,2175,2174,2174,2173,2173,2172,2172,2169,2169,2164,2164,2163,2163,2162,2162,2161,2161,2160,2160,2159,2159,2158,2158,2157,2157,2152,2152,2151,2151,2148,2148,2146,2146,2145,2145,2143,2143,2142,2142,2140,2140,2139,2139,2138,2138,2137,2137,2135,2135,2134,2134,2132,2132,2131,2131,2129,2129,2127,2127,2126,2126,2124,2124,2123,2123,2122,2122,2120,2120,2119,2119,2117,2117,2116,2116,2114,2114,2112,2112,2111,2111,2109,2109,2108,2108,2107,2107,2106,2106,2105,2105,2104,2104,2103,2103,2102,2102,2101,2101,2100,2100,2099,2099,2098,2098,2095,2095,2094,2094,2092,2092,2089,2089,2088,2088,2084,2084,2083,2083,2082,2082,2081,2081,2079,2079,2076,2076,2073,2073,2072,2072,2071,2071,2070,2070,2069,2069,2067,2067,2066,2066,2063,2063,2062,2062,2060,2060,2058,2058,2056,2056,2055,2055,2054,2054,2053,2053,2052,2052,2051,2051,2050,2050,2047,2047,2046,2046,2044,2044,2043,2043,2042,2042,2039,2039,2038,2038,2036,2036,2034,2034,2029,2029,2027,2027,2026,2026,2025,2025,2024,2024,2023,2023,2022,2022,2020,2020,2018,2018,2017,2017,2015,2015,2014,2014,2009,2009,2008,2008,2007,2007,2006,2006,2004,2004,2003,2003,2001,2001,1999,1999,1997,1997,1995,1995,1994,1994,1993,1993,1992,1992,1990,1990,1987,1987,1983,1983,1982,1982,1980,1980,1979,1979,1977,1977,1975,1975,1973,1973,1972,1972,1970,1970,1969,1969,1967,1967,1966,1966,1964,1964,1962,1962,1960,1960,1957,1957,1955,1955,1954,1954,1953,1953,1952,1952,1950,1950,1949,1949,1948,1948,1946,1946,1944,1944,1942,1942,1940,1940,1937,1937,1936,1936,1935,1935,1934,1934,1932,1932,1931,1931,1930,1930,1929,1929,1927,1927,1926,1926,1925,1925,1923,1923,1922,1922,1918,1918,1917,1917,1916,1916,1914,1914,1913,1913,1912,1912,1911,1911,1910,1910,1909,1909,1908,1908,1907,1907,1906,1906,1905,1905,1904,1904,1903,1903,1902,1902,1901,1901,1900,1900,1899,1899,1898,1898,1897,1897,1896,1896,1895,1895,1894,1894,1893,1893,1892,1892,1890,1890,1889,1889,1888,1888,1885,1885,1884,1884,1883,1883,1882,1882,1881,1881,1876,1876,1874,1874,1873,1873,1871,1871,1869,1869,1868,1868,1867,1867,1866,1866,1864,1864,1863,1863,1862,1862,1861,1861,1859,1859,1858,1858,1857,1857,1854,1854,1851,1851,1850,1850,1847,1847,1846,1846,1845,1845,1844,1844,1843,1843,1842,1842,1841,1841,1839,1839,1838,1838,1837,1837,1836,1836,1835,1835,1833,1833,1832,1832,1828,1828,1827,1827,1825,1825,1824,1824,1823,1823,1822,1822,1821,1821,1819,1819,1818,1818,1817,1817,1816,1816,1814,1814,1813,1813,1810,1810,1809,1809,1808,1808,1807,1807,1804,1804,1803,1803,1802,1802,1801,1801,1797,1797,1796,1796,1792,1792,1788,1788,1787,1787,1786,1786,1782,1782,1781,1781,1780,1780,1777,1777,1775,1775,1774,1774,1771,1771,1769,1769,1767,1767,1765,1765,1763,1763,1759,1759,1758,1758,1755,1755,1754,1754,1752,1752,1751,1751,1750,1750,1747,1747,1746,1746,1744,1744,1743,1743,1740,1740,1739,1739,1737,1737,1736,1736,1735,1735,1733,1733,1732,1732,1731,1731,1730,1730,1729,1729,1728,1728,1726,1726,1725,1725,1724,1724,1723,1723,1722,1722,1721,1721,1720,1720,1719,1719,1718,1718,1717,1717,1714,1714,1711,1711,1710,1710,1709,1709,1707,1707,1703,1703,1702,1702,1701,1701,1700,1700,1699,1699,1698,1698,1697,1697,1696,1696,1695,1695,1694,1694,1693,1693,1692,1692,1690,1690,1688,1688,1687,1687,1686,1686,1683,1683,1682,1682,1681,1681,1679,1679,1678,1678,1677,1677,1675,1675,1674,1674,1670,1670,1668,1668,1667,1667,1666,1666,1665,1665,1664,1664,1663,1663,1661,1661,1660,1660,1659,1659,1658,1658,1657,1657,1652,1652,1651,1651,1650,1650,1649,1649,1648,1648,1647,1647,1645,1645,1643,1643,1642,1642,1640,1640,1638,1638,1637,1637,1636,1636,1635,1635,1632,1632,1631,1631,1630,1630,1629,1629,1628,1628,1627,1627,1626,1626,1625,1625,1622,1622,1620,1620,1619,1619,1618,1618,1615,1615,1613,1613,1611,1611,1610,1610,1608,1608,1607,1607,1605,1605,1604,1604,1603,1603,1601,1601,1600,1600,1597,1597,1596,1596,1593,1593,1592,1592,1591,1591,1589,1589,1588,1588,1586,1586,1582,1582,1581,1581,1579,1579,1578,1578,1577,1577,1574,1574,1573,1573,1572,1572,1571,1571,1570,1570,1569,1569,1568,1568,1567,1567,1566,1566,1565,1565,1563,1563,1558,1558,1557,1557,1556,1556,1555,1555,1554,1554,1550,1550,1549,1549,1547,1547,1546,1546,1545,1545,1544,1544,1543,1543,1542,1542,1540,1540,1539,1539,1538,1538,1537,1537,1535,1535,1533,1533,1531,1531,1527,1527,1526,1526,1525,1525,1524,1524,1523,1523,1522,1522,1521,1521,1520,1520,1519,1519,1518,1518,1517,1517,1516,1516,1515,1515,1514,1514,1512,1512,1510,1510,1509,1509,1508,1508,1506,1506,1505,1505,1504,1504,1503,1503,1502,1502,1498,1498,1497,1497,1496,1496,1495,1495,1494,1494,1493,1493,1491,1491,1490,1490,1489,1489,1487,1487,1486,1486,1484,1484,1482,1482,1481,1481,1480,1480,1478,1478,1477,1477,1475,1475,1474,1474,1473,1473,1472,1472,1470,1470,1469,1469,1468,1468,1467,1467,1465,1465,1464,1464,1463,1463,1462,1462,1461,1461,1459,1459,1458,1458,1457,1457,1455,1455,1454,1454,1452,1452,1450,1450,1449,1449,1448,1448,1447,1447,1445,1445,1444,1444,1443,1443,1441,1441,1436,1436,1435,1435,1434,1434,1432,1432,1431,1431,1430,1430,1429,1429,1428,1428,1426,1426,1425,1425,1424,1424,1423,1423,1421,1421,1420,1420,1417,1417,1416,1416,1413,1413,1412,1412,1410,1410,1408,1408,1407,1407,1403,1403,1402,1402,1401,1401,1397,1397,1396,1396,1395,1395,1393,1393,1390,1390,1389,1389,1388,1388,1385,1385,1384,1384,1382,1382,1381,1381,1380,1380,1379,1379,1378,1378,1377,1377,1376,1376,1375,1375,1374,1374,1373,1373,1372,1372,1371,1371,1370,1370,1367,1367,1365,1365,1364,1364,1363,1363,1362,1362,1360,1360,1359,1359,1357,1357,1356,1356,1354,1354,1353,1353,1352,1352,1351,1351,1349,1349,1348,1348,1347,1347,1346,1346,1345,1345,1344,1344,1343,1343,1342,1342,1341,1341,1340,1340,1339,1339,1338,1338,1337,1337,1335,1335,1333,1333,1331,1331,1330,1330,1327,1327,1326,1326,1325,1325,1323,1323,1322,1322,1321,1321,1319,1319,1318,1318,1317,1317,1316,1316,1315,1315,1314,1314,1313,1313,1311,1311,1310,1310,1309,1309,1308,1308,1305,1305,1304,1304,1302,1302,1300,1300,1299,1299,1298,1298,1297,1297,1295,1295,1294,1294,1293,1293,1291,1291,1289,1289,1288,1288,1286,1286,1285,1285,1283,1283,1282,1282,1281,1281,1280,1280,1277,1277,1275,1275,1273,1273,1272,1272,1271,1271,1269,1269,1268,1268,1266,1266,1264,1264,1263,1263,1262,1262,1261,1261,1260,1260,1258,1258,1256,1256,1255,1255,1254,1254,1253,1253,1251,1251,1250,1250,1249,1249,1248,1248,1246,1246,1245,1245,1244,1244,1243,1243,1242,1242,1241,1241,1239,1239,1237,1237,1236,1236,1235,1235,1233,1233,1231,1231,1230,1230,1229,1229,1227,1227,1226,1226,1225,1225,1224,1224,1223,1223,1222,1222,1221,1221,1220,1220,1219,1219,1218,1218,1217,1217,1216,1216,1214,1214,1213,1213,1212,1212,1211,1211,1210,1210,1208,1208,1207,1207,1206,1206,1205,1205,1204,1204,1203,1203,1201,1201,1199,1199,1198,1198,1197,1197,1196,1196,1195,1195,1194,1194,1193,1193,1192,1192,1191,1191,1190,1190,1189,1189,1188,1188,1187,1187,1186,1186,1185,1185,1184,1184,1183,1183,1180,1180,1179,1179,1178,1178,1177,1177,1176,1176,1175,1175,1172,1172,1171,1171,1170,1170,1169,1169,1168,1168,1166,1166,1165,1165,1164,1164,1163,1163,1162,1162,1161,1161,1160,1160,1159,1159,1158,1158,1157,1157,1156,1156,1155,1155,1154,1154,1153,1153,1152,1152,1151,1151,1150,1150,1149,1149,1148,1148,1147,1147,1146,1146,1143,1143,1142,1142,1141,1141,1140,1140,1139,1139,1136,1136,1135,1135,1134,1134,1133,1133,1132,1132,1131,1131,1126,1126,1125,1125,1124,1124,1123,1123,1122,1122,1121,1121,1120,1120,1118,1118,1117,1117,1116,1116,1114,1114,1113,1113,1112,1112,1110,1110,1108,1108,1107,1107,1106,1106,1105,1105,1104,1104,1103,1103,1101,1101,1100,1100,1099,1099,1096,1096,1095,1095,1094,1094,1093,1093,1092,1092,1091,1091,1090,1090,1089,1089,1088,1088,1086,1086,1085,1085,1084,1084,1083,1083,1081,1081,1079,1079,1078,1078,1077,1077,1076,1076,1075,1075,1074,1074,1073,1073,1072,1072,1071,1071,1070,1070,1069,1069,1068,1068,1067,1067,1066,1066,1065,1065,1062,1062,1061,1061,1058,1058,1057,1057,1052,1052,1051,1051,1050,1050,1049,1049,1048,1048,1047,1047,1046,1046,1045,1045,1044,1044,1043,1043,1042,1042,1041,1041,1040,1040,1039,1039,1038,1038,1037,1037,1035,1035,1034,1034,1032,1032,1030,1030,1029,1029,1028,1028,1027,1027,1026,1026,1025,1025,1024,1024,1023,1023,1022,1022,1021,1021,1020,1020,1018,1018,1017,1017,1016,1016,1015,1015,1014,1014,1013,1013,1012,1012,1011,1011,1010,1010,1008,1008,1007,1007,1006,1006,1005,1005,1004,1004,1002,1002,999,999,998,998,997,997,995,995,994,994,993,993,992,992,991,991,990,990,989,989,988,988,987,987,986,986,985,985,984,984,983,983,982,982,981,981,980,980,979,979,978,978,977,977,976,976,975,975,974,974,973,973,972,972,971,971,970,970,969,969,968,968,965,965,964,964,962,962,961,961,958,958,956,956,955,955,953,953,952,952,951,951,950,950,947,947,946,946,945,945,944,944,943,943,942,942,940,940,939,939,938,938,937,937,936,936,935,935,934,934,933,933,932,932,931,931,930,930,928,928,927,927,926,926,925,925,924,924,923,923,922,922,921,921,920,920,919,919,918,918,917,917,916,916,915,915,914,914,913,913,911,911,910,910,909,909,908,908,907,907,906,906,905,905,904,904,903,903,902,902,901,901,900,900,899,899,898,898,897,897,895,895,894,894,893,893,892,892,891,891,890,890,889,889,887,887,885,885,884,884,883,883,882,882,881,881,880,880,879,879,877,877,876,876,875,875,873,873,872,872,871,871,870,870,868,868,866,866,864,864,862,862,861,861,860,860,858,858,857,857,856,856,855,855,854,854,853,853,852,852,851,851,850,850,848,848,847,847,845,845,844,844,843,843,842,842,841,841,840,840,839,839,838,838,837,837,835,835,834,834,832,832,831,831,830,830,829,829,828,828,827,827,826,826,825,825,824,824,823,823,822,822,821,821,820,820,817,817,816,816,814,814,813,813,812,812,811,811,810,810,809,809,808,808,807,807,806,806,804,804,803,803,802,802,801,801,800,800,799,799,798,798,796,796,795,795,794,794,793,793,792,792,791,791,790,790,788,788,787,787,786,786,785,785,784,784,783,783,782,782,781,781,780,780,779,779,778,778,777,777,776,776,775,775,774,774,773,773,772,772,771,771,770,770,769,769,768,768,767,767,766,766,765,765,764,764,763,763,762,762,760,760,758,758,757,757,756,756,755,755,754,754,753,753,752,752,751,751,750,750,749,749,748,748,747,747,746,746,745,745,744,744,743,743,742,742,740,740,738,738,737,737,736,736,735,735,734,734,732,732,731,731,730,730,729,729,728,728,727,727,726,726,725,725,724,724,723,723,722,722,721,721,720,720,719,719,718,718,717,717,716,716,715,715,714,714,713,713,711,711,710,710,709,709,708,708,707,707,706,706,705,705,704,704,703,703,702,702,701,701,700,700,699,699,698,698,697,697,696,696,695,695,694,694,693,693,692,692,691,691,690,690,689,689,688,688,687,687,686,686,685,685,684,684,683,683,682,682,681,681,680,680,679,679,677,677,675,675,674,674,673,673,672,672,671,671,670,670,669,669,668,668,667,667,666,666,665,665,664,664,663,663,661,661,660,660,659,659,658,658,657,657,656,656,654,654,653,653,652,652,651,651,650,650,649,649,648,648,646,646,645,645,644,644,643,643,642,642,641,641,640,640,639,639,638,638,637,637,636,636,635,635,634,634,633,633,632,632,631,631,630,630,629,629,628,628,627,627,626,626,625,625,624,624,623,623,622,622,621,621,620,620,619,619,618,618,617,617,616,616,615,615,614,614,613,613,612,612,611,611,610,610,609,609,608,608,607,607,606,606,605,605,604,604,603,603,602,602,601,601,600,600,599,599,598,598,597,597,596,596,595,595,594,594,593,593,592,592,590,590,589,589,588,588,587,587,586,586,585,585,584,584,583,583,582,582,581,581,580,580,579,579,578,578,577,577,576,576,575,575,574,574,573,573,572,572,571,571,570,570,569,569,568,568,567,567,566,566,565,565,564,564,563,563,562,562,561,561,560,560,559,559,558,558,557,557,556,556,555,555,554,554,553,553,552,552,551,551,550,550,549,549,548,548,547,547,546,546,545,545,544,544,543,543,542,542,541,541,540,540,539,539,538,538,537,537,536,536,535,535,533,533,532,532,531,531,530,530,529,529,528,528,527,527,526,526,525,525,524,524,523,523,522,522,521,521,520,520,519,519,518,518,517,517,516,516,515,515,514,514,513,513,511,511,510,510,509,509,508,508,507,507,506,506,505,505,503,503,502,502,501,501,500,500,499,499,498,498,497,497,496,496,495,495,494,494,493,493,492,492,491,491,490,490,489,489,488,488,487,487,486,486,485,485,484,484,483,483,482,482,481,481,480,480,479,479,478,478,477,477,476,476,475,475,474,474,473,473,472,472,471,471,470,470,469,469,468,468,467,467,466,466,465,465,464,464,463,463,461,461,460,460,459,459,458,458,457,457,456,456,455,455,454,454,453,453,452,452,451,451,450,450,449,449,448,448,447,447,446,446,445,445,444,444,443,443,442,442,441,441,440,440,439,439,438,438,437,437,436,436,435,435,434,434,433,433,431,431,430,430,429,429,428,428,427,427,426,426,425,425,424,424,423,423,422,422,421,421,420,420,419,419,418,418,417,417,416,416,415,415,414,414,413,413,412,412,411,411,410,410,409,409,408,408,407,407,406,406,405,405,404,404,403,403,402,402,401,401,400,400,0.0],[56465,56465,56465,50623,50623,50504,50504,46068,46068,42936,42936,40877,40877,40351,40351,40192,40192,39993,39993,39423,39423,38387,38387,38105,38105,37413,37413,37231,37231,36966,36966,36956,36956,36742,36742,36357,36357,36066,36066,34695,34695,34499,34499,33113,33113,32973,32973,32737,32737,32306,32306,31865,31865,31732,31732,31494,31494,31373,31373,31058,31058,30851,30851,30842,30842,30577,30577,30329,30329,29898,29898,29549,29549,29505,29505,29392,29392,29107,29107,28975,28975,28531,28531,28200,28200,28104,28104,28052,28052,27965,27965,27419,27419,27417,27417,27386,27386,27321,27321,27123,27123,27030,27030,27008,27008,26924,26924,26850,26850,26682,26682,26671,26671,26630,26630,26558,26558,26554,26554,26550,26550,26341,26341,26258,26258,26227,26227,26214,26214,26213,26213,25901,25901,25837,25837,25825,25825,25751,25751,25634,25634,25621,25621,25273,25273,24998,24998,24960,24960,24948,24948,24908,24908,24820,24820,24818,24818,24771,24771,24731,24731,24708,24708,24621,24621,24534,24534,24495,24495,24488,24488,24448,24448,24430,24430,24413,24413,24330,24330,24282,24282,24270,24270,24256,24256,24225,24225,24216,24216,24213,24213,24073,24073,24011,24011,23984,23984,23920,23920,23901,23901,23823,23823,23765,23765,23757,23757,23496,23496,23196,23196,23186,23186,23136,23136,23105,23105,23094,23094,23025,23025,23004,23004,22982,22982,22930,22930,22867,22867,22855,22855,22805,22805,22796,22796,22788,22788,22744,22744,22648,22648,22602,22602,22585,22585,22543,22543,22480,22480,22431,22431,22421,22421,22369,22369,22334,22334,22258,22258,22231,22231,22214,22214,22121,22121,21994,21994,21989,21989,21987,21987,21947,21947,21882,21882,21880,21880,21838,21838,21737,21737,21719,21719,21714,21714,21695,21695,21656,21656,21525,21525,21520,21520,21371,21371,21365,21365,21358,21358,21353,21353,21306,21306,21197,21197,21081,21081,21053,21053,20951,20951,20950,20950,20948,20948,20674,20674,20656,20656,20649,20649,20580,20580,20563,20563,20547,20547,20520,20520,20517,20517,20443,20443,20436,20436,20429,20429,20388,20388,20387,20387,20289,20289,20264,20264,20152,20152,20101,20101,20090,20090,20086,20086,20083,20083,20077,20077,20067,20067,19901,19901,19878,19878,19877,19877,19869,19869,19805,19805,19750,19750,19745,19745,19632,19632,19605,19605,19583,19583,19524,19524,19521,19521,19459,19459,19368,19368,19276,19276,19274,19274,19266,19266,19233,19233,19216,19216,19208,19208,19184,19184,19060,19060,18973,18973,18916,18916,18851,18851,18828,18828,18826,18826,18723,18723,18712,18712,18688,18688,18623,18623,18581,18581,18572,18572,18514,18514,18395,18395,18363,18363,18308,18308,18301,18301,18288,18288,18287,18287,18266,18266,18253,18253,18244,18244,18230,18230,18219,18219,18202,18202,18193,18193,18187,18187,18174,18174,18129,18129,18120,18120,18062,18062,18044,18044,17988,17988,17985,17985,17971,17971,17964,17964,17954,17954,17950,17950,17949,17949,17939,17939,17911,17911,17903,17903,17896,17896,17887,17887,17854,17854,17838,17838,17792,17792,17772,17772,17741,17741,17702,17702,17685,17685,17668,17668,17569,17569,17527,17527,17525,17525,17493,17493,17489,17489,17448,17448,17442,17442,17421,17421,17418,17418,17289,17289,17271,17271,17250,17250,17215,17215,17177,17177,17164,17164,17150,17150,17020,17020,16951,16951,16950,16950,16947,16947,16921,16921,16907,16907,16900,16900,16856,16856,16848,16848,16809,16809,16794,16794,16776,16776,16741,16741,16687,16687,16674,16674,16640,16640,16614,16614,16609,16609,16598,16598,16591,16591,16590,16590,16586,16586,16584,16584,16486,16486,16483,16483,16465,16465,16453,16453,16449,16449,16447,16447,16445,16445,16428,16428,16402,16402,16385,16385,16368,16368,16334,16334,16296,16296,16287,16287,16262,16262,16236,16236,16224,16224,16220,16220,16192,16192,16181,16181,16166,16166,16107,16107,16099,16099,16070,16070,16051,16051,16031,16031,16004,16004,15978,15978,15972,15972,15954,15954,15843,15843,15823,15823,15747,15747,15740,15740,15689,15689,15656,15656,15641,15641,15616,15616,15599,15599,15595,15595,15589,15589,15581,15581,15574,15574,15558,15558,15550,15550,15508,15508,15494,15494,15481,15481,15473,15473,15459,15459,15453,15453,15414,15414,15401,15401,15398,15398,15392,15392,15360,15360,15348,15348,15347,15347,15294,15294,15266,15266,15262,15262,15250,15250,15238,15238,15231,15231,15221,15221,15215,15215,15195,15195,15190,15190,15165,15165,15146,15146,15091,15091,15088,15088,15063,15063,15055,15055,15022,15022,15020,15020,15001,15001,14966,14966,14962,14962,14953,14953,14935,14935,14908,14908,14885,14885,14880,14880,14856,14856,14832,14832,14829,14829,14818,14818,14814,14814,14803,14803,14782,14782,14766,14766,14759,14759,14740,14740,14733,14733,14731,14731,14722,14722,14701,14701,14675,14675,14669,14669,14624,14624,14618,14618,14612,14612,14606,14606,14596,14596,14594,14594,14570,14570,14569,14569,14561,14561,14560,14560,14530,14530,14529,14529,14502,14502,14497,14497,14485,14485,14474,14474,14469,14469,14456,14456,14437,14437,14429,14429,14420,14420,14396,14396,14395,14395,14387,14387,14375,14375,14366,14366,14346,14346,14339,14339,14318,14318,14296,14296,14289,14289,14255,14255,14228,14228,14213,14213,14205,14205,14203,14203,14179,14179,14124,14124,14088,14088,14054,14054,14047,14047,14037,14037,14031,14031,14028,14028,14012,14012,14011,14011,14009,14009,13989,13989,13973,13973,13962,13962,13955,13955,13921,13921,13897,13897,13894,13894,13880,13880,13851,13851,13847,13847,13826,13826,13811,13811,13809,13809,13772,13772,13763,13763,13748,13748,13739,13739,13733,13733,13726,13726,13696,13696,13694,13694,13665,13665,13658,13658,13655,13655,13609,13609,13603,13603,13594,13594,13583,13583,13579,13579,13578,13578,13552,13552,13550,13550,13542,13542,13540,13540,13539,13539,13527,13527,13520,13520,13502,13502,13495,13495,13493,13493,13471,13471,13466,13466,13448,13448,13447,13447,13446,13446,13445,13445,13441,13441,13437,13437,13431,13431,13430,13430,13429,13429,13422,13422,13420,13420,13414,13414,13395,13395,13393,13393,13389,13389,13386,13386,13379,13379,13370,13370,13355,13355,13353,13353,13344,13344,13339,13339,13331,13331,13328,13328,13316,13316,13302,13302,13290,13290,13289,13289,13258,13258,13248,13248,13235,13235,13221,13221,13216,13216,13203,13203,13192,13192,13177,13177,13167,13167,13155,13155,13149,13149,13140,13140,13133,13133,13130,13130,13126,13126,13113,13113,13111,13111,13107,13107,13087,13087,13083,13083,13077,13077,13076,13076,13054,13054,13031,13031,13029,13029,13001,13001,12990,12990,12989,12989,12976,12976,12975,12975,12958,12958,12953,12953,12946,12946,12942,12942,12939,12939,12938,12938,12916,12916,12909,12909,12907,12907,12887,12887,12884,12884,12874,12874,12871,12871,12868,12868,12864,12864,12856,12856,12855,12855,12846,12846,12830,12830,12817,12817,12811,12811,12806,12806,12787,12787,12761,12761,12760,12760,12741,12741,12730,12730,12693,12693,12690,12690,12683,12683,12675,12675,12668,12668,12658,12658,12653,12653,12641,12641,12637,12637,12630,12630,12625,12625,12617,12617,12614,12614,12611,12611,12601,12601,12597,12597,12592,12592,12590,12590,12585,12585,12584,12584,12574,12574,12567,12567,12562,12562,12559,12559,12552,12552,12549,12549,12542,12542,12537,12537,12517,12517,12500,12500,12495,12495,12489,12489,12487,12487,12477,12477,12472,12472,12464,12464,12456,12456,12438,12438,12425,12425,12409,12409,12393,12393,12392,12392,12390,12390,12388,12388,12386,12386,12381,12381,12377,12377,12370,12370,12362,12362,12359,12359,12358,12358,12355,12355,12354,12354,12352,12352,12350,12350,12337,12337,12334,12334,12313,12313,12310,12310,12308,12308,12303,12303,12276,12276,12272,12272,12269,12269,12260,12260,12258,12258,12247,12247,12244,12244,12235,12235,12233,12233,12217,12217,12212,12212,12207,12207,12201,12201,12191,12191,12190,12190,12183,12183,12178,12178,12161,12161,12155,12155,12150,12150,12149,12149,12148,12148,12141,12141,12083,12083,12068,12068,12067,12067,12066,12066,12063,12063,12058,12058,12041,12041,12037,12037,12025,12025,12010,12010,12001,12001,11995,11995,11992,11992,11979,11979,11970,11970,11967,11967,11953,11953,11942,11942,11939,11939,11935,11935,11932,11932,11918,11918,11915,11915,11879,11879,11877,11877,11851,11851,11846,11846,11837,11837,11829,11829,11820,11820,11816,11816,11782,11782,11772,11772,11771,11771,11767,11767,11766,11766,11763,11763,11744,11744,11738,11738,11702,11702,11696,11696,11694,11694,11693,11693,11691,11691,11687,11687,11685,11685,11667,11667,11653,11653,11649,11649,11633,11633,11629,11629,11610,11610,11605,11605,11578,11578,11562,11562,11556,11556,11555,11555,11553,11553,11540,11540,11535,11535,11534,11534,11532,11532,11530,11530,11517,11517,11507,11507,11501,11501,11499,11499,11493,11493,11478,11478,11474,11474,11473,11473,11470,11470,11461,11461,11450,11450,11449,11449,11447,11447,11445,11445,11444,11444,11440,11440,11438,11438,11421,11421,11417,11417,11396,11396,11381,11381,11380,11380,11377,11377,11362,11362,11357,11357,11356,11356,11353,11353,11338,11338,11309,11309,11308,11308,11305,11305,11303,11303,11296,11296,11295,11295,11270,11270,11262,11262,11259,11259,11258,11258,11245,11245,11244,11244,11242,11242,11235,11235,11233,11233,11227,11227,11223,11223,11218,11218,11215,11215,11214,11214,11205,11205,11196,11196,11192,11192,11189,11189,11187,11187,11184,11184,11182,11182,11181,11181,11178,11178,11154,11154,11143,11143,11096,11096,11092,11092,11089,11089,11073,11073,11067,11067,11065,11065,11061,11061,11054,11054,11050,11050,11047,11047,11046,11046,11041,11041,11037,11037,11034,11034,11030,11030,11028,11028,11027,11027,11015,11015,11012,11012,10999,10999,10995,10995,10993,10993,10980,10980,10974,10974,10972,10972,10969,10969,10966,10966,10965,10965,10959,10959,10957,10957,10953,10953,10951,10951,10941,10941,10940,10940,10926,10926,10917,10917,10892,10892,10883,10883,10882,10882,10879,10879,10875,10875,10870,10870,10868,10868,10867,10867,10861,10861,10860,10860,10845,10845,10825,10825,10824,10824,10823,10823,10816,10816,10812,10812,10808,10808,10802,10802,10795,10795,10794,10794,10789,10789,10788,10788,10785,10785,10784,10784,10778,10778,10777,10777,10776,10776,10772,10772,10766,10766,10765,10765,10750,10750,10741,10741,10737,10737,10730,10730,10729,10729,10712,10712,10711,10711,10709,10709,10697,10697,10694,10694,10689,10689,10671,10671,10642,10642,10637,10637,10636,10636,10632,10632,10631,10631,10625,10625,10623,10623,10619,10619,10617,10617,10614,10614,10610,10610,10609,10609,10606,10606,10596,10596,10592,10592,10591,10591,10589,10589,10572,10572,10567,10567,10565,10565,10558,10558,10556,10556,10552,10552,10547,10547,10539,10539,10533,10533,10529,10529,10526,10526,10512,10512,10502,10502,10494,10494,10480,10480,10477,10477,10475,10475,10465,10465,10464,10464,10454,10454,10453,10453,10450,10450,10441,10441,10429,10429,10422,10422,10410,10410,10409,10409,10406,10406,10403,10403,10402,10402,10397,10397,10396,10396,10389,10389,10386,10386,10380,10380,10373,10373,10370,10370,10369,10369,10354,10354,10352,10352,10347,10347,10344,10344,10337,10337,10336,10336,10335,10335,10334,10334,10323,10323,10322,10322,10321,10321,10320,10320,10317,10317,10311,10311,10308,10308,10307,10307,10294,10294,10291,10291,10282,10282,10264,10264,10261,10261,10259,10259,10258,10258,10255,10255,10254,10254,10251,10251,10246,10246,10245,10245,10243,10243,10242,10242,10241,10241,10237,10237,10232,10232,10229,10229,10228,10228,10226,10226,10210,10210,10198,10198,10194,10194,10184,10184,10173,10173,10166,10166,10150,10150,10146,10146,10133,10133,10123,10123,10105,10105,10101,10101,10095,10095,10089,10089,10082,10082,10080,10080,10078,10078,10075,10075,10071,10071,10068,10068,10063,10063,10061,10061,10057,10057,10055,10055,10048,10048,10043,10043,10042,10042,10041,10041,10029,10029,10028,10028,10025,10025,10014,10014,10007,10007,10003,10003,10002,10002,9996,9996,9994,9994,9986,9986,9979,9979,9977,9977,9973,9973,9970,9970,9951,9951,9944,9944,9943,9943,9939,9939,9937,9937,9936,9936,9932,9932,9928,9928,9927,9927,9922,9922,9920,9920,9912,9912,9890,9890,9888,9888,9875,9875,9870,9870,9858,9858,9855,9855,9850,9850,9842,9842,9840,9840,9836,9836,9806,9806,9801,9801,9799,9799,9792,9792,9785,9785,9779,9779,9776,9776,9774,9774,9771,9771,9770,9770,9761,9761,9759,9759,9756,9756,9750,9750,9742,9742,9738,9738,9735,9735,9727,9727,9717,9717,9713,9713,9712,9712,9695,9695,9694,9694,9691,9691,9690,9690,9684,9684,9683,9683,9678,9678,9677,9677,9675,9675,9670,9670,9667,9667,9662,9662,9657,9657,9652,9652,9641,9641,9639,9639,9629,9629,9617,9617,9613,9613,9612,9612,9602,9602,9598,9598,9597,9597,9595,9595,9577,9577,9574,9574,9571,9571,9565,9565,9559,9559,9558,9558,9553,9553,9551,9551,9550,9550,9548,9548,9540,9540,9539,9539,9528,9528,9526,9526,9508,9508,9505,9505,9502,9502,9499,9499,9496,9496,9494,9494,9493,9493,9488,9488,9486,9486,9484,9484,9483,9483,9474,9474,9470,9470,9467,9467,9461,9461,9459,9459,9456,9456,9449,9449,9443,9443,9440,9440,9435,9435,9434,9434,9433,9433,9428,9428,9425,9425,9422,9422,9420,9420,9414,9414,9413,9413,9409,9409,9407,9407,9406,9406,9402,9402,9396,9396,9394,9394,9385,9385,9382,9382,9380,9380,9374,9374,9371,9371,9369,9369,9363,9363,9359,9359,9355,9355,9349,9349,9348,9348,9343,9343,9340,9340,9320,9320,9314,9314,9304,9304,9290,9290,9287,9287,9286,9286,9282,9282,9281,9281,9280,9280,9276,9276,9275,9275,9258,9258,9256,9256,9248,9248,9241,9241,9240,9240,9232,9232,9230,9230,9222,9222,9221,9221,9216,9216,9213,9213,9208,9208,9207,9207,9199,9199,9198,9198,9186,9186,9184,9184,9182,9182,9180,9180,9179,9179,9177,9177,9148,9148,9146,9146,9128,9128,9120,9120,9113,9113,9110,9110,9107,9107,9106,9106,9103,9103,9102,9102,9092,9092,9084,9084,9082,9082,9081,9081,9072,9072,9055,9055,9052,9052,9046,9046,9042,9042,9038,9038,9034,9034,9031,9031,9019,9019,8997,8997,8992,8992,8991,8991,8988,8988,8987,8987,8981,8981,8977,8977,8972,8972,8966,8966,8964,8964,8955,8955,8948,8948,8946,8946,8925,8925,8921,8921,8920,8920,8918,8918,8913,8913,8911,8911,8909,8909,8904,8904,8898,8898,8895,8895,8889,8889,8885,8885,8883,8883,8882,8882,8880,8880,8875,8875,8874,8874,8873,8873,8868,8868,8866,8866,8861,8861,8860,8860,8857,8857,8854,8854,8852,8852,8833,8833,8831,8831,8829,8829,8828,8828,8826,8826,8819,8819,8817,8817,8807,8807,8805,8805,8801,8801,8797,8797,8793,8793,8791,8791,8779,8779,8778,8778,8776,8776,8772,8772,8771,8771,8770,8770,8766,8766,8765,8765,8762,8762,8759,8759,8756,8756,8748,8748,8743,8743,8741,8741,8739,8739,8736,8736,8728,8728,8727,8727,8726,8726,8723,8723,8720,8720,8717,8717,8714,8714,8708,8708,8702,8702,8700,8700,8699,8699,8691,8691,8688,8688,8687,8687,8685,8685,8682,8682,8680,8680,8678,8678,8672,8672,8667,8667,8662,8662,8656,8656,8655,8655,8648,8648,8646,8646,8643,8643,8638,8638,8630,8630,8628,8628,8627,8627,8623,8623,8622,8622,8619,8619,8618,8618,8616,8616,8612,8612,8607,8607,8606,8606,8602,8602,8600,8600,8596,8596,8590,8590,8582,8582,8581,8581,8579,8579,8576,8576,8573,8573,8572,8572,8567,8567,8565,8565,8561,8561,8558,8558,8548,8548,8546,8546,8540,8540,8535,8535,8534,8534,8526,8526,8523,8523,8519,8519,8515,8515,8512,8512,8507,8507,8502,8502,8497,8497,8494,8494,8488,8488,8481,8481,8479,8479,8475,8475,8468,8468,8463,8463,8460,8460,8456,8456,8454,8454,8453,8453,8450,8450,8449,8449,8448,8448,8447,8447,8446,8446,8441,8441,8436,8436,8433,8433,8428,8428,8414,8414,8412,8412,8404,8404,8402,8402,8400,8400,8393,8393,8387,8387,8386,8386,8382,8382,8381,8381,8379,8379,8377,8377,8369,8369,8349,8349,8341,8341,8340,8340,8337,8337,8334,8334,8333,8333,8328,8328,8322,8322,8321,8321,8320,8320,8319,8319,8318,8318,8316,8316,8315,8315,8312,8312,8309,8309,8300,8300,8295,8295,8294,8294,8286,8286,8277,8277,8276,8276,8274,8274,8269,8269,8257,8257,8251,8251,8246,8246,8242,8242,8241,8241,8237,8237,8235,8235,8234,8234,8233,8233,8228,8228,8222,8222,8220,8220,8219,8219,8208,8208,8197,8197,8193,8193,8192,8192,8183,8183,8181,8181,8180,8180,8166,8166,8163,8163,8154,8154,8153,8153,8150,8150,8146,8146,8141,8141,8138,8138,8137,8137,8136,8136,8131,8131,8114,8114,8113,8113,8110,8110,8107,8107,8105,8105,8103,8103,8095,8095,8093,8093,8088,8088,8086,8086,8081,8081,8079,8079,8077,8077,8076,8076,8075,8075,8074,8074,8070,8070,8069,8069,8068,8068,8066,8066,8065,8065,8062,8062,8058,8058,8057,8057,8051,8051,8041,8041,8038,8038,8036,8036,8032,8032,8025,8025,8017,8017,8014,8014,8012,8012,8011,8011,8010,8010,8009,8009,8008,8008,8007,8007,8004,8004,7998,7998,7994,7994,7989,7989,7988,7988,7986,7986,7984,7984,7982,7982,7981,7981,7977,7977,7975,7975,7970,7970,7968,7968,7966,7966,7960,7960,7958,7958,7945,7945,7940,7940,7939,7939,7937,7937,7935,7935,7934,7934,7932,7932,7930,7930,7929,7929,7928,7928,7924,7924,7923,7923,7919,7919,7917,7917,7913,7913,7912,7912,7910,7910,7906,7906,7899,7899,7895,7895,7892,7892,7890,7890,7885,7885,7879,7879,7878,7878,7877,7877,7874,7874,7869,7869,7868,7868,7857,7857,7856,7856,7855,7855,7854,7854,7852,7852,7851,7851,7848,7848,7835,7835,7832,7832,7831,7831,7830,7830,7827,7827,7824,7824,7818,7818,7817,7817,7815,7815,7814,7814,7811,7811,7809,7809,7808,7808,7802,7802,7801,7801,7799,7799,7788,7788,7786,7786,7785,7785,7776,7776,7748,7748,7746,7746,7743,7743,7736,7736,7735,7735,7733,7733,7732,7732,7731,7731,7729,7729,7726,7726,7723,7723,7722,7722,7717,7717,7715,7715,7711,7711,7710,7710,7705,7705,7701,7701,7696,7696,7695,7695,7689,7689,7688,7688,7685,7685,7683,7683,7679,7679,7678,7678,7677,7677,7669,7669,7667,7667,7656,7656,7654,7654,7653,7653,7650,7650,7648,7648,7646,7646,7643,7643,7638,7638,7636,7636,7630,7630,7626,7626,7624,7624,7623,7623,7615,7615,7609,7609,7607,7607,7606,7606,7604,7604,7602,7602,7599,7599,7598,7598,7589,7589,7587,7587,7584,7584,7577,7577,7574,7574,7573,7573,7570,7570,7567,7567,7560,7560,7558,7558,7557,7557,7552,7552,7545,7545,7541,7541,7540,7540,7539,7539,7538,7538,7537,7537,7533,7533,7531,7531,7527,7527,7524,7524,7523,7523,7521,7521,7519,7519,7517,7517,7510,7510,7507,7507,7503,7503,7501,7501,7496,7496,7489,7489,7485,7485,7479,7479,7475,7475,7474,7474,7473,7473,7471,7471,7470,7470,7469,7469,7468,7468,7467,7467,7461,7461,7458,7458,7456,7456,7452,7452,7447,7447,7443,7443,7432,7432,7431,7431,7430,7430,7424,7424,7423,7423,7421,7421,7419,7419,7415,7415,7412,7412,7411,7411,7410,7410,7409,7409,7401,7401,7396,7396,7395,7395,7393,7393,7388,7388,7385,7385,7384,7384,7380,7380,7377,7377,7372,7372,7367,7367,7365,7365,7364,7364,7361,7361,7360,7360,7358,7358,7355,7355,7351,7351,7350,7350,7347,7347,7346,7346,7344,7344,7342,7342,7339,7339,7338,7338,7337,7337,7336,7336,7330,7330,7326,7326,7321,7321,7319,7319,7318,7318,7315,7315,7314,7314,7307,7307,7306,7306,7305,7305,7301,7301,7300,7300,7294,7294,7293,7293,7292,7292,7285,7285,7280,7280,7279,7279,7278,7278,7277,7277,7276,7276,7273,7273,7269,7269,7268,7268,7267,7267,7265,7265,7263,7263,7260,7260,7257,7257,7255,7255,7248,7248,7247,7247,7244,7244,7242,7242,7237,7237,7230,7230,7229,7229,7222,7222,7220,7220,7218,7218,7214,7214,7213,7213,7208,7208,7203,7203,7201,7201,7199,7199,7189,7189,7187,7187,7180,7180,7175,7175,7171,7171,7169,7169,7168,7168,7163,7163,7158,7158,7157,7157,7156,7156,7150,7150,7149,7149,7147,7147,7136,7136,7135,7135,7129,7129,7125,7125,7121,7121,7116,7116,7113,7113,7109,7109,7107,7107,7106,7106,7102,7102,7094,7094,7091,7091,7089,7089,7088,7088,7085,7085,7084,7084,7082,7082,7081,7081,7076,7076,7074,7074,7068,7068,7067,7067,7065,7065,7063,7063,7062,7062,7059,7059,7057,7057,7056,7056,7048,7048,7044,7044,7043,7043,7042,7042,7040,7040,7039,7039,7037,7037,7036,7036,7035,7035,7030,7030,7027,7027,7024,7024,7023,7023,7022,7022,7019,7019,7018,7018,7016,7016,7014,7014,7006,7006,7004,7004,7000,7000,6999,6999,6988,6988,6987,6987,6984,6984,6977,6977,6976,6976,6971,6971,6970,6970,6969,6969,6968,6968,6966,6966,6962,6962,6959,6959,6954,6954,6953,6953,6948,6948,6946,6946,6940,6940,6937,6937,6930,6930,6929,6929,6928,6928,6927,6927,6926,6926,6924,6924,6923,6923,6914,6914,6913,6913,6907,6907,6906,6906,6905,6905,6904,6904,6903,6903,6902,6902,6899,6899,6898,6898,6896,6896,6894,6894,6893,6893,6885,6885,6884,6884,6882,6882,6881,6881,6880,6880,6877,6877,6875,6875,6873,6873,6870,6870,6868,6868,6865,6865,6861,6861,6857,6857,6854,6854,6847,6847,6843,6843,6842,6842,6839,6839,6836,6836,6835,6835,6829,6829,6822,6822,6814,6814,6812,6812,6809,6809,6808,6808,6806,6806,6803,6803,6800,6800,6795,6795,6793,6793,6788,6788,6787,6787,6786,6786,6782,6782,6777,6777,6775,6775,6771,6771,6752,6752,6748,6748,6746,6746,6745,6745,6737,6737,6733,6733,6732,6732,6721,6721,6718,6718,6716,6716,6713,6713,6711,6711,6709,6709,6708,6708,6707,6707,6706,6706,6705,6705,6703,6703,6701,6701,6700,6700,6696,6696,6694,6694,6693,6693,6692,6692,6691,6691,6690,6690,6685,6685,6684,6684,6681,6681,6680,6680,6679,6679,6678,6678,6677,6677,6674,6674,6672,6672,6667,6667,6665,6665,6664,6664,6662,6662,6661,6661,6653,6653,6649,6649,6643,6643,6642,6642,6641,6641,6640,6640,6638,6638,6637,6637,6636,6636,6633,6633,6630,6630,6626,6626,6619,6619,6614,6614,6610,6610,6608,6608,6607,6607,6604,6604,6603,6603,6601,6601,6598,6598,6593,6593,6592,6592,6591,6591,6588,6588,6587,6587,6583,6583,6576,6576,6574,6574,6572,6572,6570,6570,6569,6569,6568,6568,6567,6567,6564,6564,6562,6562,6561,6561,6555,6555,6554,6554,6552,6552,6549,6549,6544,6544,6536,6536,6534,6534,6532,6532,6530,6530,6529,6529,6526,6526,6525,6525,6522,6522,6521,6521,6519,6519,6518,6518,6515,6515,6513,6513,6511,6511,6508,6508,6507,6507,6506,6506,6505,6505,6504,6504,6499,6499,6498,6498,6497,6497,6496,6496,6495,6495,6493,6493,6492,6492,6490,6490,6484,6484,6482,6482,6480,6480,6476,6476,6475,6475,6472,6472,6465,6465,6464,6464,6462,6462,6461,6461,6457,6457,6456,6456,6450,6450,6449,6449,6448,6448,6446,6446,6445,6445,6444,6444,6442,6442,6436,6436,6434,6434,6430,6430,6427,6427,6426,6426,6425,6425,6422,6422,6421,6421,6417,6417,6411,6411,6410,6410,6409,6409,6407,6407,6401,6401,6399,6399,6396,6396,6393,6393,6390,6390,6389,6389,6388,6388,6385,6385,6384,6384,6381,6381,6380,6380,6373,6373,6372,6372,6370,6370,6368,6368,6361,6361,6359,6359,6357,6357,6356,6356,6349,6349,6347,6347,6345,6345,6342,6342,6340,6340,6338,6338,6337,6337,6335,6335,6331,6331,6327,6327,6326,6326,6325,6325,6316,6316,6315,6315,6314,6314,6313,6313,6311,6311,6308,6308,6306,6306,6305,6305,6303,6303,6300,6300,6299,6299,6298,6298,6297,6297,6296,6296,6295,6295,6294,6294,6289,6289,6287,6287,6285,6285,6277,6277,6275,6275,6271,6271,6269,6269,6268,6268,6267,6267,6266,6266,6262,6262,6259,6259,6258,6258,6257,6257,6255,6255,6253,6253,6252,6252,6251,6251,6249,6249,6247,6247,6245,6245,6244,6244,6242,6242,6240,6240,6235,6235,6233,6233,6232,6232,6228,6228,6227,6227,6224,6224,6223,6223,6222,6222,6221,6221,6220,6220,6216,6216,6215,6215,6212,6212,6211,6211,6210,6210,6209,6209,6206,6206,6202,6202,6200,6200,6197,6197,6194,6194,6192,6192,6191,6191,6190,6190,6187,6187,6176,6176,6175,6175,6171,6171,6167,6167,6166,6166,6163,6163,6161,6161,6159,6159,6154,6154,6153,6153,6148,6148,6146,6146,6143,6143,6134,6134,6133,6133,6130,6130,6128,6128,6127,6127,6124,6124,6123,6123,6121,6121,6120,6120,6119,6119,6117,6117,6116,6116,6114,6114,6113,6113,6107,6107,6106,6106,6104,6104,6101,6101,6099,6099,6096,6096,6091,6091,6089,6089,6086,6086,6081,6081,6076,6076,6074,6074,6066,6066,6065,6065,6063,6063,6058,6058,6057,6057,6056,6056,6055,6055,6053,6053,6052,6052,6051,6051,6047,6047,6045,6045,6038,6038,6036,6036,6032,6032,6023,6023,6022,6022,6013,6013,6007,6007,6006,6006,6004,6004,6000,6000,5997,5997,5995,5995,5994,5994,5992,5992,5990,5990,5988,5988,5986,5986,5985,5985,5982,5982,5981,5981,5980,5980,5979,5979,5974,5974,5970,5970,5968,5968,5967,5967,5966,5966,5965,5965,5963,5963,5961,5961,5958,5958,5957,5957,5956,5956,5954,5954,5953,5953,5947,5947,5945,5945,5944,5944,5942,5942,5941,5941,5939,5939,5938,5938,5937,5937,5933,5933,5927,5927,5923,5923,5918,5918,5913,5913,5911,5911,5909,5909,5903,5903,5901,5901,5897,5897,5896,5896,5894,5894,5892,5892,5890,5890,5886,5886,5884,5884,5880,5880,5879,5879,5878,5878,5872,5872,5871,5871,5869,5869,5867,5867,5864,5864,5860,5860,5859,5859,5855,5855,5853,5853,5849,5849,5848,5848,5836,5836,5833,5833,5832,5832,5831,5831,5829,5829,5820,5820,5819,5819,5818,5818,5817,5817,5816,5816,5811,5811,5809,5809,5808,5808,5806,5806,5804,5804,5802,5802,5795,5795,5794,5794,5789,5789,5787,5787,5776,5776,5774,5774,5773,5773,5772,5772,5771,5771,5770,5770,5766,5766,5765,5765,5764,5764,5763,5763,5753,5753,5749,5749,5748,5748,5745,5745,5744,5744,5743,5743,5742,5742,5740,5740,5739,5739,5735,5735,5734,5734,5733,5733,5729,5729,5725,5725,5724,5724,5723,5723,5722,5722,5716,5716,5708,5708,5705,5705,5704,5704,5702,5702,5701,5701,5699,5699,5698,5698,5697,5697,5695,5695,5694,5694,5693,5693,5692,5692,5684,5684,5683,5683,5679,5679,5677,5677,5676,5676,5673,5673,5672,5672,5670,5670,5669,5669,5668,5668,5667,5667,5662,5662,5660,5660,5655,5655,5654,5654,5653,5653,5651,5651,5650,5650,5645,5645,5644,5644,5642,5642,5641,5641,5640,5640,5639,5639,5638,5638,5634,5634,5633,5633,5631,5631,5630,5630,5628,5628,5627,5627,5626,5626,5625,5625,5621,5621,5620,5620,5611,5611,5604,5604,5603,5603,5602,5602,5601,5601,5597,5597,5595,5595,5592,5592,5589,5589,5587,5587,5586,5586,5582,5582,5580,5580,5574,5574,5572,5572,5570,5570,5569,5569,5568,5568,5566,5566,5564,5564,5561,5561,5559,5559,5558,5558,5557,5557,5555,5555,5553,5553,5552,5552,5550,5550,5549,5549,5547,5547,5545,5545,5541,5541,5540,5540,5533,5533,5529,5529,5528,5528,5526,5526,5525,5525,5524,5524,5523,5523,5522,5522,5520,5520,5515,5515,5514,5514,5513,5513,5512,5512,5509,5509,5508,5508,5504,5504,5500,5500,5499,5499,5498,5498,5497,5497,5495,5495,5493,5493,5490,5490,5489,5489,5487,5487,5485,5485,5484,5484,5481,5481,5480,5480,5479,5479,5478,5478,5476,5476,5469,5469,5464,5464,5463,5463,5461,5461,5458,5458,5457,5457,5456,5456,5452,5452,5451,5451,5448,5448,5447,5447,5444,5444,5442,5442,5439,5439,5434,5434,5433,5433,5431,5431,5430,5430,5427,5427,5426,5426,5425,5425,5423,5423,5422,5422,5420,5420,5415,5415,5411,5411,5409,5409,5406,5406,5403,5403,5399,5399,5394,5394,5393,5393,5392,5392,5391,5391,5390,5390,5386,5386,5384,5384,5383,5383,5381,5381,5379,5379,5370,5370,5366,5366,5362,5362,5361,5361,5353,5353,5351,5351,5350,5350,5347,5347,5343,5343,5339,5339,5336,5336,5335,5335,5334,5334,5329,5329,5328,5328,5326,5326,5322,5322,5321,5321,5317,5317,5316,5316,5314,5314,5312,5312,5311,5311,5309,5309,5308,5308,5307,5307,5306,5306,5305,5305,5296,5296,5294,5294,5293,5293,5290,5290,5289,5289,5285,5285,5280,5280,5279,5279,5277,5277,5275,5275,5270,5270,5267,5267,5265,5265,5264,5264,5256,5256,5255,5255,5254,5254,5253,5253,5252,5252,5250,5250,5249,5249,5246,5246,5244,5244,5243,5243,5242,5242,5241,5241,5238,5238,5237,5237,5236,5236,5232,5232,5231,5231,5230,5230,5223,5223,5221,5221,5219,5219,5218,5218,5215,5215,5213,5213,5212,5212,5208,5208,5205,5205,5204,5204,5203,5203,5198,5198,5197,5197,5195,5195,5191,5191,5190,5190,5186,5186,5184,5184,5181,5181,5180,5180,5179,5179,5178,5178,5177,5177,5174,5174,5172,5172,5171,5171,5169,5169,5168,5168,5166,5166,5164,5164,5162,5162,5161,5161,5160,5160,5159,5159,5158,5158,5157,5157,5155,5155,5154,5154,5153,5153,5148,5148,5147,5147,5146,5146,5143,5143,5142,5142,5139,5139,5138,5138,5137,5137,5133,5133,5129,5129,5127,5127,5126,5126,5124,5124,5122,5122,5121,5121,5120,5120,5118,5118,5117,5117,5116,5116,5115,5115,5111,5111,5109,5109,5107,5107,5106,5106,5103,5103,5102,5102,5101,5101,5099,5099,5097,5097,5096,5096,5094,5094,5089,5089,5088,5088,5087,5087,5086,5086,5085,5085,5084,5084,5082,5082,5079,5079,5077,5077,5076,5076,5075,5075,5074,5074,5073,5073,5070,5070,5069,5069,5066,5066,5063,5063,5062,5062,5061,5061,5060,5060,5058,5058,5056,5056,5054,5054,5053,5053,5051,5051,5048,5048,5040,5040,5038,5038,5037,5037,5035,5035,5034,5034,5033,5033,5032,5032,5029,5029,5026,5026,5025,5025,5023,5023,5021,5021,5019,5019,5018,5018,5017,5017,5016,5016,5015,5015,5010,5010,5007,5007,5004,5004,5003,5003,5002,5002,5000,5000,4999,4999,4998,4998,4996,4996,4991,4991,4990,4990,4989,4989,4988,4988,4986,4986,4983,4983,4982,4982,4981,4981,4980,4980,4979,4979,4977,4977,4975,4975,4974,4974,4973,4973,4972,4972,4969,4969,4967,4967,4966,4966,4965,4965,4964,4964,4963,4963,4962,4962,4961,4961,4960,4960,4958,4958,4956,4956,4955,4955,4953,4953,4949,4949,4948,4948,4945,4945,4944,4944,4941,4941,4940,4940,4938,4938,4934,4934,4931,4931,4930,4930,4929,4929,4927,4927,4923,4923,4922,4922,4920,4920,4917,4917,4916,4916,4915,4915,4914,4914,4913,4913,4909,4909,4908,4908,4907,4907,4906,4906,4904,4904,4903,4903,4902,4902,4901,4901,4900,4900,4898,4898,4897,4897,4896,4896,4895,4895,4894,4894,4893,4893,4892,4892,4891,4891,4890,4890,4889,4889,4887,4887,4886,4886,4885,4885,4882,4882,4881,4881,4879,4879,4878,4878,4877,4877,4876,4876,4875,4875,4869,4869,4866,4866,4865,4865,4863,4863,4861,4861,4860,4860,4856,4856,4854,4854,4853,4853,4852,4852,4850,4850,4847,4847,4846,4846,4845,4845,4844,4844,4842,4842,4838,4838,4837,4837,4835,4835,4832,4832,4831,4831,4827,4827,4825,4825,4823,4823,4822,4822,4821,4821,4820,4820,4817,4817,4815,4815,4814,4814,4813,4813,4809,4809,4808,4808,4806,4806,4803,4803,4802,4802,4800,4800,4796,4796,4795,4795,4794,4794,4792,4792,4790,4790,4789,4789,4787,4787,4784,4784,4783,4783,4782,4782,4781,4781,4778,4778,4770,4770,4766,4766,4762,4762,4761,4761,4760,4760,4759,4759,4758,4758,4757,4757,4756,4756,4753,4753,4752,4752,4750,4750,4748,4748,4747,4747,4746,4746,4744,4744,4741,4741,4739,4739,4738,4738,4737,4737,4736,4736,4734,4734,4733,4733,4731,4731,4729,4729,4728,4728,4727,4727,4726,4726,4725,4725,4721,4721,4720,4720,4719,4719,4716,4716,4714,4714,4713,4713,4710,4710,4703,4703,4702,4702,4701,4701,4700,4700,4699,4699,4696,4696,4695,4695,4694,4694,4693,4693,4690,4690,4685,4685,4681,4681,4680,4680,4679,4679,4678,4678,4677,4677,4674,4674,4672,4672,4671,4671,4670,4670,4668,4668,4667,4667,4665,4665,4664,4664,4663,4663,4662,4662,4661,4661,4659,4659,4657,4657,4654,4654,4653,4653,4652,4652,4651,4651,4650,4650,4649,4649,4647,4647,4646,4646,4645,4645,4644,4644,4642,4642,4638,4638,4637,4637,4634,4634,4633,4633,4632,4632,4631,4631,4627,4627,4626,4626,4625,4625,4623,4623,4620,4620,4619,4619,4618,4618,4617,4617,4614,4614,4613,4613,4608,4608,4607,4607,4606,4606,4605,4605,4602,4602,4601,4601,4600,4600,4595,4595,4594,4594,4593,4593,4592,4592,4591,4591,4587,4587,4586,4586,4585,4585,4584,4584,4583,4583,4581,4581,4576,4576,4575,4575,4570,4570,4568,4568,4567,4567,4566,4566,4564,4564,4563,4563,4561,4561,4560,4560,4552,4552,4551,4551,4548,4548,4547,4547,4546,4546,4544,4544,4543,4543,4541,4541,4540,4540,4539,4539,4538,4538,4537,4537,4536,4536,4533,4533,4531,4531,4528,4528,4524,4524,4523,4523,4522,4522,4521,4521,4520,4520,4519,4519,4518,4518,4517,4517,4515,4515,4514,4514,4508,4508,4507,4507,4506,4506,4505,4505,4500,4500,4499,4499,4498,4498,4497,4497,4496,4496,4495,4495,4494,4494,4493,4493,4492,4492,4491,4491,4490,4490,4489,4489,4488,4488,4487,4487,4485,4485,4484,4484,4483,4483,4482,4482,4481,4481,4480,4480,4479,4479,4476,4476,4473,4473,4471,4471,4469,4469,4468,4468,4462,4462,4461,4461,4460,4460,4459,4459,4458,4458,4456,4456,4455,4455,4454,4454,4453,4453,4452,4452,4450,4450,4449,4449,4445,4445,4440,4440,4439,4439,4438,4438,4435,4435,4434,4434,4433,4433,4431,4431,4429,4429,4428,4428,4426,4426,4425,4425,4423,4423,4422,4422,4420,4420,4418,4418,4417,4417,4416,4416,4413,4413,4411,4411,4410,4410,4409,4409,4407,4407,4406,4406,4405,4405,4404,4404,4401,4401,4400,4400,4396,4396,4395,4395,4394,4394,4392,4392,4389,4389,4383,4383,4381,4381,4380,4380,4378,4378,4376,4376,4375,4375,4374,4374,4373,4373,4371,4371,4370,4370,4368,4368,4366,4366,4364,4364,4358,4358,4356,4356,4355,4355,4354,4354,4352,4352,4350,4350,4349,4349,4347,4347,4345,4345,4344,4344,4343,4343,4342,4342,4338,4338,4337,4337,4336,4336,4335,4335,4333,4333,4332,4332,4330,4330,4326,4326,4325,4325,4324,4324,4323,4323,4320,4320,4319,4319,4318,4318,4315,4315,4311,4311,4310,4310,4309,4309,4308,4308,4307,4307,4306,4306,4305,4305,4303,4303,4301,4301,4300,4300,4298,4298,4297,4297,4296,4296,4294,4294,4292,4292,4284,4284,4283,4283,4282,4282,4280,4280,4279,4279,4278,4278,4276,4276,4275,4275,4272,4272,4270,4270,4267,4267,4265,4265,4262,4262,4261,4261,4257,4257,4256,4256,4255,4255,4254,4254,4251,4251,4250,4250,4249,4249,4245,4245,4243,4243,4242,4242,4240,4240,4238,4238,4237,4237,4236,4236,4231,4231,4230,4230,4228,4228,4226,4226,4225,4225,4223,4223,4222,4222,4221,4221,4220,4220,4219,4219,4218,4218,4215,4215,4214,4214,4208,4208,4207,4207,4206,4206,4205,4205,4204,4204,4202,4202,4201,4201,4198,4198,4196,4196,4195,4195,4193,4193,4187,4187,4185,4185,4180,4180,4178,4178,4176,4176,4175,4175,4174,4174,4172,4172,4171,4171,4170,4170,4166,4166,4165,4165,4164,4164,4162,4162,4161,4161,4160,4160,4158,4158,4152,4152,4149,4149,4148,4148,4145,4145,4143,4143,4142,4142,4140,4140,4137,4137,4136,4136,4134,4134,4133,4133,4131,4131,4128,4128,4126,4126,4122,4122,4120,4120,4118,4118,4117,4117,4116,4116,4115,4115,4114,4114,4112,4112,4111,4111,4108,4108,4107,4107,4106,4106,4104,4104,4103,4103,4102,4102,4099,4099,4096,4096,4094,4094,4093,4093,4092,4092,4091,4091,4090,4090,4089,4089,4086,4086,4084,4084,4083,4083,4082,4082,4081,4081,4077,4077,4075,4075,4073,4073,4072,4072,4067,4067,4063,4063,4061,4061,4060,4060,4055,4055,4053,4053,4052,4052,4049,4049,4047,4047,4046,4046,4045,4045,4040,4040,4039,4039,4036,4036,4035,4035,4033,4033,4032,4032,4030,4030,4029,4029,4028,4028,4026,4026,4024,4024,4022,4022,4020,4020,4019,4019,4015,4015,4012,4012,4010,4010,4009,4009,4008,4008,4007,4007,4005,4005,4004,4004,4001,4001,3998,3998,3997,3997,3995,3995,3994,3994,3993,3993,3992,3992,3991,3991,3989,3989,3988,3988,3987,3987,3986,3986,3985,3985,3983,3983,3982,3982,3981,3981,3980,3980,3979,3979,3978,3978,3977,3977,3976,3976,3975,3975,3974,3974,3973,3973,3969,3969,3967,3967,3966,3966,3965,3965,3964,3964,3963,3963,3962,3962,3960,3960,3958,3958,3957,3957,3955,3955,3951,3951,3949,3949,3948,3948,3947,3947,3946,3946,3945,3945,3943,3943,3941,3941,3939,3939,3938,3938,3936,3936,3935,3935,3934,3934,3932,3932,3931,3931,3929,3929,3927,3927,3926,3926,3915,3915,3914,3914,3913,3913,3909,3909,3908,3908,3905,3905,3903,3903,3902,3902,3899,3899,3897,3897,3896,3896,3895,3895,3894,3894,3891,3891,3889,3889,3887,3887,3885,3885,3884,3884,3883,3883,3882,3882,3881,3881,3880,3880,3879,3879,3878,3878,3876,3876,3875,3875,3873,3873,3872,3872,3870,3870,3868,3868,3866,3866,3865,3865,3864,3864,3862,3862,3861,3861,3860,3860,3858,3858,3857,3857,3856,3856,3854,3854,3849,3849,3847,3847,3843,3843,3842,3842,3840,3840,3839,3839,3838,3838,3835,3835,3834,3834,3832,3832,3827,3827,3826,3826,3825,3825,3824,3824,3823,3823,3822,3822,3818,3818,3817,3817,3810,3810,3807,3807,3805,3805,3804,3804,3800,3800,3799,3799,3798,3798,3795,3795,3794,3794,3793,3793,3791,3791,3790,3790,3788,3788,3787,3787,3785,3785,3783,3783,3780,3780,3773,3773,3772,3772,3771,3771,3766,3766,3764,3764,3763,3763,3762,3762,3761,3761,3760,3760,3759,3759,3756,3756,3754,3754,3753,3753,3752,3752,3748,3748,3747,3747,3745,3745,3744,3744,3743,3743,3742,3742,3738,3738,3737,3737,3733,3733,3732,3732,3731,3731,3729,3729,3728,3728,3727,3727,3726,3726,3725,3725,3722,3722,3718,3718,3717,3717,3716,3716,3714,3714,3713,3713,3711,3711,3709,3709,3706,3706,3705,3705,3704,3704,3702,3702,3700,3700,3699,3699,3698,3698,3696,3696,3690,3690,3688,3688,3687,3687,3686,3686,3685,3685,3683,3683,3682,3682,3679,3679,3678,3678,3677,3677,3675,3675,3673,3673,3672,3672,3670,3670,3669,3669,3667,3667,3664,3664,3663,3663,3662,3662,3661,3661,3660,3660,3657,3657,3656,3656,3655,3655,3653,3653,3651,3651,3649,3649,3648,3648,3646,3646,3642,3642,3641,3641,3636,3636,3635,3635,3634,3634,3631,3631,3629,3629,3627,3627,3624,3624,3622,3622,3620,3620,3619,3619,3617,3617,3616,3616,3612,3612,3611,3611,3610,3610,3609,3609,3608,3608,3607,3607,3602,3602,3601,3601,3599,3599,3597,3597,3595,3595,3594,3594,3592,3592,3591,3591,3590,3590,3589,3589,3588,3588,3587,3587,3586,3586,3584,3584,3581,3581,3578,3578,3577,3577,3574,3574,3572,3572,3571,3571,3570,3570,3569,3569,3568,3568,3564,3564,3563,3563,3562,3562,3561,3561,3559,3559,3557,3557,3556,3556,3555,3555,3553,3553,3550,3550,3548,3548,3547,3547,3546,3546,3542,3542,3541,3541,3539,3539,3538,3538,3537,3537,3535,3535,3534,3534,3533,3533,3532,3532,3529,3529,3528,3528,3527,3527,3525,3525,3523,3523,3522,3522,3521,3521,3518,3518,3517,3517,3516,3516,3514,3514,3512,3512,3508,3508,3504,3504,3502,3502,3500,3500,3497,3497,3491,3491,3489,3489,3488,3488,3484,3484,3483,3483,3482,3482,3481,3481,3480,3480,3479,3479,3477,3477,3476,3476,3474,3474,3470,3470,3468,3468,3467,3467,3465,3465,3463,3463,3462,3462,3461,3461,3460,3460,3459,3459,3458,3458,3457,3457,3456,3456,3454,3454,3453,3453,3450,3450,3448,3448,3447,3447,3446,3446,3444,3444,3440,3440,3438,3438,3436,3436,3434,3434,3433,3433,3432,3432,3431,3431,3430,3430,3425,3425,3424,3424,3423,3423,3422,3422,3421,3421,3420,3420,3417,3417,3416,3416,3415,3415,3413,3413,3412,3412,3411,3411,3409,3409,3407,3407,3403,3403,3402,3402,3401,3401,3399,3399,3398,3398,3394,3394,3393,3393,3390,3390,3389,3389,3388,3388,3387,3387,3386,3386,3385,3385,3384,3384,3383,3383,3380,3380,3378,3378,3377,3377,3375,3375,3373,3373,3372,3372,3369,3369,3366,3366,3363,3363,3361,3361,3359,3359,3358,3358,3356,3356,3354,3354,3353,3353,3352,3352,3351,3351,3348,3348,3347,3347,3346,3346,3345,3345,3344,3344,3343,3343,3342,3342,3340,3340,3335,3335,3334,3334,3333,3333,3332,3332,3330,3330,3329,3329,3328,3328,3327,3327,3326,3326,3323,3323,3314,3314,3313,3313,3312,3312,3308,3308,3307,3307,3306,3306,3305,3305,3304,3304,3302,3302,3300,3300,3299,3299,3298,3298,3297,3297,3296,3296,3295,3295,3294,3294,3292,3292,3290,3290,3284,3284,3283,3283,3281,3281,3280,3280,3278,3278,3277,3277,3274,3274,3273,3273,3271,3271,3270,3270,3269,3269,3268,3268,3267,3267,3266,3266,3265,3265,3264,3264,3263,3263,3262,3262,3261,3261,3259,3259,3258,3258,3255,3255,3254,3254,3250,3250,3247,3247,3246,3246,3245,3245,3241,3241,3240,3240,3239,3239,3238,3238,3237,3237,3236,3236,3235,3235,3233,3233,3230,3230,3229,3229,3227,3227,3226,3226,3225,3225,3223,3223,3222,3222,3221,3221,3220,3220,3218,3218,3216,3216,3213,3213,3212,3212,3211,3211,3210,3210,3208,3208,3206,3206,3205,3205,3203,3203,3202,3202,3200,3200,3199,3199,3196,3196,3195,3195,3193,3193,3192,3192,3188,3188,3186,3186,3185,3185,3182,3182,3181,3181,3180,3180,3179,3179,3178,3178,3177,3177,3176,3176,3175,3175,3170,3170,3169,3169,3168,3168,3166,3166,3165,3165,3163,3163,3162,3162,3161,3161,3160,3160,3159,3159,3158,3158,3157,3157,3153,3153,3152,3152,3149,3149,3148,3148,3147,3147,3146,3146,3145,3145,3143,3143,3142,3142,3139,3139,3138,3138,3137,3137,3134,3134,3133,3133,3132,3132,3131,3131,3130,3130,3129,3129,3128,3128,3127,3127,3126,3126,3125,3125,3124,3124,3121,3121,3120,3120,3119,3119,3118,3118,3117,3117,3116,3116,3115,3115,3109,3109,3107,3107,3102,3102,3100,3100,3097,3097,3096,3096,3095,3095,3094,3094,3092,3092,3091,3091,3088,3088,3087,3087,3086,3086,3085,3085,3084,3084,3082,3082,3081,3081,3080,3080,3079,3079,3078,3078,3077,3077,3076,3076,3071,3071,3070,3070,3068,3068,3067,3067,3063,3063,3060,3060,3058,3058,3057,3057,3056,3056,3051,3051,3050,3050,3048,3048,3047,3047,3046,3046,3044,3044,3043,3043,3041,3041,3040,3040,3039,3039,3037,3037,3036,3036,3034,3034,3033,3033,3032,3032,3031,3031,3026,3026,3025,3025,3022,3022,3021,3021,3020,3020,3019,3019,3018,3018,3017,3017,3016,3016,3014,3014,3013,3013,3012,3012,3011,3011,3009,3009,3007,3007,3006,3006,3004,3004,3003,3003,3002,3002,3000,3000,2999,2999,2998,2998,2996,2996,2995,2995,2994,2994,2993,2993,2992,2992,2991,2991,2990,2990,2989,2989,2988,2988,2987,2987,2986,2986,2985,2985,2984,2984,2983,2983,2982,2982,2979,2979,2978,2978,2977,2977,2976,2976,2975,2975,2974,2974,2973,2973,2972,2972,2971,2971,2970,2970,2968,2968,2967,2967,2966,2966,2964,2964,2963,2963,2961,2961,2960,2960,2958,2958,2957,2957,2956,2956,2954,2954,2953,2953,2952,2952,2950,2950,2949,2949,2948,2948,2947,2947,2945,2945,2944,2944,2943,2943,2942,2942,2940,2940,2939,2939,2938,2938,2936,2936,2935,2935,2934,2934,2932,2932,2931,2931,2930,2930,2929,2929,2928,2928,2927,2927,2926,2926,2925,2925,2924,2924,2923,2923,2922,2922,2918,2918,2917,2917,2916,2916,2915,2915,2914,2914,2911,2911,2909,2909,2907,2907,2905,2905,2902,2902,2901,2901,2900,2900,2899,2899,2897,2897,2896,2896,2895,2895,2894,2894,2893,2893,2892,2892,2891,2891,2890,2890,2889,2889,2888,2888,2886,2886,2885,2885,2884,2884,2883,2883,2882,2882,2880,2880,2879,2879,2878,2878,2877,2877,2876,2876,2873,2873,2871,2871,2870,2870,2869,2869,2867,2867,2866,2866,2863,2863,2862,2862,2861,2861,2859,2859,2858,2858,2855,2855,2854,2854,2853,2853,2852,2852,2849,2849,2848,2848,2847,2847,2846,2846,2845,2845,2842,2842,2841,2841,2840,2840,2839,2839,2836,2836,2835,2835,2833,2833,2832,2832,2831,2831,2830,2830,2829,2829,2825,2825,2820,2820,2819,2819,2818,2818,2815,2815,2814,2814,2812,2812,2811,2811,2810,2810,2809,2809,2808,2808,2806,2806,2805,2805,2803,2803,2802,2802,2801,2801,2799,2799,2797,2797,2794,2794,2793,2793,2792,2792,2789,2789,2788,2788,2787,2787,2785,2785,2784,2784,2781,2781,2780,2780,2778,2778,2777,2777,2776,2776,2775,2775,2774,2774,2773,2773,2771,2771,2770,2770,2769,2769,2768,2768,2764,2764,2763,2763,2762,2762,2761,2761,2760,2760,2759,2759,2756,2756,2755,2755,2753,2753,2750,2750,2749,2749,2746,2746,2744,2744,2742,2742,2741,2741,2740,2740,2739,2739,2737,2737,2736,2736,2735,2735,2734,2734,2733,2733,2732,2732,2731,2731,2730,2730,2728,2728,2727,2727,2726,2726,2725,2725,2724,2724,2723,2723,2722,2722,2721,2721,2720,2720,2719,2719,2718,2718,2717,2717,2714,2714,2713,2713,2712,2712,2711,2711,2710,2710,2708,2708,2707,2707,2706,2706,2704,2704,2700,2700,2697,2697,2695,2695,2694,2694,2692,2692,2691,2691,2685,2685,2682,2682,2681,2681,2680,2680,2677,2677,2676,2676,2674,2674,2672,2672,2670,2670,2669,2669,2668,2668,2667,2667,2666,2666,2665,2665,2663,2663,2662,2662,2661,2661,2659,2659,2657,2657,2656,2656,2654,2654,2653,2653,2652,2652,2651,2651,2650,2650,2649,2649,2647,2647,2646,2646,2645,2645,2644,2644,2641,2641,2640,2640,2639,2639,2638,2638,2637,2637,2636,2636,2635,2635,2634,2634,2633,2633,2631,2631,2630,2630,2629,2629,2628,2628,2627,2627,2626,2626,2624,2624,2623,2623,2622,2622,2621,2621,2618,2618,2617,2617,2616,2616,2615,2615,2613,2613,2611,2611,2610,2610,2609,2609,2608,2608,2607,2607,2606,2606,2604,2604,2602,2602,2601,2601,2599,2599,2597,2597,2596,2596,2594,2594,2593,2593,2591,2591,2588,2588,2586,2586,2585,2585,2584,2584,2583,2583,2582,2582,2581,2581,2580,2580,2579,2579,2578,2578,2577,2577,2576,2576,2574,2574,2572,2572,2571,2571,2568,2568,2566,2566,2565,2565,2564,2564,2563,2563,2562,2562,2560,2560,2558,2558,2557,2557,2556,2556,2553,2553,2552,2552,2551,2551,2550,2550,2549,2549,2546,2546,2543,2543,2541,2541,2536,2536,2534,2534,2533,2533,2532,2532,2531,2531,2530,2530,2529,2529,2527,2527,2525,2525,2523,2523,2522,2522,2521,2521,2519,2519,2518,2518,2517,2517,2516,2516,2514,2514,2512,2512,2511,2511,2510,2510,2509,2509,2508,2508,2507,2507,2506,2506,2504,2504,2503,2503,2499,2499,2498,2498,2497,2497,2494,2494,2493,2493,2492,2492,2491,2491,2490,2490,2487,2487,2485,2485,2484,2484,2483,2483,2482,2482,2481,2481,2480,2480,2478,2478,2477,2477,2476,2476,2475,2475,2474,2474,2473,2473,2472,2472,2470,2470,2467,2467,2466,2466,2464,2464,2463,2463,2462,2462,2460,2460,2458,2458,2454,2454,2453,2453,2450,2450,2446,2446,2445,2445,2443,2443,2442,2442,2441,2441,2440,2440,2439,2439,2438,2438,2434,2434,2433,2433,2432,2432,2430,2430,2429,2429,2428,2428,2427,2427,2426,2426,2425,2425,2424,2424,2423,2423,2420,2420,2418,2418,2417,2417,2416,2416,2415,2415,2413,2413,2410,2410,2409,2409,2408,2408,2406,2406,2401,2401,2400,2400,2399,2399,2397,2397,2396,2396,2395,2395,2394,2394,2393,2393,2392,2392,2391,2391,2390,2390,2389,2389,2388,2388,2387,2387,2385,2385,2384,2384,2383,2383,2382,2382,2380,2380,2379,2379,2378,2378,2375,2375,2374,2374,2373,2373,2371,2371,2370,2370,2369,2369,2368,2368,2367,2367,2366,2366,2362,2362,2359,2359,2358,2358,2356,2356,2355,2355,2354,2354,2353,2353,2352,2352,2351,2351,2350,2350,2349,2349,2348,2348,2347,2347,2346,2346,2345,2345,2344,2344,2343,2343,2342,2342,2341,2341,2340,2340,2338,2338,2337,2337,2336,2336,2335,2335,2333,2333,2332,2332,2331,2331,2330,2330,2329,2329,2328,2328,2327,2327,2326,2326,2325,2325,2324,2324,2323,2323,2320,2320,2319,2319,2317,2317,2316,2316,2315,2315,2314,2314,2311,2311,2310,2310,2309,2309,2308,2308,2307,2307,2305,2305,2302,2302,2301,2301,2296,2296,2294,2294,2293,2293,2292,2292,2290,2290,2288,2288,2287,2287,2286,2286,2285,2285,2283,2283,2282,2282,2281,2281,2279,2279,2278,2278,2277,2277,2276,2276,2275,2275,2274,2274,2273,2273,2271,2271,2270,2270,2268,2268,2267,2267,2266,2266,2265,2265,2264,2264,2263,2263,2262,2262,2261,2261,2259,2259,2257,2257,2256,2256,2253,2253,2250,2250,2249,2249,2248,2248,2247,2247,2246,2246,2245,2245,2243,2243,2242,2242,2240,2240,2239,2239,2238,2238,2237,2237,2236,2236,2235,2235,2233,2233,2232,2232,2230,2230,2229,2229,2228,2228,2227,2227,2226,2226,2225,2225,2222,2222,2220,2220,2219,2219,2217,2217,2214,2214,2213,2213,2212,2212,2211,2211,2209,2209,2207,2207,2206,2206,2204,2204,2203,2203,2202,2202,2201,2201,2199,2199,2198,2198,2195,2195,2194,2194,2193,2193,2192,2192,2190,2190,2189,2189,2188,2188,2187,2187,2185,2185,2184,2184,2182,2182,2179,2179,2177,2177,2176,2176,2174,2174,2171,2171,2170,2170,2169,2169,2168,2168,2167,2167,2166,2166,2165,2165,2164,2164,2161,2161,2160,2160,2159,2159,2158,2158,2157,2157,2156,2156,2154,2154,2153,2153,2151,2151,2150,2150,2149,2149,2147,2147,2145,2145,2144,2144,2143,2143,2140,2140,2139,2139,2138,2138,2137,2137,2136,2136,2135,2135,2133,2133,2132,2132,2129,2129,2128,2128,2127,2127,2125,2125,2124,2124,2123,2123,2122,2122,2121,2121,2120,2120,2119,2119,2117,2117,2116,2116,2115,2115,2113,2113,2112,2112,2111,2111,2110,2110,2109,2109,2106,2106,2104,2104,2103,2103,2101,2101,2099,2099,2097,2097,2095,2095,2094,2094,2093,2093,2092,2092,2091,2091,2090,2090,2089,2089,2088,2088,2086,2086,2085,2085,2084,2084,2083,2083,2082,2082,2081,2081,2079,2079,2077,2077,2074,2074,2073,2073,2072,2072,2070,2070,2069,2069,2068,2068,2067,2067,2066,2066,2065,2065,2063,2063,2061,2061,2060,2060,2059,2059,2058,2058,2057,2057,2056,2056,2054,2054,2051,2051,2050,2050,2049,2049,2048,2048,2047,2047,2045,2045,2044,2044,2043,2043,2042,2042,2041,2041,2039,2039,2038,2038,2036,2036,2033,2033,2032,2032,2030,2030,2029,2029,2028,2028,2027,2027,2026,2026,2025,2025,2024,2024,2023,2023,2022,2022,2021,2021,2020,2020,2019,2019,2018,2018,2017,2017,2015,2015,2014,2014,2012,2012,2011,2011,2009,2009,2008,2008,2007,2007,2005,2005,2004,2004,2003,2003,2002,2002,2000,2000,1998,1998,1997,1997,1993,1993,1992,1992,1990,1990,1989,1989,1988,1988,1986,1986,1985,1985,1984,1984,1983,1983,1981,1981,1980,1980,1979,1979,1978,1978,1977,1977,1976,1976,1974,1974,1973,1973,1970,1970,1969,1969,1968,1968,1967,1967,1966,1966,1965,1965,1964,1964,1963,1963,1962,1962,1961,1961,1960,1960,1959,1959,1958,1958,1954,1954,1952,1952,1947,1947,1946,1946,1945,1945,1944,1944,1943,1943,1942,1942,1941,1941,1940,1940,1939,1939,1938,1938,1937,1937,1936,1936,1935,1935,1934,1934,1933,1933,1930,1930,1929,1929,1928,1928,1926,1926,1925,1925,1924,1924,1923,1923,1922,1922,1921,1921,1919,1919,1918,1918,1916,1916,1915,1915,1913,1913,1912,1912,1911,1911,1909,1909,1908,1908,1907,1907,1906,1906,1905,1905,1903,1903,1902,1902,1901,1901,1900,1900,1897,1897,1896,1896,1894,1894,1893,1893,1892,1892,1891,1891,1890,1890,1889,1889,1887,1887,1885,1885,1884,1884,1881,1881,1880,1880,1878,1878,1876,1876,1875,1875,1871,1871,1870,1870,1869,1869,1867,1867,1866,1866,1864,1864,1863,1863,1861,1861,1860,1860,1859,1859,1858,1858,1857,1857,1856,1856,1854,1854,1853,1853,1852,1852,1851,1851,1850,1850,1849,1849,1848,1848,1847,1847,1846,1846,1845,1845,1844,1844,1843,1843,1842,1842,1841,1841,1840,1840,1838,1838,1837,1837,1836,1836,1834,1834,1833,1833,1832,1832,1830,1830,1829,1829,1827,1827,1826,1826,1824,1824,1823,1823,1822,1822,1821,1821,1820,1820,1819,1819,1817,1817,1816,1816,1812,1812,1810,1810,1809,1809,1807,1807,1806,1806,1805,1805,1803,1803,1802,1802,1801,1801,1800,1800,1798,1798,1797,1797,1796,1796,1794,1794,1793,1793,1791,1791,1790,1790,1789,1789,1788,1788,1787,1787,1786,1786,1783,1783,1782,1782,1781,1781,1780,1780,1779,1779,1778,1778,1777,1777,1775,1775,1774,1774,1773,1773,1772,1772,1771,1771,1769,1769,1768,1768,1767,1767,1765,1765,1764,1764,1763,1763,1762,1762,1761,1761,1760,1760,1759,1759,1758,1758,1757,1757,1756,1756,1755,1755,1753,1753,1751,1751,1750,1750,1748,1748,1747,1747,1745,1745,1742,1742,1740,1740,1738,1738,1737,1737,1736,1736,1735,1735,1734,1734,1733,1733,1732,1732,1731,1731,1729,1729,1727,1727,1725,1725,1723,1723,1722,1722,1720,1720,1719,1719,1718,1718,1717,1717,1716,1716,1715,1715,1714,1714,1712,1712,1711,1711,1710,1710,1708,1708,1705,1705,1703,1703,1702,1702,1701,1701,1699,1699,1698,1698,1696,1696,1695,1695,1694,1694,1693,1693,1692,1692,1690,1690,1688,1688,1687,1687,1686,1686,1685,1685,1684,1684,1683,1683,1682,1682,1680,1680,1679,1679,1678,1678,1677,1677,1676,1676,1675,1675,1673,1673,1672,1672,1670,1670,1669,1669,1668,1668,1667,1667,1666,1666,1665,1665,1664,1664,1663,1663,1662,1662,1661,1661,1658,1658,1657,1657,1656,1656,1655,1655,1654,1654,1653,1653,1652,1652,1651,1651,1650,1650,1648,1648,1645,1645,1643,1643,1642,1642,1641,1641,1640,1640,1639,1639,1637,1637,1636,1636,1635,1635,1634,1634,1633,1633,1632,1632,1628,1628,1626,1626,1625,1625,1624,1624,1623,1623,1622,1622,1620,1620,1619,1619,1618,1618,1617,1617,1615,1615,1612,1612,1611,1611,1610,1610,1609,1609,1608,1608,1606,1606,1605,1605,1604,1604,1603,1603,1601,1601,1599,1599,1598,1598,1596,1596,1595,1595,1594,1594,1593,1593,1592,1592,1591,1591,1590,1590,1589,1589,1588,1588,1587,1587,1586,1586,1585,1585,1584,1584,1583,1583,1582,1582,1581,1581,1580,1580,1579,1579,1577,1577,1576,1576,1575,1575,1574,1574,1572,1572,1571,1571,1569,1569,1567,1567,1566,1566,1565,1565,1564,1564,1563,1563,1562,1562,1561,1561,1560,1560,1559,1559,1556,1556,1554,1554,1552,1552,1550,1550,1549,1549,1547,1547,1545,1545,1543,1543,1542,1542,1541,1541,1540,1540,1539,1539,1538,1538,1537,1537,1536,1536,1535,1535,1534,1534,1532,1532,1531,1531,1530,1530,1529,1529,1528,1528,1527,1527,1524,1524,1523,1523,1522,1522,1521,1521,1520,1520,1519,1519,1518,1518,1517,1517,1516,1516,1515,1515,1514,1514,1513,1513,1512,1512,1510,1510,1509,1509,1508,1508,1506,1506,1505,1505,1504,1504,1503,1503,1502,1502,1500,1500,1498,1498,1497,1497,1496,1496,1495,1495,1494,1494,1493,1493,1491,1491,1490,1490,1488,1488,1487,1487,1486,1486,1485,1485,1484,1484,1482,1482,1481,1481,1480,1480,1479,1479,1478,1478,1476,1476,1472,1472,1470,1470,1469,1469,1467,1467,1466,1466,1465,1465,1464,1464,1463,1463,1462,1462,1461,1461,1460,1460,1459,1459,1457,1457,1456,1456,1453,1453,1452,1452,1451,1451,1450,1450,1448,1448,1447,1447,1446,1446,1445,1445,1444,1444,1443,1443,1442,1442,1441,1441,1440,1440,1439,1439,1438,1438,1437,1437,1436,1436,1435,1435,1431,1431,1430,1430,1429,1429,1428,1428,1426,1426,1425,1425,1424,1424,1423,1423,1422,1422,1421,1421,1420,1420,1419,1419,1417,1417,1416,1416,1415,1415,1414,1414,1412,1412,1411,1411,1410,1410,1406,1406,1405,1405,1404,1404,1403,1403,1401,1401,1400,1400,1399,1399,1398,1398,1397,1397,1395,1395,1393,1393,1392,1392,1391,1391,1390,1390,1389,1389,1388,1388,1387,1387,1386,1386,1385,1385,1383,1383,1382,1382,1381,1381,1380,1380,1379,1379,1378,1378,1377,1377,1376,1376,1375,1375,1374,1374,1372,1372,1371,1371,1370,1370,1368,1368,1367,1367,1366,1366,1365,1365,1364,1364,1363,1363,1362,1362,1361,1361,1360,1360,1359,1359,1358,1358,1357,1357,1356,1356,1355,1355,1354,1354,1353,1353,1352,1352,1351,1351,1350,1350,1349,1349,1348,1348,1347,1347,1346,1346,1345,1345,1344,1344,1343,1343,1342,1342,1341,1341,1340,1340,1339,1339,1338,1338,1337,1337,1336,1336,1335,1335,1334,1334,1333,1333,1332,1332,1331,1331,1330,1330,1329,1329,1328,1328,1327,1327,1326,1326,1325,1325,1324,1324,1323,1323,1322,1322,1321,1321,1320,1320,1318,1318,1317,1317,1316,1316,1313,1313,1311,1311,1310,1310,1308,1308,1307,1307,1306,1306,1305,1305,1304,1304,1303,1303,1302,1302,1301,1301,1299,1299,1298,1298,1297,1297,1296,1296,1295,1295,1294,1294,1293,1293,1291,1291,1290,1290,1289,1289,1288,1288,1287,1287,1286,1286,1285,1285,1284,1284,1283,1283,1281,1281,1280,1280,1279,1279,1278,1278,1277,1277,1276,1276,1275,1275,1273,1273,1272,1272,1271,1271,1269,1269,1268,1268,1267,1267,1266,1266,1264,1264,1263,1263,1261,1261,1260,1260,1259,1259,1258,1258,1257,1257,1256,1256,1255,1255,1254,1254,1253,1253,1251,1251,1250,1250,1249,1249,1248,1248,1247,1247,1246,1246,1244,1244,1243,1243,1242,1242,1240,1240,1239,1239,1238,1238,1237,1237,1236,1236,1235,1235,1234,1234,1232,1232,1230,1230,1229,1229,1228,1228,1225,1225,1224,1224,1222,1222,1221,1221,1220,1220,1218,1218,1216,1216,1215,1215,1214,1214,1213,1213,1211,1211,1210,1210,1209,1209,1208,1208,1207,1207,1206,1206,1205,1205,1204,1204,1203,1203,1202,1202,1201,1201,1200,1200,1199,1199,1197,1197,1196,1196,1195,1195,1194,1194,1193,1193,1192,1192,1191,1191,1190,1190,1189,1189,1188,1188,1187,1187,1186,1186,1185,1185,1184,1184,1183,1183,1182,1182,1181,1181,1180,1180,1179,1179,1178,1178,1177,1177,1176,1176,1175,1175,1174,1174,1173,1173,1172,1172,1171,1171,1170,1170,1169,1169,1168,1168,1167,1167,1166,1166,1165,1165,1164,1164,1163,1163,1162,1162,1161,1161,1160,1160,1159,1159,1158,1158,1157,1157,1155,1155,1154,1154,1151,1151,1149,1149,1148,1148,1147,1147,1146,1146,1145,1145,1144,1144,1143,1143,1142,1142,1141,1141,1140,1140,1139,1139,1138,1138,1137,1137,1136,1136,1135,1135,1134,1134,1132,1132,1131,1131,1130,1130,1129,1129,1128,1128,1127,1127,1126,1126,1124,1124,1122,1122,1121,1121,1120,1120,1119,1119,1118,1118,1117,1117,1116,1116,1115,1115,1114,1114,1113,1113,1111,1111,1110,1110,1109,1109,1108,1108,1106,1106,1105,1105,1104,1104,1103,1103,1102,1102,1101,1101,1099,1099,1097,1097,1096,1096,1095,1095,1094,1094,1093,1093,1092,1092,1091,1091,1090,1090,1089,1089,1088,1088,1087,1087,1086,1086,1085,1085,1084,1084,1083,1083,1082,1082,1081,1081,1079,1079,1078,1078,1077,1077,1076,1076,1075,1075,1074,1074,1073,1073,1072,1072,1070,1070,1069,1069,1068,1068,1067,1067,1066,1066,1065,1065,1064,1064,1063,1063,1062,1062,1061,1061,1059,1059,1058,1058,1057,1057,1056,1056,1055,1055,1053,1053,1052,1052,1051,1051,1050,1050,1049,1049,1048,1048,1047,1047,1046,1046,1045,1045,1044,1044,1042,1042,1041,1041,1039,1039,1037,1037,1036,1036,1035,1035,1034,1034,1033,1033,1032,1032,1031,1031,1030,1030,1029,1029,1028,1028,1027,1027,1026,1026,1025,1025,1024,1024,1023,1023,1021,1021,1020,1020,1019,1019,1018,1018,1017,1017,1016,1016,1015,1015,1014,1014,1013,1013,1012,1012,1011,1011,1010,1010,1009,1009,1008,1008,1007,1007,1006,1006,1005,1005,1003,1003,1002,1002,1001,1001,1000,1000,999,999,998,998,997,997,995,995,994,994,993,993,991,991,990,990,989,989,988,988,987,987,986,986,985,985,984,984,983,983,982,982,981,981,980,980,979,979,978,978,977,977,976,976,975,975,974,974,973,973,972,972,971,971,970,970,969,969,968,968,967,967,966,966,965,965,964,964,963,963,962,962,961,961,959,959,958,958,957,957,956,956,955,955,954,954,953,953,952,952,951,951,950,950,949,949,945,945,944,944,943,943,941,941,940,940,939,939,938,938,937,937,936,936,935,935,934,934,933,933,932,932,931,931,930,930,929,929,927,927,926,926,925,925,924,924,923,923,922,922,921,921,920,920,919,919,918,918,917,917,916,916,915,915,913,913,912,912,911,911,910,910,909,909,908,908,907,907,906,906,904,904,903,903,902,902,901,901,900,900,899,899,898,898,897,897,896,896,895,895,894,894,892,892,891,891,890,890,889,889,888,888,887,887,886,886,885,885,884,884,883,883,882,882,881,881,878,878,877,877,876,876,874,874,873,873,872,872,871,871,870,870,869,869,868,868,867,867,866,866,865,865,864,864,863,863,862,862,861,861,860,860,859,859,858,858,857,857,856,856,855,855,854,854,853,853,852,852,851,851,850,850,849,849,848,848,847,847,846,846,845,845,844,844,843,843,842,842,841,841,840,840,839,839,838,838,837,837,836,836,835,835,834,834,833,833,832,832,831,831,830,830,828,828,827,827,826,826,824,824,823,823,822,822,821,821,820,820,819,819,818,818,817,817,814,814,813,813,812,812,811,811,810,810,809,809,808,808,807,807,806,806,805,805,804,804,803,803,802,802,801,801,800,800,799,799,797,797,796,796,795,795,794,794,793,793,792,792,791,791,790,790,789,789,788,788,787,787,786,786,785,785,784,784,783,783,782,782,781,781,780,780,779,779,778,778,777,777,776,776,775,775,774,774,772,772,771,771,770,770,769,769,768,768,767,767,766,766,765,765,764,764,763,763,762,762,761,761,760,760,758,758,757,757,756,756,755,755,754,754,753,753,752,752,751,751,750,750,749,749,748,748,747,747,745,745,744,744,743,743,742,742,741,741,740,740,739,739,738,738,737,737,736,736,734,734,733,733,732,732,731,731,730,730,729,729,728,728,727,727,726,726,725,725,724,724,723,723,722,722,720,720,719,719,718,718,717,717,716,716,715,715,714,714,713,713,712,712,711,711,710,710,709,709,708,708,707,707,706,706,705,705,704,704,703,703,702,702,701,701,700,700,699,699,698,698,697,697,696,696,695,695,694,694,693,693,692,692,691,691,690,690,689,689,688,688,687,687,686,686,685,685,684,684,683,683,682,682,681,681,680,680,679,679,678,678,677,677,676,676,675,675,674,674,673,673,672,672,671,671,670,670,669,669,668,668,667,667,666,666,665,665,664,664,663,663,662,662,661,661,660,660,659,659,658,658,657,657,655,655,654,654,653,653,652,652,651,651,650,650,648,648,647,647,646,646,645,645,644,644,643,643,642,642,641,641,640,640,639,639,638,638,637,637,636,636,635,635,634,634,633,633,632,632,631,631,630,630,629,629,628,628,627,627,626,626,625,625,624,624,623,623,622,622,621,621,620,620,619,619,618,618,617,617,616,616,615,615,614,614,613,613,612,612,611,611,610,610,609,609,608,608,607,607,606,606,605,605,603,603,602,602,601,601,600,600,599,599,598,598,597,597,596,596,595,595,594,594,593,593,592,592,591,591,590,590,589,589,588,588,587,587,586,586,585,585,584,584,583,583,582,582,581,581,580,580,579,579,578,578,577,577,576,576,575,575,574,574,573,573,572,572,571,571,570,570,569,569,568,568,567,567,566,566,565,565,564,564,563,563,562,562,561,561,560,560,559,559,558,558,557,557,556,556,555,555,554,554,553,553,552,552,551,551,550,550,549,549,548,548,547,547,546,546,545,545,544,544,543,543,542,542,541,541,540,540,539,539,538,538,537,537,536,536,535,535,534,534,533,533,532,532,531,531,530,530,529,529,528,528,527,527,526,526,525,525,524,524,523,523,522,522,521,521,520,520,519,519,518,518,517,517,516,516,515,515,514,514,513,513,512,512,511,511,510,510,509,509,508,508,507,507,506,506,505,505,504,504,503,503,502,502,501,501,500,500,499,499,498,498,497,497,496,496,495,495,494,494,493,493,492,492,491,491,490,490,489,489,488,488,487,487,486,486,485,485,484,484,483,483,482,482,481,481,478,478,477,477,474,474,473,473,472,472,470,470,465,465,463,463,462,462,461,461,460,460,459,459,458,458,457,457,456,456,455,455,453,453,452,452,451,451,449,449,448,448,447,447,443,443,442,442,438,438,437,437,436,436,435,435,434,434,433,433,432,432,431,431,430,430,428,428,426,426,425,425,422,422,419,419,418,418,417,417,415,415,413,413,412,412,411,411,409,409,405,405,400,400,0.0],[50623,50623,50623,50504,50504,42936,42936,40877,40877,40192,40192,39423,39423,38387,38387,38105,38105,37413,37413,37231,37231,36966,36966,36956,36956,34695,34695,34499,34499,33113,33113,32973,32973,32737,32737,32306,32306,31865,31865,31732,31732,31494,31494,31058,31058,30851,30851,30577,30577,30329,30329,29898,29898,29549,29549,29505,29505,28975,28975,28531,28531,28104,28104,28052,28052,27965,27965,27417,27417,27386,27386,27321,27321,26924,26924,26850,26850,26682,26682,26671,26671,26558,26558,26554,26554,26550,26550,26341,26341,26227,26227,26214,26214,26213,26213,25901,25901,25837,25837,25825,25825,25634,25634,25621,25621,24960,24960,24948,24948,24908,24908,24820,24820,24771,24771,24731,24731,24621,24621,24534,24534,24495,24495,24488,24488,24430,24430,24413,24413,24282,24282,24270,24270,24256,24256,24225,24225,24213,24213,24073,24073,23984,23984,23920,23920,23901,23901,23823,23823,23765,23765,23196,23196,23136,23136,23105,23105,23004,23004,22982,22982,22867,22867,22855,22855,22744,22744,22585,22585,22543,22543,22431,22431,22421,22421,22369,22369,22258,22258,22231,22231,22214,22214,21994,21994,21989,21989,21987,21987,21947,21947,21880,21880,21838,21838,21719,21719,21695,21695,21656,21656,21520,21520,21371,21371,21358,21358,21306,21306,21053,21053,20951,20951,20950,20950,20948,20948,20656,20656,20649,20649,20580,20580,20547,20547,20517,20517,20443,20443,20436,20436,20429,20429,20388,20388,20387,20387,20289,20289,20264,20264,20152,20152,20090,20090,20086,20086,20083,20083,20077,20077,19877,19877,19869,19869,19805,19805,19750,19750,19745,19745,19632,19632,19605,19605,19583,19583,19521,19521,19459,19459,19276,19276,19274,19274,19266,19266,19233,19233,19208,19208,19184,19184,19060,19060,18851,18851,18828,18828,18826,18826,18723,18723,18688,18688,18623,18623,18395,18395,18363,18363,18301,18301,18287,18287,18266,18266,18253,18253,18219,18219,18202,18202,18187,18187,18174,18174,18120,18120,18044,18044,17988,17988,17985,17985,17964,17964,17950,17950,17949,17949,17939,17939,17911,17911,17903,17903,17896,17896,17887,17887,17854,17854,17838,17838,17792,17792,17772,17772,17702,17702,17685,17685,17668,17668,17569,17569,17493,17493,17489,17489,17448,17448,17418,17418,17271,17271,17250,17250,17164,17164,17150,17150,17020,17020,16951,16951,16947,16947,16921,16921,16900,16900,16856,16856,16848,16848,16809,16809,16794,16794,16776,16776,16741,16741,16687,16687,16674,16674,16640,16640,16614,16614,16609,16609,16591,16591,16590,16590,16584,16584,16486,16486,16483,16483,16465,16465,16453,16453,16449,16449,16445,16445,16428,16428,16402,16402,16368,16368,16334,16334,16296,16296,16287,16287,16262,16262,16236,16236,16224,16224,16220,16220,16192,16192,16181,16181,16166,16166,16107,16107,16070,16070,16004,16004,15978,15978,15972,15972,15954,15954,15843,15843,15835,15835,15823,15823,15740,15740,15689,15689,15656,15656,15616,15616,15595,15595,15589,15589,15581,15581,15558,15558,15550,15550,15508,15508,15494,15494,15481,15481,15473,15473,15459,15459,15453,15453,15401,15401,15398,15398,15360,15360,15348,15348,15294,15294,15266,15266,15262,15262,15250,15250,15238,15238,15215,15215,15195,15195,15190,15190,15165,15165,15146,15146,15091,15091,15088,15088,15063,15063,15055,15055,15022,15022,15020,15020,15001,15001,14962,14962,14953,14953,14935,14935,14908,14908,14880,14880,14856,14856,14832,14832,14829,14829,14818,14818,14814,14814,14803,14803,14766,14766,14740,14740,14733,14733,14731,14731,14722,14722,14701,14701,14675,14675,14669,14669,14624,14624,14612,14612,14606,14606,14596,14596,14594,14594,14570,14570,14569,14569,14561,14561,14560,14560,14542,14542,14530,14530,14529,14529,14502,14502,14493,14493,14485,14485,14469,14469,14456,14456,14437,14437,14429,14429,14420,14420,14396,14396,14395,14395,14375,14375,14366,14366,14346,14346,14340,14340,14339,14339,14318,14318,14296,14296,14289,14289,14255,14255,14179,14179,14124,14124,14088,14088,14037,14037,14031,14031,14028,14028,14011,14011,14009,14009,13989,13989,13973,13973,13962,13962,13958,13958,13955,13955,13921,13921,13897,13897,13894,13894,13880,13880,13851,13851,13847,13847,13811,13811,13772,13772,13763,13763,13748,13748,13739,13739,13726,13726,13696,13696,13665,13665,13658,13658,13655,13655,13609,13609,13603,13603,13594,13594,13579,13579,13578,13578,13552,13552,13542,13542,13540,13540,13539,13539,13520,13520,13495,13495,13493,13493,13471,13471,13466,13466,13447,13447,13446,13446,13441,13441,13431,13431,13430,13430,13429,13429,13420,13420,13414,13414,13393,13393,13389,13389,13386,13386,13381,13381,13379,13379,13355,13355,13344,13344,13331,13331,13316,13316,13302,13302,13290,13290,13289,13289,13258,13258,13248,13248,13235,13235,13216,13216,13203,13203,13192,13192,13177,13177,13167,13167,13155,13155,13149,13149,13140,13140,13133,13133,13130,13130,13126,13126,13113,13113,13111,13111,13107,13107,13087,13087,13083,13083,13077,13077,13076,13076,13054,13054,13029,13029,13001,13001,12990,12990,12989,12989,12976,12976,12975,12975,12953,12953,12946,12946,12942,12942,12938,12938,12916,12916,12891,12891,12887,12887,12874,12874,12871,12871,12868,12868,12864,12864,12856,12856,12855,12855,12817,12817,12811,12811,12806,12806,12741,12741,12730,12730,12711,12711,12693,12693,12675,12675,12668,12668,12658,12658,12637,12637,12625,12625,12617,12617,12611,12611,12601,12601,12597,12597,12592,12592,12590,12590,12584,12584,12574,12574,12562,12562,12559,12559,12552,12552,12549,12549,12542,12542,12537,12537,12517,12517,12500,12500,12495,12495,12489,12489,12487,12487,12477,12477,12473,12473,12472,12472,12456,12456,12438,12438,12409,12409,12393,12393,12392,12392,12388,12388,12386,12386,12370,12370,12362,12362,12359,12359,12358,12358,12355,12355,12352,12352,12350,12350,12337,12337,12313,12313,12310,12310,12308,12308,12303,12303,12276,12276,12269,12269,12260,12260,12258,12258,12247,12247,12235,12235,12233,12233,12217,12217,12190,12190,12178,12178,12161,12161,12155,12155,12150,12150,12149,12149,12148,12148,12141,12141,12068,12068,12067,12067,12066,12066,12063,12063,12058,12058,12041,12041,12010,12010,12001,12001,11995,11995,11992,11992,11979,11979,11970,11970,11967,11967,11953,11953,11935,11935,11932,11932,11915,11915,11879,11879,11877,11877,11846,11846,11829,11829,11820,11820,11816,11816,11772,11772,11771,11771,11767,11767,11766,11766,11765,11765,11738,11738,11702,11702,11694,11694,11693,11693,11691,11691,11687,11687,11685,11685,11649,11649,11629,11629,11610,11610,11605,11605,11578,11578,11556,11556,11553,11553,11540,11540,11534,11534,11532,11532,11517,11517,11507,11507,11501,11501,11499,11499,11493,11493,11491,11491,11478,11478,11474,11474,11473,11473,11470,11470,11461,11461,11445,11445,11444,11444,11438,11438,11421,11421,11417,11417,11398,11398,11396,11396,11381,11381,11380,11380,11377,11377,11362,11362,11357,11357,11356,11356,11338,11338,11309,11309,11308,11308,11305,11305,11303,11303,11296,11296,11295,11295,11270,11270,11259,11259,11258,11258,11245,11245,11244,11244,11242,11242,11233,11233,11227,11227,11223,11223,11214,11214,11205,11205,11196,11196,11192,11192,11189,11189,11187,11187,11184,11184,11182,11182,11181,11181,11178,11178,11096,11096,11092,11092,11089,11089,11079,11079,11073,11073,11065,11065,11061,11061,11054,11054,11050,11050,11047,11047,11046,11046,11034,11034,11030,11030,11028,11028,11027,11027,11012,11012,10999,10999,10995,10995,10993,10993,10980,10980,10974,10974,10972,10972,10969,10969,10965,10965,10959,10959,10957,10957,10951,10951,10941,10941,10892,10892,10883,10883,10882,10882,10879,10879,10875,10875,10870,10870,10868,10868,10867,10867,10861,10861,10860,10860,10825,10825,10824,10824,10823,10823,10816,10816,10812,10812,10808,10808,10802,10802,10795,10795,10794,10794,10788,10788,10784,10784,10778,10778,10777,10777,10776,10776,10772,10772,10766,10766,10765,10765,10750,10750,10741,10741,10737,10737,10730,10730,10712,10712,10711,10711,10709,10709,10698,10698,10697,10697,10694,10694,10689,10689,10642,10642,10636,10636,10632,10632,10631,10631,10625,10625,10623,10623,10617,10617,10614,10614,10610,10610,10609,10609,10606,10606,10596,10596,10591,10591,10585,10585,10572,10572,10567,10567,10565,10565,10558,10558,10552,10552,10547,10547,10539,10539,10533,10533,10526,10526,10512,10512,10511,10511,10502,10502,10494,10494,10480,10480,10477,10477,10475,10475,10465,10465,10464,10464,10454,10454,10453,10453,10441,10441,10429,10429,10422,10422,10410,10410,10409,10409,10406,10406,10403,10403,10402,10402,10397,10397,10396,10396,10389,10389,10386,10386,10380,10380,10373,10373,10370,10370,10347,10347,10345,10345,10344,10344,10336,10336,10335,10335,10334,10334,10323,10323,10322,10322,10321,10321,10317,10317,10311,10311,10308,10308,10307,10307,10294,10294,10282,10282,10268,10268,10261,10261,10259,10259,10258,10258,10255,10255,10251,10251,10246,10246,10245,10245,10243,10243,10242,10242,10241,10241,10232,10232,10229,10229,10228,10228,10226,10226,10213,10213,10210,10210,10198,10198,10184,10184,10173,10173,10166,10166,10150,10150,10146,10146,10133,10133,10123,10123,10101,10101,10095,10095,10082,10082,10080,10080,10078,10078,10075,10075,10068,10068,10063,10063,10061,10061,10057,10057,10055,10055,10051,10051,10048,10048,10043,10043,10042,10042,10041,10041,10034,10034,10029,10029,10028,10028,10025,10025,10014,10014,10012,10012,10007,10007,10003,10003,10002,10002,9986,9986,9979,9979,9973,9973,9951,9951,9944,9944,9939,9939,9937,9937,9936,9936,9932,9932,9927,9927,9923,9923,9922,9922,9920,9920,9912,9912,9890,9890,9875,9875,9870,9870,9850,9850,9842,9842,9840,9840,9836,9836,9801,9801,9785,9785,9776,9776,9774,9774,9771,9771,9770,9770,9761,9761,9759,9759,9756,9756,9750,9750,9738,9738,9735,9735,9713,9713,9712,9712,9690,9690,9678,9678,9670,9670,9667,9667,9662,9662,9657,9657,9652,9652,9641,9641,9639,9639,9629,9629,9617,9617,9613,9613,9612,9612,9598,9598,9597,9597,9595,9595,9574,9574,9565,9565,9559,9559,9553,9553,9551,9551,9550,9550,9548,9548,9540,9540,9539,9539,9528,9528,9508,9508,9505,9505,9502,9502,9499,9499,9494,9494,9483,9483,9474,9474,9467,9467,9461,9461,9459,9459,9456,9456,9449,9449,9443,9443,9440,9440,9434,9434,9433,9433,9428,9428,9425,9425,9422,9422,9420,9420,9414,9414,9413,9413,9409,9409,9407,9407,9406,9406,9396,9396,9394,9394,9385,9385,9371,9371,9363,9363,9359,9359,9355,9355,9349,9349,9348,9348,9343,9343,9340,9340,9336,9336,9320,9320,9314,9314,9304,9304,9290,9290,9287,9287,9286,9286,9282,9282,9280,9280,9275,9275,9258,9258,9248,9248,9241,9241,9240,9240,9232,9232,9221,9221,9216,9216,9213,9213,9208,9208,9198,9198,9184,9184,9182,9182,9180,9180,9179,9179,9148,9148,9146,9146,9128,9128,9113,9113,9110,9110,9107,9107,9106,9106,9103,9103,9102,9102,9092,9092,9084,9084,9082,9082,9081,9081,9055,9055,9052,9052,9042,9042,9038,9038,9034,9034,9031,9031,9019,9019,8997,8997,8992,8992,8991,8991,8988,8988,8987,8987,8981,8981,8977,8977,8972,8972,8966,8966,8955,8955,8948,8948,8946,8946,8921,8921,8920,8920,8918,8918,8913,8913,8911,8911,8909,8909,8904,8904,8898,8898,8889,8889,8886,8886,8885,8885,8883,8883,8882,8882,8880,8880,8866,8866,8861,8861,8860,8860,8857,8857,8854,8854,8852,8852,8831,8831,8829,8829,8828,8828,8826,8826,8819,8819,8807,8807,8805,8805,8801,8801,8791,8791,8779,8779,8778,8778,8772,8772,8771,8771,8770,8770,8762,8762,8759,8759,8756,8756,8748,8748,8743,8743,8739,8739,8728,8728,8727,8727,8726,8726,8724,8724,8723,8723,8720,8720,8717,8717,8714,8714,8708,8708,8702,8702,8700,8700,8699,8699,8691,8691,8688,8688,8687,8687,8685,8685,8678,8678,8672,8672,8667,8667,8662,8662,8657,8657,8656,8656,8655,8655,8648,8648,8646,8646,8643,8643,8638,8638,8630,8630,8628,8628,8627,8627,8623,8623,8622,8622,8619,8619,8616,8616,8612,8612,8607,8607,8606,8606,8602,8602,8596,8596,8590,8590,8582,8582,8581,8581,8579,8579,8573,8573,8572,8572,8565,8565,8561,8561,8558,8558,8548,8548,8546,8546,8540,8540,8535,8535,8526,8526,8523,8523,8515,8515,8512,8512,8502,8502,8497,8497,8488,8488,8481,8481,8479,8479,8475,8475,8468,8468,8463,8463,8460,8460,8456,8456,8454,8454,8453,8453,8450,8450,8449,8449,8448,8448,8447,8447,8446,8446,8441,8441,8436,8436,8433,8433,8428,8428,8414,8414,8412,8412,8404,8404,8402,8402,8400,8400,8387,8387,8386,8386,8382,8382,8381,8381,8379,8379,8369,8369,8358,8358,8351,8351,8349,8349,8341,8341,8337,8337,8334,8334,8333,8333,8328,8328,8322,8322,8321,8321,8320,8320,8318,8318,8316,8316,8315,8315,8312,8312,8309,8309,8300,8300,8295,8295,8294,8294,8286,8286,8277,8277,8274,8274,8269,8269,8257,8257,8251,8251,8246,8246,8242,8242,8237,8237,8234,8234,8233,8233,8228,8228,8222,8222,8220,8220,8219,8219,8208,8208,8193,8193,8192,8192,8183,8183,8180,8180,8154,8154,8153,8153,8150,8150,8146,8146,8144,8144,8138,8138,8137,8137,8131,8131,8113,8113,8110,8110,8107,8107,8105,8105,8103,8103,8095,8095,8093,8093,8088,8088,8086,8086,8081,8081,8079,8079,8077,8077,8076,8076,8075,8075,8069,8069,8066,8066,8065,8065,8062,8062,8058,8058,8057,8057,8051,8051,8036,8036,8025,8025,8017,8017,8014,8014,8012,8012,8011,8011,8008,8008,8007,8007,8004,8004,7998,7998,7994,7994,7989,7989,7988,7988,7986,7986,7984,7984,7982,7982,7981,7981,7977,7977,7975,7975,7968,7968,7966,7966,7958,7958,7945,7945,7937,7937,7935,7935,7932,7932,7930,7930,7929,7929,7928,7928,7924,7924,7923,7923,7919,7919,7917,7917,7913,7913,7906,7906,7899,7899,7895,7895,7890,7890,7879,7879,7878,7878,7874,7874,7869,7869,7864,7864,7862,7862,7852,7852,7851,7851,7848,7848,7835,7835,7832,7832,7831,7831,7830,7830,7827,7827,7824,7824,7818,7818,7817,7817,7815,7815,7814,7814,7811,7811,7808,7808,7802,7802,7801,7801,7786,7786,7785,7785,7776,7776,7748,7748,7746,7746,7743,7743,7735,7735,7733,7733,7732,7732,7731,7731,7729,7729,7726,7726,7723,7723,7722,7722,7717,7717,7715,7715,7711,7711,7705,7705,7696,7696,7695,7695,7685,7685,7683,7683,7678,7678,7677,7677,7669,7669,7667,7667,7656,7656,7654,7654,7653,7653,7650,7650,7643,7643,7631,7631,7630,7630,7627,7627,7626,7626,7623,7623,7609,7609,7607,7607,7604,7604,7602,7602,7599,7599,7598,7598,7589,7589,7577,7577,7574,7574,7573,7573,7570,7570,7567,7567,7560,7560,7558,7558,7557,7557,7545,7545,7541,7541,7540,7540,7539,7539,7538,7538,7537,7537,7533,7533,7524,7524,7523,7523,7521,7521,7519,7519,7517,7517,7510,7510,7507,7507,7501,7501,7496,7496,7489,7489,7485,7485,7479,7479,7475,7475,7474,7474,7470,7470,7469,7469,7467,7467,7456,7456,7455,7455,7452,7452,7443,7443,7441,7441,7432,7432,7431,7431,7430,7430,7424,7424,7423,7423,7420,7420,7419,7419,7415,7415,7412,7412,7411,7411,7409,7409,7396,7396,7395,7395,7393,7393,7385,7385,7384,7384,7380,7380,7377,7377,7372,7372,7361,7361,7360,7360,7358,7358,7355,7355,7351,7351,7350,7350,7347,7347,7346,7346,7344,7344,7342,7342,7339,7339,7338,7338,7337,7337,7326,7326,7321,7321,7319,7319,7318,7318,7315,7315,7314,7314,7307,7307,7306,7306,7305,7305,7301,7301,7300,7300,7294,7294,7293,7293,7292,7292,7285,7285,7280,7280,7279,7279,7278,7278,7277,7277,7276,7276,7273,7273,7269,7269,7265,7265,7263,7263,7260,7260,7257,7257,7248,7248,7247,7247,7239,7239,7237,7237,7230,7230,7229,7229,7222,7222,7218,7218,7214,7214,7208,7208,7203,7203,7189,7189,7187,7187,7180,7180,7179,7179,7169,7169,7168,7168,7163,7163,7158,7158,7157,7157,7156,7156,7152,7152,7150,7150,7149,7149,7147,7147,7142,7142,7136,7136,7135,7135,7129,7129,7125,7125,7121,7121,7120,7120,7117,7117,7116,7116,7113,7113,7109,7109,7107,7107,7106,7106,7103,7103,7102,7102,7094,7094,7091,7091,7089,7089,7085,7085,7084,7084,7082,7082,7081,7081,7076,7076,7068,7068,7067,7067,7065,7065,7063,7063,7062,7062,7059,7059,7057,7057,7056,7056,7048,7048,7044,7044,7043,7043,7042,7042,7039,7039,7036,7036,7030,7030,7027,7027,7023,7023,7022,7022,7019,7019,7018,7018,7017,7017,7016,7016,7014,7014,7012,7012,7006,7006,7000,7000,6988,6988,6987,6987,6984,6984,6977,6977,6976,6976,6970,6970,6969,6969,6968,6968,6966,6966,6962,6962,6959,6959,6954,6954,6948,6948,6946,6946,6940,6940,6930,6930,6929,6929,6928,6928,6927,6927,6926,6926,6924,6924,6923,6923,6920,6920,6914,6914,6913,6913,6907,6907,6906,6906,6905,6905,6903,6903,6902,6902,6899,6899,6898,6898,6896,6896,6894,6894,6893,6893,6885,6885,6884,6884,6883,6883,6882,6882,6881,6881,6880,6880,6877,6877,6875,6875,6873,6873,6870,6870,6868,6868,6861,6861,6857,6857,6854,6854,6843,6843,6842,6842,6839,6839,6829,6829,6826,6826,6822,6822,6814,6814,6812,6812,6809,6809,6808,6808,6806,6806,6796,6796,6793,6793,6788,6788,6787,6787,6786,6786,6782,6782,6777,6777,6775,6775,6752,6752,6746,6746,6745,6745,6737,6737,6736,6736,6733,6733,6721,6721,6716,6716,6709,6709,6708,6708,6707,6707,6706,6706,6703,6703,6701,6701,6700,6700,6696,6696,6694,6694,6693,6693,6692,6692,6691,6691,6690,6690,6685,6685,6684,6684,6681,6681,6680,6680,6679,6679,6678,6678,6674,6674,6672,6672,6667,6667,6665,6665,6664,6664,6662,6662,6661,6661,6653,6653,6643,6643,6642,6642,6641,6641,6638,6638,6636,6636,6633,6633,6630,6630,6626,6626,6619,6619,6614,6614,6610,6610,6608,6608,6607,6607,6604,6604,6603,6603,6601,6601,6598,6598,6593,6593,6592,6592,6591,6591,6588,6588,6587,6587,6576,6576,6570,6570,6568,6568,6567,6567,6566,6566,6564,6564,6562,6562,6561,6561,6555,6555,6554,6554,6552,6552,6549,6549,6544,6544,6536,6536,6534,6534,6532,6532,6530,6530,6529,6529,6526,6526,6525,6525,6522,6522,6521,6521,6519,6519,6518,6518,6515,6515,6513,6513,6511,6511,6507,6507,6506,6506,6505,6505,6504,6504,6498,6498,6497,6497,6496,6496,6495,6495,6493,6493,6492,6492,6491,6491,6490,6490,6484,6484,6482,6482,6480,6480,6476,6476,6472,6472,6465,6465,6464,6464,6462,6462,6461,6461,6456,6456,6450,6450,6449,6449,6448,6448,6445,6445,6444,6444,6436,6436,6434,6434,6430,6430,6427,6427,6425,6425,6421,6421,6411,6411,6409,6409,6407,6407,6401,6401,6399,6399,6396,6396,6390,6390,6389,6389,6388,6388,6385,6385,6384,6384,6380,6380,6373,6373,6372,6372,6368,6368,6361,6361,6359,6359,6357,6357,6356,6356,6355,6355,6349,6349,6345,6345,6342,6342,6340,6340,6338,6338,6337,6337,6331,6331,6326,6326,6325,6325,6316,6316,6315,6315,6314,6314,6313,6313,6308,6308,6306,6306,6305,6305,6303,6303,6300,6300,6299,6299,6297,6297,6296,6296,6295,6295,6294,6294,6289,6289,6287,6287,6285,6285,6275,6275,6271,6271,6269,6269,6268,6268,6262,6262,6259,6259,6258,6258,6257,6257,6255,6255,6253,6253,6252,6252,6251,6251,6249,6249,6248,6248,6247,6247,6245,6245,6244,6244,6242,6242,6240,6240,6235,6235,6233,6233,6232,6232,6228,6228,6227,6227,6223,6223,6222,6222,6221,6221,6220,6220,6215,6215,6212,6212,6210,6210,6209,6209,6206,6206,6200,6200,6197,6197,6194,6194,6192,6192,6191,6191,6187,6187,6175,6175,6171,6171,6166,6166,6163,6163,6161,6161,6159,6159,6154,6154,6153,6153,6151,6151,6148,6148,6146,6146,6143,6143,6134,6134,6133,6133,6130,6130,6128,6128,6127,6127,6123,6123,6122,6122,6121,6121,6120,6120,6119,6119,6117,6117,6116,6116,6114,6114,6113,6113,6107,6107,6106,6106,6102,6102,6101,6101,6099,6099,6096,6096,6091,6091,6089,6089,6086,6086,6081,6081,6074,6074,6066,6066,6059,6059,6058,6058,6056,6056,6055,6055,6053,6053,6052,6052,6047,6047,6045,6045,6038,6038,6036,6036,6032,6032,6023,6023,6013,6013,6007,6007,6006,6006,6004,6004,6000,6000,5997,5997,5995,5995,5994,5994,5992,5992,5988,5988,5986,5986,5981,5981,5980,5980,5979,5979,5974,5974,5967,5967,5966,5966,5965,5965,5963,5963,5961,5961,5958,5958,5957,5957,5956,5956,5954,5954,5953,5953,5945,5945,5944,5944,5942,5942,5941,5941,5939,5939,5938,5938,5937,5937,5933,5933,5930,5930,5923,5923,5922,5922,5918,5918,5913,5913,5911,5911,5909,5909,5901,5901,5897,5897,5896,5896,5894,5894,5890,5890,5886,5886,5884,5884,5880,5880,5879,5879,5873,5873,5872,5872,5871,5871,5869,5869,5867,5867,5864,5864,5860,5860,5855,5855,5853,5853,5836,5836,5833,5833,5832,5832,5829,5829,5820,5820,5819,5819,5818,5818,5817,5817,5811,5811,5809,5809,5806,5806,5802,5802,5794,5794,5789,5789,5787,5787,5783,5783,5780,5780,5776,5776,5774,5774,5772,5772,5771,5771,5770,5770,5766,5766,5765,5765,5764,5764,5763,5763,5753,5753,5752,5752,5749,5749,5748,5748,5745,5745,5744,5744,5743,5743,5742,5742,5739,5739,5735,5735,5733,5733,5729,5729,5725,5725,5724,5724,5723,5723,5722,5722,5714,5714,5710,5710,5708,5708,5705,5705,5704,5704,5702,5702,5701,5701,5699,5699,5698,5698,5697,5697,5695,5695,5694,5694,5693,5693,5690,5690,5684,5684,5677,5677,5676,5676,5673,5673,5672,5672,5670,5670,5669,5669,5668,5668,5667,5667,5662,5662,5660,5660,5655,5655,5654,5654,5653,5653,5651,5651,5650,5650,5649,5649,5645,5645,5644,5644,5642,5642,5641,5641,5640,5640,5639,5639,5638,5638,5634,5634,5633,5633,5631,5631,5630,5630,5628,5628,5626,5626,5625,5625,5621,5621,5620,5620,5619,5619,5611,5611,5604,5604,5603,5603,5602,5602,5601,5601,5599,5599,5597,5597,5595,5595,5592,5592,5587,5587,5586,5586,5583,5583,5582,5582,5580,5580,5574,5574,5572,5572,5570,5570,5569,5569,5564,5564,5561,5561,5559,5559,5558,5558,5557,5557,5555,5555,5553,5553,5552,5552,5550,5550,5549,5549,5545,5545,5543,5543,5541,5541,5540,5540,5533,5533,5529,5529,5528,5528,5526,5526,5524,5524,5523,5523,5522,5522,5520,5520,5515,5515,5514,5514,5513,5513,5512,5512,5504,5504,5499,5499,5498,5498,5497,5497,5496,5496,5495,5495,5493,5493,5490,5490,5489,5489,5487,5487,5485,5485,5481,5481,5480,5480,5479,5479,5478,5478,5476,5476,5469,5469,5464,5464,5463,5463,5461,5461,5458,5458,5457,5457,5456,5456,5451,5451,5448,5448,5447,5447,5444,5444,5443,5443,5442,5442,5441,5441,5431,5431,5430,5430,5426,5426,5423,5423,5422,5422,5420,5420,5417,5417,5416,5416,5415,5415,5411,5411,5410,5410,5409,5409,5403,5403,5394,5394,5393,5393,5386,5386,5384,5384,5381,5381,5379,5379,5372,5372,5370,5370,5366,5366,5362,5362,5361,5361,5353,5353,5351,5351,5350,5350,5343,5343,5339,5339,5336,5336,5335,5335,5334,5334,5329,5329,5328,5328,5326,5326,5322,5322,5321,5321,5317,5317,5316,5316,5314,5314,5312,5312,5309,5309,5308,5308,5307,5307,5306,5306,5305,5305,5296,5296,5294,5294,5293,5293,5289,5289,5288,5288,5285,5285,5280,5280,5279,5279,5277,5277,5275,5275,5271,5271,5270,5270,5267,5267,5265,5265,5264,5264,5256,5256,5255,5255,5254,5254,5253,5253,5252,5252,5250,5250,5246,5246,5245,5245,5244,5244,5243,5243,5241,5241,5238,5238,5236,5236,5232,5232,5231,5231,5223,5223,5221,5221,5219,5219,5218,5218,5214,5214,5213,5213,5212,5212,5209,5209,5208,5208,5205,5205,5204,5204,5203,5203,5199,5199,5198,5198,5197,5197,5195,5195,5191,5191,5186,5186,5184,5184,5181,5181,5180,5180,5179,5179,5178,5178,5177,5177,5174,5174,5172,5172,5171,5171,5169,5169,5166,5166,5164,5164,5162,5162,5161,5161,5160,5160,5159,5159,5158,5158,5157,5157,5155,5155,5154,5154,5153,5153,5148,5148,5147,5147,5146,5146,5143,5143,5138,5138,5137,5137,5133,5133,5129,5129,5127,5127,5126,5126,5124,5124,5122,5122,5121,5121,5120,5120,5116,5116,5111,5111,5109,5109,5107,5107,5104,5104,5103,5103,5102,5102,5101,5101,5099,5099,5098,5098,5097,5097,5096,5096,5094,5094,5089,5089,5088,5088,5087,5087,5085,5085,5084,5084,5082,5082,5079,5079,5077,5077,5076,5076,5075,5075,5074,5074,5069,5069,5066,5066,5063,5063,5062,5062,5061,5061,5060,5060,5058,5058,5056,5056,5054,5054,5053,5053,5048,5048,504
[truncated: 8,253,719 more chars]
